# Grassroots community actors leading the way in the prevention of youth violent radicalization

**DOI:** 10.1371/journal.pone.0239897

**Published:** 2020-10-12

**Authors:** Lídia Puigvert, Emilia Aiello, Esther Oliver, Mimar Ramis-Salas

**Affiliations:** 1 Department of Sociology, University of Barcelona, Barcelona, Spain; 2 Institute of Criminology, University of Cambridge, Cambridge, United Kingdom; 3 Harvard Kennedy School of Government, Harvard University, Cambridge, Massachusetts, United States of America; University of Sao Paulo Medical School, BRAZIL

## Abstract

Violence-free family ties, non-violent peers or attachment to society have been pointed out as protective factors against different types of extremism and violent radicalization by international literature. However, more detail needs to be provided about which specific aspects within these realms (friendship/family/community) are effective in challenging violence and how they operate in practice. Recent research conducted under the framework of the PROTON project (Horizon 2020) has analyzed the social and ethical impacts of counter-terrorism and organized crime policies in six European countries. In this article we discuss some identified common features among practices that, developed by organized actors operating at the local level (e.g.: grassroots-based associations, educational institutions, other type of organized networks for prevention, NGOs), are contributing to preventing youth violent radicalization, a phenomenon of growing concern in Europe and beyond. Standing on a solid rejection to violence, these shared features are the following: a bottom-up approach in setting allies with key stakeholders from the community or/and family members to intervene; the promotion of trustworthy and healthy friendship relationships; debunking the lure surrounding violent subjects (“false heroes”) and violence in the different contexts, especially in the socioeducational one.

## Introduction

In 2018, a total of 129 foiled, failed and completed attacks were reported by EU Member States, a number that decreased significantly after a sharp spike in 2017 [1]. In addition, the suspects were predominantly males, being almost half of them younger than 30 years old, which indicates their young profile [1]. As extremism and violent radicalization have continued to occur worldwide and have spread within Western countries due to ethnic, religious or political factors [2], there is a concern not only at the national and regional level but also at the family level, and other social actors such as schools and public authorities who have witnessed this phenomenon have also become increasingly concerned [3–5].

International organizations such as the European Union (EU), the Organization for Security and Co-operation in Europe (OSCE), or the United Nations Office of Counter-Terrorism have recognized the importance of preventing violent radicalization at the root and urged different sectors of society to take action. In its communication ‘Preventing Radicalisation to Terrorism and Violent Extremism: Strengthening the EU’s Response’ [6], the EU clearly outlined that *effective prevention means involving non-governmental organisations*, *front line workers*, *security services and experts in the field* (p. 4). Moreover, it expressed that EU national strategies to prevent and counter radicalization need to build *trust within and between the communities*, *promoting a better understanding of each other’s sensitivities and problems*, *engaging different sections of society*, *and much more* (p. 4). Therefore, *local actors need to be properly equipped to recognise radicalised behaviour* (…), and *such strategies should engage with families and communities* (p. 6–7). Finally, the communication also states that a multiagency approach through a collaboration of policies, prison and probation services, and social service providers and school communities is a must.

Although the positions of these organizations are clear, many of the existing policies and initiatives oriented towards preventing violent radicalisation still lack a solid evidence-based approach. More evidence is needed to better inform organized actors operating at the local level on how to conduct prevention and approach the problem as well as on how to evaluate the programs’ effectiveness [7]. Although efforts are being made, for instance, in forums such as the *Radicalization Awareness Network* (RAN) in Europe and other forums of a similar nature, more discussion about how to conduct effective prevention and how to raise awareness about the violence that we face in the complex and globalized societies of today is needed. These actions should take into account the hazards posed by violent radicalization, while sharing good practices and discussing the lessons already learned [8–10], as well as those policies that have led to more stigmatization in some way and that have had negative impacts [11–14].

This is the gap that the EU-funded PROTON project (2016–2019), on which this article is based, has aimed to close [15]. Counting with a research consortium constituted by 21 partner institutions from Europe and beyond, PROTON’s main goal was to improve existing knowledge on the processes of recruitment to organized crime and terrorist networks through an innovative integration between social and computational science to obtain more solid data to better inform policies. Thus, drawing on the work done by the CREA-UB team (led by prof. Lídia Puigvert) under the PROTON research, the aim of this article is to present and discuss the features that have been identified among the practices oriented towards preventing different forms of youth violent radicalization and that have shown to have impact on the ground. It should be expressly clarified at this early point that the area of concern of this work is that of the field of *prevention* of youth violent radicalization. As defined by the European Commission, the phenomenon of violent radicalization is a complex process in which an individual or a group embraces a radical ideology or belief that accepts, uses or condones violence, including acts of terrorism. Standing on this, our work engages with prevention of radicalization as that very stage prior to the de facto turning to terrorism, understanding that this process and its evolution (towards either the perpetration of violence, or withdrawing from it), although being of high complexity due to the inter-linked dynamics operating at the micro, meso and macro level, does not remain fixed and unchangeable, hence actions can be put into place to successfully advance prevention.

This explained, it is the intention of this article to contribute empirical evidence and, with the voices of the community agents and grassroots stakeholders, to enrich the scientific discussion about how to advance the prevention of youth violent radicalization. In turn, a more general reflection is brought to the debate, which is linked to those underlying factors that are also present in other situations of violence victimization. Such factors need to be addressed by academic literature on both youth violent radicalization and violence prevention at a more general level. This not solely as a way to create theoretical knowledge that truly grasps the nuances of complex societal phenomenon through which youth violence manifests and perpetuates, but which can also inform future actions and policies on how to advance towards violence-free relationships among youth and good coexistence in democratic societies.

## Literature review

### Radicalization leading to violent extremism as a topic of concern

The concern in Western countries about radicalization and violent extremism comes from not only Islamist-inspired extremism but also from the rising presence, capacity and alarming actions of extremist far-right and far-left parties that have emerged all over the world [16]. Already known forms of political extremism that seemed to be less present for a period of time have re-emerged with more power, achieving political representation at the regional, national and even European levels [17–19]. The emergence of either far-right or far-left parties with neo-fascist discourses indicates the rise of racism, nationalism, authoritarianism and traditional conservative positions in some sectors of European societies and beyond, which can jeopardize the European democratic political project [18, 20, 21].

In this context, in both the EU and the US, there has been a coordinated policy to develop programs oriented towards counterradicalization, prevention of recruitment, and deradicalization [22–24]. Counterterrorist policies and programs have ranged from military strategies more focused on the use of force and active security to other more conciliatory approaches aimed at preventing the growth of violent extremism and developing the countering violent extremism (CVE) approach [24].

Radicalization and recruitment to terrorism are different but interrelated processes. ‘Radicalization to action’ or ‘recruitment to terrorism’ is defined—drawing on existing EU definitions—in general terms as *the unlawful use of violence or threat of violence (…)*, *carried out by non-state actor organizations motivated by religious*, *political*, *or other ideological beliefs*, *that aim to instill fear in and coerce governments or societies in pursuit of their goals (…)* [25, p. 14]. According to the literature review conducted by Wesiburd and colleagues in the framework of the PROTON project, the elements used as predictors for involvement in different types of offending behaviors, such as gang involvement and general violent offending, have been considered risk factors [25, p. 17]. Risk factors solely increase the propensity of offending and do not predict offending as a given fact. However, deepening the analysis of these risk factors can help identify and differentiate the types of individuals who may be at a greater risk of offending behaviors. In the context of the EU, three social risk factors have been identified as more latent, namely, poor integration, institutional trust and collective relative deprivation. At the individual level, it is also relevant that subjects who have criminal histories, who score high on religious fundamentalism and who have radical peers need to be considered as being at the greatest risk [25, p. 96].

### The construction of the violent self: Interactions that count

Existing research carried out over the last two decades on what has been theorized as the *preventive socialization of violence* has mainly focused on adolescents and young adults and has confirmed the primary role of peers as socializing agents, who have an equal or more impact than family relatives [26, 27] According to this line of research, there is a predominant coercive discourse that is widespread in mainstream media and social networks and operative on interaction sites where youth are engaged, which coercively socializes many youth to be attracted towards violence [28]. With this predominant socialization that many youth experience, they are exposed to increasingly more violent situations and peer relationships, which would explain their increasing desensitization to violence in general.

Literature in the field of violent radicalization has also unveiled the core relevance of friendship bonds and peer interactions. In this sense, the ‘bunch of guys’ theory of terrorism, upheld by Sageman [29] and backed by empirical research, shows that the triggering factor to join a terrorist group is based on pre-existing friendship ties and that the evolving group of future perpetrators seems more similar to these networks rather than to a formal terrorist cell with a defined hierarchy and division of labor. In this regard, Sageman’s research has shown that the decision to join a terrorist group is not individual but collective, based on childhood friendship ties. This author also mentions the attractiveness attached to terrorist groups, which makes some young people prone to joining terrorist groups to upgrade their reputation. Other empirical studies have also shown the key relevance of social networks, which are either a pushing or a pulling factor in violent radicalization. A study conducted by the United Nations [30] revealed that a third of the individuals surveyed stated that they were pushed to leave for Syria by a friend or relative. Other investigations observed that having out-group friendships or associations is a protective factor for joining radicalized groups [31], while having more homogenous social networks is positively correlated with radical beliefs and attitudes [32].

In line with the abovementioned studies, the research carried out by Gill, Horgan and Deckert [33] on the sociodemographic network characteristics and antecedent behaviors of lone-actor terrorists provides important insights. Lone-actors do not have a uniform profile, and within the time period leading up to most lone-actor terrorist events, other people generally knew about the grievance that later resulted in the terrorist plot or action. This reveals that for a very large majority of lone-actors, significant others were aware of the individual’s commitment to a specific extremist ideology. Similarly, family and friends were aware of the individual’s intent to engage in a terrorist-related activity, as the offender had verbally told them. Finally, in most cases, the offender had produced letters or other types of public statements prior to the event to outline his/her beliefs, although not explicitly disclosing his/her violent intentions. In all, what authors emphasize is that those people who were aware of the individual’s intent to engage in violence, although they had key information beforehand, did not report the case to the relevant authorities. Other traits identified by this study were that many of these types of criminals were socially isolated and regularly engaged in detectable and observable ranges of activities with a wider pressure group, social movement, or terrorist organization. In addition, lone-actor terrorist events were very rarely sudden and impulsive but rather planned.

As observed, social networks of potential offenders obtain signals that can enable them to identify the individuals’ intention to perpetrate acts of violence. However, in many cases, they feel reluctant to report the potential offenders, partly due to the fear of consequences of doing so or for fear of damaging their relationship with them [34]. Therefore, it is important to raise awareness of the key role of potential offenders’ social networks as bystanders who can be active agents in preventing potential attacks.

### The quest for significance and the need for community resilience

In their analysis of violent political extremism, Kruglanski and colleagues [35] explained that the use of violence can be partly predicted by a set of conditions that evoke a common psychological state of personal insignificance. These authors researched the impact of economic failure and social detachment as specific threats to individual feelings of insignificance. In their analysis of subjects with extremist ideologies, the authors found that individuals who experienced failure at work, who were rejected in social relationships, or who were victims of abuse were more likely to pursue their ideological goals and thus more prone to joining extremist groups.

This is consistent with the work of Lyons [36], who found that poor integration is a significant risk factor for the radicalization of belief. According to this author’s work, immigrants who are at the margins of society and who do not identify with either their heritage culture or the culture of the larger society and who have experienced a significant loss are at the greatest risk of radicalization. In turn, this process is aggravated by exclusion from others in the larger society.

In similar terms, Jasko, La Free and Krugaleski [37] have argued for the “quest for significance theory”, developed in the field of social psychology, to better explain and understand how subjects undergo the process of violent radicalization. This theory identifies three general drivers: *need*, *narrative* and *social networks*. First, this theory explicates that subjects have a need for personal significance, that is, to matter to someone and to have a meaning in one’s life. Second, when this is linked to a violence-justifying ideological narrative, the narrative pushes the subject towards radicalization by delineating a collective cause that can earn the individual the significance and meaning that he or she desires. Finally, a network of people who subscribe to that narrative leads individuals to perceive the violence-justifying narrative as cognitively accessible and morally accepted. Thus, some types of social networks end up legitimizing violence, showing that the presence of radicalized others in the individual’s social milieu should increase his or her likelihood of using violence. This process has been identified in relation to friends and significant others but not with respect to family members. As mentioned in the previous section, this reveals the major impact of friends as a primary source of socialization [29]. As Jasko, La Free and Krugaleski hypothesize, it is likely that nonviolent social connections serve as a protective factor that prevents a person from engaging in extreme violent behavior.

Other studies have investigated the importance of self-esteem and empathy in preventing violent radicalization. Feddes and colleagues [38] performed a longitudinal evaluation of resilience training in the Netherlands as a way of preventing violent radicalization among male and female Muslim adolescents and young adults with a migrant background. Their results indicated that an empathy intervention is successful in countering violent radicalization when aimed at empowering individuals in combination with strengthening inclusion. Based on their investigation, authors explain that the acquisition of a radical belief system comprises four elements, namely, perceiving out-group authorities as illegitimate, perceiving the in-group as superior, perceiving distance towards other people, and finally, feeling alienated and disconnected from society.

In all, as an antidote to the loss of meaning and disengagement, much literature in the field of counterterrorism acknowledges the need to deploy measures oriented towards building what has been popularized as *community resilience* [39–41], that is, forms of better equipping young people and the community as a whole with knowledge about the risks posed by violent terrorism and how to identify them. The importance of these alternatives to security-oriented measures thus lies in their relevance to enhancing social bonding and social bridging as a way of decreasing the risk of violence, as well as in their role of building social connections between communities and institutions or governing bodies. The government’s partnering with community members can provide both systems for early intervention in violent extremism and systems for strengthening bonds and bridging social networks, thus enhancing community resilience [41].

There are scant details on how the prevention practices and programs are specifically being implemented on the ground or on the particular elements underlying them that are making them effective. In turn, there is little research analyzing both prevention practices implemented on the ground and their features, in dialogue with what previous scientific literature has revealed about the risks and protective factors leading to violent radicalization and youth violence prevention in general. With this article, we aim to shed some light on these questions.

## Methods and materials

### The CREA-UB PROTON study

This article uses data from the EU-funded project PROTON (2016–2019) and specifically from the research tasks developed by the CREA-UB team, which focused on analyzing the social and ethical impacts of policies against organized crime (Task 1.2.), and those against terrorist networks (Task 2.2.) in Europe. The PROTON team worked with its own definitions of *organized crime* and *terrorism* [42, p. 1; p. 9], considered as two different phenomena, but in many instances interrelated. For instance, organized crime activities are many times used to fund terrorist networks. In the study commissioned to the CREA-UB team, we collected data through three sources: desk research on scientific literature and gray literature about the impact of policies on terrorist networks, desk research on policy mapping of the policies and protocols in the EU member states regarding terrorism, and qualitative fieldwork.

Framed by the broad research carried out by PROTON and the results obtained by the CREA-UB team in its study, this article narrows the focus on one specific factor, which is the role played by organized actors implementing prevention practices at the local level (e.g., grassroots-based associations, schools, other types of organized networks for prevention, NGOs) and how in some cases they set the conditions on the ground to create both contexts and relationships that successfully challenge and prevent youth violent radicalization. Embedded in these realms—contexts and relationships—the data presented in this article address the following research question: *Are there either specific practices that organized actors operating at the local level engage in or common features that underlie these practices that contribute to enlarging the practices’ impact on the prevention of youth violent radicalization*? To address this question for this article, the data gathered by CREA-UB PROTON study related to the analysis of the societal and ethical impact of policies against terrorist networks (PROTON Task 2.2.) [43] were reanalyzed and discussed within the specific context of the literature on violent radicalization, youth violence, and violence prevention, specifically elaborated and synthesized for this work.

#### The use of communicative methodology of research in the PROTON study: Unveiling the potential of transformation

The CREA-UB study was conducted within the frame of the *communicative methodology of research*, which is oriented towards the social transformation of reality [44]. The communicative approach is in line with the dual nature of current social theory, which accounts for the influence of systems and social actors and the interactions between both. In line with this approach, novel knowledge is both uncovered and created by means of an intersubjective discussion between the researcher and the researched subjects. Throughout this action of knowledge creation, researchers bring current scientific insights on the topic that is being discussed—the systems’ views—and the researched subjects bring their *lifeworlds* [45], that is, their lived experiences, memories, perceptions, and views. In the case of the PROTON project, when conducting the fieldwork, the research team engaged in a conversation with the subjects interviewed, explicating the insights from the fields of sociology, criminology, or social psychology on violent radicalization and youth violence, and later opening this discussion to incorporate the *lifeworlds* of the subjects themselves—their experiences in terms of meaning creation and loss, perceptions of racism and its impact on violence, the role of sexual-affective relationships, the barriers that organized actors encounter when doing their work—and other elements relevant to violent radicalization processes.

In this way, the communicative methodology sets the ground for a two-way discussion between researchers and researched subjects about the topic under analysis. In a final stage, knowledge created within this process not solely informs the body of research about youth violent radicalization and its prevention, specially capturing those ‘sites of transformation’ but also contributes to the practice done by actors operating in the ground. By sites of transformation, we refer to those contexts and relationships that are making possible to challenge this intricate phenomenon, which many times are overlooked by scientific research. In the case of the PROTON research and other investigations done in the past using the communicative approach [46], as researchers rooted in current complex societies, we are moved not solely to explain the ‘why’ of the research problem under analysis but also to those conditions and relationships that can make emerge alternative realities and social change.

#### Study setting and participants

Under the CREA-UB study framed within the largest PROTON project fieldwork was conducted in six European countries: Italy, Germany, the Netherlands, Romania, Spain and the UK. In many instances, fieldwork was carried jointly for aspects related to organized crime, and terrorism, as extensive literature and legal documents have already recognized the straight linked among both. The mentioned countries were selected following two main criteria. First, for their diversity in terms of geographical distribution (North West, South West, North East, South East of Europe). And second, for being countries which have been the host or in-transit scenario of long-lasting organized crime activities, and/or which have been in the spotlight of terrorist attacks in the last decades, what led them to develop specific legal and prevention infrastructure. In each of these national settings, fieldwork participants were selected according to their *type of profile*:

1. experts in the field of organized crime and violent radicalization: scholars or, in some cases, individuals in charge of institutional organizations addressing organized crime or violent radicalization.
2. stakeholders and organized actors operating at the local level who are developing programs and practices specifically aimed at preventing violent radicalization, and in some particular cases, those who are working on the prevention of youth violence in general. Most of these actors are grassroots-based associations, NGOs or charities, organized networks for prevention, or educational institutions. All of them can be consulted in the “Annex I. Fieldwork study participants”, published in the PROTON general report on terrorism [25, p. 246]. As a rule, the name of these institutions is disclosed, but not the personal names of the people interviewed from each institution.
3. end-users, i.e., those individuals who are directly or indirectly affected by policies and actions oriented towards preventing organized crime and violent radicalization. In this category, family members of youth who had been involved in organized crime networks and families and other community members who had participated in educational centers as volunteers working on prevention were included. They provided information on the impact of the policies and programs om their daily lives.

In addition, six different domains of actions of the experts, stakeholders and end-users were considered: media, prison, migration, religion, education and neighborhoods.

#### Data collection

Data collection phase including recruitment of participants took place between March 2017 and November 2017. A dynamic snowball strategy was used for the recruitment of participants, contacting first experts and stakeholders, and then end-users. Experts were identified mainly through published international literature and policy reports on the topic. Interviews were first launched with experts who also collaborated in identifying stakeholders eventually recruited for the fieldwork. CREA’s networks of contacts of community partners across Europe operating on the ground (with whom researchers had already collaborated in past international scientific investigations) were also of assistance to identify key stakeholders working on the field of prevention of youth violence radicalization as well as to triangulate already selected contacts who would be potential nods for subsequent research participants. Once experts and stakeholders were identified, and recruited, end-users were identified with the former’s collaboration and then recruited for participation in the fieldwork.

Semi-structured interviews were conducted with the group of experts and stakeholders. In the case of the end-users, communicative daily life stories were carried out. Fieldwork was led by different members of the research team depending on the language preferred by research participants (all six languages were covered by members of the research team). It was conducted both online (using Skype) and in-person. In person fieldwork was done in Spain, the UK, and Germany, convened most of the times at places of convenience for the interviewees such as headquarters of their organizations. Online fieldwork was conducted with research participants from Romania, the Netherlands and Italy. The protocols used were similar in all six countries but considered the context and national legislation for both counterterrorism and organized crime. A detailed account of qualitative protocols used can be found in Annex II. Guidelines for Qualitative Interviews, of the PROTON general report on terrorism [25].

In the overall research conducted by the CREA-UB team, the fieldwork carried out in the six countries included 34 interviews with experts, 29 interviews with stakeholders, 8 communicative daily life stories with end-users, and 7 communicative focus groups with stakeholders.

Given the main focus of this article, we have mainly used the data from the 29 interviews conducted with the group of *stakeholders* in Spain, the UK, Germany and the Netherlands (Italy and Romania were countries in which fieldwork was mostly focused on organized crime). Additional data from the experts and end-users are considered solely as a complement to the information provided by the stakeholders.

#### Data analysis

Once all data were collected and analyzed for the general PROTON research, for this study, we focused the analysis on the following defined research question: *Are there either specific practices that organized actors operating at the local level engage in or common features that underlie these practices that contribute to enlarging the practices’ impact on the prevention of youth violent radicalization*?

All the practices aimed at preventing and addressing youth violent radicalization described by the actors operating on the ground were carefully reviewed by the research team. Particular examples provided by specific end-users and experts were also considered when specifically referring to those practices implemented on the ground.

Once the practices were identified, a new coding scheme was constructed, taking into account the emerging categories from the scientific literature in the field of youth violent radicalization and violence prevention. In turn, the data were also interpreted on the basis of the communicative approach, namely, according to the transformative and exclusionary dimensions (See Table 1 below). As for the transformative elements, were defined the elements as those contributing to the prevention or effective addressing of youth violent radicalization. The exclusionary elements were identified as the elements hampering the prevention of youth violent radicalization or those contributing to pushing the youth into violent radicalization. In the following table, the coding scheme used is shown.

**Table 1:** Coding scheme: Elements with which the practices implemented at the grassroots-level either for preventing or addressing violence/violence radicalization were analyzed.

	*Transformative elements*	Exclusionary elements
Types of allies gained (community actors, families, institutions, others)		
Sexual-affective relationships and enhanced social relationships		
Treatment of and discourses surrounding violence: glamor, disdain, honor, others		
Narratives related to violence and narratives opposed to violence		
Direct prevention of youth radicalization/prevention of violence in general		
Meaning and significance (real alternatives: employment opportunities, school opportunities, other ways of meaning construction)		

#### Ethical issues

All research activities conducted within the framework of H2020 PROTON research project, underwent through a strict ethical screening and evaluation. Framed in this, the CREA-UB study passed through and was approved by three different Ethics Committees which screened its evaluation in an *ex-ante*, *in-itinere*, *and ex-post* phase. First, the compulsory Ethical Review in the evaluation of Horizon2020 (EU Framework for Research and Innovation) research projects. Second, PROTON’s Ethical and Legal Advisory Group (ELAG), which was in charge of independently reviewing the activities and final outputs of the PROTON project. ELAG was constituted by four members: Lisa Claydon, Senior Lecturer in Law at the Open University Law School at Walton (UK); Azrini Wahidin, Associate Dean, Research and Innovation, in the School of Social Science, Business and Law, Teesside University (UK); Ziad Abdeen, President of the ethic committee at Al-Quads University (East Jerusalem, Palestine), and Hassan Ehsan Masood, Professor at the Research Professional News and Imperial College, London (United Kingdom). Finally, the CREA-UB research study was also overviewed by the Commission of Bioethics of the University of Barcelona (Institutional Review Board: IRB 0003099).

Each study participants were provided with consent forms and agreed to the conditions and terms of the investigation. When referring to organizations, the real name of the entity is disclosed. However, synonyms are used in the case of personal names.

## Results

This section presents and discusses the findings of the analysis conducted, structured in four subsections. The initial three sub-sections are related to the transformative dimension of our analysis; each of them approaches and discusses a common feature shared by those prevention practices identified in the analyses, and which are having positive impact. A core theme underlying these shared features is their solid embedding in the rejection of violence. The four subsection reports the findings related to the exclusionary dimension of our analysis. Although the exclusionary dimension lays beyond the scope of this work, a mention to the most common elements needs to be made, specially at the light of how they affect the work that stakeholders do on the ground.

### Building allies with diverse community actors to reject violence

The organized actors who were interviewed, who were from different nationalities and even worked in different social realities and confronted different levels of violence, emphasized that a core practice when detecting and disrupting networks of violence while working on the ground is making allies with significant others from the communities. Nonviolent peers and family networks such as parents, siblings, or even other relatives and closed members of the community who reject violence, are key actors in this regard. In what follows, some examples are discussed.

Colin, a senior worker from the charity Active Change Foundation [47] emphasized that many of the referrals the charity receives come from family members. He explained that the family is the first to note whether its son or daughter is adopting an extremist ideology that can lead to violence:

“Because we have a good relationship with the community, the father or the mother may come to the center and ask for help. When the family comes and asks for help, it is because the boy or the girl is flirting with violence… They know that they are changing their friends, they know other things and do not know how to cope with that… There is always something (…)” (Male stakeholder, UK)

The Active Change Foundation (ACF) is located in London, and it was created to protect and safeguard young people and families from unrest and violence in all forms. It works in different ways, by raising public awareness, challenging conflict through dialogue, developing resilience through training and by providing direct support services. In 2014, the ACF released the YouTube campaign “#NotInMy Name” (#NIMN) to fight back and show people “the true face of Islam”, showing that ISIS has nothing to do with what Islam. The campaign went viral and had over 885,000 views on YouTube and over 6.6 million tweets using the hashtag. This campaign also reveals the type of community-based work developed by the ACF, which is based on working hand in hand with peers to reject violence and on a bottom-up approach.

The relevance of making allies with key actors when preventing and addressing youth violent radicalization is also stressed and exemplified by activists from Mothers For Life [48]. This is a global network of parents who have experienced violent jihadist radicalization within their own families. These family-relatives have united with the mission to provide others in a similar situation with the safety of a secure network that can help them, coordinating activities such as guidance and counselling. Mothers for Life are currently present in 12 countries worldwide and the activities are carried out “by parents for parents”. In this study, we interviewed a member of the network, Mary, whose son left for Syria in 2012. Mary explained that many times policies oriented towards preventing and addressing youth violent radicalization do not count on families but push them out:

“The families end up being seen as nobodies, not counting and having no voice, either in the prevention of the problem or in the detection or the deradicalization process”. (Female stakeholder, international organization)

To counteract this, Mothers for Life members attend schools, intervene and speak with young adolescents, and assist parents and families affected by violent jihadi radicalization by placing them in touch with experienced councilors on counterradicalization and deradicalization, by connecting them with other families to talk about the fears, and by providing assessment. As an interviewee explains,

“We do prevention work in schools with youth (…) [although] it is very difficult to get into schools because of the political factors, we struggle, unless we have some very motivated teachers that take the initiative to push it forward and get us into the schools (…) We need to work in teams, with community members on side with the authorities and politicians making the decisions.” (Female stakeholder, international organization)

In the above quotation, Mary expresses that it is a must to involve the community members when doing prevention, involving the collaboration of teachers, family members, authorities, and policy makers. Each side intervenes from its position, and the sides collaborate with each other.

In Germany, the case of HAYAT was analyzed, and the stakeholder from this organization also valued the relevance of making appropriate allies. HAYAT [49], which means “Life” in Turkish and Arabic, was the first German counseling program for persons involved in radical Salafist groups or persons who were on the path towards violent jihadist radicalization, as well as those traveling to Syria and other combat zones. Abdela, a social worker from HAYAT, explained that as in the case of the Active Change Foundation, in many instances, the parents are the ones who come to them, notice possible cases of radicalization and ask for help on how to proceed:

“We only work in the context [of young persons], but the parents come to us. […] we make a sketch of the family, i.e., what role does the adolescent have in the family, what are the relationships (…) And then where does this lead him, in what direction, is there even a radicalization? (…) We try to resolve these conflicts and find a trustworthy balance and then the parents can look into it.” (Male stakeholder, UK)

The HAYAT community stakeholder interviewed mentions two interlinked factors when performing prevention. First, it is important to involve the parents and work directly with them when there is a potential case of youth radicalization. Second, it is important to rebuild a relationship and a bond with the young person who is undergoing this process. The interviewee adds the following:

“You need this bond, you cannot deradicalize a person without this bond. So, it is relational work. But we don’t do this, because we don’t work with the youth directly, we only work with the parents who already have this bond, which is broken, and we try to repair it.” (Female stakeholder, Germany)

Another of the initiatives explored was the Diamond program, developed by SIPI, the Foundation for Integration and Intercultural Participation in the Netherlands. SIPI is a grassroots organization founded by highly educated Moroccan women, with more than 10 years of experience working with youth who have been radicalized. For our study, we interviewed Karen, a worker from SIPI who explained the Diamond intervention. This coaching program is aimed at preventing Islamic radicalization by working with a community- and family-based approach. Its target is young adults with double ethnic identities who have experienced difficulties coping with these identities and who are at risk of losing ties with the mainstream society, that is, those youth who are searching for identity, who need a meaning in life, or who have been identified as lacking independent thinking. Diamond consists of a one-year individual coaching program, in which there is weekly contact in the natural surroundings of the young people. In this way, Diamond uses an informal approach aimed at creating trust and involving different community agents, from community members to family or school members and friends. The trainer who provides the training shares the same background as those who attend the program.

By means of involving different key actors relevant to youth who are at risk of radicalization and who are searching for identity, this initiative brings another key topic to the discussion, that is, the idea that many times in the process of violent radicalization other problems emerge. As a way of dealing with the loss of meaning, the youth search for a self-justified need for justice, romantic adventures, negative social influences, or similar ideas. Another feature of the program, as explained by the interviewee, is that those minors who participated in this coaching program were mainly identified by the school, the police, their parents, or other key figures. Additionally, the success of the Diamond coaching program in building meaning for youth at risk of radicalization depended on the inclusion of community and family members as a core asset. Through this initiative, parents had a chance to obtain guidance on how to emotionally support their child and on how to discuss norms and values.

Nonviolent allies made on the ground in the prevention and addressing of violent radicalization are not only limited to family or peer circles but extend to other agents in the community. An example of this is the work developed by the local Dutch National Police. The radicalization commissioner for one of the major cities in the Netherlands and a senior professional in the field who was interviewed in our study reflected that those agents working at the local level are the ones who face the conflict first-hand. The neighborhoods and cities have become the grounds on which the battles are fought, either to reject violence and create bonds of trust among different communities or to solely disrupt networks. He explained how counterterrorism is led at the local level by the police:

“The important issue is the balance between repression and prevention. Counterterrorism activities can have bad consequences (…) We invest in alliances with all groups at three levels: the neighborhoods, the police officers at the team level within the police, and the city (…) Also, [attention needs to be put on] focusing on recruiters, it is important to get signals from the society on recruitment. We collect many signals from allies, so that we can act immediately when the radicalization is beginning.” (Male stakeholder, The Netherlands)

The evidence analyzed in this section reveals how allies are built on the ground by community actors deploying prevention practices. These allies can be family members or other relatives, teachers and other core actors in educational centers, institutional agents such as the police, and many others.

### Trustworthy and healthy friendship relationships: “Real friends do not hurt”

Another feature identified across the different practices studied is their focus on re-establishing nonviolent trustworthy relationships among the youth at risk and their emphasis on working on defining a healthy friendship. The participants interviewed explained that to be able to have a dialogue with the youth at risk of radicalization who are in many cases *flirting with* extremism—in the words of one of our interviewees—first, ties of trust need to be created to then be able to openly discuss healthy friendship relationships free of any type of violence and coercion.

One of our interviewees, Peter from the St Giles Trust Foundation [50], reflects upon these two factors. The St Giles Trust Foundation is an organization based in London that provides services for individuals affected by issues such as an offending background, homelessness, addiction or gang involvement. Since a few years ago, this organization has collaborated with the association ConnectFutures in the UK [51], working specifically on the prevention of violent radicalization. Peter explained that a valuable aspect of their approach when first establishing ties of trust with the youth on the ground is that many of their staff share similar background characteristics with their clients; they are people who might have previous criminal convictions or who might have been involved in gangs themselves. He argues that practitioners need to empathize with the people with whom they work while also providing tools to address the often underlying problems, such as a situation of material deprivation, lack of employment, lack of housing, or others.

The other factor that he mentions is that practitioners need to identify signals that reveal that an individual is getting trapped in a circle of violence. Being a practitioner himself, when talking with him, he raised the following clear questions: When is it that the mindset of youth changes? How can we prevent the problem from occurring? He explained that there are key moments, such as when there is an overdose, when the individual becomes involved in a shooting, is almost killed and is at the hospital, that are turning points for an urgent intervention aimed at stopping the problem from developing further. However, organized actors working on the ground need to be faster in identifying even earlier moments. According to him, it is these very moments when signals are derived from changes in the networks of friends, from specific violent behaviors or from specific interactions that are key and need to be captured by bystanders.

Similar to the case of the St Giles Trust Foundation, Safer London is a charity operating in the city of London that works to prevent and address gang violence, vulnerability and sexual exploitation and that also works in the field of violent radicalization prevention. For our study, we interviewed Elisabeth, an experienced senior professional who has worked for many years with charities and who has supported disadvantaged communities. She explained that one aspect on which the charity’s programs focus on the most is on re-establishing the networks of trust of those youth who have been victims of gang crime or who are at risk of violent radicalization. According to her, many youth often become coercively involved in violent networks without realizing that those people whom they think of as their friends are not their friends. Once they are inside, it is a loop, as many of them also end up being the perpetrators of violence. Based on this, there is a need to guide the youth in distinguishing the healthy and toxic interactions among those whom they consider their friends. Elisabeth explained that because they are aware of the deep harm that gang crime and other similar types of exploitation such as violent radicalization cause to youth social-affective relationships, they have developed a program particularly focused on discussing what a healthy relationship free of violence is:

“One of the questions we asked them was ‘how many friends do you have when you are in serious trouble and whom you can call upon when you are in trouble?’ And the girls would say “ooh, I’ve got 10… 8…” So, three months later, when we do this same questionnaire again and we ask the same question, often the answer is “none”. And that is an indication for us whether they realize that these friendships were actually not appropriate friendships. (…) So, learning about what is a healthy relationship, is understanding that violence is not a part of a healthy relationship (…)” (Female stakeholder, UK)

In this case, the topic of what is a violence-free relationship is particularly treated in sessions addressed towards girls who have been sexually exploited and many times recruited to gangs and other types of organized crime networks by people whom they considered their friends. The links she made when talking about prevention of violent radicalization is that many times girls join extremist groups through fake friends and by following them. Two key elements are clearly observed in this: the key role that peers plays in processes of both radicalization and recruitment, as well as how gender underlies these dynamics. This will be discussed in the following section.

From a different viewpoint, an end-user interviewed for the study explained this very aspect mentioned by the community organizer from the UK. This is the case of a Dutch adult male who converted to Islam, radicalized and fled to Syria, and who is now deradicalized and back in the Netherlands. He currently invests most of his time doing prevention workshops in schools and other organizations mainly working with youth. When talking with him, he reflected on the importance of always having and keeping real friends. He reflected on the idea of how in the process of radicalization real affective relationships are deeply eroded and, in most cases, finally broken up.

“Many times, they (radicals) talk about burning the bridges behind you. And I think that this is a beautiful way of describing it [what a radical group is]: You go off a bridge, you go to an extremist group, and when you burn down the bridge you cannot come back anymore. You cannot come back to your friends, to your family, and that is a problem that you see with a lot of radicals—Salafists, extreme-rights, others…” (Male end-user, the Netherlands)

He observed that real friends can be lifesavers, truly acting as a protective barrier. Prevention campaigns need to be oriented towards this and addressed the building of a ‘big network of prevention’—in this end-user’s own words, a network in which people can act as upstanding agents:

“(…) What we have to do in Europe is build a big network of prevention, and we should help people know how to recognize extremism, how to deal with extremist organizations. And we can do it in schools, we can do it in sports clubs, almost everywhere … on television… we can do much more (….)” (Male end-user, the Netherlands)

As observed above, the practitioners working on the ground are aware of the deep impact that peers have on youth. Networks of friendships are crucial in processes of gang involvement and violent radicalization, and the practitioners involved in prevention note that this is something that needs to be approached when doing prevention; otherwise, there is a high risk of failure. Front line workers and practitioners working on the ground engage with the youth at risk of radicalization not only by approaching the material problems that the youth face but also by helping them keep in mind that they need to discuss behaviors and think of themselves in relation to their close friends, bringing in this element to make the youth realize to what extent these interactions are healthy or not. A community stakeholder from Active Change Foundation elaborated this aspect in a very illustrative way: “You have stronger bonds with your friends rather than with your father or your mother when you are an adolescent; therefore you follow what your friends do or your friends do what you do (…)”. Those practices that are seriously committed to building community resilience bring the topic of friendships rooted in the rejection of any type of violence to the forefront of the discussion.

### Going beyond the lure of violence: Aggressors as cowards, not as heroes

The third feature shared by the initiatives studied is an element that, although identified and recognized by the organized actors interviewed and working on the prevention of youth violent radicalization and youth violence in general, is many times raised as a side factor. This is the lure of violence and of violent individuals, which is an underlying aspect of the socialization of many youth, especially due to what was mentioned in the previous sections, i.e., the existence of a coercive dominant discourse that links attractiveness with violent attitudes and behaviors.

When asked in our fieldwork whether this attraction towards violence and individuals with violent personality was recognized among the youth falling into violent radicalization, most of the stakeholders interviewed identified this element. They noted that youth involved in these networks end up being victims of exploitation, and that they sometimes become the exploiters themselves, thus getting trapped in a vicious circle of violence. The stakeholder interviewed from the local Dutch National Police at the Netherlands pondered about this issue when reflecting about the reasons why young women and girls who have sexual-affective relationships with males involved in organized crime or terrorist networks are reluctant to collaborate with police authorities:

“Girls are victims, but they are also facilitators. When they are facilitating one of the members of the organized crime networks we also investigate their role… And many times I have the feeling that they are not in it voluntarily, but most times they don’t want to talk about it, so it is very hard to get an idea about if it is their own idea or if they are forced to do certain things. So it’s hard (…) Many times they get involved through boyfriends… most women we see in our investigation is that they are girlfriends of the criminals… we also see aunts that play a role… There are family ties and relationships (…) [When investigations come] most times they keep protecting them (Male stakeholder, The Netherlands)

This element was also approached in the interview with Colin, the stakeholder from Active Change Foundation in London, when talking about gender and the role and status of girls in gang related issues and violent extremism. His observation -the one of someone who has been “for a long time in this game”- illuminates the fierce and coercive environment which girls navigate:

“Many girls who have had babies with these gang boys, and then boys have gone into prison, and he’s come out, he’s left her, …she stuck… when she thought that he loved her, and then she found out that she was just a passed in face. Who really wants a girl with “no class”? If that makes any sense (…) If you have been in this game long enough you see…” (Male stakeholder, UK)

In all, the challenge that many of the discussions with the practitioners revolved around is how to create a real transformative and sustainable alternative to contest this attraction to violence. In some of the practices studied, the relevance of this factor was more conscious, and the initiatives were designed by taking this into account. In other practices, although this was noted, this factor was not entirely discussed and broached with the youth. It was somehow a taken for granted element, understood as consequence of the situation of material deprivation of the individual rather as something that deserves to be tackled as a single risk factor. In the fieldwork conducted we discussed, for instance, how on many occasions, the terrorist perpetrators and terrorist acts have been portrayed by mass media as glamourous and attractive [52]. An expert interviewed reflected on this factor in relation to the ISIS case:

If you look at the ISIS propaganda, large parts of it are very violent. They consciously attempt to appear like a very violent computer game, but you have to see that there are different types of recruitment. People are interested in joining these groups for many different reasons (…) (Male expert, Germany)

What this interviewee stressed is that the violence exalts and impresses ISIS members, who join this type of a group for a combination of reasons. However, he agrees that the search for violence and its attractiveness act as a pulling force on many youths.

Elisabeth, a stakeholder of Safer London, identified this lure of violence among youth involved in organized crime and in processes of violent radicalization. She reflected upon this factor in relation to a specific case that she dealt with years before. The case concerned two girls who were born and raised in the UK, from Muslim background, and who joined ISIS and fled to Syria. These two female adolescents were groomed online, searching for the (mistaken) love of jihadi male fighters, whom they had believed that they were going to marry:

“… So, they had like a ‘poster-boy’: a good-looking blond, blue-eyes, Australian, jihadi… they [the groomers] were using these images as saying: “This is the one who you are going be meeting, you are not going to be meeting a Bengali or a Pakistani man. These are special men who come here, and we need to find wives for them”. (Female stakeholder, UK)

Community organizers and practitioners involved in prevention actions explained that the girls who had gone to Syria and joined ISIS were also girls who, in many cases, converted to Islam. This is not a new reality; Western media chains have reported hundreds of cases both in the US and Europe. In these cases, the component explained by Elizabeth can also be identified, that is, young females are groomed in their search for the ‘jihadi lover’. Thus, the pulling factor to join ISIS or other similar terrorist groups is not religion. Another factor explained by some community actors is that girls involved in organized crime networks and/or undergoing processes of violent radicalization also adopt violent behaviors and attitudes, following the path of their male mates. Empirical research in the field of gender studies and violence has already shown how the relationship between violence and attraction constitutes a strong risk factor for gender violence victimization, which underlies in many cases other situations of enduring violence (domestic, family, among teens, etc.) [28]. However, this specific aspect has barely been discussed at the light of specific literature on female violent radicalization [7, 53].

A social worker from Gangway [54], an organization who works on outreach to vulnerable young people and adults in public spaces (e.g., parks, streets) in Berlin, Germany, also recognized that those people who are violent are not the ones who are rejected, but on the contrary, those who have more success and who are even more respected:

“And society even rewards that (…) Of course, we see that if I punch you, I can see the success immediately and I’m even admired for it, or respected…” (Female stakeholder, Germany)

As the rule works in the opposite way than it should (violent subjects are many times recognized and rewarded instead of being rejected), transformative initiatives within the realm of prevention emerge from deglamorizing and contesting this lure of violence, specially within the educational context. As a latent element in all the practices identified, this lure has not always been approached or discussed explicitly by organized actors working on the ground.

St Giles Trust and Connect Futures implement the BRAVE project (building resistance against violence and extremism), delivered across the city of Birmingham in England and some selected boroughs in London. Within BRAVE, a set of workshops in schools and educational centers are being implemented, aimed at building resilience against violence and extremism by focusing on issues such as gang activity, online radicalization and exploitation. In the workshops, two aspects are discussed. First, the prevention of gang exploitation, specially by dispelling the positive attractive myths revolving around gangs and criminal culture, while also explaining the grooming techniques that are used to attract young people to criminal activity. Second, real cases studies and examples from far right and Islamic extremism are shared. This way, the spaces created by the BRAVE project allow engaging with youth about meaningful conversations surrounding the culture of gangs and violent subjects (e.g.: what it truly is, its consequences, what’s behind, etc.), thus opening up a vein to debunk existing myths around it.

Another approach focusing on breaking the link between violence and attractiveness and which offers transformative alternatives has been identified in those schools that are part of the international Network of Schools as Learning Communities, and which implement “successful educational actions” (SEAs) [55]. Schools organized as Learning Communities exist in Europe in countries such as Spain, Italy, Portugal, or Malta, and throughout Latin-America. They are organized on the basis of two key principles. First, the implementation of SEAs, universal and transferable actions based on scientific evidence which have shown to be effective in contributing to the educational success of all students, and to the improvement of school climate. And second, the recreation and implementation of these universal SEAs in each school, counting with the participation of the whole educational community. For our study, we interviewed the head teacher of a public school located on the outskirts of an industrial city near Barcelona, which is organized as a Learning Community and has, for years now, implemented the SEA defined as “dialogic model of prevention and conflict resolution”.

Placed in a neighborhood of which 90% of the inhabitants are of migrant and Muslim background, this school has been recognized several times by public and private institutions in Catalonia, as well as reported in the media, for both the outstanding educational results of its students (scoring results that stand above the average of those obtained in schools located in middle-high SES neighborhoods in the region), and the role that the school is playing in contributing to school climate and to preventing and reducing conflicts in a highly-diverse neighborhood. The dialogic model of prevention and conflict resolution has been highlighted by existing literature for playing a key role in this regard [56, 57]. As the head teacher interviewed explains, this consists in the involvement of the entire educational community members -from students, teachers, school staff, as well as parents and other family and community members, including representatives of both religious institutions with presence in the neighborhood, as well as of neighbors’ associations- in all the educational decisions taken in the school, as well as in the resolution of any conflict occurred in the school and related to the school context. This way, the school counts with a ‘coexistence agreement’, which represents those shared values and commitments between the school and all institutions of the neighborhood.

This dialogic structure of inter-relation between the school and the neighborhood and the coexistence agreement is well-mirrored within the own school. An outstanding norm in the school, and present across its different spaces is that of zero tolerance towards violence. To enforce this norm, students created the “Zero violence Brave club”, which acts as a shield of protection for victims in situations of bullying or aggression. Hence, when any situation of conflict or violence emerges within the school and among students, instead of hiding it or turning their back to it, students are committed to explaining it to the adults at the school, either to the teacher or to those adults who volunteer at the school. Accordingly, the idea being encouraged is that speaking up against violence and standing on the side of victims is what brave people do:

“We do talk a lot about the attitude of the violent person. We know that it does not pass from one kid from another… But that it is built on complicities. When this occurs, kids know that they are second-order harassers. We report this way, we call things by their name. If someone is an aggressor, he or she is an aggressor…. We also know who is the brave one. We try to find consensus, for instance, in the teachers’ meetings.” (Female stakeholder, Spain)

As argued by her, if the principle of zero tolerance towards violence is key in the construction of a violence-free context within school, the way teachers react in situations of conflict and aggression between students is very relevant. In this sense, the language they use needs to go beyond that of “harming others is not correct”, to make explicit that “harming others is not correct, and cowards do it”. As violence is often portrayed with glamour and linked to attractiveness, she explains that adults need to do the opposite, and shift such attractiveness towards the moments when children do good, making this explicit with the type of interactions and language used by them:

We have learned to use the word “you are a rock star”, “you are brave”, the little ones love to be the brave ones… but the ones who are 12 years old, adolescents… they want to be ‘the rock stars’” (…) (Female stakeholder, Spain)

A central aspect has been treated in this subsection, that of the glamorization of violence and how it permeates youth interactions due to the coercive dominant discourse which shapes the socialization of many of them. Contesting youth violent radicalization requires acknowledging its existence, and directly engaging this dimension at the time of working with youth, from the early ages within formal school contexts, to those other manifold informal spaces in which prevention is being carried out.

### Community voices regarding the unintended consequences of some institutional prevention policies

Narratives and testimonies from the stakeholders and end-users interviewed revealed at least three aspects which, directly related to already implemented institutional policies, are eroding and in some instances counteracting the positive impact of prevention practices implemented on the ground by organized community actors. These aspects are the unintended effects of police interventions, second order harassment, and the stigmatization of the Muslim community. These aspects directly inform the exclusionary dimension of our communicative analysis, as they suppose a barrier for the building of allies on the ground, contribute to the spread of narratives that link violent radicalization to specific minority groups, suppose the undermining of material conditions of targeted groups, among others unintended consequences.

Evidence from the fieldwork echoed what existing research already showed regarding the negative impact of prevention and counter-terrorism policies that include police interventions with measures such as the stop and search methods. Stakeholders from the UK, Germany and the Netherlands explained that most of the times stop and search methods are implemented to identify potential terrorists in diverse settings, but that these mechanisms end up put in practice in neighborhoods predominantly inhabited by racial and ethnic minority groups. In this regard, some stakeholders listed the fact that young adults pertaining to the Muslim community are more likely to be stopped and searched by the police than the average population, an experience that at least two female end-users of Muslim background interviewed identified as familiar:

“Yes a feeling of criminalization exists… Especially among young people… They are constantly stopped without being given any particular explanation” (Male end-user, Spain)

“For example a friend of mine was at the airport, because she wanted to travel somewhere, I don´t remember where she was going. So she was asked to step aside and take off her veil, to see whether she was hiding something there. I mean, then you would also have to ask women who do not wear a veil to step aside and have their bellies searched or something. I think this is a pity, there is no equal treatment.” (Male end-user, Germany).

Similar examples of this perceived criminalization towards the Muslim community were provided by participants (stakeholders, and end-users) of a focus group held in the UK. Participants mentioned the effects that the implementation of the PREVENT strategy (part of the UK counter-terrorism strategy) [58] have had among the Muslim community. According to a female participant of Muslim background, the PREVENT strategy has generated fear and distrust among Muslims in the UK, as instead of being a measure of safeguarding, has spread a discourse which associations Islam and Muslims to terrorism, thus criminalizing not only to adults Muslims but also to children. Participants also emphasized that if this Strategy had been designed and truly implemented counting with the community, such environment would not have been created.

Another example of a negative impact related to stop and search measures when implemented targeting entire social groups are instances of indiscriminate police intervention in spaces of worship such as Mosques. This was mentioned by stakeholders linked to faith communities from Germany and Spain who explained that in some cases there has been forceful intrusion into Mosques:

“Measures are necessary for the protection of society in general. Protocols work well on paper, but they do not work well when applied to the whole population. It can be understood differently depending on how they are implemented. In the police bodies, laws are applied negatively. For instance, in 2009 there was an Imam who had no regular documents. He was arrested on a casual Friday when the Mosque was full (600–700 people), with weapons, with shoes, etc. This is an erroneous application of the protocols, without taking into account sensitive issues; so protocols can be applied in different ways. Islam is associated with terrorism, in the media, and this fact causes huge damage (…)” (Male stakeholder & end-user, Spain).

Existing literature has already underscored numerous facets of harassment directed at suspects’ relatives, including isolation, police brutality, undignified treatment and further consequences such as on the family economy, emotional and psychological wellbeing [59]. This sort of harassment against family members of potential suspects and children is what has been defined as *second order harassment*, that is, punishing not only the suspect or convict, but the people around the suspect or convict regardless of their involvement in the radicalization or not. Data gathered evidence that most of the previous negative aspects of police intervention are not only applicable to the suspect or terrorist him or herself but also to their families. Second order harassment against families is manifested in actions ranging from the disclosure of personal data of suspects thus leading to the public identification of family and relatives, lack of social services and support systems leading for instance to younger siblings follow their older siblings path, harassment or termination of their contracts in their workplaces, to severe implications on health. The narrative of the stakeholder from Mothers for Life (already introduced in previous subsections) gives a sense of how harassment impacts on families:

“Often families are targeted and treated as criminals as well. Because it is a very delicate situation, they are scrutinized by the authorities, the laws are still not consistent all the way through, policies are vague, there is nothing indicating how anybody is getting any support or assistance. It’s more about a top level, about jail sentences, and even then it is unclear how the trial is to proceed” (Female stakeholder, international organization)

A stakeholder from Germany explained how second order harassment in this case perpetrated by the police impacted on both the lives of suspects’ family members, and the work that community organizations such as the one that he represents do on the ground with victims -based on building trust day after day:

“It becomes problematic, we now have a case of a girl whose brother has gone to AL Qaeda in Syria, and she receives counselling from us, we guide her to keep the contact with her brother to influence him and to find out what is going on. She participates in our Parents group. And suddenly the Criminal Police came and confiscated her mobile phone, with all kind of data on it, and the police officer requested all the laptops arguing that she was still in contact with her brother, and she told him about the counselling, and he just said they don’t care about counselling. We try to get to their superiors now. These were the comments of a small police officer, and this is not how it works! (…)” (Male stakeholder, Germany).

Evidence collected in the fieldwork suggests that second order harassment also affects those people working with suspects in prevention and/or deradicalization. Especially deradicalization implies working with people who are or have been radicalized and have been at least close to terrorist networks. Thus, proximity of the workers to terrorist networks is close, due to the crucial work they are doing to help those who want to get out of these networks and those who are at risk of becoming further involved in these networks to actually achieve their goal of pursuing a different path. It should be noted here that this type of second order harassment as far as we have observed in our work only affects members of the Muslim community, who for instance see themselves much more affected by rough measures and public defamation than a non-Muslim. In the fieldwork conducted for this study no non-Muslim individual has suffered from second order of harassment due to their work in prevention or deradicalization and their potential ties to suspects or terrorists. This does not mean that it could not be the case, but it is at least a less present reality so far. An example provided by an expert interviewed for the case of Germany helps to better understand how this type of harassment of those working on the ground takes place:

“There was this case recently of Violence Prevention Network in Hessen and the two colleagues who suddenly were suspended from work, because a journalist and a self-identified Islamism expert in Hessen, in Frankfurt, said that these counsellors who were Muslim, were undercover islamists (…) but of course they were no islamists (…) They are doing work by going towards the people, projects with youth in prison and then they couldn´t go there anymore, and you can imagine, they are in conversations with someone who is considering leaving this, leaving this ideology and to integrate into society, but knowing that they will never be truly accepted. And then suddenly his counsellor is being suspended because he is being defamed as an islamist, that is a huge loss of confidence.(…) that affects especially Muslims more than anybody else, obvious.” (Male expert, Germany).

The stigmatisation of the Muslim community as a suspect community is not only result of some misleading forms of police intervention or negative law enforcement procedures. Evidence illustrates that it is also further enhanced from diverse social spheres, including science, media or education. Two stakeholders of Muslim background working for the Kompass project in Germany, and who are both working in prevention of radicalization and deradicalization of Muslim youth in youth retention centers, emphasize the need to eradicate anti-Muslim racism from science. They complain that a frequent phenomenon in academic circles when approaching issues related to Islam and Muslim communities is that of reaching conclusions that are not accurate, and which contribute to making some social groups even more vulnerable:

“We are both working on our dissertations and we see how things are communicated, how populist things are communicated, and when non-Muslims, self-identified experts in Islam elaborate theories on Muslims and about the religion Islam. Central terms of our faith such as Islamism, Salafism and Jihadism are used in science in a very diffused manner, and have become catchwords for international terrorism. We have to provide our own and corrective theories, and draw on our tradition to clarify this before the youth (…)” (Male and female end-users, Germany)

Another field in which such stigmatization is observed is in media, which in many instances is still feeding Islamophobic discourse and actions instilling a greater sense of fear, vulnerability, insecurity, and helplessness of the Muslim community. Anti-Muslim discourse in the media is no news, and examples of this can be observed across countries. An example of this was provided by another stakeholder in Germany regarding a case of an Imam working in deradicalization accused for connections to terrorist networks. The yellow press would call him ‘Prügelimam’ [Beating Imam], even after the case was filed. The stakeholder interviewed explained:

“Until today [7years later] when he was now arrested, the Bildzeitung and the Bayrischer Rundfunk publishes headlines like: the Beating Imam is a terrorist now. The headlines were outrageous.” (Male stakeholder, Germany).

A young Muslim man explains that he has felt victimized by the media and even feels blame for terrorist attacks although he has nothing to do with it.

“Dignity… I think they stigmatize us, they put us in the In the eye of the storm, I have often felt victimized (…) and even I blamed myself to a certain point (…)”. (Male end-user, Spain)

Despite the progress made by European and international institutions such as the Fundamental Rights Agency (FRA) or the Association of European Journalists about the monitoring of media compliance with ethical regulations, and how terrorism is reported, or even the existence of editorial guidelines provided by recognized broadcast institutions such as the BBC about how to not perpetuate stigmatization of specific ethnic minorities in the media, in general, there is still a spread of negative discourse that can affect the integrity of the person, as well as foster the feelings of exclusion, discrimination and criminalization.

Finally, stigmatization of the Muslim community also takes place in education. Some of the stakeholders and end-users interviewed mentioned the PREVENT Strategy in UK or the existing PODERAI protocol in Catalonia [60], Spain, as programs that have worsened existing stigmatization, thus underlining the impact of counter-terrorist policies on different social spheres. These type of policies foresee that teachers should be sensitized to the problem of radicalization in order to report potential signs of radicalization among their students, although scientific literature has showed that in most instances thorough training is not provided to the teachers [61]. Stakeholders interviewed in Catalonia and in the UK complain that the identification of early signs of radicalization end up most of the times relying on the sole criteria of every single teacher. For instance, a high-school teacher in the United Kingdom, explained that with the PREVENT strategy, students who used to talk about their concerns stopped doing it because they did not feel with the trust of not being reported. More evidence of this impact is given by stakeholders interviewed in Spain, in this case the principal of the primary school in Spain (introduced in the previous section), committed with an educational project that promotes inclusion rather than segregation:

“(…) From the department of education and the Mossos [local police in Catalonia] teachers were called to explain the PRODERAI CE protocol, and also to explain the indicators that could be considered as risk of radicalization. At the end of the course, all the schools’ principals were called for a meeting, but just those from the school with higher diversity (…) This is not prudent, because principals do not know the teachers’ sensitivity once receiving such information (…) It also seems that the Education Department only applies this protocol to those centres with greater multiculturalism, and that is not the focus of the problem, but rather it is a "problem" / situation that affects everyone, not only those schools with more cultural diversity.” (Female stakeholder, Spain)

In all, the elements discussed in this subsection illustrate some of the side-effects that counter-terrorist policies are having on targeted communities. They are of relevance and need to be take into account for supposing a burden to both the fundamental rights of some individuals, as well as for negatively impacting on the prevention work that organized actors on the ground are developing.

## Discussion and conclusions

The threat posed by violent extremism and terrorist groups operating on a global scale is currently one of the topics of most concern in Western societies, and it is in the spotlight on the political agendas of governments and public institutions. The potential reach of these groups has become transnational, thus making it possible to attract and capture individuals located in different national contexts who are experiencing similar troubled situations at both the individual and community level, e.g., from the loss of meaning to community disconnection. Embedded in complex intercultural and unequal societies [62], youth growing up in the 21^st^ century navigate in an interconnected world, facing risks that half a century ago used to be offline but are now also perpetrated online, e.g., from cyberbullying to grooming [63, 64]. Drawing on all this, there is an urgent need not only to better understand the factors pushing many youth towards violent radicalization but also to develop a more consistent body of research to guide how to implement prevention policies and practices on the ground.

In line with the abovementioned research, although there is agreement that there is a need to complement the policies focused on the use of force and active security by developing bottom-up policies and actions oriented towards prevention on the ground, clear evidence-based insights on how to do this are still sometimes vague and scarce. Aimed at filling this gap, in this article, we have explained the practices that are being implemented on the ground by organized actors that are contributing to preventing youth undergoing vulnerable situations from falling into processes of violent radicalization. Drawing on the communicative analysis of qualitative data collected, we have discussed the shared underlying features identified among practices implemented across countries that are contributing to prevention (transformative dimension), as well as those aspects that according to actors themselves, are either eroding or even counteracting the positive effects of such practices (exclusionary dimension). All this has been done in an attempt to shed light on the discussion about how to make communities more resilient to violent extremism and terrorism, as well as to protect youth from falling victims of violence [39–41, 65].

The first feature discussed is that all prevention practices identified are being implemented with a bottom-up approach, making allies with different community actors to reach and work with youth at risk and sharing a solid rejection of any type of violence. Those doing prevention on the field set networks of allies and operate through these when deploying their programs, emphasizing the need to involve all relevant stakeholders in this prevention endeavor, such as family relatives, educational agents, police agents, among others. Most of the times these connections take the approach of first identifying and later working with youth at risk of falling into violent networks. Our work performed within the framework of the PROTON project showed that counterterrorist policies solely oriented towards disrupting networks, although perceived as required, are not enough to identify and challenge the risks that extremist ideologies pose. In this regard, evidence collected shows that specific types of police interventions such as those which include stop and search methods, have the potential to be indiscriminately applied towards specific social groups, such as racial and ethnic minorities, or migrants. This type of practices when applied in biased ways can nurture and increase criminalization towards such groups, and also have a backlash effect on the prevention work that those operating on the ground develop, mainly based on building bonds of trust in the relationship with those at risk.

In this line, evidence gathered reveals that community agents, peers and family relatives are indeed needed for both identifying those sites where youth might become vulnerable to violent radicalization, as well as for reporting potential cases of radicalization and preventing others to fall victims. Being these findings in line with existing literature about the role played by family and close friends [33], evidence underscores the urgency of acknowledging how each of them can be a key ally when working on the ground and articulating a shared narrative of violence rejection, consistent enough to contest the existing glamorization of violence in its many manifestations. Hence, although the existence of protocols at national levels, and well-defined EU legal schemes foresee ways of proceeding to *prevent*, *protect*, *pursue and respond* to violent radicalization and terrorism [66], when such measures are deployed on the ground, more intra and inter-level coordination with the manifold actors is still required for their proper enforcement.

The second feature discussed was that practices that have a positive impact underscore the importance of building ties of trust with those at risk to then be able to openly discuss what healthy friendship relationships consist in. As the existing literature has stressed, the interactions that youth have can either prevent them from falling into networks of violence or lead them towards violence [25]. Sagerman [29] theorized the idea of the ‘bunch of guys’, explaining that networks of peers are key in explicating why youth join terrorist networks. Similarly, Jasko, La Free and Kruglanski [35] explored the psychological state that can lead to a process of radicalization, arguing for three factors that together can trigger radicalization: a state of personal insignificance, a violence-justifying ideological narrative, and a network of people who subscribe to that narrative. Accordingly, an element that makes the practices discussed to be impactful is that they encourage youth to think critically about what it means to have a friend, while working on creating alternative contexts that are meaningful and to which the youth can belong articulating their multiple identities: the youth can express doubts, share concerns, and show their fears.

Stakeholders interviewed explained that many times the youth who are *flirting with violence* have an impaired ability to identify who is a real friend, especially in those situations when they have fallen victim to exploitation. Organized actors who work on the ground and understand the daily reality of many of these youth and the context in which they operate (e.g.: from resource limitations, to daily experiences of racism or islamophobia, or even contexts of violence), create interventions aimed at challenging the assumptions that the youth have about those whom they consider to be their friends but whom many times pose them, and even their families at risk. This is done at the same time as the building of relations that are freely chosen. Evidence from the different practices analyzed indicates that the educational and other public spaces in which the youth participate are crucial not only for promoting their emotional health at the individual level [67] but also for being sources of collective meaning creation that moves them away from violence. In line with this, when these spaces are ruled according to explicit norms that incorporate shared values such as the defense of cultural diversity, equality and democratic freedom, and once more, clear rejection of any type of violence, both prevention of violent radicalization, as well as the promotion of alternative models of interaction are more likely to be effective.

Therefore, if effective actions to fight youth radicalization and extremism are to be designed, meaning creation standing on these shared values and a clear position of violence rejection and condemnation cannot be overlooked. The on-going glamorization of violence emergent from different offline and online spaces in which youth are engaged already underlies and accompanies the whole process of their socialization of many youth. Fighting it requires that those working at different levels are able to understand how this shape -and undermine- youth identities, preferences and actions.

Finally, the third feature identified is the lure of violence and violent subjects and the role that the lure plays in processes of violent radicalization. It is also identified how actors working at the grassroots level first recognize it and then create transformative alternatives to challenge this lure. Stakeholders interviewed identified that those youth with whom they work are often motivated by the excitement generated by violence. Violence is thus glamourized while being embedded in those sites of socialization of many adolescents and young adults, so elements to both identify the glamorization and contest it are needed. Evidence collected has shown that on many occasions, terrorist acts have been portrayed by the mass media with overtones of glamor and excitement, which instead of condemning violence, shows it as fashionable or even fancy. As explained by stakeholders, these type of narratives and discourses are likely the norm rather than the exception among youth involved in gang related issues and prone to violent radicalization.

Consequently, our findings indicate that contesting such glamorization of violence requires first, to recognize how this coercive dominant discourse operates and is manifested in the different realms of social life (e.g.: mainstream media, sites of socialization including the private sphere and public spaces, etc.), and second, to design and put in place actions oriented to tackle it, such as the creation of spaces that allow debating about what radicalization, gang groups, or extremist ideas are about, their implications and consequences, among others.

Some of the practices identified and reviewed in this work which addressed the lure of violence (described in subsection 3) are outstanding as they acknowledge the relevance of treating this very aspect in a direct way, and not as a side-issue -collateral for instance to specific material conditions. An example of this is the *dialogic model of prevention and resolution of conflicts* that is being used in some schools organized as Learning Communities, involving families and community members in the construction of contexts free of violence in which solidarity prevails [68]. The relevant contribution of this model is that through it, children both reject violence and build a shield in front of the perpetrators of violence, thus showing everyone that those who are violent are not the ‘machos’ but the ‘cowards’ [56].

One of the aspects identified as having a negative impact on the positive advancement of prevention practices—unintended effects of police interventions was mentioned above. Furthermore, both second order harassment and stigmatization of specific ethnic and racial groups derived from the implementation of prevention and counter-terrorist institutional policies are also aspects that need to be borne in mind. Narratives and testimonies from both stakeholders and end-users suggest that these aspects derived from the mis-implementation of policies undermine the work of grassroots actors in the field of prevention. Furthermore, these aspects also feed narratives and a social imaginary of certain social groups (in the current context of Islamophobia, the Muslim community has been victim of such stigmatization) as the ones more prone to violence and violent radicalization. Analyses conducted for the broad CREA-UB study in PROTON, -including narratives of stakeholders and end-users specifically revised for this work- support that counter-terrorist and violent radicalization prevention strategies are needed, as also the work that police and other institutions are doing. However, when it comes to considering how these policies are designed and deployed on the ground, stakeholders operating at the grassroots level which count with their networks of organizational infrastructure need to be taken on board. The case of the UK with its Prevent strategy, or the existing PRODERAI protocol in Catalonia are significant examples in this regard. Although in a different scale, both cases reveal not a negation from the side of stakeholders of the issues of violent radicalization and the problem and threat that it supposes, but a rejection of how the problem has been addressed from the side of institutional authorities and experts. Although scientific literature has emerged in the last years discussing and assessing the impact of these cases (specially the British one) much more can be learnt of these experiences, specially looking at how implementation processes need to be done, even more when the issues at hand are topics as sensitive as violent radicalization and extremism, and the ethical concerns raised.

At this point, at least three limitations of this study and potential areas of future scientific inquiry can be pointed out and explained. First, we have identified three shared common features among practices developed by organized actors across Europe, which are contributing to preventing youth violence radicalization. However, other underlying features of these practices not addressed in this study can be further explored, not solely looking at how they operate in dialogue with the ones already identified, but also for their prominence in making a practice more effective in its work towards prevention. For instance, the way in which organizations deal with crime perpetrators on the ground when developing their work is an additional aspect that requires further analysis. Such analysis can inquiry on the (dis)similarities in the positions taken by organizations themselves vis-à-vis crime perpetrators (dialogue/non-dialogue), or even if there are instances (and if so, when) in which organizations act as mediators between institutional bodies such as the police, and extremist groups, for instance, in negotiating end of violence or access to information.

Second, it was not the main focus of this study to deepen into the exclusionary aspects of the practices, but to focus on those ones that are contributing to their effectiveness. Nonetheless, exclusionary aspects observed were derived from the implementation of institutional policies. Taking this into account, more research needs to be done specifically looking at how the effects of institutional policies at the grassroots level influence and erodes the work of stakeholders doing prevention as well as its impact on end-users.

Finally, the relevance of the coercive dominant discourse and the role it plays in pushing the link between attractiveness and violence has been discussed in this study, as well as how it is also shaping existing violent radicalization processes. However, not being this the main area of concern of this study, this aspect has not been approached in depth. Existing literature has emphasized the role played by friends and peer interactions in processes of radicalization, such as for instance, the impact of unhealthy or toxic relationships. Further consideration needs to be given on looking at this from the perspective of how sexual-affective relationships in which there is violence can become a catalyst of youth victimization processes in the context of violent radicalization.

In summary, the evidence presented in this article aimed to shed new light on how effective prevention of youth violent radicalization can be conducted. Examples of real cases have been analyzed, which have shown that the underlying common features of effective practices are based on a clear rejection to violence. While doing so, the voice has been given to community agents -stakeholders, and end-users- operating on the ground who devote their hands and hearts to preventing the youth from falling into violence while looking for a way out of it.

## Supporting information

S1 File (PDF)

S2 File (PDF)
