# Supplementary material for: Grassroots community actors leading the way in the prevention of youth violent radicalization
Source: PLoS One. 2020 Oct 12;15(10):e0239897. doi: 10.1371/journal.pone.0239897 (PMC7549796; doi:10.1371/journal.pone.0239897)
Supplement: S1 File — (PDF) [file pone.0239897.s001.pdf]

PROTON

Modelling the processes leading
to organised crime and terrorist networks

Co-funded by the
European Union

D1.1 Report on factors relating to OC

December 2017 (M15)

WP1 - OC Networks: Social, Psychological & Economic Factors

Tasks T1.1, T1.2, T1.3, T1.4, T1.5, T1.6, T1.7

Authors: UCSC-Transcrime, UB – CREA, VU/VUmc and WODC, UNIPV, UNIPA

Modelling the PRocesses leading to Organised crime and TerrOrist Networks
FCT-16-2015

This project has received funding from the European Union's Horizon 2020
research and innovation programme under grant agreement N° 699824.

Technical References

Project Acronym	PROTON
Project Title	Modelling the PRocesses leading to Organised crime and TerrOrist Networks
Project Coordinator	Ernesto Savona<br>Università Cattolica del Sacro Cuore<br>ernesto.savona@unicatt.it
Project Duration	October 2016 – September 2019 (36 months)

Deliverable No.	D1.1
Dissemination level [1]	PU
Work Package	WP1 - OC Networks: Social, Psychological & Economic Factors
Task	T1.1, T1.2, T1.3, T1.4, T1.5, T1.6, T1.7
Lead beneficiary	1 (UCSC-Transcrime)
Contributing beneficiary(ies)	7 (VU/VUmc), 8 (UB – CREA), 13 (UNIPA), 14 (UNIPV), 21 (WODC)
Due date of deliverable	31 December 2017
Actual submission date	22 December 2017

[1] PU = Public
PP = Restricted to other programme participants (including the Commission Services)
RE = Restricted to a group specified by the consortium (including the Commission Services)
CO = Confidential, only for members of the consortium (including the Commission Services)

Security Advisory Board Review	
Comments	No security sensitivity issues
Recommended Distribution	It is therefore suitable for public dissemination as required by the DoA
Date	21 December 2017

Document history			
V	Date	Beneficiary	Author
V1	8/12/2017	UCSC	UCSC, VU/VUmc and WODC, UNIPA, UB-CREA
REV	22/12/2017	SAB	SAB members
V2	24/03/2018	UCSC	UNIPV

Summary

This document constitutes the deliverable D1.1 *Report on factors relating to OC* and collects the outputs of Work Package 1, *OC Networks: Social, Psychological & Economic Factors,* coordinated by Università Cattolica del Sacro Cuore–Transcrime (UCSC–Transcrime).

The deliverable includes the following sections:

Chapter 1: Report on the systematic review (task T1.1, led by UCSC–Transcrime);

Chapter 2: Report on the OC policy analysis (task T1.2, led by UB-CREA);

Chapter 3: Report on criminal careers of OC offenders in context (Task T1.3, led by VU/VUmc and WODC);

Chapter 4: Report on recruitment into mafias (task T1.4, led by UCSC–Transcrime);

Chapter 5: Report on emotional and cognitive determinants of OC involvement (task T1.5, led by UNIPV);

Chapter 6: Report on socio-economic inequalities and OC development (task T1.6, led by UNIPA);

Chapter 7: Report on the policymakers' contribution (task T1.7, led by UCSC-Transcrime, in cooperation with Brå, DPPS, EUCPN, EUROPOL, UNODC and WODC).

Table of contents

CHAPTER 1: SYSTEMATIC REVIEW OF THE SOCIAL, PSYCHOLOGICAL AND ECONOMIC FACTORS RELATING TO CRIMINALISATION AND RECRUITMENT TO OC....... 5

CHAPTER 2: ETHICAL AND SOCIETAL IMPACTS OF ORGANISED CRIME POLICIES 72

CHAPTER 3: REPORT ON CRIMINAL CAREERS OF OC OFFENDERS IN CONTEXT 145

CHAPTER 4: RECRUITMENT INTO MAFIAS: CRIMINAL CAREERS OF MAFIA MEMBERS AND MAFIA BOSSES....... 192

CHAPTER 5: REPORT ON EMOTIONAL AND COGNITIVE DETERMINANTS OF OC INVOLVEMENT 313

CHAPTER 6: REPORT ON SOCIO-ECONOMIC INEQUALITIES AND OC DEVELOPMENT 332

CHAPTER 7: POLICY MAKERS' CONTRIBUTION 358

CHAPTER 1: Systematic review of the social, psychological and economic factors relating to criminalisation and recruitment to OC (UCSC-Transcrime)

Authors: Ernesto Savona, Francesco Calderoni, Elisa Superchi, Tommaso Comunale, Gian Maria Campedelli, Martina Marchesi, Alexander Kamprad

Table of contents

SUMMARY 6

1. ORGANISED CRIME AND THE FACTORS RELATING TO RECRUITMENT TO ORGANISED CRIME NETWORKS 7

1.1. CONCEPT AND DEFINITION OF ORGANISED CRIME 7

1.2. THE FACTORS RELATING TO CRIMINALISATION AND RECRUITMENT TO OC NETWORKS 9

1.3. RESEARCH QUESTIONS 11

2. DATA AND METHODS 13

2.1. TYPES OF ORGANISED CRIME GROUPS 13

2.2. IDENTIFICATION OF THE SOURCES 15

2.3. SELECTION OF RELEVANT STUDIES 18

2.4. CORROBORATION OF FINDINGS 19

3. FINDINGS 20

3.1. DESCRIPTION OF THE INCLUDED STUDIES 20

3.2. SOCIAL, PSYCHOLOGICAL, AND ECONOMIC FACTORS RELATING TO RECRUITMENT TO ORGANISED CRIME GROUPS 26

3.3. RECRUITMENT ACROSS TYPES OF ORGANISED CRIME GROUPS 42

4. DISCUSSION AND CONCLUSION 46

5. REFERENCES 51

METHODOLOGICAL ANNEX 62

CRITERIA FOR INCLUDING STUDIES IN THE REVIEW 62

SEARCH METHODS **64**

SELECTION OF STUDIES **68**

CORROBORATION OF FINDINGS **71**

Summary

This report presents a systematic review of the social, psychological, and economic factors relating to criminalisation and recruitment to organised crime groups (OCGs). It encompasses different types of OCGs, namely mafias, drug trafficking organisations (DTOs), gangs, and a residual category including other criminal organisations.

This review has three objectives: (i.) identifying the most commonly reported factors leading to recruitment into OCGs, (ii.) highlighting how they vary across OCGs, and (iii.) assessing the validity and generalisability of research findings. The review searched all possible relevant studies indexed in selected databases and published in five languages (i.e. English, French, German, Italian, and Spanish), without limitations as to their year of publication or geographic origin. Starting from 48,731 potentially eligible records, and after experts' suggestions, the systematic review includes 47 empirical studies employing quantitative, qualitative, or mixed-methods approaches.

The findings show that social and economic factors are the most commonly reported factors relating to recruitment into OCGs, while psychological factors are marginal. Individuals with violent attitudes and behaviour, low socio-economic status, and kinship and blood ties with OC offenders are more likely to join OCGs. Although factors are highly interrelated and shared across OCGs, their predominance varies across types of criminal organisations. For instance, individuals join mafias and gangs attracted by strong group identity, whereas individuals enter DTOs mainly for financial gain. Despite internal consistency, findings can hardly be generalised beyond the OCG and the geographic area under analysis. This is due to the nature of the data and research designs adopted by the reviewed literature. Recommendations for future research are discussed.

1. Organised crime and the factors relating to recruitment to Organised Crime networks

1.1. Concept and definition of Organised Crime

Organised crime (henceforth OC) has significant impacts on a wide range of countries all over the world. Globalisation has facilitated the flow of people and goods, and this also applies to criminal organisations (Adamoli, Di Nicola, Savona, & Zoffi, 1998; Morselli, Turcotte, & Tenti, 2011; Passas, 1999; Varese, 2011a). Research on OC originated in the United States during the twentieth century (Woodiwiss, 2003). American scholars have in-depth analysed the Italian-American mafias (Abadinsky, 1981; Albini, 1971; Block & Scarpitti, 1985; Cressey, 1969), drug trafficking organisations (henceforth DTOs) and gangs (Adler, 1993; Albanese, 1985; Kelly, 1997; Matza & Sykes, 1961; Reuter & Haaga, 1989; Thrasher, 1927). In Europe, studies focused on the Italian mafias (Gambetta, 1996; Paoli, 2003), but also on organised crime groups (henceforth OCGs) from different ethnicities and countries (Fijnaut & Paoli, 2004b; Varese, 2005). In Asia, works have particularly examined the Chinese Triads and the Japanese Yakuza (Chu, 2000; Hill, 2003; Kaplan & Dubro, 2003). More recently, researchers analysed OCGs in Latin America, with a particular attention to the development of DTOs and criminal gangs (Bagley, 2004; Bagley & Rosen, 2015; Beittel, 2012; Bunker, 2015; de la Miyar, 2016; Vásquez, 2015).

The variety of studies across countries and the heterogeneity of criminal organisations they encompass reveal the complexity of the concept of OC. The concept and definition of OC have long been debated by institutions and academia (Calderoni, 2012; Finckenauer, 2005; Hagan, 1983, 2006; Symeonidou-Kastanidou, 2007; Von Lampe, 2008, 2015). The term 'organised crime' first emerged in late-nineteenth century in the U.S. and its conceptualisation varied over the past century (Fijnaut & Paoli, 2004a; Kenney & Finckenauer, 1995). OC was first associated with activities protected by public officials (e.g. prostitution and racketeering), then with criminal frauds, extortion and racketeering (Woodiwiss, 2003). Subsequently, in the 1950s, the organisation-based approach named 'alien conspiracy' became predominant. According to this theory, OC had to be intended as criminal organisations composed by foreign criminals, namely Italian immigrants, dominating

profitable US illegal markets (Cressey, 1969). The concept of OC began to be mafia-centred, although already in the 1960s the majority of American scholars started to reject this paradigm (Albini, 1971; Smith, 1975). In the 1970s, the criticism of the 'alien conspiracy' theory lead scholars to develop the 'illegal enterprise' paradigm posing the attention on the role criminal organisations in supplying illegal products and services (Arlacchi, 1983). The relevance of the economic dimension of OC also acquired a dominant position Europe, which remained out of the debate until the mid-1970s (Fijnaut & Paoli, 2004a). Ever since, the label OC has increasingly become more popular, in the U.S. as well as in Europe, with authors proposing various definitions.[1]

As with the conceptualisation of OC, over the last century the literature has debated on OC defining features. More recent contributions show the different aspects highlighted by scholars who conducted theoretical reviews seeking for a comprehensive OC definition.

Hagan reported that OC could be defined as "continuing organized groups that monopolize illegal enterprises through violence and threats and enjoy immunity of operation through corruption" (2006, p. 133). Von Lampe and colleagues (2006) conducted a meta-theoretical classification of the literature identifying four basic dimensions of OC: (i.) individuals, (ii.) structures, (iii.) activities, (iv.) and systemic conditions. Finckenauer (2007) embraced a different approach, focusing on OC distinguishing features. These include the role of ethnicity and kinship in enforcing individuals' mutual trust, profit-oriented activities, and the presence of a hierarchical organisational structure allowing for the provision of illegal services and the penetration of legitimate businesses. This latter aspect is what Varese (2010a) identified as the primary OC feature, seeking a definition not overlapping with other constructs (e.g. 'criminal network'). The author defined OC "as an organized crime group [that] attempts to regulate and control the production and distribution of a given commodity or service unlawfully" (2010a, p. 14). Definitions of OC are consistent in pointing at the embeddedness of OC into society as key to the interpretation of their criminal activities

Though some OC features are recurrent among the above contributions, they show the heterogeneity of the concept and definition of OC. Furthermore, from a legal and political point of view the adoption of a unique definition is problematic (Paoli, 2014a). This is also due to the fact that OCGs operate across countries and adapt to different contextual factors. Nevertheless, lately

---

[1] For a comprehensive list of definitions of OC see Klaus von Lampe's website on the Definitions of Organized Crime (www.organized-crime.de/organizedcrimedefinitions.htm).

governments and international organisations dealing with OC have often adopted the general definition provided by the United Nations Convention against Transnational Organized Crime (United Nations, 2000, p. 5):

> *'Organized criminal' group shall mean a structured group of three or more persons, existing for a period of time and acting in concert with the aim of committing one or more serious crimes or offences established in accordance with this Convention, in order to obtain, directly or indirectly, a financial or other material benefit.*

Though this type of definitions have been criticised by scholars for being too vague and inefficient (see Calderoni, 2012; Paoli, 2014a), the present study adopts the United Nations' definition. For the purpose of this systematic review, in fact, it is desirable to consider a broad definition, as it allows for more flexibility when searching for potentially relevant studies. Conversely, more precise or narrower definitions could result in excessively specific search criteria, leading to a limited number of included studies. To overcome possible limitations associated with the heterogeneity of OC and the adoption of such definition, this study identifies four categories grouping similar types of OCGs: mafias, DTOs, gangs, and other criminal organisations (see section 2.1).

1.2. The factors relating to criminalisation and recruitment to OC networks

Criminological studies have long focused on differences in offending patterns between individuals rather than on risk factors or changes in offending patterns within individuals over time (Farrington, 2003). Nonetheless, scholars have recently turned to a risk-based approach to identify the factors that lead individuals to join OCGs within the society they belong to. This process has been mainly driven by the expansion of developmental and life-course criminology[2] during the 1990s (Farrington, 2003, p. 222; Kleemans & De Poot, 2008). Several researchers have addressed within-individual changes in OC (Kleemans & De Poot, 2008; Morselli, 2003; Morselli & Tremblay, 2004; Van Koppen, de Poot, & Blokland, 2010; Van Koppen, Poot, Kleemans, & Nieuwbeerta, 2010), while others have taken a closer look at risk factors for

---

[2] Developmental and life-course criminology (DLC), term coined by Farrington (2003), is concerned with key factors for offending, effects of life events and life transitions on offending and development of offending.

joining OCGs (Kleemans & De Poot, 2008; Kleemans & Van de Bunt, 1999; Kleemans & Van Koppen, 2014; Klein & Maxson, 2006; Lyman & Potter, 2006).

Criminals do not operate within a social vacuum, but they are embedded in the social environment. The idea that studying criminal groups with a focus on social relations could help in understanding criminal organisations developed at the beginning of the 1970s (Albini, 1971; Cressey, 1969; Ianni, 1974). In OCGs more than other criminal groups, the social factors play a major role in the study of the processes of recruitment of new members. OCGs differ from high-volume criminal groups under several aspects: (i.) transnational nature, (ii.) importance given to social relations, and (iii.) need for several co-offenders and specific expertise for the complexity of the activities conducted (Cornish & Clarke, 2002; Kleemans & De Poot, 2008). For these reasons, relation with co-offenders and contacts with the legal world have a crucial role for the success of OC-related activities.

Kleemans and Van Koppen (2014) identified the aspects leading to recruitment and criminalisation into OCGs. Firstly, they pointed out that unlawful behaviour and active roles in OCGs comprise adults with no previous criminal involvement, as “late starters are not exceptional” (2014, p. 290). Thus, a career in OC does not necessarily start in early adolescence, with individual characteristics and long-term risk factors as an explanation for a lifetime involvement in crime. Conversely, social ties create a “social snowball effect”, intended as the process by which people get involved in OC through people close to them or acquaintances and the less they continue generating new criminal collaborations. Leisure activity as well as work relations can create the opportunity to enter OCGs. For instance, occupations involving mobility, transport, and logistics may create fertile soil for cross-border OC activities. These findings highlight how an in depth-analysis of social embeddedness could shed light on criminal recruitment, and particularly on the aspect of individuals’ late criminal involvement.

Psychological factors also influence individuals’ recruitment into OCGs. Several studies mainly relied on the Psychopathy Check List-Revised (Ostrosky, Borja, Rebollar, & Díaz, 2012), a controversial instrument with a number of limitations, the most important being the diagnosis of psychopathy itself (Jones, Wagner, Faigman, & Raichle, 2013). Other neuroscientific tools such as neuropsychological and genetic tests and even neuroimaging are widely used in courts to explain, and sometimes justify, criminal behaviours (Walsh & Beaver, 2009). To advance understanding of the psychological determinants of criminal behaviours in an innovative manner, it would be necessary to adopt these new tools as well, and thus move beyond the tools and models used by

classic criminology. Moreover, when investigating the psychological factors favouring recruitment into OCGs, researchers should also take into consideration other types of disorders, like substance use disorders, low-self-control and/or past disorders and negative history of development.

The literature has also analysed the economic-related risk factors for recruitment into OCGs. For instance, Lavezzi (2008, 2014) identified the factors and characteristics making Sicily's economy and its activities vulnerable to OCGs penetrations. Extortion and protection are common in Sicily where the economy is characterised by a large proportion of small firms, large sectors of traditional/low-tech economic activities, a large construction sector, and a large public sector (Lavezzi, 2008). Opportunities for OCGs are also created by the absence of Rule of Law alongside the presence of illegal and informal markets (Bandiera, 2003). For example, the imperfections in the credit markets can create opportunities for usury. Finally, inequality has been indicated as a possible facilitator in the emergence of OCGs: data from Italian regions shows a remarkable correlation between high income inequality and the diffusion of OC (Daniele, 2009). Condition of economic disadvantage can result in individuals' propensity to join OCGs (Carvalho & Soares, 2016).

1.3. Research questions

The literature has argued the importance of the social environment for individuals' involvement into OC (Albini, 1971; Kleemans & De Poot, 2008; Kleemans & Van de Bunt, 1999; Kleemans & Van Koppen, 2014; Morselli, 2009; Van Koppen, de Poot, & Blokland, 2010). Notwithstanding the growing interest on the social embeddedness of OC, less is known about the processes leading to the recruitment into OCGs. A systematic and scientific approach on empirically based findings will provide better understanding of OC, essential for effective, evidence-based intervention, and prevention policies.

As a first step of this Project, this systematic review aims at providing a comprehensive overview of the current knowledge on the social, psychological, and economic factors relating to criminalisation and recruitment to OCGs. The general aim can be subdivided into the following three main objectives:

- **Objective 1**: Identifying the most commonly reported social, psychological, and economic factors leading to criminalisation and recruitment to OCGs;
- **Objective 2**: Highlighting how the most relevant factors leading to criminalisation and recruitment to OCGs vary across different types of

criminal organisations, i.e. mafias, DTOs, gangs, and other criminal organisations;

- **Objective 3**: Assessing the validity and generalisability of research findings of studies employing different research methods and focusing on different countries.

This systematic review seeks to answer two sets of research questions. The first set refers to the various factors relating to recruitment and criminalisation into different OCGs addressed in the literature (Objectives 1 and 2); the second set refers to the validity and generalisability of the findings resulting from different methods and geographic area of interest (Objective 3):

- *What are the most commonly reported social, psychological, and economic factors leading to criminalisation and recruitment to OC networks? Are them to be viewed independently of one another? Are there relevant differences among OCGs in terms of factors leading to criminalisation and recruitment?*
- *Which methods do included studies apply? Are finding consistent through studies employing different methods and adopting different geographic scopes?*

2. Data and methods

2.1. Types of Organised Crime Groups

This systematic review frames OCGs into four categories: mafias, DTOs, gangs, and a residual category labelled "other criminal organisations".

Mafias

Although "mafia" has been used as a synonym for OC, the mafias constitute one specific form of OC. Besides sharing the features of OC, they are characterised by (i.) a remarkable longevity, (ii.) an organisational and cultural complexity highlighting the honour of their members and relying on the respect of the code of silence (i.e. *omertà*), and (iii.) the ability to control legitimate markets as well as to exercise a political dominion over their areas of settlement, frequently using private violence and simultaneously supplying protection (Paoli, 2014b, p. 121; see also Gambetta, 1996; Finckenauer, 2007). In this perspective, Varese (2010b) stresses the differences between the mafias and other OCGs, focusing on the various protection services that it can offer to clients, which include protection against extortion, competitors, and law enforcement.

Although originally referring to the Sicilian Cosa Nostra, the concept of mafia today extends to other criminal groups and territories. Scholars regard as mafias the Sicilian Cosa Nostra, the Calabrian 'Ndrangheta, the Neapolitan Camorra, the Italian-American La Cosa Nostra, the Japanese Yakuza, the Chinese Triads, and the Russian mafia (Chu, 2000; Gambetta, 1996; Hill, 2003; Varese, 2005). Besides being active in their territory of origin, these groups have established their presence worldwide. For instance, nowadays the Calabrian 'Ndrangheta is one of the most powerful OCG present across several Italian regions (particularly in Northern Italy), as well as beyond Italian borders (particularly Germany, Switzerland, Canada, and Australia) (Calderoni, Berlusconi, Garofalo, Giommoni, & Sarno, 2015; Campana, 2013; Sarno, 2014; Sciarrone & Storti, 2014; Sergi & Lavorgna, 2016; Transcrime, 2013; Varese, 2006).

Drug trafficking organisations

DTOs are "complex organizations with highly defined command-and-control structures that produce, transport, and/or distribute large quantities of one or more illicit drugs" (U.S. Department of Justice, 2010, p. 10). This market is particularly attractive for OCGs as illicit drugs are "the highest value illicit

commodities trafficked internationally, by quite a wide margin" (UNODC, 2010, p. 275).

Empirical studies have demonstrated that a variety of criminal actors participate in the global trafficking of such goods, including OCGs (Benson & Decker, 2010; Desroches, 2007; Dorn, Levi, & King, 2005; Eck & Gersh, 2000; Natarajan, Zanella, & Yu, 2015; Pearson & Hobbs, 2001). DTOs operate in a business-like structure, with a clear internal hierarchy and subdivision of tasks and a shared values and accepted behaviours among members (Clapper, 2015; Knox & Gray, 2014). Like other global supply chains, DTOs have become increasingly decentralised, diversified, and complex: their internal roles include franchisees, precursor and retail suppliers, local and wholesale distributors, cross-border smugglers and logistical facilitators (Miró, 2003). To protect their trading operations, they need to exercise violence. The frequent emergence and disruption of DTOs, as well as their expansion of their areas of interest, intensifies conflict and competition among them, thereby contributing to the frequent use of violence (Astorga & Shirk, 2010).

GANGS

The conceptual definitions of gang and gang membership have long sparked the debate among scholars and policy makers. More recently, a definition adopted by the Eurogang research network has reached consensus among several researchers (see Curry, 2015).[3] However, this definition considers as gangs those groups mainly composed of youth delinquents. Given the important share of adult offenders in OC and the relevance of the ties to the legitimate world, the systematic review will exclude youth (street) gangs and prison gangs. The literature generally considers youth street gangs as different from OCGs (Decker & Pyrooz, 2014). Furthermore, a recent systematic review has already assessed the factors leading to youth gang membership (Higginson, Mazerolle, Benier, & Bedford, 2014). Prison gangs occur in a specific and institutionalised settings and recruitment is influenced by contextual factors (Blevins, Johnson Listwan, Cullen, & Lero Jonson, 2010; Wood, Alleyne, Mozova, & James, 2014). While there is a relevant literature on prison gangs, this field is mostly separate from the literature on OC, which emphasises the social embeddedness and the role of ties with the legitimate world.

---

[3] The Eurogang research network is an international research network of American and European scholars interested in studying gangs outside the U.S.

Consequently, this systematic review adopts the following working definition of criminal gang: "a group of people following a common code of conduct, having common beliefs and identifiers, existing in a semi-structured organisation or hierarchy, and attempting to accomplish their goals through criminal activity" (Langston, 2003, p. 2). It therefore encompasses studies on criminal gangs composed of adults with different degrees of organisational structure, like Latin American gangs, and street gangs.

OTHER CRIMINAL ORGANISATIONS

Other criminal organisations is a residual category that includes all criminal organisations do not fit within the three previous categories. This category refers to groups that "(i.) utilize violence or threats of violence, (ii.) provide illicit goods that are in public demand, and (iii.) assure immunity for their operators through corruption and enforcement" (Hagan, 2015, p. 395). These comprise for instance outlaw motorcycle gangs and other OCGs active in specific world regions (e.g. the Balkans).

The above overview highlights that researchers have studied several forms of OCGs from different perspectives. Nowadays, the number of studies on OC grows and several themes are under debate. Among them, Kleemans (2014) identifies the study of criminal careers of OC offenders as one of the main emerging theoretical issues. In line with this argument, this systematic review aims at identifying the social, psychological, and economic factors relating to recruitment and criminalisation to OC networks. Since the recruitment into OCGs has received scarce attention, this study adopts a broader perspective: all factors associated with OCGs members and playing a role in their criminal career were deemed to be crucial also in their recruitment into OCGs.

2.2. Identification of the sources

This review applies a systematic approach in identifying and then selecting relevant sources. All literature reviews aim at making sense of a large bodies of information on an issue, but the traditional approach may incorporate some biases. These may derive from the authors' prior knowledge about certain research topics or their arbitrary choices about the relevance of the sources. On the contrary, systematic reviews include all available empirical evidence on a specific topic and apply scientific criteria in the selection of relevant sources, thus providing readers with comprehensive and reliable information (Petticrew & Roberts, 2008).

This review relied on a three-fold query structure ensuring systematic, thorough, and efficient results. Since the aim is to collect all information on

social, psychological, and economic factors relating to the recruitment into mafias, DTOs, gangs, and other criminal organisations, the queries incorporated all these aspects. The search terms from each of the three main categories (i.e. type of OCGs, the type of factors, and recruitment) combined formed the queries (Figure 1). The Boolean Operator "OR" connected keywords pertaining to the same category, while the Boolean Operator "AND" connected keywords from different categories (see Methodological annex). This query structure ensured to retrieve all the studies containing at least one term from each word category.

Figure 1 – Query structure

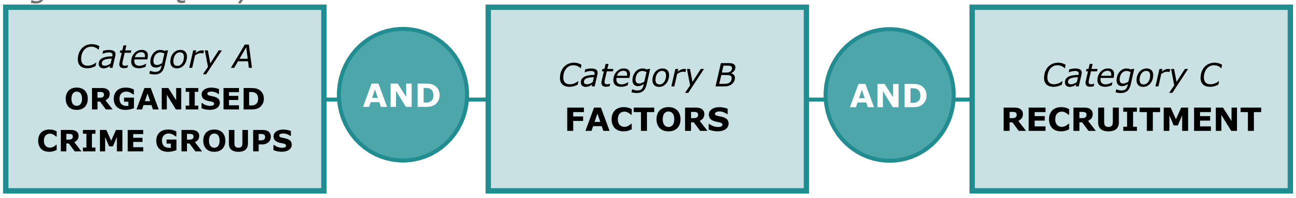

Given the transdisciplinary approach of this systematic review, the search for relevant studies relied on 12 databases relating to different research disciplines. The suitable studies encompassed academic and grey literature written in English, French, German, Italian, and Spanish, and pertaining to social, psychological, and economic disciplines, without limitations as to their year of publication or geographic origin. Table 1 reports the list of databases and sub-databases, indicating the language and technique applied for the search.

Table 1 – List of databases and search techniques

Language	Database	Sub-database	Search technique
English	EBSCO	Criminal Justice Abstracts	Abstract
	Open Grey		Full-text
	ProQuest	Social Sciences Premium	Abstract
		NJCRS	
		PsycInfo	
		Abi/Inform	
		International Bibliography of the Social Sciences	
		Public Health Database	
		Military Database	
		EconLit	
		PsycArticles	
	PubMed		Title and Abstract
	Scopus		Title, Abstract & Keyword
	Web of Science		Title
French	Google Scholar		Full-text
	Sudoc.Abes		Title
German	Sowiport		Title
Italian	Riviste Web		Full-text
Spanish	Liliacs		Title, Abstract & Subject
	ProQuest	Latin America & Iberia Database	Full-text

2.3. Selection of relevant studies

The identification of the queries and databases led to the collection of 48,731 unique studies that were screened for their eligibility for this systematic review (Figure 2).[4] A team of trained researchers applying shared criteria in performing the title and abstract screening of each study: the document must report on OCGs falling within the scope of this systematic review, addressing the social, psychological, and economic factors leading to the recruitment into them, and making an original research contribution (e.g. excluding news articles or reviews of any type).[5]

Out of the initial studies, 48,613 did not meet the inclusion criteria. The team searched for the full texts of the 118 remaining studies, failing to retrieve only few of them. The research team identified additional literature from the bibliographies of the downloaded studies. Moreover, several experts in the field of OC suggested further studies that could be relevant for the scope of this systematic review.[6] These activities resulted in 130 studies available for eligibility based on full-text screening.

The full-text screening process allowed to retain only the studies meeting stricter criteria. Firstly, only empirical studies were included. A detailed classification of the approach, data and methods, and findings of each of the remaining 57 empirical studies allowed further screening. This systematic review included studies adopting quantitative, qualitative, or mixed-methods approaches, provided that they have a clear research aim and appropriate research methodology, design, recruitment strategy, and data collection.[7] As a result, 47 studies met all inclusion criteria (Figure 2).

---

[4] As explained in detail in the Methodological annex, the initial 50,291 records included duplicate studies.

[5] The Methodological annex, reports a detailed description of the screening process presented in this section.

[6] The experts that contributed to this systematic review are: Jay Albanese (Virginia Commonwealth University, USA), Paolo Campana (University of Cambridge, UK), Scott Decker (Arizona State University, USA), Edward Kleemans (Vrije University of Amsterdam, NL), Klaus Von Lampe (John Jay College of Criminal Justice, USA), Carlo Morselli (University of Montreal, CA), Letizia Paoli (Katholieke Universiteit Leuven, BE), David Pyrooz (University of Colorado Boulder, USA), Sonja Wolf (Centro de Investigación y Docencia Económicas, MEX).

[7] The included studies meet the quality criteria set out by an adapted version of the Critical Appraisal Skills Programme (2017) for qualitative studies (see Methodological annex).

Figure 2 – Flowchart of search and screening process

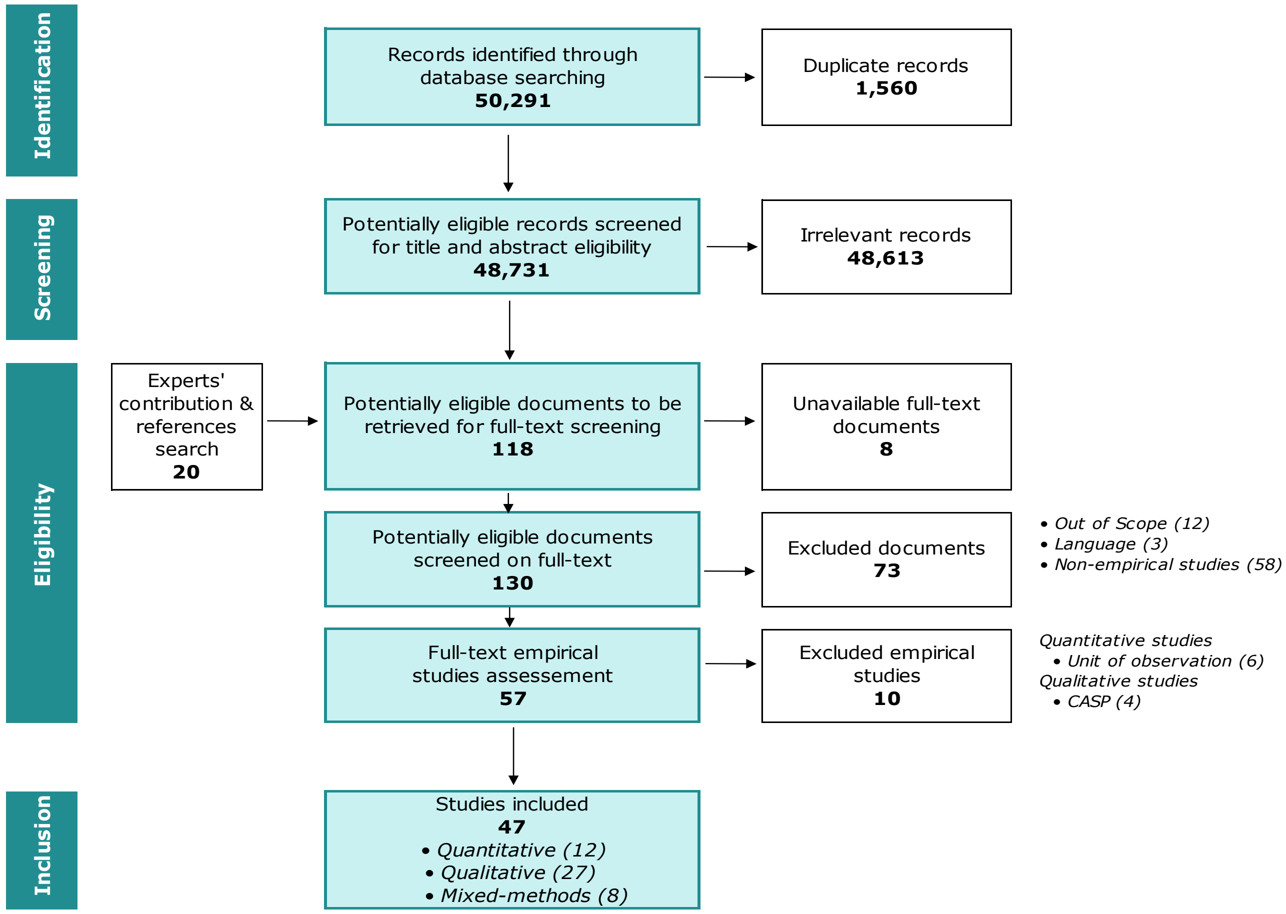

2.4. Corroboration of findings

This systematic review relied on grey literature to validate the results on the social, psychological, and economic factors relating to the recruitment into OCGs. Inclusion of grey literature allows to "take into account some important contextual information, without losing the level of rigor required for a systematic review" (Benzies, Premji, Hayden, & Serrett, 2006, p. 59). The grey literature taken into consideration consisted of all available reports issued by the Italian governmental, judicial, and law enforcement institutions on the presence of the Italian mafias in Italy and abroad, namely the Italian Parliamentary Anti-Mafia Commission (CPA), the Italian National Anti-Mafia and Counterterrorism Directorate (DNA), and Anti-Mafia Investigative Directorate (DIA). These sources are relevant for this review as the mafias have some peculiarities differentiating them from the other types of OCGs.

Only reports containing relevant information for the purposes of this review were used to corroborate results, namely 8 reports issued by the CPA, 9 by the DNA, and 23 by the DIA (see Methodological annex). Overall, the studies included in the systematic review and the Italian grey literature were consistent in identifying the factors relating to recruitment into OCGs.

3. Findings

3.1. Description of the included studies

The included studies were published between 1969 and 2017, spanning different geographical regions and languages. They also differ according to the type of sources: 6% (n=3) are dissertations, 36% (n=17) are books or chapters, and 58% (n=27) are journal articles. The articles come from 19 journals, mostly pertaining to criminology and related fields of studies (Figure 3).

Figure 3 – Types of sources and number of articles per journal (n=47)

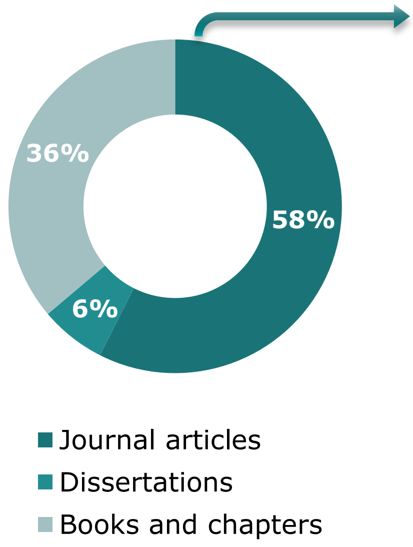

Journal	N. of articles
British Journal of Criminology	3
Crime, Law & Social Change	3
Trends in Organized Crime	3
European Journal of Criminology	2
Global Crime	2
Canadian Journal of Criminology	1
Criminal Behaviour and Mental Health	1
Criminology	1
European Sociological Review	1
International Journal of Social Psychology	1
Journal of Criminal Justice	1
Journal of Economic Behavior & Organization	1
Justice Policy Journal	1
Law & Society Review	1
Policing: An International Journal of Police Strategies & Management	1
Research and Reports in Forensic Medical Science	1
Revista Electrónica de Ciencia Penal y Criminología	1
Social Problems	1
The Howard Journal of Crime and Justice	1

Source: authors' elaboration of the studies included in this systematic review (see Methodological annex for details).

The selected studies rely on different types of data collection and methods of analysis. In investigating the social, psychological, and economic factors relating to the recruitment into OCGs, they use both primary and secondary data, and often the combination of the two (Figure 4, on the left). Due to the difficulties to study the recruitment into OCGs, almost half of the studies (49%, n=23) relied on the information coming from official data, investigative and judicial files, as well as other documents (e.g. bibliographies and tapes). The remaining half of the studies either integrated information from secondary data with interviews (23%, n=11), or conducted their research entirely through surveys, interviews, and conversations with informants (28%, n=13).

Figure 4 – Types of data collection and methods of analysis (n=47)

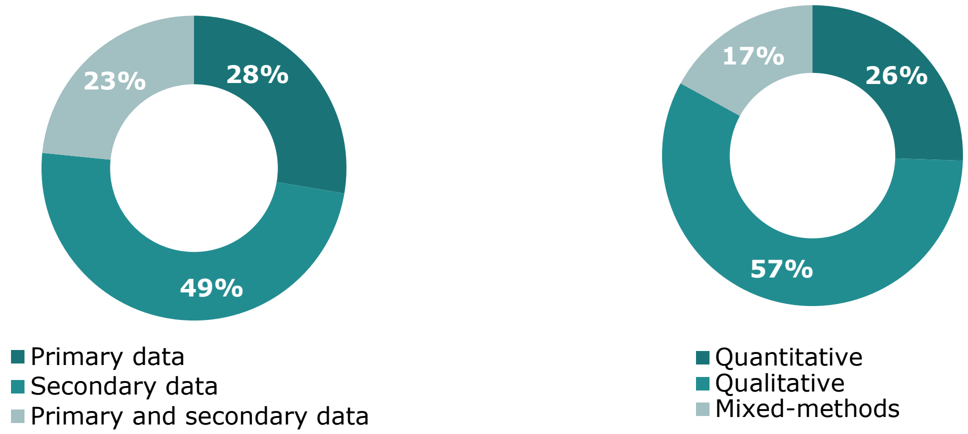

Source: authors' elaboration of the studies included in this systematic review (see Methodological annex for details).

The studies applied different methods of analysis (Figure 4, on the right). Most of them favoured a qualitative approach in dealing with the topic of factor relating to the recruitment into OCGs. The final literature list consisted of 12 quantitative (26%), 27 qualitative (57%), and 8 mixed-methods (17%) studies (see Table 3, Table 4, and Table 5, respectively). The scarcity of quantitative studies could be due to the fact that the topic of the research itself, i.e. the recruitment into OCGs, is not particularly suitable for such methodological approach. The quantitative approach is further hindered by the difficulties in operationalising and measuring the social, psychological, and economic factors intervening in the recruiting process.

The included studies also differ regarding the type of OCGs they focus on (Figure 5). Almost half of them (42%, n=20) deal with the mafias, especially with the Italian ones, 30% (n=14) discuss about other criminal organisation, above all Dutch ones, and the remaining about gangs (15%, n=7), and DTOs (13%, n=6).

Figure 5 – Types of OCGs studied (n=47)

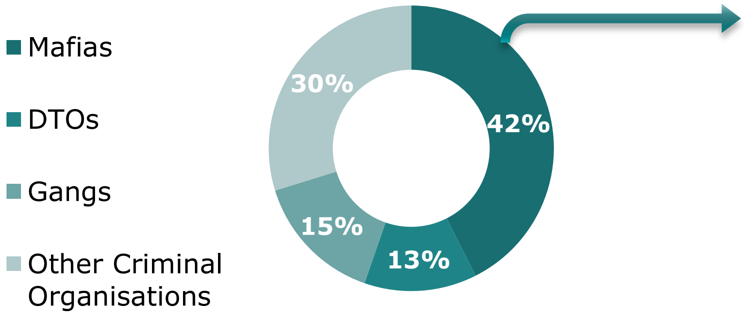

Type of mafia	N. of studies
Cosa Nostra	7
'Ndrangheta	4
Camorra	3
Russian mafia	3
La Cosa Nostra	2
Chinese Triads	1

Source: authors' elaboration of the studies included in this systematic review (see Methodological annex for details).

Notwithstanding the differences among the included studies, each of them reported on the social, psychological, and/or economic factors associated with recruitment of individuals to OCGs. After carefully reading all the studies, the research team created categories of factors to organise the relevant information and systematise the knowledge on this field of study (Table 2).

Table 2 – Categories of socio-economic and psychological factors associated with recruitment of individuals to OCGs

Category of factors	Explanation of included factors
**Age**	Factors on the age at which individuals are likely to be recruited into OCGs (e.g. youngsters)
**Gender**	Factors on the differences between genders in being involved in OCGs (e.g. majority of OCGs members are males)
**Ethnicity**	Factors on the ethnic features of OCGs members (e.g. ethnic homogeneity)
**Educational background**	Factors on the level of education of individuals recruited by OCGs (e.g. illiteracy)
**Employment**	Factors on the how the lack of employment or the presence of specific work settings can be facilitators for joining OCGs (e.g. employees of logistic companies)
**Economic conditions**	Factors on how certain economic conditions can facilitate recruitment into OCGs (e.g. poverty)
**Social ties**	Factors on the relations that provide opportunities for being involved in OCGs (e.g. family ties)
**Group identity**	Factors on the values and subculture shared by OCGs members (e.g. honour)
**Psychological factors**	Factors on disorders related to the recruitment into OCGs (e.g. being antisocial)
**Criminal background and skills**	Factors on how previous criminal records, specific skills and attitudes can increase the opportunities to enter OCGs (e.g. risk-taking behaviours)
**Silence/*Omertà***	Factors on the ability of enforcing the code of silence for being part of OCGs

The total number of factors identified across the 47 included studies is 184. The identification of factors in each study and their systematisation into 11 categories allows to analyse how many times they are reported (Figure 6). The most recurring factors are those related to social ties and criminal background and skills, which respectively appear 38 and 35 times throughout the included studies.

Figure 6 – Number of studies reporting on factor categories (n=184)

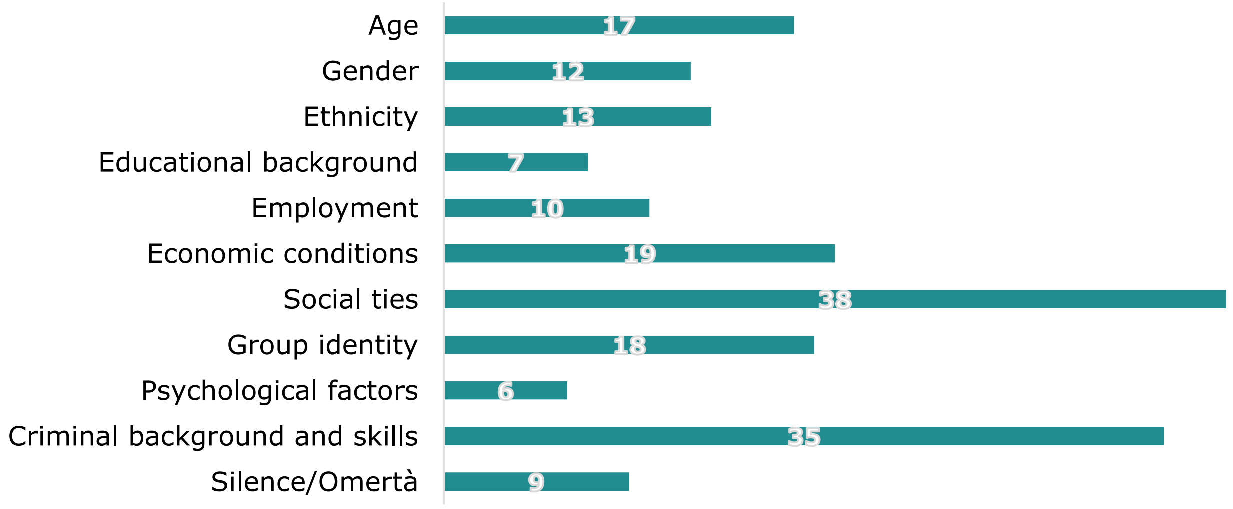

Source: authors' elaboration of the studies included in this systematic review (see Methodological annex for details).

Figure 7 shows the main geographic area where each category of factors was assessed (i.e. each study reports on factors characterising the recruitment into the OCGs active in that region). It shows that Italy registers the highest number of factors for almost all categories.[8] This could be the due to the fact that Italy is the territory of origin of several mafia groups. This assumption is confirmed by the fact that the category silence/*omertà* is present only in Italy and North America, where the Sicilian mafia expanded (i.e. La Cosa Nostra). Figure 8 highlights the different methodological approaches adopted by the studies reporting on each category of factors. As already underlined, the qualitative approach seems to fit better the study of the factors relating to recruitment into OCGs. Figure 9 shows how the categories of factors spread out across the different OCGs. Most factors relate to the mafias, as they are a typical OCG that is present in different regions and has some specific shared features. For instance, they rely on the kinship and blood ties among members and their ability to respect the code of silence (i.e. *omertà*).

---

[8] For this reason, Italy is separated from the other EU countries in the subdivision into geographic areas.

Figure 7 – Geographic distribution of the factor categories (n=184)

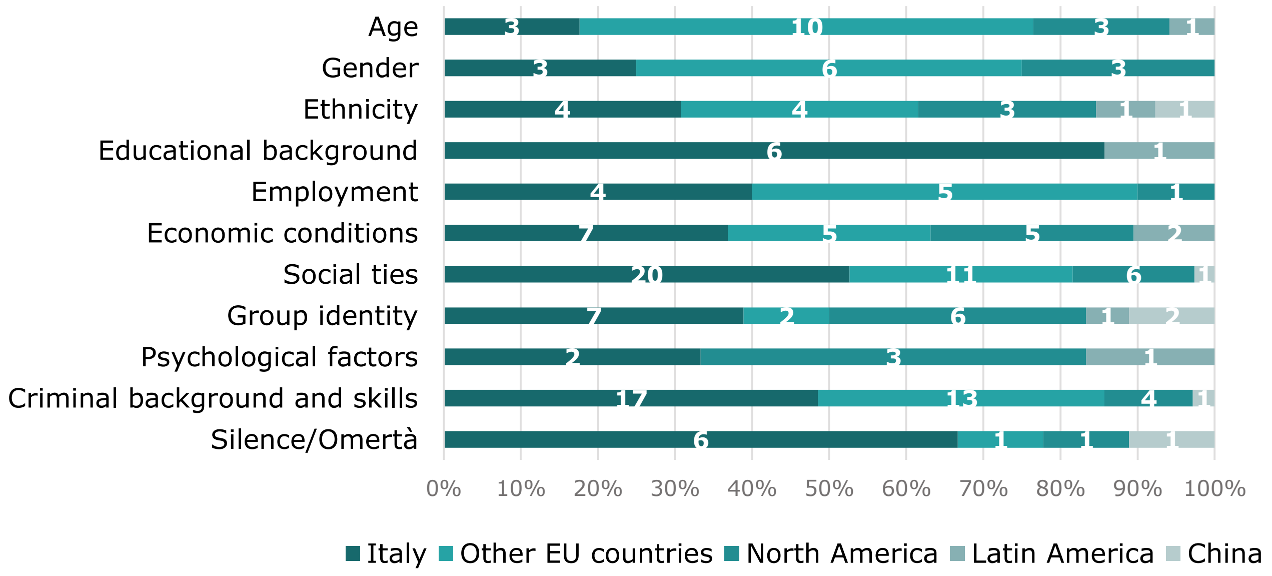

Source: authors' elaboration of the studies included in this systematic review (see Methodological annex for details).

Figure 8 – Factor categories per type of study (n=184)

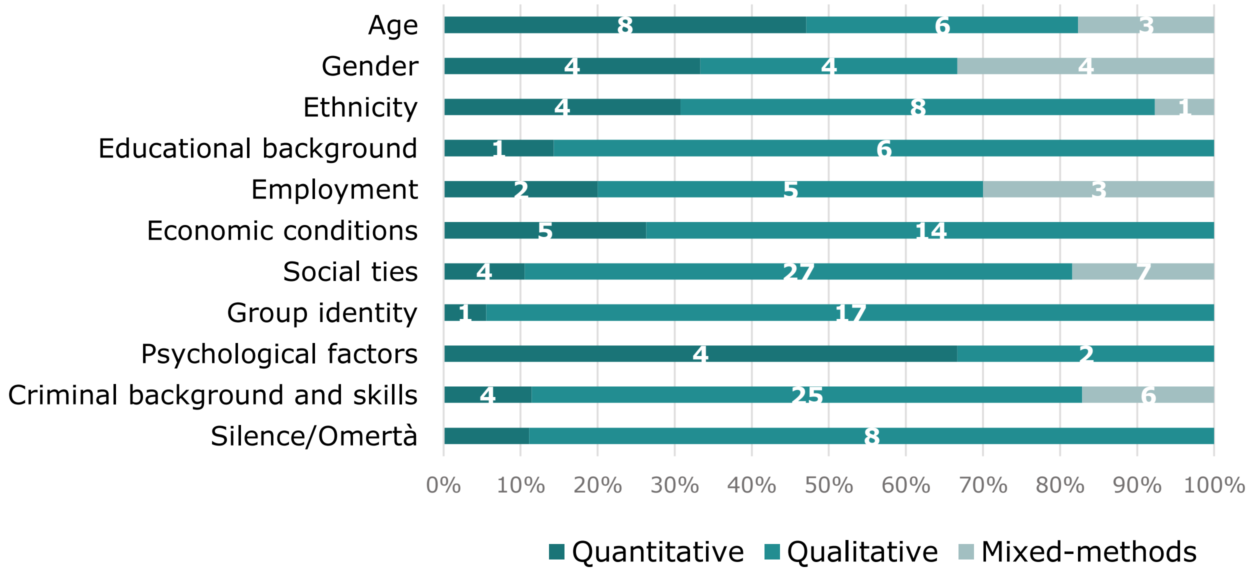

Source: authors' elaboration of the studies included in this systematic review (see Methodological annex for details).

Figure 9 – Factor categories per type of OCG studied (n=184)

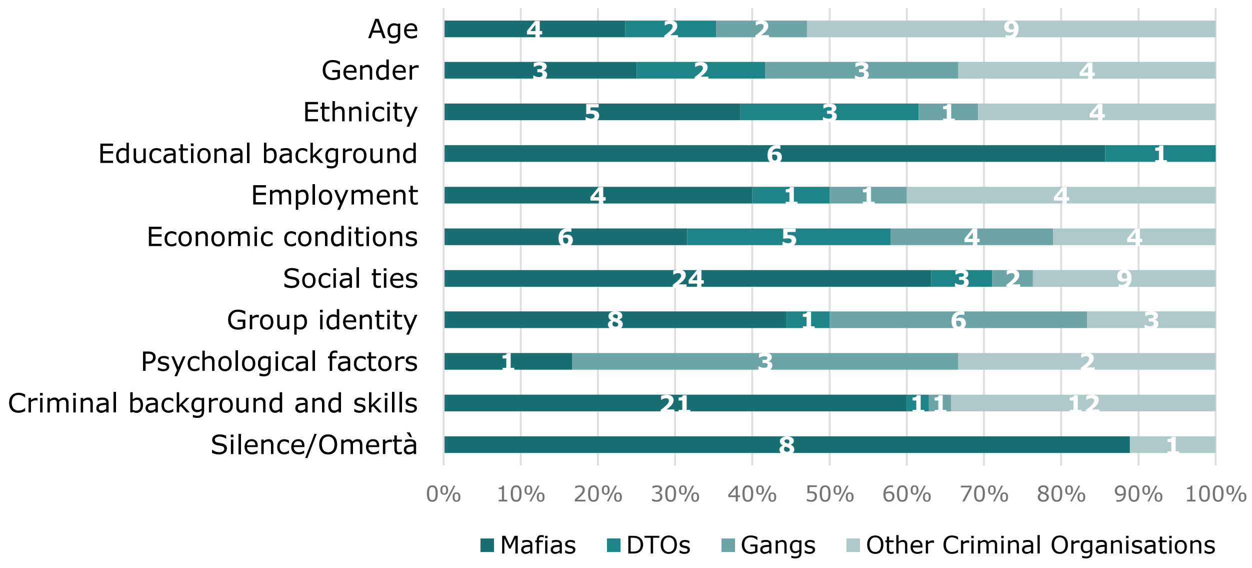

Source: authors' elaboration of the studies included in this systematic review (see Methodological annex for details).

Findings identified by the Italian grey literature were consistent with the ones reported by included studies on the mafias (Figure 10 and Figure 11).

Figure 10 – Number of Italian grey literature reports by factor categories (n=162)

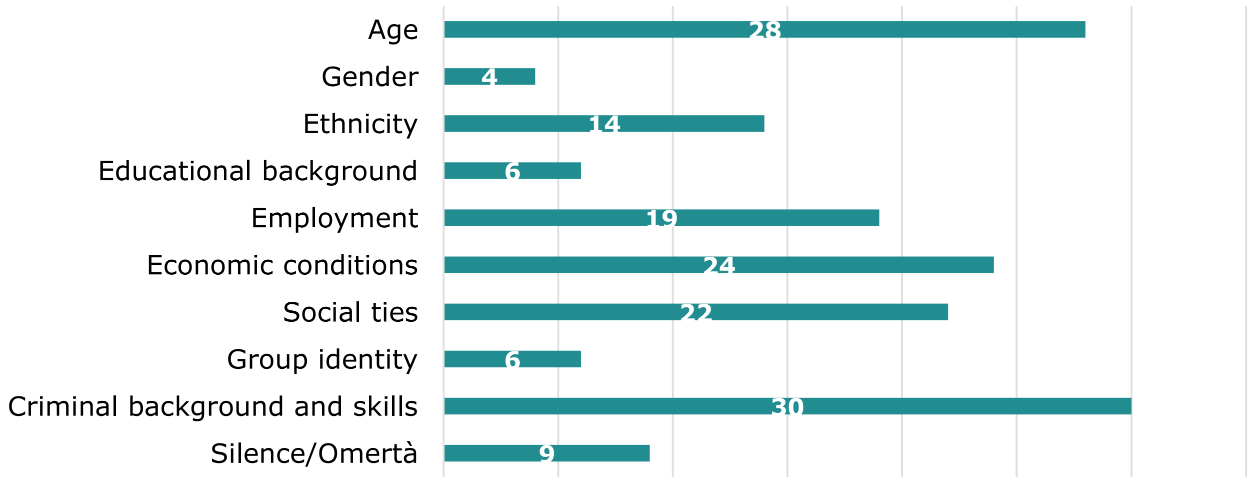

Source: authors' elaboration of the Italian grey literature used in this systematic review (see Methodological annex for details).

Figure 11 – Factor categories by Italian grey literature sources (n=162)

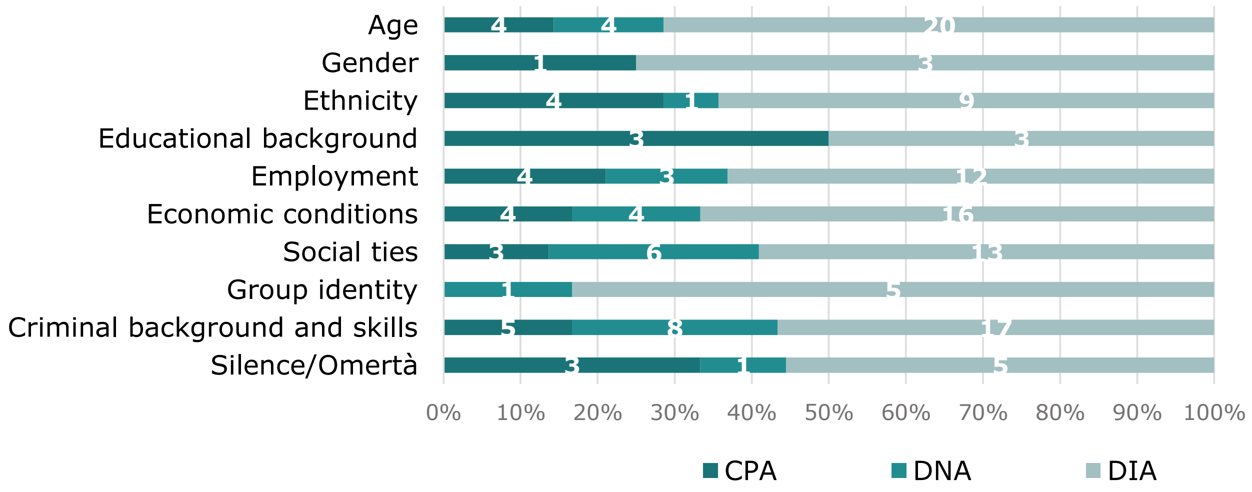

Source: authors' elaboration of the Italian grey literature used in this systematic review (see Methodological annex for details).

3.2. Social, psychological, and economic factors relating to recruitment to Organised Crime Groups

Age

The literature often reports the age of individuals' involvement into OC. In identifying age of onset, scholars point out both the involvement of youngsters (Arlacchi, 1983; Arsovska, 2015; Behan, 1996; Blokland, van Hout, van der Leest, & Soudijn, 2017; Carvalho & Soares, 2016; Ciconte, 1992; Cressey, 1969; Hixon, 2010; Kirby, Francis, Humphreys, & Soothill, 2016; Ostrosky et al., 2012) and adults (Blokland et al., 2017; Kissner & Pyrooz, 2009; Kleemans & de Poot, 2008; Salinas & Regadera, 2016; Salinas, Requena, & De la Corte, 2011; Unlu & Ekici, 2012; Van Koppen, Poot, et al., 2010). Though some OCGs tend to recruit more adults than youngsters (e.g. outlaw motorcycle gangs), most of them are composed of both young people and adult members.

Vulnerable young people join OCGs during adolescence and early adulthood because criminal organisations emerge as an attractive lifestyle dominated by the cult of violence and show off of material goods (Behan, 1996; Hixon, 2010; Ostrosky et al., 2012). Risk of being recruited at younger ages is also positively associated with problems at school, drug use, low socio-economic conditions, and the feeling of disillusion for the future (Arsovska, 2015; Behan, 1996; Carvalho & Soares, 2016). As a result, OC offenders are significantly

younger than general offenders in terms of age at first sanction (Kirby et al., 2016).

Individuals engage in OC in adulthood due to the possibility to access profitable criminal activities can occur throughout individuals' lives as a result of social connections and skills acquired with age (Blokland et al., 2017; Kleemans & de Poot, 2008) (see section on Social ties). In some cases, the adult profile of OC offenders is due to the fact that specific OCGs, e.g. outlaw motorcycle gangs, have restricted membership to adults (Blokland et al., 2017).

Gender

Individuals who join OCGs are predominantly males (Brotherton & Barrios, 2004; Hixon, 2010; Kirby et al., 2016; Salinas et al., 2011; Unlu & Ekici, 2012; Varese, 2011a, 2013) and this proportion is even higher when OC offenders are compared with general offenders (Salinas et al., 2011).

Recent studies (Brotherton & Barrios, 2004; Requena, de Juan, Salinas, & de la Corte, 2014; Salinas & Regadera, 2016; Van San & Sikkens, 2017; Varese, 2011a) have focused on the factors that lead women to recruitment into OCGs. Women's participation into OCGs mainly occurs through family or emotional ties, especially within DTOs. In addition, women's role within some OCGs may change from home country to new territories. Studying the Russian mafia in Italy, Varese (2011a, p. 249) points out that women "are allowed to play a greater role in the new setting because they can be trusted and trustworthy individuals are in short supply in the new setting".[9]

Ethnicity

Criminological theories of strain, social disorganisation, and subcultures developed over the last century examined ethnic differences in offending and crime patterns.[10] In line with these theories, ethnic marginality has been found

---

[9] Several studies have investigated the participation of women in OC (Fiandaca, 2007; Fleetwood, 2014; Ingrascì, 2007; Kleemans, Kruisbergen, & Kouwenberg, 2014; Siebert, 1996). In particular, Fiandaca (2007) highlights the role of women in the mafias and other OCGs over a period of 20 years and across countries.

[10] Drawing from the original work of Merton (1938), Agnew (1992) developed the general strain theory (GST) arguing that stressful events may lead to crime. Further studies tested the GTS finding that individuals with some ethnic backgrounds, namely Hispanic and African Americans, are more likely to turn to crime as a result of poverty, disrupted families, and discrimination (Agnew, 2006; Pérez, Jennings, & Gover, 2008; see also Leeper Piquero & Sealock, 2010). Similarly, the social disorganisation theory states that ethnic heterogeneity coupled with low economic status, residential mobility, and family disruption lead to increases in crime rates among neighbourhoods (Shaw & McKay, 1972). Subcultural theories

to be associated with involvement in OC. Individuals can enter OCGs to overcome the marginalisation related with their ethnic minority status (Arsovska, 2015; Gordon, 2000). Moreover, some ethnicities are related with a higher risk of joining certain types of OCGs, e.g. black individuals in drug-trafficking gangs (Carvalho & Soares, 2016).

In addition, the systematic review highlights that ethnic homogeneity is a relevant element for most OCGs (Albini, 1971; Ciconte, 1992; Cressey, 1969; Decker & Chapman, 2008; Hess, 1993; Lupo, 1993; Varese, 2011b, 2013; Wang, 2013; Zhang & Chin, 2002). The illicit nature of OCGs activities and the risk of detection and arrest for their members requires mutual trust relations developed through ethnic ties (Decker & Chapman, 2008; Zhang & Chin, 2002). Ethnic homogeneity not only has favoured the establishment of OCGs within their territory of origin (Arlacchi, 1983; Behan, 1996; Brancaccio, 2017; Ciconte, 1992; Gambetta, 1996; Hess, 1993), but has also contributed to their expansion across countries. With this regard, the literature reports on Italian mafias in the U.S. and Germany, Russian mafias in Italy, and *maras* (i.e. Central American street gangs) and Chinese triads in the U.S. (Albini, 1971; Cressey, 1969; Miguel Cruz, 2010; Sciarrone & Storti, 2014; Varese, 2011a, 2013; Zhang & Chin, 2002).

Despite the prevalence of ethnic homogeneity within OCGs, foreigners or illegal immigrants may also become involved in OC. As Salinas and Regadera (2016, p. 62) indicate, irregular residents' situation "hinders their opportunities to find a legitimated job and explains their deep-rooted involvement in organised crime activities". The Italian grey literature has also reported the recruitment of illegal immigrants into Italian mafias, especially for low-level tasks (e.g. drug retail) (Commissione Parlamentare Antimafia, 1991, 2003, 2006, Direzione Investigativa Antimafia, 2008, 2012a, 2012b, 2013b, 2014a, 2014b, 2015b; Direzione Nazionale Antimafia, 2015).

EDUCATIONAL BACKGROUND

Low education levels are positively associated with individuals' recruitment into OCGs (Albini, 1971; Carvalho & Soares, 2016; Ciconte, 1992; Hess, 1993; Sales, 2015). The Italian grey literature supports this association with frequent reference to the relation between poor education, low economic conditions, and scarce access to legitimate jobs as risk factors for youths' involvement in the mafias (Commissione Parlamentare Antimafia, 2003, 2006, 2012,

---

explain crime involvement among lower-class individuals belonging to ethnic minorities as a result of frustration and/or reaction to the dominant culture (Cloward & Ohlin, 1960; Cohen, 1955; Miller, 1958).

Direzione Investigativa Antimafia, 2000b, 2006, 2014b). Illiterate people from lower socio-economic background are therefore more likely to join OCGs. Their illiteracy does not hinder the accumulation of wealth and eventually a long criminal career as bosses (Hess, 1993).

However, a study suggested that mafia members' educational levels may have increased over time. Arlacchi (1983) highlights that while in the past mafia members were mainly illiterate and lower class individuals, since the 1960s mafiosi include literate middle class individuals. Combined with traditional mafia norms and values, a higher education would allow Mafiosi to exploit illegal business opportunities arising within a market economy. Nevertheless, the growth in mafia members' education levels may simply reflect the general increased education in the overall population during the same period (Roser & Ortiz-Ospina, 2017).

Employment

The lack of legitimate occupations facilitates involvement into OCGs (Brancaccio, 2017; Ciconte, 1992; Gambetta, 1996; Jhi & Gerber, 2015; Sales, 2015; Wang, 2013). A study has found the probability of becoming a gang member almost three times lower for individuals with employment experience than for those who have always been unemployed (Jhi & Gerber, 2015).

Nevertheless, some occupations present specific opportunities for criminal behaviour (Ciconte, 1992; Kleemans & de Poot, 2008; Kleemans & Van de Bunt, 2008; Salinas et al., 2011; Van Koppen, 2013). These occupations share some characteristics, like leaving a certain degree of independence to employees, allowing for social exchange, and being related with mobility and logistics. Several individuals recruited into OCGs work in the transport industry, especially truck drivers, owners of car companies, and car dealers (Kleemans & de Poot, 2008; Kleemans & Van de Bunt, 2008; Van Koppen, 2013). The licit employment can conceal illicit activities, which in turn can complement the legal salary (Salinas et al., 2011).

Economic conditions

Being raised in poor city suburbs and socio-economically deprived environments facilitates recruitment into OCGs (Brotherton & Barrios, 2004; Carvalho & Soares, 2016; Ciconte, 1992; Decker & Chapman, 2008; Gordon, 2000; Jhi & Gerber, 2015; Sergi, 2016; Van San & Sikkens, 2017). Individuals from low socio-economic status may drop out of school to contribute to the family expenses and to improve their living conditions. In turn, they may be attracted to criminal activities as a way to pursue material and emotional reward (Gordon, 2000; Ostrosky et al., 2012). This mechanism explains the

continuous recruitment of individuals into Italian mafias. Youngsters living in suburbs in conditions characterized by lack of money, goods, or means of support are fascinated by the advantages offered by a career into mafias (Albini, 1971; Arsovska, 2015; Behan, 1996; Brancaccio, 2017; Hess, 1993; Sales, 2015). The Italian grey literature has indeed frequently underlined how mafias can easily recruit numerous new members from economically disadvantaged areas (Commissione Parlamentare Antimafia, 1994, 2003, 2006, 2012, Direzione Investigativa Antimafia, 2000a, 2000b, 2003, 2006, 2008, 2010a, 2010b, 2011, 2012b, 2013a, 2013b, 2014a, 2014b, 2015a, 2016a, 2016b, Direzione Nazionale Antimafia, 2000, 2002, 2005, 2006, 2014).

Nevertheless, individuals from higher socio-economic conditions can also join OCGs, mainly attracted by monetary gain (Gordon, 2000; Unlu & Ekici, 2012; Van Koppen, 2013; Wang, 2013). Involvement in criminal activities, particularly in OC activities, ensures high incomes. This could be a factor facilitating recruitment of individuals facing debts, specific needs (e.g. drug addiction), or difficult life events (e.g. sudden illness) (Van Koppen, 2013).

SOCIAL TIES

Not only social relations are "the least common denominator of organized crime" (McIllwain, 1999, p. 304), but they also represent a factor facilitating the involvement into criminal activities. Several studies underline how kinship and blood ties (Arlacchi, 1983; Arsovska, 2015; Behan, 1996; Brancaccio, 2017; Ciconte, 1992; Cressey, 1969; Decker & Chapman, 2008; Gambetta, 1996; Kissner & Pyrooz, 2009; Lo, 2010; Requena et al., 2014; Salinas & Regadera, 2016; Salinas et al., 2011; Sciarrone, 2014; Sergi, 2016; Van Koppen, 2013; Varese, 2001, 2006, 2011a), as well as social ties developed in disparate spheres of everyday life (e.g. leisure and work ties) can facilitate access to OC (Decker & Chapman, 2008; Gambetta, 1996; Gordon, 2000; Kleemans & de Poot, 2008; Kleemans & Van de Bunt, 2008; Morselli, 2003; Paoli, 2003; Sales, 2015; Van Koppen, 2013; Van San & Sikkens, 2017; Varese, 2001, 2011b, 2013; Wang, 2013).

Being part of an OCG results from cultural transmission and gradual learning, therefore kinship and blood ties have a crucial role in the recruitment of new members (Gordon, 2000; Sergi, 2016). An empirical study investigating the relation between gangs and learning mechanisms reports that parental gang membership significantly predicted individuals' gang membership (Kissner & Pyrooz, 2009). As criminals cannot rely on third parties to regulate their businesses and disputes, they prefer to associate with family members or close friends (Van Koppen, 2013). Being part of a criminal family may *per se* facilitate one's recruitment into OCGs, especially in the mafias (Albini, 1971;

Arlacchi, 1983; Ciconte, 1992; Gambetta, 1996; Hess, 1993; Lupo, 1993; Paoli, 2003; Sciarrone, 2014; Sergi, 2016). In these criminal settings, the concept of family plays a primary role. Besides exploiting kinship and blood ties, individuals can join such OCGs through the acceptance of a "status contract": through their affiliation ceremony (which in some Italian mafia is called "baptism"), they assume a permanent new identity as "men of honour". During this ceremony, several rituals help creating brotherhood ties among members, thus also enabling "contract of fraternization". This contract extends the bonds of loyalty and obligation beyond the family ties, providing members with mutual support and trust necessary to pursue groups' aims and satisfy the needs of each of them (Paoli, 2003).

Social relations developed in various areas of individuals' lives can also facilitate their recruitment into OCGs. Kleemans and De Poot (2008, p. 75) proposed the concept "social opportunity structure" to refer to "social ties providing access to profitable criminal opportunities". Individuals can join criminal settings because of their experience and expert knowledge and/or ability to connect the criminal members with other third parties, like other criminal groups or representatives of legal businesses (Morselli, 2003; Varese, 2011a). Work, social, and criminal ties are therefore interwoven. Leisure activities and side-lines often act as a catalyst for contacts between representatives of the legal and criminal settings (Kleemans & de Poot, 2008; Kleemans & Van de Bunt, 2008; Van Koppen, 2013). The social opportunity structure may explain why some individuals get involved into OCGs in adulthood: their expertise meets a specific criminal opportunity, and they seize that opportunity. Individuals can indeed change their attitude towards criminal opportunities due specific life events (e.g. as bankruptcy) (Kleemans & de Poot, 2008; Van Koppen, 2013).

Group identity

Individuals who join OCGs belong to subcultures characterised by values as honour and loyalty, and are recruited through ritualised procedures (Albini, 1971; Arlacchi, 1983; Brotherton & Barrios, 2004; Cressey, 1969; Gambetta, 1996; Hess, 1993; Hixon, 2010; Lo, 2010; Paoli, 2003; Sciarrone, 2014; Sergi, 2016; Zhang & Chin, 2002). In addition, a major factor leading individuals to recruitment into OCGs is the sense of belonging and of social identity provided by the criminal group (Arsovska, 2015; Brotherton & Barrios, 2004; Densley, 2012; García, 2006; Hixon, 2010; May, 2009).

Several included studies have highlighted the ceremonies of initiation to OCGs, especially in the mafias (Albini, 1971; Gambetta, 1996; Hess, 1993; Hixon, 2010; Paoli, 2003; Sergi, 2016). To be recruited into the mafias, individuals

must have psychic attitudes and a moral code typical of “men of honour”, who are respected and capable of violent revenge if necessary (Gambetta, 1996; Hess, 1993). During initiation ceremonies, members have to pronounce an oath in front of other associates, together with the “promise of providing for the family of a members in the event of his death or incarceration” (Albini, 1971, pp. 113–114; see also Paoli, 2003; Sergi, 2016). Strong sense of loyalty and social cohesion among individuals are further reinforced through social interactions within the OCGs and mutual offending behaviour (Densley, 2012). These processes enhance members’ sense of group identity (García, 2006).

PSYCHOLOGICAL FACTORS

Some studies have revealed that childhood conduct disorder, substance use disorder, and/or abnormal psychopathological traits increase individuals’ risk of involvement in OC activities (Hixon, 2010; Kissner & Pyrooz, 2009; May, 2009; Ostrosky et al., 2012; Schimmenti, Caprì, La Barbera, & Caretti, 2014; Sergi, 2016). Individuals recruited into OCGs usually have an extensive history of negative and arrested development during adolescence, resulting in antisocial personality disorders during their adulthood (Hixon, 2010).

Most OC members register medium–high psychopathy level, in line with their criminal skills. They show lack of empathy and inhibition, marked cruelty and callousness, affective impairments, an average antisocial lifestyle. These features allow them to engage in violent and risky activities and commit physical assaults for immediate gratification, regardless of potential prosecution (Ostrosky et al., 2012). Another study confirmed the psychopathy and antisocial traits characterizing mafias’ members, also underlying their infantile and dependent personality traits, demonstrated by their obedience to the willingness of their organisations. Mafia members differ from other offenders in that they do not suffer from substance use disorder. Substance use may indeed impair the reliability of mafia members, who always have to display the honourable principles of their organisations (Schimmenti et al., 2014).

Nevertheless, criminals involved in “high-profile” activities, like money-laundering, present different characteristics. These individuals maintain quite a normal lifestyle, but they lack affective bonds, they have increased arrogance, callousness, and a strong desire for recognition and for a certain economic status. These features allow them to achieve their goals through unethical and illicit nonviolent activities (Ostrosky et al., 2012).

CRIMINAL BACKGROUND AND SKILLS

Individuals who aim at joining OCGs must prove to have violent attitudes, making people with a criminal background more likely to be recruited by OCGs (Albini, 1971; Arlacchi, 1983; Behan, 1996; Blokland et al., 2017; Brancaccio, 2017; Gambetta, 1996; Hess, 1993). Several included studies have highlighted the long criminal history of OC members (Albini, 1971; Arsovska, 2015; Blokland et al., 2017; Brancaccio, 2017; Cressey, 1969; Decker & Chapman, 2008; Densley, 2012; Gambetta, 1996; Kirby et al., 2016; Kleemans & de Poot, 2008; Lupo, 1993; Morselli, 2003; Sales, 2015; Salinas et al., 2011; Van Koppen, de Poot, & Blokland, 2010; Van Koppen, de Poot, Kleemans, & Nieuwbeerta, 2010; Varese, 2011b). In some cases OCGs seeking for specific criminal skills, or for a special expertise required for certain illegal activities, may recruit individuals with existing criminal records in prison (Behan, 1996; Ciconte, 1992), or outside (Arlacchi, 1983; Cressey, 1969; Gambetta, 1996; Lo, 2010; Salinas & Regadera, 2016; Salinas et al., 2011; Sergi, 2016; Van Koppen, 2013).

Attitudes towards violent and risk-taking behaviours are essential to be part of OCGs. Behan (1996, p. 52) argues that, for the Neapolitan Camorra, "perhaps the most potent ideological weapon [is] the cult of violence." Violence must not only be ideological, but also used efficiently to reinforce one's criminal status (Albini, 1971). For this reason, individuals' recruitment often takes place among those with existing criminal records (Blokland et al., 2017; Kirby et al., 2016; Requena et al., 2014). Comparing OC offenders with general offenders, Van Koppen and colleagues (2010, p. 370) highlight that "organized crime offenders […] have a more serious criminal history in terms of offence seriousness" and "are imprisoned three times longer than offenders in the comparison group of general offenders". This results in the prison environment facilitating the chances of recruitment into OCGs. Behan (1996) and Ciconte (1992) underline the positive association between prison background and recruitment into OCGs, as individuals seek for protection and personal safety within the penitentiary.

Possession of criminal skills or special expertise is another relevant feature to be recruited into OCGs. The former refers to the ability of avoiding police detection and allows OCGs to test the loyalty and criminal potential of individuals (Densley, 2012). The latter refers to high levels of knowledge, or the right set of skills, making individuals suited for certain types of illegal businesses. Special expertise can derive from criminal or legitimate experience (Van Koppen, 2013; Salinas & Regadera, 2016). In this regard, Gambetta (1996, p. 66) points out that "occasionally recruitment occurs when there is a need for someone with special expertise, […] someone skilled in handling

explosives or in the case of drug dealing, a chemist. Even a translator can become crucial […].” Having a special expertise, either from criminal or legitimate experience, also explains the involvement in OCGs during adulthood (Kleemans & de Poot, 2008).

Silence/Omertà

Individuals joining OCGs cannot forgo the code of silence, also known as *omertà*, required to commit OC activities (Albini, 1971; Ciconte, 1992; Cressey, 1969; Gambetta, 1996; Hess, 1993; Lo, 2010; Lupo, 1993; Paoli, 2003; Requena et al., 2014). Albini (1971, pp. 107–108) underlines that “*omertà* is not unique to any one organization or society. *Omertà* is a behavior and attitude [and] […] it represents suspicion and resentment of government and law”. The relevance of adhering to a code of silence to join OCGs has frequently been underlined by Italian grey literature on the mafias in Italy and abroad (e.g. in the U.S.) (Commissione Parlamentare Antimafia, 2003, 2006, 2012, Direzione Investigativa Antimafia, 2001, 2007, 2009, 2014b, 2016b; Direzione Nazionale Antimafia, 2006).

Table 3 – Details on the included quantitative studies

Source	Type of OCG	Sample*	Data collection	Data analysis	Categories of factors	Geographic scope	Relevant findings
**Blokland et al (2017)**	Other Criminal Organisations	601	Official data	Descriptive statistics<br>T-test<br>Chi square<br>Logistic regression	Age<br>Criminal background and skills	Netherlands	Outlaw motorcycle gangs' membership is positively associated with having a criminal record
**Carvalho and Soares (2016)**	DTOs	230	Interviews	Descriptive statistics<br>Mincerian regression	Age<br>Ethnicity<br>Educational background<br>Economic conditions	Brazil	Individuals join DTOs for monetary returns
**Jhi and Gerber (2015)**	Gangs	190	Survey	Descriptive statistics<br>Logistic regression	Employment<br>Economic conditions	U.S.	Gang membership is positively associated with unemployment
**Kirby et al. (2016)**	Other Criminal Organisations	4,109	Official data	Descriptive statistics<br>Chi square<br>Kruskal-Wallis test	Age<br>Gender<br>Ethnicity<br>Criminal background and skills	U.K.	Compared with general crime offenders, OC offenders are more predominantly male, more ethnically heterogeneous and with more drug offences records
**Kissner and Pyrooz (2009)**	Gangs	200	Interviews	Logistic regression	Age<br>Social ties<br>Psychological factors	U.S.	Persistent gang involvement is associated with poor self-control
**May (2009)**	Gangs	138	Survey	Descriptive statistics<br>Correlation<br>Logistic regression	Group identity<br>Psychological factors	U.S.	Gangs constitute a form of social support for members
**Ostrosky et al. (2012)**	OCGs & DTOs	82	Interviews; Documents	Descriptive statistics	Age<br>Economic conditions<br>Psychological factors	Mexico	Individuals join DTOs seeking for a higher economic income and a lifestyle characterised by the accumulation and showing off of material values
**Requena et al. (2014)**	OCGs	200	Investigative files	Descriptive statistics	Gender<br>Social ties<br>Silence/*Omertà*	Spain	Women get involved in OCGs through their personal networks, i.e. family ties and emotional ties
**Salinas and Regadera (2016)**	OCGs	2384	Investigative files	Descriptive statistics	Age<br>Gender<br>Ethnicity<br>Social ties<br>Criminal background and skills	Spain	Individuals get involved in OCGs because they possess special expertise developed outside the criminal world

Source	Type of OCG	Sample*	Data collection	Data analysis	Categories of factors	Geographic scope	Relevant findings
**Schimmenti et al. (2014)**	Mafias	69	Interviews	Descriptive statistics<br>T-test<br>Chi square<br>Logistic regression	Psychological factors	Italy	High levels of antisocial traits and low levels of interpersonal-affective traits of psychopathy characterise mafia members
**Unlu and Ekici (2012)**	DTOs	230	Investigative files	Descriptive statistics<br>Chi square	Age<br>Gender<br>Economic conditions	Turkey	Male adults from low-income societies can join DTOs and work as couriers for monetary returns
**Wang (2013)**	DTOs	222	Interviews	Content analysis<br>CHAID<br>Logistic regression	Ethnicity<br>Employment<br>Economic conditions<br>Social ties	U.K.	Early starters get involved in DTOs because of entertainment expenses, while late starters because of financial difficulties

*The sample reports the number of individuals included in the studies.

Table 4 – Details on the included qualitative studies

Source	Type of OCG	Data collection	Categories of factors	Geographic scope	Main findings
**Albini (1971)**	Mafias	Interviews<br>Informants<br>Documents	Educational background<br>Economic conditions<br>Social ties<br>Group identity<br>Criminal background and skills<br>Silence/*Omertà*	Italy	Recruitment of individuals into OCGs is also based on friendship, kinship, contract, and patron-client relationships
**Arlacchi (1983)**	Mafias	Interviews<br>Investigative files<br>Judicial records	Age<br>Educational background<br>Social ties<br>Group identity<br>Criminal background and skills	Italy	Individuals who get involved in mafias come from the middle-class, have an educational background and possess managerial skills
**Arsovska (2015)**	Other Criminal Organisations	Interviews<br>Investigative files<br>Judicial records	Age<br>Ethnicity<br>Economic conditions<br>Social ties<br>Group identity<br>Criminal background and skills	Balkans	Individuals are recruited into OCGs based on ethnic ties, kinship ties, and display of violent behaviour
**Behan (1996)**	Mafias	Interviews<br>Investigative files	Age<br>Economic conditions<br>Social ties<br>Criminal background and skills	Italy	Incarcerated individuals and especially youngsters attracted by the cult of violence can join the mafias
**Brancaccio (2017)**	Mafias	Investigative files<br>Judicial records	Employment<br>Economic conditions<br>Social ties<br>Criminal background and skills	Italy	Individuals enter the mafias because of kinship and blood ties, coupled with low economic conditions and violent/risk taking behaviour
**Brotherton and Barrios (2004)**	Gangs	Interviews<br>Documents	Economic conditions<br>Group identity	U.S.	While females join gangs through blood ties, males rely on kinship and social relations developed in prison

Source	Type of OCG	Data collection	Categories of factors	Geographic scope	Main findings
**Ciconte (1992)**	Mafias	Judicial records<br>Documents	Age<br>Ethnicity<br>Educational background<br>Employment<br>Economic conditions<br>Social ties<br>Criminal background and skills<br>Silence/*Omertà*	Italy	Kinship and blood ties and the lack of legitimate occupations facilitate the involvement into the mafias
**Cressey (1969)**	Mafias	Interviews<br>Investigative files<br>Judicial records	Age<br>Ethnicity<br>Social ties<br>Group identity<br>Criminal background and skills<br>Silence/*Omertà*	U.S.	Values like honour, loyalty, and silence are crucial for individuals aiming at joining the mafias
**Decker and Chapman (2008)**	DTOs	Interviews	Ethnicity<br>Economic conditions<br>Social ties<br>Criminal background and skills	U.S.<br>Latin America	Ethnic ties have a crucial role in DTOs membership
**Densley (2012)**	Gangs	Interviews	Group identity<br>Criminal background and skills	U.K.	Individuals are recruited into gangs based on personal features like criminal competency and group loyalty
**Gambetta (1996)**	Mafias	Interviews<br>Judicial records	Employment<br>Social ties<br>Group identity<br>Criminal background and skills<br>Silence/*Omertà*	Italy	Being able to enforce the code of silence and possessing relevant expertise are crucial factors for being recruited into the mafias
**García (2006)**	DTOs	Tapes and CDs	Group identity	Mexico	Individuals get involved in DTOs because of their need of power, belonging, respect, security, and pride
**Gordon (2000)**	Gangs	Interviews	Ethnicity<br>Economic conditions<br>Social ties	Canada	Individuals often access gangs through close friends due to ethnic marginality and the attraction of supportive peer groups

Source	Type of OCG	Data collection	Categories of factors	Geographic scope	Main findings
**Hess (1993)**	Mafias	Judicial records<br>Documents	Educational background<br>Economic conditions<br>Social ties<br>Group identity<br>Criminal background and skills<br>Silence/*Omertà*	Italy	Individuals get involved in the mafias through social relations, especially because of their ability to connect the criminal members with other third parties, like other criminal groups or representative of legal businesses
**Hixon (2010)**	Gangs	Interviews	Gender<br>Group identity<br>Psychological factors	U.S.	Individuals recruited into OCGs usually have an history of negative and arrested development resulting in antisocial personality disorders
**Lo (2010)**	Mafias	Documents	Social ties<br>Group identity<br>Criminal background and skills<br>Silence/*Omertà*	China	Being able to connect people from different environments favour recruitment into Chinese Triads
**Lupo (1993)**	Mafias	Judicial records<br>Documents	Ethnicity<br>Social ties<br>Criminal background and skills<br>Silence/*Omertà*	Italy	The mafias mainly recruit new members based on ethnicity and kinship and blood ties
**Paoli (2003)**	Mafias	Interviews<br>Documents	Social ties<br>Group identity<br>Silence/*Omertà*	Italy	The contract of fraternization among recruited mafia members extends the bonds of loyalty and obligation beyond the family ties, providing them with mutual support and trust
**Sales (2015)**	Mafias	Judicial records<br>Documents	Educational background<br>Employment<br>Economic conditions<br>Social ties<br>Criminal background and skills	Italy	Individuals capable of strategic use of violence are recruited into the mafias
**Sciarrone (2014)**	Mafias	Judicial records	Social ties	Italy	The mafias mainly recruit new members based blood and kinship ties which are reinforced through an initiation ceremony with specific rituals
**Sergi (2016)**	Other Criminal Organisations	Investigative files<br>Documents	Economic conditions<br>Social ties<br>Group identity<br>Psychological factors	Italy	Being part of an OCG results from cultural transmission and gradual learning, therefore kinship and blood ties have a crucial role in the recruitment of new members
**Van Koppen (2013)**	Other Criminal Organisations	Investigative files	Employment<br>Economic conditions<br>Social ties	Netherlands	Individuals engage in OC activities exploiting their skills and kinship and blood ties, as criminals prefer to unfold their activities with family members or close friends

Source	Type of OCG	Data collection	Categories of factors	Geographic scope	Main findings
**Van San and Sikkens (2017)**	DTOs	Interviews<br>Informants	Gender<br>Economic conditions<br>Social ties	Netherlands<br>Peru	Female smugglers join DTOs mainly through their personal networks, i.e. family ties, romantic relationships, and friendships
**Varese (2001)**	Mafias	Interviews<br>Investigative files<br>Judicial records<br>Documents	Social ties<br>Criminal background and skills	Russia	Individuals getting involved in the mafias are recruited from a pool of trusted aspirants with no previous connections with law-enforcement agents
**Varese (2006)**	Mafias	Documents	Social ties	Italy	The mafias kin-based system of recruitment facilitates transplantation of criminal activities in a new region: when an entire blood family migrates the criminal group automatically reconstitutes itself
**Varese (2011b)**	Mafias	Interviews<br>Judicial records<br>Documents	Ethnicity<br>Social ties<br>Criminal background and skills	Italy<br>Hungary<br>U.S.<br>China	When Russian mafia groups migrates, they recruit new local members based on their dependability and proven ability to use violence
**Zhang and Chin (2001)**	Other Criminal Organisations	Interviews	Ethnicity<br>Group identity	U.S.<br>China	Individuals enter Chinese Triads because of their commitment to making money and direct connections in the Chinese communities

Table 5 – Details on the included mixed-methods studies

Source	Type of OCG	Data collection	Categories of factors	Geographic scope	Main findings
**Kleemans and de Poot (2008)**	Other Criminal Organisations	Investigative files	Age<br>Employment<br>Social ties<br>Criminal background and skills	Netherlands	Social relations in leisure and work settings may provide opportunities for joining OCGs throughout individuals' lives
**Kleemans and Van de Bunt (2008)**	Other Criminal Organisations	Investigative files	Employment<br>Social ties	Netherlands	Social relations in leisure and work settings may provide opportunities for joining OCGs throughout individuals' lives
**Morselli (2003)**	Mafias	Investigative files<br>Documents	Social ties<br>Criminal background and skills	U.S.	Social relations and their management are crucial in the involvement and career in the mafias
**Salinas, Requena and de la Corte (2011)**	Other Criminal Organisations	Interviews<br>Survey	Age<br>Gender<br>Employment<br>Social ties<br>Criminal background and skills	Spain	Individuals join OCGs because their employment could favour illegal activities or to have an additional income to their salary
**Van Koppen et al. (2010)**	Other Criminal Organisations	Investigative files	Age<br>Criminal background and skills	Netherlands	Most of individuals get involved in OCGs when they are adults
**Van Koppen, de Poot, and Blokland (2010)**	Other Criminal Organisations	Investigative files	Criminal background and skills	Netherlands	Compared with general crime offenders, OC offenders more often have previous and serious judicial records
**Varese (2011a)**	Mafias	Investigative files	Gender<br>Ethnicity<br>Social ties	Italy	Women getting involved in the mafias have a more relevant role abroad than in their territory of origin
**Varese (2013)**	Mafias	Investigative files	Gender<br>Ethnicity<br>Social ties	Italy	Individuals getting involved in the mafias abroad mainly focus on economic investments and resource acquisition

3.3. Recruitment across types of Organised Crime Groups

MAFIAS

The literature argues that the recruitment into mafias mainly results from the conjunction of social and economic factors. In particular, it focuses on the role and function of the social relations that mafia members have both within and beyond their OCG, and the personal attitudes and criminal skills necessary for entering such organisations.

Studies report that most mafia members come from low socio-economic conditions and frequently have kinship or blood ties within the OCGs (Albini, 1971; Brancaccio, 2017; Ciconte, 1992; Hess, 1993; Lupo, 1993; Paoli, 2003). In disadvantaged areas, individuals perceive government and law as mechanism of exploitation, thus developing distrust of state authorities and of those who lie outside the bound of family and kinship ties (Albini, 1971). The primary role of kinship and blood ties characterises all the mafias, although some internal differences exist. In the 'Ndrangheta, blood and mafia families frequently overlap, and this aspect seems to have facilitated its expansion beyond traditional territories (Sciarrone, 2014; Varese, 2006). Conversely, other mafias try to minimise the number of blood relatives within each criminal family in favour of criminal attitudes and skills (Arlacchi, 1983; Behan, 1996; Gambetta, 1996). Recruitment indeed occurs when there is a need for someone with special expertise, which can be acquired through criminal experience or experience gained in legitimate settings (Arlacchi, 1983; Behan, 1996; Cressey, 1969; Gambetta, 1996; Hess, 1993). The mafias therefore also include individuals from the middle-class, who have studied and gained some professional expertise that the OCGs could benefit from (Arlacchi, 1983).

Rituals characterising the mafias aim at reinforcing kinship and blood ties (Albini, 1971; Brancaccio, 2017; Ciconte, 1992; Gambetta, 1996; Hess, 1993; Lupo, 1993; Paoli, 2003). The rituals descend from solemn liturgies, old masonic rituals, and ancient secret brotherhood oaths adapted to the needs of the mafias. Every new member taking the oath becomes a "man of honour" and accepts the "contract of fraternization" for life (Paoli, 2003). Increased cohesion among members ensures both secrecy and trust, which are strongly interrelated. Given that they recur to violence, the mafias resort to secrecy to avoid law enforcement repressive action. In turn, since they operate outside of state jurisdiction through secrecy, violence serves to manage illicit businesses and solve conflicts (Paoli, 2003). In this perspective, having a risk-taking and violent attitude, as well as their *omertà*, are necessary skills to join the mafias

(Albini, 1971; Arlacchi, 1983; Behan, 1996; Ciconte, 1992; Gambetta, 1996; Hess, 1993; Lupo, 1993).

The Italian grey literature is consistent with the literature on socio-economic factors leading to the recruitment into Italian mafias.[11] In particular, they mostly revolve around factors as criminal background and skills, age and economic conditions (Figure 10 and Figure 11). They repeatedly claim that the socio-economic underdevelopment of certain areas determines the conditions favouring the persistence and permeability of the mafia subculture, especially among younger generations (Commissione Parlamentare Antimafia, 2006, 2012, Direzione Investigativa Antimafia, 2010b, 2010a, 2013b, 2014b, 2014a, Direzione Nazionale Antimafia, 2002, 2005, 2006; Direzione Nazionale Antimafia e Antiterrorismo, 2016).

Drug trafficking organisations

The studies included by this systematic review highlight the relevance of social and economic factors leading to recruitment into DTOs.

Involvement in DTOs can be explained by monetary returns. Individuals from low-income societies are more likely to join DTOs than individuals with other economic backgrounds (Carvalho & Soares, 2016; Decker & Chapman, 2008; Unlu & Ekici, 2012; Van San & Sikkens, 2017; Wang, 2013). However, financial gains alone do not explain individuals' involvement in DTOs (Unlu & Ekici, 2012).

The literature often portrays DTOs as ethnically homogeneous groups whose membership is gained through ethnic and social ties (Decker & Chapman, 2008; Wang, 2013). DTOs seek to recruit individuals within the same ethnic group as the transnational nature of their criminal activities requires relations based on mutual trust and loyalty. For this reason, involvement into DTOs is often facilitated by kinship and blood ties. Most opportunities for recruitment into DTOs arise through social relations, especially for women, whose entrance in the drug business is positively associated with love relationships (Van San & Sikkens, 2017). Recruitment may also occur through work ties (Van San & Sikkens, 2017; Wang, 2013).

Gangs

The majority of the studies addressing the recruitment of individuals into gangs mainly include social and psychological factors.

---

[11] The included Italian grey literature does not report about any psychological factors facilitating the recruitment into the mafias.

As for social factors, young adults turn to gangs because the group provides a strong sense of belonging and loyalty (Brotherton & Barrios, 2004; Densley, 2012; May, 2009). Social cohesion is reinforced by membership attitudes, behaviors and members' interpersonal interactions operating to establish a group identity. Gang cohesiveness is therefore the result of the combination of group dynamics and criminal behaviours shaping gang members' criminal identity. Studying gang subculture, Hixon (2010, p. 169) identifies three stages of individuals' self-initiation: (i.) rite of separation, (ii.) rite of transition, (iii.) rite of incorporation. The rite of separation initiates the members into the gang, which becomes perceived as a family. The separation process also involves the abandonment of mainstream norms and clothes, and the acquisition of gang symbols and clothing items. Subsequently, the transition phase takes place and the initiate starts wearing the clothing also outside the gang context. The rite of incorporation finally occurs when already established gang members consider the initiate a suitable candidate for full membership. Gang norms and cultural expectations are shared by the initiate willing to adopt them. Gang participation, loyalty and commitment to the group increase over time.

As for psychological factors, individuals in this recruitment process are characterized by low-self-control, substance use disorders, and/or antisocial personality disorder. Engagement in violent behavior and history of negative development are also positively associated with higher risk of joining gangs (Hixon, 2010; May, 2009).

OTHER CRIMINAL ORGANISATIONS

This study identified a number of relevant publications addressing the factors leading to recruitment into 'other criminal organisations', a residual category including outlaw motorcycle gangs, and OCGs in Albania, China, Italy, the Netherlands, Spain, and the U.K. The majority of the reported findings relate to social and economic factors.

The literature on organised crime reports that individuals join criminal organisations both in their teenage years (Arsovska, 2015; Blokland et al., 2017; Kirby et al., 2016; Ostrosky et al., 2012) and during adulthood (Blokland et al., 2017; Kleemans & de Poot, 2008; Salinas & Regadera, 2016; Van Koppen, Poot, et al., 2010). The recruitment of youngsters takes place because of the attractive criminal lifestyle and youths' marginalisation coupled with limited economic opportunities (Arsovska, 2015; Ostrosky et al., 2012). Conversely, adults join criminal organisations through the social opportunity structure mechanism resulting from the skills and social ties they have acquired with age (Blokland et al., 2017; Kleemans & de Poot, 2008; Salinas & Regadera, 2016; Van Koppen, de Poot, & Blokland, 2010). Professional

expertise developed mainly in the licit world may be exploited to reach new economic power, security, and take advantages of illegal business opportunities. More specifically, individuals who are self-employed (e.g. artisans and retail merchants) can more easily perform illegal activities through an extended professional network and/or social ties with criminals (Kleemans & Van de Bunt, 2008; Van Koppen, 2013).

The included studies also reveal a positive relation between kinship and blood ties, and individuals' involvement into criminal organisations (Requena et al., 2014; Salinas et al., 2011; Van Koppen, 2013). Having family members, relatives, or partners already members of criminal organisations facilitates the recruitment into criminal groups, whose criminal activities rely on mutual trust relations.

Finally, the literature highlights that criminal organisations tend to recruit crime prone individuals, violent individuals, and individuals with a criminal background (Blokland et al., 2017; Kirby et al., 2016; Salinas et al., 2011; Van Koppen, de Poot, & Blokland, 2010). Compared with general offenders, OC offenders are more likely to have prior convictions and to have spent more time in prison as a result of their involvement into criminal organisations (Van Koppen, de Poot, & Blokland, 2010).

4. Discussion and conclusion

This systematic review synthetises empirical findings on the social, psychological, and economic factors relating to criminalisation and recruitment to OCGs. This section discusses the main findings, analysing the sources included and assessing the validity and generalisability of the results. It also addresses implications and suggestions for future research.

MAIN FINDINGS

Social and economic factors are frequent in the literature addressing recruitment into OCGs, with psychological factors playing a minor role. Research findings show that all factors are highly interrelated, although the interaction varies across studies and types of OCGs. Furthermore, some factors are similarly present across OCGs, while others are more relevant for some OCGs than for others.

The literature consistently argues the importance of violent attitudes and behaviour, criminal background, low economic status, and particular social relations. These factors promote individuals' involvement into OC, regardless of the types of OCG. First, violence characterises OCGs activities and is often used to reinforce one's status within the organisation. Second, people who turn to OC usually have a significant criminal history and/or a prison background resulting from their proneness to violent and risk-taking behaviour. Third, people living in poor and socially disorganised areas experience the lack of access to legitimate means to attain commonly accepted goals. These mechanisms affect individuals' satisfaction and success, promoting the search of illegal alternatives to overcome these difficulties. Fourth, social relations also play a crucial role in the involvement into OCGs. Social proximity and interaction with members of OCGs promote the participation in OC. This includes kinship and other blood ties, allowing to enhance mutual trust and loyalty.

Despite many similarities across OCGs, some differences also exist. The mafias often rely on kinship and blood ties with other members, and initiation rituals among affiliates to reinforce their group identity. Rituality and culture factor is essential also for individuals' joining gangs, as these groups give members a sense of belonging. Conversely, individuals' recruitment into DTOs is mostly driven by monetary returns. Finally, individuals enter other types of OCGs during adulthood as they develop specialised expertise and social ties with criminals only later in life.

SOURCES INCLUDED IN THE SYSTEMATIC REVIEW

Despite the initial hypothesis that only a small amount of research on OCGs would have addressed the topic of recruitment and even lesser would have gone beyond mere theoretical discussions, this systematic review has succeeded in retrieving relevant information from several sources. The review is comprehensive: the search of relevant literature was extensive, conducted in multiple languages and through several databases. It considered all studies related to the scope of this review in the broadest sense, with no limitations as to their year of publication or geographic scope. The fact that only 0.1% of the initial 48,731 studies has entered this review results from two stringent methodological choices: included studies must be empirical and must report on factors specifically relating to recruitment into OCGs (Figure 2).

The 47 studies included in the review adopt quantitative, qualitative and mixed-methods approaches in investigating OC membership (Figure 4). The scarcity of quantitative data on the social, psychological, and economic factors relating to recruitment have led to the predominance of qualitative studies. Among the studies adopting a qualitative approach, many are monographies (Figure 3). These sources would not normally enter a systematic review as they are rarely indexed in databases, they seldom include relevant keywords in their titles, and usually lack abstracts. Nevertheless, the research team was able to identify, screen, and include them through experts' contribution.

The literature on OC has only lately embraced quantitative-oriented approaches. This also emerges from this systematic review when looking at mixed-methods and quantitative included studies. The studies adopting a mixed-methods approach (n=8) used judicial data coupled with interviews to analyse recruitment into the mafias and other criminal organisations. Conversely, quantitative studies (n=12) have mostly focused on DTOs, gangs, and other criminal organisations. All these studies were published over the last decade (2009-2017) and mainly relied on judicial data and investigative files. The recent shift towards a quantitative approach may have been favoured by the systematic collection of data related to OC and stored in structured databases, scholars' access to restricted or confidential information on OC offenders, and growing interest of governmental institutions to understand recruitment into OCGs. Regardless of the causes, this development is particularly interesting, as it will likely bring new insights into the field of OC research in the coming years.

VALIDITY AND GENERALISABILITY OF RESEARCH FINDINGS

The variety of methodological approaches adopted by the included studies guarantees the inclusion of all factors on recruitment into OCGs. The quantitative, qualitative, and mixed-methods studies are indeed consistent in

pointing at the same factors. Each approach has advantages or disadvantages for the analysis of specific factors. For instance, while quantitative studies usually focus on age, gender, and economic conditions, qualitative designs shed light on group identity and silence/*omertà*, or other factors that are more difficult to quantify (Figure 8).

The consistency of the results was further corroborated by relevant grey literature related to this field of study. Specifically, reports issued by Italian governmental bodies, legal authorities and law enforcement agencies to contrast the mafias across the country identify the same factors emerging from the review. These factors are not only emerging from empirical studies on the Italian mafias operating in their territory of origin, but also include studies on foreign mafias operating in other geographic areas.

However, the heterogeneity of the concept of OCG, as well as research designs and the data sources hinder the generalisability of findings beyond the OCG and the geographic area under analysis in each study. On the one hand, there is no unique definition of OC and the literature in this field of study encompasses disparate regions and criminal organisations. As discussed, this review has favoured a broad definition allowing flexibility in the selection of the studies. This enabled a comprehensive overview of the factors relating to recruitment into mafias, DTOs, gangs, and other criminal organisations. On the other hand, included studies suffer from limitations regarding data and methods they use. Many of the qualitative studies included adopt a case study design, which limits external generalisability of findings (Table 4). Among them, there are several monographies, which predominantly address the mafias in their territories of origin. Since the study of OC throughout the decades has focused on the Italian mafias, Italy is overrepresented when looking at the distribution of factors by geographic areas (Figure 7). The main limitation of quantitative and mixed-methods studies lies in their data sources. They mostly rely on secondary sources, mainly investigative and judicial data (Table 3 and Table 5). Data thus refer to non-random samples which can suffer from the "dark number" issue, i.e. the discrepancy between reality of events and the reported offences and identified offenders. Remarkably, particularly favourable research conditions in the Netherlands have led to an important number of studies focusing on this area. Scholars can rely on data coming from an ongoing research project supported by the Dutch Ministry of Justice (the Organized Crime Monitor).

META-ANALYSIS OF RESEARCH FINDINGS

The differences among included quantitative studies prevent the conduction of a meta-analysis. This methodology consists in creating a database with the reported results of selected studies to analyse them descriptively and inferentially to test certain hypotheses (Glass, 1976; Glass, Smith, & McGaw, 1981; Wolf, 1986). This approach allows to integrate, summarise, and review previous quantitative research on a specific issue, representing an "alternative to the casual, narrative discussions of research studies" (Glass, 1976, p. 3).

The research design of meta-analysis generally suits studies having similar populations, measuring techniques, definitions of variables and, above all, the same outcome variable (Glass et al., 1981). The 12 included quantitative studies clearly do not meet most of these requirements. Even though the variables in these studies fit into the framework of the factors for recruitment, they encompass, in fact, a variety of different concepts (e.g. the variables falling within the factor "criminal background and skills" include both a "having a violent attitude" and "having a prison background"). In one or two instances, up to four studies analyse comparable variables. However, they radically differ in the chosen dependent variable (e.g. some focus on the age of involvement into DTOs, others on gang membership). Overall, the populations and recruitment factors considered in these studies were too heterogeneous as to perform a meta-analysis.

FUTURE RESEARCH

In conclusion, this study highlights that there is a long-lasting interest in OC, though little attention has been paid to the factors relating to criminalisation and recruitment into OCGs. This systematic review has shed the light on social, psychological, and economic factors facilitating pathways into OC.

A number of implications emerge for future research. The first of them is the need for more quantitative studies. Only recently a limited set of studies has relied on quantitative data to define and measure the recruitment of individuals into OCGs. This weakness makes it difficult to identify the factors facilitating the involvement in OC and compare them across different types of OCGs and regions. The exchange of information between law enforcement agencies, scholars, and policy-makers may foster quantitative research in the study of recruitment into OCGs this field. The increased availability of official and judicial data would favour the proliferation of the recently emerged quantitative approach in this field of study. Compared to qualitative research, quantitative investigations may increase the external validity of the results, and facilitate replications of the same analyses adopting different scopes. The proliferation of quantitative studies would also allow for meta-analysis of research findings.

Secondly, results suggest that research should focus on factors relating to recruitment into OCGs with more detail. Many empirical studies only briefly touched upon the factors that this systematic review has found to be crucial. For instance, individuals' criminal background and social relations (i.e. work and family ties) with OC offenders are among the most important factors for individuals' involvement into OCGs. Some other factors as psychological features (e.g. personality traits) were underreported in the literature. Future studies should include them in their analyses to provide a more comprehensive outlook on recruitment in OC. Thirdly, current research has concentrated only on certain geographical areas. Up to date, there is scarce knowledge about recruitment processes in particular regions of the world, although OC appears to be present worldwide. Studies may therefore focus on underrepresented regions in the current OC literature (e.g. Asian or African countries).

These improvements would positively affect not only future systematic reviews, but also the policy making processes. Indeed, the results coming from quantitative studies may provide institutions with analyses useful to better understand the nature of recruitment into OCGs. A systematic analysis of the most recurrent factors relating to recruitment in OC would allow for the identification of individual red flags and risky environments (both a geographical and social level). The analysis of specific factors and regions would facilitate in designing tailored policies.

5. References

STUDIES INCLUDED IN THE SYSTEMATIC REVIEW

Albini, J. L. (1971). *The American mafia: Genesis of a legend*. New York: Appleton-Century-Crofts.

Arlacchi, P. (1983). *La mafia imprenditrice*. Bologna: Il Mulino.

Arsovska, J. (2015). *Decoding Albanian organized crime: Culture, politics, and globalization*. Oakland: University of California Press.

Behan, T. (1996). *The Camorra*. Routledge.

Blokland, A., van Hout, L., van der Leest, W., & Soudijn, M. (2017). Not your average biker; criminal careers of members of Dutch outlaw motorcycle gangs. *Trends in Organized Crime*. https://doi.org/10.1007/s12117-017-9303-x

Brancaccio, L. (2017). *I clan di camorra: genesi e storia*. Donzelli Editore.

Brotherton, D., & Barrios, L. (2004). *The Almighty Latin King and Queen Nation: Street Politics and the Transformation of a New York City Gang*. Columbia University Press.

Carvalho, L. S., & Soares, R. R. (2016). Living on the edge: Youth entry, career and exit in drug-selling gangs. *Journal of Economic Behavior & Organization*, *121*, 77–98. https://doi.org/10.1016/j.jebo.2015.10.018

Ciconte, E. (1992). *"Ndrangheta dall"unità a oggi*. Bari: Editori Laterza.

Cressey, D. R. (1969). *Theft of the Nation: The Structure and Operations of Organized Crime in America*. Transaction Publishers.

Decker, S. H., & Chapman, M. T. (2008). Roles, Recruitment into, and Remaining Involved in the Drug Smuggling Trade. In *Drug Smugglers on Drug Smuggling: Lessons from the Inside* (pp. 88–113). Philadelphia: Temple University Press.

Densley, J. A. (2012). Street Gang Recruitment: Signaling, Screening, and Selection. *Social Problems*, *59*(3), 301–321. https://doi.org/10.1525/sp.2012.59.3.301

Gambetta, D. (1996). *The Sicilian Mafia: The Business of Private Protection*. Harvard University Press.

García, M. M. (2006). "Narcoballads": The Psychology and Recruitment Process of the "Narco." *Global Crime*, *7*(2), 200–213. https://doi.org/10.1080/17440570601014461

Gordon, R. M. (2000). Criminal Business Organizations, Street Gangs and Wanna-be Groups: A Vancouver Perspective. *Canadian Journal of Criminology*, *42*, 39.

Hess, H. (1993). *Mafia: Le origini e la struttura*. Roma-Bari: Editori Laterza.

Hixon, S. J. (2010). *Archetypal perspectives on Nordic and Germanic initiation symbols, mythology, and rites of passage in a European American self-referenced White supremacist gang* (PhD dissertation, Saybrook University). Ann Arbor: ProQuest/UMI (Publication No. 3418929).

Jhi, K. Y., & Gerber, J. (2015). Texan Gangs In "Da Hood: The Impact of Actual and Perceptual Neighborhood Qualities on Gang Membership. *Justice Policy Journal*, *12*.

Kirby, S., Francis, B., Humphreys, L., & Soothill, K. (2016). Using the UK general offender database as a means to measure and analyse organized crime. *Policing: An International Journal*, *39*(1), 78–94. https://doi.org/10.1108/PIJPSM-03-2015-0024

Kissner, J., & Pyrooz, D. C. (2009). Self-control, differential association, and gang membership: A theoretical and empirical extension of the literature. *Journal of Criminal Justice*, *37*(5), 478–487. https://doi.org/10.1016/j.jcrimjus.2009.07.008

Kleemans, E. R., & de Poot, C. J. (2008). Criminal Careers in Organized Crime and Social Opportunity Structure. *European Journal of Criminology*, *5*(1), 69–98. https://doi.org/10.1177/1477370807084225

Kleemans, E. R., & Van de Bunt, H. G. (2008). Organised crime, occupations and opportunity. *Global Crime*, *9*(3), 185–197. https://doi.org/10.1080/17440570802254254

Lo, W. T. (2010). Beyond Social Capital: Triad Organized Crime in Hong Kong and China. *British Journal of Criminology*, *50*(5), 851–872. https://doi.org/10.1093/bjc/azq022

Lupo, S. (1993). *Storia della mafia*. Roma: Donzelli Editore.

May, C. (2009). *The relationship between risk factors, social support and gangs* (PhD dissertation, Alliant International University). Ann Arbor: ProQuest/UMI (Publication No. 3421105).

Morselli, C. (2003). Career opportunities and network-based privileges in the Cosa Nostra. *Crime, Law and Social Change*, *39*(4), 383–418. https://doi.org/10.1023/A:1024020609694

Ostrosky, F., Borja, K. C., Rebollar, C. R., & Díaz, K. X. (2012). Neuropsychological profiles of members of organized crime and drug-traffic organizations. *Research and Reports in Forensic Medical Science*, 19–30. https://doi.org/10.2147/RRFMS.S32352

Paoli, L. (2003). *Mafia Brotherhoods: Organized Crime, Italian Style*. Oxford University Press.

Requena, L., de Juan, M., Salinas, A. G., & de la Corte, L. (2014). A psychosocial study on crime and gender: Position, role and status of women in a sample of Spanish criminal organizations / Un estudio psicosocial sobre la delincuencia y género. Posición, rol y estatus de la mujer en una muestra española de organizaciones criminales. *Revista de Psicología Social*, *29*(1), 121–149. https://doi.org/10.1080/02134748.2013.878572

Sales, I. (2015). *Storia dell'Italia mafiosa: perché le mafie hanno avuto successo*. Rubbettino.

Salinas, A. G., & Regadera, S. F. (2016). Multiple affiliations in criminal organizations: analysis of a Spanish sample. *Crime, Law and Social Change*, *65*(1–2), 47–65. https://doi.org/10.1007/s10611-015-9597-z

Salinas, A. G., Requena, L., & De la Corte, L. (2011). ¿Existe un perfil de delincuente organizado? Exploración a partir de una muestra española. *Revista Electrónica de Ciencia Penal Y Criminología*, *13*.

Schimmenti, A., Caprì, C., La Barbera, D., & Caretti, V. (2014). Mafia and psychopathy: Mafia and psychopathy. *Criminal Behaviour and Mental Health*, *24*(5), 321–331. https://doi.org/10.1002/cbm.1902

Sciarrone, R. (2014). 'Ndrangheta: A Reticular Organization. In N. Serenata (Ed.), *The 'Ndrangheta and Sacra Corona Unita* (Vol. 12, pp. 81–99). Springer, Cham. https://doi.org/10.1007/978-3-319-04930-4_6

Sergi, A. (2016). A qualitative reading of the ecological (dis)organisation of criminal associations. The case of the "Famiglia Basilischi" in Italy. *Trends in Organized Crime*, *19*(2), 149–174. https://doi.org/10.1007/s12117-015-9254-z

Unlu, A., & Ekici, B. (2012). The extent to which demographic characteristics determine international drug couriers' profiles: a cross-sectional study in Istanbul. *Trends in Organized Crime*, *15*(4), 296–312. https://doi.org/10.1007/s12117-012-9152-6

Van Koppen, M. V. (2013). Involvement mechanisms for organized crime. *Crime, Law and Social Change*, *59*(1), 1–20. https://doi.org/10.1007/s10611-012-9396-8

Van Koppen, M. V., de Poot, C. J., & Blokland, A. A. J. (2010). Comparing Criminal Careers of Organized Crime Offenders and General Offenders. *European Journal of Criminology*, *7*(5), 356–374. https://doi.org/10.1177/1477370810373730

Van Koppen, M. V., de Poot, C. J., Kleemans, E. R., & Nieuwbeerta, P. (2010). Criminal Trajectories in Organized Crime. *British Journal of Criminology*, *50*(1), 102–123. https://doi.org/10.1093/bjc/azp067

Van San, M., & Sikkens, E. (2017). Families, Lovers, and Friends: Women, Social Networks, and Transnational Cocaine Smuggling from Curaçao and Peru. *The Howard Journal of Crime and Justice*, *56*(3), 343–357. https://doi.org/10.1111/hojo.12218

Varese, F. (2001). *The Russian Mafia: Private Protection in a New Market Economy*. OUP Oxford.

Varese, F. (2006). How Mafias Migrate: The Case of the `Ndrangheta in Northern Italy. *Law & Society Review*, *40*(2), 411–444. https://doi.org/10.1111/j.1540-5893.2006.00260.x

Varese, F. (2011a). How Mafias Take Advantage of Globalization: The Russian Mafia in Italy. *British Journal of Criminology*, *52*(2), 235–253. https://doi.org/10.1093/bjc/azr077

Varese, F. (2011b). *Mafias on the Move: How Organized Crime Conquers New Territories*. Princeton University Press.

Varese, F. (2013). The Structure and the Content of Criminal Connections: The Russian Mafia in Italy. *European Sociological Review*, *29*(5), 899–909. https://doi.org/10.1093/esr/jcs067

Wang, W. (2013). *Conventional capital, criminal capital, and criminal careers in drug trafficking* (PhD dissertation, Arts and Social Sciences: School of Criminology). Burnaby, B.C.: Simon Fraser University Library.

Zhang, S., & Chin, K.-L. (2002). Enter the Dragon: Inside Chinese Human Smuggling Organizations. *Criminology*, *40*(4), 737–768. https://doi.org/10.1111/j.1745-9125.2002.tb00972.x

ITALIAN GREY LITERATURE

Commissione Parlamentare Antimafia. (1991). *Commissione Parlamentare d'Inchiesta sul Fenomeno della Mafia e sulle altre Associazioni Criminali Similari, Doc. XXIII, n. 34, X legislatura*. Rome.

Commissione Parlamentare Antimafia. (1994). *Commissione Parlamentare d'Inchiesta sul Fenomeno della Mafia e sulle altre Associazioni Criminali Similari, Doc. XXIII, n. 14, XI legislatura*. Rome.

Commissione Parlamentare Antimafia. (2003). *Commissione Parlamentare d'Inchiesta sul Fenomeno della Criminalità Organizzata Mafiosa o Similare, Doc. XXIII, n. 3, XIV legislatura*. Rome.

Commissione Parlamentare Antimafia. (2006). *Commissione Parlamentare d'Inchiesta sul Fenomeno della Criminalità Organizzata Mafiosa o Similare, Doc. XXIII, n 16, Tomo II, XIV legislatura*. Rome.

Commissione Parlamentare Antimafia. (2012). *Commissione Parlamentare d'Inchiesta sul Fenomeno della Mafia e sulle altre Associazioni Criminali, anche Straniere, Doc. XXIII, n. 9, XVI legislatura*. Rome.

Direzione Investigativa Antimafia. (2000a). *Relazione del Ministro dell'Interno al Parlamento sull'attività svolta e sui risultati conseguiti dalla Direzione Investigativa Antimafia. 1° Semestre 2000*. Rome.

Direzione Investigativa Antimafia. (2000b). *Relazione del Ministro dell'Interno al Parlamento sull'attività svolta e sui risultati conseguiti dalla Direzione Investigativa Antimafia. 2° Semestre 2000*. Rome.

Direzione Investigativa Antimafia. (2001). *Relazione del Ministro dell'Interno al Parlamento sull'attività svolta e sui risultati conseguiti dalla Direzione Investigativa Antimafia. 2° Semestre 2001*. Rome.

Direzione Investigativa Antimafia. (2003). *Relazione del Ministro dell'Interno al Parlamento sull'attività svolta e sui risultati conseguiti dalla Direzione Investigativa Antimafia. 1° Semestre 2003*. Rome.

Direzione Investigativa Antimafia. (2006). *Relazione del Ministro dell'Interno al Parlamento sull'attività svolta e sui risultati conseguiti dalla Direzione Investigativa Antimafia. 2° Semestre 2006*. Rome.

Direzione Investigativa Antimafia. (2007). *Relazione del Ministro dell'Interno al Parlamento sull'attività svolta e sui risultati conseguiti dalla Direzione Investigativa Antimafia. 1° Semestre 2007*. Rome.

Direzione Investigativa Antimafia. (2008). *Relazione del Ministro dell'Interno al Parlamento sull'attività svolta e sui risultati conseguiti dalla Direzione Investigativa Antimafia. 2° Semestre 2008*. Rome.

Direzione Investigativa Antimafia. (2009). *Relazione del Ministro dell'Interno al Parlamento sull'attività svolta e sui risultati conseguiti dalla Direzione Investigativa Antimafia. 1° Semestre 2009*. Rome.

Direzione Investigativa Antimafia. (2010a). *Relazione del Ministro dell'Interno al Parlamento sull'attività svolta e sui risultati conseguiti dalla Direzione Investigativa Antimafia. 1° Semestre 2010*. Rome.

Direzione Investigativa Antimafia. (2010b). *Relazione del Ministro dell'Interno al Parlamento sull'attività svolta e sui risultati conseguiti dalla Direzione Investigativa Antimafia. 2° Semestre 2010*. Rome.

Direzione Investigativa Antimafia. (2011). *Relazione del Ministro dell'Interno al Parlamento sull'attività svolta e sui risultati conseguiti dalla Direzione Investigativa Antimafia. 1° Semestre 2011*. Rome.

Direzione Investigativa Antimafia. (2012a). *Relazione del Ministro dell'Interno al Parlamento sull'attività svolta e sui risultati conseguiti dalla Direzione Investigativa Antimafia. 1° Semestre 2012*. Rome.

Direzione Investigativa Antimafia. (2012b). *Relazione del Ministro dell'Interno al Parlamento sull'attività svolta e sui risultati conseguiti dalla Direzione Investigativa Antimafia. 2° Semestre 2012*. Rome.

Direzione Investigativa Antimafia. (2013a). *Relazione del Ministro dell'Interno al Parlamento sull'attività svolta e sui risultati conseguiti dalla Direzione Investigativa Antimafia. 1° Semestre 2013*. Rome.

Direzione Investigativa Antimafia. (2013b). *Relazione del Ministro dell'Interno al Parlamento sull'attività svolta e sui risultati conseguiti dalla Direzione Investigativa Antimafia. 2° Semestre 2013*. Rome.

Direzione Investigativa Antimafia. (2014a). *Relazione del Ministro dell'Interno al Parlamento sull'attività svolta e sui risultati conseguiti dalla Direzione Investigativa Antimafia. 1° Semestre 2014*. Rome.

Direzione Investigativa Antimafia. (2014b). *Relazione del Ministro dell'Interno al Parlamento sull'attività svolta e sui risultati conseguiti dalla Direzione Investigativa Antimafia. 2° Semestre 2014*. Rome.

Direzione Investigativa Antimafia. (2015a). *Relazione del Ministro dell'Interno al Parlamento sull'attività svolta e sui risultati conseguiti dalla Direzione Investigativa Antimafia. 1° Semestre 2015*. Rome.

Direzione Investigativa Antimafia. (2015b). *Relazione del Ministro dell'Interno al Parlamento sull'attività svolta e sui risultati conseguiti dalla Direzione Investigativa Antimafia. 2° Semestre 2015*. Rome.

Direzione Investigativa Antimafia. (2016a). *Relazione del Ministro dell'Interno al Parlamento sull'attività svolta e sui risultati conseguiti dalla Direzione Investigativa Antimafia. 1° Semestre 2016*. Rome.

Direzione Investigativa Antimafia. (2016b). *Relazione del Ministro dell'Interno al Parlamento sull'attività svolta e sui risultati conseguiti dalla Direzione Investigativa Antimafia. 2° Semestre 2016*. Rome.

Direzione Nazionale Antimafia. (2000). *Relazione annuale sulle attività svolte dal Procuratore nazionale antimafia e dalla Direzione nazionale antimafia nonché sulle dinamiche e strategie della criminalità organizzata di tipo mafioso nel periodo 1° Luglio 1999 – 30 Giugno 2000*. Rome.

Direzione Nazionale Antimafia. (2002). *Relazione annuale sulle attività svolte dal Procuratore nazionale antimafia e dalla Direzione nazionale antimafia nonché sulle dinamiche e strategie della criminalità organizzata di tipo mafioso nel periodo 1° Luglio 2001 – 30 Giugno 2002*. Rome.

Direzione Nazionale Antimafia. (2005). *Relazione annuale sulle attività svolte dal Procuratore nazionale antimafia e dalla Direzione nazionale antimafia nonché sulle dinamiche e strategie della criminalità organizzata di tipo mafioso nel periodo 1° Luglio 2004 – 30 Giugno 2005*. Rome.

Direzione Nazionale Antimafia. (2006). *Relazione annuale sulle attività svolte dal Procuratore nazionale antimafia e dalla Direzione nazionale antimafia nonché sulle dinamiche e strategie della criminalità organizzata di tipo mafioso nel periodo 1° Luglio 2005 – 30 Giugno 2006*. Rome.

Direzione Nazionale Antimafia. (2014). *Relazione annuale sulle attività svolte dal Procuratore nazionale antimafia e dalla Direzione nazionale antimafia nonché sulle dinamiche e strategie della criminalità organizzata di tipo mafioso nel periodo 1° Luglio 2012 – 30 Giugno 2013*. Rome.

Direzione Nazionale Antimafia. (2015). *Relazione annuale sulle attività svolte dal Procuratore nazionale antimafia e dalla Direzione nazionale antimafia nonché sulle dinamiche e strategie della criminalità organizzata di tipo mafioso nel periodo 1° Luglio 2013 – 30 Giugno 2014*. Rome.

Direzione Nazionale Antimafia e Antiterrorismo. (2016). *Relazione annuale sulle attività svolte dal Procuratore nazionale e dalla Direzione nazionale antimafia e antiterrorismo nonché sulle dinamiche e strategie della criminalità organizzata di tipo mafioso nel periodo 1° Luglio 2014 – 30 Giugno 2015*. Rome.

Other references

Abadinsky, H. (1981). *The Mafia in America: an oral history*. New York: Praeger.

Adamoli, S., Di Nicola, A., Savona, E. U., & Zoffi, P. (1998). *Organised Crime Around the World*. European Institute for Crime Prevention and Control. Retrieved from http://www.heuni.fi/material/attachments/heuni/reports/6KdD32kXX/Hreport_31.pdf

Adler, P. A. (1993). *Wheeling and Dealing: An Ethnography of an Upper-Level Drug Dealing and Smuggling Community, second edition*. New York: Columbia University Press.

Agnew, R. (1992). Foundation for a General Strain Theory. *Criminology*, *30*(1), 47–87.

Agnew, R. (2006). *Pressured into crime: An overview of general strain theory*. Los Angeles: Roxbury.

Albanese, J. S. (1985). *Organized crime in America*. Cincinnati: Anderson Pub. Co.

Albini, J. L. (1971). *The American mafia: Genesis of a legend*. New York: Appleton-Century-Crofts.

Astorga, L., & Shirk, D. A. (2010). Drug Trafficking Organizations and Counter-Drug Strategies in the U.S.-Mexican Context. *Center for US-Mexican Studies*.

Bagley, B. M. (2004). Globalisation and Latin American and Caribbean Organised Crime. *Global Crime*, *6*(1), 32–53. https://doi.org/10.1080/1744057042000297963

Bagley, B. M., & Rosen, J. D. (2015). *Drug trafficking, organized crime, and violence in the Americas today*. Gainesville: University press of Florida.

Bandiera, O. (2003). Land Reform, the Market for Protection, and the Origins of the Sicilian Mafia: Theory and Evidence. *Journal of Law, Economics, and Organization*, *19*(1), 218–244. https://doi.org/10.1093/jleo/19.1.218

Beittel, J. S. (2012). Mexico’s drug trafficking organizations: Source and scope of the rising violence. In W. Chambers & R. Hale (Eds.), *Mexico’s Drug Trafficking Violence: Scope, Consequences and Response* (pp. 1–46). Hauppauge: Nova Science Publishers, Inc.

Benson, J. S., & Decker, S. H. (2010). The organizational structure of international drug smuggling. *Journal of Criminal Justice*, *38*(2), 130–138.

Benzies, K. M., Premji, S., Hayden, K. A., & Serrett, K. (2006). State-of-the-Evidence Reviews: Advantages and Challenges of Including Grey Literature. *Worldviews on Evidence-Based Nursing*, *3*(2), 55–61. https://doi.org/10.1111/j.1741-6787.2006.00051.x

Blevins, K. R., Johnson Listwan, S., Cullen, F. T., & Lero Jonson, C. (2010). A General Strain Theory of Prison Violence and Misconduct: An Integrated Model of Inmate Behavior. *Journal of Contemporary Criminal Justice*, *26*(2), 148–166. https://doi.org/10.1177/1043986209359369

Block, A. A., & Scarpitti, F. R. (1985). *Poisoning for Profit: The Mafia and Toxic Waste in America*. New York: William Morrow & Company.

Bunker, R. J. (2015). Cameron H. Holmes: Organized crime in Mexico: Assessing the threat to North American economies. *Trends in Organized Crime*, *18*(4), 326–328. https://doi.org/10.1007/s12117-015-9240-5

Calderoni, F. (2012). Definition That Does Not Work: The Impact of the EU Framework Decision on the Fight against Organized Crime, A. *Common Market Law Review*, *49*, 1365–1394.

Calderoni, F., Berlusconi, G., Garofalo, L., Giommoni, L., & Sarno, F. (2015). The Italian mafias in the world: A systematic assessment of the mobility of criminal groups. *European Journal of Criminology*, *13*(4), 413–433. https://doi.org/10.1177/1477370815623570

Campana, P. (2013). Understanding Then Responding to Italian Organized Crime Operations across Territories. *Policing*, *7*(3), 316–325.

Carvalho, L. S., & Soares, R. R. (2016). Living on the edge: Youth entry, career and exit in drug-selling gangs. *Journal of Economic Behavior & Organization*, *121*, 77–98. https://doi.org/10.1016/j.jebo.2015.10.018

Chu, Y. K. (2000). *The Triads as Business*. London: Routledge.

Clapper, J. R. (2015). *Statement for the Record Worldwide Threat Assessment of the U.S. IC Before the Senate Armed Services Committee*. Washington: Office of the Director of National Intelligence. Retrieved from https://www.dni.gov/files/documents/Unclassified_2015_ATA_SFR_-_SASC_FINAL.pdf

Cloward, R., & Ohlin, L. (1960). *Delinquency and Opportunity*. New York: The Free Press.

Cohen, A. K. (1955). *Delinquent Boys: The Culture of the Gang*. Glencoe, IL: Free Press.

Cornish, D. B., & Clarke, R. V. (2002). Analyzing Organized Crime. In A. R. Piquero & S. G. Tibbetts, *Rational Choice and Criminal Behavior: Recent Research and Future Challenges*. New York: Garland.

Cressey, D. R. (1969). *Theft of the Nation: The Structure and Operations of Organized Crime in America*. New Brunswick and London: Transaction Publishers.

Critical Appraisal Skills Programme. (2017). *CASP Qualitative Checklist*. [online] Available at: http://www.casp-uk.net/checklists Accessed: 11/10/2017.

Curry, G. D. (2015). The Logic of Defining Gangs Revisited. In S. H. Decker & D. C. Pyrooz (Eds.), *The Handbook of Gangs* (pp. 7–27). Hoboken: John Wiley & Sons, Inc.

Daniele, V. (2009). Organized crime and regional development. A review of the Italian case. *Trends in Organized Crime*, *12*(3–4), 211. https://doi.org/10.1007/s12117-009-9079-8

de la Miyar, J. R. B. (2016). The economic consequences of the mexican drug war. *Peace Economics, Peace Science and Public Policy*, *22*(3), 213–246. https://doi.org/10.1515/peps-2016-0014

Decker, S. H., & Pyrooz, D. C. (2014). Gangs Another Form of Organized Crime? In L. Paoli (Ed.), *Oxford Handbook of Organized Crime*. Oxford: Oxford University Press.

Desroches, F. (2007). Research on upper level drug trafficking: a review. *Journal of Drug Issues*, *37*(4), 827–844.

Dorn, N., Levi, M., & King, L. (2005). *Literature review on upper level drug trafficking* (Home Office Online Report No. 22/05). London: Home Office Research, Development and Statistics Directorate.

Eck, J. E., & Gersh, J. S. (2000). Drug Trafficking as a Cottage Industry. In M. Natarajan & M. Hough (Eds.), *Illegal drug markets: from research to prevention policy* (pp. 241–271). Boulder: Lynne Rienner Publishers.

Edwards, A., & Gill, P. (2002). The Politics of "Transnational Organized Crime": Discourse, Reflexivity and the Narration of "Threat." *The British Journal of Politics and International Relations*, *4*(2), 245–270. https://doi.org/10.1111/1467-856X.t01-1-00004

Farrington, D. P. (2003). Developmental and Life-Course Criminology: Key Theoretical and Empirical Issues-the 2002 Sutherland Award Address. *Criminology*, *41*(2), 221–225. https://doi.org/10.1111/j.1745-9125.2003.tb00987.x

Fiandaca, G. (Ed.). (2007). *Women and the mafia: Female roles in organized crime structures* (Vol. 5). New York: Springer Science & Business Media.

Fijnaut, C., & Paoli, L. (2004a). Introduction to Part I: The History of the Concept. In C. Fijnaut & L. Paoli (Eds.), *Organised Crime in Europe: Concepts, Patterns and Control Policies in the European Union and beyond* (pp. 21–46). Dordrecht: Springer. https://doi.org/10.1007/978-1-4020-2765-9_1

Fijnaut, C., & Paoli, L. (2004b). *Organised Crime in Europe: Concepts, Patterns and Control Policies in the European Union and Beyond*. New York: Springer Science & Business Media.

Finckenauer, J. O. (2005). Problems of definition: What is organized crime? *Trends in Organized Crime*, *8*(3), 63–83. https://doi.org/10.1007/s12117-005-1038-4

Finckenauer, J. O. (2007). *Mafia and Organized Crime: A Beginner's Guide*. Oxford: Oneworld Publications.

Fleetwood, J. (2014). *Drug mules women in the international cocaine trade*. Basingstoke: Palgrave Macmillan.

Gambetta, D. (1996). *The Sicilian Mafia: The Business of Private Protection*. Harvard University Press.

Glass, G. V. (1976). Primary, secondary, and meta-analysis of research. *Educational Researcher*, *5*(10), 3–38.

Glass, G. V., Smith, M. L., & McGaw, B. (1981). *Meta-Analysis in Social Research*. Beverly Hills: SAGE Publications.

Hagan, F. E. (1983). The Organized Crime Continuum: A Further Specification of a New Conceptual Model. *Criminal Justice Review*, *8*(2), 52.

Hagan, F. E. (2006). "Organized crime" and "organized crime": Indeterminate problems of definition. *Trends in Organized Crime*, *9*(4), 127–137. https://doi.org/10.1007/s12117-006-1017-4

Hagan, F. E. (2015). *Introduction to Criminology: Theories, Methods, and Criminal Behavior*. Los Angeles: SAGE Publications.

Higginson, A., Mazerolle, L., Benier, K. H., & Bedford, L. (2014). Predictors of youth gang membership in low-and middle-income countries: A systematic review. *Campbell Systematic Reviews*. Retrieved from https://campbellcollaboration.org/library/predictors-of-youth-gang-membership-low-and-middle-income-countries.html

Hill, P. B. E. (2003). *The Japanese Mafia*. New York: Oxford University Press.

Ianni, F. A. J. (1974). *Black Mafia: Ethnic Succession in Organized Crime* (First Edition). New York: Simon & Schuster.

Ingrascì, O. (2007). *Donne d'onore: storie di mafia al femminile*. Milan: Pearson Italia S.p.a.

Jones, O. D., Wagner, A. D., Faigman, D. L., & Raichle, M. E. (2013). Neuroscientists in court. *Nature Reviews Neuroscience*, *14*(10), 730–736. https://doi.org/10.1038/nrn3585

Kaplan, D. E., & Dubro, A. (2003). *Yakuza: Japan's Criminal Underworld*. University of California Press.

Kelly, R. J. (1997). Political-Criminal Nexus: The United States. *Trends in Organized Crime*, *3*(1), 78–81. https://doi.org/10.1007/s12117-997-1147-3

Kenney, D. J., & Finckenauer, J. O. (1995). *Organized Crime in America*. Belmont: Wadsworth Pyb. Co.

Kleemans, E. R. (2014). Theoretical perspectives on organized crime. In L. Paoli (Ed.), *The Oxford Handbook of Organized Crime* (pp. 32–52). Oxford: Oxford University Press.

Kleemans, E. R., & De Poot, C. J. (2008). Criminal Careers in Organized Crime and Social Opportunity Structure. *European Journal of Criminology*, *5*(1), 69–98. https://doi.org/10.1177/1477370807084225

Kleemans, E. R., Kruisbergen, E. W., & Kouwenberg, R. F. (2014). Women, brokerage and transnational organized crime. Empirical results from the Dutch Organized Crime Monitor. *Trends in Organized Crime*, *17*(1–2), 16–30. https://doi.org/10.1007/s12117-013-9203-7

Kleemans, E. R., & Van de Bunt, H. G. (1999). The social embeddedness of organized crime. *Transnational Organized Crime, 5*(1), 19–36.

Kleemans, E. R., & Van Koppen, M. V. (2014). Careers in organized crime. In G. Bruinsma & D. Weisburd, *Encyclopedia of Criminology and Criminal Justice* (pp. 285–295). New York: Springer.

Klein, M. W., & Maxson, C. L. (2006). *Street Gang Patterns and Policies*. New York: Oxford University Press.

Knox, J., & Gray, D. H. (2014). The National and International Threat of Drug Trafficking Organizations. *Global Security Studies*, *5*(3), 27–34.

Langston, M. (2003). Addressing the Need for a Uniform Definition of Gang-Involved Crime. *FBI Law Enforcement Bulletin*, *72*, 7.

Lavezzi, A. M. (2008). Economic structure and vulnerability to organised crime: Evidence from Sicily. *Global Crime*, *9*(3), 198–220. https://doi.org/10.1080/17440570802254312

Lavezzi, A. M. (2014). Organised crime and the economy: a framework for policy prescriptions. *Global Crime*, *15*(1–2), 164–190. https://doi.org/10.1080/17440572.2013.868626

Leeper Piquero, N., & Sealock, M. D. (2010). Race, Crime, and General Strain Theory. *Youth Violence and Juvenile Justice*, *8*(3), 170–186. https://doi.org/10.1177/1541204009361174

Loeber, R., & Farrington, D. P. (2014). Age-crime curve. In Gerben Bruinsma & D. Weisburd (Eds.), *Encyclopedia of Criminology and Criminal Justice* (pp. 12–18). New York: Springer.

Lyman, M. D., & Potter, G. W. (2006). *Organized Crime* (4 edition). Upper Saddle River: Prentice Hall.

Mahood, Q., Van Eerd, D., & Irvin, E. (2014). Searching for grey literature for systematic reviews: challenges and benefits. *Research Synthesis Methods*, *5*(3), 221–234. https://doi.org/10.1002/jrsm.1106

Matza, D., & Sykes, G. M. (1961). Juvenile Delinquency and Subterranean Values. *American Sociological Review*, *26*(5), 712–719. https://doi.org/10.2307/2090200

McIllwain, J. S. (1999). Organized crime: A social network approach. *Crime, Law and Social Change*, *32*(4), 301–323.

Merton, R. K. (1938). Social structure and anomie. *American Sociological Review*, *3*, 572–682.

Miguel Cruz, J. (2010). Central American maras: from youth street gangs to transnational protection rackets. *Global Crime*, *11*(4), 379–398. https://doi.org/10.1080/17440572.2010.519518

Miller, W. B. (1958). Lower Class Culture as a Generating Milieu of Gang Delinquency. *Journal of Social Issues*, *14*(3), 5–19. https://doi.org/10.1111/j.1540-4560.1958.tb01413.x

Miró, R. J. (2003). *Organized Crime and Terrorist Activity in Mexico, 1999-2002*. Washington D.C.: Federal Research Division. Retrieved from https://www.loc.gov/rr/frd/pdf-files/OrgCrime_Mexico.pdf

Morselli, C. (2003). Career opportunities and network-based privileges in the Cosa Nostra. *Crime, Law and Social Change*, *39*(4), 383–418. https://doi.org/10.1023/A:1024020609694

Morselli, C. (2009). *Inside Criminal Networks*. New York, NY: Springer.

Morselli, C., & Tremblay, P. (2004). Criminal Achievement, Offender Networks and the Benefits of Low Self-Control. *Criminology*, *42*(3), 773–804.

Morselli, C., Turcotte, M., & Tenti, V. (2011). The mobility of criminal groups. *Global Crime*, *12*(3), 165–188. https://doi.org/10.1080/17440572.2011.589593

Natarajan, M., Zanella, M., & Yu, C. (2015). Classifying the Variety of Drug Trafficking Organizations. *Journal of Drug Issues*, *45*(4), 409–430. https://doi.org/10.1177/0022042615603391

Ostrosky, F., Borja, K. C., Rebollar, C. R., & Díaz, K. X. (2012). Neuropsychological profiles of members of organized crime and drug-traffic organizations. *Research and Reports in Forensic Medical Science*, 19–30. https://doi.org/10.2147/RRFMS.S32352

Paoli, L. (2003). *Mafia brotherhoods: organized crime, Italian style*. Oxford: Oxford University Press.

Paoli, L. (2014a). Organized Crime, Types of. In Gerben Bruinsma & D. Weisburd (Eds.), *Encyclopedia of Criminology and Criminal Justice* (pp. 3376–3387). New York: Springer.

Paoli, L. (2014b). The Italian Mafia. In L. Paoli (Ed.), *The Oxford Handbook of Organized Crime* (pp. 121–141). New York: Oxford University Press.

Passas, N. (1999). *Transnational Crime*. Farnham: Ashgate.

Pearson, G., & Hobbs, D. (2001). *Middle Market Drug Distribution* (Home Office Research Study No. 227). London: Home Office Research, Development and Statistics Directorate.

Pérez, D. M., Jennings, W. G., & Gover, A. R. (2008). Specifying General Strain Theory: An Ethnically Relevant Approach. *Deviant Behavior*, *29*(6), 544–578. https://doi.org/10.1080/01639620701839385

Petticrew, M., & Roberts, H. (Eds.). (2008). *Systematic Reviews in the Social Sciences*. Oxford: Blackwell Publishing Ltd. https://doi.org/10.1002/9780470754887

Reuter, P. H., & Haaga, J. (1989). The Organization of High-Level Drug Markets [Product Page]. Retrieved January 12, 2017, from http://www.rand.org/pubs/notes/N2830.html

Roser, M., & Ortiz-Ospina, E. (2017). Global Rise of Education. *Published online at Our World In Data*. Retrieved November 24, 2017, from https://ourworldindata.org/global-rise-of-education.

Sarno, F. (2014). Italian Mafias in Europe: Between Perception and Reality. A Comparison of Press Articles in Spain, Germany and the Netherlands. *Trends in Organized Crime*, 1–29.

Sciarrone, R., & Storti, L. (2014). The Territorial Expansion of Mafia-Type Organized Crime. The Case of the Italian Mafia in Germany. *Crime, Law and Social Change*, *61*(1), 37–60.

Sergi, A., & Lavorgna, A. (2016). *'Ndrangheta: The Glocal Dimensions of the Most Powerful Italian Mafia*. Cham: Springer International Publishing.

Shaw, C. R., & McKay, H. D. (1972). *Juvenile delinquency and urban areas*. Chicago: University of Chicago Press.

Siebert, R. (1996). *Secrets of Life and Death: Women and the Mafia*. London: Verso.

Symeonidou-Kastanidou, E. (2007). Towards a New Definition of Organised Crime in the European Union. *European Journal of Crime, Criminal Law and Criminal Justice*, *15*, 83.

Thrasher, F. M. (1927). *The gang: A study of 1,313 gangs in Chicago* (Abridged edition). University Of Chicago Press.

Transcrime. (2013). *Progetto PON Sicurezza 2007-2013. Gli investimenti delle mafie* (Rapporto finale del progetto "I beni sequestrati e confiscati alle organizzazioni criminali nelle regioni dell'Obiettivo Convergenza: dalle strategie di investimento della criminalità all'impiego di fondi comunitari nel riutilizzo dei beni già destinati"). Roma: Ministero dell'Interno.

United Nations. (2000). *Convention on Transnational Organized Crime* (No. Assembly Resolution 55/95 of 15 Nov 2000). Retrieved from http://www.unodc.org/unodc/treaties/CTOC/

UNODC. (2010). *The globalization of crime: a transnational organized crime threat assessment*. Wien. Retrieved from https://www.unodc.org/unodc/en/data-and-analysis/tocta-2010.html

U.S. Department of Justice. (2010). *National Drug Threat Assessment 2010* (National Drug Intelligence Center). Retrieved from https://www.justice.gov/archive/ndic/pubs38/38661/

Van Koppen, M. V., de Poot, C. J., & Blokland, A. A. J. (2010). Comparing Criminal Careers of Organized Crime Offenders and General Offenders. *European Journal of Criminology*, *7*(5), 356–374. https://doi.org/10.1177/1477370810373730

Van Koppen, M. V., Poot, C. J. de, Kleemans, E. R., & Nieuwbeerta, P. (2010). Criminal Trajectories in Organized Crime. *British Journal of Criminology*, *50*(1), 102–123. https://doi.org/10.1093/bjc/azp067

Varese, F. (2005). *The Russian Mafia*. New York: Oxford University Press.

Varese, F. (2006). How Mafias Migrate: The Case of the `Ndrangheta in Northern Italy. *Law & Society Review*, *40*(2), 411–444. https://doi.org/10.1111/j.1540-5893.2006.00260.x

Varese, F. (2010a). General Introduction: What is Organized Crime?". In F. Varese (Ed.), *Organized Crime* (pp. 1–35). Critical Concepts in Criminology. London; New York: Routledge.

Varese, F. (2010b). *Organized crime: critical concepts in criminology*. London; New York: Routledge.

Varese, F. (2011). How Mafias Take Advantage of Globalization: The Russian Mafia in Italy. *British Journal of Criminology*, *52*(2), 235–253. https://doi.org/10.1093/bjc/azr077

Vásquez, J. C. R. (2015). Crime and Punishment in Colombia. In *The Encyclopedia of Crime and Punishment*. Hoboken: John Wiley & Sons, Inc.

Von Lampe, K. (2008). Organized Crime in Europe: Conceptions and Realities. *Policing*, *2*(1), 7–17. https://doi.org/10.1093/police/pan015

Von Lampe, K. (2015). *Organized Crime: Analyzing Illegal Activities, Criminal Structures, and Extra-legal Governance* (1 edition). John Jay College of Criminal Justice, New York: SAGE Publications.

Von Lampe, K., Van Dijck, M., Hornsby, R., Markina, A., & Verpoest, K. (2006). Organised Crime Is.......Findings from a Cross-National Review of Literature." In. In P. C. Van Duyne, A. Maljevic, M. Van Dijck, K. Von Lampe, & J. L. Newell (Eds.), *The Organisation of Crime for Profit: Conduct, Law and Measurement* (pp. 17–41). Nijmegen: Wolf Legal Publishers.

Walsh, A., & Beaver, K. M. (2009). Biosocial Criminology. In *Handbook on Crime and Deviance*. New York: Springer.

Wolf, F. M. (1986). *Meta-analysis: Quantitative methods for research synthesis*. Beverly Hills: SAGE Publications.

Wood, J. L., Alleyne, E., Mozova, K., & James, M. (2014). Predicting involvement in prison gang activity: Street gang membership, social and psychological factors. *Law and Human Behavior, 38*(3), 203–211. https://doi.org/10.1037/lhb0000053

Woodiwiss, M. (2003). Transnational Organized Crime: The Strange Career of an American Concept. In M. E. Beare (Ed.), *Critical Reflections on Transnational Organized Crime, Money Laundering, and Corruption* (pp. 1–34). Toronto: University of Toronto Press. https://doi.org/10.3138/9781442670242-003

Methodological annex

Criteria for including studies in the review

Scope

This systematic review aims at identifying the social, psychological, and economic factors leading to recruitment into organised crime groups (OCGs). The focus is on empirical studies that provided evidence-based findings about the association between the social, psychological, and economic characteristics of a person and their membership in a criminal organisation.

Types of criminal organisations

The review considered four types of OCGs:[12]

- Mafias, i.e. the Italian mafias, the Russian mafia, the Yakuza, and the Triads;
- Drug trafficking organisations (DTOs), e.g. Mexican cartels;
- Gangs, e.g. street gangs;[13]
- Other criminal organisations, a residual category including for instance outlaw motorcycle gangs.

This classification allowed for the inclusion, and eventually a comparison, of a wide variety of criminal organisations.

Types of factors leading to recruitment into OCGs

Any social, psychological, or economic factors that are found to drive recruitment into OCGs were considered. The identification of relevant factors was a twofold process. Firstly, the research team reported all information related to recruitment emerging from each study. After the data collection, researchers created 11 categories comprising all factors stemming from the included studies. These categories are: age, gender, ethnicity, educational background, employment, economic conditions, social ties, group identity, psychological factors, criminal background and skills, and silence/*omertà*.

---

[12] See section 2.1 for more details on each OCG.

[13] Youth gang were beyond the scope of the review. They are not normally considered OCGs (Decker & Pyrooz, 2014). Also, a recent systematic review shedding light onto the factors leading to youth gang membership already exists (Higginson, Mazerolle, Benier, & Bedford, 2014).

TYPES OF OUTCOME MEASURES

The outcome of interest of this systematic review was membership in OCGs. The review included self-reported, peer-reported, practitioner-reported, or police-reported measures of individual OCG membership.

TYPES OF METHODOLOGICAL DESIGNS

The scarcity of quantitative literature on factors leading to membership in OCGs made it advisable to apply a broad methodological scope. The systematic review thus covered quantitative, qualitative, and mixed-methods studies.

To be included, quantitative studies needed to contain any assessment of the relationship between a social, psychological, or economic factor and OCG membership. Also, they needed to measure the outcome at individual level. Mixed-methods and qualitative studies, on the other hand, needed to discuss the effects of either individual or contextual factors on individual participation in OCGs.

Furthermore, to be considered, all studies needed to report on the sampling strategy, data collection, and the type of analysis that was conducted. They also needed to meet the quality criteria set out by an adapted version of the Critical Appraisal Skills Programme (CASP) for qualitative studies (Critical Appraisal Skills Programme, 2017).[14] This consisted of 10 questions:

1. Was there a clear statement of the aims of the research?
2. Is the methodology appropriate?
3. Was the research design appropriate to address the aims of the research?
4. Was the recruitment strategy appropriate to the aims of the research?
5. Was the data collected in a way that addressed the research issue?
6. Has the relationship between researcher and participants been adequately considered?
7. Have ethical issues been taken into consideration?
8. Was the data analysis sufficiently rigorous?
9. Is there a clear statement of findings?
10. How valuable is the research?

A study was rated as low-quality and thus excluded from the review if they do not meet criteria n.3 and 8.[15]

---

[14] The difference between the original Qualitative Research Checklist and the adapted version of the CASP is that specific reference to qualitative studies was erased to make the checklist applicable to mixed-methods and quantitative studies where appropriate.

[15] As reported in the section on Selection of studies, 4 studies were excluded during the full-text screening because they did not meet the CASP criteria. Specifically, they provided a

Exclusion criteria

The review excluded studies that were out of scope, e.g. when it was on youth gangs or adopted a macro-perspective on socio-economic factors that facilitate involvement with criminal groups. The review also excluded merely theoretical literature. The research team categorized these studies as “contextual” and “theoretical”.

Search methods

Search terms

The search terms fell into three main categories (Figure 1): A) types of organised crime groups; B) social, psychological, or economic factors; C) recruitment of individuals into OCGs. Table 6 shows the search terms subdivided into categories. The Boolean Operator “OR” connected keywords pertaining to the same category, while the Boolean Operator “AND” connected keywords from different categories. This query structure ensured to retrieve only those studies that contained at least one term from each word category.

---

quick overview of the factors relating to recruitment into OCGs, without performing rigorous analyses.

Table 6 – Search categories and related search terms

Category A	Category B	Category C
criminal organisation	disadvantag*	predictor*
criminal association	econom*	driver*
organized crime	salar*	determinant*
organised crime	inequalit*	involve*
mafia	povert*	starter*
gang*	occupation*	recruit*
criminal gang*	inequalit*	network*
crim* network*	migrant*	correlat*
dto*	immigration*	member*
drug trafficking organ*	social embeddedness	factor*
drug cartel*	school*	criminal career*
motorcycle gang*	environment*	criminal trajector*
criminal group*	scholar*	
crim* cartel	social integrat*	
Italian mafia	race*	
Russian mafia	racis*	
Japanese mafia	integr* soc*	
Chinese mafia	household*	
narco* boss*	demograph*	
	famil*	
	emargina*	
	behavioral	
	psych*	

Search locations and languages

The research team identified several scientific databases (n=12) and conducted the search for relevant studies in five different languages (i.e. English, French, German, Italian and Spanish) and, using search engines for various research disciplines (see Table 1). Both academically published and grey literature were considered. No limitations applied as to where the literature originated from geographically, or when it was published. The research team attended two meetings with a librarian to ensure that all relevant databases for the systematic review had been included and to validate the search terms and queries.

Table 1 reports the list of databases indicating in which language the search was conducted and which search technique was applied. When available, the preferred technique was to search title, abstract and keywords. Table 7 shows the list of databases and related queries used to perform the research.

Table 7 – Database and related queries

Database	Queries
EBSCO	AB (("criminal organisation" OR "criminal association" OR "organized crime" OR "organised crime" OR mafia OR gang* OR criminal gang* OR crim* network* OR DTO* OR drug trafficking organ* OR drug cartel* OR motorcycle gang* OR criminal group* OR crim* cartel OR "Italian Mafia" OR "Russian Mafia" OR "Japanese Mafia" OR "Chinese Mafia" OR narco* boss*)) AND AB ((disadvantag* OR econom* OR salar* OR inequalit* OR povert* OR occupation* OR inequalit* OR migrant* OR immigration* OR "social embeddedness" OR school* OR environment* OR scholar* OR social integrat* OR race* OR racis* OR integr* soc* OR household* OR demograph* OR famil* OR emargina* OR behavioral OR behavioural OR psych*)) AND AB ((predictor* OR driver* OR determinant* OR involv* OR starter* OR recruit* OR network* OR correlat* OR member* OR factor* criminal career* OR criminal trajector*))
Open Grey	("criminal organisation" OR "criminal association" OR "organized crime" OR "organised crime" OR mafia OR gang* OR criminal gang* OR crim* network* OR DTO* OR drug trafficking organ* OR drug cartel* OR motorcycle gang* OR criminal group* OR crim* cartel OR "Italian Mafia" OR "Russian Mafia" OR "Japanese Mafia" OR "Chinese Mafia" OR narco* boss*) AND (disadvantag* OR econom* OR salar* OR inequalit* OR povert* OR occupation* OR inequalit* OR migrant* OR immigration* OR "social embeddedness" OR school* OR environment* OR scholar* OR social integrat* OR race* OR racis* OR integr* soc* OR household* OR demograph* OR famil* OR emargina* OR behavioral OR behavioural OR psych*) AND (predictor* OR driver* OR determinant* OR involv* OR starter* OR recruit* OR network* OR correlat* OR member* OR factor* OR criminal career* OR criminal trajector*) NOT(narcosis OR ganglion* OR narcolept* OR marathon* OR organ* OR maraviroc* OR gangetic* OR gangue OR "marangoni" OR narcoleps* OR ganger OR mafic OR maranh*) lang:"en"
ProQuest (English)	AB("criminal organisation" OR "criminal association" OR "organized crime" OR "organised crime" OR "mafia" OR gang* OR criminal gang* OR crim* network* OR dto* OR drug trafficking organ* OR drug cartel* OR motorcycle gang* OR criminal group* OR crim* cartel OR "Italian Mafia" OR "Russian Mafia" OR "Japanese Mafia" OR "Chinese Mafia" OR narco* boss*) AND AB(disadvantag* OR econom* OR salar* OR inequalit* OR povert* OR occupation* OR inequalit* OR migrant* OR immigration* OR "social embeddedness" OR school* OR environment* OR scholar* OR social integrat* OR race* OR racis* OR integr* soc* OR household* OR demograph* OR famil* OR emargina* OR behavioral OR behavioural OR psych*) AND AB(predictor* OR driver* OR determinant* OR involv* OR starter* OR recruit* OR network* OR correlat* OR member* OR factor* OR criminal career* OR criminal trajector*)
PubMed	("organised crime"[Title/Abstract] OR "criminal organization"[Title/Abstract] OR "mafia"[Title/Abstract] OR gang[Title/Abstract] OR "drug trafficking organization"[Title/Abstract]) AND (recruitment[Title/Abstract] OR factor[Title/Abstract] OR risk[Title/Abstract] OR predict[Title/Abstract]) AND (social[Title/Abstract] OR economic[Title/Abstract] OR psycholog[Title/Abstract])
Scopus	(TITLE-ABS-KEY (("organised crime" OR "criminal organization" OR "mafia" OR gang OR "drug trafficking organisation")) AND TITLE-ABS-KEY ((recruitment OR factor OR risk OR predictor OR social OR economic OR psycholog)) AND NOT TITLE-ABS-KEY ((gangl OR narcosis OR narcolept OR

Database	Queries
	marathon OR organ OR organs OR maraviroc OR gangetic OR steroids))) AND (LIMIT-TO (SUBJAREA, "SOCI") OR LIMIT-TO (SUBJAREA, "MEDI") OR LIMIT-TO (SUBJAREA, "PSYC") OR LIMIT-TO (SUBJAREA, "ARTS") OR LIMIT-TO (SUBJAREA, "ECON") OR LIMIT-TO (SUBJAREA, "BUSI") OR LIMIT-TO (SUBJAREA, "NURS") OR LIMIT-TO (SUBJAREA, "NEUR") OR LIMIT-TO (SUBJAREA, "HEAL")) AND (LIMIT-TO (SRCTYPE, "j") OR LIMIT-TO (SRCTYPE, "b") OR LIMIT-TO (SRCTYPE, "k") OR LIMIT-TO (SRCTYPE, "p") OR LIMIT-TO (SRCTYPE , "d"))
Web of Science	(TI=("criminal organisation" OR "criminal association" OR "organized crime" OR "organised crime" OR mafia OR crimin* OR gang* OR criminal gang* OR crim* network* OR DTO* OR drug trafficking organ* OR drug cartel* OR motorcycle gang* OR criminal group* OR crim* cartel OR "Italian Mafia" OR "Russian Mafia" OR "Japanese Mafia" OR "Chinese Mafia" OR narco* boss*) AND TI=(disadvantag* OR econom* OR salar* OR inequalit* OR povert* OR occupation* OR inequalit* OR migrant* OR immigration* OR "social embeddedness" OR school* OR environment* OR scholar* OR social integrat* OR race* OR racis* OR integr* soc* OR household* OR demograph* OR famil* OR emargina* OR behavioral OR behavioural OR psych*) AND TI=(predictor* OR driver* OR determinant* OR involv* OR starter* OR recruit* OR network* OR correlat* OR member* OR factor* OR criminal career* OR criminal trajector*)) AND LANGUAGE: (English); Indexes=SCI-EXPANDED, SSCI, A&HCI, CPCI-S, CPCI-SSH, BKCI-S, BKCI-SSH, ESCI, CCR-EXPANDED, IC Timespan=All years
Google Scholar	(milieu OR organisat* criminelle* OR criminalité organisée OR criminels organisés OR cartel criminel OR mafia OR gang*) AND (economi* OR social* OR psych* OR facteur* OR risq* OR recrut*)
Sudoc.Abes	(milieu OR organisat* criminelle* OR criminalité organisée OR criminels organisés OR cartel criminel OR mafia OR gang*) AND (economi* OR social* OR psych* OR facteur* OR risq* OR recrut*)
Sowiport	("organisierte kriminalität" OR kriminelle* organisation* OR kriminelle* vereinigung* OR kriminelle* gruppe* OR bande* OR bandenkriminalität OR kartell* OR gang* OR bandenmäßig* OR mafia* OR mafiaähnlich* OR motorradclub* OR rockerclub*) AND (armut* OR benachteilig* OR ungleichheit* OR arbeitslosigkeit OR ausländ* OR migrant* OR famil* OR analphabetismus OR bildung* OR ausbildung* OR schul* OR umgebung* OR faktor* OR prädiktor*)
Liliacs	(pandillas OR mafia OR "grupo criminal" OR "asociacion criminal" OR "crimen organizado" OR cartel OR "delincuencia organizada") AND (riesgo OR reclutamiento OR "carrera criminal" OR factor)
ProQuest (Spanish)	(pandillas OR mafia OR "grupo criminal" OR "asociacion criminal" OR "crimen organizado" OR cartel OR "delincuencia organizada") AND (riesgo OR reclutamiento OR "carrera criminal" OR factor)
Riviste Web	("crimine organizzato" OR "criminalità organizzata" OR "associazione delinquere" OR mafia OR gang OR bande OR "organizzazione criminale") AND (reclut* OR fattor* OR rischi* OR carriera)

MULTISTAGE APPROACH TO SEARCHING

Apart from identifying relevant literature through scientific databases, researchers also contacted several scholars in the field of OC to receive

suggestions on relevant studies that may not have been included in the systematic review yet. The contacted experts were: Jay Albanese (Virginia Commonwealth University, USA), Paolo Campana (University of Cambridge, UK), Scott Decker (Arizona State University), Edward Kleemans (Vrije University of Amsterdam, NL), Klaus Von Lampe (John Jay College of Criminal Justice, USA), Carlo Morselli (University of Montreal, CA), Letizia Paoli (Katholieke Universiteit Leuven, BE), David Pyrooz (University of Colorado Boulder, USA), Sonja Wolf (Centro de Investigación y Docencia Económicas, MEX).

The research team further identified relevant literature from the bibliographies of the studies that were selected for full-text screening. Like the selection of studies from the database searches, these additional studies were assessed for full-text eligibility.

Selection of studies

Preparatory activities

The review process incorporated all studies that were retrieved from searching the databases. Metadata for each study was imported to the Covidence platform which provides an environment to manage and conduct systematic reviews.[16] As a first step, duplicates were identified and removed. This led to a reduction from 50,291 to an eventual number of 48,731 unique studies.

The next step of the review, the preliminary abstract screening, was conducted by a team of six researchers that underwent specific training. The training included a comprehensive briefing on the purpose and scope of the systematic review, followed by a tentative screening phase during which each reviewer independently screened a set of 100 studies. The results of this screening were discussed among all researchers to reveal divergent interpretations and other issues, and maintain common criteria for the inclusion of studies in the systematic review.

Title and abstract screening

The next step of the review consisted in the screening of the title and abstract of each of the studies that were identified from searching the databases.

The inclusion criteria for the title and abstract screening were:

---

[16] The Covidence platform is a core component of Cochrane's review production toolkit improving the production of systematic reviews. It allows to import citations of the studies included in the systematic review and fasten the screening phase by enabling the members of the review team to collaborate and perform the double-checked screening simultaneously keeping track of all passages.

1. The document reported on OCGs
2. The document addressed factors leading to the recruitment to OCGs
3. The document was academic and makes an original research contribution (e.g. no news articles or reviews or any type)

Out of the 48,731 initial records, 48,613 did not meet the inclusion criteria. The title and abstract screening thus resulted in the identification of 118 records to be included in the next stage of the systematic review (see Figure 2).

To ensure reliability, two reviewers screened each document. A third researcher settled divergent screening decisions, or where necessary in consultation with the full review team. The share of non-uniform decisions between two researchers remained below 10% of all screened documents. When it was unclear whether a study met the inclusion criteria, e.g. because the abstract was missing or provided limited information, it was kept for full-text screening.

ELIGIBILITY FOR FULL-TEXT SCREENING

Based on the title and abstract screening, an initial number of 118 studies were deemed suitable for full-text screening (see Figure 2). Among these, however, the full texts for 8 studies could not be retrieved. These studies had thus to be excluded from the review, because: (i.) an electronic version was not available, (ii.) a paper-based version could not be retrieved, and (iii.) the corresponding authors or publishing institutions were unable to provide a copy of the requested document.

Based on the experts' suggestions and identification of additional literature from the bibliographies of relevant studies, 20 documents were added. This made for a total number of 130 studies that entered full-text screening.

FULL-TEXT SCREENING

The full-text screening criteria were:

1. The document reported on OCGs
2. The document addressed factors leading to the recruitment to OCGs
3. The document was academic and makes an original research contribution (e.g. no news articles or reviews or any type)
4. The text was written in English, French, German, Italian, or Spanish
5. The study was empirical

The full-text screening criteria resulted in the identification of 57 empirical studies (see Figure 2). Among the 73 documents excluded during the full-text screening, 3 were in languages other than the ones considered in the systematic review (i.e. Japanese and Russian), 12 were out of scope (mostly

on youth gangs), and 58 were non-empirical (i.e. theoretical contributions or documents discussing contextual factors related to OCGs).

A detailed classification of the findings of each study allowed to further screen the empirical studies. The research team organsed the relevant contents according to the following criteria:

- The study's approach, i.e. quantitative, qualitative, or mixed-methods
- The unit of observation, i.e. individuals, groups, regions, countries, transcripts, or songs
- The number of observations included in the sample
- The data collection method, i.e. interviews, survey, observation, tapping, investigative files, or official data
- The type of analysis conducted, e.g. descriptive statistics, regressions, social network analysis, content analysis
- The factors identified in the study, e.g. age, social relations, and reputation
- For quantitative studies, the direction (i.e. positive or negative) of the effect of each factor on the dependent variable (i.e. OCGs membership)

Based on this classification, 6 out of the 57 quantitative empirical studies were excluded because they had a unit of observation other than individuals.

The remaining 51 studies were assessed for their quality using the amended CASP checklist tool. This checklist considers the clarity of the research aim as well as the appropriateness of the research methodology, design, recruitment strategy, and data collection. It also considers the rigorousness of data analysis, the clarity of the statement of findings, and the overall research value. Eventually, the authors' critical self-evaluation and attention to ethical issues are considered. Due to their low-quality, 4 qualitative studies were excluded. The research team agreed on excluding these studies based on their insufficient ratings on two key items of the CASP checklist, namely the appropriateness of the research design and the rigorousness of the data analysis.

Studies included

Out of the 48,731 initial records, plus the studies suggested by experts or stemming from the bibliographies of relevant studies, only 47 met all criteria for inclusion in the systematic review (see Figure 2).[17] The final literature list consisted of 12 quantitative, 8 mixed-methods, and 27 qualitative studies.

---

[17] To ensure reliability, two reviewers screened each document and the full review team settled divergent screening decisions, which remained below 10% of all screened documents.

Corroboration of findings

Results stemming from the studies included in this systematic review were corroborated through the analysis of the Italian grey literature on the mafias. The reports issued by the Italian Parliamentary Anti-Mafia Commission (CPA), the Italian National Anti-Mafia and Counterterrorism Directorate (DNA), and Anti-Mafia Investigative Directorate (DIA) validate the results on the Italian mafias, adding some contextual information. These literature included all reports issued periodically by the CPA between 1975 and 2017, annually by the DNA between 1999 and 2017, and biannually by the DIA between 1998 and 2017. Most reports were available on the institutions websites and missing ones were directly requested to competent authorities.[18]

Only reports containing relevant information for the purposes of this systematic review were used to corroborate results. The search for relevant reports relied on two sets of keywords (i.e. see Table 6, categories B e C). The included grey literature consisted of 8 reports from CPA, 23 from DNA, and 9 from DIA.

[18] Specifically, CPA and DIA reports are available at http://www.camera.it and http://direzioneinvestigativaantimafia.interno.gov.it, respectively. DNA reports are not indexed in any database but downloadable from the internet.

CHAPTER 2: Ethical and Societal Impacts of Organised Crime Policies (UB-CREA)

Authors: Lídia Puigvert-Mallart; Emilia Aiello, Tinka Schubert

Table of contents

INTRODUCTION 73

1. METHODOLOGY AND SCOPE 74

1.1. DEFINING WHAT IS 'ETHICAL IMPACTS' AND 'SOCIETAL IMPACTS' 75
1.2. DATA COLLECTION TECHNIQUES 76
DESK RESEARCH ON SCIENTIFIC LITERATURE AND GREY LITERATURE ABOUT THE IMPACT OF POLICIES THAT FIGHT AGAINST OC 76
DESK RESEARCH FOR POLICY MAPPING ON THE POLICIES AND PROTOCOLS IN THE EU MEMBER STATES REGARDING OC 76
QUALITATIVE FIELDWORK 77
BARRIERS ENCOUNTERED TO CONDUCT THE FIELDWORK FORESEEN 78

2. FINDINGS 79

2.1. LITERATURE REVIEW ON THE IMPACT OF ORGANISED CRIME AND PUBLICATIONS ABOUT THE TRANSPOSITION AND IMPACT OF THE FD2008/841/JHA ABOUT ORGANISED CRIME 79
2.2. ETHICAL IMPACTS AND SOCIETAL IMPACTS OF OC POLICIES: RESULTS FROM THE QUALITATIVE FIELDWORK 82
EXCLUSIONARY DIMENSION FOUND IN THE IMPACT ANALYSIS OF OC POLICIES: ELEMENTS THAT HINDER THE ACHIEVEMENT OF POSITIVE SOCIETAL IMPACTS AND THE PROTECTION OF FUNDAMENTAL RIGHTS 82
TRANSFORMATIVE DIMENSION FOUND IN THE IMPACT ANALYSIS OF OC POLICIES: ELEMENTS THAT PROMOTE THE ACHIEVEMENT OF POSITIVE SOCIETAL IMPACTS AND THE PROTECTION OF FUNDAMENTAL RIGHTS 90
2.3 CONTRIBUTIONS TO INFORM THE OPERATIONALISATION OF THE INPUT FOR ABM SIMULATIONS 103

3 FINAL REMARKS 104

4 REFERENCES 106

ANNEX I. DEFINITION AND EUROPEAN LEGISLATION ON ORGANISED CRIME 108

DEFINITION 108
EUROPEAN LEGISLATION 111

ANNEX II. FIELDWORK STUDY PARTICIPANTS 116

FIELDWORK STUDY PARTICIPANTS.. 116

ANNEX III. GUIDELINES FOR QUALITATIVE FIELDWORK 122

INTRODUCTION

In this report there are presented the main findings of Task 1.2., conducted with the general aim of *analysing the societal and ethical impact of specific organised crime policies to make sure that PROTON supports the development of better evidence-based policies.*

In order to embrace the task three main activities have been conducted: desk research on scientific literature and grey literature about the *impact of policies that fight against organised crime* (hereinafter, OC); desk research for policy mapping on the policies and protocols in the EU member states regarding OC, and as our specific aim was to identify the impacts, and of most relevance for the aim of the report, qualitative fieldwork has been conducted, analysing six case studies (Germany, Italy, The Netherlands, Romania, Spain, and UK).

The methodology used was the communicative methodology of research (Gómez, Puigvert, Flecha, 2011), which focused on analysing the 'transformative' (those elements that contribute to transform a given reality) and 'exclusionary dimension' (those elements that perpetuate a condition of inequality or/and which hindrance the transformation) of data. In order to analyse the 'societal and ethical impacts' and set a standard for a definition of what was understood by 'societal impacts' and 'ethical impacts' we have drawn on the existing 'Better regulation: guide lines and toolbox' (European Commission, 2017).

Considering the complexity of carrying out a societal and ethical impact analysis of the policies which fight against organised crime for not identifying a set of specific policies in the field of organised crime, but multiple [the own nature of what is considered 'organised crime' under the FD2008/851/JHA], the added value of this T.1.2 has been therefore, the identification done in dialogue with stakeholders and end-users of those 'transformative elements' (positive impacts) and 'exclusionary elements' (negative impacts), which are being taken into account in their policies based on programmes, actions and strategies implemented for fighting against OC in the selected country of the fieldwork. This is key in order to provide further insights of the factors that either promote or prevent the recruitment into OC groups, contributing to the elaboration of *dialogic evidence-based policies* informed by PROTON. Drawing from this, these programmes, actions and strategies analysed respond to and implement the existing policies which fight against organised crime.

As final remarks, the research conducted up to this point, the literature reviewed and the fieldwork carried out, evidences that in the field of fighting

against OC the measures and strategies are more oriented to the disruption of the networks (at the system level) instead of the prevention (at the subject level: equipping those more vulnerable with 'protective factors' to prevent falling in OC, protecting their fundamental rights). Policies and strategies in OC are related to legal actions, police and justice cooperation and investigation means. In this way, the EU and in consequence the MSs have strategies and protocols to deal with the different manifestations of OC in a more integral perspective. In other words, every member state has also specific measures regarding specific OC threats, but policies of OC in general, are focused on very particular areas such as police investigations and justice cooperation. More specifically, the great majority of experts, stakeholders and end-users interviewed highlighted the key importance of developing more actions and programmes focused to protect those more vulnerable to be recruited.

1. METHODOLOGY AND SCOPE

The methodology used has been the communicative methodology of research, an approach characterised for its dialogic orientation, which has been recognised by its transformative potential (Sordé & Mertens, 2014). Scientific publications have analysed in-depth the reasons why this methodology is effective in contributing to the scientific, social and political impact of the research' results, outlining the suitability of this methods to conduct studies with those social groups most of the time excluded from scientific research such as ethnic minorities (Flecha, 2014; Gómez et al., 2011; Sordé & Mertens, 2014). These publications have pointed at the own premises on which the communicative methodology stands (for instance, the adoption of a dual conception of reality, which includes *systems* and *lifeworld [Habermas, 1984]),* as well as the methodological strategies that it uses in order to include the voices of the 'researched' subjects as key elements to illuminate new knowledge for informing social change.

Collected data has been analysed according the dimensions defined by the communicative methodology: the *transformative* and *exclusionary* dimension. This way of analysing data was what defined the transformative scope of the current study, as its aim was not only to analyse and describe reality identifying the exclusionary components, but also identifying the transformative effects of those actions implemented in the prevention of both recruitment in organised crime and terrorist networks that have demonstrated to be successful in order to overcome them. This said, as the *transformative dimension* were considered all those elements that foster positive social and ethical impacts, and as *exclusionary dimension* those elements that hindered them.

1.1. Defining what is 'ethical impacts' and 'societal impacts'

As mentioned in the Introduction, the 'societal impacts' and 'ethical impacts' have been defined drawing on the existing *'Better regulation: guide lines and toolbox'* of the European Commission. This regulation sets out the principles that the European Commission follows when preparing new initiatives and proposals and when managing and evaluating existing legislation. The advice of using as a departing point this Toolbox derived from the existing work of the *Expert Group on Evaluation Methodologies for the Interim and Ex-post evaluations of Horizon 2020*, whose members were in dialogue with the research team of the FP7 IMPACT-EV project (2014-2017), of which Prof Lidia Puigvert is member. As observed, both the Expert Group and the IMPACT-EV project were committed for the analysis of impacts of 'research projects', and the T.1.2. was oriented to the analysis of the impacts of 'policies'. However, we relied on advice derived from these consortiums for the definition of impacts (to set a methodological criteria) rather than of generating for PROTON T.1.2 and T.1.2. new understandings of both 'societal and ethical impacts'.

This clarification done, the #Tool 19 of the Better regulation 'Toolbox'[1] was used for the definition of 'ethical impact' and 'social impacts'. For the 'ethical impacts', we included the 'Fundamental rights impacts' of the #Tool 19, which capture the different dimensions of the fundamental rights:

1. General; 2. Dignity; 3. Individuals, private and family life, freedom of conscience and expression; 4. Personal data; 5. Asylum and protection of removal, expulsion or extradition; 6. Property rights and the right to conduct a business; 7. Gender equality, equality treatment and opportunities, non –discrimination, and rights of persons with disabilities; 8. Rights of the child; 9. Good administration / Effective remedy/ Justice.

And we defined in turn,

- *Transformative dimension:* Elements that contribute to protect and promote the fundamental rights of a person
- *Exclusionary dimension:* Elements that affect the protection and promotion of the fundamental rights of a person

For the 'societal impacts', we included the 'social impacts' identified by the #Tool 19, which are indeed elements linked to advancing the realisation of economic, social and cultural rights:

1. Employment; 2. Working Conditions; 3. Effects on income, distribution, social protection and social inclusion; 4. Governance, participation and good administration; 5. Public health and safety health systems; 6. Crime, Terrorism and Security; 7. Education & Training and education & training systems; 8. Culture; 9. Social impacts in third countries

---

[1] This Toolbox complements the better regulation guidelines presented in SWD (2017) 350. It should be mentioned here that 'Tool 19 #Identification/screening of impacts' of Chapter 3: Identifying Impacts in IAs, Evaluations and Fitness Checks" [SWD(2017)350, corresponds to the "**Tool 16** #Identification/screening of impacts' of Chapter 3 of the previous version of this guideline: for the complementation of Better Regulation Guideless presented in SWD(2015).

And we defined,

- *Exclusionary dimension:* Elements that advance the social impacts
- *Transformative dimension:* Elements that hampers the social impacts

1.2. Data collection techniques

DESK RESEARCH ON SCIENTIFIC LITERATURE AND GREY LITERATURE ABOUT THE IMPACT OF POLICIES THAT FIGHT AGAINST OC

The scientific literature review was conducted considering the following criteria: articles in the database Web of Science and published after 2012 (within the 5-years period 2012-2017). We have used keywords either targeting OC in general or dealing with its particular forms (i.e. counter organised crime + impact; migrant smuggling + impact; illicit drug trafficking + impact; money-loundering + impact; etc.). Scientific research projects funded by EU-funding were also revised.

Additionally, grey literature was consulted, especially from European and national country institutions concerned to either OC or CT such as the EUCPN, the RAN, Europol, OSCE, and other organisms from the European Commission, and national country agencies in charge of security issues.

DESK RESEARCH FOR POLICY MAPPING ON THE POLICIES AND PROTOCOLS IN THE EU MEMBER STATES REGARDING OC

We have done a mapping on the policies and protocols in the European Union (EU hereinafter) Member States (MSs hereinafter) regarding oc. All our data regarding European policies and measures were of public access through different web searchers such as http://eur-lex.europa.eu/, while the information regarding policies and strategies of the EU MSs were gathered through public sources (the corresponding Ministry in the field of OC for each country).

We have mapped the policies for specific OC areas (human trafficking, corruption, drug trafficking and cybercrime)[2] for the six countries in which fieldwork was conducted.

For this task, barriers were encountered due to the complexity of identifying all legislations and strategies in relation to OC (many types of crime are considered) for all the EU member states. Given that this task had already been done in previous research, we focused on the general definition of OC following suggestions from the coordinator. Mapping legislation for each type of crime was out of scope for the time given to the whole task.

---

[2] These particular threats of OC were chosen considering the Serious and Organised Crime Threat Assessment 2017 (SOCTA report elaborated by EUROPOL and also the Euro Crimes (art. 83(1) TFEU).

QUALITATIVE FIELDWORK

Qualitative fieldwork was conducted in six EU countries: Italy, Germany, The Netherlands, Romania, Spain and The United Kingdom. The fieldwork was conducted for OC and TN together (extensive literature and legal documents have already recognised the tight linked between both).

We selected experts, stakeholders and end-users of different domains, which were considered relevant for the analysis of the impact of policies in OC, CT and in both. These domains are *media, prison, migration, religion, education and neighbourhoods.*

In order to reach the subjects, we followed the coming criteria. First, in the case of experts, we tracked persons with sound evidence of their expertise in the subject. For the majority of cases, these are people who have a long scientific record in the field, publications in top-ranked journals, and as evidence of their knowledge on the topic, they have participated in European and national research projects. Besides, many of these experts are also people who have been involved in the elaboration of policies, participating directly in the process of policy-making (policy-makers) or indirectly being asked to contribute with their academic expertise to assess policy-makers (academics).

A snowball strategy was followed, contacting first experts and stakeholders and later on, end-users. Regarding stakeholders, persons active at the public sphere in relation to OC were reached. For instance, NGOs or associations specifically created with the aim of preventing some type of OC, either public or private organisms which with their task develop a preventive work in OC (e.g.: entities developing education programs located in the most vulnerable neighbourhoods, NGO focused on media literacy, NGOs carrying out prison voluntary work, etc.).

Finally, end-users were contacted as individuals recommended by the own stakeholders in the six countries or chosen for being recognised through social media networks and among the research team networks for being very active at the grassroots level developing a task in preventing the recruitment in organised crime networks. The majority of end-users selected to participate in the fieldwork are lay individuals that due to their background (belong to ethnic and cultural minorities, are involved in neighbourhood associations, migrants' associations, and others) can provide key information on the impact of the policies and programmes in their daily lives.

Fieldwork involved mainly semi-structured interviews, and in a less extent focus groups, which were conducted jointly with relevant stakeholders and end-users, and daily life stories with end-users.

The guidelines[3] used for the interviews are based on the #Tool 19 of the European Commission 'Identification/screening of Impacts'. Nevertheless, every guideline referred to the specificities of the country being studied, and

[3] Examples of the guidelines used is annexed. See Annex 2: Country Samples of a general guideline for qualitative fieldwork.

was modified depending on the profile of the expert or stakeholder in order to obtain as much information as possible regarding ethical and societal impacts. Moreover, each interviewee was provided with a consent form giving detailed information about the PROTON project and his/her participation in the fieldwork.

The following table summarises the overall techniques done in the qualitative fieldwork: [4]

**December 2017**	
Interviews with experts	34
Interviews with stakeholders (not foreseen in the initial fieldwork)	29
Daily life stories with end-users	8
Focus groups with stakeholders	7
**Overall**	**77**

**ESPANYA**	
Interviews with experts	7
Interviews with stakeholders	1
Daily life stories with end-users	6
Focus groups with stakeholders and end-users	3
**FINAL Nr. OF TECHNIQUES DONE: 17**	

**ITALY**	
Interviews with experts	5
Interviews with stakeholders	3
Daily life stories with end-users	0
Focus groups with stakeholders and end-users	0
**FINAL Nr. OF TECHNIQUES DONE: 9**	

**UNITED KINGDOM**	
Interviews with experts	6
Interviews with stakeholders	5
Daily life stories with end-users	0
Focus groups with stakeholders and end-users	2
**FINAL Nr. OF TECHNIQUES DONE: 13**	

**GERMANY**	
Interviews with experts	7
Interviews with stakeholders	5
Daily life stories with end-users	1
Focus groups with stakeholders and end-users	2
**FINAL Nr. OF TECHNIQUES DONE: 15**	

**ROMANIA**	
Interviews with experts	3
Interviews with stakeholders	7
Daily life stories with end-users	0
Focus groups with stakeholders and end-users	0
**FINAL Nr. OF TECHNIQUES DONE: 10**	

**NETHERLANDS**	
Interviews with experts	6
Interviews with stakeholders	7
Daily life stories with end-users	1
Focus groups with stakeholders and end-users	0
**FINAL Nr. OF TECHNIQUES DONE: 14**	

BARRIERS ENCOUNTERED TO CONDUCT THE FIELDWORK FORESEEN

As mentioned, all the profiles initially contacted were looked for according if they were representative of at least one of the six domains of our analysis. This provided in many cases very concrete information for the analysis of the impact of policies in specific domains, ensuring that experts and stakeholders would contribute with new information from new perspectives. However, this

[4] In order to see the entire list of participants in the fieldwork see *Annex 3. Fieldwork study participants*

categorisation also delayed the fieldwork as when someone (expert, stakeholder), from a specific domain declined to participate we needed to proceed to find a similar profile (for instance, expert in the field of religious affairs with expertise in terrorism and organised crime) to fill the gap.

Another barrier encountered was for conducting focus groups with stakeholders and end-users. In order to overcome this difficulty and do not delay the entire work, we decided to conduct interviews with stakeholders. Thus, through a different technique we were able to respond to our final aim. Stakeholders provided extremely useful information, sending grey literature of their organisations, reports that they have themselves either collected, participated in their elaboration or which considered that would be of our interest. However, as it is understandable, in many cases they were reluctant to put us in contact with end-users as their concerned that this would involve approaching sensitive issues for them.

Finally, more than 200 people were invited to participate in the fieldwork (some of them accepted, other declined, and some of them did not reply to the petition), what can be also considered for the dissemination of the existence of PROTON (the email sent had the most relevant information of the project, as well as its website).

2. FINDINGS

2.1. Literature review on the impact of Organised Crime and publications about the transposition and impact of the FD2008/841/JHA about organised crime

Our review indicates that there is no available scientific literature about the general topic of OC and the impact of policies focused on prevention. Limited literature has been identified specifically focused on one type of OC: the impact evaluation of Human Trafficking policies.

SUMMARY	
counter organised crime + social impact	4 articles found, none of them useful for the scope of T.1.2.
organised crime + policy + Europe	32 articles found, not useful for the scope of T.1.2.
counter narcotics + organised crime	9 articles found, the most of them outside the context of the EU and not useful for the scope of T.1.2.
counter narcotics + impact	41 articles found, the majority of them about health issues
transnational organised crime + Europe	13 articles found, none of them useful for the scope of T.1.2.
Counter migrant smuggling + impact	1 article found, related to health issues
migrant smuggling and impact	11 articles found, none of them useful for the scope of

	T.1.2.
illicit drug trafficking + impact	33 articles found, four potentially useful for T.1.2.
counter illicit drug trafficking	23 articles found, one potentially useful for T.1.2.
illicit trafficking firearms	17 articles found, one potentially useful for T.1.2.
Schengen Information System	5 articles found, one potentially useful for T.1.2.

As identified in the grey literature review, the FD2008/841/JHA has been evaluated and assessed in multiple occasions and by different experts and institutions. For instance, the Commission issued a report in 2016 to the European Parliament and the Council based on Article 10 of Council FD 2008/841/JHA, which states that:

> *The framework decision does not achieve the minimum degree of approximation of acts of directing or participating in a criminal organisation on the basis of a single concept of such an organisation. Most Member States have adopted self-standing offences in relation to participation in a criminal organisation in accordance with Article 2. Two Member States have not done so, but many Member States have gone beyond the minimum requirements. Some of them make the national provisions broader by not referring to all elements of the definition of organised crime, e.g. they do not mention the criterion of benefit or scope of predicate offence.* (European Commission, 2016, p.2)

In other reviews, for instance the European Parliament, Directorate general for internal policies elaborated a document with similar conclusions:

> *The Framework Decision provides a sophisticated framework of criminalisation on participation in a criminal organisation. However, it requires improvement both in terms of legal certainty and its scope, and in terms of the level of harmonisation it achieves*. (Mitsilegas, 2011, p.1)

A key EU publication should be mentioned here, "Study on paving the way for future policy initiatives in the field of fight against organised crime":

> *This study is an evaluation of the practical application of legal and investigative tools stemming from Framework Decision 2008/841/JHA on the fight against organised crime, other EU and international regulations and national legislation. The aim of this study is twofold: To assess the impact of Framework Decision 2008/841/JHA and other relevant EU and national legislation on the fight against organised crime through comparative legal analysis. To provide a comparative analysis of investigative tools and other measures used at the national and EU level for the purpose of fighting organised crime, with a focus on the operational results of these tools*.[5]

We add in what follows, the overall conclusions of the study regarding compliance with the Framework Decision and its impact (see section 11,5 of

[5] To see the entire document, see: https://publications.europa.eu/en/publication-detail/-/publication/a1183e4b-1164-4595-a742-fb4514ddd10d.

the Report: Conclusions, pg. 488). Indeed, these conclusions are worth to be included in the present report as they comprise the overall impact of *2008/841/JHA* and complements from a legal approach the results for T.1.2.:

- *The Framework Decision differs considerably from its original proposal and the most important provisions are optional*
- *Motivations for creating organised crime legislation are primarily national, rather than stemming from a need to comply with the Framework Decision*
- *Most MS were compliant with the minimum standards before the Framework Decision was issued*
- *The Framework Decision should be seen in the context of a range of other measures in the fight against organised crime*
- *MS law often goes beyond the minimum standards set out in the Framework Decision*
- *Following from the principle of minimum harmonisation, transposition of the Framework Decision is in some instances too broad*
- *While national legislation is often broadly worded, it was reported to be infrequently used in practice*
- *Compliance through case law or jurisprudence may increase uncertainty*
- *It is not clear that future, additional legislation would address the limitations of the Framework Decision*

To sum up, except for Denmark and Sweden, all MSs have introduced the key elements of the Framework Decision and introduced a self-standing offence of participation in a criminal organisation and/or conspiracy to commit offences (Varese, 2016). However, harmonisation among MSs is limited because of the broad and vague terminology of the Framework Decision.

2.2. Ethical impacts and societal impacts of OC policies: results from the qualitative fieldwork

In this section we explain the main findings of the qualitative fieldwork conducted. Results are structured according the two dimensions analysed by the communicative methodology of research, the *exclusionary* and the *transformative*. As mentioned in point 2 of this report, 'Methodology and Scope', the negative ethical and societal impacts are included as *exclusionary* elements of the measures, actions and programmes. On the contrary, the positive ethical and societal impacts are incorporated as 'transformative elements' of the policies. This structure allows seeing those elements that need to be taken into account in order to extend the societal and ethical impacts of EU policies that fight against organised crime.

Exclusionary dimension found in the impact analysis of OC policies: elements that hinder the achievement of positive societal impacts and the protection of fundamental rights

Positive transposition of the EU legal frameworks for preventing and tackling some types of organised crime but poor law enforcement at the local and regional level

Drawing from the policy-mapping of the EU legal framework and grey literature revised (Di Nicola et al., 2015) there is an agreement that for the case of 2008/841/JHA and other EU legislations that tackle specific types of organised crime there has been at the member states positive transposition of these frameworks. However, there is in some cases scarce capacity for the law enforcement at the local and regional level. This has been reported for instance, in the case of the implementation of legislation to tackle trafficking of human beings (EU Anti-Trafficking Directive 2011/36/EU). According to interviewees, the poor capacity for the enforcement of legislations by some EU member states forces most of the times non-profit organisations, which exercise their action in very specific local domains, to assume responsibility.

See what a stakeholder in Romania explained:

> "With the legal framework, Romania was one of the first countries to adopt it, but then we didn't really have implementation [of the Directive against human beings trafficking] (…) We do have good laws, but we don't have implementation: we don't have standard of care for survivors for example, which is crucial and then you have a law that involves a lot of actors, a lot of ministers… but then, down the line, you ask, ok who is responsible for this in your organisation, or in your Ministry, and people

> don't know… So it is on the map, but it is not anyone's priority" (Stakeholder, Romania)

Talking about the Serious and Organised Crime Strategy (2013) of the UK, a stakeholder interviewed commented in a similar way:

> "(…) they recognised that there is a fusion between terrorism and organised crime as the Organised Crime Strategy has been elaborated following the same structure than the Counter-Terrorism, … So, basically they are recognising the similarities, we know from where it is coming, and we need a comprehensive strategy to deal with both, but because everything has the same causation it makes sense to have a unified approach... The problem with that is that you have done all the talking, but you have not developed the grassroots organisations to deal with it on the ground, and everything moves in the ground" (Stakeholder, UK)

Thus, institutional actors in charge of the elaboration of the legislation need to develop also the framework to endorse them at the different levels of implementation, creating the conditions at the grassroots level to make the legislation possible.

Sexual affective relationship

Most of the policies, actions and programmes developed to fight against organised crime are not taking into account with the necessary emphasis the existence of an imposed dominant model of socialisation of adolescents into relationships that link attraction to violent behaviours (as broadly explored by the research on preventive socialisation of gender violence developed by Puigvert). This link is playing a role in recruitment and also for the very perpetration of the network. This plays a role in hampering, for instance, the collaboration of girls with police, as well as continue reproducing the dominant violent discourse.

- Resistances to collaborate with the police:

The identification of this violent model of attraction helps explaining why many girls who have relationships with young men involved in organised crime put resistances at the time of collaborating with the police. Girls and young women can become both, victims as well as facilitators of the networks:

> Interviewee: "Girls are victims but they are also facilitators. When they are facilitating one of the members of the OC networks we also investigate their role… And many times I have the feeling that they are not in it voluntarily, but most times they don't want to talk about it, so it is very hard to get an idea about if it is their own idea or if they are forced to do certain things. So it's hard…. Many times we don't focus on girls as victims. Of course we do in the Human Trafficking Unit, but in our investigations in which we try to tackle the criminal networks we most of the time see them as also being part of the criminal network (…) Many times they get involved through boyfriends… most women we see in our investigation is that they are girlfriends of the criminals… we also see

> aunts that play a role… There are family ties and relationships (…) [When investigations come] Most times they keep protecting them… We had a really hard time… We sometimes think: 'well, we now find a girlfriend and we know that she has to know more, so we think: "let's go to talk to her and invest on a good relation with her". We hope that she would tell us very much... and trying to get out of the criminal situation in which she got involved in. But many times, I don't hope… They don't choose for that; they choose to keep on protecting the people for which they work for.
>
> *Interviewer: Even that if this involves going to prison?*
>
> Interviewee: Yes, yes, yes… So I think that we should work out some other measures to deal with that because I think that they are really… they could be really important in providing us with information and also … And I think that in many times they are the victims too…. " (Stakeholder, The Netherlands)

- Reproduction of the dominant violent discourse

Not taking into account this very important aspect perpetuates the dominant discourse which links attraction to violence. The following quotations from the Netherlands and the UK provide an example of the importance of this aspect.

> "I've seen myself many times: 'the more violent or aggressive you behave, the higher you are in the ranks of the street. Violence is promoted and they are very attracted to it. I also see this in social media: they like to watch all types of videos of violent acts, they like to watch videos of people being very aggressive or violent, or they also like to take selfies with firearms, for example or such things. So, violence is very incorporated in their life since very early and very strongly (…) In relation to girls who get involved …. We see… in one of our biggest investigations, or projects, we had a special focus on women because we thought that we are always focused on males, so lets see and try to get a better view of what women are doing and then we see that many of these guys who are in these types of organisations have girlfriends who play a certain role and who also… quite seem to like the more type of violent male to be with (…) What we begin to see is that they [girls] many times facilitate also… So many times their rooms or houses function as special locations for drugs, or arms or other items used for the crimes. For example, one of these guys had to threaten or even shoot someone and he leaves his motorbike and the jacket he was wearing at his girlfriend's place…. For example… So we know for sure that she knows a lot more about what's happening and what he is doing… We are pretty sure that there are a lot of girls out there who know a lot about the criminal networks (…)" (Stakeholder, The Netherlands)
>
> "Many girls who have had babies with these gang boys, and then boys have gone into prison, and he's come out, he's left her, ...she stucks… when she thought that he loved her, and then she found out that she

> was just a passed in face. Who really wants a girl with no class? If that makes any sense… [The play is:] All what you want to do is sit with the boys, smoke with the boys, but you want to be the mother of my child. Really? Really? If you wasn't the mother of my child you would sit with me. But you are. And I can't do that. Because that is not what I am about. And none of them are about that. I have never meet anybody who would have their girls sitting with the team. If you have been in this game long enough you see…" (Stakeholder, UK)

There is multiple evidence of how this is influencing the perpetuation of networks and letting this issue unaddressed generating more gender violence and perpetuating gender inequalities.

In the UK, scarce protection of children, teenagers and other vulnerable people who are being exploited by county lines gangs:

In the UK there is high concern by stakeholders about the 'county lines gangs', which according to some interviewees remains under-address by institutional authorities. In this sense, actions are needed especially to protect children who are being used as 'mules', what constitutes an extreme violation of human rights.

County lines gangs is a new phenomenon in the UK. A report issued by the National Crime Agency of the UK (2016) attributes the following features to this type of gangs (see page 2):

Section 34(5) of the Policing and Crime Act 2009 defines gang-related drug dealing activity as 'the unlawful production, supply, importation of a controlled drug which occurs in the course of, or is otherwise related to, the activities of a group that:

a) Consists of at least 3 people; and
b) Has one or more characteristics that enable the members to be identified by others as a groups

For the purpose of this report, a county lines gang features these additional criteria:

- *The gang/gang members are based or have origin within urban city location*
- *They have established a market in a rural town, into which they supply drugs.*
- *They utilise a mobile phone number to facilitate drugs orders from and supply to costumers in the county town*
- *Their criminality systematically exploits young and vulnerable people*

As mentioned, stakeholders interviewed have explained that current actions are not effectively protecting children (they are not under the normal 'radar' of police) and other vulnerable people of falling victims to county lines gangs, such as Roma migrants or women who are most of the times sexually exploited in these networks.

In this sense, in response to the report of the UK National Crime Agency, the NGO Children's Safety from the UK released a press communication raising awareness about how vulnerable children are to being exploited:

> "Gangs are grooming the most vulnerable young people in our society with drugs and alcohol or promises of status and wealth, then using threats, violence and sexual abuse to coerce and control their victims. The coercion of children into drug dealing is traumatic and puts them at great risk – they are often forced to carry drugs inside their bodies, and sent across the country to stay in 'trap houses' and sell drugs to dangerous adults." [Children Society Press Release][6]

The following quotation shows how Travellers and Roma are exploited by county lines gangsters from London. In this sense, it seems like a hierarchy within the criminal groups is established, influenced by ethnicity (those most vulnerable and most at the margins, at the bottom –Roma and migrants), and by the level of violence executed (gangs from London consider themselves the most 'hard', and the other gangsters in county lines more 'soft'):

> "I've now discovered the connection between travellers groups in Kent and gangs in London: (possible Somali guys)… Romanian Roma used by the Somali… as traffickers…. This in the Margate Beach (this is part of the County Lines problems)" (Stakeholder, UK)

Women are, once more, revictimised -being victims and facilitators in this criminal activity. The interviewee emphasises how women are subdued to criminals and in most of the cases not opposing to it -see again the identification of a predominant violent model of attraction:

> "Women who have done offence of carrying drugs [in the county lines], and then others that have very complex needs. There are girls that get involved in gangs: sexual exploitation happens; siblings; girlfriends are in higher risk…: the attitude that girls have is astonishing, you would think that feminism never happened (…) You would not believe that we are living in the 21st century. So we challenge this stuff. And we are keen to do more". (Stakeholder, UK)

Stakeholders, practitioners working at the ground emphasise that prevention and intervention with girls suffering this situation should exist in those places where we do know that we can detect them, for instance in hospitals:

> "Another of our fantasy projects would be to have workers in the hospitals for girls: because we know that many of the girls go to the hospitals to ask for contraception. … They know that if a girl comes for the third time in a month is not for fun… if a nurse would have the information… but what about is there is a female worker based in a hospital… Would you have chat with our worker? And hopefully have a girl to open up". (Stakeholder, UK)

---

[6] To see the entire press release, visit: https://www.childrenssociety.org.uk/news-and-blogs/press-releases/the-childrens-society-responds-to-the-nca-report-on-county-lines.

In some cases, resistances of police officers in the units of OC at the top level to collaborate and share information with stakeholders working at the grassroots level hampers prevention

Some stakeholders interviewed have emphasised that, in many occasions, police officers working at the top level and prosecutors have resistances to collaborate and share information with those stakeholders who are working in prevention at the grassroots level. Often, there is a questioning form the side of police officers working at the top level [tackling serious crimes] of the importance of doing prevention activities for considering them neither relevant nor effective. This is observed in the reluctance to share information with other stakeholders, and in the devaluation of the positive effect of preventive programmes.

According to an interviewee, police officer at the top level working in an OC unit in the Netherlands, the sharing of information under specific circumstances and when there is agreement of confidentiality is key to better tracking cases and the networks of facilitators:

> "We should invest much in preventive measures. But I see that there is a lot of people which… [suggesting that 'people who do not support that]. Let me give an example: few months ago we had a meeting from my colleagues from [other cities], and from some National Departments, and there were also many prosecutors there. We had a presentation about lot of criminal networks, which commit lot of murders. And someone made a joke saying: Yes, some years ago someone said that prevention and youth worker would answer to do", and then everyone started laughing about that. But I don't think that this is something to laugh about, that is reality (…) There is a lot of work [in a preventive way] being done, but almost everything is on a local level. But our Serious Crime Unit, for which I work is not so good connected to local police stations… and also not to these local organisations… so there is not a good information flow from the street level, where for example youth workers and police officers work together on a problem. There is not much information going from them to us, and back. And I think that this should be organised better. I think that this should be done… That's why I gave the example of the joke that some high officer from the prosecution organisation made. People who are dealing with serious crime.. lot of people who are dealing with serious crime in the Netherlands, they don't really feel connected to preventive measures …. And they don't have an idea about that. There is not enough attention for the factors why many young people look up to criminals and want to become the same. When you work at the local police station you see young people who get in trouble and there is lot attention for them. But when they grow bigger, when they out of petty crimes and grow into a more serious crime we lose our sight on them [those agents working at the local level]. And they reach our attention again when they are big criminals… but then we don't do preventive measures any more, we only look at the criminal activities… I think that this is not a good situation.. And from our Serious Crimes

> Units and the Departments for which I work for there should be more connection to… better connection to the local level and also be more aware of preventive policies which would be more effective." (Stakeholder, The Netherlands)

The importance of sharing information among the different stakeholders to better approaching cases was also highlighted by another police officer interviewed (Netherlands), member of a Unit in charge of Trafficking of Human Beings. This stakeholder insisted that in order to track the chain there has to be more collaboration among the different actors.

Related to this, it is relevant to observe what is the position of stakeholders who are working at the grassroots level and who also explain that the attitude that police officers of the top units have is challenging and that indeed, collaboration is needed in order to tackle the recruitment and disrupt the networks. It is worth to mention what a stakeholder emphasises in this regard, that the fact that police and other actors working at the ground collaborate does not involve that police officers have to play the role of 'social works'. Drawing from this, policies should encourage the collaboration, defining what are the roles of each part:

> "Cooperation with local authorities in terms of extremism and organised crime: understanding what intervention actually is. Intervention takes a lot of time, a lot of listening. It is not the way the police system runs (…) However, they are needed…. Police officers are not social workers, people have to understand that. Polices do 'stop and search' -now they call it 'stop and detention', but it is actually the same although they change it the name". (Stakeholder, UK)

> "Sometimes the relation with the police, their attitude towards the victim can be problematic. Our job is to challenge this, to challenge this attitude. The role of our organisation is to advocate for the victims" (Stakeholder, UK)

Prisons and the risk situation for human rights abuse and adverse effects on health

For the case of Italy and Spain, two interviewees raised concern about the type of regime imprisonment, which those judged for the offence of being member of an organised crime network can be referred to. They explained a similar situation: the National Constitution in both cases affirms that imprisonment or other measures should be aimed at reintegration[7]. However, in some cases the regimes that are being implemented for those who enter into prison for having committed a crime related to the belonging of an organised crime network are not oriented towards their social reintegration into society. These interviewees

---

[7] "Punishments entailing imprisonment and security measures shall be aimed at rehabilitation and social reintegration and may not consist of forced labour" (Spanish constitution. Art. 25.2).
"Punishment must not consist of measures contrary to humane precepts and shall aim at reforming the person upon whom sentence is passed" (Italian Constitution Art. 27.3).

have explained that a hard regime of isolation and solitary confinement can be applied, with permanent intervention of their communications, among other measures. This constitutes a violation of human dignity. It is important to mention here that in Italy this only applies to the most severe cases of organised crime members, when it is found that the subject could still represent danger to society if being in contact with other individuals.

Despite this negative impact regarding prison conditions, it should be stressed that in Catalonia the inmates are not classified according to the crimes for which they had been sentenced. They receive an individual treatment depending on their needs. According to the expert of prisons in Catalonia, they have special programs for inmates who have committed white-collar crimes or drug trafficking crimes. These programs are focused on the compensation of damages to the society through community work with the aim of making them realise the damage that their crimes are causing to society. These programs would have a positive impact on the resocialisation of these inmates decreasing the probability to re-offending.

Racism among the institutional structures which prevent some type of legislations to be properly-endorsed

Some stakeholders interviewed have explained and raise concern about those situations in which the existence of racism among the very institutional structures (professionals of prisons, police officers, professionals of social affairs departments, health-care professionals), legislation for protecting victims of organised crime networks is not endorsed, causing a serious negative impact on human rights.

One of the interviewee, stakeholder working at the Department of Social Affairs in the Barcelona City Council explained the case of a 13-years old girl, Roma from Bosnia, which was under her supervision at the Unit for Child and Teenagers Attention:

> "She was a brilliant student at the school. We looked for scholarships for her because she wanted to continue. But suddenly, she disappeared. Her family said that she had been sent to Bosnia to take care of her grandmother who was ill. However, some months later we identified that she was with a baby at the prison centre in X. We investigated the case and of course we saw that she was there wit false documentation [she was a minor in a regular prison for over-18 year-old inmates]. The case was that she got a boyfriend, also a guy under the tutorisation of the Unit for Child and Teenagers Attention who was involved in an organised crime network specialised in thefts. So, she end up in the prison of X, and the in the women prisons of Y for this case. But we achieved to demonstrate that she was a minor and thus to move her from there and bring her to a juvenile reform centre. At that point she was pregnant again, and when she was brought to the hospital and had the baby she ran away. But com'on, will you tell me that all those prison workers and other professionals who have been with her have not realised that she was a minor? This occurred because she was Roma so nobody cares

> about it! They just excuse themselves under the argument that 'there is not a protocol for these cases'" (Stakeholder, Spain).

TRANSFORMATIVE DIMENSION FOUND IN THE IMPACT ANALYSIS OF OC POLICIES: ELEMENTS THAT PROMOTE THE ACHIEVEMENT OF POSITIVE SOCIETAL IMPACTS AND THE PROTECTION OF FUNDAMENTAL RIGHTS

In this section are described the positive impacts, those elements that are indeed contributing to enhance the ethical and societal impact of policies that fight against OC, and which when taken into account contribute to prevent people from being recruited into OC networks.

First, we have identified a set of actions oriented to stop people being drawn into different types of serious and organised crime:

1. Equipping vulnerable people with a set of educational and employment skills and a network of 'protective' community agents, creating links of solidarity, which prevent them to fall in OC networks

The actions that are described in what follows have shown to open up ways for the social integration of vulnerable communities at the grassroots levels, this for instance by means of creating educational opportunities for children and adult people, by means of creating employment opportunities that challenge the mafia or other type of illicit activities that can be an alternative way of surviving, or by opening up democratic spaces of social participation in the neighbourhoods where agents themselves give their views and organise regarding those issues that affect their daily lives and the lives of their families.

In most of the cases, these actions and programmes, although designed, implemented and managed at the local level, count with the support of EU organisms and thus are the deployment of specific policy actions. For instance, some of the initiatives that have been identified that are having an impact in diverting people from falling into OC networks are being carried out by local organisations that implement them through EU-funded projects.

***a) "But for them, the opportunity to set up their business and become entrepreneur, that's a big difference":* Active labour market policies and other programmes oriented to creating employment opportunities for those at risk of getting involved in gang crime or mafia-type activities.**

Several stakeholders working with youth people and other at-risk people of falling in OC networks have welcomed active labour market policies specifically oriented to them, and taking into account their needs and also their motivations. This is a common issue highlighted by practitioners: those active labour market polices and other employment opportunities which capitalise the entrepreneurial skills and leadership capacity of those young people who have been involved in organised crime networks (gangs, drug-dealing, gambling,

among others) have a major societal impact. Therefore, actions that take into account these acquired 'skills' from the delinquent activity and the motivations of former criminals, and divert them towards achieving positive outcomes are more effective than those that do not.

> *"People involved in gangs have amazing entrepreneurial skills, so it just depends on how you focus these skills"* (Stakeholder, UK)

The same is emphasised by another stakeholder, who remembers that it should also be considered that sometimes it is unrealistic to take for granted that a person who has a criminal record will be easily employed (they are less 'employable'). For this reason, programmes oriented to capitalise their skills and promote them to become entrepreneurs are even more attractive:

> "But when a young person has been involved in criminal activity, there is no point in saying to them (…) "you can get a job"… because they have a criminal record… [referring that it is difficult for them to get a formal job due to the criminal record, although the person wants to be employed]. I did volunteer with an organisation that works with these people to help them set up social business. Because like it or not, they are good business people… they know how to negotiate, they are good dealing with lots of money and are used to that, they are used to doing business from a very early age, they are used to all of those things... So let's take that skills, harnessing these skills and give them set up social business. [Explains about an organisation in the community] What they do is going to the banks, and the banks will give them mentors. So the business is in the city of London… They find business mentors for these young people to help them to set up their business, give them loans, microloans … And now there is a group "pride", pride for being elegit... They call it "the elegit"… that now they are legitimate … The only other jobs that they will get with no education and a criminal record is cleaning or manual labour. But for them, the opportunity to set up their business and become entrepreneur, that's a big difference. We need more of that…" (Stakeholder, UK)

Another 'successful initiative' has been identified in Barcelona, with members of the sub-Saharan community who formerly operated as unauthorised street vendors selling, in some cases, counterfeiting goods. This is a cooperative which have received the support of the Barcelona City Council in order to achieve that street vendors organised themselves in a cooperative and settle in a specific place. The project has the aim to sell their products at fairs and markets in order to offer a legal and respectable opportunity to this community, empowering them and giving them skills to keep them out of OC networks. Thus, this is a policy which resulted from the efforts of the municipal government to find solutions to the complex situation of unauthorised street vending in Barcelona trying to cover the social, labour and relational needs of the former street vendors. This initiative has a positive impact creating positive referents and real working opportunities.

One of the members of the cooperative, a young adult from Senegal reflects about this experience highlighting the good support they have received from the City Council. According to him, the City Council promoted this initiative as a way to avoid conflict with both the police and other local businesses, which complained that they were doing an irregular activity -using the streets. He also emphasised that the number of people employed is still low, but that the success of the initiative currently relies on that this is an innovative project, which is enhancing social cohesion among the different groups in the city. Moreover, another positive thing of this cooperative is that, first, it allows them to do what they know to do -selling; and also that it has enabled them to promote a specific line of products (African products), which differentiates them from other local business.

> "What we said was that it was necessary to reduce tension with the police, but that there are tendencies… we had been working as a Senegalese association, like spiritual movements. Because we believe in two things, a professional work and a spiritual work, and that is very important from the Sufi culture; to be able to fulfil yourself you need to know how to serve (…) Spanish people, people from Africa participate in the association, many people… It's to link faith with work. When there was repression and fines the City Council wasn't going to reduce the tension and they said: we're going to do something: so they made the first coop, the sister coop, with the issue of scrap. At that moment I was linked as a volunteer. Then DIOMCOP was born, to give a way out to the people who were doing street vending. I think that none of both projects are successful for the moment, their success will depend on them being instruments for the community: on them being instruments of cohesion, training, of growth at those levels… This has not pulled out yet with neither of them, they're innovative projects, that might be helping people to at least have an opportunity… with their limitations… Because here we're talking about 350 people on street vending in high seasons (…) The other way out has been: instead of giving them documentation, with a classic company, through occupational plans, the ones in DIOM, have chosen to do what they know to do: sell. To defend their product. Businessmen are saying: what are you selling? The same products we are selling. That's why what we are trying to do is to look at the African line, other products… To be competitive in other products, so as not to have conflicts with other products. Civil society, there are other conflicts… It is being studied along the way… (…) Barcelona's City Council, due to the Barcelona trademark, does not want there to be street vending, but at the same time the project itself has limitations, right now there are few people, but with regard to next year there will be more people. But that is part of the solution, it is not the solution. People are still doing scrap… There will still be street vending… It depends on the moment, on whether there's tension. I play the role of the bridge between the collective and the City Council" (End-user, Spain)

b) Evidence-based inclusive education: programmes that tackle early school leaving and promote successful education for all students, adult education and family training

An inclusive education based on existing evidence of what has been identified that works to promote successful education for all and thus preventing early school leaving of those students in at-risk situation. According to multiple stakeholders inclusive education policies are a core protection factor for the prevention of recruitment in OC networks. Framed within this broad action, some programmes have been identified with sound societal impact:

Schools organised as *Learning Communities*:[8]

In Scampia, Palermo, one of the most deprived areas in Europe and also the most affected by Mafia networks, the 'successful educational actions' implemented in the framework of a Learning Community have shown to improve students' attainment in school, and the improvement of the school coexistence.[9] In schools organised as Learning Communities in order to achieve excellent academic results for all importance is given to interactions (not only with the children, but also with the community) and to the participation of family and community members in the regular classrooms (as volunteers) and therefore, in the management of the school. The school is open to the community. In the Learning Community in Scampia, *Interactive Groups* have been implemented, counting with the active participation of family and community members. As expressed by a teacher of the school, the impact of this activity has not only been on students but also on their families and the community, creating positive role models at home while empowering them with high educational and social expectations.

> "We work a lot in the south of Italy to prevent early school leaving. For this reason we started the Learning Community [in the framework of the SEAs4All project[10]]. And it is having a big impact, the engagement of family has been crucial (…) I work in a neighbourhood in which the market of drugs is huge. This is the first neighbourhood in Europe where commercialisation is taking place. And many families live from these families. But what you do in the school in order to reduce early school leaving, it is not the same that what they experience at home. Many of the parents of children in our school are in prison. And many women are doing prison at home because they are commercialising (…)" (End-user, Italy)

> "There is an improvement of both academic success and community participation in the school, in a neighbourhood in which children speak the dialect and they have to face the National tests. But we yet need to improve academic results. A good training and family education can be a precursor to improve (…) With the Interactive Groups we have many

---

[8] More information about the schools as 'Learning Communities' can be consulted in the following link: http://utopiadream.info/ca/presentacion/definicion/.

[9] For more information, visit the Seas4All project: http://seas4all.eu/.

[10] For more information on the SEAs4All Project see the website http://seas4all.eu

> examples of this. The active participation of the families in the practice and didactic practice of the IG" [This is positive] (End-user, Italy)

The good results achieved in a short period of time and the improvement of coexistence were reasons why this school was on the Italian national TV.

In the UK, as in other countries, one of the stakeholders reflected about the very negative consequences in the lives of youth from deprived areas of having experienced educational segregation since very early ages. Exclusionary education expels youth from the formal educational schemes, thus leaving them unprotected in front of potential risks of falling into organised crime networks:

> "The ones who are engaged with the gang teams are not in education, they might be in a "Pupil Referral Unit" (this is an alternative educational provision) -actually some people call these units "prisons referral units", as after these referrals you may end-up in prisons [Joking about the fact that there is the assumption that after going through educational segregation you have major odds of ending up in prison]" (Stakeholder, UK)

A parallel example provides a Spanish stakeholder (participated in a focus group in which the topic discussed was youth who are detained in juvenile detention centres and there their involvement in organised crime networks), who insisted that educational segregation in many cases occurs even in kindergarten, what marks the life trajectory of many youth:

> "Some of them, as they are out of the system and with almost any opportunity, will end-up involved in OC groups and later in prison (…) we work with those who have passed through all the social reintegration circuits but which have failed, we are in the last stage". (Stakeholder, Spain)

Participants of this focus groups, which were teachers and social workers from a juvenile detention centre, and a worker from the Unit for Children and Adolescent Attention, agreed that segregation has a tremendous impact on those youths who are in these centres. Again, when they were asked about at what point the system has failed, they agreed on answering that it was in the exclusionary education.

c) Programmes oriented to learn the country's language as a 'protective factor'

Several interviewees (stakeholders and end-users with a migrant background) have positively valued those home-language programmes targeted to migrants. According to them, immigrant adults need English for daily life, since participating in the social and public spaces of the community, to communicating in the schools, with the doctor, and indeed being able of accessing the labour market. Knowing the host-country language is definitely a 'protective factor' in front of the vulnerabilities that can lead to be recruited in OC networks.

For the case of the Roma migrants in some cities from the UK (for instance, Coventry), interviewees mentioned that learning English has made a huge difference in their living conditions in comparison with other groups of Roma in other British cities. Stakeholders from the 'Roma Project' in Coventry explained that the mastery of the language in the case of this group has protected them from being deceived and exploited by others.

d) Multicultural and diverse spaces of dialogue and interaction at the very grassroots level that promote positive role models (rejecting violent behaviours) and stories of success

This has been acknowledged in different initiatives and highlighted as of major importance, but when it comes accompanied with interactions with persons from the community that are positive role models for others (in the case of migrant communities, and also in the case of ethnic minorities or in general vulnerable groups) due to the 'stories of success' that they can represent. Prevention in recruitment again, needs to be done at the very grassroots level with the participation of all social agents. It is a 'bystander intervention', which needs to have a clear position against illicit activities, while also presenting alternatives for the improvement of the living conditions of those who are in a more vulnerable situation.

> "We need to work in those lines of common work, cooperative, to break frontiers and I repeat again, the ones in the bottom. Because if there is one thing that brings all of us in the bottom together, it is scarcity at all levels. That among the ones in the bottom there are people who also have a job (…) (End-user, Spain)

A stakeholder, social worker at the Unit of Social Services working in one of the most ethnically diverse neighbourhoods of the second largest city of Catalonia, explains how neighbours themselves have the capacity of solving problems when they emerge, but in order for this to occur, the neighbourhood needs to be organised. The difference between neighbourhoods of big cities which have similar socio-economic conditions, and in some of them there are high levels of criminality, according to stakeholders, is influenced by the role that an 'organised neighbourhood' can function as a 'protective factor'.

> "As administration we weren't dedicated to promoting the neighbourhood identity instead of the citizenship. Citizenship is a judicial and legal position that comes recognised, or not, from the undocumented ones and the ones who have documents. However, the one of the neighbourhood, which is the one we should all strengthen are those proximity relationships that enrich us and generate conflict but which are solved in a very close field, domestic, on the floor, on the square, which is where they act in the best way. The identities of the citizenship that are not being respected need to be changed" (Stakeholder, Spain)

> "There was a block where there were 10 families here and 10 families here. And there was a fight that Roma and Pakistanis had come to argue. The water goes into one apartment from the third floor, from one from the fifth, the water skipped one floor/flat. It was a tube that was broken.

> We had to sue each other there and all that in order to get into the flat. A disaster. I talked to the Roma and I talked to the Pakistani. Eleven Roma and nine Pakistani. You couldn't get into the block there. And I say, let's take a president from the Pakistani and I take a president from the Roma. And we make the Roma president and the Pakistani president and let them find their way and pay each 15-20 € whatever and each of them finds their way. I came to take them and now it's a model floor. They all get along well. And there's a friendship. (End-user, Spain)

2. Support those shops and businesses at the local level which refuse to pay the extortion money

This action has been identified in associations and organisations working in Italy, who are to some extent challenging mafia at the local level. Here we will analyse the example of Addiopizzo.

Addiopizzo NGO: [11] Addiopizzo is a movement that emerged in Palermo, Sicily, in 2010, that works to create a society free from paying the "pizzo" (extortion money) to the mafia. They initially developed a consumer campaign focused to support shops and business which refused to pay the pizzo, publically recognising those who joined the initiative giving them the Addiopizzo sticker. This was to show "I don't back the mafia" meanwhile it encouraged consumers to shop in these shops. In less than 7 years more than 1000 shop have joined them and more than 184 schools are involved in anti-racketeering training.

> "We fight protection money, saying: we are aware that protection money is slavery and the entire community is accepting to be ruled by the mafia. When we started most of the shops were paying so we suggested that the consumers are paying to the mafia when they accept to live in a town where most of the shops pay the mafia. So we first developed a cultural approach by then we developed an economic approach: we developed an ethical consumer campaign… Focusing to support those shops, which rebel against the mafia and do not pay. Basically we started recruiting a list of shops, which are out of the mafia, and we encouraged the travellers to those towns to support these shops." (Sakeholder, Italy)

This is being effective in so far it tackles the demand. Besides this, it is worth mentioning that one of the most successful activities that this NGO is carrying out is the educational one, as defeating mafia in Sicily is much related to "creating the conditions to keep young people away from the mafia hands" (Stakeholder of Addiopizzo). It is for this reason that Addiopizzo volunteers develop cultural and educational activities collaborating with the local authorities, the schools, the church, and especially carrying out its activities in the most deprived areas of the city. For instance, the interviewee explained that one of the activities developed was the "Addiopizzo card", based on a crowd funding strategy. All the money collected was spent on a project implemented in one of the most deprived areas of the city, which was at high

---

[11] For further information about Addipizzo see: http://www.addiopizzo.org/

risk of being controlled by the mafia leaders. For this project the very neighbours decided which were the building priorities that had to be addressed. They decided to construct a community area in the neighbourhood, a playground for the kids and a new gym in the school. The importance of this relied on showing the community that collective money could have a collective benefit, meanwhile, mafia money tried to keep people under poverty. This initiative would have the positive effect of promoting social cohesion in the neighbourhood through these activities as well as education in the field of mafia to prevent the recruitment. These types of programmes should be studied in further detail in order to identify which are those elements that make them successful and thus to be able of transferring them to other contexts, such as the 'successful actions'.

3. Programmes and actions about 'healthy relationships' challenging the association between violence and attractiveness as a way to prevent sexual exploitation and falling in organised crime activities

In line with what was explained in the exclusionary section, related to the negative consequence of not taking into account the existence of a violent model of socialisation in youth, in the UK some organisations have been identified that already include in their tasks programmes about what are and what are not 'healthy relationships'. Such initiatives have a positive impact on contributing to the recovery process, especially of girls and young women who have been victims of sexual exploitation.

> "When we first started to work with young women we had an evaluation questionnaire, so the initial evaluation which we do at various points of the work to measure the journey that the young girl is on. One of the questions we asked them is "How many friends you have when you are in serious trouble and call upon when you are in trouble?". And girls would say "ooh, 10.. 8… However, many they have. Three months later when we are doing this same questionnaire again and we ask the same question, often the answer is "None". And that is an indication for us about if they realise that these friendship were actually not appropriate friendship (…) From that point on we work with them on a range of things like identifying who are the people in their life in whom they have interest, how to nurture the relationships with those people and build strong bonds with those people and family members (…)" (Stakeholder, UK)

> "Girls are socialised in violence and they become desensitised to violence so they expect that this is part of their relationships. So, learning about what is a healthy relationship, is understanding that violence is not part of a healthy relationship (…)" (Stakeholder, UK)

As observed, a key element that this programme fosters is also the critical thinking and reflecting among end-users about who are friends and who are not, as they play a capital role in recruitment to OC networks.

4. Use of peers as role models that reject violence in mentoring programmes and other programmes oriented to prevention of those in

more vulnerable situations to be recruited in organised crime activity, by thinking critically.

There is evidence of the effectiveness of using peers that reject violence as role models in educational programmes and mentoring programmes to both increase academic expectations and empower those in most-disadvantage situations to think critically about risk situations.

Associations working in Romania oriented to widening the educational opportunities of those in disadvantage situations and prevent them from being victims of organised crime constitute an example. For instance, the Ofensiva Tinerlor Asociata: *an Association dedicated to offering young people alternative educational opportunities by involving them in non-formal learning activities. The association is working with and for people belonging to different backgrounds for promoting cultural diversity, responsibility in the community and decisional transparency in public administration.* [12]

The association runs workshops and training aimed at promoting diversity and inclusion, and self-development, in order to generate spaces of exchange between diverse students to avoid discrimination and promote tolerance. The workshops, implemented in the most deprived areas, have the aim to find a motivation and raise expectations between students from a disadvantaged background. They have widely worked with the Roma community.

Besides, stakeholders who participated in the focus groups conducted in Spain (in which users from migrant and ethnic background participated), also pointed out the relevance of promoting peers as role model:

> "[A male Roma adult community agent who has been working as mediator in the neighbourhood, talking about the good results that schools organised as Learning Communities have] I'm going to give you an example, you get to the education department, well look you need to go to San Cosme to the San Cosme and Sant Demián school. The kids finish sixth grade and have to go to do secondary school. And there is a tremendous disorientation. All of them are Roma except for one. Let's see if you can. Well, the truth is I went very confused as well. I go there. The tutor was there. She didn't answer to me any of the three days I went there. Anyway, I go to talk to the kids. Hey, well the question adults have, what do you want to do when you grow up. And one of them tells me he wants to go to the street market with his father. I'll go to the construction because you make cash there. I'll go as a porter here to the airport. Another one told me that I, my uncle sells drugs… Well, you know, the kids. Then I explain to them, there are things. I asked the non-Roma and he did like this, I don't know. It was a boy. What do I know. The second day I told them look there are things you said that are not ok. Like selling drugs and this is not ok, but going to the street market going to work to the airport as a porter well, they're normal things but the world doesn't end there, there is more. And he starts

[12] To see more information about Ofensive Tinerlor Asociate, see: https://europa.eu/youth/volunteering/organisation/947386294_tr.

> explaining that I am coming back from university, what do you think. And he/she starts explaining to them. He/she starts explaining to them and a girl, I still remember, she was called Luisa. And she comes and touches me. What's wrong? And she says 'you are Roma'. – 'Yes. Same as you.' And she says 'you talk very weird' (laughter). Well, I started to tell them the reasons, many. The third day I continue good morning. And we start, of course I ask the kids again, and look, you know what? 'What is that thing about university, that's where the non-Roma with a hat go'. – 'Well yes, that's it, but they don't do that here. The thing about the hat.' I told them, 'I want to be a nurse', 'I want to be a lawyer, that way I have my niece in prison and I get her out'. Well, the thinking had changed a little bit. The one who spoke about stealing and drugs said 'I don't want to study nor work but I don't want to sell drugs either'. Well at least we have made an important step. High expectations had been generated. (End-user, Spain)

In a similar line, another end-user, a young adult Pakistani who is currently studying in university (he accessed as an adult) explains about the work that the Association of Pakistani Students develop among the Pakistani community:

> "Basically I told you earlier in the Pakistani Student Association, what is the base work, we know the institutional problem there is, there are ghetto schools, there are aggregation schools and we go to a school they tell you look you can see a boy who is bad and has a car but well and there is also another boy or another girl who especially with effort, having the same problems or sometimes less, has come out of these problems and has improved herself and is now studying medicine and will be or is already a doctor and to provide positive role models to our community to the youngest ones, we are focusing a lot on that work right now in order to break a little bit and work what is personal but solve we will go on complaining of course they will listen (…)" (End-user, Spain)

5. Campaigns that challenge the recruitment in criminal activities acknowledging their 'fake' attractiveness, while launching alternative messages.

Social media campaigns have been identified that are oriented to prevent those more vulnerable to fall into networks of trafficking of human beings and sexual exploitation which present features that allow them to tackle and question the 'glamour' of the risks posed by some OC groups (OC groups of which their recruitment methods revolve around presenting themselves as an option in which youth will have a promising future):

For instance, the eLiberare Association, which works on the prevention of Trafficking of Human Beings, explained the "Bani pe Bune campaign"[13]:

---

[13] See more information about this campaign in the following link:

> "Bani pe Bune campaign is a recruitment site for fake jobs with the purpose of informing people of the dangers of jobs that look "too good to be true". For the campaign we have flyers that look like 100 euros on one side, and on the other side is the advertisement "Do you want to make a 100 euros per day?" website (www.banipebune.ro).
>
> Once a person gets on the website, he or she is encouraged to apply for employment online involving many benefits. Once the participant applies, a video on human trafficking in Romania appears as well as statistics on the website: http://banipebune.ro/aplica/" [this is an extract from the Associations' website]

The successful aspect of this type of campaigns are that they are oriented to unveil the 'fake attractiveness', which is promoted around the illicit offence. This can be also understood with the case of Addiopizzo (already explained) and its campaign about the "Pizzo free shops": it shows as attractive those shops that are overtly positioned against mafia, and which condemn the illicit activity.

6. Relevance of promoting compulsory education programmes in juvenile detention centres

Within the field of prisons, several stakeholders mentioned programmes that are focused on strengthening and encouraging the 'protective factors' in those who have been involved in OC networks, at the prison context. In cases of juvenile detention centres, promoting compulsory education programmes and encourage youth to participate in them has been identified as oriented to both, higher the socio-economic opportunities of reincorporation to society as well as promoting the identification of 'positive and protective interactions'.

7. Within the field of prisons, actions that tackle the mental health needs of organised crime offenders

Programmes developed in prisons, which attend the mental health needs of offenders are needed in order to better prepare end-users for their way out of prisons and prevent relapse. These programmes have a direct societal impact on end-users themselves and indirect societal impact on the broader society. This is also mentioned in the European Security Agenda.

8. Within the field of prisons, vocational training, career development advice and official educational programmes oriented to develop a transition plan prior to a prisoner's release.

Actions oriented to prepare offenders for their way out of prison, encouraging them to take activities of vocational training, career development advices as well as official education have shown to have a major societal impact, according to stakeholders.

In this sense, organisations such as *St Giles Trust* (SGT) (and others) have developed programmes with sound societal impact, such as programmes oriented to reduce the likelihood of prison leavers re-offending by meeting both their physical needs (housing and access to health and benefits services)

and emotional needs (positive support networks and internal narratives) as they transition back into the community. One of the successful programmes that this charity has developed is the "Peer to the Future",[14] which aimed at improving the lives of offenders, both inside and outside prison. As mentioned by the stakeholder of this organisation, a key issue of the programmes is the inclusion of former offenders in the delivery of the programmes:

> "The multiplier effect is an important feature of this work: ex-offenders are recruited to provide advice to their peers, bringing simultaneous benefits to both groups. Peer advisors have lived and breathed what their clients are experiencing and, with professional training, they can engage them on equal terms as effective agents of change (…) the SGT model of combining the use of Peer Advisors with delivering a "through the gates" service". (Stakeholder, UK)

> "[as an organisation that works in prisons, employing former-offenders, the stakeholder explains] Advice working in prisons! Would you give me a job in the community? We have never recruited someone who had been in prison… So we were like, let's try! And what we thought was a work very ideal for other ex-prisoners to do: these workers need to meet other prisoners at the prisons gate: if there is one person who understands what it is to be in prison, it is a former prisoner (…) So it consists in meeting them "at the gate of the prison": if there is something that our Housing Team that were working in prisons knew for years is that in prisons you need to organise many things: social services, if you have drug issues … the methadone prescription… etc. etc.… because intervening at the right moment, at the prisons gates, you just save them from a week after when the prisoner is released, he coming to ask for the service in a very bad condition… (…) So we needed people out of the gates, and ex-offenders were brilliant doing this: because they understood the motivations and feelings of other offenders" (Stakeholder, UK)

For those inmates that are involved in vocational training or other similar activities, this can be positive and accelerate their access to probation:

> "So with many judges, if they hear that the offender, the gang, has had a good behaviour (for instance, that social workers explain that in the last weeks they have been visiting them, they have been involved in good tasks, etc. etc.…) they remove the sentence….. They negotiate community sentence instead of custodian sentences for young people, buy using ankle tag…. Especially for young people involved in county lines gangs (…)" Although Britain is still very bad, it has improved in relation to youth prison… (Stakeholder, UK)

Finally, the following table summarises the evidence-based elements identified in the results, which advance the societal and ethical impact of OC policies.

---

[14] For more information see: http://stgilestrust.org.uk/misc/Evaluation%20into%20peer-led%20resettlement%20support%20in%20Leeds%20full%20report.pdf.

TABLE 1. Elements that advance the societal and ethical impact of OC policies

**Elements that advance the societal and ethical impact of OC policies**
1. Equipping vulnerable people with a set of educational and employment skills and a network of 'protective' community agents, creating links of solidarity, which prevent them to fall in OC networks
1.1. Active labour market policies and other programmes oriented to creating employment opportunities for those at risk of getting involved in gang crime or mafia-type activities.
1.2. Evidence-based inclusive education: programmes that tackle early school leaving and promote successful education for all students, adult education and family training
1.3. Programmes oriented to learn the country's language as a 'protective factor'
1.4. Multicultural and diverse spaces of dialogue and interaction at the very grassroots level that promote positive role models (rejecting violent behaviours) and stories of success
2. Support those shops and businesses at the local level which refuse to pay the extortion money
3. Programmes and actions about 'healthy relationships' challenging the association between violence and attractiveness as a way to preventing sexual exploitation and falling in organised crime activities
4. Use of peers as role models that reject violence in mentoring programmes and other programmes oriented to prevention of those in more vulnerable situations to be recruited in organised crime activity, by thinking critically.
5. Campaigns that challenge the recruitment in criminal activities acknowledging their 'fake' attractiveness, while launching alternative messages.
6. Relevance of promoting compulsory education programmes in juvenile detention centres
7. *Within the field of prisons, a*ctions that tackle the mental health needs of organised crime offenders.
8. *Within the field of prisons, v*ocational training, career development advice and official educational programmes oriented to develop a transition plan prior to a prisoner's release

2.3 Contributions to inform the operationalisation of the input for ABM simulations

The following table gathers the factors identified as relevant in the recruitment into OC and TN; they inform the operationalisation of the input for the ABM simulations.

TABLE 2. Contributions to start the operationalisation of the input for ABM simulations

**Contributions to start the operationalisation of the input for ABM simulations**	
1	The more policies encourage and support those not paying extortion money = the less income for the mafias.
2	The more policies support job opportunities targeted to those at risk (based on school drop out, low attendance, low income, health and not only migrant profile) = less risk factors
3	The more active labour market polices and other employment opportunities which capitalise the entrepreneurial skills and leadership capacity of those young people who have been involved or are in the process of engaging in organised crime networks = major social impact
4	The less glamour is put on the narrative discourses about violence in the campaigns against OC and TN = less popularity among youth
5	The more policies focused on evidence-based inclusive education = less risk factors
6	The more training on critical thinking which includes unveiling attraction to violence (=) more success in rejecting fake jobs, promises related to OC or violent extremism
7	The more obligation on coordinating actions among different professionals working in the same territory (+) and better defined competences of each of them (=) the better are chances for effective implementation of their work)
8	The stronger the network of 'protective' community agents (+) the higher the links of solidarity (truly trust) (=) more role models for rejecting OC and TN
9	The better the language skills, the better inclusion and deeper engagement with the community
10	The more programmes that promote 'healthy relationships' challenging the association between violence and attractiveness, the weaker are pull factors
11	The more critical thinking about who are friends and who are not, as they play a capital role in recruitment to OC and TN, the less attraction to them
12	No policies oriented to resocialisation and integration means, more risk situations for human rights abuse
13	The more focus on only migrant profiles (=) more risk situations for human rights abuse
14	The less stigmatising or criminalising ethnic minorities (+) higher counts on their collaboration (=) more in favour of/more support of stop and search prevention
15	The more policies identifying second order of harassment of the families and people working in prevention or deradicalisation (+) more bystander intervention = higher support from community members and professionals
16	The more policies that promote interfaith dialogue and cooperation among religious leaders = less radicalisation/disengagement from "real" religion
17	The more prevention and deradicalisation strategies developed in the community and with the community fostering counter-narratives that do not glamourise, but reject violence and radicalisation = less radicalisation
18	The more focus given to critical thinking based on the identification of real friendship and the dominant discourse on violent masculinities = less pull factors
19	The more policies based on healthy relationships (+) less mirage of upward mobility among girls / victims of grooming and therefore = less pull factors
20	The more support to grassroots movements to counteract violent extremist positions = less radicalisation

3 FINAL REMARKS

Task 1.2. has focused on analysing the societal and ethical impact of some policies that fight against organised crime. The communicative methodology used to achieve such ambitious endeavour has allowed us to identify the negative impacts (the exclusionary dimension), as well as to shed light on the positive impacts (the transformative dimension) of some of these actions and programmes: elements which when taken into account in the design and implementation of the policies are contributing to advance towards the societal and ethical impacts on end-users.

Bearing in mind the study limitations due to the complexity of the task given the general and broad definition of OC in the FD2008/841/JHA (multiple policies among different legal systems that are deployed in the EU member states) and the short-time limit, the work done constitutes a first approach of the impacts of actions, programmes and strategies that are being developed as part of broader policies against OC. In this sense, the added value of the study conducted is that evidence obtained is the result of a direct inter-subjective dialogue with those social agents, which are either designing these policies or/and implementing them, as well as with those people whose lives are being affected by them.

The transformative elements identified illuminate aspects of the OC policies, which when taken into account are advancing towards preventing the recruitment. Nonetheless, the analysis conducted shows that although the EU legislative and non-legislative initiatives to counter-security threats recognise and emphasise the importance of prevention, measures are still largely more oriented to the disruption of the networks.

All in all, several examples of preventive actions and strategies for recruitment that have been proven to work have been identified and discussed. The final Table on ‘Elements that advance the societal and ethical impact of OC policies’ is a summary of them. However, more needs to be done in order to prevent that OC networks exploit the vulnerabilities of those people who are already at risk. Successfully fighting organised crime supposes an enterprise that requires the collaboration of multiple agents, doing both prevention and intervention at different levels, from the subjects’ side (equipping those more vulnerable to not be recruited, protecting their fundamental rights) and at the system level (strengthening law enforcement). Some of the actions identified have already been pointed out as recommendations by existing legislation. Two issues, which are commonly approached as indirect aspects have been showed that need to be tackled directly to enhance prevention at the subject level. First, educational segregation since very early ages, what triggers and maximises peoples’ vulnerabilities. Second, the work on promoting ‘healthy relationships’ and recognising the huge impact that early socialisation in a predominant model of relationships which associates violence with attraction is having as a pull factor that influences on the processes of recruitment, as well as on the perpetuation of this business. To void of “attractiveness” everything that

surrounds OC networks is still a pending task in prevention policies at the subject level.

All in all, studying the societal and ethical impact of policies requires as a must going to the ground through fieldwork analysis, asking the very agents involved in their design and implementation as well as end-users about the social advancements achieved or the ones that have not yet been accomplished. These types of evaluations in order to be fair with the commitment of all those involved in the realisation of their tasks cannot solely rely on methodologies limited to analysis of the content of the policies. This can indeed complement qualitative in-depth fieldwork analyses but at any case replace them. In this sense, as already pointed out by the FP7 IMPACT-EV project and as recognised by SIOR[15], this way of analysing social impact supposes making knowledge open to society, contributing to inform *dialogic evidence-based policies*.

[15] The first Social Impact Open Repository in the world: http://sior.ub.edu/jspui/sior.jsp.

4 REFERENCES

Council of the European Union. (2008). *Council Framework Decision 2008/841/JHA of 24 October 2008 on the fight against organised crime.* Retrieved from: http://eur-lex.europa.eu/eli/dec_framw/2008/841/oj.

Di Nicola, A., Gounev, P., Levi, M., Rubin, J., Vettori, B., Baratto, G., Betgens, M. Bezlov, T., Bressan, S., Constantino, F., Cauduro, A., Disley, E., Faion, M., Ilcheva, M., Kojouharov, A. Pardal, M., Terenghi, F., & Tzvetkova, M. (2015). *Study on paving the way for future policy initiatives in the field of fight against organised crime: the effectiveness of specific criminal law measures targeting organised crime*. Luxemburg: Publications Office of the European Union. Retrieved from: http://bookshop.europa.eu/en/study-on-paving-the-way-for-future-policy-initiatives-in-the-field-of-fight-against-organised-crime-pbHR0614242/

European Commission (2017). TOOL 19. "Guidelines for a Better Regulation". Toolbox complements the better regulation guidelines presented in SWD(2017)350. Retrieved from: https://ec.europa.eu/info/sites/info/files/better-regulation-toolbox.pdf

European Commission. (2016). *Report from the Commission to the European Parliament and the Council based on Article 10 of Council FD 2008/841/JHA, COM(2016) 448 final.* Retrieved from https://ec.europa.eu/home-affairs/sites/homeaffairs/files/e-library/documents/policies/organized-crime-and-human-trafficking/general/docs/report_on_the_fight_against_organised_crime_20160707_en.pdf

European Union (2011). Directive 2011/36/eu of the European Parliament and of the Council on preventing and combating trafficking in human beings and protecting its victims, and replacing Council Framework Decision 2002/629/JHA Retrieved from https://ec.europa.eu/anti-trafficking/legislation-and-case-law-eu-legislation-criminal-law/directive-201136eu_en

Flecha, R. (2014). Using Mixed Methods From a Communicative Orientation: Researching With Grassroots Roma. *Journal of Mixed Methods Research*, *8*(3), 245–254. doi:10.1177/1558689814527945

Flecha, R. (2014-2017). IMPACT-EV. Evaluating the impact and outcomes of European SSH Research. European Commission, 7th Framework Programme.

Gómez, A., Puigvert, L., & Flecha, R. (2011). Critical Communicative Methodology: Informing Real Social Transformation through Research. *Qualitative Inquiry, 17*(3), 235–245. doi:10.1177/1077800410397802

GOV.UK (2013). *Serious and Organised Crime Strategy*. Policy Paper. Home Office and The Rt Hon Norman Baker. Retrieved from: https://www.gov.uk/government/publications/serious-organised-crime-strategy

Habermas, J. (1984) [1981]. *Theory of Communicative Action*, Volume One: *Reason and the Rationalization of Society*. Boston, Massachusetts: Beacon Press. ISBN 978-0-8070-1507-0.

Mitsilegas, V. (2011). The Council Framework Decision on the Fight against Organised Crime: What can be done to strengthen EU legislation in the field? European Parliament. Directorate General For Internal Policies. Retrieved from http://www.europarl.europa.eu/RegData/etudes/note/join/2011/453195/IPOL-LIBE_NT(2011)453195_EN.pdf

National Crime Agency [NCA] (2016). *County Lines Gang Violence, Exploitation & Drug Supply.* 0346-CAD National Briefing Report. Drug Threat Team, Organised Crime Command. Retrieved from: http://www.nationalcrimeagency.gov.uk/publications/753-county-lines-gang-violence-exploitation-and-drug-supply-2016/file

Reed, M. (2017). *The Children's Society responds to the NCA report on "county lines" exploitation.* Press releases, Children Society News and Blogs. Retrieved from: https://www.childrenssociety.org.uk/news-and-blogs/press-releases/the-childrens-society-responds-to-the-nca-report-on-county-lines

Sordé-Martí, T. & Mertens, D. M. (2014). Mixed Methods Research With Groups at Risk New Developments and Key Debates. *Journal of Mixed Methods Research*, *8*(3), 207–211. doi:10.1177/1558689814527916

ANNEX I. DEFINITION AND EUROPEAN LEGISLATION ON ORGANISED CRIME

DEFINITION

The first time that the EU criminalized a criminal organization was in 1998 with the adoption of **Joint Action 98/733/JHA** making it a criminal offence to participate in a criminal organisation in the Member States of the European Union. This first step was followed by the United Nations Convention against Transnational Organised Crime (UNTOC, hereinafter) becoming the main international instrument in the fight against transnational organised crime. However, in 2005 the Commission decided to create a framework decision on the fight against organised crime providing a consistent instrument to tackle OC in the EU and which repealed the Joint Action 98/733/JHA. Nevertheless, it seems that this Framework decision did not achieve the expectations and the Commission decided to issue a declaration questioning the value:

- The framework decision does not achieve the minimum degree of approximation of acts of directing or participating in a criminal organization on the basis of a single concept of such an organisation.
- The framework decision enables Member States not to introduce the concept of criminal organisation but to continue to apply existing national criminal law by having recourse to general rules on participation and preparation of specific offences.

Other authors, such as Calderoni (2012) have affirmed also the problems of the Framework Decision 2008/841/JHA. The first issue is the very limited description of the typical features of organized crime, which is too vague to comply with the general principles of criminal law. Secondly, the quantitative selection of the predicate offences of a criminal organization without taking into account that sanctioning policies among different legal systems can vary significantly. Finally, the double model offence.

The FD 2008/841/JHA in the article 1 adopted the definition of organised crime with a double model offence.

1. *'criminal organisation' means a structured association, established over a period of time, of more than two persons acting in concert with a view to committing offences which are punishable by deprivation of liberty or a detention order of a maximum of at least four years or a more serious penalty, to obtain, directly or indirectly, a financial or other material benefit;*
2. *'structured association' means an association that is not randomly formed for the immediate commission of an offence, nor does it need to have formally defined roles for its members, continuity of its membership, or a developed structure.*

In the article 2 the FD 2008/841/JHA collects the offences relating to participation in a criminal organisation:
Each Member State shall take the necessary measures to ensure that one or both of the following types of conduct related to a criminal organisation are regarded as offences:

a) *conduct by any person who, with intent and with knowledge of either the aim and general activity of the criminal organisation or its intention to commit the offences in question, actively takes part in the organisation's criminal activities, including the provision of information or material means, the recruitment of new members and all forms of financing of its activities, knowing that such participation will contribute to the achievement of the organisation's criminal activities;*
b) *conduct by any person consisting in an agreement with one or more persons that an activity should be pursued, which if carried out, would amount to the commission of offences referred to in Article 1, even if that person does not take part in the actual execution of the activity*.

Considering that this is the main legal framework, there are three main approaches to the criminalization of organized crime among the Member States of the EU and each approach developed a different solution to criminal organization offences. First, the civil law approach based on the concept of criminal association or criminal organization. In general, it criminalises the participation in a criminal association. Second, the common law approach is based on the offence of conspiracy (as the agreement to commit a crime, and the concept and proof of the agreement is its main element). Finally, the Scandinavian approach has been based on the rejection of Criminal Organization Offences and relying instead on the general provisions of criminal law (complicity, aiding and abetting).

The Report from the Commission to the European Parliament and the Council based on Article 10 of Council FD 2008/841/JHA provides an assessment of the relevant national provisions of Member States that base their systems on a self-standing offence. In this assessment, the report evaluates which country is fulfilling the provisions of the FD 2008/481/JHA. However, the overview does not cover Denmark, Sweden and United Kingdom, only the remaining 28 Member States. It is also important to stress that articles from 1 to 8 require implementation, while articles 9 to 12 do not require. This report is so useful to evaluate how the EU members are implementing the TOC legislation.

The article 83 of the Lisbon treaty defines the called *Eurocrimes*, affirming that:

> *The European Parliament and the Council may, by means of directives adopted in accordance with the ordinary legislative procedure, establish minimum rules concerning the definition of criminal offences and sanctions in the areas of particularly serious crime with a cross-border dimension resulting from the nature or impact of such offences or from a special need to combat them on a common basis.*

> *These areas of crime are the following: terrorism, trafficking in human beings and sexual exploitation of women and children, illicit drug trafficking, illicit arms trafficking, money laundering, corruption, counterfeiting of means of payment, computer crime and* ***organised crime****. On the basis of developments in crime, the Council may adopt a decision identifying other areas of crime that meet the criteria specified in this paragraph. It shall act unanimously after obtaining the consent of the European Parliament.*

SOCTA (Serious and Organized Crime Threat Assessment)[16] (2017) also adopts the definition of organised crime by the EC, although at the same time they recognize that this definition does not work.

> *"Since the year 2000, the United Nations Convention against Transnational Organized Crime has provided an internationally shared definition of an organised criminal group as "a group of three or more persons existing over a period of time acting in concert with the aim of committing crimes for financial or material benefit." This definition was also adopted in the EU's Council Framework Decision 2008/841/JHA of 24 October 2008 on the fight against organised crime and continues to reflect law enforcement authorities' conceptualisation of organized crime across the world. However, this definition does not adequately describe the complex and flexible nature of modern organised crime networks".*

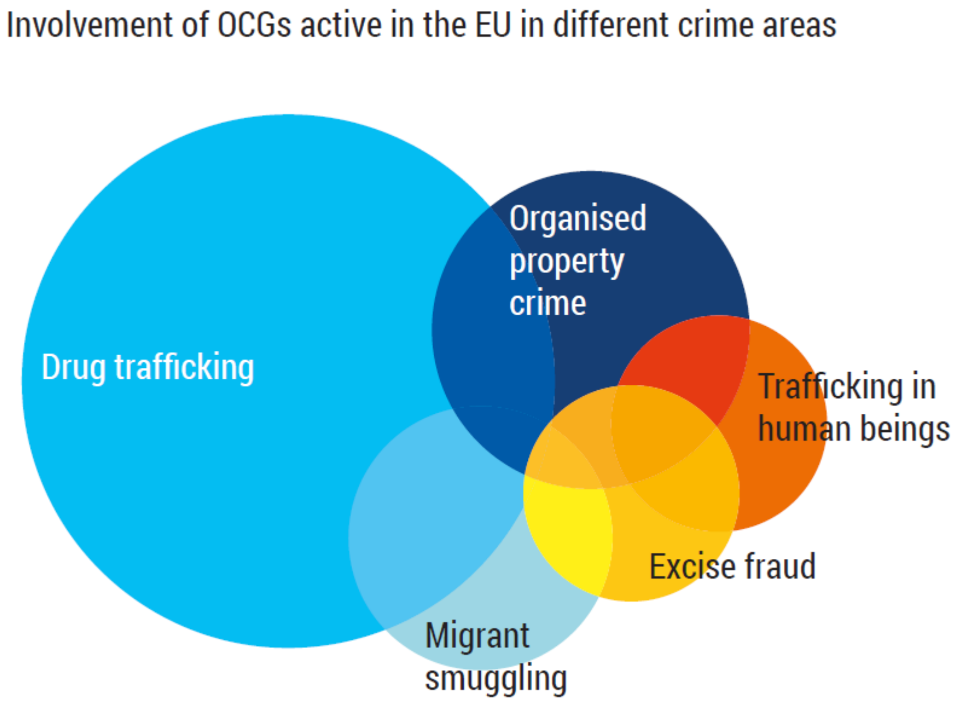

[16] For further information, see: https://www.europol.europa.eu/activities-services/main-reports/serious-and-organised-crime-threat-assessment.

These are the main areas involved in OC in the EU. However, SOCTA remarks that 45% of the OCGs are involved in more than one criminal activity (SOCTA, 2017[17]).

Europol affirms that: serious and organised crime is an increasingly dynamic and complex phenomenon that requires robust, intelligence-led response by EU law enforcement. While traditional crime areas such as international drug trafficking remain a principal cause of concern, the effects of globalisation in society and business have facilitated the emergence of significant new variations in criminal activity. Criminal networks exploit legislative loopholes, the internet and conditions associated with the economic crisis to generate illicit profits at low risk, for example. One of Europol's flagship reports, the Serious and Organised Crime Threat Assessment (SOCTA) updates Europe's law enforcement community and decision-makers on such developments in serious and organised crime and the threats it poses to the EU. Informed by its analysis of the prevailing threats, the SOCTA identifies a number of high priority crime areas that the operational response in the EU should focus on.

Europol's current SOCTA, published in 2017, identifies the following eight priority crime threats: cybercrime, drug production, trafficking and distribution, migrant smuggling, organised property crime, trafficking in human beings, criminal finances and money laundering, document fraud and online trade in illicit goods and services.

EUROPEAN LEGISLATION

The EU has been elaborating action plans, programmes and strategies either targeting organised crime in general or dealing with its particular forms. The problem has been addressed within the EU's foreign policy and accession strategy, but most of the relevant actions have been undertaken as part of police and judicial cooperation in criminal matters (EU Response to Organised Crime, 2013).

a) European Agenda on Security

The most recent and updated European Security Strategy, called European Agenda on Security COM(2015) 185 final (EAS hereinafter) reaffirms the Internal Security Strategy 2010-2014, where the main concerns are: terrorism, organised crime and Cyber security. Moreover, in this document are gathered the latest mechanism to counter terrorism and also Organised Crime and Cybersecurity. The main actions appointed by the EAS 2015 are extending the work of the EU Policy Cycle to neighbouring countries; reviewing possible measures for non-conviction based confiscation; reviewing legislation on firearms with proposals in 2016; adopting a post-2016 strategy on human trafficking; launching joint actions and cooperation strategies with key third countries to combat smuggling of migrants; reviewing existing policy and legislation on environmental crime, for proposals in 2016.

---

[17] For further information, see: https://www.europol.europa.eu/socta/2017/.

b) The EU policy cycle to tackle organised and serious international crime

The policy cycle is a methodology adopted in 2010 by the European Union to address the most important criminal threats affecting the EU. Each cycle lasts four years and optimises coordination and cooperation on chosen crime priorities. The criminal threats are identified on the basis of criminal intelligence and then agreed at political level. During the cycle, all concerned services and stakeholders, at national and EU level, are invited to allocate resources and mutually reinforce efforts. Emerging threats are also monitored so that they can effectively be responded to.

This methodology considers four different steps. The first step is the Serious and Organised Crime Threat Assessment (SOCTA) delivered by EUROPOL on data from member states' law enforcement authorities among other authorities. The SOCTA provides an analysis of the present and future threats posed by serious and organised crime to the EU and a set of crime priorities.

Second, it provides a choice of crime priorities and multi-annual strategic plans (MASPs). It is the Standing Committee on operational cooperation on internal Security (COSI) who examines the SOCTA priorities and together with other commissions and agencies elaborates a 4-year strategic plan.

Thirdly, the MASPs are implemented by Operational Action Plans (OAPs) one per priority and per year. The OAPs include joint member states/agencies actions, but also agencies actions and national actions. The joint actions are carried out as EMPACT (European Multidisciplinary Platform Against Criminal Threats) projects.

Finally, there is a Commission review and interim assessment to adapt and modify the MASPs or the priorities if necessary. There is also an independent evaluation at the end of the policy cycle. EUROPOL mentioned that this evaluation was in progress and there is no information if it will be public or private, but it would be a good source for the impact. According to the draft Council conclusions on the continuation of the EU policy cycle for OC 2018-2021 this document is already finished, but it is EU restricted.

Moreover, there is no public access to MASPs and OAPs documents. Some of them are partially public or private. Therefore, it is only possible to know the general strategy of the EU, but not the particular plans.

The EMPACT projects according to the SOCTA2013 are: facilitation of illegal immigrations; trafficking in human beings; counterfeit goods; Excise and MTIC fraud; synthetic drugs; cocaine and heroin; illicit firearms trafficking; organised property crime and cybercrime (SOCTA, 2013)[18].

[18] For further information, see: EU Serious and Organised Crime Threat Assessment 2013.

c) The programme on prevention, and fight against crime (ISEC)

ISEC is a programme of the European Commission in the area of Migration and Home Affairs. This programme was inside a general framework called "Security and Safeguarding Liberties (2007-2013) (SSL)", which is composed of the two Programmes the ISEC and "Prevention, Preparedness and Consequence Management of Terrorism and other Security Related Risks (CIPS)". CIPS programme concerns critical infrastructure and other security issues, including operational issues in areas such as crisis management, environment, public health, transport, research and technological development.

ISEC had a budget of EUR 600 million for the period 2007–13 and contributes to citizens' security through projects that prevent and combat crime. Terrorism, human trafficking, child abuse, cybercrime, illicit drug and arms trafficking, corruption and fraud are a particular focus. The programme has four key strands: crime prevention, law enforcement, witness protection and support and victim protection.[19]

This programme considers prevention and intervention, and it includes also terrorism as one of the crimes to tackle.

In the COM(2011)318 final on the mid-term evaluation of the Framework Programme "Security and Safeguarding Liberties" (2007-2013) is stated that the initial priorities of the ISEC programme have been reconfirmed in the Commission's communication of 22 November 2010 on the Internal Security Strategy in Action ("Internal Security Strategy") as part of the future strategic objectives and priority actions in the field of Internal Security.

Overall, the ISEC level of achievement of results is broadly satisfactory, being in line with initial expectations in 60% of projects and better than expected for another 34%.

Examples of ISEC projects:

- **Police cooperation**. A Joint Customs Operation involving several countries led to the seizure of 4.5 tons of cocaine. Another project supported an international operation against child trafficking. The operation is not yet completed but, by mid-2010, 26 of the 90 suspects investigated had already been charged. Similarly, another operation against Eastern European gangs involved in illegal immigration led to the arrest of some 90 people.
- **Mutual understanding:** projects aimed at networking (such as the ATLAS network of European anti-terrorism units and the Financial Investigation Units (FIU) Network) had an important role in increasing the knowledge about the different systems and legal frameworks existing in the various Member States.

[19] For further information, see: https://ec.europa.eu/home-affairs/financing/fundings/security-and-safeguarding-liberties/prevention-of-and-fight-against-crime_en.

- **Cross-border cooperation:** An example of a Joint Investigation Team (JIT) is the "COSPOL FII" project, aimed at addressing irregular migration from Eastern Europe and dismantling the criminal gangs involved.
- **Intelligence and information sharing**
- **Assistance to victims**
- **Stakeholders and policy makers' awareness**: organization of conferences

d) REPORT on the fight against corruption and follow-up of the CRIM resolution (2015/2110(INI)).

This report emphasises the importance to adopt a European Action Plan to eradicate OC, corruption and money laundering. The main covered areas are:

- Correct transposition of existing rules, monitoring their application and assessing whether they are effective.
- Priorities and operational structure for the fight against OC and corruption: for example, combating crimes of association rather than simply combating so-called target crimes, or the creation of a specialist EUROPOL unit.
- A stronger legislative framework: fill any gaps that may exist. For example, draft minimum rules concerning the definition of offences and penalties, etc.
- More effective police and judicial cooperation at EU level: specific actions in this regard among other measures such as: exchange all data deemed necessary and relevant concerning persons convicted of an offence linked to OC.
- Seizing the assets of criminal organisations and facilitating their re-use for social purposes: employing a common method for seizing criminal organizations assets in the EU.
- Preventing OC and corruption from infiltrating the legal economy: for example, with blacklists of any undertakings, which have proven links with OC.
- European Public Prosecutor's Office (EPPO)
- Specific areas requiring action:
    - Counterfeiting
    - Drug trafficking
    - Gambling
    - Tax haven
    - Environmental crimes
    - Cybercrime
    - **Organised crime and terrorism:** growing convergence and nexus between OC and Terrorism
    - Organised crime and HT and smuggling

e) Council conclusions on setting the EU's priorities for the fight against serious and organised crime between 2018 and 2021

THE COUNCIL sets the following priorities, in no particular order, for the fight against serious and organised crime between 2018 and 2021[20]:

---

[20] For further information, see: http://data.consilium.europa.eu/doc/document/ST-8654-2017-INIT/en/pdf.

- To fight cybercrime, by (1) disrupting the criminal activities related to attacks against information systems, particularly those following a Crime-as-a-Service business model and working as enablers for online crime, by (2) combating child sexual abuse and child sexual exploitation, including the production and dissemination of child abuse material, and by (3) targeting criminals involved in fraud and counterfeiting of non-cash means of payment, including large-scale payment card fraud (especially card-not-present fraud), emerging threats to other non-cash means of payment and enabling criminal activities
- To (1) disrupt the activities of Organised Crime Groups (OCGs) involved in the wholesale trafficking of cannabis, cocaine and heroin to the EU, to (2) tackle the criminal networks involved in the trafficking and distribution of multiple types of drugs on EU markets and to (3) reduce the production of synthetic drugs and New Psychoactive Substances (NPS) in the EU and to dismantle OCGs involved in their production, trafficking and distribution.
- To disrupt OCGs who facilitate illegal immigration by providing facilitation services to irregular migrants along the main migratory routes crossing the external border of the EU and within the EU, particularly focussing on those whose methods endanger people's lives, those offering their services online and making use of document fraud as part of their business model.
- To combat organised property crime by concentrating on disrupting highly mobile OCGs carrying out organised thefts and burglaries across the EU. This should include OCGs using new technologies or enhanced countermeasures which exploit the lacking interoperability of cross-border surveillance tools.
- To fight against the trafficking in human beings (THB) in the EU for all forms of exploitation, including sexual and labour exploitation as well as all forms of child trafficking.
- To disrupt the capacity of OCGs and specialists involved in excise fraud and Missing Trader Intra Community (MTIC) fraud.
- To disrupt OCGs involved in the illicit trafficking, distribution and use of firearms.
- To disrupt OCGs involved in environmental crime, more particularly wildlife and illicit waste trafficking.
- To combat criminal finances and money laundering and facilitate asset recovery in view of effectively confiscating the criminal profits of OCGs, especially targeting money laundering syndicates offering money laundering services to other OCGs and those OCGs making extensive use of emerging new payment methods to launder criminal proceeds.
- To combat document fraud in the EU, targeting OCGs involved in the production and provision of fraudulent and false documents to other criminals.

ANNEX II. FIELDWORK STUDY PARTICIPANTS

Fieldwork study participants

Country	Institution	Profile
Germany	University not identified due to confidentiality reasons	Expert, female. Collaborates with FP projects. Expert on terrorism and radicalization
Germany	University not identified due to confidentiality reasons	Expert, male. Head of a research centre related to religious issues
Germany	University not identified due to confidentiality reasons	Expert, male. Managing director of a centre related to Islam and law
Germany	University not identified due to confidentiality reasons	Expert, male. Research interests related to migration, integration and intercultural conflicts
Germany	University not identified due to confidentiality reasons	Expert, male. Director of a centre on police and security research
Germany	University not identified due to confidentiality reasons	Expert, male. Research interest related to Arab studies
Germany	University not identified due to confidentiality reasons	Expert, male. Expert in de-radicalization
Germany	NGO working on violence prevention	Stakeholder, male. Expert on prevention of online radicalization
Germany	Institution working in the field of de-radicalization	Stakeholder, female. Works with families on de-radicalization
Germany	NGO working on street work	Stakeholder, male. Social street worker on violence prevention
Germany	Mothers for Life Association	End-user, female. Lost her son in Syria, member of the association
Germany	Federal Ministry of Interior	Stakeholder, female.
Germany	Kompass	Misbah Arshad, female. Works on prevention of radicalization and deradicalisation in youth detention centres
Germany	Kompass	Mustafa Cimsit, male. Imam and works on prevention of radicalization and deradicalisation in youth detention centres
Germany		End-user, female. Young Muslim participated in prevention programme

Germany	Religious Association	Stakeholder, male. Representative of Muslim associations
Italy	Non-profit research centre not identified due to confidentiality reasons	Expert, male. founder and senior researcher of a research centre. Producer of documentaries for national and international media.
Italy	University not identified due to confidentiality reasons	Expert, male. Researcher involved in Religious Affairs.
Italy	Institute of Research at the National Research Council	Expert, male. His main research interests concern educational organizations, professional learning and educational policies.
Italy	University not identified due to confidentiality reasons	Expert, female. Her research focuses on criminal law.
Italy	University not identified due to confidentiality reasons	Expert, male. His research interests include terrorism and political violence, radicalisation, and international migration and security.
Italy	Addiopizzo	Stakeholder, male. Director. Addiopizzo is one of the most famous association in Sicily to build a community of businesses and consumers who refuse Mafia extortion money
Italy	School not identified due to confidentiality reasons	Stakeholder, female. She is an institute professor.
Italy	Association Antigone	Stakeholder, male. Work in prisons with association for rights and guarantees in the criminal system.
Netherlands	University not identified due to confidentiality reasons	Expert, female. Senior-Researcher and Lecturer at a Dutch university. Expertise in Justice, policy and Media. Formerly involved with responsibilities in Amnesty International Dutch section.
Netherlands	University not identified due to confidentiality reasons	Expert, female. Researcher involved in several European projects. Expertise in communities and neighbourhoods.
Netherlands	University not identified due to confidentiality reasons	Expert, female. Her research focuses on migrations, migration control and terrorism.
Netherlands	University not identified due to confidentiality reasons	Expert, female. Researcher involved in several European projects. Expertise in criminal justice and social work.
Netherlands	Ministry of Justice and Security	Expert, male. Policy maker at Ministry of Justice and Security
Netherlands	Ministry of Justice and Security	Expert, female. Policy maker. Head of unit
Netherlands	City council	Stakeholder, female. Worker in a city council
Netherlands	Dutch National Police	Stakeholder, male. Chief of police district.

Netherlands	Dutch National Police	Stakeholder, female. Responsible of Organized Crime in a police district.
Netherlands	Dutch National Police	Stakeholder, female. Involved in the work of Trafficking on Human Beings
Netherlands	Foundation for Intercultural Participation and Integration (SIPI) in Amsterdam.	Stakeholder, female. Project manager.
Netherlands	Critical Mass Foundation	Stakeholder, female. Project leader
Netherlands	Foundation Peace Education Projects	Stakeholder, male. Responsible of projects development
Netherlands	Any specific institution	End-user, male. He was radicalised and now is de-radicalised.
Romania	University not identified due to confidentiality reasons	Expert, male. He conducts research in the area of probation and prison fields. He works with recognized agencies in relation to criminology.
Romania	University not identified due to confidentiality reasons	Expert, female. Researcher involved in international security affairs.
Romania	Media Wise Society	Stakeholder, female. Involved in media education.
Romania	Active Watch	Stakeholder, male. He works in this NGO specialized in media monitoring and interventions for combating discrimination.
Romania	Asociatia Impotriva Crimei Organizate si Consiliere Antidrog	Stakeholder, male, neighbourhood. This association works to combat violence at the national level.
Romania	Fundatia de Sprijin Comunitar	Stakeholder, male. Responsible of projects. The foundation supports disadvantaged groups of community and development of the local communities.
Romania	Association for Dialogue, Employment and Migration CONECT	Stakeholder, male. President of the association.
Romania	Ofensiva Tinerilor	Stakeholder, male. Member of this association that works with young people belonging to different ethnic groups. He is also parliamentary advisor at the Romanian Parliament.
Romania	Asociatia Eliberare	Stakeholder, female. Director of this association working on human trafficking.
Spain	University not identified due to confidentiality reasons	Expert, male. Researcher in a group on public policies
Spain	University not identified due to confidentiality reasons	Expert, male. Part of the Advisory Council for Religious Diversity of a regional government
Spain	Catalan government/Regional government not identified	Expert, female. Head of security of the penitentiary services of the government

	due to confidentiality reasons	
Spain	University not identified due to confidentiality reasons	Expert, male. Has worked on racism, anti-Semitism and jihadism issues
Spain	University not identified due to confidentiality reasons	Expert, female. Member of an observatory on human rights and the penal system
Spain	Catalan government/regional government not identified due to confidentiality reasons	Expert, male. Head of Commission for the Attention to the Family and the Community in the Catalan Department of Education
Spain	Catalan government/regional government not identified due to confidentiality reasons	Expert, male. Field of rehabilitation of penitentiary services of the government
Spain	Regional TV	Stakeholder, male. Vice-president of the table for diversity of the TV program
Spain	Catalan primary school	End-user/female. Director of a primary school in Spain organized as a Learning Community, located in a deprived neighbourhood.
Spain	Catalan primary school	End-user/female. Teacher of a primary school in Spain organized as a Learning Community, located in a deprived neighbourhood.
Spain	Catalan primary school	End-user/female. Teacher of a primary school in Spain organized as a Learning Community, located in a deprived neighbourhood.
Spain	Jewish synagogue	End-user/male. Rabbi from a Jewish synagogue in Barcelona
Spain	Mosque	End-user/male. Migrant, Imam of a Spanish mosque.
Spain	Roma Association	End-user/male. Roma, community leader
Spain	Roma activist	End-user/male. Roma, community leader
Spain	Pakistani Neighbourhood Association	End-user/male. Migrant, recognized community leader
Spain	Badalona Neighbourhood Association	End-user/male. Activist. President of the Neighbourhood Association
Spain	Department of Social Affairs of Badalona (Consorci Badalona Sud)	End-user/male. Worker in the Department of Social Affairs of Badalona (Consorci Badalona Sud)
Spain	Women’s association	End-user/female. Member of a Women’s association from Badalona
Spain	Juvenile Detention Centre	Stakeholder/female. Social worker and teacher in a Catalan Juvenile Detention Centre
Spain	Juvenile Detention Centre	Stakeholder/female. Responsible of the Education in a Catalan Juvenile Detention Centre

Spain	Unit for Child and Teenagers Attention, Barcelona City Council	Stakeholder/female. Professional from the Unit for Child and Teenagers Attention, Barcelona City Council].
Spain	University	End-user, male. Community leader collaborating with integration issues
Spain	Muslim Students Association	End-user, female. Muslim student, member of the Association of Muslim Students in Tarragona
Spain	Cooperative of Senegalese Workers	End-user, male. Sub-Saharan member of the DIOMCOOP Cooperative of Senegalese Workers
Spain	Neighbour	End-user, female. Muslim mother, volunteer at a Learning Community
Spain	Migrants association	End-user, male. Pakistani senior community leader from the city of Badalona
United Kingdom	Social Platform on Cities and Social Cohesion	Expert, male. Partner of the Social Platform on Cities and Social Cohesion. Expertise in the neighbourhood domain.
United Kingdom	University not identified due to confidentiality reasons	Expert, male. Head of the Department of Social Science. Expertise in religious affairs and attitudes of and towards ethno-religious minorities.
United Kingdom	University not identified due to confidentiality reasons	Expert, male. International expert in human rights and Muslim studies. Partner of the TOLERANCE Project. Integration of Ethnic Minorities
United Kingdom	University not identified due to confidentiality reasons	Expert, male. Senior Lecturer in Criminology & Criminal Justice. Expertise in prisons.
United Kingdom	University not identified due to confidentiality reasons	Expert, female. Senior Lecturer. Expertise in Education, Communication and Language.
United Kingdom	University not identified due to confidentiality reasons	Expert, male. Lecturer in Psychology. Expertise in Countering radicalisation.
United Kingdom	Active Change Foundation (ACF)	Stakeholder, male. Chief Executive ACF. Expertise in the neighbourhood domain.
United Kingdom	Primary School	Stakeholder, male. Professor at primary school. Interested in the CT strategy in the educational spectrum.
United Kingdom	Cambridgeshire Race Equality & Diversity Service (CREDS)	Stakeholder, female. Worker at the Race Equality and Diversity Service.
United Kingdom	St Giles Trust organization	Stakeholder, male. Expertise in organized crime. Experiences of prison, homelessness, unemployment, addiction.
United Kingdom	Safer London	Stakeholder, female. Expertise in organized crime. Experiences in youth, gangs, exploitation and crime.
United Kingdom	The Roma Project	Stakeholder, male. Director of the project.
United Kingdom	The Roma Project	Stakeholder, male. Member of the Board.

United Kingdom	Race Equality & Diversity Service (CREDS)	Stakeholder, female. Social worker.
United Kingdom	Race Equality & Diversity Service (CREDS)	Stakeholder, male. Worker
United Kingdom	Race Equality & Diversity Service (CREDS)	End-user, female. Muslim woman
United Kingdom	Prevent Safeguarding Team	Stakeholder, female. Lead trainer for Prevent in schools

ANNEX III. GUIDELINES FOR QUALITATIVE FIELDWORK

QUESTIONNAIRE GERMANY

TERRORISM

INTRODUCTION: Considering European legislation against terrorism, and the current waves of terrorist acts, which have led to the implementation of measures and policies in many EU Member states. The aim of the interview is to go further in the implementation of measures and programs against terrorism in Germany and measure their impact. Terrorism is a recurrent issue in the European Security Strategy and one of the main priorities of the Union in the field of freedom, security and justice. Several EU documents deal with the prevention of radicalization and recruitment to terrorism, such as the EU Strategy for combating radicalization and recruitment to Terrorism of the Council of the European Union (5643/5/14) or the COM (2013)941 final, Preventing Radicalization to Terrorism and Violent Extremism: strengthening the EU's Response. This last communication affirms that several Member States have already implemented measures to prevent radicalization, both internally and externally.

In the case of Germany, the most recent and important measure we have knowledge about is the:

Strategie der Bundesregierung zur Extremismusprävention und Demokratieförderung, One of the aims of the strategy is the prevention of extremism including measures that prevent and counteract the order of values of the Basic Law and the democratic constitutional state. Preventive measures are aimed at **endangered people or groups, their environment and their networks, and, where appropriate, potential perpetrators**, in order to prevent and to interrupt (violent) actions.

While another measure of the strategy in 2004 was the establishment of the **GTAZ (Joint Counter-Terrorism Centre)**, which is not an autonomous authority but a joint co-operation and communication platform used by 40 **internal security agencies**.

1. National tools used in the fight against terrorism in Germany (in particular about prevention of radicalization)

- Do you have knowledge about any other policy or measure that is being applied in Germany regarding the prevention of radicalization or recruitment in general? (For example, the EU have recommended that the EU Member states apply protocols to detect radicalization in schools/ prisons; or tools to counteract on-line terrorist propaganda and hate speech)
- And in particular regarding the areas of? :
  - Media

- Religion
- Neighbourhoods and community development
- Prisons
- Education
- Migration

- Is there any other measure of the aforementioned areas not related to TN in particular, but which could have an effect to? For example, the EU have recommended that the EU Member states enhance social inclusion trough education and non-formal learning or trough renovating plans for deprived neighbourhoods.
- Which is the main strategy followed by the police in Germany? We have knowledge that after the attacks against the United States on 11 September 2001, the German police decided to identify individuals on the basis of demographic and socio-economic criteria derived from the profile of the 9/11 terrorists, namely: being male, Muslim, aged between 18 and 40, a student or former student, and a native or national of a specified country with a predominantly Muslim population. As a result, data on up to 300,000 individuals was screened, and a total of 32,000 people were identified as "sleepers" and subject to closer scrutiny. However, no one was ever charged with a criminal offence (OSCE Report, 2014).
- Do the prevent strategy in Germany provide training to the agencies in charge of identifying early-stage radicalization? *According to the lit review: Fischbacher-Smith (2016) stresses that the current policy puts the responsibilities for identifying early-stage radicalization on organizations for whom it is not their primary concern (and for which they are ill-equipped), and there is an insufficient research evidence base available to them to show how effective the various forms of intervention might be.*

2. Impact of the measures/policies in the fight against terrorism

- Does the strategy have received comments of disagreement? Or controversial discussions? (In the affirmative case, what kind of comments or discussions?) *According to the lit review in prevention measures: Alam & Husband (2013) affirms that the introduction of counter-terrorism policy in the form of PREVENT was met with a wide range of strong resistance. Many councillors responsible for the implementation of this policy saw it as discriminatory in its unambiguous targeting of Muslim communities. At the same time, Muslims saw PREVENT as an assault on their integrity as law-abiding citizens. The introduction of pre-crime counter-terrorism legislation marks the intrusion of the construction of the enemy with anger, disgust; fear and risk (see McCulloch and Pickering, (2009)).*
- Does the strategy foresee tools or mechanisms to evaluate the impact? (In the affirmative case: how is the impact measured?; how are these tools applied?...)
- To what extent is the strategy affecting fundamental rights?
- Does the strategic plan affect the right to life privacy (including their home and communications)? *The literature reviews that CT measures foresee*

special powers of secret surveillance of citizens/ special search and seizure powers.

- Do some measures against CT involve the processing of personal data?
  - Do you know who is responsible for processing personal data?
  - Is the security of data processing activities provided for from a technical and organisational point of view?
  - Are any safeguards which render the interference into the right of data protection proportionate and necessary provided for?
- How does the strategic plan entail any different treatment of groups or individuals directly on grounds of racial or ethnic origin? Or could it lead to indirect discrimination? *The lit review affirms that some preventive measures such as Stop and Search or excessive patrolling in some regions can lead to discrimination and the creation of suspect communities. On the other hand, according to McCulloch and Pickering (2009) high levels of police discretion increase the risk of the arbitrary exercise of power and may 'lead to intensified politicization of policing and law.*

ORGANISED CRIME

INTRODUCTION: The EU has been elaborating action plans, programs and strategies either targeting organised crime in general or dealing with its particular forms. The problem has been addressed within the EU's foreign policy and accession strategy, but most of the relevant actions have been undertaken as part of police and judicial cooperation in criminal matters (EU Response to Organised Crime, 2013). OC is also a priority in the European Agenda on Security. However, the main achievements followed by this strategy are anti-money laundering packages such as financial intelligence units, asset recovery offices, among others.
Nevertheless, Germany does not include Organized crime as one of the priorities in the *White Paper on German security policy and the future of the Bundeswehr.*

Germany has transposed the **Council Framework decision 2008/841/JHA**. In Germany, the jurisprudence has consistently applied a restrictive interpretation of **Article 129 of the Criminal Code on criminal organization**. In particular, it required that the members of the association pursue a common goal and feel part of a common union, where the individual will is submitted to the common will of the group.

Some other controversies regarding this FD 2008/841/JHA are the following:

1. The use of **special investigative means** inherently carries a potential risk for abuse. Few are the European states that have not been affected by a nationwide scandal involving leakage of information collected through covert techniques. For example, **the government Trojan horse controversy in Germany** (Study on paving the way for future policy initiatives in the field of fight against organised crime: the effectiveness of specific criminal law measures targeting organised crime, Final Report, 2015. European

Commission).

- Do you think that these measures are applied indiscriminately? Or mainly to specific communities?
- **Do you have knowledge about any other policy or measure that is being applied in Germany regarding the prevention of organised crime groups?**
- **And in particular regarding the areas of? :**
  - Media
  - Religion
  - Neighbourhoods and community development
  - Prisons
  - Education
  - Migration
- **Is there any other measure of the aforementioned areas not related to OC in particular, but which could have an effect to?** For example, renovating plans for deprived neighbourhoods.
- **Does the strategy foresee tools or mechanisms to evaluate the impact? (In the affirmative case: how is the impact measured?; how are these tools applied?...)**

2. National specialist legal and investigative tools

Information about the implementation of legal and investigative tools used in the fight against organised crime. For instance: surveillance, interception of communication, covert investigations, controlled deliveries, informants, among others.
Perception about:
- Groups against which tools are most used
- Impact → whether the national legislation foresees tools to measure the impact
- Possible ways to improve each tool in order to achieve greater impact

3. Impact of the measures/policies in the fight against organised crime

- Do the last measures/ policies regarding OC receive comments of disagreement? Or controversial discussions? (In the affirmative case, what kind of comments or discussions?)
- Do the measures in OC foresee tools or mechanisms to evaluate the impact? (In the affirmative case: how is the impact measured? how are these tools applied?...)
- To what extent are the measures against Organised Crime affecting fundamental rights? (For example, wiretapping, protective custody, confiscation of mafia assets.

QUESTIONNAIRE ITALY

TERRORISM

INTRODUCTION: Considering European legislation against terrorism, and the current waves of terrorist acts, which have led to the implementation of measures and policies in many EU Member states. The aim of the interview is to go further in the implementation of measures against terrorism in Italy. Terrorism is a recurrent issue in the European Security Strategy and one of the main priorities of the Union in the field of freedom, security and justice. Several EU documents deal with the prevention of radicalization and recruitment to terrorism, such as the EU Strategy for combating radicalization and recruitment to Terrorism of the Council of the European Union (5643/5/14) or the COM (2013)941 final, Preventing Radicalization to Terrorism and Violent Extremism: strengthening the EU's Response. This last communication affirms that several Member States have already implemented measures to prevent radicalization, both internally and externally. However, comprehensive approaches under the strand of the EU CT Strategy aimed at addressing radicalization and recruitment are not widely used.

In the case of Italy, the most recent and important measure we have knowledge about is the:

Decreto-legge 18 febbraio 2015, n. 7, which contains urgent measures for counter-terrorism. This decree establishes changes in the penal code such as including the act of travelling to foreign territory as well as the increase of the penalty for many terrorist acts. Moreover, other changes are included such as the preservation of the acquired data and telematic traffic, when they are indispensable for the continuation of the activity aimed at the prevention, or the increase in expenses for the protection of national interests (in particular, for the upgrading of the aeronautical surveillance equipment and security in the central Mediterranean), among others.

Previous to the *decreto legge of 2015*, there is also **the Law no. 155 "Urgent measures to fight international terrorism"** of 2005**.** However, the aforementioned decrees are focused mainly in penal and procedural changes, or the protection of critical facilities.

1. National tools used in the fight against terrorism in Italy (in particular about prevention of radicalization)

- **Do you have knowledge about any other policy or measure that is being applied in Italy regarding the prevention of radicalization or recruitment in general?** (For example, the EU have recommended that the EU Member states apply protocols to detect radicalization in schools/ prisons; or tools to counteract on-line terrorist propaganda and hate speech)
- **And in particular regarding the areas of…?**
  - Media
  - Religion
  - Neighbourhoods and community development

- Prisons
- Education
- Migration

– **Is there any other measure of the aforementioned areas not related to TN in particular, but which could have an effect to?** For example, the EU have recommended that the EU Member states enhance social inclusion through education and non-formal learning or through renovating plans for deprived neighborhoods.

2. Impact of the measures/policies in the fight against terrorism

– Do the last Decreto-legge 18 febbraio 2015, n. 7, which contains urgent measures for the counter-terrorism, have received comments of disagreement? Or controversial discussions? (In the affirmative case, what kind of comments or discussions?)
– Do the new measures of the last Decreto-legge 18 febbraio 2015 foresee tools or mechanisms to evaluate the impact? (In the affirmative case: how is the impact measured? How are these tools applied?...)
– To what extent is the last Decreto-legge 18 febbraio 2015 affecting fundamental rights?
– Do the last Decreto-legge 18 febbraio 2015 have beneficial and negative impacts (Conflict of interests)? Which are these rights in conflict?
– Does the last Decreto-legge 18 febbraio 2015 make the public better informed about radicalization/ recruitment in terrorist groups?
– Does the last Decreto-legge 18 febbraio 2015 affect the public's access to information?
– Does the last Decreto-legge 18 febbraio 2015 affect political parties or civic organisations?
– Is the last Decreto-legge 18 febbraio 2015 affecting on particular risks groups? (social groups, mobility, region…)

ORGANISED CRIME

INTRODUCTION: The EU has been elaborating action plans, programs and strategies either targeting organised crime in general or dealing with its particular forms. The problem has been addressed within the EU's foreign policy and accession strategy, but most of the relevant actions have been undertaken as part of police and judicial cooperation in criminal matters (EU Response to Organised Crime, 2013). OC is also a priority in the European Agenda on Security. However, the main achievements followed by this strategy are anti-money laundering packages such as financial intelligence units, asset recovery offices, among others.

Italy does not have a National Security Strategy in the strict sense of the word. However, there are some measures in this sense such as the art. 146 of the Italian Penal Code; some preventive measures such as wiretapping, protective custody, confiscation of mafia assets (Law 646 (1982)), special penalties and prison regimes for persons convicted for Mafia style crimes (the offence of 'mafia-type association' (Article 416 bis c.c.); and the 'hard prison regime'

(Article 41 bis p.a.a.); but also some indirect policies such as the **security for development 2007-2013**.

Finally, the EU is enforcing *special legal and investigative tools* such as surveillance; interception of communication; covert investigations; controlled deliveries; informants, among others. However, once more these measures are not specifically focused in the prevention of recruitment in organized crime groups.

1. National tools used in the fight against organised crime

The aim is to gather information about:

- The tools used in the fight against organised crime, we can talk about the transposing of the Framework Decision 2008/841/JHA. However, this information has been analysed in several occasions.
- Other relevant national laws/measures.
- Perceived barriers and facilitators to the implementation of the aforementioned tools
- Frequency of the use of national legislative provisions, their use in practice, and their impact.

- **Do you have knowledge about any other policy or measure that is being applied in Italy regarding the prevention of organised crime groups?**
- **And in particular regarding the areas of? :**
  - Media
  - Religion
  - Neighbourhoods and community development
  - Prisons
  - Education
  - Migration
- **Is there any other measure of the aforementioned areas not related to OC in particular, but which could have an effect to?** For example, renovating plans for deprived neighbourhoods.

2. National specialist legal and investigative tools

Information about the implementation of legal and investigative tools used in the fight against organised crime. For instance: surveillance, interception of communication, covert investigations, controlled deliveries, informants, among others.

Perception about:

- Groups against which tools are most used
- Impact whether the national legislation foresees tools to measure the impact
- Possible ways to improve each tool in order to achieve greater impact

3. Impact of the measures/policies in the fight against organised crime

– Do the last measures/ policies regarding OC receive comments of disagreement? Or controversial discussions? (In the affirmative case, what kind of comments or discussions?)

– Do the measures in OC foresee tools or mechanisms to evaluate the impact? (In the affirmative case: how is the impact measured? how are these tools applied?...)

– To what extent are the measures against Organised Crime affecting fundamental rights? (for example, the art. 146 of the Italian Penal Code; some preventive measures such as wiretapping, protective custody, confiscation of mafia assets (Law 646 (1982)), offence of 'mafia-type association' (Article 416 bis c.c.); or the 'hard prison regime' (Article 41 bis p.a.a.).

– To what extent are the measures against organised crime aforementioned before have social impact?

– Does the measure impact on poverty rates? Or severe material deprivation?

- Does the measure impact on cultural diversity? Are all actors treated on equal footing? Are there specific effects on particular risk groups?
- Does the measure affect the right to take collective action?
- Do the policies improve security? Do the policies impact on crime rates?
- Do the policies impact more on a specific type of crime than others?

QUESTIONNAIRE NETHERLANDS

EXPERT IN MIGRATION AND HUMAN RIGHTS [TERRORISM]

INTRODUCTION: Considering European legislation against terrorism, and the current waves of terrorist acts, which have led to the implementation of measures and policies in many EU Member states. The aim of the interview is to go further in the implementation of measures and programs against terrorism in The Netherlands and measure their impact. Terrorism is a recurrent issue in the European Security Strategy and one of the main priorities of the Union in the field of freedom, security and justice. Several EU documents deal with the prevention of radicalization and recruitment to terrorism, such as the EU Strategy for combating radicalization and recruitment to Terrorism of the Council of the European Union (5643/5/14) or the COM (2013)941 final, Preventing Radicalization to Terrorism and Violent Extremism: strengthening the EU's Response. This last communication affirms that several Member States have already implemented measures to prevent radicalization, both internally and externally.

In the case of The Netherlands, the most recent and important protocol we have knowledge about is the:

The Netherlands comprehensive action programme to combat jihadism: overview of measures and actions.

1. **Impact of the measures/policies in the fight against terrorism in the field of migration**

In the last months, due to the increase of refugees in many EU States the Schengen Area has been modified. For example, there have been an increase in the police checks inside E.U. Member States. As you stress in your paper called SEARCHING FOR "ILLEGAL" JUNK IN THE TRUNK: UNDERLYING INTENTIONS OF (CR)IMMIGRATION CONTROLS IN SCHENGEN'S INTERNAL BORDER AREAS: *This is likely to cause new tensions within states, as the already heightened concerns about terrorism by Islamic State sympathizers is giving rise to increased identity controls on Muslims.*
Crimmigration, the growing merger of crime control and immigration control. As far as Europe is concerned, the debate has usually focused on securitization at the level of political and policy discourses. In most instances the focus is on criminalization rather than on crime and concrete attempts to deal with it, which is unsatisfactory from the perspective of criminology as a discipline (Killias, 2011).

– **Ethical Impact (Fundamental Rights Impact)**
  - In general, what is your opinion about the strategy in terms of fundamental rights affected by these measures (database- register; profiling stop and search; alleged terrorists, etc.)
  - Do you think that the application of these measures is promoting the social effect of suspect-communities?
  - Did these measures (related to border control and crimmigration) receive

comments of disagreement? Or controversial discussions? (In the affirmative case, what kind of comments or discussions?)

- Do the measures foresee tools to evaluate the impact? (In the affirmative case: how is the impact measure? How are these tools applied?...)
- To what extent is the strategy affecting fundamental rights?
- Does the strategy have beneficial and negative impacts (Conflict of interests)? Which are these rights in conflict?
- Do the prevent strategy in The Netherlands provide training to the agencies in charge of border control? *According to the lit review: Fischbacher-Smith (2016) stresses that the current policy puts the responsibilities for identifying early-stage radicalization on organizations for whom it is not their primary concern (and for which they are ill-equipped), and there is an insufficient research evidence base available to them to show how effective the various forms of intervention might be.*
- To what extent, are communities considered in the strategy?
- Did the government take into account the opinion (not only of minorities or organizations, but also of experts)?
- Effectivity of the plan → does the strategy have a real effect on terrorism risks?
- Perception of the police or other official agents

– **Societal Impact**

Preventive measures should enforce not only security measures such as stop and search, but also a prevention from a more comprehensive perspective. For example, measures which enforce the social integration trough employment or education programs. Do you know if the government is working on these kind of measures?

- Employment: new jobs created, labour market mobility…
- Improvement of working conditions
- Vocational training/ education
- Distribution of incomes/ social protection schemes
- Participation of the communities
- Public health

– **Role that play the media**

- If media content fuels the crimmigration process. Examples

QUESTIONNAIRE ROMANIA

TERRORISM

INTRODUCTION: Considering European legislation against terrorism, and the current waves of terrorist acts, which have led to the implementation of measures and policies in many EU Member states. The aim of the interview is to go further in the implementation of measures against terrorism in Romania. Terrorism is a recurrent issue in the European Security Strategy and one of the main priorities of the Union in the field of freedom, security and justice. Several EU documents deal with the prevention of radicalization and recruitment to terrorism, such as the EU Strategy for combating radicalization and recruitment to Terrorism of the Council of the European Union (5643/5/14) or the COM (2013)941 final, Preventing Radicalization to Terrorism and Violent Extremism: strengthening the EU's Response. This last communication affirms that several Member States have already implemented measures to prevent radicalization, both internally and externally. However, comprehensive approaches under the strand of the EU CT Strategy aimed at addressing radicalization and recruitment are not widely used.

In the case of Romania, the main law contributing to the coordination of the implementation of the agreements and arrangements related to preventing and combating terrorism are:

Law no. 535 / 2004 on preventing and combating terrorism. According to the provisions of art. 6, "the prevention and suppression of terrorism is organized and conducted in a unitary manner". The cooperation between national competent authorities is conducted within the National System for Preventing and Countering Terrorism. However, the Romanian Intelligence Service is the national authority in the field of countering terrorism that technically coordinates the cooperation between 20 public authorities and institutions within the System. The approval of the lists containing persons suspected of committing or financing terrorist acts is made through Government Decision (information retrieved from the answer to the questionnaire on the code of conduct on politico-military aspects of security FSC (DEC/2/09) 2016)**.**

The National Strategy on Preventing and Countering Terrorism (2002): the Strategy identifies the main aspects of the terrorist phenomenon, as a threat to Romania, defines the objectives for preventing and countering terrorism and establishes the main directions for the implementation of the National System for Preventing and Countering Terrorism.

There are further measures and instruments in the field of financing terrorism for instance, the *Order no 9 /2005 of the President of the Romanian Financial Supervisory Authority on the approval of the Instructions no 4 / 2005 on preventing the financing of terrorist acts; Government Emergency Ordinance no 135 / 2005 on the amendment of the Law no 656 / 2002 on preventing and sanctioning money laundering and on measures to prevent and combat financing terrorist acts*, among others.

1. **National tools used in the fight against terrorism in Romania (in particular about prevention of radicalization)**

- **Do you have knowledge about any other policy or measure that is being applied in Romania regarding the prevention of radicalization or recruitment in general?** (For example, the EU have recommended that the EU Member states apply protocols to detect radicalization in schools/ prisons; or tools to counteract on-line terrorist propaganda and hate speech. However, in the national plan and in the Law 535/2004 these areas are not considered).
- **And in particular regarding the areas of? :**
  - Media
  - Religion
  - Neighbourhoods and community development
  - Prisons
  - Education
  - Migration
- **Is there any other measure of the aforementioned areas not related to TN in particular, but which could have an effect to?** For example, the EU have recommended that the EU Member states enhance social inclusion through education and non-formal learning or through renovating plans for deprived neighbourhoods.

2. Impact of the measures/policies in the fight against terrorism

- Do the Law no. 535 / 2004 or/ and the National Strategy received comments of disagreement? Or controversial discussions? (In the affirmative case, what kind of comments or discussions?)
- Do the new measures of the Law no. 535 / 2004 or/ and the National Strategy foresee tools or mechanisms to evaluate the impact? (In the affirmative case: how is the impact measured? How are these tools applied?...)
- To what extent is the Law no. 535 / 2004 or/ and the National Strategy affecting fundamental rights?
- Do the Law no. 535 / 2004 or/ and the National Strategy have beneficial and negative impacts (Conflict of interests)? Which are these rights in conflict?
- Do the Law no. 535 / 2004 or/and the National Strategy make the public better informed about radicalization/ recruitment in terrorist groups?
- Do the Law no. 535 / 2004 or/and the National Strategy affect the public's access to information?
- Do the Law no. 535 / 2004 and/ or the National Strategy affect political parties or civic organisations?
- Are the Law no. 535 / 2004 or/ and the National Strategy affecting on particular risks groups? (social groups, mobility, region…)

ORGANISED CRIME

INTRODUCTION: The EU has been elaborating action plans, programs and strategies either targeting organised crime in general or dealing with its particular forms. The problem has been addressed within the EU's foreign policy and accession strategy, but most of the relevant actions have been undertaken as part of police and judicial cooperation in criminal matters (EU Response to Organised Crime, 2013). OC is also a priority in the European Agenda on Security. However, the main achievements followed by this strategy are anti-money laundering packages such as financial intelligence units, asset recovery offices, among others.
Romania has a National Defense Strategy (2015-2019) in the strict sense of the word, where organized crime groups are one of the priorities. However, the measures to combat this kind of criminal groups are not written in detail.

Finally, the EU is enforcing *special legal and investigative tools* such as surveillance; interception of communication; covert investigations; controlled deliveries; informants, among others. However, once more these measures are not specified in the national strategic plans.

4. National tools used in the fight against organised crime

The aim is to gather information about:

- The tools used in the fight against organised crime, we can talk about the transposing of the Framework Decision 2008/841/JHA. However, this information has been analysed in several occasions.
- Other relevant national laws/measures.
- Perceived barriers and facilitators to the implementation of the aforementioned tools
- Frequency of the use of national legislative provisions, their use in practice, and their impact.

– Do you have knowledge about any other policy or measure that is being applied in Romania regarding the prevention of organised crime groups?
– And in particular regarding the areas of? :
    - Media
    - Religion
    - Neighbourhoods and community development
    - Prisons
    - Education
    - Migration
– Is there any other measure of the aforementioned areas not related to OC in particular, but which could have an effect to? For example, renovating plans for deprived neighbourhoods.

5. National specialist legal and investigative tools

Information about the implementation of legal and investigative tools used in the fight against organised crime. For instance: surveillance, interception of

communication, covert investigations, controlled deliveries, informants, among others.<br>Perception about:<br>- Groups against which tools are most used<br>- Impact whether the national legislation foresees tools to measure the impact<br>- Possible ways to improve each tool in order to achieve greater impact

6. Impact of the measures/policies in the fight against organised crime

- Do the last measures/ policies regarding OC receive comments of disagreement? Or controversial discussions? (In the affirmative case, what kind of comments or discussions?)
- Do the measures in OC foresee tools or mechanisms to evaluate the impact? (In the affirmative case: how is the impact measured? how are these tools applied?...)
- To what extent are the measures against Organised Crime affecting fundamental rights?
- To what extent are the measures against organised crime aforementioned before have social impact?
  - Does the measure impact on poverty rates? Or severe material deprivation?
  - Does the measure impact on cultural diversity? Are all actors treated on equal footing? Are there specific effects on particular risk groups?
  - Does the measure affect the right to take collective action?
  - Do the policies improve security? Do the policies impact on crime rates?
  - Do the policies impact more on a specific type of crime than others?

ENTREVISTA ESPAÑA : EXPERTO RELIGIÓN

TERRORISMO

INTRODUCCIÓN: considerando la legislación de la Unión Europea contra el terrorismo y las atentados terroristas de los últimos meses, los cuales han llevado a la implementación de medidas y políticas de emergencia en muchos países miembro de la UE. El objetivo de la entrevista es profundizar en el efecto que han tenido estas medidas y políticas en España. El terrorismo es un tema recurrente en la Agenda Europea de Seguridad y también uno de las principales prioridades de la UE en el área de libertad, seguridad y justicia. Muchos documentos de la UE intentan dar pautas para la prevención del terrorismo como sería el caso de la Estrategia de la UE para combatir la radicalización y el reclutamiento para el terrorismo del Consejo de la UE (5643/5/14) o la COM (2013)941 Previniendo la radicalización al terrorismo y al extremismo violento: reforzando la estrategia de la UE.

En el caso de España, las medidas/estrategias sobre las que tenemos conocimiento son las siguientes:

- **Plan estratégico nacional de lucha contra la radicalización violenta. (PEN-LCRV). "Un marco para el respeto y el entendimiento común".** Este plan, siguiendo el modelo de la UE contiene 3 áreas:
    - **Prevenir (antes):** destinado a asegurar la integración y la convivencia social, especialmente de las comunidades o colectivos más vulnerables o en situación de riesgo, interviniendo desde la detección de situaciones de falta de integración social o conflictividad, tratando de impedir procesos de radicalización violenta.
    - **Vigilar (durante):** funciones de observación, vigilancia y tratamiento.
    - **Actuar (después):** seguir a comunidades/ colectivos o individuos en los que ya se han producido los procesos de radicalización violenta.

En el área de prevenir del plan se especifica que:

- En el marco de la educación y la sensibilización social: se asegurará por parte de la Administración el conocimiento de la formación y educación de los diferentes actores educativos, en materias directamente relacionadas con la problemática tratada, incluyendo a grupos vulnerables o en riesgo de radicalización violenta. **Se regulará la formación específica de formadores en aquellas cuestiones ideológicas, culturales o religiosas especialmente conflictivas, garantizándose el conocimiento del marco constitucional español** y las iniciativas especificadas sobre integración, exclusión y segregación social y radicalización violenta en el "área de prevenir".

El plan también especifica que deberían incorporarse en el plan comunidades religiosas:

- Se trata de Incorporar al seno del GN-LCRV no sólo a personas, organismos e instituciones relevantes o con influencia en la comunidad, sino también a

otros actores potenciales del sector privado, como organizaciones no gubernamentales, seguridad privada, personal académico, docente y educativo, **religioso**, sanitario y de asistencia social, entre otros.

- ¿Tiene conocimiento que las estrategias/ medidas que se prevén en el plan se están llevando a cabo? ¿El plan prevé mecanismos de evaluación de impacto en temas de religión?
- ¿Hay medidas/ legislación concreta en temas de religión que se esté usando para la prevención de la radicalización, aunque ese no sea su objetivo principal?
- ¿Ha recibido comentarios de desacuerdo el plan estratégico en temas de religión? Según la literatura científica, la estrategia de Prevenir produce el estigma de ciertas comunidades religiosas y ha recibida numerosas críticas.
- ¿Se tuvo en cuenta el diálogo con diferentes comunidades religiosas para crear el protocolo?
- ¿Tiene conocimiento si se está incluyendo a las comunidades religiosas actualmente en temas de protocolos anti-radicalización?
- ¿Hay evidencia que el programa está trabajando para que haya una mejor información sobre procesos de radicalización? ¿Y sobre la confusión entre religión islámica y terrorismo islamista?

1. Impacto Ético (Impacto en los derechos humanos)

a. General

- ¿El plan estratégico contra el terrorismo está afectando los derechos individuales de las personas que pertenecen a comunidades religiosas?

- ¿El plan estratégico tiene impactos negativos y a la vez también positivos? ¿Cuáles son los derechos en disputa? Por ejemplo, la literatura científica indica que las políticas anti-terroristas presentan la disputa entre privacidad vs seguridad.

b. Libertad de expresión y de consciencia

- ¿El plan estratégico está afectando la libertad de pensamiento, conciencia o religión?

- ¿Está el plan estratégico afectando la libertad de expresión e información? ¿y de asamblea y asociación?

c. Igualdad de género, de oportunidades, de no discriminación.

- ¿Los planes estratégicos están dando un trato diferente a determinados grupos o individuos debido a su orientación religiosa?

d. Datos personales

- ¿Tiene conocimiento de si se están reteniendo datos personales de sospechosos por "terrorismo" según su participación en actos/ comunidades religiosas? ¿hay garantías? ¿se tienen en cuenta mecanismos de revisión y supervisión?

2. Impacto Social

a. Gobernanza y participación

- ¿El programa estratégico tiene en cuenta organizaciones sociales o entidades religiosas?
- ¿hay un trato diferente dependiendo de si las organizaciones o entidades tienen vínculos religiosos?
- ¿El plan promueve una mejor información sobre los procesos de radicalización?
- ¿el plan estratégico afecta organizaciones cívicas, sociales o entidades religiosas?

b. Seguridad pública

- ¿La estrategia tiene un impacto en la seguridad de los individuos?
- ¿La estrategia produce efectos predominantemente sobre grupos en riesgo?

c. Terrorismo y seguridad

- ¿La estrategia tiene un efecto en la seguridad? ¿en concreto, en delitos de terrorismo?
- ¿La estrategia aumenta la probabilidad de detectar casos de radicalización?
- ¿La estrategia aumenta las capacidades de los cuerpos policiales para contrarrestar el terrorismo?

DELINCUENCIA ORGANIZADA

INTRODUCCIÓN: la UE ha elaborado planes de acción, programas y estrategias ya sean para combatir la delincuencia organizada en general como para lidiar con sus formas específicas. La delincuencia organizada es una prioridad en la Agenda Europea de Seguridad. No obstante, los máximos logros seguidos por esta estrategia son medidas de lavado de dinero o creación de unidades de inteligencia financiera o recuperación de activos. De modo que no existen medidas concretas relacionadas con asuntos religiosos. Son básicamente cambios en el código penal y estrategias de inteligencia para impedir el asentamiento de los grupos criminales organizados y poner a disposición de la justicia a los que ya operan dentro de nuestras fronteras.

– ¿Tiene conocimiento sobre protocoles específicos en materia de religión que se estén aplicando para evitar reclutamiento en bandas de delincuencia organizada?
– ¿Hay medidas/ protocolos concretos en temas de religión que se esté usando para la prevención de la delincuencia organizada, aunque ese no sea su objetivo principal?

QUESTIONNAIRE UK

EXPERT for the field of IMMIGRATION

The document contains:

a) Background information about organised crime and terrorism policies in the United Kingdom
b) Questionnaire to the expert for the field of immigration

BACKGROUND INFORMATION ABOUT ORGANISED CRIME AND TERRORISM POLICIES IN THE UNITED KINGDOM

TERRORISM POLICIES AND LEGAL FRAMEWORK

Criminal and procedural code

The United Kingdom has a long tradition in Terrorism strategies, protocols and legislation, before and since the attacks of the 9/11 in the United States.

The firsts acts defining terrorism as a criminal offence were expressed in the Prevention of Terrorism (Temporary Provisions) Act 1989, which was one of the Prevention of Terrorism Acts of the United Kingdom related to the troubles in Northern Ireland. Subsequently, further provisions were created, and some of them, such as the Act 2000 and following received some controversial discussions and statements for being against the European Convention on Human Rights.

The Act 2000 has been described as the centrepiece of the United Kingdom's legislation on terrorism, but at the same time, some measures such as the stop-and-search powers under section 44 of the Act have been ruled illegal by the European Court of Human Rights. In response to the terrorist attacks on 11 September 2001, further counter terrorism powers were introduced under the Anti-Terrorism, Crime and Security Act 2001 ("the ATCS Act"). However, the introduction of some procedural changes received many controversial discussions. The most recent legislation is the Act 2015[21], which introduced once more a critical point: the statutory duty of reporting cases of radicalization to school staff, local authorities, prison personnel and NHS trusts[22].

Strategic Plans

The Prevent strategy, published by the Government in 2011, is part of the overall counter-terrorism strategy, CONTEST[23]. The aim of the Prevent strategy is to reduce the threat from terrorism by stopping people becoming

---

[21] http://www.legislation.gov.uk/ukpga/2015/6/part/1/chapter/2.

[22] This statutory duty is very controversial for the fact that teachers and other staff have not the training or knowledge enough about radicalization to report suspects.

[23] https://www.gov.uk/government/uploads/system/uploads/attachment_data/file/97994/contest-summary.pdf.

terrorists or supporting terrorism. In the Act this has simply been expressed as the need to "prevent people from being drawn into terrorism".

The most recent strategy in the United Kingdom is the Counter-Extremism Strategy 2015. The strategy response is focused in four areas. First, countering extremism ideologies with the aim of confronting and challenging extremist propaganda and ensuring no space goes uncontested.

Second, building a partnership with all those opposed to extremism standing with and building the capacity of mainstream individuals, community organisations and others in our society who work every day to challenge extremists and protect vulnerable individuals. This point has received many critiques because includes the *statutory Prevent duty, so that all local authorities, schools, universities and colleges, NHS Trusts and Foundation Trusts, police, probation services and prisons are clear that they must take action to prevent people being drawn into terrorism*[24]*.*

Thirdly, disrupt extremism creating new mechanisms or revising existing ones. For example, in areas of asylum, citizenship and immigration; helping the public to report extremism;

Finally, promoting more cohesive communities through measures such as the National Citizen Service helping young people to become more active and responsible citizens[25]; providing English language training; enforcing the disappearance of illegal cultural practices. For example, tackling Violence against women and girls delivering comprehensive programmes to prevent Female Genital Mutilation or forced marriage. Finally, one of the cohesive measures is the promotion of more opportunities in terms of housing, health and education. These measures will rely on the Casey review[26].

In this guide it is also introduced the controversial definition of extremism:

> *"vocal or active opposition to <u>fundamental British values</u>, including democracy, the rule of law, individual liberty and mutual respect and tolerance of different faiths and beliefs. We also include in our definition of extremism calls for the death of members of our armed forces".*

What is understand as fundamental British values is subjective and leading to confusion. It has been criticized by many academics such as the expert in education interviewed for the fieldwork in UK.

Another polemic point in the prevent strategy is definition as a factor to detect individuals in risk of being drawn into terrorism. This includes not just violent extremism but also non-violent extremism, which can create an atmosphere conducive to terrorism and can popularise views which terrorists exploit.

---

[24] The prevent duty applied in schools has been one of the most controversial points of the strategy. There are several documents addressing this. For example: Preventing education? Human rights and CT in schools (Rights Watch UK).

[25] In the strategy is affirmed that there is an evaluation of this service called: Evaluation of National Citizen Service, July 2013, NatCen Social Research, Office of Public Management and New Philanthropy Capital.

[26] https://www.gov.uk/government/uploads/system/uploads/attachment_data/file/575973/The_Casey_Review_Report.pdf.

In the prevent strategy is detailed the process to be followed by the local authorities to prevent cases of extremism. From the training to the report of the cases, as well as the Office for Standards in Education, Children's Services and Skills (Ofsted) in charge of inspecting that schools are accomplishing with fundamental British values.

There is another document focused on the training of the local authorities aimed at this goal. It is the Channel Duty Guidance: Protecting vulnerable people from being drawn into terrorism. Channel is the program to which the people suspected of being radicalised are referred to. The Channel strategy was first piloted in 2007 and rolled out across England and Wales in April 2012. Channel is a programme which focuses on providing support at an early stage to people who are identified as being vulnerable to being drawn into terrorism. The programme uses a multi-agency approach to protect vulnerable people by: a. identifying individuals at risk; b. assessing the nature and extent of that risk; and c. developing the most appropriate support plan for the individuals concerned. The Channel duty guide lists the functioning of the program and also the authorities' eligible to take part of the program. The Channel program is important to consider as one of the main measures of the counter-extremism strategy because it includes the cooperation between many local actors and allies in a grassroots level. For instance, organization, which work with the most vulnerable population.

There is another document reviewing the actions and methodology to be followed in cases of detection of extremism. This document is called Counter-terrorism local profiles: an updated guide[27]. In this document is affirmed that integration is an important part in the prevention of terrorism.

> *'Creating the Conditions for Integration' which outlines the Government's approach for creating an integrated society. The report highlights the role of local authorities in promoting integration and challenging extremism. However, at the same time, it seems like the Channel program is a government intelligence strategy, gathering intelligence from different sources.*

ORGANISED CRIME POLICIES AND LEGAL FRAMEWORK

Criminal and procedural code

Offences committed by joint offenders in prosecution of common purpose 20. When two or more persons form a common intention to prosecute an unlawful purpose in conjunction with one another and in the prosecution of such purpose, an offence is committed of such a nature that its commission was a probable consequence of the prosecution of such purpose, each of them is deemed to have committed the offence.

In the Serious and Organised Crime Strategy (2013) organised crime is explained that there is no legal definition of organised crime in England and

---

[27] https://www.gov.uk/government/uploads/system/uploads/attachment_data/file/118203/counter-terrorism-local-profiles.pdf.

Wales. However, in this strategy, organised crime is serious crime planned, coordinated and conducted by people working together on a continuing basis. Their motivation is often, but not always, financial gain.

Strategic Plan

The United Kingdom is the only country for our fieldwork offering a specific strategy to combat organised crime, which is called serious and Organised Crime Strategy (2013). The strategy is following the framework developed for Counter- terrorism, focusing in four areas: pursue (Prosecuting and disrupting serious and organised crime), prevent (Preventing people from engaging in serious and organised crime), protect (Increasing protection against serious and organised crime) and prepare (Reducing the impact of serious and organised crime).

This strategy introduces for the first time Prevent programmes for serious and organised crime, in England and Wales. According to the strategy achieving success in PREVENT would mean that: fewer people engage in serious and organised criminal activity; and to reduce reoffending by people convicted for serious and organised crime. The specific objectives are the following:

i. Deter people from becoming involved in serious and organised crime by raising awareness of the reality and consequences

ii. Use interventions to stop people being drawn into different types of serious and organised crime

iii. Develop techniques to deter people from continuing in serious and organised criminality

iv. Establish an effective offender management framework to support work on Pursue and Prevent

It is explained in the strategy that prevention will include better education and communications about organized crime, local coordination with existing work on troubled families and gangs, and wider use of interventions (e.g. Serious Crime Prevention Orders).

The strategy relies on local powers, in other words, a local network of prevention related to terrorism.

> *Organised crime Prevent work should also be coordinated with work on preventing terrorism. It may be possible in some areas for the same local team to take responsibility for both issues.*

Main actions to stop people being drawn into OC:

- The Troubled Families Programme: focused on families that have multiple problems
- The ending gang and youth violence programme: prevent young people from becoming involved in street gangs as a previous step to enter OC groups.
- Preventing child sexual exploitation

In the strategy there are some statements could be interesting for our research. For example:

- About 50% of the international drug trafficking groups of interest to the US Department of Justice are associated with terrorist organisations.
- Crime groups can have a corrosive impact on the fabric and cohesion of communities. The abuse and exploitation of children can have a lifelong and devastating impact on victims.
- Over half of the organised crime groups operating against the UK are involved in drug-related crime; a significant proportion are also involved in violent crime.

QUESTIONNAIRE

We would like to know a little bit more about the serious and organized crime strategy, and also the prevent duty guidance against CT and how these strategies contain measures and actions, which can have a particular impact on the most vulnerable communities, such as ethnic minorities.

On the one hand we would like to talk about your perception of statements as the following:

a. The majority of illegal migrants will rely on the services of organised crime groups at some point in their journey or during their time in the UK.
b. The NCA will work closely with the Border Force which is responsible for detecting threats and seizing illicit goods, checking immigration status, searching baggage, vehicles and cargo for illicit goods or illegal immigrants, patrolling the UK coastline and searching vessels.

  – What is your perception of the protocols tackling irregular migration?
  – Does the government consider migrant communities/ civil society organizations to elaborate and introduce the measures?
  – Does the program foresee tools or mechanisms to evaluate the impact? And the efficacy?
  – Does the strategy have received comments of disagreement? Or controversial discussions?
  – What fundamental rights are affected?
    - Dignity
    - Personal data (processing of personal data, right and access to rectification?
    - Expulsion or extradition
    - Rights of the child? In case of unaccompanied minors
    - Working conditions (examples such as top manta in Spain, without a regular status in the country and with trouble to find a job)
  – Do you think that some of the prevent programs of the serious and organsied crime strategy, such as the Troubled Families Programme and the Ending Gang and Youth Violence Programme are being implemented focusing mainly to specific regions in the UK? Or to specific communities? Do you know if some of these protocols are mainly focus to migrant communities?
  – Does the government consider migrant communities/ civil society

organizations to elaborate and introduce the measures?

- What kind of preventive measures is the government using to decrease discrimination/ racism?
- What kind of barriers are migrants facing to integration?
  - Including restrictions attached to their immigration status, hostile public attitudes and discrimination.
- What kind of facilitators are migrants facing to integration?
  - Facilitators: education, role models, work permits

Regarding to the Prevent strategy against CT:

- Does the implementation of prevention strategies led to the creation of "suspect communities" on grounds of religion or belief?[28]
  - Does the government consider migrant or religious communities/ civil society organizations to elaborate and introduce the measures?
  - Does the option promote different treatment of groups or individuals on grounds of religion or belief?
  - Do some measures affect freedom of thought, conscience or religion?
  - Does the strategy have received comments of disagreement? Or controversial discussions?
  - What fundamental rights are affected?
    - Dignity
    - Personal data (processing of personal data, right and access to rectification?
    - Expulsion or extradition
    - Rights of the child? In case of unaccompanied minors
    - Working conditions (examples such as top manta in Spain, without a regular status in the country and with trouble to find a job)

[28] **According to the Lit review Alam & Husband (2013):** Muslims saw **PREVENT strategies** as an assault on their integrity as law-abiding citizens; and Muslim are being exploited to further intrusive policies of control and surveillance.

CHAPTER 3: Report on Criminal Careers of OC Offenders in Context (VU & WODC)

Authors: E.R. Kleemans (VU), M.V. van Koppen (VU), V.R. van der Geest (VU/NSCR), E.W. Kruisbergen (WODC), D.R. Madarie (WODC)

Table of contents

SUMMARY 146

1. SUMMARY OF PART 1: ANALYSIS OF CRIMINAL CAREERS 148

1.1. DATA 148
1.2. METHODS 148
1.3. RESULTS 148
1.4. CONCLUSIONS 149

2. SUMMARY OF PART 2: CRIMINAL CAREERS AND SOCIAL AND ECONOMIC EMBEDDEDNESS 152

2.1. DATA AND METHODS 152
2.2. LOGISTICAL OPERATIONS 152
2.3. OCCUPATIONAL EMBEDDEDNESS 153
2.4. SOCIAL EMBEDDEDNESS 153
2.5. BECOMING AND STAYING INVOLVED 153
2.6. CONCLUSION: SYNTHESIS OF INVOLVEMENT MECHANISMS 153

3. PART 1: ANALYSIS OF CRIMINAL CAREERS 155

3.1. INTRODUCTION 155
3.2. DATA 155
3.3. METHODS 155
3.4. RESULTS 156
3.5. CONCLUSIONS 156
3.6. REFERENCES 156

4. PART 2: TRAFFICKERS IN TRANSIT: ANALYSING THE LOGISTICS AND INVOLVEMENT MECHANISMS OF ORGANISED CRIME AT LOGISTICAL NODES IN THE NETHERLANDS 160

4.1. INTRODUCTION 160
4.2. BECOMING INVOLVED IN TRANSIT CRIME 161
4.3. INVOLVEMENT MECHANISMS IN ORGANISED CRIME 162
4.4. TRANSIT CRIME AND LOGISTICAL NODES IN THE NETHERLANDS 163
AMSTERDAM AIRPORT SCHIPHOL 164
PORT OF ROTTERDAM 164

4.5. International drug trafficking: a short crime script 165
4.6. Data and methods 166
4.7. Empirical results 167
Short description of criminal networks 167
4.8. Logistical bottlenecks: getting the drugs through 168
Defying security checks 168
Avoiding security checks 169
Neutralising security checks 171
Differences between airport and seaport 171
Required skills and assets 171
4.9. Occupational embeddedness 172
4.10. Social embeddedness 174
4.11. Becoming and staying involved in organised drug trafficking 177
Becoming involved 177
Staying involved 179
4.12. Synthesis: factors leading to involvement in organised crime 181
Economic factors 181
Occupational and social factors 182
Personal factors 183

5. CONCLUSION AND DISCUSSION 183

Dilemmas for (air)port security 184
Directions for future research 185

REFERENCES 187

APPENDIX A: THE CASES 189

Airport cases 189
Seaport cases 190

Summary

Part 1 consists of the analysis of the criminal careers of 1,841 OC offenders. The main conclusion is that individuals involved in organized crime at some point in their lives follow diverse pathways in crime. While the majority of individuals starts offending in adulthood and offends at a low rate, a smaller proportion of individuals starts offending in adolescence and shows higher offence rates.

Part 2 focuses on criminal careers and social and economic embeddedness. The analysis consists of the systematic description of the social and economic embeddedness of criminal networks active at a big airport and in a big harbour, based upon analysis of sixteen extensive (closed) police investigations.
From the analyses, several types of involvement mechanisms could be distinguished. Economic involvement mechanisms relate to the logistical nodes itself. Although logistical nodes are not meant to facilitate the trafficking of illicit products from one place to another, they do provide a breeding ground

for organized crime. Without airports and seaports, organized crime groups would have to find different methods to traffic illicit products from one country to another. Therefore, the existence of a logistical node itself is a factor that facilitates involvement in organized crime. The tension between speed and safety at logistical nodes also facilitates involvement in organized crime. Not all people and products can be equally thoroughly checked and more security checks implies lower processing speed which in turn results in less profit. Organized crime groups use this tension between speed and safety to their advantage.

Occupational and social involvement mechanisms relate to the occupational and social embeddedness of organized crime. Having a job at the airport could facilitate involvement in organized crime. Certain characteristics of jobs at the airport could make it easier to discretely traffic drugs or to explore new trafficking routes or smuggling methods. Jobs also enlarge one’s social network. Moreover, persons added to one’s social network through work, usually colleagues, are more likely to be useful as well to the organized crime group. Through the social snowball effect, persons recruited by organized crime groups recruit new persons from their social network and, this way, the cycle continues. It could therefore be stated that having criminal contacts increases the chance to become involved in organized crime.

Personal factors affect the likelihood of involvement in organized crime as well. Opportunities to become involved are not always seized by potential offenders. What makes potential offenders motivated for involvement depends in part on their personal circumstances. Examples of such personal circumstances are financial setbacks and a desire for thrill and excitement.

1. Summary of part 1: Analysis of criminal careers

The aim of this study is to describe the criminal careers of individuals involved in organized crime activities at some point in their lives. Furthermore, their criminal pathways are related to individual characteristics, such as gender and country of birth, and characteristics related to their criminal involvement, such as crime mix and time spent in prison.

1.1. Data

For the purpose of this study, information from the Dutch Organized Crime Monitor (OCM) is combined with information on criminal careers from the Dutch Offender Index (OBJD). In four sweeps of the OCM, information of a wide cross-section of 150 cases concerning various forms of organized crime was collected. For 1,841 individuals (out of 2,305) involved in these cases, entire criminal careers were reconstructed using information (*rap sheets*) from the OBJD. For these individuals, information on judicial contacts (timing and nature of offenses, but also how the criminal case was adjudicated) is available from age 12 (the minimum age of legal responsibility in the Netherlands) up to 2016 or death (if this occurred prior to 2016). Most of the individuals are male (90 percent) and a narrow majority was born in the Netherlands (53 percent, see also table 1). On average, they were 27 years old when their first judicial contact took place and they spent a total of 2.4 years in prison at the end of the observation period. The majority was involved in drug-related activities; traditional drugs (30 percent), synthetic drugs (9 percent) or both (19 percent). One out of five was involved in organized fraud. Eight percent of the individuals were involved in human smuggling and another eight percent in human trafficking. In their entire criminal career, they committed an average of just over five crimes.

1.2. Methods

Semi-parametric group-modelling is used to identify clusters of individuals with similar developmental pathways in crime over time. Multiple trajectory analyses are carried out and both the Bayesian Information Criterion and probabilities of group membership are used as a basis for selecting the optimal model. Given the chosen model, individuals are assigned to the trajectory where his or her posterior probability of group membership is highest.

1.3. Results

A six-group model was selected as the optimal model to fit the criminal careers of the 1,841 organized crime offenders. Figure 1 provides a graphical representation of the criminal careers of the six distinguished groups. Characteristics of each of the six distinct groups are presented in table 2. Most individuals are assigned to a trajectory group named very low-frequency

offenders (VLF, 53 percent). Individuals assigned to this group on average committed the smallest number of crimes (1.2) and spent the least amount of time in prison (1 year) from all groups. Also, of all groups the highest proportion women (14%) and individuals born abroad (56%) are among the VLF offenders. The criminal path followed by the low-frequency offenders (LF, 18 percent) is very similar to that followed by the VLF offenders, with the difference that LF offenders committed some more crimes during their career (4.8 on average) and spent an average of almost three years in prison. The criminal pathways of three other groups, the early bloomers (EB, 8 percent), low-frequency chronics (LFC, 10 percent) and late bloomers (LB, 5 percent) have similar shapes, but they start and peak at different stages in life. While EBs have their first judicial contact around age 17 and LFCs around age 20, LBs do not start their career before age 30. In the same way, EBs reach their offending peak around age 20, criminal careers of LFCs peak around age 30, and LBs commit the most crimes around age 45. In contrast to these differences, the total number of crimes committed (9 to 10) and the number of years spent in prison (3 to 4 years) is pretty similar for these three trajectory groups. The high-frequency chronics (HFC, 6 percent) are by far the most criminally active group. They do not only have the lowest onset age of all groups (16 years), but also committed the most crimes in their careers (25 on average) and spent the most time in prison (almost 9 years on average).

1.4. Conclusions

The analyses show that individuals involved in organized crime at some point in their lives follow diverse pathways in crime. While the majority of individuals starts offending in adulthood and offends at a low rate, a smaller proportion of individuals starts offending in adolescence and shows higher offence rates.

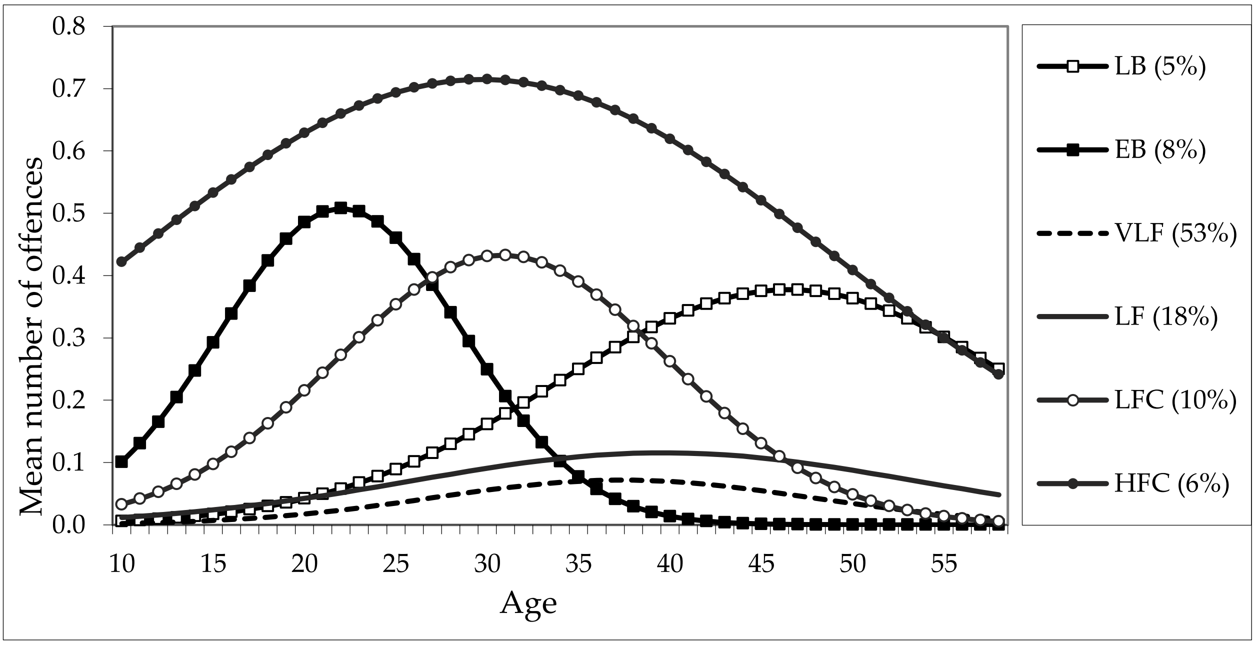

Figure 1. Trajectory groups

Late bloomers (LB); early bloomers (EB); very low-frequency offenders (VLF); low-frequency offenders (LF); Low-frequency chronic offenders (LFC); High-frequency chronic offenders (HFC).

Table 1. Descriptives total sample

	% / Mean (SD)
Male	90%
Born in the Netherlands	53%
Year of birth	1963 (10.5)
Onset age	27.2 (10.4)
Last crime in dataset	43.6 (11.1)
Follow-up age	52.8 (10.3)
Time spent in prison (years)	2.4 (3.8)
Type of organized crime	
Traditional drugs	30%
Synthetic drugs	9%
Trad+synt drugs	19%
Human smuggling	8%
Human trafficking	8%
Fraud + money laundering	21%
Other	5%
Total number of crimes in career	5.3 (7.2)
Violence	0.6 (1.5)
Sexual offences	0.02 (0.2)
Property	1.4 (3.1)
Vandalism, public order	0.6 (1.1)
Drugs	0.9 (1.6)
Traffic	0.8 (2.0)
Other	1.0 (1.9)

Table 2. Descriptives differentiated by trajectory group

	Late bloomers (N=96)	Early bloomers (N=144)	Very low-frequency offenders (N=971)	Low-frequency offenders (N=324)	Low-frequency chronic offenders (N=192)	High-frequency chronic offenders (N=114)
	Mean (SD) / %					
Type of organized crime						
Traditional drugs	35%	38%	27%	32%	39%	26%
Synthetic drugs	13%	17%	8%	8%	13%	8%
Trad+synt drugs	22%	19%	17%	18%	18%	32%
Human smuggling	2%	6%	10%	10%	4%	1%
Human trafficking	7%	3%	9%	9%	4%	4%
Fraud+ML	17%	9%	25%	20%	15%	15%
Other	4%	7%	4%	3%	7%	15%
Male	99%	97%	86%	92%	96%	99%
Born in the Netherlands	80%	64%	44%	45%	68%	82%
Year of birth	1951 (8.4)	1968 (8.4)	1963 (10.7)	1964 (11.0)	1964 (8.9)	1962 (7.0)
Onset age	29.7 (6.6)	16.9 (3.1)	30.9 (10.2)	27.9 (10.5)	20.1 (4.3)	16.2 (2.7)
Last crime in dataset	57.6 (8.4)	40.8 (8.1)	42.2 (11.1)	41.4 (11.6)	46.5 (8.1)	49.7 (7.2)
Follow-up age	63.1 (8.5)	47.9 (8.3)	52.9 (10.4)	52.2 (10.7)	51.6 (8.9)	52.8 (7.2)
Time spent in prison (years)	4.1 (4.8)	3.1 (3.7)	1.0 (1.7)	2.9 (3.5)	3.9 (4.3)	8.7 (6.0)
Total number of crimes in career	10.4 (5.7)	8.7 (4.6)	1.2 (1.5)	4.8 (3.2)	9.7 (4.1)	25.3 (9.9)
Violence	0.8 (1.2)	1.2 (1.8)	0.1 (0.4)	0.3 (0.9)	1.1 (1.4)	3.8 (3.3)
Sexual offences	0.1 (0.6)	0.03 (0.2)	0.003 (0.06)	0.009 (0.1)	0.03 (0.3)	0.03 (0.2)
Property	1.9 (2.4)	2.8 (3.1)	0.2 (0.6)	0.9 (1.6)	2.1 (2.2)	8.7 (6.9)
Vandalism, public order	0.9 (1.1)	1.0 (1.3)	0.2 (0.4)	0.9 (1.1)	0.9 (1.1)	2.4 (2.2)
Drugs	2.0 (2.1)	1.0 (1.4)	0.2 (0.5)	1.3 (1.7)	1.3 (1.6)	3.0 (3.4)
Traffic	2.6 (3.3)	1.1 (1.7)	0.2 (0.6)	0.2 (0.7)	2.2 (2.4)	3.1 (4.6)
Other	2.1 (2.5)	1.5 (1.8)	0.3 (0.6)	1.1 (1.7)	2.0 (2.4)	4.2 (3.5)

2. Summary of part 2: Criminal careers and social and economic embeddedness

The aim of this study is to analyze involvement mechanisms of organized crime. First, the study examines how drug traffickers operate at important logistical nodes in the Netherlands. Second, the study analyzes the social and occupational embeddedness of organized crime activities of these drug traffickers. The draft report is titled 'Traffickers in transit: Analyzing the logistics and involvement mechanisms of organised crime at logistical bottlenecks in the Netherlands' (Madarie & Kruisbergen, 2017).

2.1. Data and methods

The present study uses a selection of sixteen cases from the Dutch Organized Crime Monitor (OCM). The OCM is an ongoing research project (since 1998), aimed at gaining insight into organized crime in the Netherlands by examining files of closed Dutch police investigations of organized crime groups. A subset of sixteen drugs cases revolving around the main logistical nodes was selected from the OCM. Eleven cases revolved around the main airport, whereas five cases revolved around the main seaport. To analyze the logistics of organized crime activities, information regarding the modus operandi of criminal groups was gathered from the cases. To analyze the occupational and social embeddedness of organized crime activities, information on topics such as jobs, the legal work place, and social relationships was gathered. The analyses focused primarily on the eleven airport cases. The five seaport cases were used to enrich the data.

2.2. Logistical operations

Three types of tactics to traffic drugs without getting caught can be discerned. First, to defy security checks, criminal groups most often deployed drug mules or methods that were supposed to avoid unusual shapes (of drugs) on scanner images. Second, to avoid security checks at all, criminal groups attempted to recruit persons with job-related personal credentials or privileges through which drugs could be smuggled without passing security checks. Therefore, deliberate recruitment was aimed at persons employed at logistical nodes, such as (former) colleagues. Finally, to neutralize security checks, criminal groups corrupted customs or police officers. In exchange for their help, officers received money and pictures of drug mules, so they would know which persons not to check.

2.3. Occupational embeddedness

The occupational embeddedness of organized crime activities was manifested through job-related factors. Autonomy, mobility, and the similarity between legitimate duties and criminal activities facilitated discrete engagement in organized crime activities during work time. Mobility also facilitated exploration of new trafficking routes. Social capital and job-specific knowledge - both acquired through work - are two other factors that make port employees more interesting to organized crime groups. These types of contacts and knowledge are rather scarce yet very useful to groups operating at logistical nodes.

2.4. Social embeddedness

Persons with whom group members already had a social bond were more likely to be introduced to the criminal group. The social snowball effect came here into play. Current members searched for potential offenders in their network and once involved, the new group members did the same. The most common types of social bonds encountered in the cases were family ties and job-related ties, such as ties with colleagues. Involvement of colleagues was fostered in case a permissive subculture was present at the workplace, which then functioned as an offender convergence setting.

2.5. Becoming and staying involved

Social bonds and money were both important for becoming involved and staying involved. The importance of social bonds for finding and involving new group members was exemplified by the snowball effect. Whereas persons in a bad financial situation were relatively easy to recruit, the promise of luxury was also hard to resist by those who were not in a bad financial situation. Greed appeared to be more often a reason to stay involved than a need for money. Sometimes, however, group members were not immediately given the full amount of criminal money they had earned, so as to make them continue to work for the group. Threats of violence were also frequently employed to make members who wanted to leave, stay in the group. In some cases, threats were not needed because group members felt obliged to stay due to being part of a subculture in which social bonds are strong and loyalty to the group is high.

2.6. Conclusion: synthesis of involvement mechanisms

From the analyses, several types of involvement mechanisms could be distinguished. Economic involvement mechanisms relate to the logistical nodes itself. Although logistical nodes are not meant to facilitate the trafficking of illicit products from one place to another, they do provide a breeding ground for organized crime. Without ports, organized crime groups would have to find different methods to traffic illicit products from one country to another. Therefore, the existence of a logistical node itself is a factor that facilitates

involvement in organized crime. The tension between speed and safety at logistical nodes also facilitates involvement in organized crime. Not all people and products can be equally thoroughly checked and more security checks implies lower processing speed which in turn results in less profit. Organized crime groups use this tension between speed and safety to their advantage.

Occupational and social involvement mechanisms relate to the occupational and social embeddedness of organized crime. Having a job at the airport could facilitate involvement in organized crime. Certain characteristics of jobs at the airport could make it easier to discretely traffic drugs or to explore new trafficking routes or smuggling methods. Jobs also enlarge one's social network. Moreover, persons added to one's social network through work, usually colleagues, are more likely to be useful as well to the organized crime group. Through the social snowball effect, persons recruited by organized crime groups recruit new persons from their social network and, this way, the cycle continues. It could therefore be stated that having criminal contacts increases the chance to become involved in organized crime.

Personal factors affect the likelihood of involvement in organized crime as well. Opportunities to become involved are not always seized by potential offenders. What makes potential offenders motivated for involvement depends in part on their personal circumstances. Examples of such personal circumstances are financial setbacks and a desire for thrill and excitement.

3. Part 1: Analysis of Criminal Careers

3.1. Introduction

The aim of this study is to describe the criminal careers of individuals involved in organized crime activities at some point in their lives. Furthermore, their criminal pathways are related to individual characteristics, such as gender and country of birth, and characteristics related to their criminal involvement, such as crime mix and time spent in prison.

3.2. Data

For the purpose of this study, information from the Dutch Organized Crime Monitor (OCM) is combined with information on criminal careers from the Dutch Offender Index (OBJD) (for more information on the OCM and organized crime in the Netherlands, see Kleemans, 2007). In four sweeps of the OCM, information of a wide cross-section of 150 cases concerning various forms of organized crime was collected. For 1,841 individuals (out of 2,305) involved in these cases, the entire criminal career could be reconstructed using judicial information from the OBJD (comparable to *rap sheets*). With age 12 being the minimum age of criminal responsibility in the Netherlands, the abstracts span juvenile and adult offending. Information is available up to 2016 or death (if this occurred prior to 2016). Abstracts also contain information on the timing and nature of offenses, as well as how the criminal case was adjudicated. Most of the individuals are male (90 percent) and a narrow majority was born in the Netherlands (53 percent, see also Table 1).

On average, offenders were 27 years old when their first judicial contact took place and they spent a total of 2.4 years in prison by the end of the observation period. The majority was involved in drug-related activities: traditional drugs (30 percent), synthetic drugs (9 percent) or both (19 percent). One out of five was involved in organized fraud. Eight percent of the individuals were involved in human smuggling and another eight percent in human trafficking. In their entire criminal career, they committed an average of just over five crimes.

3.3. Methods

Semi-parametric group-modelling is used to identify clusters of individuals with similar developmental pathways in crime over time (Nagin, 2005). We fitted a zero-inflated Poisson model to account for the fact that judicial contacts are relatively rare events, also in a sample of organized crime offenders. We controlled for censoring, caused by shorter observation periods during the follow-up period; unobserved years were coded as missing and do not contribute to estimating the model. Multiple trajectory analyses are carried out and both the Bayesian Information Criterion and probabilities of group

membership are used to determine the optimal model. Based on this optimal model, individuals are assigned to the trajectory where his or her posterior probability of group membership is highest.

3.4. Results

A six-group model was selected as the optimal model to fit the criminal careers of the 1,841 organized crime offenders. Figure 1 provides a graphical representation of the criminal careers of the six distinguished groups. Characteristics of each of the six distinct groups are presented in Table 2. Most individuals are assigned to a trajectory group labeled *very low-frequency offenders* (VLF, 53 percent). Individuals assigned to this group on average committed the smallest number of crimes (1.2) and spent the least amount of time in prison (1 year) from all groups. Also, of all groups the highest proportion women (14%) and individuals born abroad (56%) are among the VLF offenders. The criminal path followed by the *low-frequency offenders* (LF, 18 percent) is very similar to that followed by the VLF offenders, with the difference that LF offenders committed some more crimes during their career (4.8 on average) and spent an average of almost three years in prison.

The criminal pathways of three other groups, the *early bloomers* (EB, 8 percent), *low-frequency chronics* (LFC, 10 percent) and *late bloomers* (LB, 5 percent) have similar shapes, but they start and peak at different stages in life. While EBs have their first judicial contact around age 17 and LFCs around age 20, LBs do not start their career before age 30. In the same way, EBs reach their offending peak around age 20, the criminal careers of LFCs peak around age 30, and LBs commit the most crimes around age 45. In contrast to these differences, the total number of crimes committed (9 to 10) and the number of years spent in prison (3 to 4 years) is fairly similar for these three trajectory groups. The *high-frequency chronics* (HFC, 6 percent) are by far the most criminally active group. They do not only have the lowest onset age of all groups (16 years), but also committed the most crimes in their careers (25 on average) and spent the most time in prison (almost 9 years on average).

3.5. Conclusions

The analyses show that individuals involved in organized crime at some point in their lives follow diverse pathways in crime. While the majority of individuals starts offending in adulthood and offends at a low rate, a smaller proportion of individuals starts offending in adolescence and shows higher offence rates.

3.6. References

Kleemans, E.R. (2007). Organized crime, transit crime, and racketeering. In M. Tonry, and C. Bijleveld (eds.), Crime and justice in the Netherlands. Crime and justice. A review of research, vol 35 (pp. 163-215). Chicago: University of Chicago Press.

Nagin, D.S. (2005). Group-based modeling of development. Cambridge, Mass: Harvard University Press.

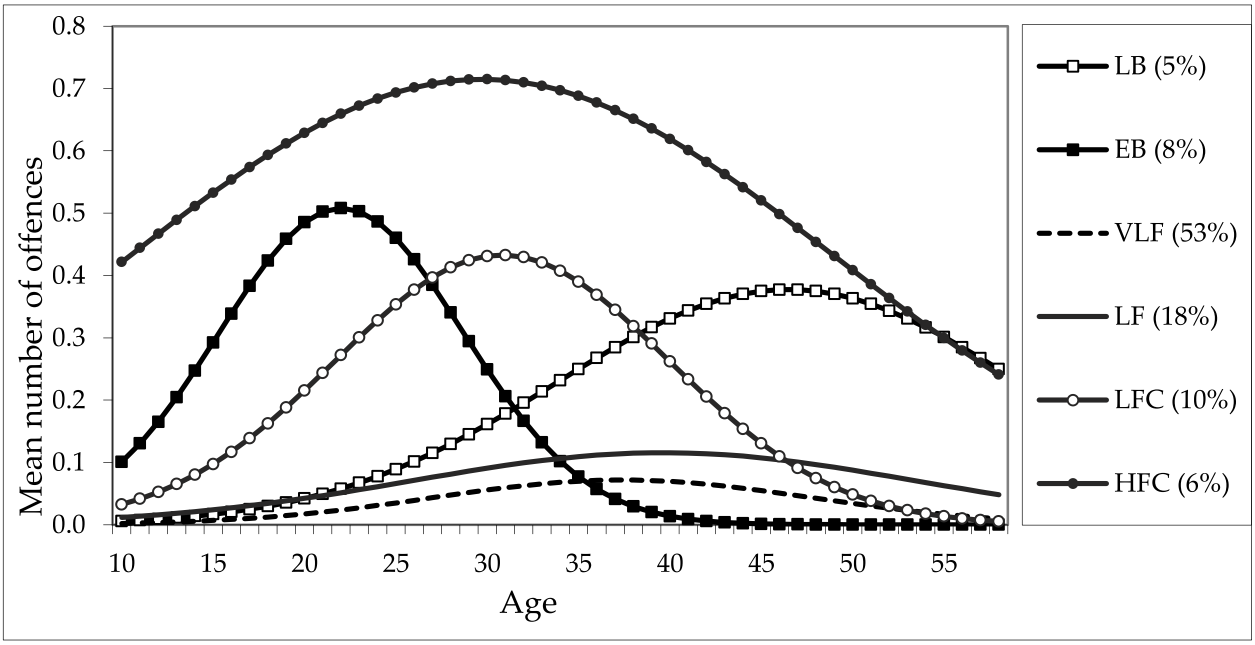

Figure 1. Trajectory groups

Late bloomers (LB); early bloomers (EB); very low-frequency offenders (VLF); low-frequency offenders (LF); Low-frequency chronic offenders (LFC); High-frequency chronic offenders (HFC)

Table 1. Descriptives total sample

	% / Mean (SD)
Male	90%
Born in the Netherlands	53%
Year of birth	1963 (10.5)
Onset age	27.2 (10.4)
Last crime in dataset	43.6 (11.1)
Follow-up age	52.8 (10.3)
Time spent in prison (years)	2.4 (3.8)
Type of organized crime	
Traditional drugs	30%
Synthetic drugs	9%
Traditional + synthetic drugs	19%
Human smuggling	8%
Human trafficking	8%
Fraud + money laundering	21%
Other	5%
Total number of crimes in career	5.3 (7.2)
Violence	0.6 (1.5)
Sexual offences	0.02 (0.2)
Property	1.4 (3.1)
Vandalism, public order	0.6 (1.1)
Drugs	0.9 (1.6)
Traffic	0.8 (2.0)
Other	1.0 (1.9)

Table 2. Descriptives differentiated by trajectory group

	Late bloomers (N=96)	Early bloomers (N=144)	Very low-frequency offenders (N=971)	Low-frequency offenders (N=324)	Low-frequency chronic offenders (N=192)	High-frequency chronic offenders (N=114)
	Mean (SD) / %					
Type of organized crime						
Traditional drugs	35%	38%	27%	32%	39%	26%
Synthetic drugs	13%	17%	8%	8%	13%	8%
Traditional + synthetic drugs	22%	19%	17%	18%	18%	32%
Human smuggling	2%	6%	10%	10%	4%	1%
Human trafficking	7%	3%	9%	9%	4%	4%
Fraud + money laundering	17%	9%	25%	20%	15%	15%
Other	4%	7%	4%	3%	7%	15%
Male	99%	97%	86%	92%	96%	99%
Born in the Netherlands	80%	64%	44%	45%	68%	82%
Year of birth	1951 (8.4)	1968 (8.4)	1963 (10.7)	1964 (11.0)	1964 (8.9)	1962 (7.0)
Onset age	29.7 (6.6)	16.9 (3.1)	30.9 (10.2)	27.9 (10.5)	20.1 (4.3)	16.2 (2.7)
Last crime in dataset	57.6 (8.4)	40.8 (8.1)	42.2 (11.1)	41.4 (11.6)	46.5 (8.1)	49.7 (7.2)
Follow-up age	63.1 (8.5)	47.9 (8.3)	52.9 (10.4)	52.2 (10.7)	51.6 (8.9)	52.8 (7.2)
Time spent in prison (years)	4.1 (4.8)	3.1 (3.7)	1.0 (1.7)	2.9 (3.5)	3.9 (4.3)	8.7 (6.0)
Total number of crimes in career	10.4 (5.7)	8.7 (4.6)	1.2 (1.5)	4.8 (3.2)	9.7 (4.1)	25.3 (9.9)
Violence	0.8 (1.2)	1.2 (1.8)	0.1 (0.4)	0.3 (0.9)	1.1 (1.4)	3.8 (3.3)
Sexual offences	0.1 (0.6)	0.03 (0.2)	0.003 (0.06)	0.009 (0.1)	0.03 (0.3)	0.03 (0.2)
Property	1.9 (2.4)	2.8 (3.1)	0.2 (0.6)	0.9 (1.6)	2.1 (2.2)	8.7 (6.9)
Vandalism, public order	0.9 (1.1)	1.0 (1.3)	0.2 (0.4)	0.9 (1.1)	0.9 (1.1)	2.4 (2.2)
Drugs	2.0 (2.1)	1.0 (1.4)	0.2 (0.5)	1.3 (1.7)	1.3 (1.6)	3.0 (3.4)
Traffic	2.6 (3.3)	1.1 (1.7)	0.2 (0.6)	0.2 (0.7)	2.2 (2.4)	3.1 (4.6)
Other	2.1 (2.5)	1.5 (1.8)	0.3 (0.6)	1.1 (1.7)	2.0 (2.4)	4.2 (3.5)

4. Part 2: Traffickers in Transit: Analysing the Logistics and Involvement Mechanisms of Organised Crime at Logistical Nodes in the Netherlands

Empirical results of the Dutch Organised Crime Monitor

4.1. Introduction

As demonstrated by the analysis of mobile bandits in the eighteenth century (Blok, 2001) or Italian mafia families that evolved in the nineteenth century (if not earlier; Paoli, 2004), organised crime is not a new phenomenon. Nevertheless, only in the past decades the topic of organised crime has been researched more systematically. This research led to new insights into involvement mechanisms and criminal trajectories in organised crime. For example, Van Koppen, De Poot, Kleemans, and Nieuwbeerta (2010) demonstrated that, unlike high volume crime offenders, organised crime offenders are generally older and have a later onset of offending. Researchers have also shed light on factors that stimulate people to engage in organised crime, like deviant group morals and financial setbacks (e.g., Kleemans & De Poot, 2008; Kruisbergen, Van de Bunt, & Kleemans, 2012; Van Koppen, 2013). Moreover, while positive life events, like having a job and being married, are often considered to have a desisting effect on engagement in high volume crime, Kleemans and De Poot (2008) noted that positive life events not always lead to desistance from engagement in organised crime. On the contrary, family ties and jobs could in fact promote involvement in organised crime. Jobs and family provide the social structures allowing criminals to find potential co-offenders. Jobs could also facilitate organised crime activities if the jobs encompass a relatively high level of autonomy, mobility, or status (Kleemans & Van de Bunt, 2008).

Research regarding involvement mechanisms and criminal careers in organised crime has thus demonstrated that factors related to engagement in crime differently affect organised crime offenders as compared to high volume crime offenders. Even though a clearer picture of involvement mechanisms and criminal trajectories in organised crime is now emerging, Von Lampe (2012) notes several aspects of organised crime research that deserve more scrutiny. First, Von Lampe states that most research on transnational organised crime focuses on destination countries, whereas the nature of criminal groups and activities in source and transit countries has been less studied. Second, Von Lampe elaborates that in-depth analyses of the modus operandi of criminals and the logistics of transnational organised crime activities are the exception

rather than the rule. Finally, Von Lampe explains that if coherent theoretical frameworks are to be developed, researchers have to compare different aspects of organised crime across different contexts (e.g., contexts defined by type of crime). In order to do so, however, researchers have to examine clearly demarcated contexts rather than general categories that encompass a diffuse range of different types of crime.

The present study addresses each of these points by studying transnational organised crime in the Netherlands. The analyses specifically focus on one type of organised crime, namely drug trafficking, and highlight the context of one important logistical node, namely airports. Airports are pivotal in the free flow of goods and citizens across the world, but they are also a crucial facility for transnational organised crime. The Netherlands functions in this context as an important transit country (Kleemans, 2007). Transit crime can best be described as international illegal trade. Although knowledge on organised crime offenders engaging in transit crime has accumulated recently, systematic analyses of offenders operating on logistical nodes are still rare. This report offers such analyses. The empirical data consists of sixteen cases of the Dutch Organised Crime Monitor. Although the main analyses focus on organised crime at airports (eleven cases), organised crime at seaports (five cases) is analysed as well in order to compare the logistics and involvement mechanisms at the airport with the seaport.

This report is structured as follows: First, a brief overview of the current state of the literature on involvement mechanisms in organised crime is presented and the main logistical nodes in the Netherlands are described. Then, the data and methods used for the analyses are outlined. In the results section that follows, the results of the analysis of smuggling methods employed by organised crime offenders and the logistics of their activities are first presented. Subsequently, the results are presented of the analysis of involvement mechanisms and the social and occupational embeddedness of the criminal careers of the same offenders. The report concludes with a summary of factors that affect involvement in organised crime and a conclusion and discussion of the results.

4.2. Becoming involved in transit crime

In this chapter, involvement mechanisms in organised crime are first discussed. Five general types of involvement mechanisms are explained and exemplified. Specific job characteristics that increase the likelihood of becoming involved in organised crime are discussed as well. The second part of this chapter elaborates upon the nature of organised crime in the Netherlands as well as two settings that are highly relevant to organised transit crime, namely the main airport in Amsterdam and the main seaport in Rotterdam. In the third part of this chapter the process of international drug trafficking is described using crime script analysis.

4.3. Involvement mechanisms in organised crime

The social and occupational embeddedness of organised crime provide important clues on how people become involved in organised crime. Five established involvement mechanisms are: Social ties, work ties, deliberate recruitment, leisure activities and sidelines, and life events (Kleemans, 2012). Social ties are an important factor for becoming involved in organised crime. In the world of organised crime, no formal mechanisms such as the law or third-party insurances exist to consolidate trust if transactions do not work out as planned. Criminal groups operate in a hostile and uncertain environment and, as often argued in organised crime research, therefore need other mechanisms to consolidate trust (Von Lampe & Johansen, 2004). Kleemans and Van de Bunt (1999) argue that social bonding is a solution to this problem because of the temporal and network embeddedness. Temporal embeddedness of social ties implies that the likelihood of cheating decreases when offenders know they will meet each other in the future and have information about each other's past. Network embeddedness implies that group members have information about one another and that they have a reputation to uphold in their network. Temporal and network embeddedness of social ties also apply to work ties at the work place. Colleagues see each other regularly and have to work together for a longer period of time. Furthermore, colleagues form a network in which information about one another is easily shared. Colleagues therefore have a reputation to uphold which facilitates trust in the long run. The same line of reasoning could be applied to persons who frequently meet because they, for example, practice the same sports, have the same hobby, or visit the same pub (Van de Bunt & Kleemans, 2007). While criminal group members sometimes recruit specific persons due to their connectedness, another reason to recruit specific persons is that they possess specific knowledge or skills valuable to the criminal group. Such deliberate recruitment could also be aimed at persons who are in a vulnerable position, for example due to negative life events such as financial setbacks. Negative life events at a later age are known to increase the likelihood of people becoming involved in organised crime (Kleemans, 2012). Positive life events could also increase the likelihood of becoming involved in organised crime by creating more possibilities for involvement. Examples of positive life events increasing the likelihood of involvement are getting married and becoming employed.

Legitimate jobs of organised crime offenders are often characterised by one or more of the following factors: autonomy, mobility, and frequent social contact (Kleemans & Van de Bunt, 2008). The more autonomy a person has, the less he or she is directly supervised, and the easier it is to engage in organised crime activities. In a similar way, the more mobile an employee is, the harder it is to directly supervise him or her, and thus the easier it is to engage in organised crime activities. A high level of autonomy and mobility both make it easier to conceal criminal activities by making it appear as if these activities are part of licit duties. Truck drivers are, for example, very mobile. So when they transport drugs, they do not immediately raise suspicion because drug

transports look very similar to licit transports. Finally, jobs involving frequent social contact increase the likelihood of criminal group members connecting to potential offenders. This is the case for, for example, shop owners, doorkeepers, and market traders. Employees who are very mobile or have a highly social job are also more likely to frequent so-called 'offender convergence settings' (Felson, 2006). These settings are places where offenders meet potential offenders and start criminal activities together, such as cafés and casinos (Le & Gilding, 2014). Offender convergence settings demonstrate that the place where people work also affects the chance of them becoming involved in organised crime. Because organised crime in the Netherlands often encompasses transit crime, persons working at logistical nodes in the Netherlands could be of great interest to organised crime groups. The most important logistical nodes in the Netherlands are described in the next paragraph.

4.4. Transit crime and logistical nodes in the Netherlands

Many types of organised crime in the Netherlands can be described as transit crime. The major businesses of organised crime groups in the Netherlands boil down to international smuggling activities. In these activities, the Netherlands could function as a destination country, a transit country or, in the case of cannabis and synthetic drugs, a source country (Kleemans, 2007). In its function as a transit country, the Netherlands is an important 'hub' for the distribution of drugs to the rest of Europe. Recent reports of the European Monitoring Centre for Drugs and Drug Addiction (EMCDDA) demonstrate that cocaine import into Europe mainly takes place through Spain and Portugal in the south and through the Netherlands and Belgium in the north (EMCDDA, 2016). The Netherlands is also an important source country for synthetic drugs such as MDMA and amphetamines since a lot of know-how on the production is available in this country (EMCDDA, 2015). The significance of the Netherlands as a transit point, or starting point, for drug traffickers relates to its demographical, geographical and logistical characteristics (Kleemans, 2014). Due to its colonial past and government policies in the eighties and nineties, the Netherlands has a relatively large number of inhabitants with roots in countries or regions where drugs such as cocaine, heroin, and hash are produced on a large scale. Because of their roots, these inhabitants are often better able to connect to these countries or regions and can therefore be the key between foreign-based crime groups and crime groups based in the Netherlands. In other words, their roots enable them to bridge 'structural holes', i.e. to form a connection between (parts of) networks that would otherwise be poorly connected due to geographical and social barriers (Burt, 1992). For example, Turkish persons are generally better able to connect to Turks in Turkey than Dutch persons with no foreign roots. If a criminal group residing in the Netherlands wants to import heroin from Turkey, a person well-connected to both the Netherlands and Turkey is very valuable to that group. Finally, because of its geographical position, the Netherlands is sometimes considered the gateway to Europe. Two important logistical nodes contribute to

the gateway-function of the Netherlands, namely the main airport and seaport. These logistical nodes are discussed next.

Amsterdam Airport Schiphol

Amsterdam Airport Schiphol is the largest airport in the Netherlands and one of the largest in Europe, with approximately 58 million passengers and 1.6 million tonnes of airfreight being processed in 2015 (Schiphol, 2017). While its enormous processing capacity and its connectedness to other parts of the world make Schiphol crucial to the Dutch economy, these factors also make exploitation of the airport attractive to criminal groups. Because such large volumes of persons and goods are processed, not all passengers and airfreight can be thoroughly checked, thereby facilitating cross-border drug transports. Several security measures have been put in place, however, in response to large-scale drug trafficking at Schiphol. For example, in 2001, security checks of personnel working at the airport increased. Before the implementation of security checks, personnel going from land- to airside and vice versa could relatively easily smuggle drugs. Due to the implemented security checks, luggage of personnel had to pass similar security procedures as luggage of passengers. Another example concerns the so-called ‘100%-checks’ that have been enacted since 2003 to tackle the increasing flow of drug mules smuggling cocaine into the Netherlands (House of Commons, 2010; Kruisbergen, 2005). All passengers and their luggage on flights from specific countries have to pass extra security checks (Kleemans, Soudijn, & Weenink, 2010).[1]

Port of Rotterdam

The Port of Rotterdam is one of the largest seaports in Europe. Just like the main airport of the Netherlands, the Port of Rotterdam is an important hub for organised crime groups (EMCDDA, 2016). Between 2009 and 2012, approximately forty tonnes of cocaine entering the Port of Rotterdam were seized (Eventon & Bewley-Taylor, 2016). In 2013, the Dutch police estimated that a quarter to half of the cocaine consumed in Europe enters via the Port of Rotterdam (Van der Ploeg, 2014). Similar to the airport, the seaport has several security systems in place aimed at detecting illicit traffic. For example, sea containers are randomly scanned and shipping-agents have to show certain documents before they can operate in the port.[2]

---

[1] This does not mean that every passenger is subjected to exactly the same type of checks (Van Haaften, 2013, p. 2).

[2] A specific modus operandi employed by organised crime groups is the ‘rip-off’ (Eski & Buijt, 2016). Characteristic of a rip-off is that criminal groups transport drugs to other countries by using sea containers ordered by legitimate companies as their transport vehicle. Sea containers are transported as ordered by the legitimate companies and upon arrival, the criminal group takes out the drug packages before they or the packages are noticed.

4.5. International drug trafficking: a short crime script

The accumulation of knowledge on topic of drug trafficking through both scientific research and federal investigations has made it possible to discern several key procedures in the process of drug trafficking. In so-called crime scripts, these sets of action are schematically organised in a chronological framework (Cornish, 1994). In other words, crime scripts clarify what occurs before, during, and after a crime is committed. Such scripts also encompass actors, equipment and locations required for crime commission. Even though different crime groups could use different equipment or locations, or change their modus operandi in response to police interventions, the flexibility of crime scripts allows for such improvisations. One of the benefits of crime scripting is that it makes the different steps in the process of crime commission easier to examine (Chiu, Leclerc, & Townsley, 2011). By doing so, crime scripting aids situational crime prevention, which focuses on environmental aspects that facilitate crime rather than on personal characteristics of perpetrators (Clarke, 1997). As for international drug trafficking, a typical crime script classified into four main activities is exemplified in Table 1 (Tompson & Chainey, 2011). The content of this crime script is based on the results of analyses of cases of the Dutch Organised Crime Monitor (see also chapter 3).

Table 1: International drug trafficking crime script.

Main activity	Key procedures	Actors, equipment, and location
Preparation	Acquiring drugs	Buying drugs from others<br>Producing drugs by yourself
Pre-activity	Transporting drugs to a logistical node	Borrowing a car<br>Using acquaintances to drive
Activity	Getting drugs on board (on the vehicle)	Drug mules<br>(Air)port employees<br>Customs officials<br>Cover materials
	Getting drugs off the vehicle	
Post-activity	*Transporting drugs to destination*	*Borrowing a car*<br>*Using acquaintances to drive*
	Stashing drugs	*House*<br>*Shed*
	Selling drugs (sometimes after further refinement)	
	Handling financial flows	*Spending cash*<br>*Money laundering*

Drug traffickers using logistical nodes such as an airport or seaport face several challenges. For example, because of the large volume of passengers and cargo processed criminal groups have to know exactly at what time and place "their" mule, luggage or container arrives. Furthermore, they have to make sure that their contraband passes security checks safely. To this end, they often need insider's help. Criminal groups could corrupt law enforcement personnel in order to evade security checks or they could corrupt port employees to provide them with information about the arrival of sea containers with drugs. Organised crime groups are thus sometimes dependent on

involvement of employees working at logistical nodes. But how do these employees become involved in organised crime? The logistical bottlenecks drug traffickers have to solve (step 3 and 4 in table 1) as well as the involvement mechanisms explaining the participation in organised crime are further elaborated in chapter 4. First, however, the data and methods used are discussed in chapter 3.

4.6. Data and methods

For the present study, a selection of cases from the Dutch Organised Crime Monitor (OCM) is used. The OCM is an ongoing research project since 1998 aimed at gaining insight into organised crime in the Netherlands. The main sources of this research project are files of closed Dutch police investigations of criminal groups engaging in a wide variety of criminal activities. All cases in the OCM result from long-term police investigations, often spanning a period of several years. The police files constitute a rich source of data and include, amongst others, the results of special investigation techniques (e.g., wiretapping and observation) and transcripts of suspect interrogations. These files are systematically summarised using an extensive checklist that has expanded throughout the years (Kruisbergen, Van de Bunt, & Kleemans, 2012).

From a total of 180 cases in the OCM, a subset of sixteen cases was selected for the purpose of this study. Because the focus of this study is drug-related organised crime at major transit points in the Netherlands, all drug cases were initially selected. Subsequently, cases in which the airport or seaport(s) played no role, or only a minor role, were filtered out. Other criteria employed to select cases were the richness of information with regard to trafficking operations as well as the recency of the cases. Eleven cases concern drug trafficking through Amsterdam Airport Schiphol, five cases concern drug trafficking through the seaport of Rotterdam and/or other seaports in the Netherlands or neighbouring countries.
The sixteen cases together include 286 offenders. The smallest case includes four offenders, the largest case includes 49 offenders. Four cases involve less than ten suspects, seven cases involve ten to twenty suspects, and five cases involve more than twenty suspects. The majority of offenders (90%) are male. In only one case, the main suspect was female. The timeframe in which the police investigations started ranges from 1991 up to 2012.

As for the nature of the criminal activities, in twelve cases cocaine was exported from South America to the Netherlands. In five cases XTC (‘ecstasy’) was exported from the Netherlands to other countries, such as England, Australia, and the United States of America. In two cases other drugs than cocaine and XTC were transported as well.[3]
To analyse the logistics of organised crime activities, information regarding the modus operandi of criminal groups was gathered from the cases. To analyse

[3] Please note that these numbers do not add up to sixteen because in several cases the suspects transported more than one type of drugs.

the occupational and social embeddedness of organised crime activities, information on topic of jobs, the legal work place, and social relationships were gathered. All information was subsequently categorised. Examples of categories are the type of airport personnel involved, the type of smuggling method employed, and the reasons offenders gave to smuggle drugs. The analyses focused primarily on the airport cases. The seaport cases were used to enrich the data. Important differences in results between the airport and seaport are discussed separately.

The remainder of this report is structured as follows: First, a brief overview of the criminal structures is provided. Furthermore, the logistics of criminal activities are discussed. Subsequently, the occupational and social embeddedness are discussed as well as personal reasons for offenders to become and stay involved in organised crime. Finally, a list summarising the involvement mechanisms is provided after which the report concludes with a general conclusion and discussion.

4.7. Empirical results

SHORT DESCRIPTION OF CRIMINAL NETWORKS

The criminal structures encountered in the studied cases are best described as criminal networks consisting of smaller criminal groups. Separate groups often had their own trade, but co-operated with other groups if it helped them in their trade. The following three cases exemplify differences in network structures.

- Case 1: several groups worked together, sometimes on ad-hoc basis, sometimes more structurally. Each group had a core with co-offenders surrounding the core members. Most group members were related through family ties.
- Case 2: two separate groups could be distinguished. Each group had a leader. Only the leaders and other high ranking members in each group communicated with each other. The two leaders, a man and a woman, once had a romance. The romance began after the woman moved in with the man. After she became involved in his drug trade, she involved her own family members as well, which is how the second related group came to exist.
- Case 7: two criminal groups were initially one criminal group as nearly all members in the network were employed in the same team of cargo handlers. After a fight between two important members, these members refused to work together any longer and split up the network in two distinct groups. A few members of the groups remained in touch, however, to communicate about criminal activities.

In most cases, members had several different types of tasks. For example, a member could have executive tasks and coordinating tasks or more managerial tasks. Most groups also had a leader or multiple leaders. Suspects

knew their place in the group. In groups that were largely based on family ties, the oldest family member in the group was usually in charge.

4.8. Logistical bottlenecks: getting the drugs through

In this section, the logistics of drug trafficking operations at the airport are described. Central to this section is the question how drugs can be smuggled across country borders without the smugglers getting caught through security checks. Three types of tactics to traffic drugs without getting caught are discerned: (1) defying security checks, (2) avoiding security checks, and (3) neutralising security checks. When criminal groups attempt to defy security checks, they do pass security checks but hope not to get caught. When avoiding security checks, smugglers do not pass security checks at all, but rather circumvent them. When attempting to neutralise security checks, offenders pass the checks, but try to make sure they will not be subjugated to checks. Before these tactics are discussed, a remark has to be made with regard to the scope and limitations of our analyses. First, due to the sensitivity of the topics discussed, examples of smuggling methods will be described with a limited level of detail. Second, discussion of a specific smuggling method does not imply that it is (still) an effective smuggling method. Although some offenders are very innovative in their use of smuggling methods, some methods might be rather dangerous (i.e., there is a high chance of detection). Furthermore, certain smuggling methods discussed, either techniques to hide drugs or the abuse of privileges by airport personnel, might now be rendered less effective because of new or increased levels of security measures.

DEFYING SECURITY CHECKS

The most common method to defy security controls was the use of drug mules. Drug mules were used in eight cases. In four out of eight cases, drug mules swallowed drug capsules. In each of these four cases, the criminal group tried to smuggle drugs into the Netherlands. In six out of eight cases, drug mules carried luggage with drugs into the plane with them. In two cases, the criminal groups deployed both mules that swallowed drug capsules and mules that carried luggage with drugs. Sometimes criminal groups simply hoped that their mules would not be checked and considered a caught mule as a business risk (case 6). Other criminal groups took precautions to try to prevent the drugs from being noticed on scanner images.

Methods aimed at security scanners were employed to defy security checks in six cases. For example, in case 10, drug mules swallowed coins in order to try to hide the drug capsules they swallowed when going through a scanner at the airport. Air freight in which drugs was hidden was frequently tampered with so as to make the packages appear normal when passing security scanners. For example, in case 3, carbon paper was used in boxes filled with drugs intended to make the boxes appear empty when going through a scanner. To send larger batches of drugs, criminal groups hid drugs in larger machines. To make transportation of these large machines appear legitimate, criminal groups

registered front companies, so they could send or receive the products in name of the company.

Case 13: The criminal group transported drugs in large machines such as woodworking machines. The machines were prepared in Europe and then flown to South America to be filled with drugs. From there, they were transported back again to Europe either by boat or by plane. To make transportation of the machines appear legitimate, the group registered a front company on paper specialised in woodworking. Although the machines became useless after preparing them for drug transports, this loss of value was more than compensated by the value of drugs transported.

AVOIDING SECURITY CHECKS

Security checks were mostly avoided by people working at the airport, such as cargo handlers and technicians, who (ab)used their job-related personal credentials and privileges for drug trafficking. For instance, in four cases, computer systems were exploited to find flight details and, if a drug mule had been arrested, passenger details. Flight details were consulted to acquire information about, amongst others, at which time flights with drugs mules and drug packages would arrive, and in which container the luggage was placed. Passenger lists were consulted to ensure that a drug mule had indeed been arrested and had not just run away. When employees did not have access to a computer system, other employees with log-in credentials helped them out.
Also in four cases, the criminal group exploited vehicles of companies operating at the airport to transport packages and luggage with drugs. With these company vehicles, they could drive discretely from the airplane or basement to other places at the airport where they would hand over the package to a co-offender, or even drive off the airport.

Case 9: Suspect A was employed by company E and could therefore use company E's vehicles. A drove with these vehicles on both landside and airside. Suspect B worked at the airport for another company, but was not able to use their vehicles at the airport. Therefore, B was asked to monitor the airport area while A left the airport area with drugs hidden in a vehicle from company E. Once A had left, B left the airport area as well. Together they drove to another location where they transferred the drug packages from company E's vehicle to B's car.

People working at the airport also abused their security badge for criminal purposes. The abuse of a personal security badge occurred in six cases. A personal security badge is provided to employees working at the airport so they can move from one section to another in order to carry out their duties. However, this freedom of movement also provided employees with many opportunities to engage in criminal activities.
Another recurring method to avoid security checks was the manipulation of luggage labels. In case 9, manipulated labels were sent to exporting countries where the labels were attached to luggage with drugs. Upon arrival in the Netherlands, cargo handlers could then easily pick up the luggage.

Most of the studied criminal groups searched within their social network for persons with job-related privileges at the airport in order to avoid security checks. However, the criminal group in the following case ensured that her members acquired job-related privileges by establishing a company of their own at the airport.

> *Case 11: Cleaning company D was owned and run by suspect G and his son H. With their company, they acquired the same privileges as other janitors working at the airport. They abused their access to airplanes and information systems of the airport. G was also in touch with different criminal groups that wanted to use (criminal) services offered by G's company. Furthermore, if drug packages had to be stored safely at the airport, G sometimes hired other persons working at the airport for 600 euros.*

Next to abusing job-related privileges and credentials, criminal groups also exploited the (technical) complexity of airplanes to avoid security checks. In two cases (2, 11), drugs were smuggled across borders by hiding drug packages in the airplane. The exporting group made sure the drugs were well hidden in the airplane and sent instructions to the importing group about where to find the drugs. Sometimes "line testing" was done by hiding a distinct object in the airplane that was to be found by the importing group. Line testing had two purposes. First, by retrieving the object, the exporting group knew that the instructions were received and correctly interpreted by the importing group. Second, the exporting group was ensured that the importing group could actually get the object out of the airplane without getting caught. The necessity of line testing and the complexity of airplanes are best illustrated in case 2 where even the police could not find the packages with drugs hidden by the exporting group:

> *Case 2: Janitors on Curacao hid drug packages in the airplane. The first time, however, the importing group could not find the packages because they were looking in the wrong place, just like the police. Subsequently, the janitors on Curacao planned to send a teddy bear as a test to see if the importing group could find the bear.*

The criminal group in case 7 hid luggage with drugs in airfreight containers. Because air freight containers are relatively large and therefore have a lot of storage space, the importing group received instructions from the exporting group about where the luggage with drugs was to be found.
Finally, criminal groups also attempted to transport drugs away from the airport area at the moments of which they believed activity of security personnel was low.

> *Case 9: Suspect J told the police during an interrogation that luggage with drugs was often transferred at specific times, because "you knew when there were customs officers". If luggage with drugs would arrive at an inconvenient moment, then the luggage would be stored for a while. Storing luggage was avoided at places with a lot of sniffer dogs.*

NEUTRALISING SECURITY CHECKS

Security checks could be neutralised by corrupting customs (or other) officers at Schiphol or at the airport of the source country to let drug mules pass through security checks. Available information in several cases indicate that corrupted officers might have been involved.[4] For example, in one of the cases an arrestee testified that customs officers received pictures of the mules beforehand so the mules would be recognised during security checks. Having contacts within customs, however, does not guarantee a "smooth" operation, as some cases involve disagreement on the amount of money a corrupted officer should receive.

DIFFERENCES BETWEEN AIRPORT AND SEAPORT

The results regarding the modus operandi of criminal groups discussed so far apply largely to both the airport and seaport. However, differences were also noted between countermeasures deployed to defy or avoid security checks at the airport and countermeasures deployed at the seaport. These differences largely pertain to the difference in general use of the ports. The main function of the seaport is facilitating the flow of goods and not the flow of persons. Conversely, the airport has to process an enormous amount of persons next to processing enormous volumes of goods.

First, drugs were frequently transported by drug mules in airport cases. However, in seaport cases, investigative units only uncovered drug transports in which drugs were covered up by deck cargo or hidden in sea containers. In none of the seaport cases persons were caught carrying drugs with them, even though in at least one seaport case corrupted personnel sailing across the sea was discovered. Because there is no (large) intercontinental flow of persons at seaports such as the ones in Rotterdam or Antwerp, criminal groups operating at seaports have to rely more on the flow of goods. Therefore, secondly, criminal groups using seaports more often used (front) companies to have a legitimate reason to hire sea containers or to legitimise container transport.

REQUIRED SKILLS AND ASSETS

The results so far demonstrate that criminal groups mostly relied on either drug mules or on job-related credentials, privileges, and knowledge to defy or avoid security checks. For example, because airplanes are technically complex, group members need to be familiar with the construction of airplanes to know where to hide drug packages. They need to know what places are large enough to store the packages and are relatively easy to open up without causing noticeable damage. If criminal groups attempt to traffic drugs in luggage without accompanying passengers, they need members who can get hold of this luggage and transport the luggage from airside to landside. In this case, the group needs members with privileges which only people working at the airport have. In brief, most personal assets required to traffic drugs at the

---

[4] Although some cases include information that civil servants, such as customs or police officers, might be involved, the cases often lack sufficient information to prove such involvement.

airport are job-related assets of people working at the airport. The role of these job-related assets, or more generally, the occupational embeddedness of organised crime activities, is discussed in the following section.

4.9. Occupational embeddedness

How do jobs facilitate organised crime? Kleemans and Van de Bunt (2008) argue that certain jobs bring persons in a better position to contribute to organised crime activities. Legitimate jobs of persons engaged in organised crime are often characterised by a relative high level of autonomy and mobility. Jobs with a high level of autonomy are often found in, amongst others, companies aiming to deliver quick services. Their workers are provided with a high level of independency and less supervision, like janitors (at airports) and truck drivers. Truck driving is also a typical example of jobs with high mobility, which makes truck drivers more suitable to transport illegal goods without raising suspicion. When individuals become involved in organised crime, it is likely that their criminal activities relate to their legal jobs. For example, a truck driver transporting legal products can easily (also) transport illegal products. Besides, the truck driver could use his or her practical knowledge and contacts from his or her legal activities as well (Van de Bunt & Kleemans, 2007; Kleemans & Van de Bunt, 2008). Not only do jobs provide the logistics and social contacts to contribute to organised crime, having the same job also increases trust amongst co-offenders since they know about each other's reputation. Furthermore, they can keep an eye on each other when working together (Kleemans & Van de Bunt, 2008). How job-related factors affect engagement in organised crime is discussed in this section. The following five factors are elaborated upon: (1) the similarity between legitimate job duties and criminal activities, (2) autonomy, (3) mobility, (4) job-specific knowledge acquired through job-related privileges, and (5) social capital of employees.

When analysing the jobs of organised crime offenders in the studied cases, a distinction can be made between civilian personnel and law enforcement personnel. Examples of civilian personnel encountered in the cases are stewards, janitors, technicians, and personnel engaged in (coordination of) cargo handling. Law enforcement personnel encompass customs and police officers. In the majority of cases, civilian personnel was found to abuse job-related privileges and knowledge to facilitate drug trafficking. In one case, a member of a criminal group became employed at the airport, but the group was arrested before he could abuse his position.

One of the job-related factors facilitating engagement in organised crime was the similarity between licit job duties and criminal activities. Because cargo handlers frequently have to move luggage from one place to another, moving luggage with drugs did not raise suspicion. In a similar way, technicians have to inspect airplanes from the inside. Therefore, moving ceilings, removing screws, and checking the water tanks when looking for hidden drugs all appeared to be part of regular duties. In general, it could be stated that employees with mainly executive duties in their legitimate job also mainly carried out executive tasks in their role of criminal group member. Employees

with legitimate coordinating and supervising duties often had more diverse types of tasks in the criminal group. For example, the director of a cleaning company in case 11 supervised criminal group members, but also engaged in logistic activities. A municipal clerk in case 14 gave the criminal group judicial advice, which relates to his legitimate job, but also transported drugs a few times. Finally, the similarity between criminal activities and legitimate job duties also applied to law enforcement personnel. For example, in case 2, criminal group members said that they had corrupted a customs officer, so he would fake checking the luggage of a mule.

A relative high level of mobility and autonomy are also advantageous for engagement in organised crime activities. Due to high mobility, stewards had plenty of opportunities to traffic drugs to and from different countries themselves and they could explore new routes to expand their drug trade. Because cargo handlers were very mobile, they had plenty of opportunities to transport luggage with drugs from one side to the other side of the airport without raising suspicion. A high level of autonomy allowed janitors and technicians to smuggle drug packages from airplanes, cargo handlers to discretely separate luggage with drugs from licit luggage, and directors to access computer systems at the airport. A high level of autonomy also facilitated changes in work schedules advantageous to the criminal group. In one of the cases, the manager of corrupted employees stated in an interview with the police that the employees frequently changed their work schedule. Employees were more or less free to switch schedules with other employees. In the following example, the coordinator of a team of cargo handlers made sure that the right employees were scheduled to work at the right time so they could easily take out luggage with drugs.

> *Case 7: Suspect K was a coordinator at a luggage handling company at the airport. He was therefore able to create or adjust work schedules so that cargo handlers involved in drug trafficking would be present at the time luggage with drugs arrived. K, however, was not a high ranking member in the criminal group. Therefore, K only initially designed the work schedule according to the criminal group's wishes. Later, group members and cargo handlers L and M created the work schedule themselves while K only had to turn a blind eye.*

This case also demonstrates how mobility, job-related privileges, and the similarity between job duties and criminal activities facilitate drug trafficking at the airport:

> *Case 7: Cargo handlers working in the luggage handling basement took luggage with drugs out of the container. One of the cargo handlers then carried the luggage to landside while pretending it was regular luggage. At a public road on landside, he handed the luggage over to another member of the criminal group. Because the road was busy, the luggage could discretely be transferred. The other member then drove the luggage out of the airport area.*

Another job-related factor facilitating engagement in organised crime is job-specific knowledge acquired through job-related privileges. For instance,

knowing at which place and time luggage arrives and where that luggage has to be transported to, is job-specific knowledge. To acquire this information, cargo handlers had to have access to computer systems in which information about the arrival of airplanes can be retrieved. Examples of other privileges resulting in job-specific knowledge are using company's vehicles in order to transport luggage or entering airplanes in order to clean them on the inside. These privileges, or duties, result in knowledge such as knowing how to manoeuvre airport vehicles and how airplanes are (roughly) constructed. It appeared from the studied cases that such knowledge is very useful to criminal groups attempting to smuggle drugs into airplanes and from airplanes, with or without luggage.
Finally, social capital of employees working at the airport mainly encompasses other contacts working at the airport or contacts that used to work at the airport. The following case demonstrates the usefulness of contacts at the airport:

> *Case 2: Suspect N was supposed to pick up luggage with drugs at the airport. This luggage, however, had already been taken away by the police. While N was looking for the luggage, he ran into one of his contacts working at the airport. This contact was able to access the computer information systems and could see that the luggage had been taken away by the police. N informed a higher ranking member, suspect O, about the police seizure.*

To summarise, the occupational embeddedness of organised crime activities is manifested through five job-related factors. Autonomy, mobility, and the similarity between legitimate duties and criminal activities all mainly facilitate discrete engagement in organised crime activities during work time. Job-specific knowledge makes a person interesting to organised crime groups because this type of knowledge is rather scarce yet very useful to groups operating at logistical nodes. Similarly, social capital acquired through work also makes a person interesting to organised crime groups because people who already work at a logistical node are not always easy to find. The usefulness of social capital in organised crime is further elaborated in the next section.

4.10. Social embeddedness

In this section, the relationship between group members and the role of settings in which these relationships are formed are examined in greater detail. First, the role of ethnicity and the types of relationships between offenders are discussed. Subsequently, subcultures and offender convergence settings encountered in the cases are discussed.

Organised crime groups cannot rely on the law or mediation to prevent (the escalation of) conflicts. Therefore, trust, proximity, and having a shared past and future are important factors in finding new members (Beckert & Wehinger, 2011; Kleemans & Van de Bunt, 1999; Loughran, Nguyen, Piquero, & Fagan, 2013; Von Lampe & Johansen, 2004). New offenders are thus often persons with whom group members already have a social bond. Typically, offenders look for potential co-offenders in their circle of friends, relatives, and

acquaintances. This often leads to greater ethnic homogeneity within criminal (sub)groups. However, as Kleemans and Van de Bunt (1999, p. 25) note: “The basis of criminal associations is not formed by ethnicity, but by the social relations that exist between various persons. This applies to both immigrant and native offenders.” Social bonds are indeed an important factor in the search for co-offenders (Morselli, 2003; Beckert & Wehinger, 2011).

A type of settings that foster strong social bonds concerns subcultures. Members of subcultures in which strong cohesion is present also demonstrate high loyalty towards the group (Kleemans, Brienen, & Van de Bunt, 2002). Certain subcultures increase the likelihood of members to engage in crime, if that subculture endorses deviating norms that approve of, or even promote, crime (Kruisbergen, Van de Bunt, & Kleemans, 2012). With the combination of a high degree of loyalty, strong cohesion, and deviating norms, subcultures provide a pool of potential co-offenders to criminal groups. Newcomers to subcultures or groups endorsing criminal norms are then socialised into criminal practices (Anand, Ashforth, & Joshi, 2005).

Another type of setting that facilitates the introduction of new members is the so-called ‘offender converge setting’ (Felson, 2006). An offender converge setting is a place where co-offenders meet with each other or with potential co-offenders to discuss criminal activities. Examples of such places are market places and cafés, but the legal workplace could also function as an offender converge setting (Kruisbergen, Van de Bunt, & Kleemans, 2012; Kleemans & Van de Bunt, 2008).

In most of the studied cases, the ethnicity of group members varied greatly. In only two cases, criminal groups were completely ethnically homogeneous. In cases with mixed ethnicities, ethnicity was more homogeneous within subgroups than between subgroups. This is exemplified by case 1:

> *Case 1: Group 1 consisted of two Lebanese men, group 2 and 3 each consisted of two Surinamese men, and group 4 consisted of two Turkish men. There was also a Colombian man who received drugs from a Surinamese and another Colombian man. The drugs were subsequently sold to a Greek, a Moroccan, an Algerian, and a native Dutch man.*

Differences in tasks were also related to differences in geographical origin between subgroups. In cases 2 and 3, for example, a subgroup originating from Israel and a subgroup originating from the Netherlands could be discerned. In both cases, the Israeli subgroup was more engaged in drug trade while the Dutch subgroup was more engaged in manufacturing drugs. In case 11, most members had a Turkish background, but mainly non-Turkish members served as brokers. Brokers are persons in a network who connect groups that would otherwise be unrelated (Burt, 1997). Through their ethnic background, non-Turkish members were better able to connect to criminal groups in countries other than the Netherlands and Turkey, which in turn expanded the drug trade of the whole criminal network.

One of the reasons ethnic homogeneity was greater within (sub)groups is that offenders frequently introduced family members as new co-offenders. The two cases in which the criminal group was completely ethnically homogeneous

were both family organisations, that is, nearly all members were relatives of each other. As Kleemans and Van de Bunt (1999) noted, social relations are of greater importance than ethnicity in the recruitment process. The importance of social relations over ethnicity is exemplified in case 8. A difference in ethnic backgrounds was noted in this case between drug mules and money mules. Money mules were all considered trustees by the leader of the criminal group. These money mules were also closely related to his family and they had the same ethnic background as the leader. The drug mules, on the contrary, were not closely related to the leader and had all sorts of ethnic backgrounds. The drug mules merely engaged in drug trafficking to earn some extra money.

In most cases, twelve out of sixteen, family ties existed amongst members. In at least six of the twelve cases, romantic partners or ex-partners co-operated in the criminal group. Besides family ties, most criminal groups also included friendships. In most cases, the relationship between recruiters and their friends and acquaintances dated years back. In a few cases it was noted that proximity was also an important factor. New group members either lived close to the recruiting group members, or they visited the same (local) stores or restaurants as the recruiting group members. In case 5, one of the suspects slowly got involved after she met one of the members who lived in an apartment above the restaurant she was working for. In case 13, one of the suspects stated that he met many of the group members years ago in the restaurant he owned back then. Finally, in case 15, suspect A met suspect B by accident when he was shopping in suspect B's food store.
Besides consulting family members or friends, criminal groups also attempted to recruit persons with certain job-related privileges. In twelve out of sixteen cases, group members consulted either direct colleagues or ex-colleagues to search for new group members.

> *Case 11: Suspect D was in charge of a cleaning company at the airport which he used as a front company to cover his drug trafficking operations. D met one of his fellow suspects a long time ago when they both owned a bar and did business together. Later on, when D became a janitor at the airport himself, he met another four of his fellow suspects. D got his five former colleagues involved in his drugs operations when starting his cleaning company.*

The introduction of new group members could also be facilitated by a permissive subculture among several members of airport personnel. In one of the cases, a person involved in the criminal investigation noted that theft of travellers' properties and drugs trafficking frequently occurred because it was a very lucrative activity, whereas the chance of getting caught was rather low (see also Schelvis & Lub, 2008).[5]

Offenders not operating in permissive subcultures sometimes met potential co-offenders in offender convergence settings. A typical offender convergence setting is jail. In three cases, group members established contacts in jail,

[5] Our data does not allow us to determine to what extent a permissive subculture is indeed present. Furthermore, as explained in section 4.2, increased security measures could have affected the pervasiveness of such a subculture.

which they used later when their criminal group needed new group members. In case 15, the market place functioned as an offender convergence setting where new contacts were established. The advantage of the market place is that it attracts people who are in the trade business and thus already possess skills and knowledge desired by criminal groups.

To conclude, persons were most likely to be introduced to criminal groups if they already had a social bond with group members, whether it was through old friendships, jobs, or proximity. The most common type of social bonds encountered in the cases concerned family ties and job-related ties.

4.11. Becoming and staying involved in organised drug trafficking

Occupational and social embeddedness are important factors in understanding the context in which people become involved in organised crime. However, the fact that a person is employed as a cargo handler does not automatically imply that this person wants to engage in drug trafficking. Similarly, a person does not join a criminal group simply because he or she has a criminal niece. Therefore, in this section, personal circumstances and motivations that cause people to become and stay involved in organised crime are discussed.

Becoming involved

From the studied cases, a plethora of personal motivations and circumstances could be distilled that could lead to engagement in organised crime. The most prominent personal motivations and circumstances are discussed here. First, in both the occupational and the social setting, criminal groups attempted to recruit new members that were in a vulnerable position. For example, in case 7, cargo handlers who trafficked drugs looked for potential co-offenders by first getting to know their colleagues better and establishing a friendship with them. Group members then had conversations with their colleagues about their home situation and tried to find out how susceptible they were to engagement in organised crime. If they found a weak spot, that colleague would subsequently be persuaded to join the criminal group.

Financial setbacks are one of the types of negative life events that often emerged as a motivation to become involved in organised crime. In several cases, suspects told the police that they had financial issues (in general) or that they had debts due to a gambling or drugs addiction. Financial issues were one of the weak spots criminal groups tried to find in new members. In case 3, a group member persuaded one of his family members who was in a bad financial situation to become involved in organised crime.

> *Case 3: Suspect P told the police that he was approached by suspect Q to become involved in drug trafficking. P states: "One night, we were having dinner with my family and Q's family. At some point, Q said he wanted to discuss something with me. After dinner, Q asked me if I wanted to go abroad to escort drug mules. He asked me how I was doing. I told him that I was in a bad financial situation and that I agreed*

> *to go abroad." P also stated that "he felt he had no choice because he thought that the money would clear his debts. Moreover, his engagement would be limited to escorting drug mules to other countries."*

Vulnerability due to a bad financial situation was also prominent in immigrants who had just arrived in the Netherlands. Living in poverty after migrating to the Netherlands was recurrently claimed to be a motivation for especially those originating from the Dutch Antilles.

> *Case 10: Suspect Z migrated to the Netherlands, but did not obtain a steady job and received no welfare payment. Therefore, he did odd jobs for suspect A, such as doing the groceries and taking the children to school, but also criminal activities related to drug trafficking. When he was released from prison, he found a job. However, after six months, he became unemployed again. Z's financial situation went from bad to worse and he became involved in A's drug trafficking business again.*

It was also noted that some suspects received social welfare payment in the Netherlands while investing their drugs money in houses and expensive cars in the Dutch Antilles. Indeed, several suspects seemed to become involved simply because they wanted a more luxurious life.

> *Case 7: Suspect R told the police about his involvement in drug trafficking: "They just asked me so that I could do more things with my children… You can earn 6000 euros and then you start to consider your options seriously."*

Whereas suspect R seemed to suggest that the amount of money makes people think seriously about involvement, other suspects suggested that they were in fact blinded by the amount of money. As a suspect in case 6 stated: "I know the kind of scene I entered and it is about a lot of money, so you know what those people do for all that money."
In a few cases, involvement in organised crime resulted from romance. In the following examples, a group member started a relationship with someone who was in a bad financial situation after which the new partner slowly became involved in the criminal group.

> *Case 10: As stated by a police officer: suspect X involved her partners in her organised crime activities. When X and Y became involved in a romantic relationship, he started to work for her by carrying out illicit activities for her criminal group. However, she did not always pay him because, as she told him, she already paid his rent and gave him food.*

Case 2 demonstrates the social snowball method that often describes the introduction of new group members. The social snowball method is a repeated process in which group members search for new members in their network and those new members do the same when they have become involved in the group (Kleemans, 2012). Sometimes, the snowball method was deployed to find people with specific assets the criminal group needed for their illicit activities. Case 2 exemplifies the snowball effect whereby (ex-)colleagues were used to find potential co-offenders.

Case 2: Suspects H and her brother I approached suspect J because they heard that he had worked at the airport. J knew people who were still working there and could smuggle luggage with drugs into the plane. J then connected H and I to someone he knew who could arrange that those people would smuggle the drugs into planes.

Finally, several suspects admitted that they initially engaged in drug trafficking for thrill and excitement. In case 14, one suspect engaged in drug trafficking because he was attracted to criminal activities and the lucrative business of the group. Other suspects in this case stated that they liked to be around the highest ranking member. Being in his presence enhanced their status in their environment. In another case, case 7, one of the suspects told the police: “I found it very exciting because I knew it was forbidden. On the other hand, I also knew that I could earn a lot of money with it.” This suspect also said that he had a debt of several thousands of euros.

STAYING INVOLVED

Money and social ties are important factors for becoming involved in organised crime. These factors are also important, however, in staying involved in organised crime. In case 2 one of the highest ranking members, suspect B, played ‘divide and rule’ with his group members. According to his group members, B paid them too little and used them. Because they were dependent on his money, however, they remained loyal to the group and to him. Many social ties existed among the members as well, ranging from direct family and family-in-law to friendships. Similarly, in case 13, one of the suspects stated that he only continued his involvement because he was not yet paid the full amount of money he had earned for his involvement: “If I would receive all the money, I would not work with drugs anymore.”

Drug mules living in poverty are not only easy bait for criminal groups, but they are also easy to keep involved. Drug mules might start smuggling to pay off their debts or to escape poverty. However, they are also made dependent by initially lending them money which they later have to pay back by smuggling drugs. Another tactic to ensure the mules do not run away is to wait until they have successfully smuggled the drugs across borders and then pay them afterwards. Nonetheless, criminal groups preferred to involve mules that were already connected to criminal group members because, as explained before, social ties promote trust in each other.
Next to a need for money, greed was another motivation to stay involved. Case 5 exemplifies the attraction of luxury:

Case 5: The main reason for suspect S to traffic drugs was to sustain his luxurious lifestyle. S liked to be the centre of attention. It was one of his dreams to become co-owner of a dancing club. S probably paid criminal money to fulfil this dream as he became co-owner during the police investigation. Suspect T told the police that S had a Ferrari and a BMW that suspect U took away from him because S owed U money. U had a very profitable club and was so rich that he actually did not need to traffic drugs for money.

Threats were another method deployed by criminal groups to force their members to stay involved. In several cases, higher ranking members threatened lower ranking members, if the latter tried to leave the group or even if they complained.

> *Case 12: Suspect C tells the police that he had to come to a restaurant because of rumours about unsatisfied members. In the restaurant, he met three higher ranking members. According to C, tensions rose during the conversation and they accused him of complaining. At some point, one of the three higher ranking members grabbed a knife on the table and said: "If something like this happens again, I will cut your throat."*

Sometimes, threats directed at members who tried to leave the group were more subtle. A suspect in case 9 said that, after he had made clear he wanted to leave the group, thieves entered his house and took away many of his belongings. Another suspect also wanted to leave because his involvement made him too nervous. He told the police:

> *Case 9: "Several nights before the first shipment, I was unable to sleep. I continued, however, because D said "You will continue". He said that face-to-face. I felt threatened and forced. Eventually, I told D a story, namely that from February 2008 on, surveillance on the airport would be stricter. That way I tried to quit. (…) They never threatened me, but I felt threatened because of the way they acted. For example, I never told them where I lived, yet they did show up at my house."*

Group members were not only threatened with violence when they tried to leave the group, but also with notifying the authorities of the illegal activities these group members had already engaged in.

> *Case 16: Suspect B was head of a spy shop and approached suspects C and D to offer them an IT-related job. Because both were interested, B challenged them to demonstrate their IT-skills. The police presume that suspect B subsequently used either C and D's demonstration or their financial rewards to pressure them into developing malware that would be used to neutralise the port's digital security systems.*

Finally, often a high degree of loyalty and strong cohesion exists between members of (criminal) subcultures (Kleemans, Brienen, & Van de Bunt, 2002), which makes it harder to leave the criminal group.

In sum, money and social bonds are amongst the most motivating factors fostering involvement in organised crime. Criminal groups often rely on the snowball method when searching for new members in their social network. Persons who are in a bad financial situation are relatively easily recruited by criminal groups. However, a strive for luxury might also be a reason to become involved and stay involved in organised crime, because large amounts of money can be earned quickly through criminal activities. Threats of violence, either implicit or explicit, were the primary method deployed by several criminal groups to keep group members, who attempted to leave the group, involved. However, sometimes threats were not needed because group members felt obliged to stay due to being part of a subculture in which social bonds are strong and group loyalty is high.

4.12. Synthesis: factors leading to involvement in organised crime

This chapter outlines the main factors facilitating involvement in organised crime groups operating at important logistical nodes in the Netherlands. The factors are distilled from the results described in the previous chapters.

In the introduction, it was explained that the Netherlands is an important transit country for international drug trafficking. Several types of drugs are traditionally imported in the Netherlands from various regions around the world, such as cocaine from South America and heroin from Turkey and other countries. Once in the Netherlands, the drugs are further distributed into Europe. The Netherlands also functions as an important source country for synthetic drugs. Both importers and exporters largely rely on the main airport and seaport in the Netherlands to smuggle their drugs across borders. Economic factors facilitating involvement in organised crime at logistical nodes are described first. Then, occupational, social and personal factors facilitating involvement are outlined. Occupational factors encompass factors related to work and are based on the analysis of the occupational embeddedness of organised crime activities. Social factors encompass factors related to social ties and are based on the analysis of the social embeddedness of organised crime activities. Finally, personal factors encompass personal circumstances and motivations. These factors are based on the analysis of personal involvement mechanisms.

Economic factors

Although this might perhaps seem counterintuitive, the licit world in fact enables the illicit world to flourish. Without logistical nodes, organised crime groups would have to find different methods to traffic their drugs from one country to another. The main air- and seaport in the Netherlands process large volumes of goods and people. Because of these large volumes, it is impossible to thoroughly perform security checks on each passenger and each product. Furthermore, more security checks imply lower processing speed which in turn results in less profit for ports and companies operating at the ports. Organised crime groups use this tension between speed and safety to their advantage.

Economic factors	Example
Existence of logistical node	Airport or seaport.
Tension between speed and safety	Deliberate recruitment of personnel at the airport who have to pass less security checks when entering or leaving airside. Alternatively, piggybacking by hiding drugs in sea containers of legitimate companies with a good reputation that have to pass security checks less often.

Please note that these economic factors cannot be (fully) eliminated. Nevertheless, these factors do provide a breeding ground for organised crime activities and are therefore listed here.

OCCUPATIONAL AND SOCIAL FACTORS

Having a job at the airport could facilitate involvement in organised crime. Certain characteristics of jobs at the airport could make it easier to discretely traffic drugs and to explore new trafficking routes or smuggling methods. Jobs also enlarge one's social network. Moreover, persons added to one's social network through work, usually colleagues, are more likely to be useful as well to the organised crime group. Through the social snowball effect, persons recruited by organised crime groups recruit new persons from their social network and so the cycle continues. It could therefore be stated that having criminal contacts increases the chance to become involved in organised crime.

**Occupational factors**	**Example**
Autonomy	Managers could adjust work schedules, so criminal group members are scheduled to be at the right place at the right time to pick up luggage with drugs.
Mobility	Stewards could observe security checks at different airports to explore new trafficking routes.
Similarity between licit job duties and criminal activities	Cargo handlers frequently have to move luggage from one place to the other. Therefore, it does not raise suspicion if they move luggage around with drugs hidden in it.
Job-related privileges and personal credentials	Janitors have freedom of movement at airside, which facilitates drug smuggling.
Job-specific knowledge	*Technicians are knowledgeable on technical details of airplanes. This knowledge enables them to hide drugs in airplanes and find hidden drug packages.*

**Social factors**	**Example**
Usefulness of social ties	*Criminal group members recruit a person working at the airport who can then introduce them to other airport employees with specific knowledge or privileges sought after by the criminal group.*
Romance	*Related to the usefulness of social ties: Romantic involvement with a criminal group member could facilitate criminal involvement.*
Criminal co-workers or norms at work	*The presence of criminal co-workers increases the likelihood of becoming involved in organised crime. Under certain conditions, the workplace could develop into a so-called offender convergence setting.*
Cohesion in subgroups	*Stimulating effect: Being part of a subculture where cohesion is strong and loyalty to the group is high, could increase the chance of becoming or staying involved in organised crime.*<br>*Diminishing effect: Social pressure in subgroups with strong cohesion between members could also prevent a person from becoming involved in organised crime.*
Ethnicity	*Although ethnicity is less important than social ties, ethnicity could be considered a precursor to social bonding: the more people have in common, the more likely they are to bond. Ethnic groups could also be considered subgroups in which cohesion could be strong. In the analysed cases, most criminal networks were ethnically heterogeneous whereas the group cores were largely ethnically homogeneous.*

PERSONAL FACTORS

Situational factors create opportunities for involvement in organised crime. These opportunities are more likely to be seized, however, by potential offenders who are already interested in involvement or easy to motivate to become involved (Clarke, 1997). What makes potential offenders motivated depends in part on their personal circumstances. Personal circumstances and motivations distilled from the analyses of the OCM cases are outlined here.

**Circumstances and motivations**	**Example**
Financial setbacks	*Being in a bad financial situation, for instance due to addictions and/or debts, makes potential offenders easier to recruit. Especially immigrants living in poverty and connected to source regions of drug production are relatively easily recruited.*
Strive for luxury	*Criminal money is sometimes quick and easy money. Potential offenders could be seduced by the amount of money offered and forget to think about the negative consequences of criminal activities if they simply desire more luxury or greater wealth.*
Thrill and excitement	*Potential offenders could be attracted to criminal activities, sometimes simply because it is forbidden.*

5. Conclusion and discussion

The present study encompassed analyses on two organised crime topics. Based on in-depth analyses of cases from the Dutch Organised Crime Monitor, this study explored how drug traffickers operate at important logistical nodes in the Netherlands. Furthermore, the social and occupational embeddedness of organised crime activities of these drug traffickers were analysed.
As for logistical operations, three types of tactics to traffic drugs without getting caught were discerned. First, to defy security checks, criminal groups most often deployed drug mules or methods that were supposed to avoid unusual shapes (of drugs) on scanner images. Second, to avoid security checks at all, criminal groups attempted to recruit persons with job-related personal credentials or privileges through which drugs could be smuggled without passing security checks. Therefore, deliberate recruitment was aimed at persons employed at logistical nodes such as (former) colleagues. Finally, to neutralise security checks, criminal groups corrupted customs or police officers. In exchange for their help, officers received money and pictures of drug mules so they would know which persons not to check.
The occupational embeddedness of organised crime activities was manifested through job-related factors. Autonomy, mobility, and the similarity between legitimate duties and criminal activities facilitated discrete engagement in organised crime activities during work time. Mobility also facilitated exploration of new trafficking routes. Legitimate duties and criminal activities were more similar in employees with executive functions than in employees with other types of functions, such as managing or coordinating functions. Social capital and job-specific knowledge both acquired through work are two other factors that make port employees more interesting to organised crime groups. These

types of contacts and knowledge are rather scarce yet very useful to groups operating at logistical nodes.

Persons with whom group members already had a social bond were more likely to be introduced to the criminal group. The snowball effect came into play here. Current members searched for potential offenders in their network and once involved, the new group members did the same. The most common types of social bonds encountered in the cases were family ties and job-related ties such as ties with colleagues. Involvement of colleagues was fostered in case a permissive subculture was present at the workplace which then functioned as an offender convergence setting.

Finally, personal motivations and circumstances leading to involvement in organised crime were examined. Social bonds and money were both important in becoming involved and staying involved. The importance of social bonds for finding and involving new group members was exemplified by the snowball effect. Whereas persons in a bad financial situation were relatively easy to recruit, the promise of luxury was also hard to resist by those who were not in a bad financial situation. Greed appeared to be more often a reason to stay involved than a need for money. Sometimes, however, group members were not immediately given the full amount of criminal money they had earned so as to make them continue to work for the group. Threats of violence were also frequently employed to make members who wanted to leave stay in the group. In some cases, threats were not needed because group members felt obliged to stay due to being part of a subculture in which social bonds are strong and loyalty to the group is high.

Dilemmas for (air)port security

Logistical nodes and companies operating at the nodes are key in the prevention of organised crime. However, decision makers involved in security issues face several dilemmas. Three of these dilemmas are outlined here: the tensions between personnel's autonomy and mobility on the one hand and security on the other, the tension between swift handling procedures and security, and the corruption dilemma.

Autonomy and mobility is more or less necessary for the flexible fulfilment of the job duties of several types of airport personnel, such as cargo handlers. However, autonomy and mobility also create opportunities to engage in criminal activities. Reducing the autonomy of airport personnel and increasing supervision on their activities could reduce security risks, but could also lead to less flexible and more time consuming procedures. This dilemma relates to the following, more general dilemma between speed of handling procedures and security.

An obvious way to reduce opportunities for drug trafficking is to increase security checks. However, increased security checks often result in less swift handling procedures for transportation of persons and cargo. Due to the economic importance of and the competition within the transportation sector, speed of handling procedures is an essential asset for airports.

One possible ingredient of a policy aiming at higher levels of security is to hire more security personnel. However, security personnel could be corrupted. It is a security officer's duty to encounter criminal activities and offenders, but this also opens up opportunities to become involved in crime (Carter, 1990). Furthermore, criminal groups might put more effort in trying to *neutralise* (corrupt) security personnel when increased security checks make it more difficult to simply *defy* or *avoid* those checks (see section 4.2).

It is because of these dilemmas alongside the large demand for and supply of drugs, the profitability of drug trade as well as the resourcefulness of offenders, that fighting drug trafficking is a truly 'wicked problem' (Rittel & Webber, 1973). This does not imply that we should just take drug trafficking for granted. The dilemmas involved in security policies, the extent of the drug trafficking problem, as well as the potential threat that organised crime poses to society in general, requires that governments, law enforcement agencies and private companies are constantly engaged in finding better solutions. Scientific research can contribute by providing an empirical base for policymakers.

Directions for future research

The introduction of highly automated processes has greatly changed the way things work at the ports in the last decades. However, the effect of automation on the modus operandi of criminal groups is less obvious from the studied cases. Although some criminal groups tried to circumvent security systems or used automated processes to their advantage, automation played no significant role in the majority of cases. The lack of abuse of automated systems in most cases could be due to the timeframe in which investigation of these cases took place. The earliest investigation dates back to 1991, whereas the latest investigation only started in 2012. Because some of the studied cases are somewhat dated, it is unsure to what extent smuggling methods of currently operating criminal groups has changed. However, the results also demonstrate behaviours that are time-persistent, such as recruitment of persons in one's own social network and abuse of job-related privileges and knowledge. Nonetheless, future research should analyse new smuggling methods and compare to what extent fundamental changes, if any, have taken place due to the advancement of automated processes.

Another question raised by the data used concerns the generalizability of the results. All studied cases revolve around large logistical nodes in the Netherlands or neighbouring countries. It may well be that the size of a logistical node affects the types of smuggling methods employed by criminal groups as well as the ease with which these methods are employed. Alternatively, although the exact execution of smuggling methods may differ between logistical nodes, the general involvement mechanisms might not. Future research is therefore urged to compare smuggling methods and involvement mechanisms between different types of logistical nodes. Only then will it be possible to examine the generalizability of the current results.

Finally, the present study is also limited by selection bias. Not all criminal activities reach the attention of law enforcement and not all can be solved. In

this study, only solved cases were examined. Furthermore, only drug trafficking cases were examined. Even though the cases analysed do demonstrate great diversity in modus operandi and the nature of crime groups, future research is suggested to deploy different methods to research this topic. For example, researchers could interview organised crime offenders themselves. By interviewing offenders, it would be interesting to find out if and how occupational and social embeddedness of organised crime activities differs between different types of organised crime.

References

Anand, V., Ashforth, B. E., & Joshi, M. (2005). Business as usual: The acceptance and perpetuation of corruption in organizations. *The Academy of Management Executive*, *19*(4), 9-23. DOI: 10.5465/AME.2004.13837437.

Beckert, J., & Wehinger, F. (2011). In the shadow: Illegal markets and economic sociology. *Socio-Economic Review*, *11*(1), 5-30. DOI: 10.1093/ser/mws020.

Blok, A. (2001). *Honour and violence*. Malden, MA: Blackwell Publishers Inc.

Burt, R. S. (1992). *Structural holes*. Cambridge, MA: Harvard University Press.

Burt, R. S. (1997). The contingent value of social capital. *Administrative Science Quarterly, 42*(2), 339-365.

Carter, D. L. (1990). Drug-related corruption of police officers: A contemporary typology. *Journal of Criminal Justice*, *18*(2), 85-98. DOI: 10.1016/0047-2352(90)90028-A.

Chiu, Y. N., Leclerc, B., & Townsley, M. (2011). Crime script analysis of drug manufacturing in clandestine laboratories: implications for prevention. *The British Journal of Criminology, 51*(2), 355-374. DOI: 10.1093/bjc/azr005.

Clarke, R.V. (1997). Introduction. In: R.V. Clarke (ed.), *Situational Crime Prevention: Successful Case Studies* (pp. 1-43). Guilderland, NY: Harrow and Heston.

Cornish, D. B. (1994). The procedural analysis of offending and its relevance for situational prevention. *Crime Prevention Studies, 3,* 151-196.

Eski, Y., & Buijt, R. (2016). Dockers in drugs: Policing the illegal drug trade and port employee corruption in the port of Rotterdam. *Policing*, 1-16. DOI: 10.1093/police/paw044.

European Monitoring Centre for Drugs and Drug Addiction (2015). *Perspectives on drugs: Synthetic drug production in Europe.* Luxembourg: Publications Office of the European Union.

European Monitoring Centre for Drugs and Drug Addiction (2016). *Perspectives on drugs: Cocaine trafficking to Europe.* Luxembourg: Publications Office of the European Union.

Eventon, R. & Bewley-Taylor, D. (2016). *An overview of recent changes in cocaine trafficking routes into Europe.* Luxembourg: Publications Office of the European Union.

Felson, M. (2006). *The ecosystem for organized crime*. Finland: European Institute for Crime Prevention and Control, affiliated with the United Nations.

House of Commons – Home Affairs Committee (2010). *The cocaine trade: Seventh report of session 2009-10, Volume 1: Report, together with formal minutes*. United Kingdom: The Stationery Office.

Kleemans, E. R. (2007). Organized Crime, Transit Crime, and Racketeering. In M. Tonry and C. Bijleveld (eds.), *Crime and Justice in the Netherlands. Crime and Justice. A Review of Research. Volume 35* (pp. 163-215). Chicago: The University of Chicago Press.

Kleemans, E. R. (2012). Organized crime and the visible hand: A theoretical critique on the economic analysis of organized crime. *Criminology and Criminal Justice, 0*(0), 1-15 .

Kleemans, E. R. (2014). Theoretical perspectives on organized crime. In L. Paoli (ed.), *Oxford Handbook of Organized Crime* (pp. 32-52). Oxford: Oxford University Press.

Kleemans, E. R., Brienen, M. E. I., & Van de Bunt, H. G. (2002). *Georganiseerde criminaliteit in Nederland*. *Tweede rapportage op basis van de WODC-monitor*. Netherlands: Boom Juridische uitgevers.

Kleemans, E. R., Soudijn, M. R., & Weenink, A. W. (2010). Situational crime prevention and cross-border crime. In: K. Bullock, R. V. Clarke, and N. Tilley (eds.), *Situational Prevention of Organised Crimes* (pp. 17-34). United Kingdom: Willan Publishing.

Kleemans, E. R., & Van De Bunt, H. G. (1999). The social embeddedness of organized crime. *Transnational Organized Crime*, *5*(1), 19-36.

Kleemans, E. R., & Van de Bunt, H. G. (2008). Organised crime, occupations and opportunity. *Global Crime*, *9*(3), 185-197. DOI: 10.1080/17440570802254254.

Kruisbergen, E. (2005). Voorlichting: Doen of laten? Theorie van afschrikwekkende voorlichtingscampagnes toegepast op de casus van bolletjesslikkers. *Beleidswetenschap, 19*(3), 38-51.

Kruisbergen, E. W., Van de Bunt, H. G., & Kleemans, E. R. (2012). *Georganiseerde criminaliteit in Nederland*. *Vierde rapportage op basis van de Monitor Georganiseerde* Criminaliteit. Netherlands: Boom Lemma uitgevers.

Le, R. & Gilding, M. (2014), Gambling and drugs: The role of gambling among Vietnamese women incarcerated for drug crimes in Australia. *Australian & New Zealand Journal of Criminology, 0*(0), 1-18. DOI: 10.1177/0004865814554307.

Loughran, T. A., Nguyen, H., Piquero, A. R., & Fagan, J. (2013). The returns to criminal capital. *American Sociological Review*, *78*(6), 925-948. DOI: 10.1177/0003122413505588.

Morselli, C. (2003). Career opportunities and network-based privileges in the Cosa Nostra. *Crime, Law & Social Change, 39*(4), 383-418. DOI: 10.1023/A:1024020609694.

Paoli, L. (2004). Italian organised crime: Mafia associations and criminal enterprises. *Global Crime, 6*(1), 19-31. DOI: 10.1080/1744057042000297954.

Rittel, H. W. J. & Webber, M. M. (1973). Dilemmas in a general theory of planning. Policy Sciences, 4, 155-169.

Schelvis, M. L. & Lub, A. (2008). *De gelegenheid te baat nemen. Een verkennend onderzoek op vijf Europese luchthavens naar kwetsbaarheden in het luchtvrachtafhandelingsproces die kunnen leiden tot criminaliteit*. Netherlands: Expertisecentrum Luchthavens.

Schiphol (2017). *Amsterdam Airport Schiphol airport facts*. Retrieved May 1, 2017 from: https://www.schiphol.nl/nl/route-development/pagina/amsterdam-airport-schiphol-airport-facts.

Tompson, L., & Chainey, S. (2011). Profiling illegal waste activity: Using crime scripts as a data collection and analytical strategy. *European Journal on Criminal Policy and Research, 17*(3), 179-201. DOI: 10.1007/s10610-011-9146-y.

Van de Bunt, H. G. & Kleemans, E. R. (2007). *Georganiseerde criminaliteit in Nederland*. *Derde rapportage op basis van de Monitor Georganiseerde Criminaliteit*. Netherlands: Boom Juridische uitgevers.

Van der Ploeg, J. (2014, April 2). Belangrijke cocaïneroutes verlegd naar haven Rotterdam. *De Volkskrant*. Retrieved from: http://www.volkskrant.nl/magazine/belangrijke-cocaineroutes-verlegd-naar-haven-rotterdam~a3626470/.

Van Haaften, M. (2013). *Tien jaar 100%-controles op Schiphol. Evaluatie rapport*. Netherlands: Bureau Discriminatiezaken Kennemerland.

Van Koppen, M. V. (2013). Involvement mechanisms for organized crime. *Crime, law and social change*, *59*(1), 1-20. DOI: 10.1007/s10611-012-9396-8.

Van Koppen, M. V., & De Poot, C. J. (2013). The truck driver who bought a café: Offenders on their involvement mechanisms for organized crime. *European Journal of Criminology, 10*(1), 74-88. DOI: 10.1177/1477370812456346.

Van Koppen, M. V., De Poot, C. J., Kleemans, E. R., & Nieuwbeerta, P. (2009). Criminal trajectories in organized crime. *British Journal of Criminology, 50*(1), 102-123. DOI: 10.1093/bjc/azp067.

Von Lampe, K. (2012). Transnational organized crime challenges for future research. *Crime, Law and Social Change*, *58*(2), 179-194. DOI: 10.1007/s10611-012-9377-y.

Von Lampe, K., & Johansen, P. O. (2004). Organized Crime and Trust: On the conceptualization and empirical relevance of trust in the context of criminal networks. *Global Crime*, *6*(2), 159-184. DOI: 10.1080/17440570500096734.

Appendix A: The cases

Airport cases

1. Marijuana, cocaine, and heroin traded by a network consisting of several groups that used each other's facilities and traded drugs with each other. Marijuana was shipped in large amounts, whereas cocaine was transported in smaller amounts by plane to the Netherlands. A janitor at the airport used his privileges to smuggle drugs from the airport, sometimes using vehicles provided by the cleaning company. Besides luggage, other smuggling methods were swallowing drug capsules and, in the case of shipped drugs, hiding drugs in machines.

2. Cocaine imported from South America by two related networks that are connected through their leaders. Cocaine was smuggled into and from planes by personnel working at the airports. Corrupt customs officials received pictures of drug mules beforehand, so they would recognise them at the airport.

3. Dutch suspects producing XTC ('ecstasy') and Israeli suspects exporting this XTC. Drugs were hidden in toy boxes that were to be transported by plane. Some flights were avoided due to the scanners used at some airports.

4. A family organisation importing cocaine from Curacao using drug mules and air freight. In the latter case, cocaine was added to a soup and sent as a frozen package.

5. Export of XTC to the United States of America and Canada by stewards who took the pills with them in their personnel luggage. After surveillance had been increased at the personnel entrance, the group bought plane tickets to get to airside with the drugs and then hand over the luggage to the stewards.

6. Cocaine import from Curacao and the Dominican Republic by a network with many ties to the exporting countries. Drugs were smuggled by mules, customs officials were corrupted, and several air routes were avoided to evade security checks.

7. Cocaine imported from South America by a network of colleagues working in the (luggage handling) basement of the airport. Most suspects worked in the same team and paid their coordinator to turn a blind eye on the drug trade. Drugs were carried off the airport with vehicles available for their regular duties.

8. Export of XTC to Asia and Oceania and import of cocaine from South America. Money mules were close to the Israeli core members, whereas drug mules were less close to the core and differed greatly in ethnicity. While the

Israelis in this network were mainly occupied with trade, the Dutch were mainly occupied with producing drugs.

9. Cocaine imported by a network of colleagues working in the (luggage handling) basement of the airport. Luggage agents, transfer agents, and coordinators all engaged in the drug trade. Luggage labels were sent back to South America for re-use and luggage reports were falsified. The group used vehicles of airlines to transport drugs off the airport.

10. Cocaine imported from South America by a Dominican family organisation with a single mother being in charge. She bought plane tickets for drug mules. The drug mules swallowed capsules and coins or carried suitcases with metal to avoid detection by security scanners.

11. Cocaine imported from Suriname and the Dutch Antilles by a group of colleagues who were employed by a cleaning company established to traffic drugs. Through their duties, they had the privilege to enter airplanes and search for the hidden drug packages, and to check computer systems for information about specific flights. In addition, they sometimes borrowed airport vehicles from persons employed by airlines.

Seaport cases

12. Hashish trade by a network consisting of several groups that sometimes worked together on ad-hoc basis and sometimes more structurally. Ships were bought by front companies to transport hashish hidden in containers to and from the Netherlands. Sometimes the hashish was not directly brought into the harbour by ship, but first unloaded onto a smaller boat and then brought to land. After a transport had succeeded and the ship was no longer necessary to the group, the ship was sold again. Persons with jobs and/or skills related to sea transport were recruited, such as captains and electricians.

13. Cocaine trade by a family organisation. The group bought machines in Israel and the Netherlands, transported the machines as air freight to South America, filled them with cocaine, and then transported them back to the Netherlands by ship. Sometimes the Netherlands served as the destination point and sometimes as a transit point to further distribute the drugs in Europe.

14. Four interrelated groups that imported cocaine and hashish, and exported synthetic drugs such as XTC. Cocaine and hashish were hidden in containers and shipped from South America to the Netherlands. XTC was hidden in air freight to the United States of America. Some suspects had connections with shipping and transportation companies. One suspect worked in the container business.

15. Cocaine exported from South America to several European ports. Certain ports were avoided due to the type of security scanners in use there. Cocaine was hidden inside the pallets and masked by different types of covers, such as

bananas. The main suspect had established useful connections during his time in jail and through his trading company. Furthermore, XTC was exported to the United States through air freight.

16. Cocaine imported from South America by a large network that hacked a track and trace website to follow containers in which drugs were hidden. They also digitally intercepted pin codes to pick up the containers before the legitimately appointed truck drivers could do so. After unloading the drugs, they returned the containers to the legitimate companies that ordered the containers in the first place.

CHAPTER 4: Recruitment into Mafias: Criminal Careers of Mafia Members and Mafia Bosses (UCSC-Transcrime)

Authors: Ernesto Savona, Francesco Calderoni, Gian Maria Campedelli, Tommaso Comunale, Marco Ferrarini, Cecilia Meneghini

Table of contents

SUMMARY 193

1 BACKGROUND AND OBJECTIVES 195

2 DATA 198

2.1 THE DAP DATASET 198
2.2 THE CASELLARIO DATASET 199
2.3 THE PROTON MAFIA MEMBERS DATASET 200

3 METHODOLOGY 201

3.1 THE CRIMINAL CAREERS' PARAMETERS 201
3.2 ANALYTICAL STRATEGY 205

4 RESULTS: THE CRIMINAL CAREERS OF MAFIA MEMBERS 208

4.1 MACRO LEVEL: THE PARAMETERS OF THE CRIMINAL CAREERS OF MAFIA MEMBERS 208
4.1.1 DESCRIPTIVE STATISTICS OF THE CRIMINAL CAREERS OF MAFIA MEMBERS 208
4.1.2 THE TRAJECTORIES OF MAFIA MEMBERS 217
4.1.3 THE CRIMINAL CAREERS BEFORE AND AFTER RECRUITMENT 219
4.2 MESO LEVEL: MAFIA MEMBERS ACROSS THE TYPES OF MAFIAS 223
4.2.1 DESCRIPTIVE STATISTICS OF THE CRIMINAL CAREERS OF MAFIA MEMBERS BY TYPE OF MAFIA 224
4.2.2 DIFFERENCES IN THE CRIMINAL CAREER OF MAFIA MEMBERS ACROSS THE TYPES OF MAFIA: LOGISTIC REGRESSIONS 226
4.2.3 TRAJECTORIES OF MEMBERS ACROSS TYPES OF MAFIA 228
4.3 MICRO LEVEL: EXPLORING THE DRIVERS OF THE RECRUITMENT INTO THE MAFIAS 229
4.3.1 FACTORS DETERMINING THE AGE OF RECRUITMENT: OLS REGRESSIONS 230
4.3.2 FACTORS DETERMINING THE AGE OF RECRUITMENT: MULTINOMIAL LOGISTIC REGRESSIONS 232
4.3.3 FACTORS DETERMINING THE AGE AT RECRUITMENT IN THE FOUR MAFIAS: OLS REGRESSIONS 234

5 RESULTS: THE CRIMINAL CAREERS OF MAFIA BOSSES 236

5.1 MACRO LEVEL: THE PARAMETERS OF THE CRIMINAL CAREERS OF MAFIA BOSSES 236
5.1.1 DESCRIPTIVE STATISTICS OF THE CRIMINAL CAREERS OF MAFIA BOSSES AND UNDERBOSSES 236
5.1.2 THE TRAJECTORIES OF MAFIA LEADERS 238
5.1.3 THE CRIMINAL CAREERS OF LEADERS BEFORE AND AFTER RECRUITMENT 241
5.2 MESO LEVEL: MAFIA BOSSES ACROSS TYPES OF MAFIAS 244
5.2.1 DESCRIPTIVE STATISTICS OF THE CRIMINAL CAREERS OF MAFIA LEADERS BY TYPE OF MAFIA 245
5.2.2 DIFFERENCES IN THE CRIMINAL CAREER OF MAFIA LEADERS ACROSS THE TYPES OF MAFIA: LOGISTIC REGRESSIONS 247
5.2.3 TRAJECTORIES OF LEADERS ACROSS TYPES OF MAFIA 249
5.3 MICRO LEVEL: EXPLORING THE DRIVERS OF MAFIA LEADERSHIP 251
5.3.1 CHARACTERISTICS OF MAFIA LEADERS 251
5.3.2 CHARACTERISTICS OF MAFIA LEADERS BEFORE RECRUITMENT 253
5.3.3 CHARACTERISTICS OF MAFIA LEADERS DURING THEIR EARLY CRIMINAL CAREER 254

6 CONCLUSIONS 258

REFERENCES 263

Summary

This report presents the study of the criminal careers of Italian mafia members and mafia leaders, with a specific focus on recruitment. The analysis applies for the first time the criminal careers framework (firstly developed by Blumstein in 1986) to the population of individuals convicted for mafia offenses between 1982 and March 2017 provided by the Italian Ministry of Justice.

The analysis follows a three-level approach that focuses on the macro (whole population), meso (by types of mafia), and micro (individual level) dimensions of mafia members' criminal careers. The three-level approach aims at four main objectives:

1. exploring the main criminal characteristics of Italian mafia members and leaders, at the aggregate level and across different types of mafia
2. identifying distinct subpopulations of mafia members and leaders with common offending trajectories over their careers
3. analysing how criminal behaviour changes after recruitment and what are the factors leading to an early age recruitment
4. investigating the variables affecting the probability of becoming a mafia leader

The analytical strategy comprises descriptive statistics, Group Based Trajectory Modelling and regression models.

Results at the macro level show that mafia members and leaders have significantly different careers: mafia leaders commit more serious and diverse crimes over a longer time span. Despite this, five different offending groups can be identified both within mafia members and mafia leaders on the basis of the number of committed offenses. The groups detected in the two populations present symmetric evolutionary processes. At the meso level, Italian mafia organisations present some differences both in terms of criminal characteristics and when considering the identified subpopulations. However, statistical estimations indicate that differences are not relevant in defining the mafia affiliation. At the micro level, findings suggest that specific factors such as the high violence, low education level, high versatility and high escalation of offenders lead to an early age recruitment into the mafia. In particular, affiliates and leaders show a peculiar escalation pattern before the first mafia offense; subsequently, they report a stabilisation or de-escalation in the crimes they commit. Finally, regression models showed that offenders exhibiting a more serious criminal behaviour in early adulthood face a higher probability of becoming mafia leaders.

1 Background and objectives

Different theories of crime aimed at explaining criminal behaviour (Cullen, Agnew, & Wilcox, 2013). However, academic research in this field has only recently focused on organised crime (henceforth OC). Consequently, no criminological theory has so far offered exhaustive explanation of OC, most likely due to the various types of groups and activities involved (Kleemans, 2014). Nevertheless, theoretical contributions and recent empirical studies provide useful insights for understanding criminal careers and recruitment pathways to OC.

A recent line of research associated with the criminal career approach has been the study of recruitment pathways of individuals into criminal groups and/or OC, pointed out as an emerging theoretical issue in OC research (Kleemans, 2014). The criminal careers framework, first developed in 1986 by Blumstein and his colleagues, has long marked the criminological debate. A criminal career is defined as "the characterization of a longitudinal sequence of offenses committed by an individual offender" (Blumstein, Cohen, Roth, & Visher, 1986, p. 12). During the evolution of the paradigm, Blumstein and colleagues identified six parameters for investigating the criminal career of an individual. The three primary components are the participation (or prevalence), the frequency, and the duration; the ancillary parameters are the specialization, the escalation, and the intermittency.[1]

The criminal career framework encountered critiques and favour of researchers, stimulating a longstanding debate on the necessity of separate models to explain different aspects of offending (Petras, Nieuwbeerta, & Piquero, 2010).

On the one hand, the major critiques came from Gottfredson and Hirschi (1986, 1987, 1988). The two scholars considered both participation and frequency as underlying features of the individual's 'criminal propensity'. The criminal propensity approach, in contrast with the criminal career one, predicts that the correlates of participation and frequency are realisations of a single underlying propensity toward criminal behaviour. From their point of view, the aggregate age-crime curve[2] constant across offense, time and subgroup-could capture all the information necessary for understanding criminal careers

[1] For the definition and description of each parameter, see Chapter 3.

[2] The age-crime curve represents the number of individuals arrested of a certain age divided by the population of individuals at that age.

without distinguishing between participation and frequency (Blumstein, 2016, p. 299).

On the other hand, other scholars stressed the importance of the criminal career paradigm and its individual parameters. Farrington and Hawkins (1991) highlighted the utility of considering different aspects of the criminal career separately in seeking to understand and predict crime. Furthermore, Farrington (1992) pointed out that the difference between participation and frequency had to be considered as a key distinction within the criminal career framework. According to Farrington, while frequency of offending remains fairly constant over time, the peak in the teenage years of the aggregated age-crime curve reflects mainly variations in prevalence (1992, p. 521). Further empirical studies have analysed criminal career dimensions, with a particular focus on the relationship between participation and frequency of offending, with different outcomes (Nagin & Smith, 1990; Osgood & Rowe, 1994; Paternoster & Triplett, 1988; Rowe, Osgood, & Nicewander, 1990). Petras et al. (2010) provided an accurate empirical assessment on the participation-frequency distinction through the examination of data on criminal convictions of a large sample of Dutch offenders (almost 5,000 people) across 60 years. Through longitudinal data, the study of the age-crime curve at the individual level revealed that "participation and frequency are linked closely and vary with age" (2010, p. 630). These studies and the conflicting views reflect the interest that the criminal career framework has sparked over the years, with relevant contributions also in terms of crime-control policies (DeLisi & Piquero, 2011; Horney, Osgood, & Marshall, 1995; Nagin & Land, 1993).

Over the last two decades, theoretical contributions and empirical studies (Kleemans & de Poot, 2008; Kleemans & Van de Bunt, 1999, 2008; Kleemans & van Koppen, 2014) have underlined the importance of the social environment as a key component for understanding criminal careers of OC offenders and recruitment to OC. Kleemans and Van de Bunt explained the importance of social ties through the concept of "social embeddedness" (1999). In hostile environments, in which offenders carry out their illegal activities, social ties provide new opportunities for offenders and contribute to find suitable solutions for problems of cooperation. The great importance of social relations and trust is one of the main characteristics that distinguishes OC from high-volume crime (Kleemans & de Poot, 2008). Many OC activities are not only logistically more complex, but also require a network of transnational contacts to identify suitable co-offenders or contacts with the legal world.

Drawing from the social embeddedness of organised crime, Kleemans and De Poot explain these processes through Social Opportunity Structure Theory (2008). This merges opportunity theory (Clarke & Felson, 1993), and social

network theory (e.g. Burt, 1992, 2005; Morselli, 2005).[3] Social relations allows to bridge social and criminal networks in various countries. In the social environment, characterised by strong and weak ties, people are drawn more and more into illegal activities. This process is generally labelled "recruitment" (Kleemans & Van de Bunt, 1999, p. 31). Kleemans and Van de Bunt (2008), highlight the importance of work-related contacts to establish criminal co-operation, stressed the embeddedness of certain organised crime activities in work settings. Therefore, the work environment may provide potential OC offenders the opportunity to develop their criminal network through the meeting with co-offenders.

Recent OC research shows that the social environment plays a major role in OC involvement (Kleemans & van Koppen, 2014). The social embeddedness concept is crucial in understanding the complex dynamics underlying individuals' recruitment to OC. The criminal careers framework, instead, represents a meaningful paradigm to interpret the criminal careers of OC offenders through the use of the six parameters as research baseline. The present study seeks to enhance the knowledge on the recruitment and criminal careers of individuals to OC through the criminal careers approach and a focus on the socio-economic factors that contribute to the individuals' involvement in offending.

---

[3] Social opportunity structure, which is unequally distributed across the population and age, explains OC involvement and success giving social ties essential value in providing access to profitable criminal opportunities. In this way, therefore, OC involvement can be understood beyond the individual differences and long-term risk factors (Kleemans & van Koppen, 2014).

2 Data

The PROTON database originates from two datasets provided by the Ministry of Justice. These data represent the entire Italian population of individuals convicted for mafia offenses between 1982 and March 2017 (N=17,391).[4] The Prison Administration Department (DAP) of the Italian Ministry of Justice provided socio-demographic data as well as arrest and imprisonment data of convicted mafia members (henceforth DAP dataset). The Criminal Records Registry (*Casellario giudiziale*) of the Ministry of Justice provided the criminal records (henceforth *Casellario* dataset) for all individuals included in the DAP dataset.[5] Formal agreements with the Ministry of Justice made the data available and guarantee the anonymity of all individuals in compliance with current privacy regulations. Each individual's name was replaced by a unique ID and allows to integrate the DAP and the *Casellario* datasets.

2.1 The DAP dataset

The DAP dataset contains socio-demographic variables, arrest and imprisonment data, information related to the type of affiliation (i.e. mafia association), and the role that mafia members held within their group. Each observation in the dataset represents one entrance in the penitentiary.

The DAP dataset includes 22 variables and represents the entire population of mafia members and bosses that were convicted for mafia offenses between 1982 and March 2017 (Table 1). The dataset also contains records for individuals that were imprisoned multiple times. Of the total number of observations (N=21,373), 17,391 regard unique individuals. Roughly 80% of individuals were imprisoned once (n=14,076), while the remaining 20% were imprisoned multiple times. Each individual has a unique numeric ID. Individuals with multiple imprisonments kept the same ID of their first imprisonment.

---

[4] The Rognoni-La Torre law introduced the crime of mafia association to the Italian Criminal Code (Art. 416-bis) in 1982. A mafia association is defined as a criminal association whose members use intimidation and the power of their associative bond, as well as a code of silence (i.e. *omertà*), to commit serious offenses (see La Spina, 2014, p. 594). The research team considered as mafia offenses the crime of mafia association and other offenses aggravated by the mafia method (i.e.: Articles 416bis, 416ter, 418 of the Italian Criminal Code, and special laws 575/1965, Art. 7 special law 152/1991 and Art. 7 special law 203/1991).

[5] The Criminal Records Registry provided preliminary data on 13,229 IDs out of 17,391 included in the DAP dataset. This is a temporary situation: integration of the data will be provided to the research team in the near future.

Table 1. Description of variables in the DAP dataset

Variable name	Variable explanations
ID	Unique ID to identify an individual without name/surname
REGISTRATION_NUMBER	Unique code to identify each entrance in the penitentiary
YEAR_BIRTH	Year of birth (DD/MM/AA)
SEX	Sex of the individual
COUNTRY_BIRTH	Country of birth
COUNTRY_RESIDENCE	Country of residence
PR_BIRTH	Italian province of birth
PR_RESIDENCE	Italian province of residence
PR_DOMICILE	Italian province of domicile
DATE_ARREST	Date of arrest (DD/MM/AA)
DATE_PROV_RELEASE	Date of provisional release (DD/MM/AA)
DATE_DEF_RELEASE	Date of definitive release (DD/MM/AA)
DATE_RELEASE	Date of release (DD/MM/AA)
DETENTION_STATUS	Individual’s current status (e.g. imprisoned, released, parole)
MARITAL_STATUS	Marital status
RELIGION	Religion
EDUCATION	Educational background
OCCUPATIONAL_STATUS	Occupational status
PROFESSION	Profession
SECTOR_EMPLOYMENT	Economic sector of employment
MAFIA_ASSOCIATION	Type of mafia association (e.g. Cosa Nostra, Camorra)
ROLE	Role within the mafia association

The research team performed data cleaning and data transformation on the entire DAP dataset. This allowed to detect and correct inaccurate records and to prepare and reshape the DAP dataset using every single ID as the unit of observation (see Annex, section 1.1).

2.2 The Casellario dataset

The *Casellario* dataset provides information on all the criminal convictions of each individual in the DAP dataset. Unique numeric IDs (N=13,229) enabled to link the *Casellario* dataset to the DAP dataset.

The *Casellario* dataset consisted of two sub-datasets:

- Offenses (*Reati*)
- Judgements (Provvedimenti)

In the Offenses dataset, each observation referred to a specific offense committed by a single individual, labelled originally with the variable PROG_CRIME (N=206,198). For each observation, the dataset provided eleven variables (Table 2).

Table 2. Description of variables in the Offenses dataset

Original name	Variable Explanation
PROG_PROV (N=102,346)	Code assigned to each criminal judgement
PROVINCE_CRIME	Italian Province where the crime was committed
YEAR_CRIME	Year when the crime was committed
LEG_SOU	Legislative source for the committed crime
YEAR_LAW	Year when the law broken by the committed crime was issued
NUMBER_LAW	Number of the law
ARTICLE	Article of the law/legislative source
ARTI_BTQ	*Bis* / *Ter* / *Quater* version of the article
ARTI_COMMA	Comma of the article
RECID	Specification of the recidivism of the committed crime
TCC	Crime commission typologies

The dataset included thousands of different offenses, as the Italian Criminal Records Registry provides detailed information on the legislative source, article number, and even paragraph of the violated provisions. While most offenses were in the Italian Criminal Code, many others were in special laws. To synthetise the several hundreds of distinct offenses committed by the mafia members, the research team classified them into categories (see Annex, section 1.2.2).

2.3 The Proton Mafia Members dataset

The Proton Mafia Members dataset (henceforth PMM) merged information from the DAP and the *Casellario* datasets, as well as information extracted during the research process. It provides socio-demographic information, information related to the type of affiliation (i.e. mafia association) and the role that mafia members held within their group, as well as information on mafia members' criminal career (descriptive statistics in Table 3).

Table 3. Descriptive statistics for some variables in the PMM dataset

Variable	
Sex	98% are males
Country of birth	97% are born in Italy
Religion	98% are Catholic
Education	82% have between 5 and 8 years of education
Age at first crime	Average: 25 years old
Age at first mafia crime	Average: 34 years old
Age at first arrest	Average: 38 years old
Number of committed crimes	Average: 16
Number of crime categories	Average: 7
Number of violent crimes	Average: 3

Notes: N=13,229.

3 Methodology

3.1 The criminal careers' parameters

The criminal careers framework has never been applied to the analysis of Italian mafia members. This section describes the operationalisation processes for five of the six parameters related to the criminal careers framework. The intermittency parameter has been discarded due to limited data availability.[6]

Participation

In the context of offending, participation refers to "the proportion of a population who are active offenders at any given time" (Blumstein, Cohen, & Farrington, 1988, p. 3). Active offenders include both new offenders and existing offenders, i.e. individuals with previous criminal convictions (see Piquero, Farrington, & Blumstein, 2003). The present study considers and compares two types of participation:

1. "External" participation
2. "Internal" participation

"External" participation considers the suitable reference population, which includes both offenders and non-offenders (Piquero et al., 2003). In this regard, the reference population is the resident population in the areas where mafia members operate. However, the share of residents who are active mafia members is bound to be very small, complicating the interpretation of results. "Internal" participation considers as reference population the number of potentially active mafia members in the dataset who, in any given year, are mafia members who are alive and aged between 14 and 90.[7]

External participation (P_t^E) and internal participation (P_t^I) in year t are calculated as follows:

1. **External participation**:

$$P_t^E = \frac{A_t}{R_t}$$

---

[6] The data at disposal do not allow to identify with certainty when a mafia member is serving prison time. This information is crucial for the computation of the intermittency parameter.

[7] The present study considers as potentially active also offenders who are serving time in prison. This methodological choice is justified by the fact that the DAP dataset does not provide full information on each conviction of mafia members, thus impeding to identify in each year whether a mafia member is in prison or not.

Where:

- A_t is the number of active mafia members in year t, i.e. the number of mafia members committing at least one crime in year t;
- R_t is the resident population aged 14 and over living in the four "mafia regions" (Sicily, Campania, Apulia, Calabria) in year t.[8]

2. Internal participation:

$$P_t^I = \frac{A_t}{M_t}$$

Where:

- A_t is the number of active mafia members in year t;
- M_t is the number of potentially active mafia members in year t, i.e. the number of mafia members in the dataset who are alive[9] and aged between 14 and 90 in year t.

Duration

The duration of a criminal career is the "length of time that an offender continues to commit crimes once beginning an active criminal career" (Rhodes, 1989, p. 3). An approach to measure the duration is to consider the active career length, intended as the number of years in a career less time in custody (see Piquero, Brame, & Lynam, 2004). Nonetheless, the majority of studies measured the duration counting the years between an offender's first and last crime (see, among others, Blumstein, Cohen, & Hsieh, 1982; Farrington, Lambert, & West, 1998; Spelman, 1994). The latter approach represents a straightforward method to calculate the duration and is more appropriate for the data of the present study, as the DAP dataset does not provide full information for all sentences' length. Consequently, the duration of individual *i* (D_i) is calculated as follows:[10]

$$D_i = T_i^{(L)} - T_i^{(F)}$$

---

[8] It must be remarked that not all the convicted mafia members were born and operate in one of the "mafia regions". However, given that the great majority of them (95.3%) was born in one of the four "mafia regions", computing the external participation taking into consideration only the residents of such regions can be considered a pertinent approximation.

[9] The DAP dataset provides only the information as to whether a mafia member died while imprisoned. Hence, mafia members aged between 14 and 90 and who did not die in prison are assumed to be alive and potentially active. This might lead to an overestimation of the number of potentially active mafia members, since some offenders might have died while not serving prison time.

[10] The duration is not computed for mafia members committing only one offense.

Where:

- $T_i^{(L)}$ is the year of the last crime committed by individual *i*;
- $T_i^{(F)}$ is the year of the first crime committed by individual *i*.

Frequency

The individual offending frequency refers to "the average number of crimes committed per year by active offenders" (Farrington, MacLeod, & Piquero, 2016, p. 339). Drawing upon this definition, the offending frequency of individual *i* (F_i) is computed as follows:

$$F_i = \frac{N_i}{D_i}$$

Where:

- N_i is the total number of offenses committed by individual *i*;
- D_i is the career duration of individual *i* (see Section 0).

To explore the evolution of the offending frequency of the entire sample, the average annual frequency in year *t* (F_t) is computed as follows:

$$F_t = \frac{N_t}{A_t}$$

Where:

- N_t is the total number of offenses committed in year *t* by the entire sample;
- A_t is the number of active mafia members in year *t* (i.e. the number of mafia members committing at least one crime in year *t*).

Specialisation

Among criminal offenders, specialisation is "the tendency to commit the same type of crime" (Kyvsgaard, 2002, p. 147).

The present study relies on the diversity index (also referred to as the Simpson's index) to operationalise the specialisation parameter. Whereas it fails to provide information on the kind of specialisation, the diversity index is a relatively straightforward approach yielding a quantitative measure of specialisation at the individual level (see Annex, section 2.1.1).

Following prior studies (McGloin, Sullivan, Piquero, & Bacon, 2008; Piquero, Oster, Mazerolle, Brame, & Dean, 1999; Sullivan, Mcgloin, Pratt, & Piquero, 2006; Wright, Pratt, & DeLisi, 2008), the diversity index for individual *i* (DI_i) is defined as follows:

$$DI_i = 1 - \sum_{m=1}^{31} p_m^{(i)} * p_m^{(i)}$$

Where:

- *m=1, 2…31* are the 31 crime categories identified in the dataset;[11]
- $p_m^{(i)}$ is the proportion of offenses committed by individual *i* in the crime category *m*.

The diversity index can be interpreted as the probability that any two offenses drawn randomly from an individual's set of offenses belong to two different crime categories (Piquero et al., 1999). When DI_i=0, offender *i* is completely specialised on one type of crime. Conversely, a value of the index approaching 1 indicates that the offender engages in a diversity of crime categories.

Escalation

The criminological literature refers to the term "escalation" to define both an increasing offense frequency and an increasing offense seriousness (Liu, Francis, & Soothill, 2011; Sherman et al., 1991). As the interest of the present study is on this latter variable, the operationalization of the escalation parameter will follow Blumstein et al. (1986) who describe the escalation as the tendency to move to more serious offense types.

The present study relies on a regression model to derive an individual-based measure of escalation (for further details, see Annex, section 2.1.2). The regression approach models crime seriousness as a linear function of the order of arrests (Blumstein et al., 1988). This methodology allows to overcome the main flaws of the other approaches estimating escalation (Liu et al., 2011).

By relying on regression models, the operationalization process of the escalation parameter is performed in two steps:

- **Assignment of a seriousness score to each crime:** crime seriousness is estimated as the average punishment calculated employing the average between the maximum and minimum length of the prison sentence[12] for each crime set by the Italian legislation. This methodological choice is supported by the availability of this information.
- **Linear regression** of the seriousness score (as dependent variable) on a temporal measure (independent variable), separately for each individual.

---

[11] See section 2.2 for a description of how the committed offenses have been associated to one of the 31 crime categories.

[12] The average length of the prison sentence is measured in months of detention.

The coefficient of the temporal variable represents the individual-specific measure of escalation. Data availability in the present study allow to employ two different temporal scales:[13]

- The crime number;
- The age at crime commission.

These two scales measure different criminological processes: while change in escalation by crime number reflects the effect of increased experience, change in escalation by age can be thought of as a maturational process (Liu et al., 2011). For this reason, the present study will compare results obtained with both temporal scales.

3.2 Analytical strategy

The analysis of the criminal careers of Italian *Mafiosi* is conducted separately for mafia members (i.e.: the whole mafia population) and mafia leaders. Mafia leaders comprise offenders who have either the role of mafia boss or mafia underboss in the PMM dataset. This choice was made for two main reasons: (i) the analyses conducted separately for bosses (n=390) and underbosses (n=1,480) showed that the two categories have similar characteristics compared to affiliates; (ii) since there are only 390 mafia bosses in the whole sample, combining the two categories allows to obtain more reliable results.

The analysis of the criminal careers of Italian mafia members and mafia leaders relies on a three-level approach that focuses on their macro, meso, and micro dimensions:

- The **macro dimension** analyses mafia members and leaders regardless of the membership to a given organisation. It represents the most aggregated level of analysis and aims at detecting overall patterns and features of these two populations.
- The **meso level** analyses mafia population and mafia leaders by type of mafia (namely, Camorra, Sicilian Mafia, 'Ndrangheta, and Apulian Mafia). It aims at highlighting similarities or differences across four mafia types:

[13] The dataset provides information on the year of commission of each crime. For both temporal scales, when two or more offenses have been committed on the same year, the highest seriousness score was considered. The rationale for this methodological choice lies in viewing an offender as "committing major offenses around their personal mean seriousness level together with other minor offenses, and taking the worst offense provides a better measure of an individual's mean seriousness level" (Liu, Francis, & Soothill, 2011, p. 182). Francis et al. (2013), who focus on escalation among organised crime offenders, also adopt a similar approach.

- Apulian Mafia, i.e. all the mafia organisations established in the Apulian region. The great majority of members of the Apulian Mafia are members of the Sacra Corona Unita;
- Camorra, the mafia organisation established in the Campania region;
- 'Ndrangheta, the mafia organisation established in the Calabria region;
- Sicilian Mafia, i.e. all the mafia organisations established in Sicily. Most of the members of the Sicilian Mafia are part of Cosa Nostra.

- The **micro level** analyses the dynamics and mechanisms of mafia involvement and careers at individual level, providing information on how mafia members act and evolve and eventually become leaders.

The analyses of each dimension employed different quantitative techniques, namely group based trajectory modelling, regression models, and statistical correlation, using the criminal careers parameters as fundamental variables in all the analyses. The analysis exploited available social and demographic variables, in addition to the criminal careers parameters calculated for each individual in the whole population.

Group Based Trajectory Modelling

Group Based Trajectory Modelling (henceforth GBTM) is a statistical technique extensively applied in criminology (see Nagin, 2016) that aims to detect different strata within a given population (Jones & Nagin, 2013). Technically, GBTM is a specialised form of finite mixture modelling and allows to analyse developmental trajectories measuring a certain outcome over certain time periods. For the analysis of criminal careers of Italian mafia members, GBTM has been implemented to find different career trajectories both at macro and meso levels. In this work, the outcome (also dependent variable) is represented by the number of offenses committed by an individual at a given age. The model allows to identify distinct subpopulations that share common offending trajectories over their careers. Its use permits to detect significant patterns and to test the outcomes of the clustering through further statistical techniques, as regression models.

Regression Models

The analysis employs three typologies of regression: (i) Ordinary Least Squares (OLS) regressions, (ii) Logistic regressions, and (iii) Multinomial logistic regression. OLS allow to analyse the factors determining the age of recruitment,[14] jointly testing what are the social and criminal variables that

[14] To compare mafia members and bosses' behaviour before and after their involvement into the mafias, the age of recruitment of each individual was set at his/her first mafia

significantly impact the offender's age at first mafia association offense. Logistic regression models are employed whenever the dependent variable is a dichotomous outcome. This is the case of the analysis of the factors determining being a boss. Moreover, the analysis relies on logistic regression models to explore the differences in the criminal career of mafia members or of mafia bosses across different mafia organisations. Multinomial logistic regressions investigate the differences in the criminal careers of bosses and underbosses. Multinomial regressions are also used in the analysis of the factors determining an early or a late recruitment into the mafia. These latter models have as dependent variable a categorical variable that divides the mafia members' population into "early recruits", "late recruits" and individuals recruited at an average age.

---

association offense. This choice required a robustness check to ensure the reliability of the results. To do so, three time-buffers (of 1, 3, and 5 years prior to the first mafia association offense) were introduced, moving backwards in time the year of recruitment. This type of recruitment was assessed through statistical correlation. Pearson's r was calculated across the criminal career parameters measured for each individual in four different time spans, namely entire career pre-mafia, (career pre-mafia)-1 year, (career pre-mafia)-3, and (career pre-mafia)-5. The analysis of the correlation allowed to understand whether recruitment could be more correctly modelled by setting the age of recruitment prior to the first mafia sentence. Correlation values are shown in Annex (section 3.1.2), proving the robustness of the choice of the recruitment moment.

4 Results: the criminal careers of mafia members

This section presents the results for the whole population of mafia members. The macro level focuses on the characteristics of the members of Italian mafias. The meso level explores similarities and differences of mafia members across mafia types. The micro level investigates personal and criminal characteristics leading to the recruitment into mafias at individual level.

4.1 Macro level: the parameters of the criminal careers of mafia members

This subsection presents the outcome of the patterns and trends in the five parameters of the criminal careers of Italian mafia members. Furthermore, it analyses the criminal trajectories and compare careers before and after the recruitment into mafias. Overall, this subsection addresses the following questions:

- *What are the main characteristics of the members of Italian mafias?*
- *How do mafia careers evolve over time?*
- *What are the patterns in mafia members' career before and after the recruitment into mafias?*

4.1.1 Descriptive statistics of the criminal careers of mafia members

Participation

The internal and the external participation show a very similar trend, with a peak in 1991 and a sharp decrease starting from 2007 (Figure 1). External participation is computed employing a more robust denominator than the internal participation. The latter relied on the identification of "potentially active mafia members", which requires some assumptions on the life span of mafia members, a variable for which exact information is unavailable (see Section 0). Nonetheless, the similar participation curve in both measurements suggests that lack of robustness is not a major issue.

Figure 1. Participation in crime

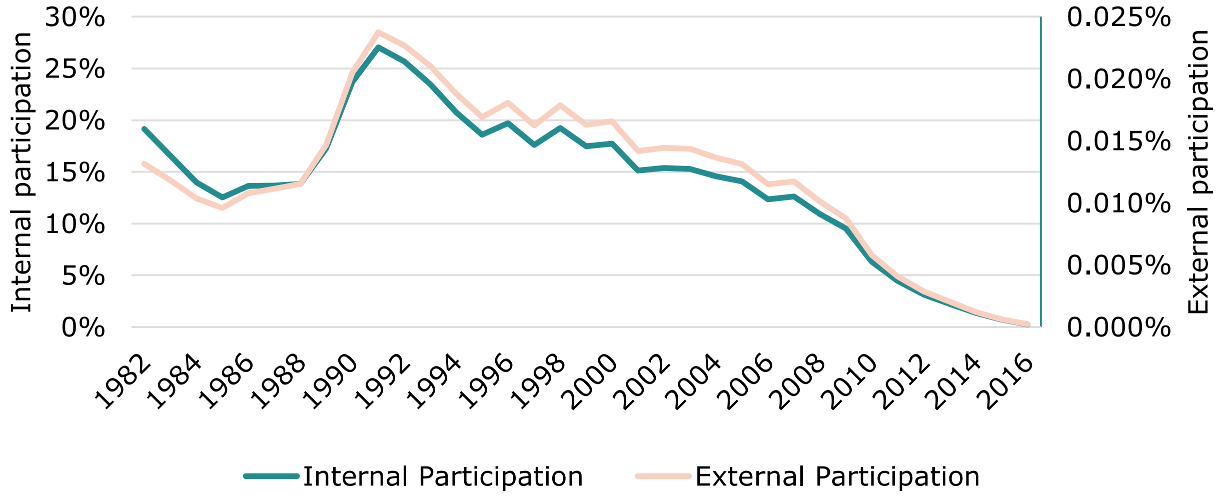

Notes: values for external participation reported on the right axis, while values for internal participation reported on the left axis.

The drop of participation starting from 2007 is influenced by the delay in recording more recent offenses in the *Casellario* dataset. As the sample only includes criminals with a final conviction for mafia offenses, in the last years only a minor share of suspects charged with a mafia offense are likely to have already received a final conviction. In Italy, proceedings for mafia offenses often require long time before the final judgement. Based on the analysis of the data from the *Casellario*, on average Italian courts issue definitive judgements (i.e. irrevocable sentences) 6 years after the commission of the mafia association crime. This estimate is in line with data disseminated by the Ministry of Justice (Ministry of Justice - Department of Judicial Organisation, 2016).[15] These data indicate that the first instance of a criminal proceeding lasts on average 1.7 years, while second instance lasts 2.5 years. Considering that the Italian judicial system has also a third judicial instance, overall duration is in line with our estimation (6 years).

The duration of criminal proceedings might not be the unique explanation for the decreasing trend in participation. The drop might also depend on an actual decrease in mafia members' participation in crime. This finding could suggest that the Italian mafias are becoming weaker and less active, e.g. due to an incapacitation effect. The increasing number of prisoners incarcerated for the

[15] These data indicate that the first instance of a criminal proceeding lasts on average 1.7 years, while second instance lasts 2.5 years. Considering that the Italian judicial system has also a third judicial instance, overall duration is in line with our estimation (6 years).

mafia association offense corroborates the hypothesis of an incapacitation effect (Figure 2).

Figure 2. Number of prisoners for the mafia association offense

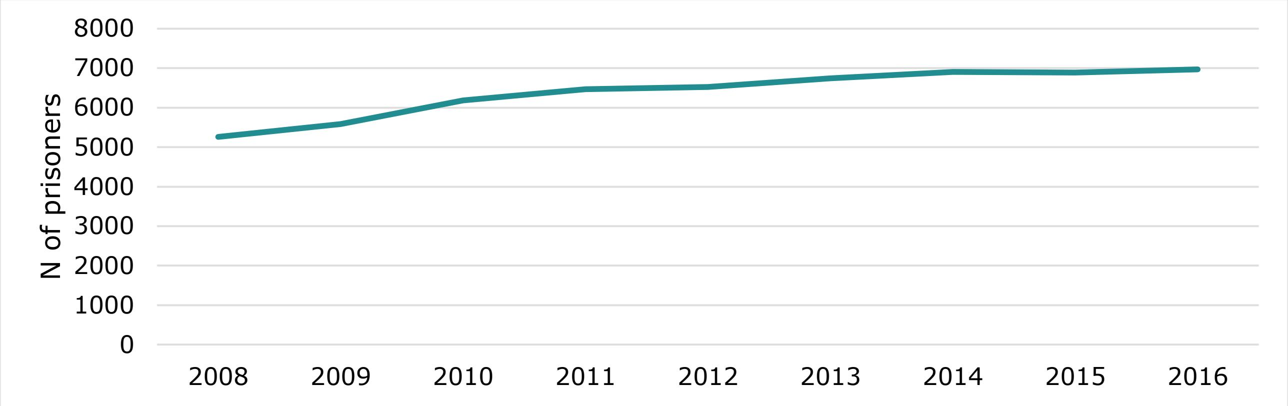

Source: ISTAT.

Further analyses are conducted to check whether the Italian mafias are experiencing an actual decrease in participation. Mafia members' participation in crime is compared to the trend in the number of mafia associations and the number of mafia suspects reported by Italian law enforcement agencies in each year (Figure 3). Reported mafia associations show a decreasing trend between 1983 and 2016, with a strong decrease from 2003 onwards. Conversely, data on reported mafia suspects, available only for 2004-2015, show a stable trend.

The collected evidence suggests that an actual decrease in mafia members' participation in crime cannot be ruled out.

Figure 3. Number of reported mafia associations and reported suspects for mafia association

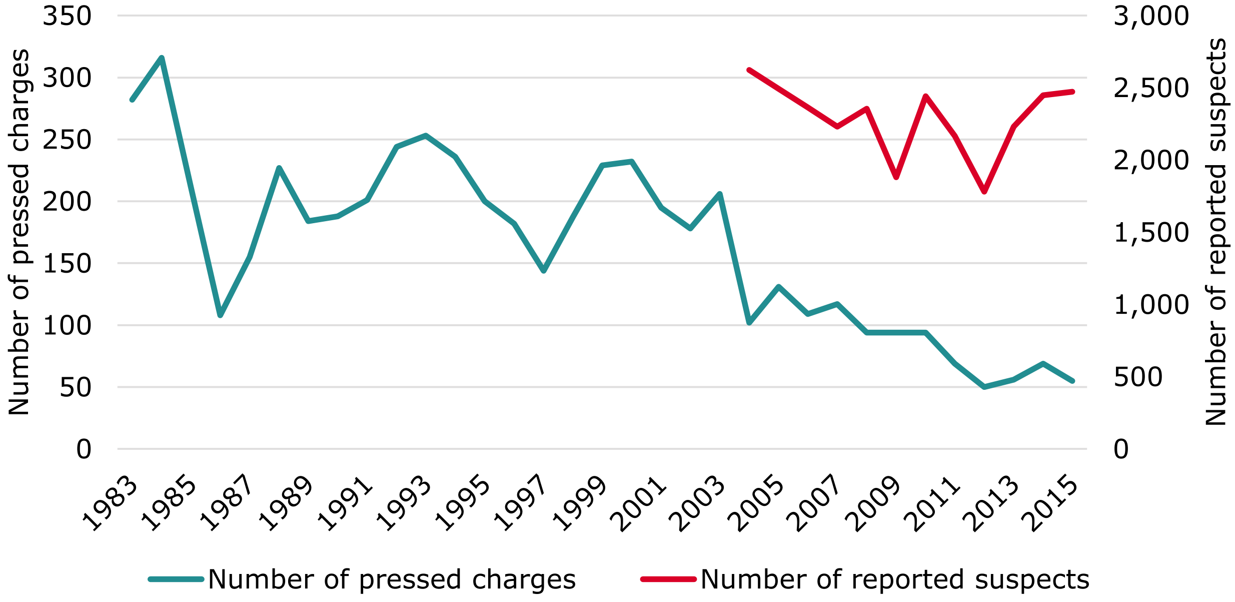

Notes: values for reported mafia associations are on the left axis, while values for the number of reported suspects are on the right axis. The number of reported suspects include alleged offenders (reported or arrested); each offender is counted only once. Source: SDI data (Police Inquiry System), mod. 165 data, ISTAT.

DURATION

The mean of the criminal career duration for the entire sample is approximately 14 years, although there is substantial variability across the sample (Figure 4) The duration is an increasing function of the number of committed crimes if this last variable is between 1 and about 50 committed crimes, while the relationship between these two variables appear less clear for a higher number of committed offenses (Figure 4).

Figure 4. Duration of the criminal careers of mafia members and scatter plot of individual duration by number of committed crimes

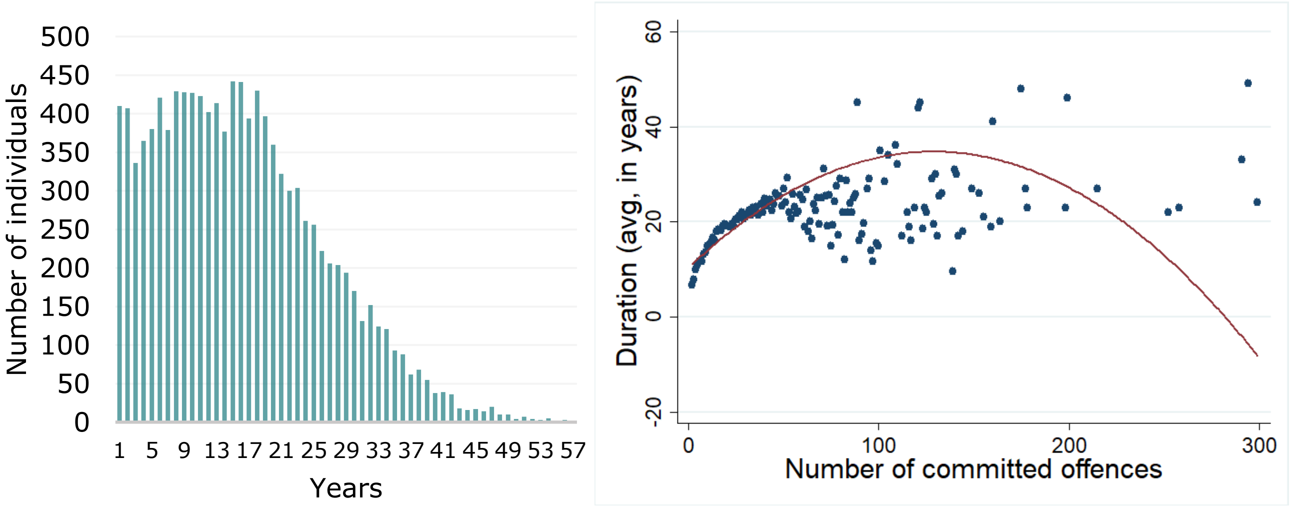

Notes: n=11,641. Mafia members with D=0 are excluded from this graph (n=1,586). The scatter plot has been estimated by computing the average duration within bins of individuals with the same number of committed offenses. A quadratic fit line is also plotted.

This average duration of mafia members is generally higher than the one found by other studies. For example, Blumstein et al. (1982) estimated an average duration of criminal careers of 5 years, with the longest careers (which averaged about 10 years among 18-year-old-starters) associated with individuals responsible for committing murder and aggravated assault offenses. Other studies obtain estimates of the mean length of a criminal career that ranges between 5 and 10 years (Farrington et al., 1998; Spelman, 1994; Tarling, Research, & Unit, 1993). However, these works examined the career length in samples of common criminals.

Although fewer in number, studies on the criminal career of serious offenders found career durations more in line with the career of Italian mafiosi. Weisburd and Waring (2001) analyse the career length among a sample of white-collar criminals, finding a mean duration among repeat offenders of about 14 years. Piquero et al. (2004) examine the career duration in a sample of serious offenders and find an average career duration of 17.3 years. In their sample, the majority of criminal careers is centred in the middle of the duration distribution (between 15 and 20 years), with few parolees exhibiting extremely short and extremely long careers. This is quite different from the distribution of the duration in the present sample, where the number of mafia members with a short criminal career is considerable.

Frequency

The offending frequency of mafia members is 1.52 offenses per year on average. The majority of mafia members commit less than 3 offenses per year (Figure 5). Data are likely to underestimate the actual frequency. Individual frequencies divide the number of committed offenses by the career length or duration. However, the duration likely includes periods when individuals are unable to offend, e.g. due to being in detention, out of the country, or in hospitals (Farrington, 1987). As a result, the estimated frequency of mafia members is generally lower than the frequency reported by other studies (see, for example, Greenwood & Abrahamse, 1982; Peterson, Braiker, & Polich, 1980). More detailed analysis should eliminate years in which the individuals were unable to offend (Farrington, 1987). However, currently available data lack sufficient information.

The yearly average frequency enables assessment of the evolution of the individual frequency across time for active mafia members, i.e. mafia members committing at least one crime in the specific year (Figure 5). The yearly average frequency ranges between 3.68 offenses in 1991 and 1.50 offenses in 2015. Similar to the participation parameter, the trend peaks in 1991 and slowly decreases afterwards. The more evident drop since 2011 may be due to the low number of final convictions in the most recent years.

Figure 5. Distribution of individual offending frequency and trend in the yearly average frequency of active mafia members

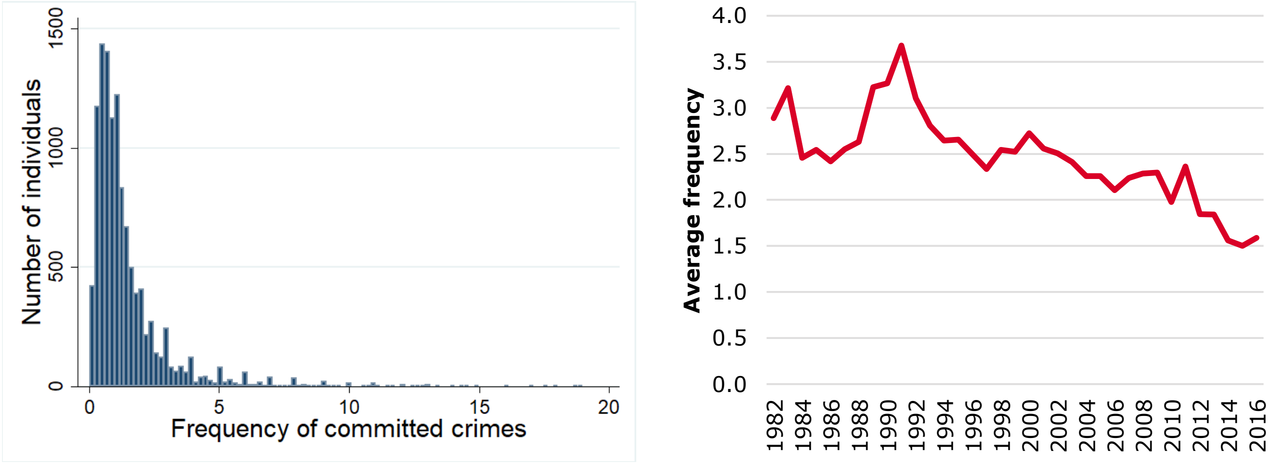

Note: n=11,641 for the distribution of individual offending frequency.

Specialisation

The distribution of the diversity index in the sample is left-skewed with a mean of 0.75 (Figure 6). The peak of the diversity index for the value *DI*=0.5 is influenced by the presence of a large set of mafia members committing only two offenses and in two different crime categories (about 92% of the mafia

members with diversity index equal to 0.5 committed only 2 offenses during their criminal career). The diversity index increases with the number of committed offenses up until the value of 30 committed crimes (Figure 6).

Figure 6. Distribution of diversity index in the sample and scatter plot of diversity index by number of committed crimes

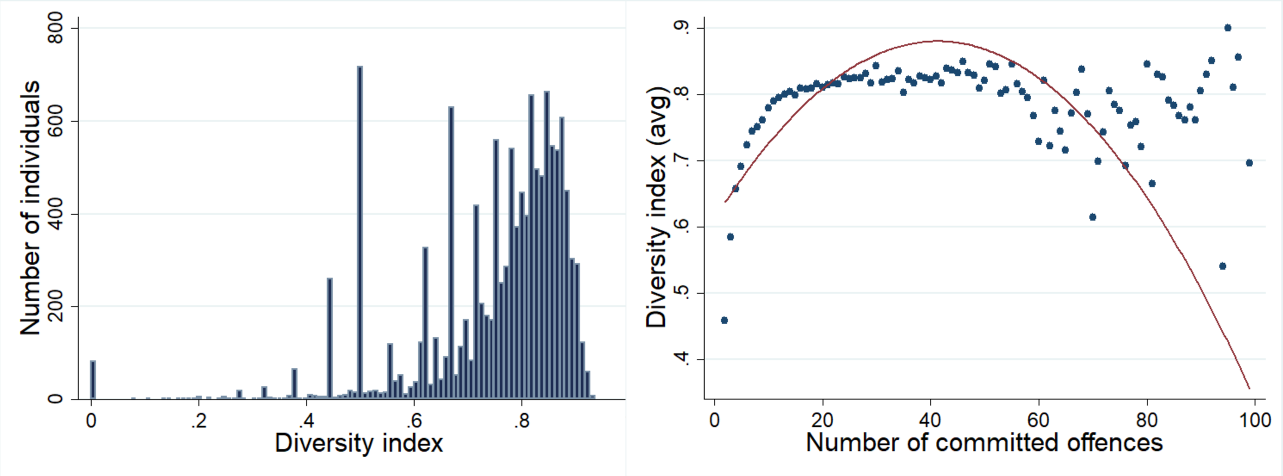

Notes: n=12,543. Mafia members who committed only one crime are excluded from the calculations (their diversity index would trivially be 0). The scatter plot has been estimated by computing the average diversity index within bins of individuals with the same number of committed offenses. A quadratic fit line is also plotted.

Average specialisation is particularly low in the sample of mafia members under investigation, suggesting that mafia members seem to be versatile offenders. Previous studies focusing on samples of general offenders have also found that, in general, most offenders are not specialised on one type of crime (Soothill, Francis, Ackerley, & Humphreys, 2007; Sullivan, McGloin, Ray, & Caudy, 2009). Francis et al. (2013) examined the specialisation of offenders involved in organised crime. They also found a mean diversity index pointing to the low specialisation of organised crime offenders. However, direct comparison warrants caution, since the maximum value that the diversity index can take depends on the number of crime categories (Sullivan et al., 2009).

Escalation

The assessment of the escalation requires the calculation of the seriousness of the different offenses. The average seriousness for mafia members is around 104 months of detention per individual (Figure 7). All offenses committed by member of the mafias were grouped into 31 crime categories. While some categories included very specific offenses with similar seriousness (e.g. robbery, murder), other categories comprise different offenses of similar types.

The categorization is overall consistent. Only less frequent and residual categories exhibit high heterogeneity in the seriousness (Figure 8).[16] The most serious categories are murder, drug trafficking criminal association, and mafia association.

Figure 7. Distribution of individual mean seriousness of committed crimes and trend of the average seriousness of committed crimes by age of the offender

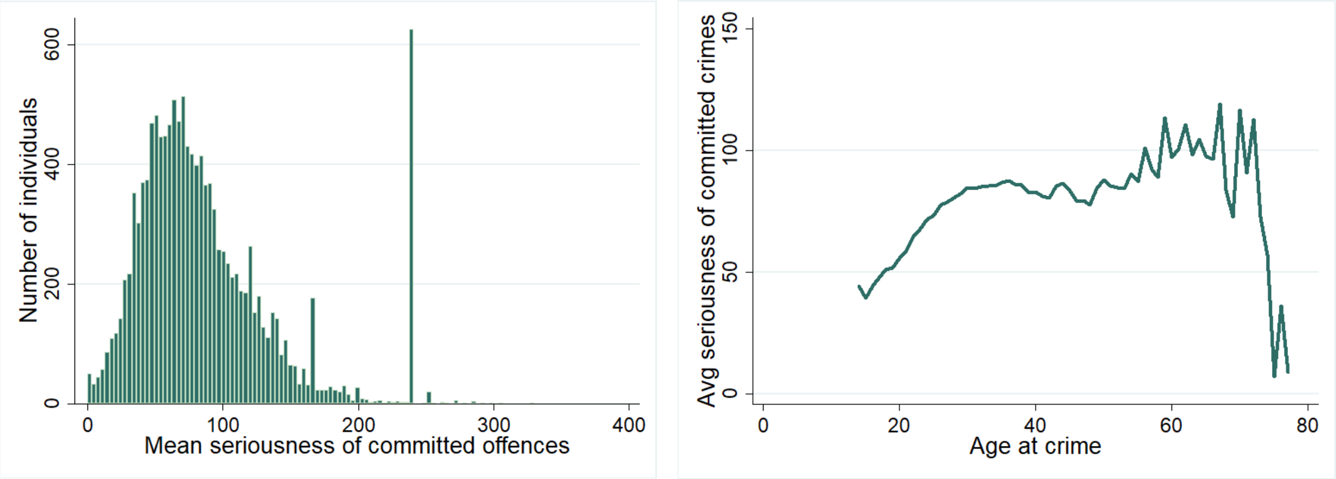

Note: N=13,229 for the distribution of the individual mean seriousness.

The assigned seriousness score is the dependent variable in the individual-level linear regression on the temporal scale using either age or crime number as independent variable. The coefficient on the temporal variable is the estimated escalation for each individual.

[16] The crime categories exhibiting high variance are the residual ones (e.g. "Other – Felonies", "Other special laws"), which indeed group different types of offenses with very different statutory penalties.

Figure 8. Box plots of seriousness score per crime category

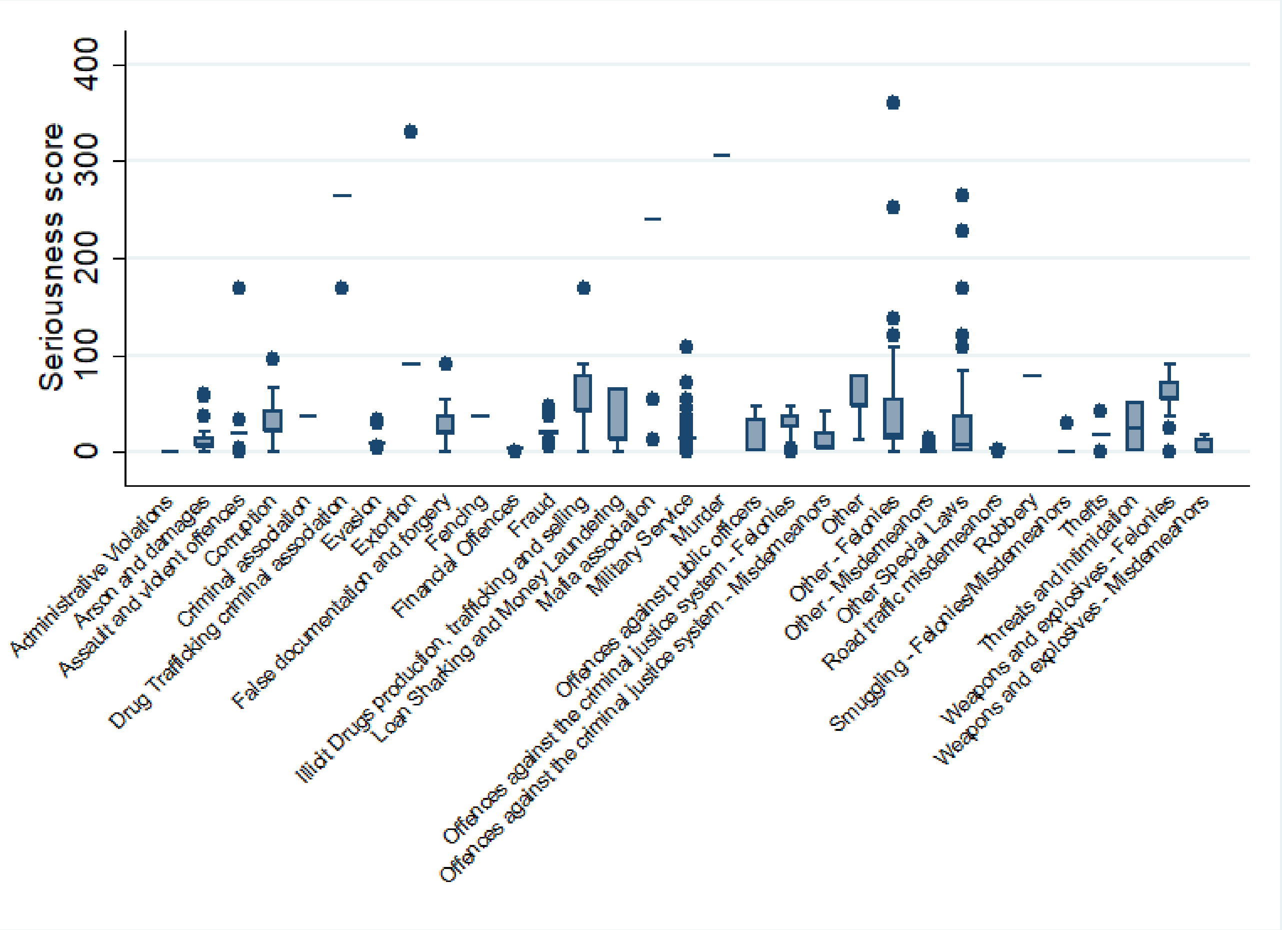

The escalation distribution for both temporal scales visually resembles to a t-student distribution with positive mean (Figure 9). On average, mafia members tend to move from less serious offenses to more serious offenses during their criminal career. This is also supported by the right pane in Figure 7, which plots the average seriousness of the committed offenses against the age of the offender. The graph shows a rapid escalation in crime seriousness between age 15 and the late 30s, followed by a gradual increase in later years, and a drop after age 70.

The distribution of escalation by age is more concentrated around its mean (4.49) compared to the distribution of escalation by crime number (whose mean is 14.18). The higher mean of escalation by crime number suggests that mafia members tend to spread the offenses they commit across many years (and with a low offending frequency), thus making escalation by crime number more apparent compared to escalation by age.

Figure 9. Distribution of the escalation by age and by crime number

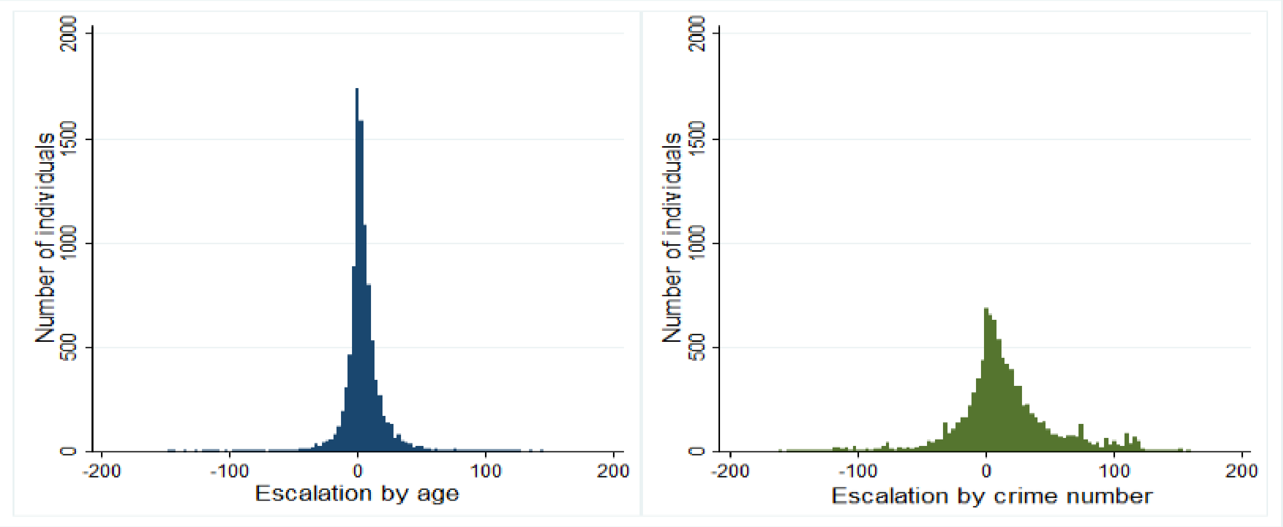

Note: n=9,760.

These results provide a first preliminary evidence on the presence of escalation in mafia members' criminal careers. Previous works on escalation found mixed evidence on the escalation in crime seriousness during an offender's criminal career. The results of the present study are in line with studies by Rojek and Erikson (1982) and Wolfgang et al. (1972), who find evidence of escalation in juvenile offenders. Francis et al. (2013) also find that the average offense seriousness of organised crime offenders increases with offenders' age, although this general trend masks more varied patterns of escalation. On the other hand, other studies suggested the presence of de-escalation in samples of adult offenders (Blumstein, Farrington, & Moitra, 1985) or found no evidence for escalation (Shelden, Horvath, & Tracy, 1989).

4.1.2 THE TRAJECTORIES OF MAFIA MEMBERS

GBTM identified five distinct groups with specific characteristics (Figure 10). For technical details, see Annex (section 3.1.1).

Figure 10. Group Based Trajectories for mafia members

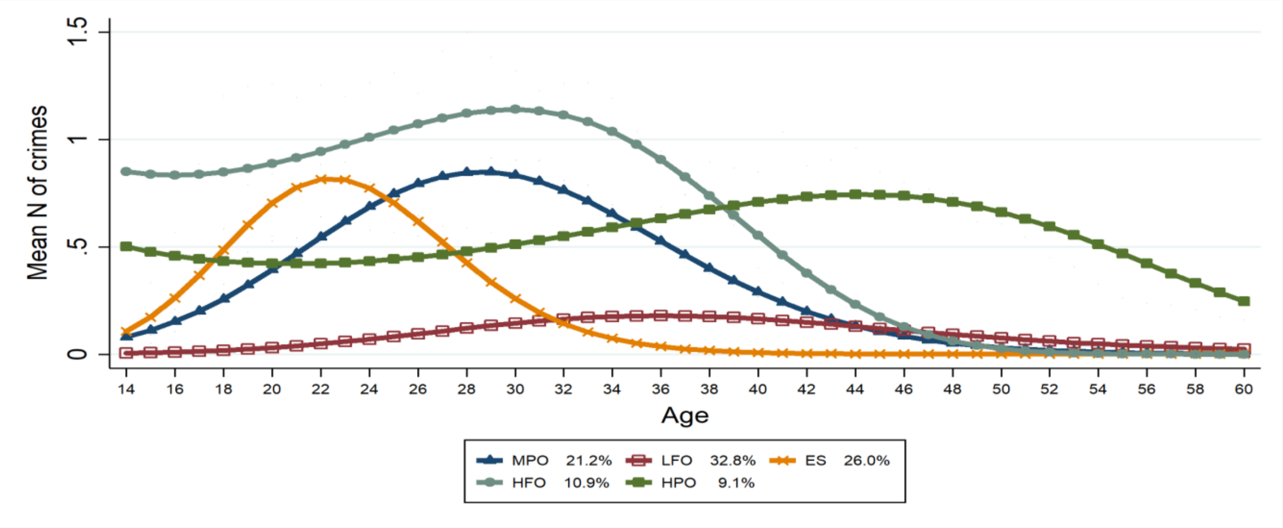

Note: N=13,229.

The GBTM detected five different groups holding specific features (Table 4):

- Group 1 (*Moderate Persistence Offenders*, MPO): MPO commit on average 19.52 offenses in their career, with frequency equal to 1.11 offenses per year.
- Group 2 (*Low Frequency Offenders*, LFO): LFO commit the lowest number of offenses on average (5.57), with frequency equal to 0.94.
- Group 3 (*Early Starters*, ES): ES engage in crime early (on average, when aged 19.80).
- Group 4 (*High Frequency Offenders*, HFO): HFO commit the highest number of offenses (36.21) with the highest frequency (3.21 offenses per year).
- Group 5 (*High Persistence Offenders*, HPO): PO commit a high number of offenses (32.97) with moderate frequency (1.33). Moreover, they tend to commit offenses also in the late adulthood.

Table 4. Criminal features of mafia members' trajectory groups

Group		Moderate Persistence Offenders (1)	Low Frequency Offenders (2)	Early Starters (3)	High Frequency Offenders (4)	High Persistence Offenders (5)	Pop Average
**Career**	Avg age at 1st crime	20.82	32.40	19.80	20.81	22.83	*24.63*
	Avg age 1st arrest	36.44	44.53	29.37	33.28	45.40	*37.87*
	Avg age 1st Mafia ass.	33.35	40.71	25.81	30.91	41.62	*34.37*
	Avg N of crimes	19.52	5.57	11.85	36.21	32.97	*15.59*
	Avg N of crime cat.	9.37	3.85	6.64	10.71	11.41	*7.06*
	Average Seriousness	68.48	111.37	81.35	76.98	62.75	*87.15*
	Avg N of violent crimes	3.47	0.87	2.35	9.41	6.10	*3.12*
**Pre-Mafia**	Avg N of crimes pre-mafia	10.06	2.10	6.26	19.26	15.77	*8.16*
	Avg Seriousness pre-Mafia	52.17	48.92	56.78	64.85	49.54	*53.79*
	Avg N of violent crimes pre-mafia	2.29	0.70	1.77	5.66	3.13	*2.25*
**Criminal Career Parameters**	Avg Duration	19.93	10.63	9.26	16.78	28.84	*14.44*
	Avg Frequency	1.11	0.94	1.80	3.21	1.33	*1.52*
	Avg Diversity Index	0.82	0.66	0.76	0.78	0.81	*0.75*
	Avg Escalation Age	3.62	4.93	5.79	4.36	2.80	*4.49*
	Avg Escalation Order	12.50	20.77	13.58	11.31	10.83	*14.18*

Notes: each distribution is divided into three colour classes. For Avg age at 1st crime, Ave age 1st arrest, and Avg age 1st mafia association, values below the average-½ st.dev. are in red; values above average+½ st.dev. are in green; values between average ± ½ st.dev. are in yellow. For all the other variables, green and red are inverted.
ANOVA tests revealed statistical significant differences across groups for all variables (Prob>F=.000)

The analysis of career features for each group allowed to gain more insights in groups' characteristics.

MPO exhibit values in line with the average of the population for the majority of the features. Only seriousness and escalation age are below the average. Conversely, average age at first crime, average number of crime categories, duration and diversity index yield values above the mean. LFO report values below the average for all the variables, except for seriousness (which is the highest across groups), frequency, escalation age and escalation order. ES show values above the mean for the variables related to criminal onset. Moreover, higher than the average. All the other values are around the mean. HFO have very high values for most variables. Indeed, except for seriousness, duration and both escalations, all the other variables are above the population mean.

4.1.3 THE CRIMINAL CAREERS BEFORE AND AFTER RECRUITMENT

To explore the differences in the criminal career of mafia members before and after the recruitment into mafias, the analysis focused on the subset of individuals (N=5,717) with information on the year of the mafia association

offense (see Annex, section 3.1.2). For this subset, a new variable (year_recruitment) reported the values of year_crime for the first mafia association. For each mafia member, the six dimensions of duration, frequency, specialization, escalation, number of crimes, and average seriousness were computed for both periods before and after recruitment (Figure 11) (see Annex, section 3.1.2).[17]

Figure 11. Distributions of the criminal career's parameters of mafia members, before and after recruitment

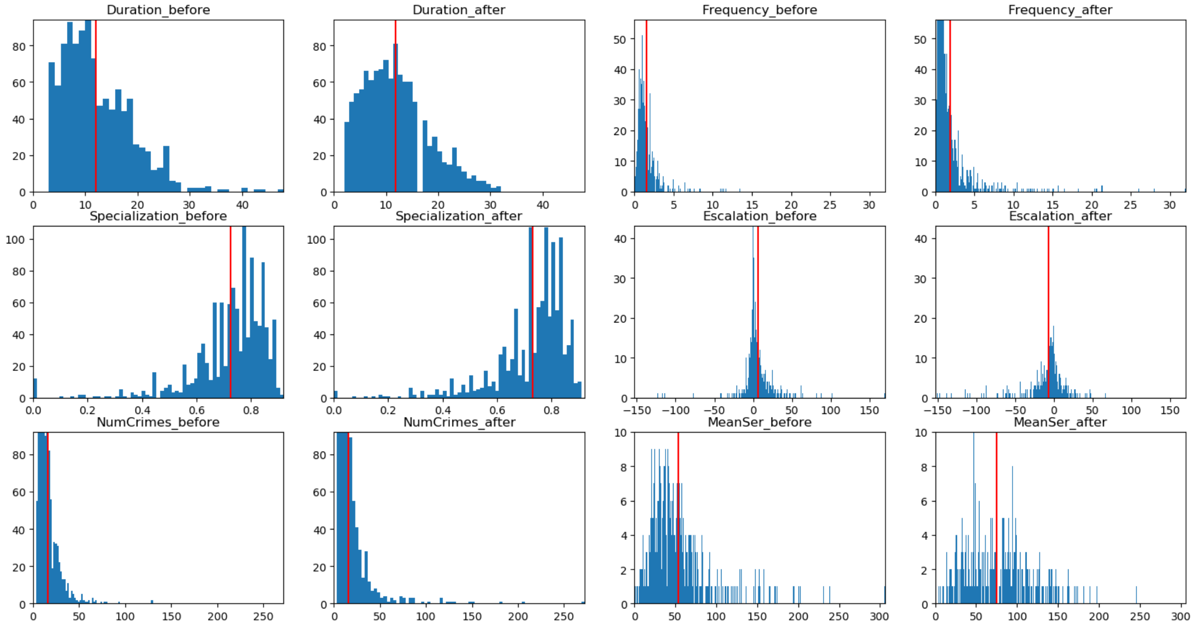

Note: the mean values are plotted as vertical red lines.

The statistical significance of the differences before and after recruitment was assessed with T-tests. Results show a statistical difference in the mean values of the parameters escalation and average seriousness (Table 5). Escalation is significantly lower after recruitment; whist the average seriousness is significantly higher. This suggests an escalation in the criminal pattern of mafia members before recruitment, followed by more serious offenses after the recruitment moment.

---

[17] To test the robustness of the choice of the variable year_recruitment, identified as the year of the first mafia association crime committed by a given member, we repeated the calculation of the parameter distributions moving backwards in time the year_recruitment variable of 1, 3 and 5 years. The analyses focused on those members with information about all the six different dimensions, and on the four time-buffers defined for 0, 1, 3 and 5 years. The correlation was computed, for each dimension, before and after recruitment, and across the time-buffers. Results show strong correlations on average, pointing out the robustness of the choice of year_recruitment (see Annex, section 3.1.2).

Table 5. Mean values of the criminal career's parameters of mafia members before and after recruitment

Parameter	before/after	Mean value	T-test score	p-value	H_0
Duration	Before	12.09	1.179	0.238	not rejected
	After	11.77			
Frequency	Before	1.58	0.001	0.900	not rejected
	After	1.92			
Specialization	Before	0.73	0.000	1.000	not rejected
	After	0.73			
Escalation	Before	6.37	12.831	0.000	rejected
	After	-6.43			
Number of crimes	Before	15.70	0.000	1.000	not rejected
	After	16.05			
Average seriousness	Before	53.55	-13.191	0.000	rejected
	After	75.10			

The average seriousness shows an almost constant escalation in the years before recruitment, followed by an almost symmetric de-escalation (Figure 12, upper pane). A more specific analysis focuses only on the "active years". Starting from the year of recruitment t0, each step backwards/ forwards is the previous/following year in which the mafia member was "active", i.e. committed at least one crime (Figure 12, lower pane). Contrary to the analysis across time, which comprises periods of both criminal activity and inactivity, the analysis across "active years" only accounts for periods of criminal activity.

Analysis of the evolution of the seriousness suggests that mafia members follow a specific escalation pattern before recruitment. Average seriousness peaks at the year of recruitment, followed by a de-escalation pattern. Prison sentences for mafia association and other serious crime likely account for an important part of the de-escalation after recruitment. Prison time reduces the offending capacity of the mafia members, bringing their annuals seriousness scores to 0, which in turn depresses the average values. Indeed, the after-recruitment behaviour clearly differs when analysing the seriousness over "active years". After increasing before recruitment and peaking in the year of recruitment, the average crime seriousness stabilises after recruitment. Removing periods of inactivity (likely to include prison time) uncovers that the seriousness of crimes after recruitment maintains higher levels than before recruitment.

Figure 12. Average seriousness before and after recruitment, all members. Actual years (upper pane) and "active years" (lower pane)

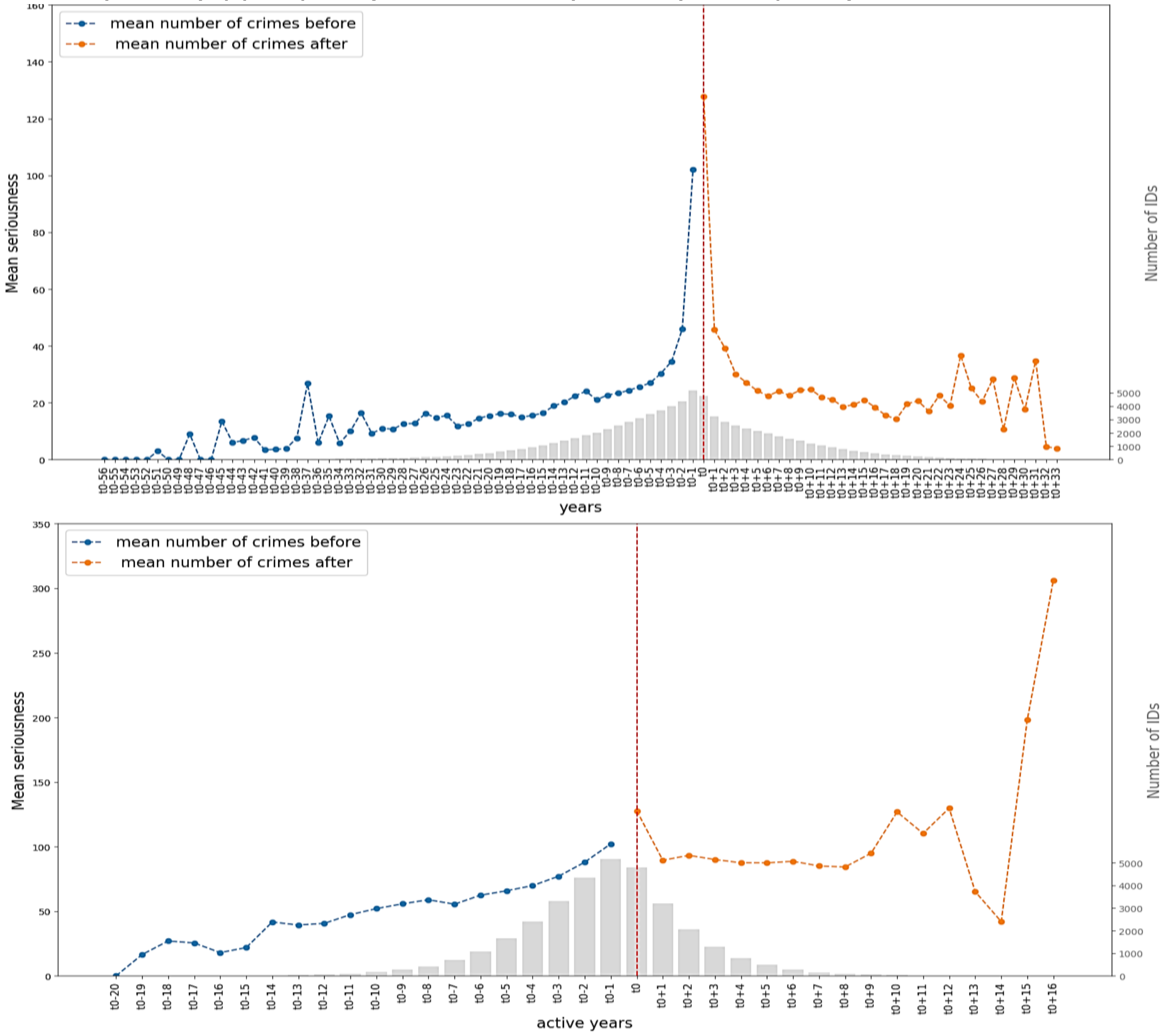

Notes: t0 is the recruitment moment (year of the first mafia offense).

A similar approach considered the number of crimes before and after recruitment (Figure 13). Across years, the average number of crimes shows, as for the escalation, an almost constant escalation before recruitment, followed by an almost symmetric de-escalation after recruitment (Figure 13, upper pane). The analysis of the average number of crimes across "active years" may remove the bias in the data due to prison sentences. It confirms the escalation before recruitment, but points up an increasing number of crimes after recruitment (Figure 13, lower pane).

Overall, analysis of the seriousness and number of crimes before and after recruitment shows a remarkable escalation. Mafia members increase their offending frequency and particularly the seriousness of the offenses. The path to mafia recruitment suggests that, on average, candidates need to show their criminal skills to be admitted in the organization. Recruitment impacts

significantly on mafia members' offending patterns. The number of crimes, when considering only "active years" continues to escalate, while the seriousness stabilises at high levels. Recruitment into the mafias performs a catalyst function in the criminal career of the members, expanding offending opportunities both in number and seriousness.

Figure 13. Average number of crimes before and after recruitment, all members. Actual years (upper pane) and "active years" (lower pane)

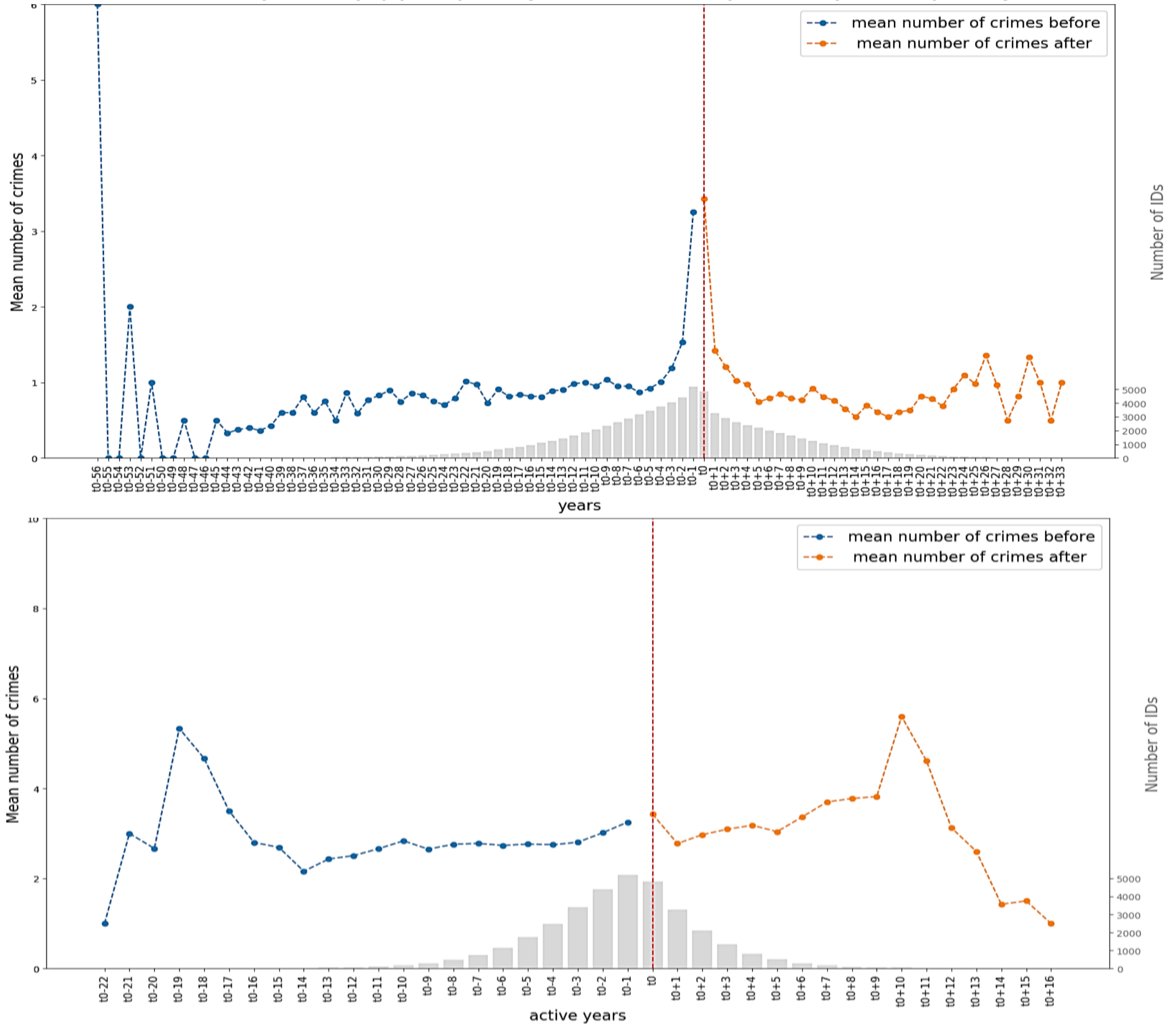

Notes: *t0 is the recruitment moment (year of the first mafia offense).*

4.2 Meso level: mafia members across the types of mafias

This subsection presents the outcomes of the five parameters related to the criminal careers of Italian mafia members divided by mafia type. It also detect developmental trajectories and analyse the existing differences and similarities

in the criminal careers across the four types of mafias. The subsection addresses the following questions:

- *Do mafia members parameters differ across different mafias?*
- *What are the developmental trajectories of mafia members across different mafias?*
- *What are the significant differences of members belonging to different mafias?*

4.2.1 DESCRIPTIVE STATISTICS OF THE CRIMINAL CAREERS OF MAFIA MEMBERS BY TYPE OF MAFIA

The parameters describing mafia members' criminal careers differ significantly across types of mafia (Table 6).

Even though both internal and external participation follow very similar trends in the four mafia organisations (Figure 14), a lower share of Apulian residents is part of the Apulian mafia compared to residents of other regions with widespread mafia presence, but on average a higher share of Apulian mafia members are active offenders in any given year. On the other hand, a lower share of 'Ndrangheta members are active offenders in the period 1990-2006 compared to members of all the other mafia organisations.

On average, Apulian mafia members seem to be the most serious offenders: they have the longest criminal career, the highest offending frequency (Figure 15) and they are the most versatile offenders (Figure 16). They also exhibit the highest escalation by age, while Camorra and Sicilian mafia members appear to have on average the highest escalation by crime number (Figure 16). The Apulian mafia experienced the greatest expansion between the 1980s and 1990s, after which it faced many arrests, trials and defections, which caused it to implement a strategy of violent conflict against the State and other criminal organisations (Massari, 2014). Moreover, contrary to other mafia organisations, recruitment procedures in the Apulian mafia are not based on biological-parental principles, causing offenders who seek to gain a leading role in the organisation to have extremely intense criminal careers (Massari, 2014).

Table 6. Descriptive statistics of the criminal career's parameters in the four Italian mafia organisations

	Type of mafia association			
**Parameter**	**Apulian Mafia**	**'Ndrangheta**	**Camorra**	**Sicilian Mafia**
Participation (internal)†	18.35%	10.98%	14.86%	12.88%
Participation (external)†	0.0093%	0.0120%	0.0115%	0.0132%
Duration***	15.14 (9.89)	13.14 (11.45)	14.56 (10.64)	14.67 (11.09)
Frequency***	1.70 (1.60)	1.46 (1.84)	1.50 (2.03)	1.43 (1.94)
Specialisation***	0.80 (0.12)	0.71 (0.15)	0.75 (0.14)	0.74 (0.15)
Escalation (by crime number)***	13.20 (35.96)	9.90 (47.92)	15.52 (38.59)	15.67 (43.59)
Escalation (by age) ***	5.35 (18.32)	3.20 (21.18)	5.07 (16.68)	4.07 (17.06)

*Notes: standard deviations reported in parenthesis. † for internal and external participation, the average participation over time is shown. For all the other parameters, the average across individual level values is presented. ANOVA tests have been run for all the parameters included in this table except for the participation: ***, ** and * indicate statistically significant differences among mafia associations at the 1, 5 and 10 per cent level.*

Figure 14. Trend of the internal and external participation in the four Italian mafia organisations

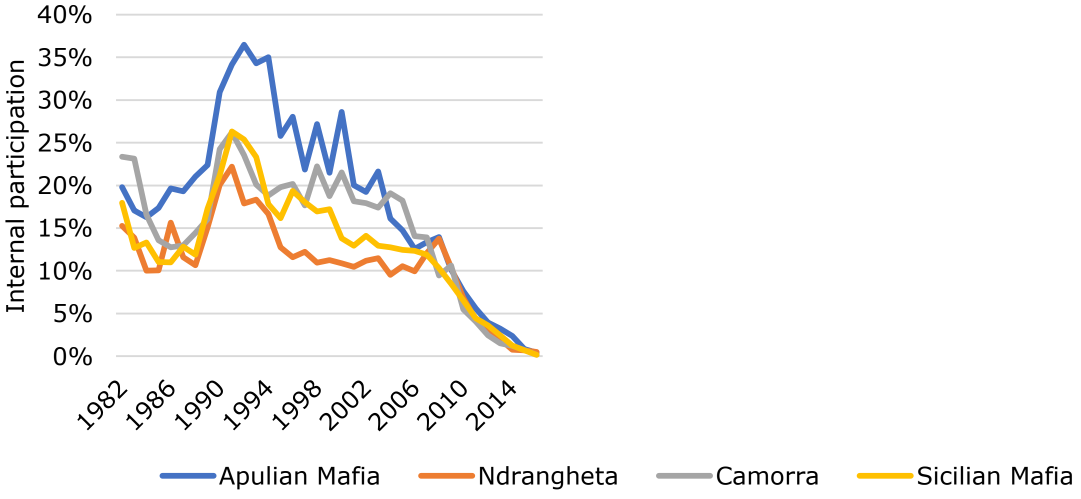

Figure 15. Distribution of the duration and of the offending frequency in the four Italian mafia organisations

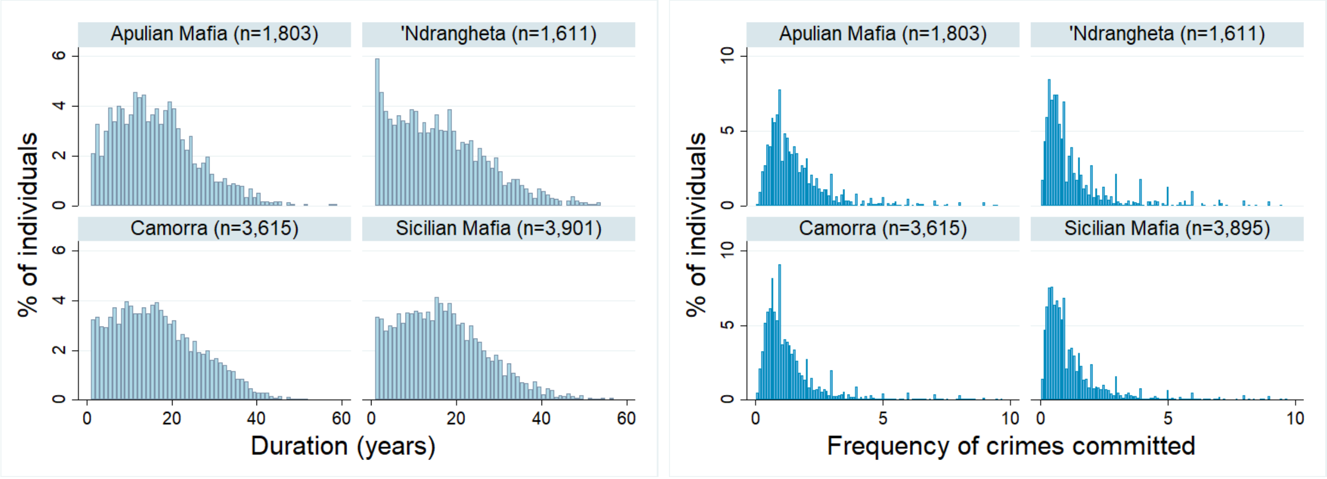

Figure 16. Distribution of the diversity index and of the escalation by crime number in the four Italian mafia organisations

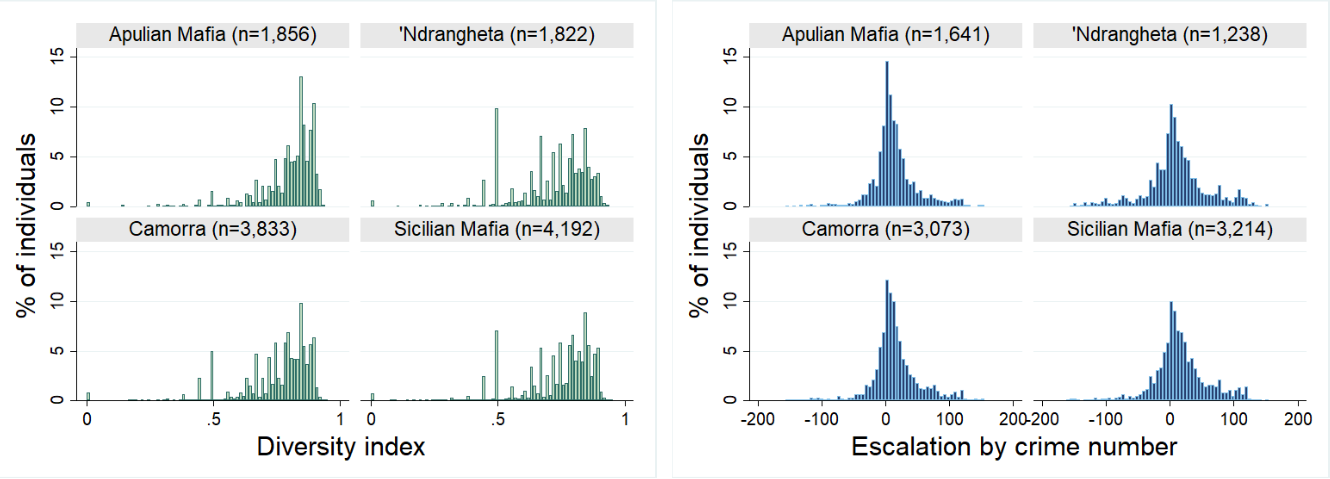

4.2.2 DIFFERENCES IN THE CRIMINAL CAREER OF MAFIA MEMBERS ACROSS THE TYPES OF MAFIA: LOGISTIC REGRESSIONS

Logistic regressions explore the differences in the criminal careers of members of different mafia types. The first specification shows that Camorra and Sicilian mafia members tend to commit more violent offenses compared to other mafia members, and Sicilian mafia members commit also more serious crimes (Table 7). However, Sicilian mafia members appear to be recruited at a later age compared to members of other mafia organisations. Compared to other mafia members, 'Ndrangheta members are more likely to reside in a region different from the one where they were born, while the opposite is true for Sicilian mafia members.

Table 7. Determinants of the mafia association: logistic regressions

	Dependent variable is a dummy for the mafia association			
	Sicilian Mafia	'Ndrangheta	Camorra	Apulian Mafia
Age at first crime	0.013<br>(0.011)	-0.034<br>(0.015)**	-0.018<br>(0.012)	0.013<br>(0.019)
Age at first MO	0.025<br>(0.008)***	0.009<br>(0.010)	-0.021<br>(0.008)***	-0.023<br>(0.011)**
N violent crimes	0.025<br>(0.008)***	-0.062<br>(0.016)***	0.019<br>(0.008)**	-0.046<br>(0.015)***
Mean crime seriousness	0.012<br>(0.001)***	-0.003<br>(0.002)*	-0.009<br>(0.002)***	-0.007<br>(0.002)***
Duration	0.006<br>(0.009)	-0.040<br>(0.013)***	-0.002<br>(0.010)	0.019<br>(0.014)
Frequency	-0.054<br>(0.041)	-0.062<br>(0.059)	-0.092<br>(0.045)**	0.217<br>(0.049)***
Diversity index	-0.270<br>(0.461)	-1.895<br>(0.563)***	-0.410<br>(0.494)	5.761<br>(0.887)***
Escalation (crime num.)	0.001<br>(0.001)	-0.004<br>(0.001)***	0.002<br>(0.001)	0.000<br>(0.002)
Transfer dummy	-0.275<br>(0.121)**	0.650<br>(0.143)***	0.015<br>(0.124)	-0.230<br>(0.174)
Years of education	0.011<br>(0.016)	0.061<br>(0.020)***	-0.023<br>(0.017)	-0.058<br>(0.024)**
Year of birth	-0.001<br>(0.008)	-0.036<br>(0.011)***	-0.017<br>(0.008)**	0.062<br>(0.012)***
Role indicators	YES	YES	YES	YES
Intercept	-0.876<br>(16.018)	71.391<br>(21.226)***	35.687<br>(16.795)**	-128.106<br>(23.304)***
Pseudo R^2	0.056	0.067	0.022	0.125
Observations	2,666	2,666	2,666	2,666

*Notes: standard errors reported in parenthesis. *, ** and *** indicate statistical significance at the 10, 5 and 1 per cent level, respectively.*

The second specification shows that members of different mafia organisations tend to commit different types of crimes (Table 8).[18] Offenders who committed a higher number of robbery, theft and related crimes, arson and assault crimes, drug-related crimes, administrative and financial crimes, associative crimes[19] and a lower number of long conviction crimes are more likely to be Apulian mafia members rather than members of other mafia organisations. On the other hand, Sicilian mafia members are characterised by a higher number of long conviction crimes and a lower number of robbery, theft and related crimes, drug-related crimes and administrative and financial crimes. Compared to other mafia members, 'Ndrangheta members commit a lower number of long conviction crimes, associative crimes and robbery, theft and related crimes. Camorra members tend to commit more long conviction crimes and less drug-related and associative crimes.

[18] Crimes have been grouped through a Principal Component Analysis (PCA) that enabled to reduce the 31 crime categories into six groupings (see the Annex in section 2.2).

[19] Excluding mafia association, the other associative crimes are criminal association and drug trafficking criminal association.

Table 8. Determinants of the mafia association (type of committed crimes): logistic regressions

	Dependent variable is a dummy for the mafia association			
	Sicilian Mafia	‘Ndrangheta	Camorra	Apulian Mafia
Age at first crime	0.021<br>(0.010)**	-0.033<br>(0.013)***	-0.021<br>(0.011)*	-0.005<br>(0.017)
Age at first MO	0.012<br>(0.007)*	0.001<br>(0.009)	-0.008<br>(0.007)	-0.025<br>(0.011)**
Duration	0.001<br>(0.008)	-0.028<br>(0.010)***	0.022<br>(0.008)***	-0.007<br>(0.012)
N long conviction crimes	0.012<br>(0.004)***	-0.020<br>(0.008)**	0.019<br>(0.004)***	-0.076<br>(0.011)***
N robbery, theft and related crimes	-0.019<br>(0.011)*	-0.064<br>(0.019)***	-0.003<br>(0.010)	0.086<br>(0.015)***
N arson and assault crimes	-0.025<br>(0.020)	-0.049<br>(0.033)	-0.033<br>(0.021)	0.143<br>(0.025)***
N drug-related crimes	-0.032<br>(0.019)*	0.014<br>(0.021)	-0.151<br>(0.030)***	0.128<br>(0.023)***
N admin, financial and road traffic crimes	-0.040<br>(0.018)**	-0.000<br>(0.015)	0.020<br>(0.013)	0.030<br>(0.017)*
N white-collar crimes	-0.023<br>(0.021)	0.006<br>(0.017)	0.029<br>(0.019)	-0.075<br>(0.058)
N associative crimes	-0.044<br>(0.063)	-0.160<br>(0.088)*	-0.164<br>(0.077)**	0.584<br>(0.078)***
Years of education	0.026<br>(0.014)*	0.052<br>(0.017)***	-0.046<br>(0.016)***	-0.048<br>(0.023)**
Year of birth	-0.018<br>(0.007)**	-0.030<br>(0.009)***	0.014<br>(0.008)*	0.049<br>(0.012)***
Role indicators	YES	YES	YES	YES
Intercept	34.607<br>(14.198)**	57.873<br>(18.333)***	-27.923<br>(15.667)*	-97.767<br>(23.171)***
Pseudo R^2	0.041	0.045	0.045	0.197
Observations	3,142	3,142	3,142	3,142

*Notes: standard errors reported in parenthesis. *, ** and *** indicate statistical significance at the 10, 5 and 1 per cent level, respectively.*

4.2.3 Trajectories of members across types of mafia

The analysis of criminal trajectories by type of mafias uncovers different patterns across the four mafia organisations (Figure 17). For technical details see Annex (section 3.2.1).

Figure 17. Group Based Trajectories by mafia type

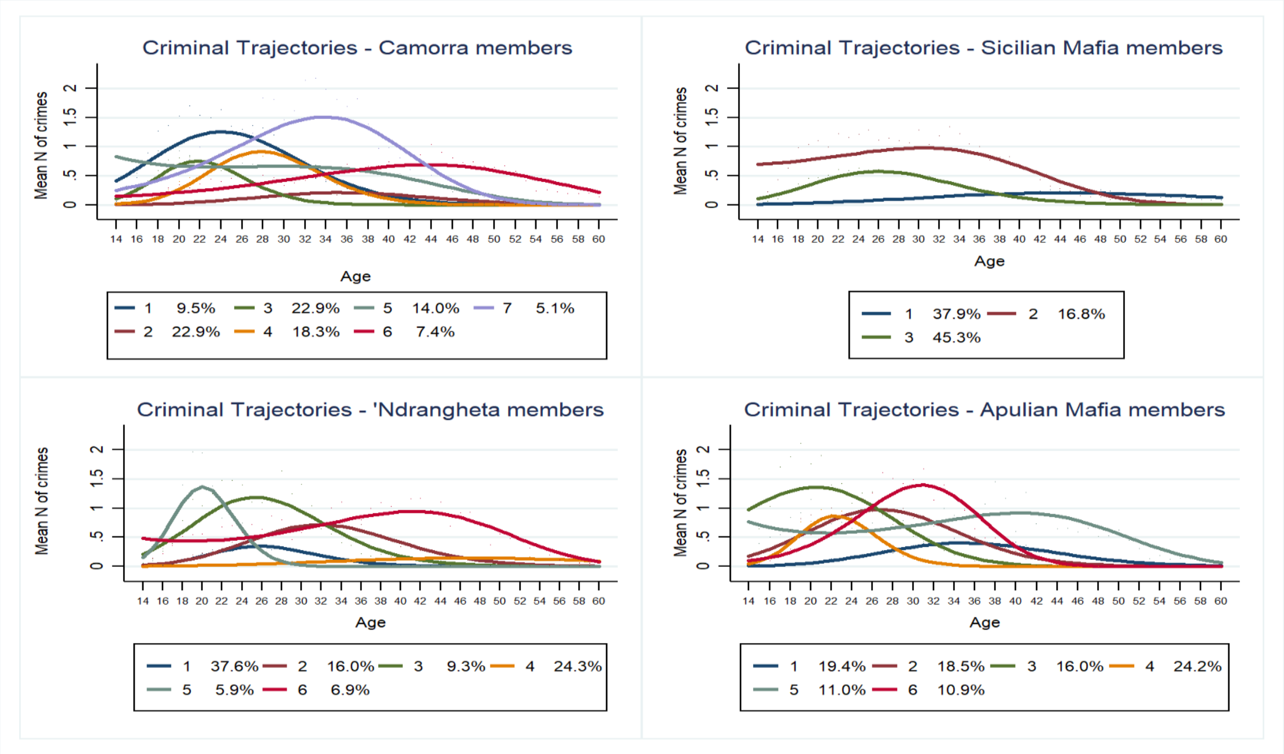

Specifically, Camorra and Apulian mafias report similar trajectories for some groups (e.g. Camorra groups 7 and 1 and Apulian groups 5 and 3, respectively). Alike, ‘Ndrangheta has similarities with Camorra and Apulian mafia (e.g. ‘Ndrangheta group 3 and Camorra group 1; also ‘Ndrangheta group 6 and Apulian group 5). Interestingly, Sicilian mafia shows different trends: they exhibit fewer groups, pointing out lower internal heterogeneity; groups' curves evolve more gradually than other mafias.

4.3 Micro level: exploring the drivers of the recruitment into the mafias

This subsection investigates the determinants of the age of recruitment into mafias at the individual level. More specifically, it addresses the following questions:

- *What social and environmental drivers influence the age of recruitment into mafias?*
- *What criminal characteristics influence the age of recruitment into mafias?*

4.3.1 Factors determining the age of recruitment: OLS regressions

Linear regressions analysed the determinants of the age of recruitment (i.e. the age of the first mafia offense, MO in the tables), and whether this variable differs among different mafia roles and mafia associations (Table 9).

Offenders who will become bosses in their mafia organisations tend to be recruited at an earlier age compared to affiliates. Moreover, more educated mafia members are recruited by the mafias when they are older.[20] This finding might have different explanations: more educated offenders may be better able to hide their criminal behaviour to law enforcement authorities, which did not detect some mafia association crimes they committed while they were younger. It might also be that offenders with higher education face more career options, and thus commit to the involvement into the mafia organisation at a later stage.

More versatile offenders who are able to commit violent crimes and who exhibit a higher escalation in crime seriousness are recruited into the mafias when they are younger. On the other hand, the higher mean seriousness of crimes committed before the first mafia offense is correlated with a later entry into the mafias. Finally, offenders who committed more robbery, thefts and related crimes and more associative crimes (other than mafia association) before their first mafia offense tend to be recruited by the mafia when they are younger. Conversely, offenders committing a higher number of drug-related crimes and white-collar crimes before recruitment enter the mafia organisations at a later age.

[20] This result is robust to employing as education variable both a proxy of the offender’s years of education and a categorical variable for the schooling degrees included in the dataset (specification in column (4); the base outcome is the elementary school qualification).

Table 9. Determinants of the age at recruitment: OLS regressions

	Dependent variable is the age at first mafia offense				
	(1)	(2)	(3)	(4)	(5)
Boss	-1.422<br>(0.497)***	-1.094<br>(0.458)**	-1.079<br>(0.458)**	-1.128<br>(0.458)**	-2.178<br>(0.429)***
Killer	-2.361<br>(1.256)*	-1.190<br>(1.080)	-1.222<br>(1.079)	-1.179<br>(1.078)	-1.296<br>(1.017)
Underboss/ Lieutenant	-0.931<br>(0.285)***	-0.303<br>(0.271)	-0.305<br>(0.271)	-0.313<br>(0.271)	-0.893<br>(0.244)***
‘Ndrangheta	1.375<br>(0.455)***	1.156<br>(0.460)**	1.134<br>(0.459)**	1.157<br>(0.459)**	0.435<br>(0.397)
Camorra	0.789<br>(0.402)**	-0.173<br>(0.366)	-0.139<br>(0.366)	-0.124<br>(0.367)	-0.111<br>(0.339)
Mafia Lucana	-0.359<br>(1.624)	0.987<br>(1.381)	1.053<br>(1.379)	1.023<br>(1.377)	-0.201<br>(1.301)
Sicilian Mafia	2.080<br>(0.386)***	0.760<br>(0.367)**	0.792<br>(0.366)**	0.824<br>(0.366)**	0.786<br>(0.331)**
Years of education	0.049<br>(0.044)	0.196<br>(0.046)***	0.201<br>(0.046)***		0.174<br>(0.040)***
Decade of birth	-6.398<br>(0.118)***	-2.730<br>(0.148)***	-2.708<br>(0.148)***	-2.697<br>(0.150)***	-3.681<br>(0.121)***
Mean seriousness pre MO.		0.014<br>(0.004)***	0.018<br>(0.004)***	0.018<br>(0.004)***	
Duration pre MO		0.756<br>(0.021)***	0.773<br>(0.022)***	0.774<br>(0.022)***	0.698<br>(0.016)***
Frequency pre MO		-0.209<br>(0.092)**	-0.057<br>(0.117)	-0.035<br>(0.117)	
Escalation pre MO		-0.012<br>(0.005)**	-0.013<br>(0.005)**	-0.013<br>(0.005)**	
Diversity index pre MO		-4.730<br>(0.909)***	-4.561<br>(0.911)***	-4.466<br>(0.910)***	
N violent crimes pre MO			-0.070<br>(0.033)**	-0.075<br>(0.033)**	
Education: academic degree				2.306<br>(1.245)*	
Education: high school				2.609<br>(0.524)***	
Education: illiterate				0.289<br>(1.155)	
Education: lit. but no formal ed.				-0.198<br>(0.669)	
Education: middle school				0.493<br>(0.261)*	
Education: prof. school				-0.152<br>(0.793)	
N long conv. crimes pre MO					0.005<br>(0.016)
N robbery, thefts crimes pre MO					-0.375<br>(0.033)***
N arson/assault crimes pre MO					-0.102<br>(0.066)
N drug crimes pre MO					0.165<br>(0.062)***
N admin and fin crimes pre MO					-0.011<br>(0.036)
N white-collar crimes pre MO					0.151<br>(0.044)***
N associative crimes pre MO					-0.653<br>(0.256)**
Intercept	64.368<br>(0.765)***	38.964<br>(1.175)***	38.252<br>(1.221)***	39.090<br>(1.190)***	43.653<br>(0.828)***
R^2	0.51	0.77	0.77	0.77	0.73
Observations	3,171	1,319	1,319	1,319	2,202

*Notes: standard errors reported in parenthesis. *, ** and *** indicate statistical significance at the 10, 5 and 1 per cent level, respectively. Base outcome for the role is the affiliate role, for the mafia association is the Apulian Mafia and for the education variable is the elementary school qualification.*

4.3.2 Factors determining the age of recruitment: multinomial logistic regressions

The previous analyses showed that the age of recruitment may significantly vary across the sample. While some members join the mafias during young adulthood, others enter in later ages. Drivers of early and late recruitment may vary significantly. This is assessed through multinomial logistic regressions, where the dependent variable is a categorical variable dividing the sample in “early recruits”, “late recruits” and offenders recruited at an “average” age (the baseline category) (Table 10).[21]

Early recruited mafia members are on average less educated than late recruits and committed more robberies, thefts and related crimes and less white-collar crimes compared to late recruits. Conversely, offenders recruited at an older age exhibit a lower escalation in crime seriousness and tend to be less versatile criminals.

[21] In Table 10, early recruits are identified as offenders whose age at recruitment is lower than the 25th percentile of the age of recruitment distribution, while the recruitment age of late recruits is higher than the 75th percentile of the same distribution. Results employing different percentile values can be found in the Annex in section 3.3. Some of the findings are robust to the change in the age limit defining “early” and “late” recruits.

Table 10. Drivers of early and late recruitment: multinomial logistic regressions

	Base outcome is "average" recruitment age (28-41 years old)			
	Early recruits (<28 years old)		Late recruits (>41 years old)	
Boss	0.354<br>(0.436)	0.964<br>(0.295)***	-1.253<br>(0.395)***	-1.328<br>(0.370)***
Killer	1.471<br>(0.704)**	0.555<br>(0.613)	-0.571<br>(1.132)	-1.014<br>(1.093)
Underboss/Lieutenant	0.197<br>(0.224)	0.424<br>(0.158)***	-0.632<br>(0.242)***	-0.636<br>(0.197)***
'Ndrangheta	-0.104<br>(0.362)	0.075<br>(0.250)	0.648<br>(0.434)	0.178<br>(0.366)
Camorra	0.146<br>(0.282)	0.041<br>(0.212)	-0.087<br>(0.379)	-0.438<br>(0.337)
Mafia Lucana	-0.128<br>(0.948)	0.438<br>(0.719)	0.809<br>(1.216)	-0.803<br>(1.176)
Sicilian Mafia	-0.081<br>(0.293)	-0.198<br>(0.204)	0.314<br>(0.373)	-0.109<br>(0.325)
Mean serious. pre MO	-0.009<br>(0.004)**		0.007<br>(0.003)**	
Duration pre MO	-0.537<br>(0.044)***	-0.386<br>(0.021)***	0.252<br>(0.024)***	0.197<br>(0.015)***
Frequency pre MO	-0.019<br>(0.089)		0.064<br>(0.187)	
Escalation pre MO	0.002<br>(0.004)		-0.020<br>(0.008)**	
Diversity index pre MO	2.261<br>(0.791)***		-2.109<br>(0.799)***	
N violent crimes pre MO	0.019<br>(0.034)		-0.065<br>(0.037)*	
N long conviction crimes pre MO		-0.014<br>(0.014)		-0.018<br>(0.013)
N robbery, theft and related crimes pre MO		0.158<br>(0.024)***		-0.211<br>(0.033)***
N arson and assault crimes pre MO		0.083<br>(0.047)*		-0.018<br>(0.055)
N drug-related crimes pre MO		-0.136<br>(0.065)**		0.050<br>(0.057)
N admin, financial, road traffic crimes pre MO		0.078<br>(0.039)**		-0.025<br>(0.022)
N white-collar crimes pre MO		-0.251<br>(0.106)**		0.232<br>(0.089)***
N associative crimes pre MO		0.476<br>(0.201)**		-0.388<br>(0.184)**
Years of education	-0.084<br>(0.040)**	-0.075<br>(0.026)***	0.083<br>(0.038)**	0.087<br>(0.030)***
Year of birth	0.105<br>(0.015)***	0.138<br>(0.010)***	-0.165<br>(0.017)***	-0.192<br>(0.013)***
Intercept	-203.058<br>(28.639)***	-268.936<br>(19.904)***	319.107<br>(33.116)***	372.586<br>(26.341)***
Pseudo R^2	0.509	0.496	0.509	0.496
Observations	1,319	2,202	1,319	2,202

*Notes: standard errors reported in parenthesis. *, ** and *** indicate statistical significance at the 10, 5 and 1 per cent level, respectively. Early recruits are offenders whose age at recruitment is lower than the 25th percentile of the age at recruitment distribution, while late recruits' age at recruitment is higher than the 75th percentile. Base outcome for the role is the affiliate role and for the mafia association is the Apulian Mafia.*

4.3.3 Factors determining the age at recruitment in the four mafias: OLS regressions

A last set of regressions investigates whether the recruitment mechanism differs in the four Italian mafia organisations (Table 11.).[22]

In the 'Ndrangheta, offenders with a higher offending frequency before the first mafia offense tend to be recruited into the mafia at younger age (although the frequency coefficient is statistically significant only at the 10 per cent level). In the Camorra, the key parameter leading to an earlier recruitment is a high diversity index value: offenders committing more diverse crimes are recruited by the Camorra at younger age. In the Sicilian Mafia, offenders exhibiting a higher escalation in crime seriousness and who committed a higher number of violent crimes (controlling for the length of the offending period) are recruited when they are younger. Finally, there seems to be no relevant factor within the Apulian mafia leading to an early age recruitment. However, both within the Apulian and the Sicilian mafia, offenders committing more serious crimes tend to enter in the mafia organisation when they are older.

Finally, the positive relationship between the education level and the age at first mafia offense is statistically significant only in the two mafia organisations with larger sample size: Camorra and Sicilian mafia.

[22] Unfortunately, the sample size is quite small in all four regression models (and especially for the Apulian Mafia and the 'Ndrangheta), thus weakening the reliability of the derived results.

Table 11. Determinants of the age at recruitment in the four Italian mafia organisations: OLS regressions

	Dependent variable is the age at first mafia offense			
	(1)	(2)	(3)	(4)
	Apulian Mafia	'Ndrangheta	Camorra	Sicilian Mafia
Boss	-0.931<br>(0.962)	-2.336<br>(1.316)*	-1.038<br>(0.748)	-0.587<br>(0.805)
Killer	-1.090<br>(3.441)	-5.566<br>(4.932)	-2.602<br>(1.308)**	1.315<br>(2.054)
Underboss/ Lieutenant	0.028<br>(0.545)	-1.874<br>(0.843)**	-0.784<br>(0.432)*	0.354<br>(0.483)
Mean seriousness pre MO	0.032<br>(0.011)***	-0.005<br>(0.014)	0.001<br>(0.008)	0.021<br>(0.006)***
Duration pre MO	0.779<br>(0.049)***	0.612<br>(0.072)***	0.839<br>(0.037)***	0.771<br>(0.038)***
Frequency pre MO	-0.185<br>(0.214)	-1.014<br>(0.529)*	-0.132<br>(0.264)	0.074<br>(0.169)
Escalation pre MO	-0.000<br>(0.009)	-0.013<br>(0.017)	0.012<br>(0.011)	-0.029<br>(0.008)***
Diversity index pre MO	-2.558<br>(1.821)	-5.703<br>(2.964)*	-7.473<br>(1.469)***	-2.702<br>(1.670)
N violent crimes pre MO	-0.107<br>(0.088)	-0.033<br>(0.173)	0.026<br>(0.065)	-0.098<br>(0.045)**
Years of education	-0.013<br>(0.105)	0.222<br>(0.153)	0.137<br>(0.071)*	0.364<br>(0.079)***
Decade of birth	-2.601<br>(0.353)***	-3.658<br>(0.497)***	-1.773<br>(0.231)***	-3.126<br>(0.255)***
Intercept	36.994<br>(2.467)***	50.225<br>(4.101)***	35.936<br>(1.851)***	38.088<br>(2.102)***
R^2	0.83	0.77	0.77	0.76
Observations	219	162	413	515

*Notes: standard errors reported in parenthesis. *, ** and *** indicate statistical significance at the 10, 5 and 1 per cent level, respectively. Base outcome for the role is the affiliate role.*

5 Results: the criminal careers of mafia bosses

This section presents the analyses focusing on mafia leaders. Mafia leaders comprise offenders in the sample whose role was either boss or underboss. The macro level focuses on the characteristics of the mafia leaders' population. The meso level explores similarities and differences of mafia leaders across mafia types. The micro level analyses individual characteristics predicting leadership within mafias.

5.1 Macro level: the parameters of the criminal careers of mafia bosses

This subsection examines the criminal career's parameters of mafia bosses and underbosses compared to mafia affiliates and killers. Furthermore, it presents different developmental trajectories of mafia leaders and compares their careers before and after the recruitment into mafias. The subsection addresses the following questions:

- *What are the main characteristics of the leaders of Italian mafias?*
- *How do mafia leaders' careers evolve over time?*
- *What are the patterns in mafia members' career before and after the recruitment into mafias?*

5.1.1 DESCRIPTIVE STATISTICS OF THE CRIMINAL CAREERS OF MAFIA BOSSES AND UNDERBOSSES

Mafia bosses and underbosses exhibit a higher average internal participation to crime compared to simple affiliates (Table 12 and Figure 18).[23] Overall, mafia leaders commit more diverse crimes (Figure 20) over a longer time span (Figure 19), and their lower escalation values (Figure 20) might be a consequence of the fact that they commit more serious crimes since the early years of their criminal career.[24]

[23] Mafia members identified as killers have the highest average internal participation: 18.03%. However, the number of mafia members identified as killers is low: they are only 48 individuals. As a consequence, results for killers must be interpreted with caution.

[24] The mean seriousness of the offenses committed before the first mafia offense is 69.57 for mafia leaders and 57.41 for mafia affiliates.

Table 12. Descriptive statistics of the criminal career's parameters for the four mafia roles

	Role in the mafia organisation			
**Parameter**	**Boss**	**Underboss**	**Killer**	**Affiliate**
Participation (internal)†	17.20%	16.57%	18.03%	14.70%
Duration***	19.03 (11.13)	16.98 (10.66)	15.17 (11.02)	12.91 (9.91)
Frequency	1.59 (2.29)	1.64 (2.00)	2.25 (1.96)	1.61 (1.76)
Specialisation***	0.78 (0.14)	0.77 (0.13)	0.80 (0.11)	0.74 (0.15)
Escalation (by crime number)	14.02 (33.50)	14.09 (39.34)	4.01 (50.43)	16.16 (43.62)
Escalation (by age)	4.01 (12.63)	4.32 (16.25)	2.46 (25.81)	5.36 (20.56)

*Notes: standard deviations reported in parenthesis. † For internal participation, the average participation over time is shown. For all the other parameters, the average across individual level values is presented. ANOVA tests have been run for all the parameters included in this table except for the participation: ***, ** and * indicate statistically significant differences among mafia associations at the 1, 5 and 10 per cent level.*

Figure 18. Trend of the internal participation by role

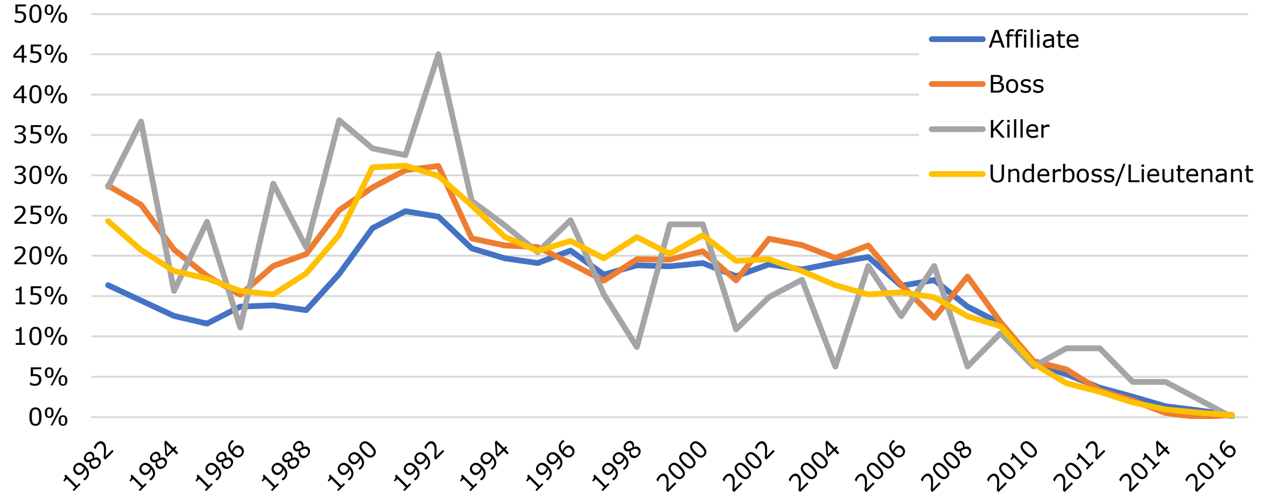

Figure 19. Distribution of the duration and of the offending frequency by role

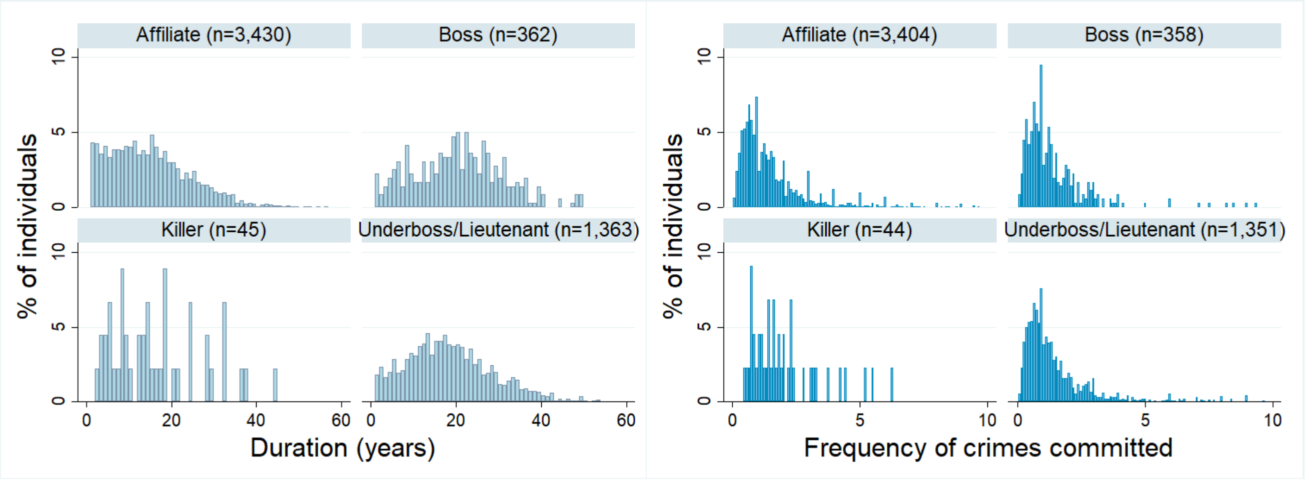

Figure 20. Distribution of the diversity index and of the escalation by crime number by role

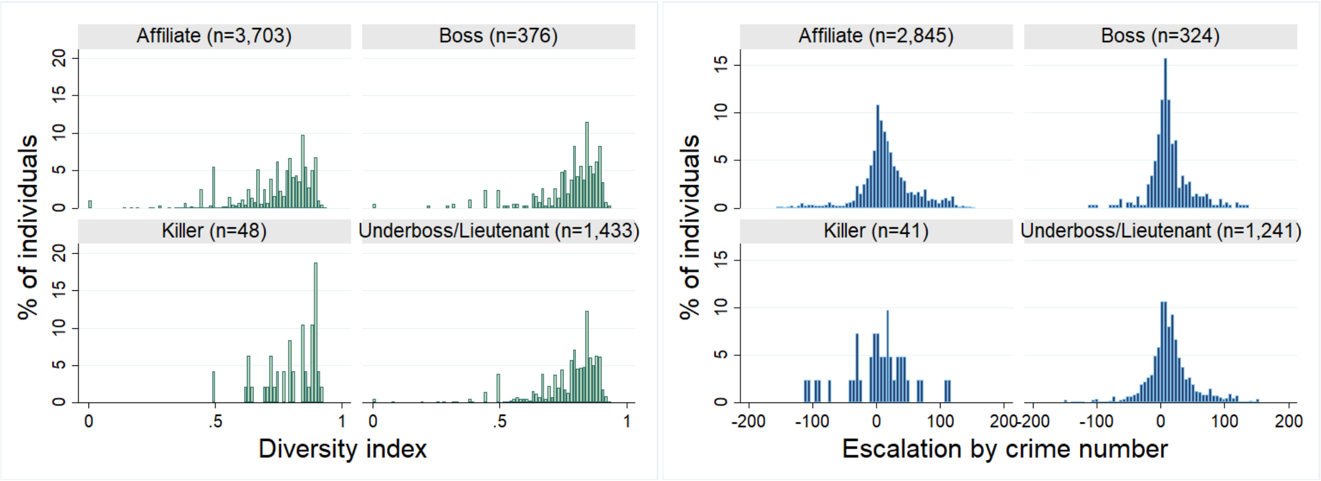

5.1.2 THE TRAJECTORIES OF MAFIA LEADERS

GBTM for mafia leaders yielded five separate groups (Figure 21). For technical details, see Annex (section 4.1.1).

Figure 21. Group Based Trajectories for mafia bosses

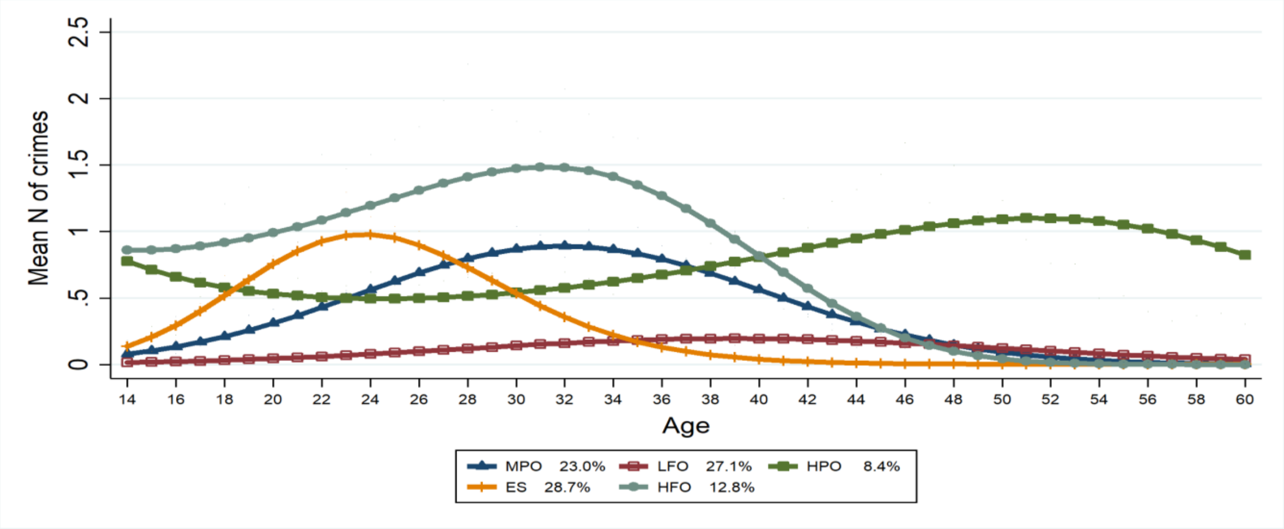

Note: n=1,870.

The leaders' trajectories share significant similarities with those analysed for the mafia population (Figure 22). Both for mafia members in general and for mafia bosses (which account for only 14.13% of mafia members), GBTM identified the same number of trajectories, with surprisingly similar paths. The similarity points out that mafia members follow specific career patterns regardless of their role, although leaders exhibit higher average values.

Figure 22. Trajectory comparison between whole mafia population and mafia bosses

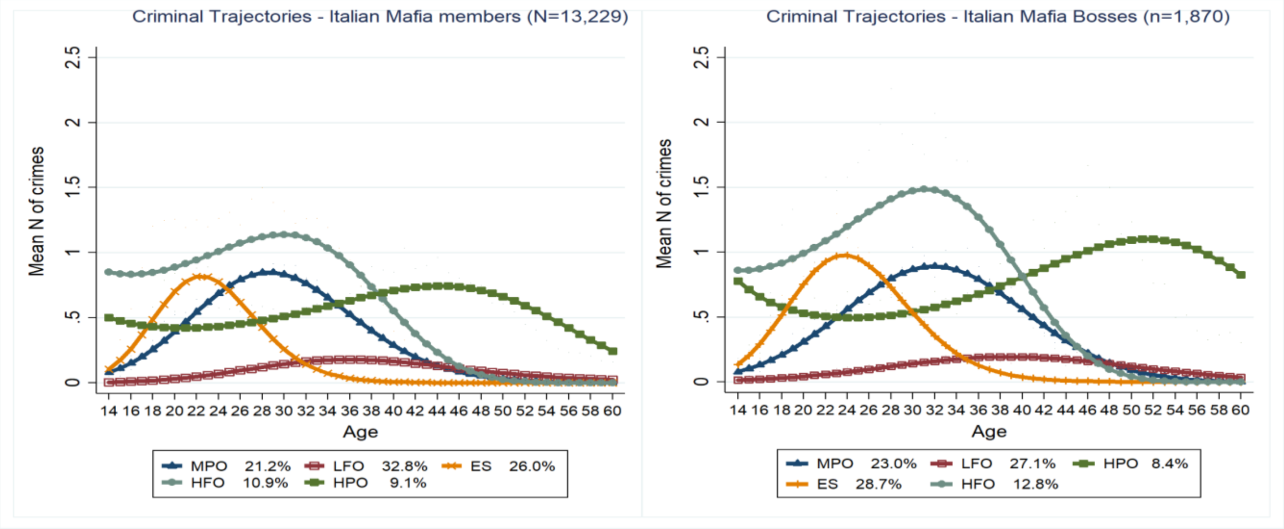

Similarly to the whole mafia members population, GBTM grouped mafia leaders in five groups:

- Group 1 (*Moderate Persistence Offenders*, MPO): MPO commit on average 23.05 offenses in their career, with frequency equal to 1.15 offenses per year.

- Group 2 (*Low Frequency Offenders*, LFO): LFO commit the lowest number of offenses (6.94), with the lowest frequency (0.88).
- Group 3 (High *Persistence Offenders*, PO): HPO remain active also in their late adulthood. Moreover, they commit a high number of offenses (43.99) with frequency around the population average (1.53).
- Group 4 (*Early Starters*, ES): ES engage in crime earlier than all the others. On average, they commit 16.44 offenses over their careers with a frequency of 1.68 offenses/year.
- Group 5 (*High Frequency Offende*rs, HFO): HFO commit the higher number of offenses (47.97) with the overall highest frequency (3.74 offenses per year).

Table 13. Criminal features of mafia leaders' trajectory groups

Group		Moderate Persistence Offenders (1)	Low Frequency Offenders (2)	High Persistence Offenders (3)	Early Starters (4)	High Frequency Offenders (5)	Pop Average
Career	Avg age at 1st crime	21.60	30.85	22.02	19.57	20.53	*23.40*
	Avg age 1st arrest	38.96	46.23	44.80	31.55	33.17	*38.64*
	Avg age 1st Mafia ass.	35.87	42.21	41.05	27.98	31.19	*34.98*
	Avg N of crimes	23.05	6.94	43.99	16.44	47.97	*21.42*
	Avg N of crime cat.	10.02	4.35	12.88	8.13	11.62	*8.31*
	Average Seriousness	83.01	115.68	80.36	85.80	92.92	*94.03*
	Avg N of violent crimes	4.85	1.31	11.01	3.38	14.53	*5.14*
Pre-Mafia	Avg N of crimes pre-mafia	11.08	2.76	19.86	9.42	24.50	*11.29*
	Avg Seriousness pre-Mafia	66.97	66.52	67.94	67.33	83.36	*69.57*
	Avg N of violent crimes pre-mafia	2.86	1.10	4.73	2.57	7.59	*3.31*
Criminal Career Parameters	Avg Duration	22.20	13.97	29.61	13.00	18.08	*17.40*
	Avg Frequency	1.15	0.88	1.53	1.68	3.74	*1.63*
	Avg Diversity Index	0.82	0.68	0.83	0.79	0.79	*0.77*
	Avg Escalation Age	4.07	3.68	2.95	5.39	3.78	*4.26*
	Avg Escalation Order	13.39	22.11	8.79	13.50	9.93	*14.07*

Notes: each distribution is divided into three colour classes. For Avg age at 1st *crime, Avg age 1*st *arrest, and Avg age 1*st *mafia association, colour red means values below the average-(st.dev./2), while green ones are values above average+(st.dev./2). Yellow values are comprised between these two thresholds. For all the other variables, green and red are inverted. ANOVA tests revealed statistical significant differences across groups for all variables (Prob>F=.000) except for Escalation Order (significance at .9 level)*

In particular, MPO report values in line with population average, with the notable exception of a very high diversity index score and a long career duration. For LFO, the only exceptions are the highest seriousness and order escalation. HPO exhibit the highest diversity and duration. Moreover, they are above the average for number of crimes (both overall and pre-mafia), of crime categories, of violent crimes. ES report the lowest age values in relation to career onset. Additionally, they show high diversity index and age escalation scores. Finally, HFO exhibit values above the population mean for all the

variables, except for seriousness, career duration, diversity and escalation (both age and order).

5.1.3 THE CRIMINAL CAREERS OF LEADERS BEFORE AND AFTER RECRUITMENT

To analysis of the differences in the criminal career of mafia leaders, before and after the recruitment into mafias, focused on the subset of leaders (N=984) with information on the year of the mafia association offense (see Annex, section 3.1.2). Similarly to what was done for the entire population of mafia members, a new variable (year_recruitment) reported the values of year_crime for the first mafia association. For each mafia leader, the six dimensions of duration, frequency, specialization, escalation, number of crimes, and average seriousness were computed for both periods before and after recruitment (see Annex, section 3.1.2).

Figure 23 shows the distributions of the different estimated parameters of the criminal careers of mafia leaders, before and after recruitment into mafias, respectively. The mean values are plotted as vertical red lines and are also shown in Table 14.

Figure 23. Distributions of the criminal career's parameters of mafia leaders, before and after recruitment

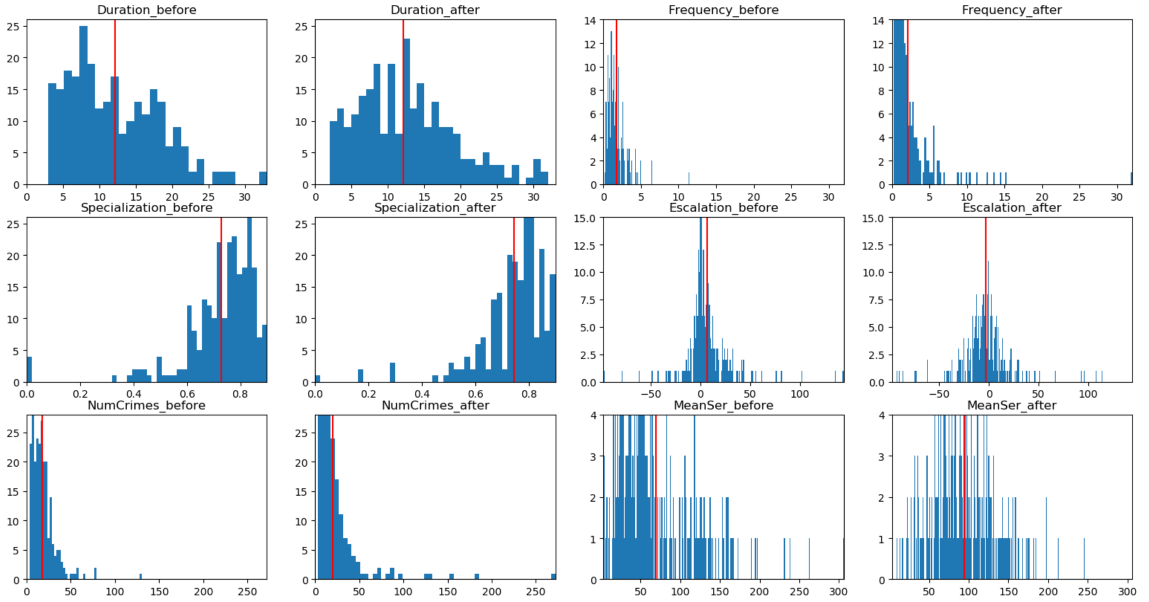

Note: mean values are plotted as vertical red lines.

Table 14. Mean values of the criminal career's parameters of mafia leaders before and after recruitment

Parameter	pre/post	Mean value	T-test score	p-value	H_0
Duration	Before	12.12	0.0	1.000	not rejected
	After	12.12			
Frequency	Before	1.75	-1.66	0.090	not rejected
	After	2.10			
Specialization	Before	0.73	-1.49	0.130	not rejected
	After	0.75			
Escalation	Before	6.72	4.72	0.000	rejected
	After	-3.44			
Number of crimes	Before	17.92	-1.03	0.300	not rejected
	After	19.91			
Average seriousness	Before	69.75	-6.14	0.000	rejected
	After	94.06			

The statistical significance of the differences before and after recruitment was assessed with T-tests. Results show a statistical difference in the mean values of the parameters escalation and mean seriousness (Table 14). Escalation is significantly lower after recruitment, while the average seriousness is significantly higher. As observed for the entire population of mafia members, this suggests an escalation in the criminal pattern of mafia members before recruitment, followed by more serious offenses after the recruitment moment.

The analysis of the seriousness and number of crimes before and after recruitment confirms this trend. Across the years, the average seriousness increases sharply until recruitment, when it peaks. Subsequently, it drops at a similar pace (Figure 24, upper pane). The decrease is quite likely exaggerated by the prison sentences imposed on most mafia leaders. Indeed, analysis of "active years" confirms the escalation until the peak in the recruitment year, but uncovers a stabilisation in the post-recruitment seriousness.

Figure 24. Average seriousness before and after recruitment, leaders only. Actual years (upper pane) and "active years" (lower pane)

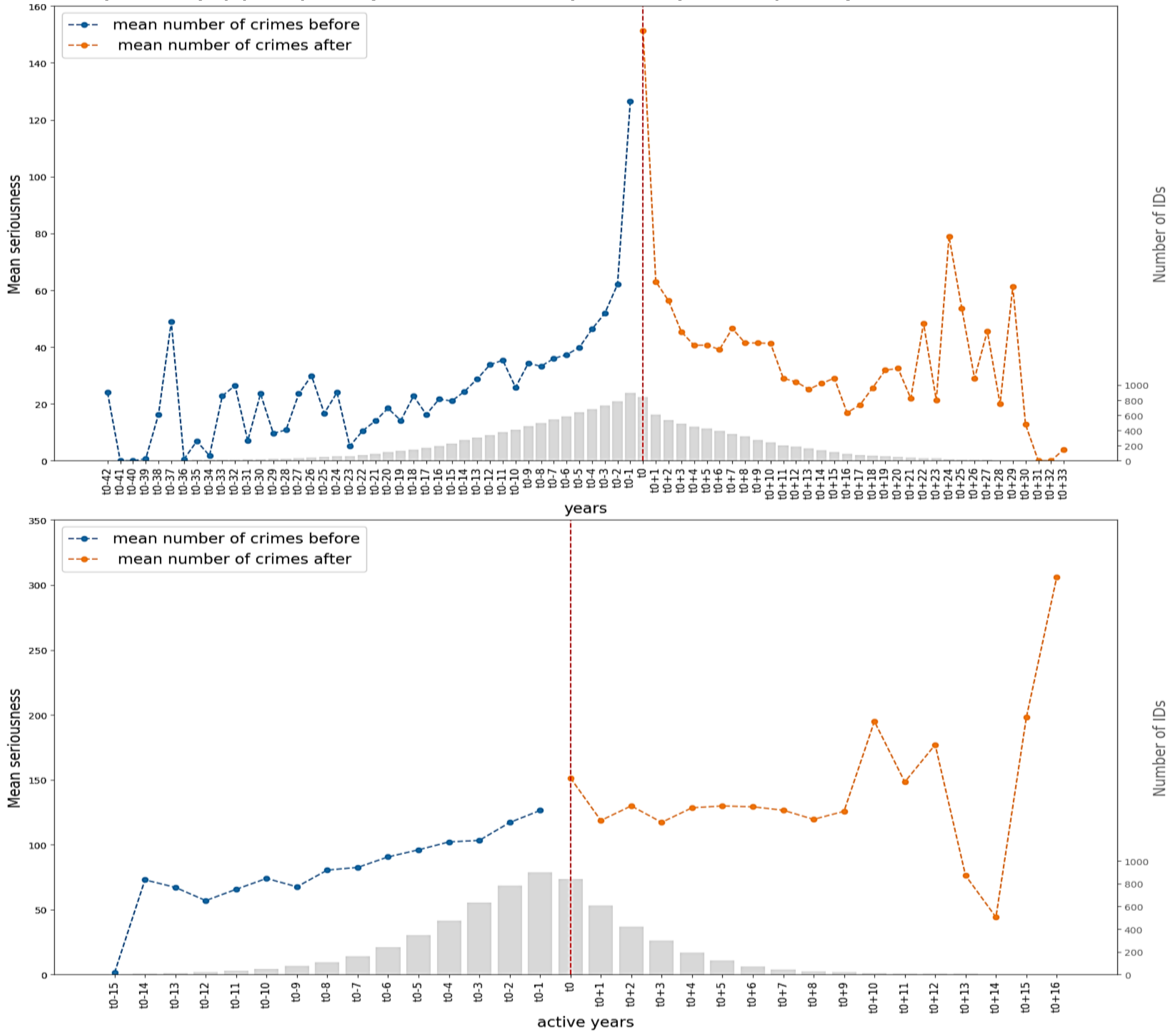

Notes: t0 is the recruitment moment (year of the first mafia offense).

The escalation/de-escalation pattern appears also in the analysis of the number of crimes (Figure 25). Focus on active years shows that in fact the number of crimes slightly drops immediately after recruitment, but then increases again. This may suggest that achievement of leadership requires maintaining a high level of offending, although small sample size for active years after recruitment impairs the possibility to assess this pattern in detail.

Figure 25. Average number of crimes before and after recruitment, leaders only. Actual years (upper pane) and "active years" (lower pane).

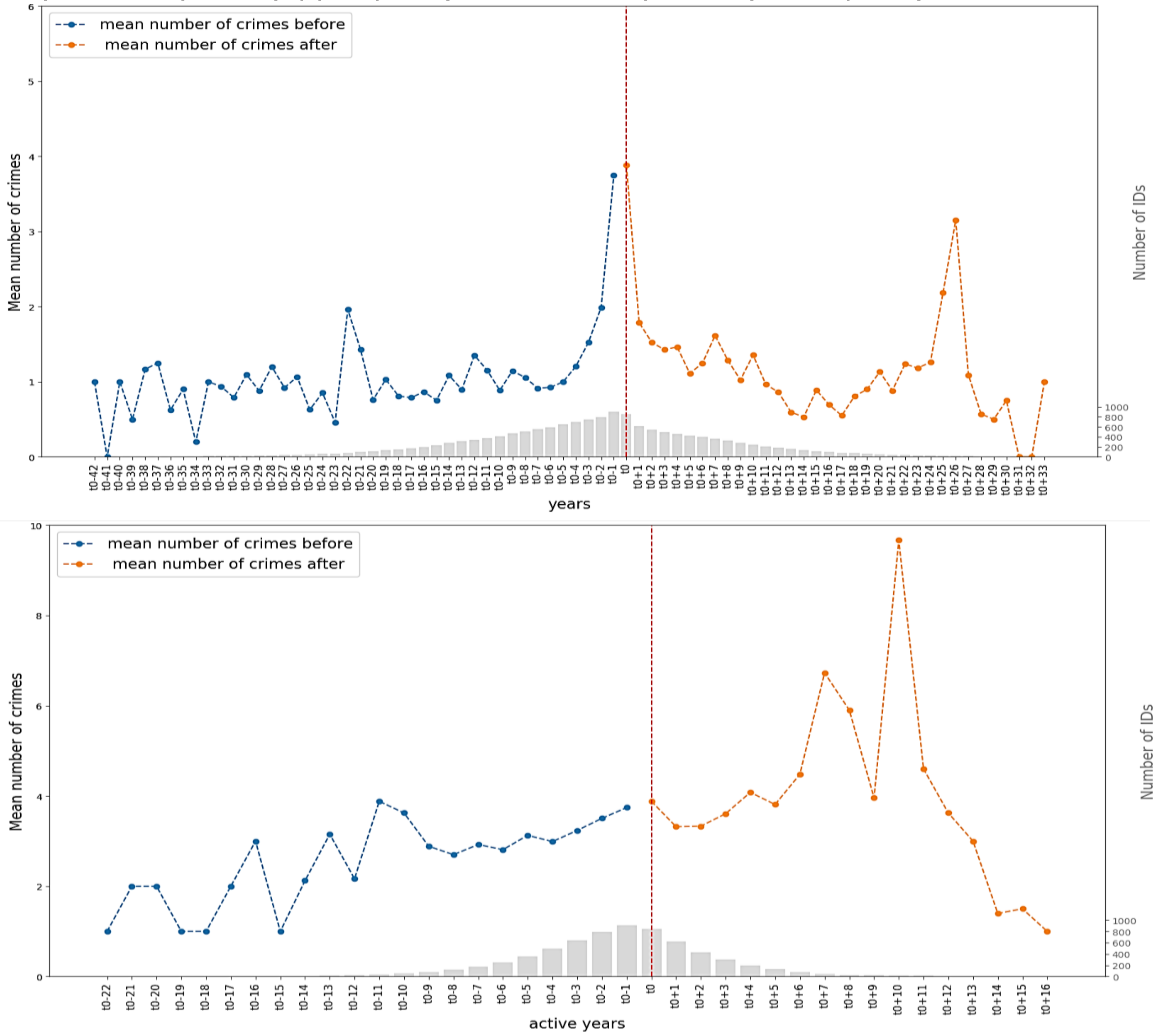

Notes: t0 is the recruitment moment (year of the first mafia offense).

Overall, the patterns are similar to those for mafia members in general (see above subsection 4.1.3). Mafia leaders escalate the number and especially the seriousness of crimes before recruitment. The year of recruitment is an important turning point in their career. Compared to the whole sample of mafia members, leaders exhibit higher average values.

5.2 Meso level: mafia bosses across types of mafias

This subsection analyses the five parameters related of the criminal careers of Italian mafia leaders by mafia type. Also, it compares trajectories and analyse the existing differences and similarities in the criminal careers of mafia leaders by type of mafia. The subsection answers to the following questions:

- *Do mafia leaders parameters differ across different mafias?*
- *What are the developmental trajectories of mafia leaders across different mafias?*
- *What are the significant differences of leaders belonging to different mafias?*

5.2.1 DESCRIPTIVE STATISTICS OF THE CRIMINAL CAREERS OF MAFIA LEADERS BY TYPE OF MAFIA

Mafia leaders across different mafia organisations differ in terms of their criminal careers' parameters (Table 15). On average, a higher share of Apulian mafia leaders are active offenders in any given year: this is especially evident over the period 1982-1996; after 1996, the internal participation to crime of Apulian mafia leaders starts exhibiting values similar to the ones of other mafia organisations (Figure 26). Like Apulian mafia members, Apulian mafia leaders commit more diverse crimes (Figure 28) at higher frequency (Figure 27) and exhibit the higher escalation in crime seriousness as they become older. Conversely, 'Ndrangheta mafia leaders have the lowest average escalation value, both when examining escalation by crime number and by age. The 'Ndrangheta has also higher shares of mafia leaders with low offending frequency, resulting in an average frequency of 1.23 crimes per year in the whole mafia leaders' sample (a substantially lower value compared to the average offending frequency of the other mafia leaders).

Table 15. Descriptive statistics of the criminal career's parameters for mafia leaders in the four Italian mafia organisations

Parameter	Apulian Mafia leaders	'Ndrangheta leaders	Camorra leaders	Sicilian Mafia leaders
Participation (internal)†	22.47%	13.30%	17.62%	16.51%
Duration	16.95<br>(9.13)	17.54<br>(11.50)	17.44<br>(10.79)	17.52<br>(10.82)
Frequency***	1.99<br>(1.66)	1.23<br>(1.53)	1.68<br>(2.20)	1.72<br>(2.29)
Specialisation***	0.82<br>(0.11)	0.75<br>(0.14)	0.78<br>(0.12)	0.76<br>(0.14)
Escalation**<br>(by crime number)	15.80<br>(27.94)	7.96<br>(43.20)	16.77<br>(34.49)	14.39<br>(41.73)
Escalation***<br>(by age)	7.34<br>(16.19)	2.37<br>(17.38)	5.65<br>(14.34)	2.50<br>(14.61)

*Notes: standard deviations reported in parenthesis. † For internal participation, the average participation over time is shown. For all the other parameters, the average across individual level values is presented. ANOVA tests have been run for all the parameters included in this table except for the participation: ***, ** and * indicate statistically significant differences among mafia associations at the 1, 5 and 10 per cent level.*

Figure 26. Trend of the internal participation to crime of mafia leaders in the four Italian mafia organisations

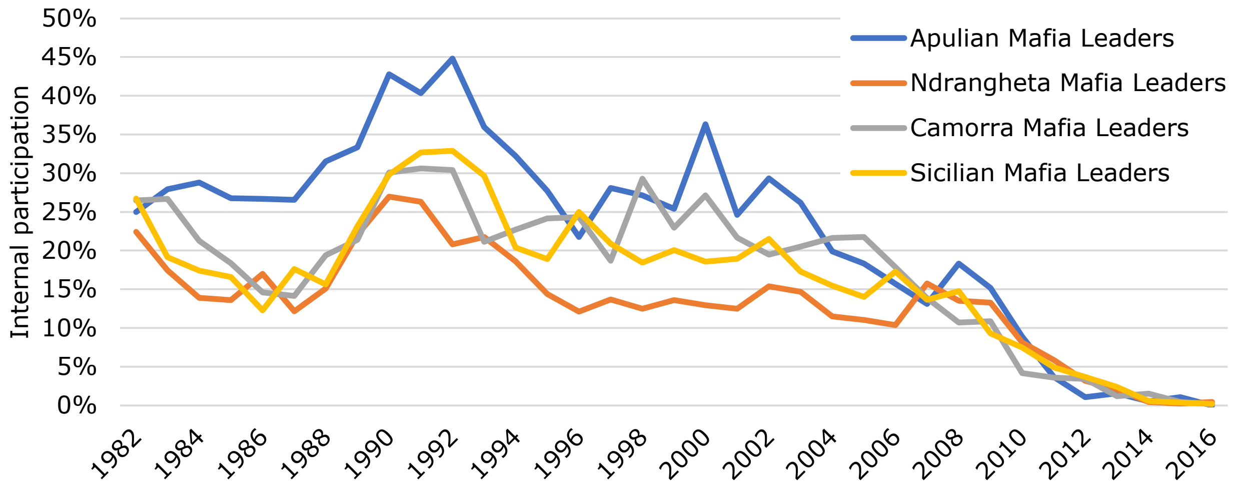

Figure 27. Distribution of the duration and of the offending frequency of mafia leaders in the four Italian mafia organisations

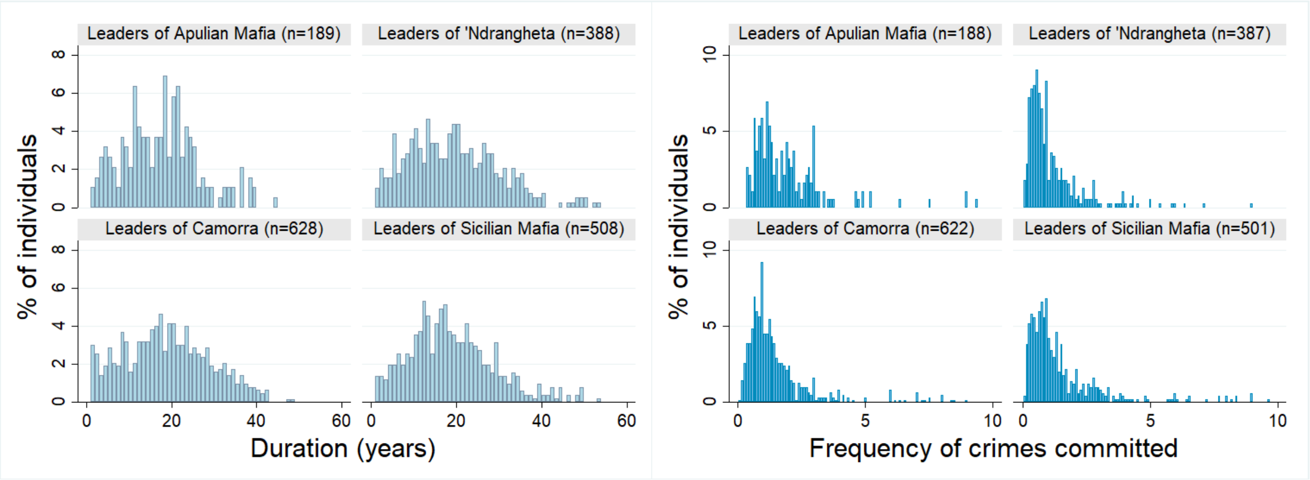

Figure 28. Distribution of the diversity index and of the escalation by crime number of mafia leaders in the four Italian mafia organisations

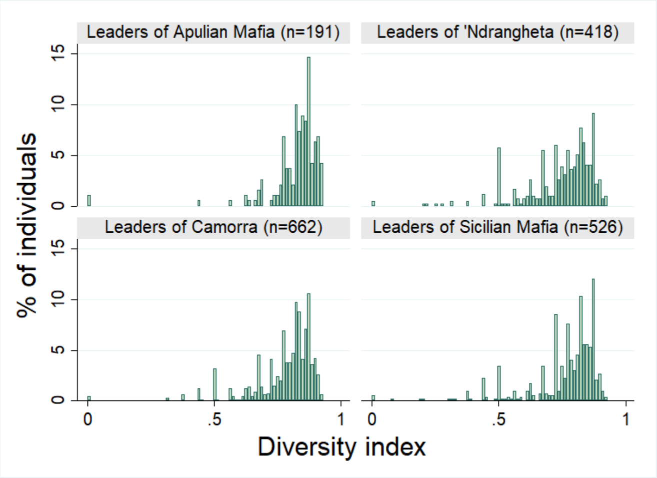

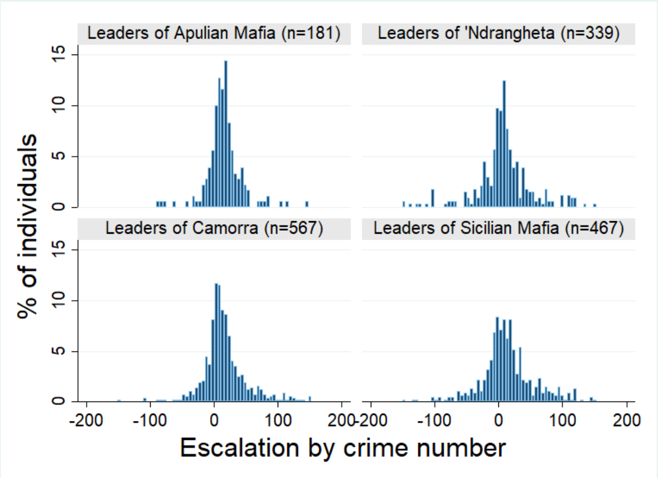

5.2.2 DIFFERENCES IN THE CRIMINAL CAREER OF MAFIA LEADERS ACROSS THE TYPES OF MAFIA: LOGISTIC REGRESSIONS

Table 16 and Table 17 explore the differences among the criminal careers of leaders of different mafia types.

Sicilian mafia leaders tend to commit more serious and violent crimes compared to leaders of other mafia organisations. However, Sicilian mafia leaders tend to commit their first crime when they are older compared other mafia leaders, while the age at first crime is significantly lower among 'Ndrangheta and Camorra leaders.

Mafia leaders committing more robbery, theft and related crimes, arson and assault crimes, associative crimes, drug-related crimes and less long conviction crimes are more likely to be leaders in the Apulian mafia. Conversely, leaders committing more long conviction crimes and less robbery, theft and related crimes are more likely to be Sicilian mafia leaders. 'Ndrangheta leaders exhibit a lower number of arson and assault crimes compared to other mafia leaders, and Camorra leaders tend to commit more long conviction crimes and less drug-related crimes.

Overall, differences among mafia leaders of different mafia types closely reflect the differences among members of different mafia types (see Table 7 and Table 8).

Table 16. Determinants of the association of mafia leaders: logistic regressions

	Dependent variable is a dummy for the mafia association			
	Sicilian Mafia	'Ndrangheta	Camorra	Apulian Mafia
Age at first crime	0.075<br>(0.020)***	-0.070<br>(0.022)***	-0.055<br>(0.022)**	0.010<br>(0.031)
Age at first MO	0.011<br>(0.012)	0.018<br>(0.014)	-0.024<br>(0.012)**	-0.013<br>(0.018)
N violent crimes	0.015<br>(0.009)*	-0.043<br>(0.018)**	0.014<br>(0.010)	-0.014<br>(0.017)
Mean crime serious.	0.013<br>(0.002)***	-0.004<br>(0.003)	-0.008<br>(0.003)***	-0.010<br>(0.004)***
Duration	0.032<br>(0.015)**	-0.051<br>(0.017)***	0.002<br>(0.015)	-0.007<br>(0.023)
Frequency	-0.000<br>(0.054)	-0.097<br>(0.083)	-0.050<br>(0.061)	0.118<br>(0.068)*
Diversity Index	0.081<br>(0.797)	-2.623<br>(0.801)***	0.080<br>(0.876)	10.145<br>(1.943)***
Escalation by crime number	0.002<br>(0.002)	-0.005<br>(0.002)**	0.002<br>(0.002)	0.000<br>(0.003)
Transfer dummy	-0.089<br>(0.201)	0.256<br>(0.219)	0.181<br>(0.201)	-0.483<br>(0.314)
Years of education	0.021<br>(0.025)	0.043<br>(0.028)	-0.029<br>(0.027)	-0.071<br>(0.039)*
Year of birth	0.023<br>(0.014)	-0.051<br>(0.015)***	-0.015<br>(0.014)	0.054<br>(0.020)***
Intercept	-49.231<br>(27.761)*	102.730<br>(30.664)***	31.563<br>(28.647)	-114.534<br>(41.068)***
Pseudo R^2	0.081	0.047	0.045	0.150
Observations	992	992	992	992

*Notes: standard errors reported in parenthesis. *, ** and *** indicate statistical significance at the 10, 5 and 1 per cent level, respectively.*

Table 17. Determinants of the association of mafia leaders (type of committed crimes): logistic regressions

	Dependent variable is a dummy for the mafia association			
	Sicilian Mafia	'Ndrangheta	Camorra	Apulian Mafia
Age at first crime	0.064<br>(0.017)***	-0.055<br>(0.018)***	-0.057<br>(0.020)***	-0.020<br>(0.030)
Age at first MO	-0.006<br>(0.011)	0.016<br>(0.012)	-0.009<br>(0.011)	-0.013<br>(0.019)
Duration	0.016<br>(0.013)	-0.031<br>(0.014)**	0.016<br>(0.014)	-0.017<br>(0.022)
N long conviction crimes	0.013<br>(0.005)**	-0.014<br>(0.009)	0.014<br>(0.005)***	-0.075<br>(0.018)***
N robbery, theft and related crimes	-0.056<br>(0.022)**	-0.031<br>(0.027)	0.005<br>(0.011)	0.095<br>(0.028)***
N arson and assault crimes	0.011<br>(0.028)	-0.087<br>(0.046)*	-0.038<br>(0.031)	0.144<br>(0.037)***
N drug-related crimes	-0.023<br>(0.035)	-0.023<br>(0.040)	-0.131<br>(0.047)***	0.166<br>(0.045)***
N admin, financial and road traffic crimes	-0.027<br>(0.023)	-0.014<br>(0.021)	0.024<br>(0.016)	0.028<br>(0.019)
N white-collar crimes	0.014<br>(0.022)	-0.081<br>(0.074)	0.018<br>(0.017)	-0.097<br>(0.098)
N associative crimes	-0.036<br>(0.099)	-0.043<br>(0.114)	-0.143<br>(0.114)	0.457<br>(0.125)***
Years of education	0.046<br>(0.023)**	0.032<br>(0.025)	-0.054<br>(0.026)**	-0.064<br>(0.040)
Year of birth	-0.005<br>(0.013)	-0.038<br>(0.014)***	0.014<br>(0.014)	0.045<br>(0.021)**
Intercept	8.282<br>(25.523)	75.178<br>(28.374)***	-25.942<br>(28.219)	-88.920<br>(41.977)**
Pseudo R^2	0.062	0.032	0.064	0.216
Observations	1,085	1,085	1,085	1,085

*Notes: standard errors reported in parenthesis. *, ** and *** indicate statistical significance at the 10, 5 and 1 per cent level, respectively.*

5.2.3 TRAJECTORIES OF LEADERS ACROSS TYPES OF MAFIA

The analysis highlights both similarities and differences between mafia types (Figure 29). For technical details, see Annex (section 4.2.1).

Figure 29. Trajectories of mafia leaders by type of mafia

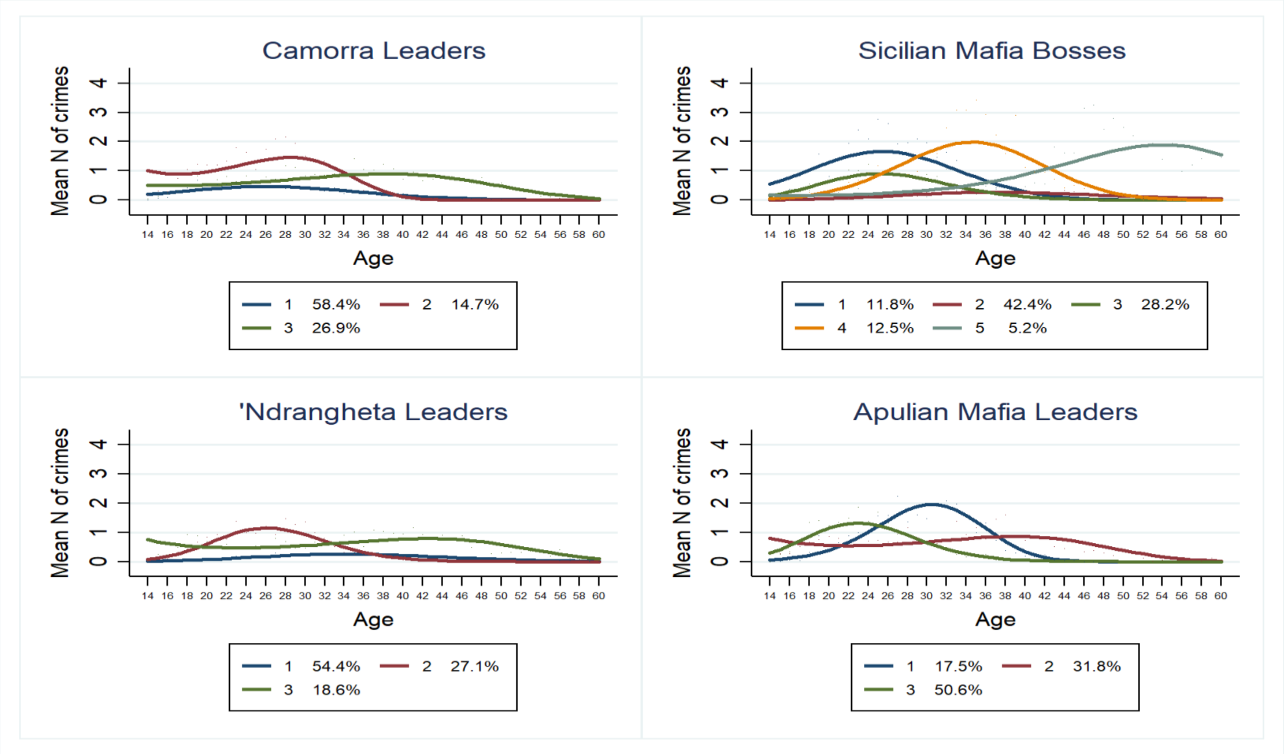

Sicilian mafia leaders show more diversified trajectories, with five groups compared to the three for all other mafias. Nevertheless, for all mafias, leaders that engage early in criminal activities tend to stop committing offenses earlier than other groups (probably due to a higher probability to get arrested in the early adulthood). In addition, leaders that peaks in their adulthood commit offenses also in the last part of their lives. Remarkably, the trajectories for Sicilian mafia members was clustered in fewer groups than other mafias (see 4.1.2). conversely, when focusing only on leaders, number of trajectories for the Sicilian mafia is higher than other mafias.

5.3 Micro level: exploring the drivers of mafia leadership

This subsection investigates whether the criminal careers of mafia leaders differ from those of simple affiliates. It also explores whether the probability of becoming a mafia leader is influenced by specific criminal behaviours during the first part of the criminal career. It addresses the following questions:

- *What are the individual characteristics that predict mafia leadership?*
- *What are the characteristics of mafia leaders in their early criminal careers?*

5.3.1 CHARACTERISTICS OF MAFIA LEADERS

The criminal career of mafia leaders is significantly different from the criminal career of simple affiliates (Table 18). On average, mafia leaders are more serious offenders compared to affiliates: they committed more serious and more diverse crimes over a longer career and with higher offending frequency. Mafia leaders are also on average more educated compared to affiliates.[25]

Offenders with a leading role in all the mafia organisations considered in the analysis have longer criminal careers and committed more serious crimes (see from columns 3 to 6 in Table 18). However, mafia leaders exhibit differences in their criminal career among different mafia organisations. Leaders of the 'Ndrangheta do not display a higher crime frequency compared to affiliates, and only leaders in the Apulian Mafia and in Camorra have a significantly higher value of the diversity index compared to affiliates. Moreover, only leaders of the Sicilian Mafia committed a higher number of violent crimes compared to affiliates.

---

[25] This result is robust to employing two different education variables: the number of years of schooling corresponding to the schooling degree assigned to individuals in the original dataset (column (1)) and a categorical variable for such schooling degrees (column (2); the base outcome is the academic degree).

Table 18. Characteristics of mafia leaders: logistic regressions

	Dependent variable is the "leading role" dummy					
	(1)	(2)	(3)	(4)	(5)	(6)
	Whole sample	Whole sample	Apulia	'Ndrangheta	Camorra	Sicily
Duration	0.051<br>(0.007)***	0.051<br>(0.007)***	0.059<br>(0.021)***	0.057<br>(0.017)***	0.063<br>(0.015)***	0.041<br>(0.011)***
Frequency	0.157<br>(0.042)***	0.158<br>(0.042)***	0.320<br>(0.111)***	0.081<br>(0.094)	0.196<br>(0.089)**	0.113<br>(0.061)*
Diversity Index	1.136<br>(0.477)**	1.190<br>(0.479)**	4.694<br>(1.862)**	0.332<br>(1.062)	1.956<br>(1.075)*	0.800<br>(0.696)
Escalation by age	0.003<br>(0.002)	0.004<br>(0.002)	-0.001<br>(0.006)	0.005<br>(0.005)	0.012<br>(0.006)**	-0.001<br>(0.004)
N violent crimes	0.026<br>(0.009)***	0.026<br>(0.009)***	-0.007<br>(0.020)	0.035<br>(0.031)	0.028<br>(0.018)	0.030<br>(0.013)**
Mean seriousness	0.015<br>(0.002)***	0.015<br>(0.002)***	0.016<br>(0.004)***	0.012<br>(0.004)***	0.017<br>(0.003)***	0.016<br>(0.002)***
N of prison entrances	0.216<br>(0.061)***	0.217<br>(0.061)***	0.031<br>(0.171)	0.085<br>(0.179)	0.265<br>(0.108)**	0.273<br>(0.094)***
Age at first MO	0.001<br>(0.007)	0.001<br>(0.007)	0.012<br>(0.023)	-0.007<br>(0.015)	-0.003<br>(0.013)	0.003<br>(0.012)
Year of birth	-0.011<br>(0.007)	-0.011<br>(0.007)	-0.017<br>(0.023)	-0.019<br>(0.016)	-0.001<br>(0.014)	-0.012<br>(0.012)
Years of education	0.050<br>(0.016)***		0.036<br>(0.051)	0.029<br>(0.040)	0.030<br>(0.031)	0.073<br>(0.025)***
Education: elementary school		-0.939<br>(0.407)**				
Education: high school		-0.402<br>(0.424)				
Education: illiterate		-1.087<br>(0.616)*				
Education: literate but no formal educ.		-0.760<br>(0.468)				
Education: middle school		-0.835<br>(0.404)**				
Education: profess. school		-0.969<br>(0.490)**				
Mafia ass. indicators	YES	YES	NO	NO	NO	NO
Intercept	17.797<br>(14.497)	17.545<br>(14.648)	26.276<br>(45.914)	34.080<br>(31.215)	-3.007<br>(27.541)	18.932<br>(24.155)
Pseudo R^2	0.097	0.098	0.076	0.085	0.087	0.100
Observations	2,693	2,693	423	402	785	1,066

*Notes: standard errors reported in parenthesis. *, ** and *** indicate statistical significance at the 10, 5 and 1 per cent level, respectively. Base outcome for the education variable is an academic degree.*

Table 19 presents the results from a multinomial logistic regression model where the dependent variable is the categorical variable for the role of the mafia member and the base comparison group is the role of affiliate in the mafia organisation. Results are very similar for mafia bosses and underbosses: both categories of mafia members have a longer criminal career, committed more serious and more violent crimes with higher frequency compared to affiliates. Moreover, both mafia bosses and underbosses have more years of education and more prison entrances compared to affiliates.

Table 19. Characteristics of mafia bosses and underbosses: multinomial logistic regressions

	Base outcome is the "affiliate" role	
	Boss	Underboss/Lieutenant
Duration	0.063<br>(0.012)***	0.051<br>(0.008)***
Frequency	0.162<br>(0.076)**	0.182<br>(0.044)***
Diversity Index	1.747<br>(0.907)*	1.106<br>(0.504)**
Escalation by age	0.002<br>(0.005)	0.003<br>(0.003)
N violent crimes	0.035<br>(0.012)***	0.021<br>(0.009)**
Mean seriousness	0.017<br>(0.003)***	0.015<br>(0.002)***
N prison entrances	0.304<br>(0.103)***	0.182<br>(0.065)***
Age at first MO	-0.000<br>(0.012)	0.001<br>(0.007)
Year of birth	-0.026<br>(0.013)**	-0.008<br>(0.008)
Years of education	0.058<br>(0.028)**	0.048<br>(0.017)***
Mafia ass. indicators	YES	YES
Intercept	44.149<br>(26.265)*	10.631<br>(15.439)
Pseudo R^2	0.083	0.083
Observations	2,693	2,693

*Notes: standard errors reported in parenthesis. *, ** and *** indicate statistical significance at the 10, 5 and 1 per cent level, respectively.*

5.3.2 CHARACTERISTICS OF MAFIA LEADERS BEFORE RECRUITMENT

After having compared the criminal career of mafia leaders and mafia affiliates, additional logit regression models explore whether the criminal career of mafia members before their first mafia offense (MO in the regression tables) influences their probability of becoming leaders in their mafia organisation (Table 20).

Mafia leaders were more serious offenders compared to affiliated even before being recruited by the mafias. Before their first mafia offense, individuals who acquired a leading role in their mafia organisation committed more serious crimes, more violent crimes and at higher frequency compared to affiliates. In addition, despite committing their first mafia offense at a younger age, mafia leaders have been active for a longer time when they first committed their first mafia offense.[26] Mafia leaders committed more long conviction crimes, more

[26] It might also be that mafia leaders have a better capacity in hiding their criminal behaviour to law enforcement authorities.

white-collar crimes and more associative crimes before their first mafia offense compared to affiliates, while they committed a lower number of robbery, theft and related crimes (column (5)).

Table 20. Pre-recruitment characteristics of mafia leaders: logistic regressions

	Dependent variable is the "leading role" dummy				
	(1)	(2)	(3)	(4)	(5)
Duration pre MO	0.040 (0.008)***	0.026 (0.007)***	0.029 (0.007)***	0.015 (0.009)*	0.035 (0.010)***
Mean seriousness pre MO	0.006 (0.001)***	0.006 (0.001)***	0.005 (0.001)***		
Frequency pre MO	0.073 (0.027)***				
N crimes pre MO		0.013 (0.004)***		0.017 (0.004)***	
N violent crimes pre MO			0.038 (0.011)***		
N associative crimes pre MO				0.166 (0.099)*	
N long conviction crimes pre MO					0.059 (0.009)***
N robbery, theft and related crimes pre MO					-0.080 (0.017)***
N arson and assault crimes pre MO					0.034 (0.033)
N drug-related crimes pre MO					0.036 (0.024)
N admin, financial and road trafficking crimes pre MO					0.002 (0.017)
N white-collar crimes pre MO					0.125 (0.059)**
Age at first MO	-0.042 (0.008)***	-0.040 (0.008)***	-0.041 (0.008)***	-0.042 (0.009)***	-0.056 (0.010)***
Year of birth	-0.050 (0.006)***	-0.052 (0.006)***	-0.053 (0.006)***	-0.055 (0.007)***	-0.057 (0.007)***
Years of education	0.037 (0.016)**	0.037 (0.015)**	0.037 (0.015)**	0.046 (0.017)***	0.048 (0.018)***
Mafia ass. indicators	YES	YES	YES	YES	YES
Intercept	97.615 (12.285)***	101.981 (11.825)***	104.375 (11.802)***	109.018 (13.415)***	112.872 (13.787)***
Pseudo R^2	0.062	0.064	0.064	0.056	0.073
Observations	2,705	2,879	2,879	2,202	2,202

*Notes: standard errors reported in parenthesis. *, ** and *** indicate statistical significance at the 10, 5 and 1 per cent level, respectively.*

5.3.3 CHARACTERISTICS OF MAFIA LEADERS DURING THEIR EARLY CRIMINAL CAREER

The analysis so far has focused on investigating whether certain characteristics of mafia offenders before their recruitment into the mafias influence their probability of becoming mafia leaders. However, most of the individuals in the sample commit their first mafia association crime quite late in their criminal career (on average at 34 years, Figure 30). Additional sets of logistic

regressions assess whether the criminal career in earlier ages (i.e. <35 y.o.) has an impact on their probability of becoming mafia leaders.

Figure 30. Distribution of the age at first mafia offense

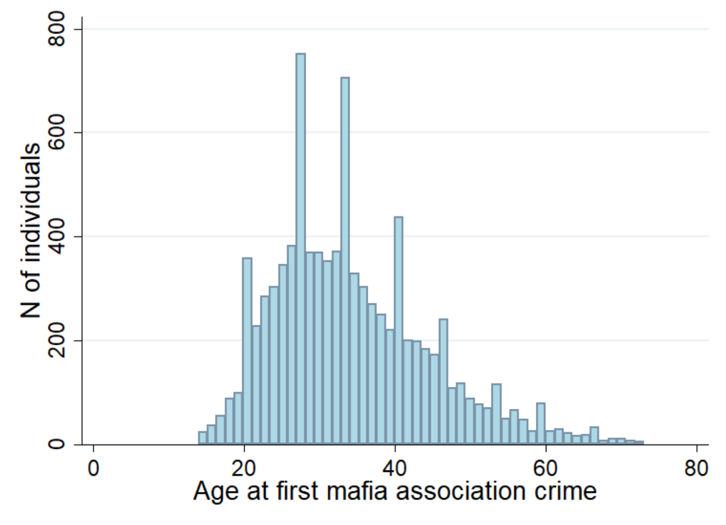

Note: n=8,983.

The number of total crimes, of violent crimes, and the mean seriousness of offenses committed in early adult life (i.e. between age 14 and 20, and between age 14 and 25) have little impact on the probability of acquiring a leading role in the mafia organisation (Table 21). Conversely, the number of total crimes and the number of violent crimes committed between 14 and 30, and between 25 and 30 positively affect the probability of becoming a mafia leader (Table 22). The models suggest that the differences between leaders and other members emerge when offenders are aged between 25 and 30. The difference persists at later stages, as the numbers of total and violent crimes committed at age 30-35, together with their higher seriousness, positively affect the probability of becoming a leader.

Table 21. Characteristics of mafia leaders at age 14-25: logistic regressions

	Dependent variable is the "leading role" dummy			
	(1)	(2)	(3)	(4)
N crimes age 14-20	0.011<br>(0.013)			
N violent crimes age 14-20		0.003<br>(0.043)		
Mean crime serious. age 14-20	0.001<br>(0.001)	0.001<br>(0.001)		
N crimes age 14-25			0.011<br>(0.006)*	
N violent crimes age 14-25				0.022<br>(0.019)
Mean crime serious. age 14-25			0.002<br>(0.001)**	0.002<br>(0.001)*
Age at first crime	0.016<br>(0.040)	0.001<br>(0.038)	-0.016<br>(0.018)	-0.023<br>(0.018)
Age at first MO	0.008<br>(0.010)	0.007<br>(0.010)	0.002<br>(0.008)	-0.000<br>(0.008)
Year of birth	-0.044<br>(0.009)***	-0.043<br>(0.009)***	-0.052<br>(0.007)***	-0.052<br>(0.007)***
Years of education	0.053<br>(0.024)**	0.053<br>(0.024)**	0.050<br>(0.018)***	0.050<br>(0.018)***
Mafia ass. indicators	YES	YES	YES	YES
Intercept	84.822<br>(17.096)***	84.226<br>(17.131)***	101.674<br>(13.210)***	102.199<br>(13.201)***
Pseudo R^2	0.060	0.059	0.049	0.048
Observations	1,377	1,377	2,226	2,226

*Notes: standard errors reported in parenthesis. *, ** and *** indicate statistical significance at the 10, 5 and 1 per cent level, respectively.*

Table 22. Characteristics of mafia leaders at age 14-35: logistic regressions

	Dependent variable is the "leading role" dummy					
	(1)	(2)	(3)	(4)	(5)	(6)
N crimes age 14-30	0.021<br>(0.004)***					
N violent crimes 14-30		0.047<br>(0.012)***				
Mean serious. age 14-30	0.003<br>(0.001)***	0.003<br>(0.001)***				
N crimes age 25-30			0.026<br>(0.006)***			
N violent crimes 25-30				0.066<br>(0.016)***		
Mean serious. age 25-30			0.002<br>(0.001)***	0.002<br>(0.001)**		
N crimes age 30-35					0.025<br>(0.006)***	
N violent crimes 30-35						0.064<br>(0.023)***
Mean serious. age 30-35					0.003<br>(0.001)***	0.003<br>(0.001)***
Age at first crime	-0.023<br>(0.013)*	-0.030<br>(0.013)**	-0.037<br>(0.013)***	-0.037<br>(0.013)***	-0.026<br>(0.010)**	-0.027<br>(0.010)***
Age at first mafia crime	0.005<br>(0.008)	0.000<br>(0.007)	-0.005<br>(0.008)	-0.006<br>(0.008)	-0.015<br>(0.008)*	-0.016<br>(0.008)*
Year of birth	-0.056<br>(0.006)***	-0.058<br>(0.006)***	-0.058<br>(0.007)***	-0.059<br>(0.007)***	-0.055<br>(0.007)***	-0.058<br>(0.007)***
Years of education	0.048<br>(0.016)***	0.049<br>(0.016)***	0.055<br>(0.018)***	0.055<br>(0.018)***	0.043<br>(0.018)**	0.043<br>(0.018)**
Mafia ass. indicators	YES	YES	YES	YES	YES	YES
Intercept	109.355<br>(12.316)***	112.735<br>(12.265)***	113.678<br>(13.634)***	116.473<br>(13.603)***	108.548<br>(14.523)***	113.086<br>(14.447)***
Pseudo R^2	0.064	0.062	0.063	0.062	0.060	0.057
Observations	2,635	2,635	2,128	2,128	1,938	1,938

*Notes: standard errors reported in parenthesis. *, ** and *** indicate statistical significance at the 10, 5 and 1 per cent level, respectively.*

6 Conclusions

This report analysed the social and criminal factors leading to recruitment into mafias, focusing on the criminal careers of mafia members and leaders. The analysis followed a three-level approach exploring the macro, meso, and micro dimension of mafia members' criminal careers.

Macro level

The career length of Italian *Mafiosi* is in line with the one found for other samples of serious offenders (Weisburd & Waring, 2001; Piquero et al., 2004). Moreover, Italian mafia members are versatile offenders and exhibit positive escalation in crime seriousness over their career. Nonetheless, available evidence suggests an actual decrease in their external and internal participation to crime in more recent years. This decrease may derive from an incapacitation effect (due to incarcerations) that weakened Italian mafias, or to limited data availability due to the duration of criminal proceedings for mafia cases.

Mafia members and leaders follow five different types of criminal trajectories. Notwithstanding the peculiarities of mafia offenders, the results of the different models are coherent with previous studies (van Koppen, de Poot, Kleemans, & Nieuwbeerta, 2010; van Onna, van der Geest, Huisman, & Denkers, 2014). Specifically, GBTM detected five groups both for mafia members and leaders: *Moderate Persistence Offenders (MPO), Low Frequency Offenders (LFO), Early Starters (ES), High Frequency Offenders (HFO), High Persistence Offenders (HPO)*. Postestimation analyses highlighted specific features of each group. MPO and HPO commit more heterogeneous offenses. LWO engage in criminal activities later, but they commit the most serious offenses overall. Conversely, ES have lower onset age and show steep escalation trends over time. Finally, HFO result as the most violent and frequent mafia members.

Surprisingly, trajectories for members and leaders were similar for number and shape. The main difference was leaders reporting higher frequencies. This suggests that mafia members can follow a finite number of career trajectories, regardless of the role within the organisation. The difference between members and leaders concern the frequency but not the overall career patterns. Furthermore, the number of offenses had limited power in differentiating between members and leaders.

Regression models provided further insight, highlighting some significant differences in the careers of mafia members and leaders. Leaders commit more serious and diverse crimes over a longer time span. Moreover, mafia leaders have a lower escalation in crime seriousness compared to affiliates.

This is likely due to the more serious offenses committed in early years of their criminal career.

Inferential models have explored the factors influencing the probability of becoming a leader in the mafia organisation. Mafia leaders are on average more serious offenders compared to affiliates even before being recruited into the mafia. Offenders who commit more serious crimes, a higher number of violent crimes and displayed a higher offending frequency before their first mafia association crime face a higher probability of becoming mafia leaders. Similarly, committing a higher number of long conviction, white-collar and associative crimes before recruitment also increases the probability of gaining leadership. From a developmental point of view, the early adulthood is crucial in determining future criminal career paths. In fact, the differences between mafia leaders and other mafia members (in terms of number, violence and seriousness of crimes committed) emerge when offenders are aged between 25 and 30 and persist at later ages.

MESO LEVEL

At the meso level, some differences arise from the subdivision of mafia members and leaders by mafia type. Taking into consideration the trajectories of members of the different mafias, the Sicilian mafia and the Camorra are the most homogeneous and heterogeneous mafias, with 3 and 7 groups, respectively. Some of the groups within Camorra share trajectory similarities with 'Ndrangheta and Apulian mafia. The Apulian mafia differs from all other types as it does not include a fraction of members exhibiting a very low frequency flat trend over time. This may be explained by the contrast between the Sacra Corona Unita and the Italian state law enforcement agencies. During the 80s and 90s, this mafia organisation was in the spotlight of law enforcement and this attention may have led to numerous convictions, thus increasing data on the frequency of Apulian mafia members overall.

Apart from these disparities, mafia types share also a stable similarity: each mafia type comprises a group of early starters. This is consistent with the results at macro level, where the models include early starters. They are the proof that a part of the Italian mafia population follows a criminal path that originates and peaks in the very first part of members' life.

Differences across organisations arise also when focusing only on mafia leaders. Differently from the whole Sicilian mafia population, Sicilian leaders are the most heterogeneous ones, with five distinct trajectory groups. Conversely, leaders of all the other mafias are divided only into three groups. Camorra and 'Ndrangheta leaders share very gradual increasing trends, while Sicilian and Apulian mafia leaders show some steep trends. This finding might reflect differences in the process of leadership acquisition in the four mafias.

Data suggest that Camorra and 'Ndrangheta leaders follow more linear trends in their crime frequency, while in many cases Sicilian and Apulian leaders exhibit, at different points in time, evident crime frequency escalations.

Regressions showed that mafia members and leaders in different mafia types present some differences, but the low value of the model fit measure in all specifications suggests that the differences are scarcely relevant in defining the type of mafia affiliation. Nonetheless, regressions highlighted that Apulian mafia members and leaders have more intense criminal careers compared to members and leaders of other organisations. They committed more diverse crimes, at higher frequency, with the highest escalation in crime seriousness and over a longer time span. Sicilian mafia members and leaders are the most serious in terms of mean seriousness and number of violent crimes. Moreover, together with members and leaders of the Camorra, Sicilian mafia members and leaders commit more long conviction crimes compared to other mafia members. From a geographical perspective, models have highlighted that 'Ndrangheta mafia members are significantly more likely to operate in a region different from the one of origin compared to other mafia members.

Micro level

The analysis has focused on the factors influencing the age of recruitment into mafias at the individual level. Factors explaining an early recruitment age include a high number of violent offenses, a high level of versatility and a high crime seriousness escalation. Another factor positively correlated with an early age recruitment is the number of robberies, thefts and related crimes committed before the first mafia offense. These results suggest that very active and violent offenders are more likely to enter the mafias earlier than other types of offenders. Moreover, having a high level of education is positively correlated with being recruited when older. This finding might have different explanations: more educated mafia members might have a better capacity in hiding from law enforcement authorities, who are unable to detect some mafia association crimes committed at younger age. It may also be that more educated mafia members face more career options and thus commit to the involvement into the mafia at a later stage.

Finally, analyses on the age of recruitment highlighted differences by mafia roles. Offenders who will become mafia leaders are recruited when they are younger than mere affiliates. Notwithstanding the young age of recruitment, future mafia leaders exhibit longer duration before recruitment.

Considering the relevance of recruitment in understanding mafia careers, the analysis focused on criminal careers parameters before and after the recruitment. The results show that mafia members report increasing number and seriousness of offenses in the years before recruitment. Afterwards, the

offending patterns stabilises, possibly due also to convictions and detentions. Besides the role of conviction and detection, this behaviour might be interpreted through two hypotheses. On one hand, once inside the organisation, mafia members may no longer need to commit serious offenses to demonstrate their value; thus they may gradually reduce crime seriousness or decide to reduce offending activity to minimise the risk of detection (*Protective de-escalation*). On the other hand, the lack of escalation can be explained by a cyclical turnover within the organisation. New recruits substitute older members, and the organisation itself imposes a change in the tasks of the older recruits that have successfully passed the training period (*Structural de-escalation*). Additional data may help in shedding light on the actual motivations behind these dynamics.

Policy implications

The analysis highlighted different patterns that, in many cases, start during early adulthood. Mafia members, and especially leaders, start their careers early and continue offending for several years, well beyond traditional desistance thresholds.

Mafia members also commit a significant number of crimes before recruitment, offering insight for designing specific crime prevention and law enforcement strategies. On the preventive side, young males at early stages of patterns similar to the mafia trajectories might be offered specific programmes to prevent further escalation. On the law enforcement side, the number and frequency of offenses committed by individuals already into the path to recruitment may justify more serious responses aiming at incapacitation and simultaneously disruption of the potential pool of mafia recruits. In a broader policy perspective, the study of mafia careers may contribute to develop models and strategies aiming at an early identification of individuals more likely to become a leader and consequent tailored interventions.

Future research

Future research may expand in several directions. First, analyses may focus on specific parameters of criminal careers, assessing what affects e.g. the frequency or the duration of mafia careers. Furthermore, research may assess whether and how mafias have changed over the last decades. For instance, data allow to investigate the spatial dimension of Italian mafias to understand whether mafia members have moved to non-traditional geographical areas and to what extent. The behavioural evolution of mafia members in terms of criminal history may point out new patterns in the criminal careers of mafia members when far from the traditional territories. Finally, research may try to evaluate the impact of the anti-mafia policies in Italy. For example, future analyses may demonstrate whether the decline in participation is just the

result of the duration of criminal proceedings or corresponds to an actual decrease in the participation in the mafias due to cultural changes or law enforcement pressure.

References

Blumstein, A. (2016). From Incapacitation to Criminal Careers. *Journal of Research in Crime and Delinquency*, *53*(3), 291–305. https://doi.org/10.1177/0022427815622020

Blumstein, A., Cohen, J., & Farrington, D. P. (1988). Criminal Career Research: Its Value for Criminology. *Criminology*, *26*(1), 1–35. https://doi.org/10.1111/j.1745-9125.1988.tb00829.x

Blumstein, A., Cohen, J., & Hsieh, P. (1982). *The duration of adult criminal careers: final report to National Institute of Justice*. National Institute of Justice, Carnegie-Mellon University School of Urban and Public.

Blumstein, A., Cohen, J., Roth, J. A., & Visher, C. A. (1986). *Criminal careers and "career criminals"* (National Research Council (U S.) Panel on Research on Criminal Careers). Washington, DC: National Academy Press.

Blumstein, A., Farrington, D. P., & Moitra, S. (1985). Delinquency Careers: Innocents, Desisters, and Persisters. *Crime and Justice*, *6*, 187–219.

Burt, R. S. (1992). *Structural Holes: The Social Structure of Competition*. Cambridge: Harvard University Press.

Burt, R. S. (2005). *Brokerage and closure: An introduction to social capital*. Oxford: Oxford University Press.

Clarke, R. V., & Felson, M. (1993). *Routine Activity and Rational Choice*. Transaction Publishers.

Cullen, F. T., Agnew, R., & Wilcox, P. (2013). *Criminological Theory: Past to Present: Essential Readings* (5 edition). New York: Oxford University Press.

DeLisi, M., & Piquero, A. R. (2011). New frontiers in criminal careers research, 2000–2011: A state-of-the-art review. *Journal of Criminal Justice*, *39*(4), 289–301. https://doi.org/10.1016/j.jcrimjus.2011.05.001

Farrington, D. P. (1987). Predicting Individual Crime Rates. *Crime and Justice*, *9*, 53–101.

Farrington, D. P. (1992). Criminal career research in the United Kingdom. *The British Journal of Criminology*, *32*(4), 521–536.

Farrington, D. P., & Hawkins, J. D. (1991). Predicting participation, early onset and later persistence in officially recorded offending. *Criminal Behaviour and Mental Health*, *1*(1), 1–33. https://doi.org/10.1002/cbm.1991.1.1.1

Farrington, D. P., Lambert, S., & West, D. J. (1998). Criminal Careers of Two Generations of Family Members in the Cambridge Study in Delinquent Development. *Studies on Crime and Crime Prevention*, *7*(1), 85–106.

Farrington, D. P., MacLeod, J. F., & Piquero, A. R. (2016). Mathematical Models of Criminal Careers. *Journal of Research in Crime and Delinquency*, *53*(3), 336–355. https://doi.org/10.1177/0022427815620237

Francis, B., Humphreys, L., Kirby, S., & Soothill, K. (2013). *Understanding criminal careers in organised crime* (Report). Home Office. Retrieved from http://eprints.lancs.ac.uk/67009/

Gottfredson, M., & Hirschi, T. (1986). The True Value of Lambda Would Appear to Be Zero: An Essay on Career Criminals, Criminal Careers, Selective Incapacitation, Cohort Studies, and Related Topics. *Criminology*, *24*(2), 213–234. https://doi.org/10.1111/j.1745-9125.1986.tb01494.x

Gottfredson, M., & Hirschi, T. (1987). The Methodological Adequacy of Longitudinal Research on Crime. *Criminology*, *25*(3), 581–614. https://doi.org/10.1111/j.1745-9125.1987.tb00812.x

Gottfredson, M., & Hirschi, T. (1988). Science, Public Policy, and the Career Paradigm. *Criminology*, *26*(1), 37–55. https://doi.org/10.1111/j.1745-9125.1988.tb00830.x

Greenwood, P. W., & Abrahamse, A. F. (1982). *Selective Incapacitation*. Rand Corporation.

Horney, J., Osgood, D. W., & Marshall, I. H. (1995). Criminal Careers in the Short-Term: Intra-Individual Variability in Crime and Its Relation to Local Life Circumstances. *American Sociological Review*, *60*(5), 655–673. https://doi.org/10.2307/2096316

Jones, B. L., & Nagin, D. S. (2013). A Note on a Stata Plugin for Estimating Group-based Trajectory Models. *Sociological Methods & Research*, *42*(4), 608–613. https://doi.org/10.1177/0049124113503141

Kleemans, E. R. (2014). Theoretical perspectives on organized crime. In L. Paoli (Ed.), *Oxford Handbook on Organized Crime* (pp. 32–52). Oxford: Oxford University Press.

Kleemans, E. R., & de Poot, C. J. (2008). Criminal Careers in Organized Crime and Social Opportunity Structure. *European Journal of Criminology*, *5*(1), 69–98.

Kleemans, E. R., & Van de Bunt, H. G. (1999). The social embeddedness of organized crime. *Transnational Organized Crime*, *5*(1), 19–36.

Kleemans, E. R., & Van de Bunt, H. G. (2008). Organised crime, occupations and opportunity. *Global Crime*, *9*(3), 185–197. https://doi.org/10.1080/17440570802254254

Kleemans, E. R., & van Koppen, V. M. (2014). Careers in organized crime. In D. Weisburd & G. Bruinsma (Eds.), *Encyclopedia of Criminology and Criminal Justice* (pp. 285–295). New York: Springer.

Kyvsgaard, B. (2002). *The Criminal Career: The Danish Longitudinal Study*. Cambridge University Press.

La Spina, A. (2014). The fight against the Italian mafia. In L. Paoli (Ed.), *The Oxford Handbook of Organized Crime* (pp. 593–611).

Liu, J., Francis, B., & Soothill, K. (2011). A Longitudinal Study of Escalation in Crime Seriousness. *Journal of Quantitative Criminology*, *27*(2), 175–196. https://doi.org/10.1007/s10940-010-9102-x

Massari, M. (2014). The Sacra Corona Unita: Origins, Characteristics, and Strategies. In *The 'Ndrangheta and Sacra Corona Unita* (pp. 101–116). Springer, Cham. https://doi.org/10.1007/978-3-319-04930-4_7

McGloin, J. M., Sullivan, C. J., Piquero, A. R., & Bacon, S. (2008). Investigating the Stability of Co-Offending and Co-Offenders Among a Sample of Youthful Offenders. *Criminology*, *46*(1), 155–188. https://doi.org/10.1111/j.1745-9125.2008.00105.x

Ministry of Justice - Department of Judicial Organisation. (2016). Monitoraggio della giustizia penale - Secondo trimestre 2016. Retrieved from https://www.giustizia.it/giustizia/it/mg_1_14_1.page?facetNode_1=0_17&contentId=SST1267241&previsiousPage=mg_1_14

Morselli, C. (2005). *Contacts, opportunities, and criminal enterprise*. Toronto: University of Toronto Press.

Nagin, D. S. (2016). Group-based Trajectory Modeling and Criminal Career Research. *Journal of Research in Crime and Delinquency*, *53*(3), 356–371. https://doi.org/10.1177/0022427815611710

Nagin, D. S., & Land, K. C. (1993). Age, Criminal Careers, and Population Heterogeneity: Specification and Estimation of a Nonparametric, Mixed Poisson Model*. *Criminology*, *31*(3), 327–362. https://doi.org/10.1111/j.1745-9125.1993.tb01133.x

Nagin, D. S., & Smith, D. A. (1990). Participation in and frequency of delinquent behavior: A test for structural differences. *Journal of Quantitative Criminology*, *6*(4), 335–356. https://doi.org/10.1007/BF01066675

Osgood, D. W., & Rowe, D. C. (1994). Bridging Criminal Careers, Theory, and Policy Through Latent Variable Models of Individual Offending*. *Criminology*, *32*(4), 517–554. https://doi.org/10.1111/j.1745-9125.1994.tb01164.x

Paternoster, R., & Triplett, R. (1988). Disaggregating Self-Reported Delinquency and Its Implications for Theory*. *Criminology*, *26*(4), 591–626. https://doi.org/10.1111/j.1745-9125.1988.tb00856.x

Peterson, M. A., Braiker, H. B., & Polich, S. M. (1980). *Who Commits Crimes: A Survey of Prison Inmates*. Oelgeschlager, Gunn & Hain.

Petras, H., Nieuwbeerta, P., & Piquero, A. R. (2010). Participation and Frequency During Criminal Careers Across the Life Span. *Criminology*, *48*(2), 607–637. https://doi.org/10.1111/j.1745-9125.2010.00197.x

Piquero, A. R., Brame, R., & Lynam, D. (2004). Studying Criminal Career Length Through Early Adulthood Among Serious Offenders. *Crime & Delinquency*, *50*(3), 412–435. https://doi.org/10.1177/0011128703260333

Piquero, A. R., Farrington, D. P., & Blumstein, A. (2003). The Criminal Career Paradigm. *Crime and Justice*, *30*, 359–506.

Piquero, A. R., Oster, R. P., Mazerolle, P., Brame, R., & Dean, C. W. (1999). Onset Age and Offense Specialization. *Journal of Research in Crime and Delinquency*, *36*(3), 275–299. https://doi.org/10.1177/0022427899036003002

Rhodes, W. (1989). The Criminal Career: Estimates of the Duration and Frequency of Crime Commission. *Journal of Quantitative Criminology*, *5*(1), 3–32.

Rojek, D. G., & Erickson, M. L. (1982). Delinquent Careers A Test of the Career Escalation Model. *Criminology*, *20*(1), 5–28. https://doi.org/10.1111/j.1745-9125.1982.tb00445.x

Rowe, D. C., Osgood, D. W., & Nicewander, W. A. (1990). A Latent Trait Approach to Unifying Criminal Careers *. *Criminology*, *28*(2), 237–270. https://doi.org/10.1111/j.1745-9125.1990.tb01325.x

Shelden, R. G., Horvath, J. A., & Tracy, S. (1989). Do Status Offenders Get Worse? Some Clarifications on the Question of Escalation. *Crime & Delinquency*, *35*(2), 202–216. https://doi.org/10.1177/0011128789035002002

Sherman, L. W., Schmidt, J. D., Rogan, D. P., Gartin, P. R., Cohn, E. G., Collins, D. J., & Bacich, A. R. (1991). From Initial Deterrence to Longterm Escalation: Short-Custody Arrest for Poverty Ghetto Domestic Violence. *Criminology*, *29*(4), 821–850. https://doi.org/10.1111/j.1745-9125.1991.tb01089.x

Soothill, K., Francis, B., Ackerley, E., & Humphreys, L. (2007). Changing Patterns of Offending Behaviour Among Young Adults. *British Journal of Criminology*, *48*(1), 75–95. https://doi.org/10.1093/bjc/azm039

Spelman, W. (1994). *Criminal Incapacitation*. Boston, MA: Springer. https://doi.org/10.1007/978-1-4757-4885-7

Sullivan, C. J., Mcgloin, J. M., Pratt, T. C., & Piquero, A. R. (2006). Rethinking the "norm" of offender generality: Investigating specialization in the short-term. *Criminology*, *44*(1), 199–233. https://doi.org/10.1111/j.1745-9125.2006.00047.x

Sullivan, C. J., McGloin, J. M., Ray, J. V., & Caudy, M. S. (2009). Detecting Specialization in Offending: Comparing Analytic Approaches. *Journal of Quantitative Criminology*, *25*(4), 419–441. https://doi.org/10.1007/s10940-009-9074-x

Tarling, R., Research, G. B. H. O., & Unit, P. (1993). *Analysing offending : data, models and interpretations*. London HMSO. Retrieved from http://trove.nla.gov.au/version/12981356

van Koppen, V. M., de Poot, C. J., Kleemans, E. R., & Nieuwbeerta, P. (2010). Criminal Trajectories in Organized Crime. *The British Journal of Criminology*, *50*(1), 102–123. https://doi.org/10.1093/bjc/azp067

van Onna, J. H. R., van der Geest, V. R., Huisman, W., & Denkers, A. J. M. (2014). Criminal Trajectories of White-collar Offenders. *Journal of Research in Crime and Delinquency*, *51*(6), 759–784. https://doi.org/10.1177/0022427814531489

Weisburd, D., & Waring, E. (2001). *White-Collar Crime and Criminal Careers*. Cambridge University Press.

Wolfgang, M. E., Figlio, R. M., & Sellin, J. T. (1972). *Delinquency in a Birth Cohort*. University of Chicago Press.

Wright, K. A., Pratt, T. C., & DeLisi, M. (2008). Examining Offending Specialization in a Sample of Male Multiple Homicide Offenders. *Homicide Studies*, *12*(4), 381–398. https://doi.org/10.1177/1088767908323930

ANNEX

Table of contents

INTRODUCTION 267

1 DATA TREATMENT 268

1.1 THE DAP DATASET 268
1.1.1 DATA CLEANING AND TRANSFORMATION OF THE DAP DATASET 268
1.1.2 RESHAPING OF THE DAP DATASET 275
1.1.3 DESCRIPTIVE STATISTICS – DAP DATASET 278
1.1.4 DESCRIPTIVE STATISTICS - MISSING VALUES IN THE DAP DATASET 286
1.2 THE CASELLARIO DATASET 286
1.2.1 RESHAPING OF THE CASELLARIO DATASET 286
1.2.2 CLASSIFICATION OF OFFENCES IN THE CASELLARIO DATASET 287
1.2.3 DESCRIPTIVE STATISTICS – CASELLARIO DATASET 290
1.2.4 DESCRIPTIVE STATISTICS – MISSING VALUES IN THE CASELLARIO DATASET 293

2 METHODOLOGY 294

2.1 COMPUTATION OF THE PARAMETERS OF CRIMINAL CAREER 294
2.1.1 SPECIALISATION 294
2.1.2 ESCALATION 295
2.2 PRINCIPAL COMPONENT ANALYSIS (PCA) 297
2.3 COMPARISON OF CRIMINAL CAREERS BEFORE AND AFTER RECRUITMENT 299
2.3.1 CLEANING PROCEDURE OF MAFIA MEMBERS' IDS 299
2.3.2 CREATION OF VARIABLE YEAR_RECRUITMENT 299

3 RESULTS: THE CRIMINAL CAREERS OF MAFIA MEMBERS 301

3.1 MACRO LEVEL: THE PARAMETERS OF THE CRIMINAL CAREERS OF MAFIA MEMBERS. 301
3.1.1 TRAJECTORIES IDENTIFIED THROUGH GROUP BASED TRAJECTORY MODELLING (GBTM) 301
3.1.2 COMPARISON OF CRIMINAL CAREERS BEFORE AND AFTER RECRUITMENT 303
3.2 MESO LEVEL: THE SIMILARITIES AND DIFFERENCES OF MAFIA MEMBERS ACROSS THE TYPES OF MAFIAS 304
3.2.1 TRAJECTORIES BY TYPE OF MAFIA IDENTIFIED THROUGH GROUP BASED TRAJECTORY MODELLING (GBTM) 304
3.3 MICRO LEVEL: EXPLORING THE DRIVERS OF THE RECRUITMENT INTO THE MAFIAS. 305

4 RESULTS: THE CRIMINAL CAREERS OF MAFIA BOSSES 308

4.1 MACRO LEVEL: THE PARAMETERS OF THE CRIMINAL CAREERS OF MAFIA BOSSES ... 308
4.1.1 TRAJECTORIES IDENTIFIED THROUGH GROUP BASED TRAJECTORY MODELLING (GBTM) 308
4.2 MESO LEVEL: SIMILARITIES AND DIFFERENCES OF MAFIA BOSSES ACROSS TYPES OF MAFIAS 310
4.2.1 TRAJECTORIES OF BOSSES BY TYPE OF MAFIA IDENTIFIED THROUGH GROUP BASED TRAJECTORY MODELLING (GBTM) 310

REFERENCES 312

Introduction

This document is an Annex to the report of Task 1.4. This Annex includes four sections providing:

- Section 1: information on data treatment and descriptive statistics on the two source datasets.
- Section 2: additional methodological information on the calculation of the parameters of the criminal careers, on how crime categories have been grouped to reduce their number and on the methodology followed to compare the criminal careers before and after recruitment.
- Section 3: additional results on the criminal careers of mafia members.
- Section 4: additional results on the criminal careers of mafia bosses.

1 Data treatment

This research project bases its analyses on the PROTON database, which originates from two distinct datasets provided by the Italian Ministry of Justice: the DAP dataset and the Casellario dataset. This section provides information on processes of data cleaning, data reshaping, and presents the descriptive statistics of both the DAP (Section 1.1) and the Casellario (Section 1.2) datasets.

1.1 The DAP dataset

1.1.1 DATA CLEANING AND TRANSFORMATION OF THE DAP DATASET

The research team identified different entries reporting missing values (e.g. "-", "Not available", "Not defined"). All these entries were harmonised by giving them a uniform value. Subsequently, computations were performed with regard to the ID. Individuals with multiple imprisonments kept the same ID, assigned at the time of their first imprisonment. Roughly 20% of individuals (n=3,315) were imprisoned multiple times (Table 1). In few cases (n=132) different IDs referred to the same mafia member, probably due to inaccuracies while assigning the ID after the first imprisonment. By crossing the DAP dataset with the *Casellario* dataset, the research team was able to detect and correct those IDs in the DAP dataset.[1]

Table 1. Number of imprisonments per number of individuals.

Imprisonments	N	%
1	14,076	80.94%
2	2,746	15.79%
3	481	2.77%
4	78	0.45%
5	10	0.06%
Total	17,391	100.00%

[1] The variable referring to the date of provisional release (DATE_PROV_RELEASE) was not considered for data cleaning and further analyses, i.e. discarded from the dataset, because 95% of its values were missing (n=20,305).

The following paragraphs describe in detail the data cleaning operations that were performed on each variable.

PR_AGGREGATED

The new variable PR_AGGREGATED combined PR_DOMICILE and PR_RESIDENCE, resulting in only 0.99% of missing values (n=211). The new aggregated variable contains either the values of PR_DOMICILE or the values of PR_RESIDENCE when PR_DOMICILE was missing. The research team started from PR_DOMICILE because it represents a more accurate description of the geographic location where mafia members were living at the time of imprisonment. With this imputation, PR_AGGREGATED resulted in a reduction of missing values from 52.39% to 0.99% (Figure 1).

COUNTRY_RESIDENCE_CLEAN

The new variable COUNTRY_RESIDENCE_CLEAN aimed at reducing the missing values for COUNTRY_RESIDENCE. The new variable was created combining the available values for country of residence of mafia members and imputing missing values based on the aggregated variable referring to the province of domicile and residence (PR_AGGREGATED). When a mafia member had a missing value for country of residence, but a value for PR_AGGREGATED was available, the research team assigned "ITALY" as the country of residence. This was possible because data on provinces was available only in regard to Italian places of residence and domicile. This imputation led to reduction of missing values from 8.21% to 0.74% (n=158) (Figure 1).

Figure 1. Number of missing values before and after data cleaning (%), N=21,373.

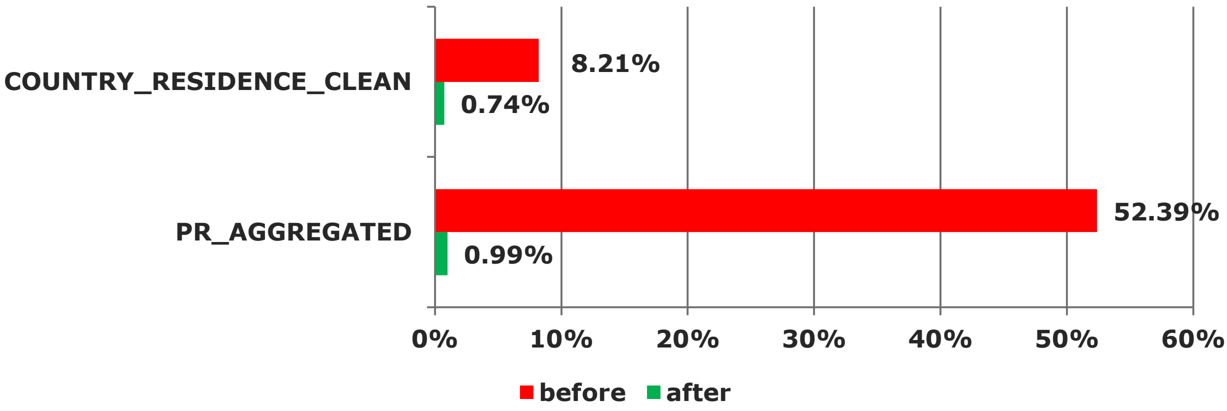

MAFIA_ASSOCIATION

The variable MAFIA_ASSOCIATION refers to the mafia group to which the mafia member was affiliated at the time of imprisonment. In some cases, the specificity of the values for MAFIA_ASSOCIATION varied for different times of

imprisonments. In all cases, the corresponding mafia groups pertained to the same geographic location. Therefore, only the most specific values were kept. For example, a value could show "OTHER MAFIAS APULIA" at the first time of imprisonment of an individual, but "SACRA CORONA UNITA" at the second time. In this case, researchers converted the former into the latter, resulting in a value of "SACRA CORONA UNITA" at both times of imprisonment, being "SACRA CORONA UNITA" the most prominent mafia group in Apulia. The research team followed the same approach when MAFIA_ASSOCIATION was missing (see examples in Table 2).

Table 2. Imputation of values for variable MAFIA_ASSOCIATION (example).

Before

ID	YEAR_BIRTH	MAFIA_ASSOCIATION
101	1958	OTHER MAFIAS APULIA
101	1958	SACRA CORONA UNITA

After

ID	YEAR_BIRTH	MAFIA_ASSOCIATION
101	1958	SACRA CORONA UNITA
101	1958	SACRA CORONA UNITA

Before

ID	YEAR_BIRTH	MAFIA_ASSOCIATION
158	1969	
158	1969	CAMORRA

After

ID	YEAR_BIRTH	MAFIA_ASSOCIATION
158	1969	CAMORRA
158	1969	CAMORRA

MAFIA_ASSOCIATION_CLEAN

The new variable MAFIA_ASSOCIATION_CLEAN, combining MAFIA_ASSOCIATION and PR_BIRTH, was created through the following imputation process aimed at reducing the missing values for MAFIA_ASSOCIATION. The research team first calculated the number of individuals (IDs) born in each Italian province (86 values of PR_BIRTH), then the relative frequencies of the mafia groups (values of MAFIA_ASSOCIATION) for those provinces with a number of IDs greater or equal than 100. Subsequently, if the relative frequency of a mafia group for each Italian province was greater or equal than 85%, than that value was imputed into a new variable named MAFIA_ASSOCIATION_CLEAN for all observations associated with that Italian province (Figure 2).[2] This imputation led to a reduction of missing values from 55.14% (n=9,589) to 30.89% (n=5,372).

Figure 2. Stages of the process leading to MAFIA_ASSOCIATION_CLEAN.

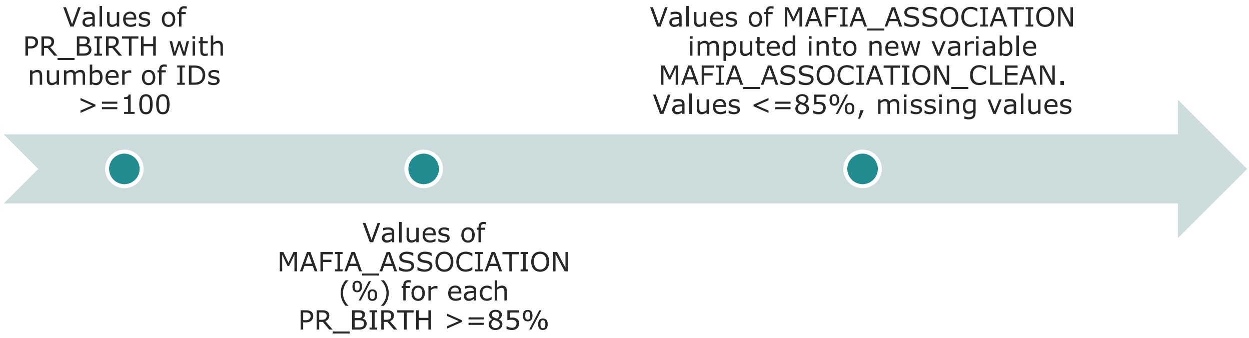

MAFIA_ASSOCIATION_AGGREGATED

The new variable MAFIA_ASSOCIATION_AGGREGATED was created grouping the different values for MAFIA_ASSOCIATION by Italian region. The aggregation produced five categories (values) for MAFIA_ASSOCIATION_AGGREGATED: "MAFIA_CALABRIA", "MAFIA_SICILY", "MAFIA_CAMPANIA", "MAFIA_APULIA", "MAFIA_LUCANA" (see Table 3).

Table 3. Reduction of categories for variable MAFIA_ASSOCIATION.

Before

**MAFIA_ASSOCIATION**
COSA NOSTRA, STIDDA, OTHER MAFIA SICILY
CAMORRA
NDRANGHETA, OTHER MAFIAS CALABRIA
SACRA CORONA UNITA, OTHER MAFIAS APULIA
OTHER MAFIAS LUCANA

After

**MAFIA_ASSOCIATION_AGGREGATED**
MAFIA_SICILY
MAFIA_CAMPANIA
MAFIA_CALABRIA
MAFIA_APULIA
MAFIA_LUCANA

[2] Values of MAFIA_ASSOCIATION_2 were considered as missing for Italian provinces with relative frequencies of mafia groups less than 85%.

MAFIA_ASSOCIATION_AGGREGATED_2

The new variable MAFIA_ASSOCIATION_AGGREGATED_CLEAN, combining MAFIA_ASSOCIATION_AGGREGATED and PR_BIRTH, was created through the following imputation process aimed at reducing the missing values for MAFIA_ASSOCIATION_AGGREGATED (same number of missing values as for MAFIA_ASSOCIATION). The research team first calculated the number of individuals (IDs) born in each Italian province (86 values of PR_BIRTH), then the relative frequencies of the mafia groups (values of MAFIA_ASSOCIATION_AGGREGATED) for those provinces with a number of IDs greater or equal than 100. Subsequently, if the relative frequency of a mafia group for each Italian province was greater or equal than 85%, than that value was imputed into a new variable named MAFIA_ASSOCIATION_AGGREGATED__CLEAN for all observations associated with that Italian province (Figure 3).[3] This imputation led to a reduction of missing values from 55.14% (n=9,589) to 20.96% (n=3,646).

Figure 3. Stages of the process leading to MAFIA_ASSOCIATION_ AGGREGATED_CLEAN.

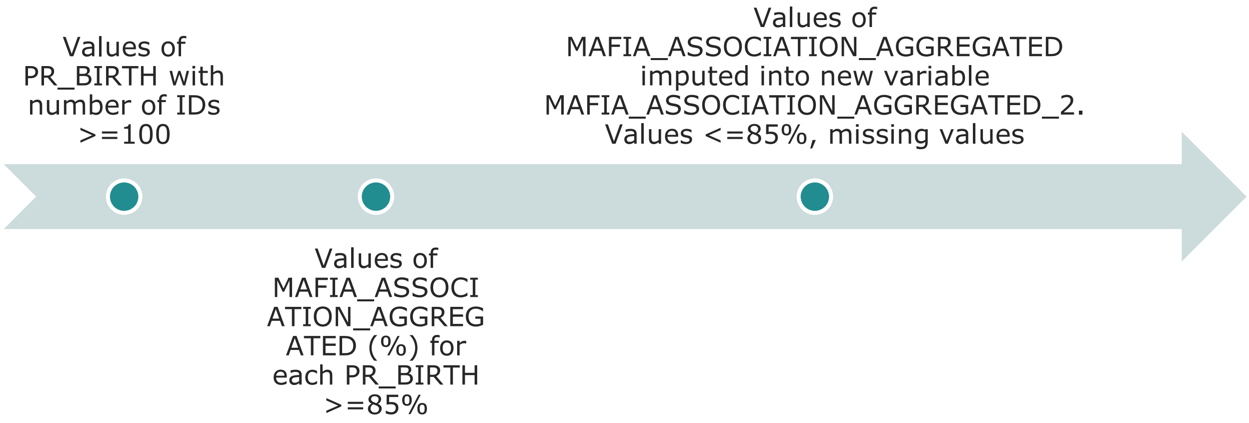

EDUCATION

The variable EDUCATION refers to the educational background. The research team aggregated different values referring to the same type of education. Thus, "UNIVERSITY DIPLOMA", "ACADEMIC DEGREE AND POST LAUREAM", and "BACHELOR DEGREE" were all values aggregated into a value labelled "ACADEMIC DEGREE" (Table 4). The imputation produced more consistent categories and reduced their number from twelve to seven: 1) "ILLITERATE", 2) "NO FORMAL EDUCATION, BUT LITERATE", 3) "ELEMENTARY SCHOOL", 4) "MIDDLE SCHOOL", 5) "PROFESSIONAL SCHOOL", 6) "HIGH SCHOOL", 7) "ACADEMIC DEGREE". Among the original categories, "OTHER" (n=41) was

---

[3] Values of MAFIA_ASSOCIATION_AGGREGATED_2 were considered as missing for Italian provinces with relative frequencies of mafia groups less than 85%.

considered as a missing value since it was not possible to infer the educational background from it.

Table 4. Reduction of categories for variable EDUCATION.

Before	After
OTHER	MIDDLE SCHOOL
ILLITERATE	ELEMENTARY SCHOOL
HIGH SCHOOL	HIGH SCHOOL
PROFESSIONAL SCHOOL	LITERATE, BUT NO FORMAL EDUCATION
UNIVERSITY DIPLOMA	PROFESSIONAL SCHOOL
BACHELOR DEGREE	ACADEMIC DEGREE
ACADEMIC DEGREE AND POST LAUREAM	ILLITERATE
ELEMENTARY SCHOOL	
MIDDLE SCHOOL	
HIGH SCHOOL	
LITERATE, BUT NO FORMAL EDUCATION	

PROFESSION

The variable PROFESSION refers to the profession of the mafia member. The team aggregated different professions which could be considered part of the same job category, following as guidelines the job categories provided by the Italian National Institute for Statistics (Istat).[4] Each category has subcategories which contain additional subcategories, allowing for an accurate identification of each job within its category. The research team chose to assign main categories to allow for a higher level of aggregation. Seven categories were coded: 1) "WORKMAN/HEAD-WORKMAN", 2) "DIRECTOR/MANAGER", 3) "EMPLOYEE", 4) "ENTREPRENEUR", 5) "SELF-EMPLOYED", 6) "FREELANCE PROFESSIONAL", 7) "OTHER". The latter category includes jobs that could not be unambiguously assigned to one of the other six categories listed above. To give an example, the categories listed in Table 5 were values of PROFESSION which were categorised into a single category.

[4] For further details, see http://www.istat.it/it/files/2013/07/la_classificazione_delle_professioni.pdf.

Table 5. Professional categories (example).

Subcategory	Main category
POWER PLANT WORKER	WORKMAN/HEAD-WORKMAN
APPRENTICE	
WORKMAN-HEADWORKMAN	
CARPENTER	
COACHBUILDER	
BLACKSMITH	
SURVEYOR	
LABORER	
MECHANIC	
MACHINIST	
OPERATOR OF AGRICULTURAL MACHINERY	
OPERATOR EARTH-REMOVING MACHINERY	
WELDER	
WOOD TURNING OPERATOR	

OCCUPATIONAL_STATUS

The variable OCCUPATIONAL_STATUS refers to the employment status of the mafia member at the time of imprisonment. Values referring to the same type of occupational status were aggregated (Table 6). As a result, twelve categories were converted into four: 1) "EMPLOYED", 2) "UNEMPLOYED", 3) "RETIRED", 4) "OTHER". The latter category includes individuals unfit for work, those doing military service, and mafia members whose occupational status was either "STUDENT" or "HOUSEWIFE".

Table 6. Categorisation of variable OCCUPATIONAL_STATUS.

Before	After
OTHER OCCUPATIONAL STATUS	EMPLOYED
HOUSEWIFE	UNEMPLOYED
UNEMPLOYED	RETIRED
JOB SEEKER	OTHER
FIRST TIME JOB SEEKER	
MILITARY SERVICE/CIVILIAN	
UNFIT FOR WORK	
OCCASIONAL EMPLOYEE	
EMPLOYED	
PENSIONER	
RETIRED	
STUDENT	

ADDITIONAL IMPUTATIONS ON THE VARIABLES

Two additional data cleaning steps were taken. Imputations of missing values for observations regarding the same individuals at multiple times of imprisonment were performed for the following variables: COUNTRY_BIRTH, PR_BIRTH, COUNTRY_BIRTH_CLEAN, PR_AGGREGATED, MARIAL_STATUS, RELIGION, EDUCATION, SECTOR_EMPLOYMENT, MAFIA_ASSOCIATION and ROLE. When values for the above-mentioned variables were missing for some imprisonments, but were available for others, the missing values were imputed.

These variables too were presenting diverging values for multiple imprisonments. For example, a mafia member may have been recorded as "ILLITERATE" at the time of his/her first imprisonment, while the value associated with the second imprisonment may have been "ELEMENTARY SCHOOL". In case of conflicting values within the same ID, the research team opted for the most recent values, except in the case of EDUCATION and ROLE. For the latter two variables, "higher" values were kept, where "higher" refers to higher education and higher role (e.g., in order of importance: "BOSS", "UNDERBOSS/LIEUTENANT", "ASSOCIATE", "KILLER").

After completing the data transformation and cleaning, the research team proceeded to reshaping the DAP dataset.

1.1.2 RESHAPING OF THE DAP DATASET

The DAP dataset includes 21,373 observations referring to 17,391 individuals who entered the penitentiary for the mafia association crime. The number of observations exceeds the number of unique individuals because some mafia members entered the penitentiary more than once due to a mafia association conviction or convictions for other offences.

For the purpose of the analysis, it is convenient to work with a database where each observation (i.e. row) identifies a unique individual. This shape of the dataset allows to more easily derive aggregate statistics on the whole population. As a consequence, the research team converted the DAP dataset from long form (where each observation identifies a different entrance in the penitentiary) to wide form (where each observation refers to a different individual). In the wide form dataset, there are two types of variables:

1. Variables uniquely identified for each individual and that do not change across imprisonments, such as:

a. Year of birth, sex, country of birth, province of birth, region of birth, country of residence and aggregated province: these variables were uniquely identified for each individual in the DAP dataset;

b. Religion, education, marital status, sector of employment, mafia association and role: these variables were not uniquely identified for each individual in the original DAP dataset, since for some individuals they changed across different imprisonments. However, the data cleaning process allowed to derive a unique value of such variables for each individual.

2. Variables uniquely identified for each imprisonment but not for each individual, such as: date of arrest, date of provisional release, date of definitive release, date of release, detention status, occupational status and profession. For each of these variables, the wide shape of the dataset reports five different columns identifying the value of the variable at the first, second, third, fourth and fifth imprisonment (see example in Table 7).

Table 7. Reshaping of the DAP dataset.

ID	SEX	EDUCATION	DATE_ARREST	DATE_RELEASE
155	M	ILLITERATE	01/01/1990	07/11/1998
155	M	ILLITERATE	14/05/2005	05/10/2023
203	M	MIDDLE SCHOOL	02/06/1988	20/04/2001

ID	SEX	EDUCATION	DATE_ARREST1	DATE_RELEASE1	DATE_ARREST2	DATE_RELEASE2
155	M	ILLITERATE	01/01/1990	07/11/1998	14/05/2005	05/10/2023
203	M	MIDDLE SCHOOL	02/06/1988	20/04/2001		

The reshaping of the DAP dataset led to a new dataset containing 75 variables, of which 60 are followed by a number representing the number of imprisonment (e.g. DATE_ARREST1) (Table 8). Of these 60 variables, 20 are new variables created by the research team for analyses purposes whose values are calculated as follows:

- AGE_ARREST1: difference between DATE_ARREST1 and YEAR_BIRTH;
- DAYS_IMPRISONMENT1: difference between DATE_END_SENTENCE1 and DATE_ARREST1;
- MONTHS_IMPRISONMENT1: derived from DAY_IMPRISONMENT1.
- YEARS_IMPRISONMENT1: derived from MONTHS_IMPRISONMENT1.

Table 8. DAP dataset - After reshaping (N=17,391).

**Variable name**
ID
YEAR_BIRTH
SEX
COUNTRY_BIRTH
PR_BIRTH
REGION_BIRTH
COUNTRY_RESIDENCE_CLEAN
PR_ AGGREGATED
REGION_AGGREGATED
MARITAL_STATUS
RELIGION
SECTOR_EMPLOYMENT
MAFIA_ASSOCIATION
MAFIA_ASSOCIATION_CLEAN
MAFIA_ASSOCIATION_AGGREGATED
MAFIA_ASSOCIATION_AGGREGATED_CLEAN
EDUCATION
ROLE
REGISTRATION_NUMBER1
DETENTION_STATUS1
OCCUPATIONAL_STATUS1
PROFESSION1
DATE_ARREST1
AGE_ARREST1
DATE_DEF_RELEASE1
DATE_RELEASE1
DATE_END_SENTENCE1
DAYS_IMPRISONMENT1
MONTHS_IMPRISONMENT1
YEARS_IMPRISONMENT1

Note: Variables in grey refer to the number of imprisonment and are repeated for each imprisonment (maximum five times).

The reshaping of the DAP dataset enabled calculation of univariate and bivariate descriptive statistics on the variables included in Table 8. For an overview of these statistics, see below.

1.1.3 DESCRIPTIVE STATISTICS – DAP DATASET

This section presents some descriptive statistics for the main variables of the DAP dataset. Percentages of mafia members are shown by year of birth (Figure 4) , by sex (Figure 5), by country (Figure 6) and province of birth (Figure 7), province of domicile (Figure 8), by economic sector of employment (Figure 11), by affiliation (Figure 12), by educational background (Figure 13), by role (Figure 14), by detention status at first arrest (Figure 15), and by number of years in prison after first imprisonment (Figure 17).

Percentages of mafia members by region of birth are also presented (Figure 9), together with those by region of domicile (Figure 10) and the age at first arrest (Figure 16). Other percentages include the role of mafia members by mafia type (Figure 18) and by sex (Figure 19 and Figure 20). Education by role (Figure 21), professional status by profession at first arrest (Figure 22), and profession at first arrest by role (Figure 23) percentages are also given.

Figure 4. Mafia members by year of birth (%), N=17,391.

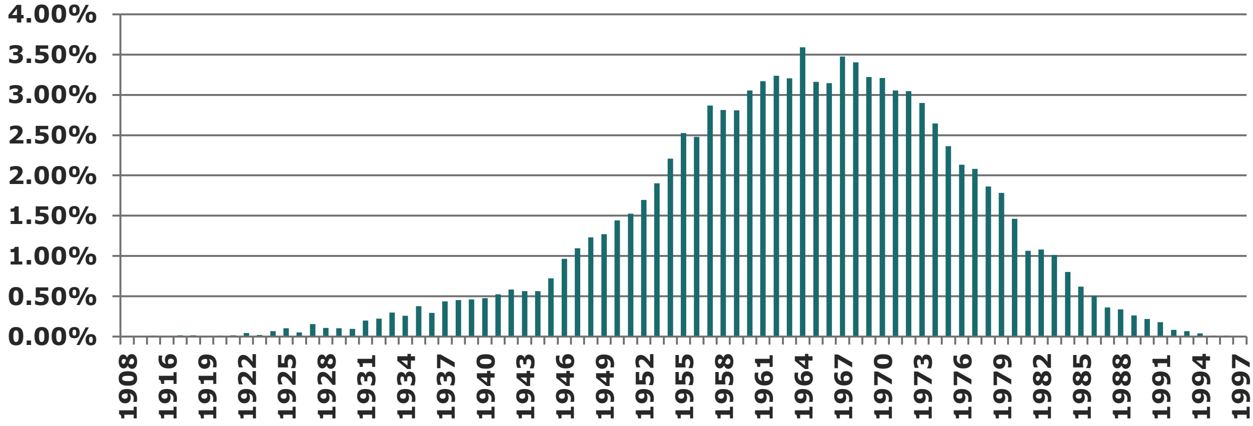

Figure 5. Mafia members by sex (%), N=17,391.

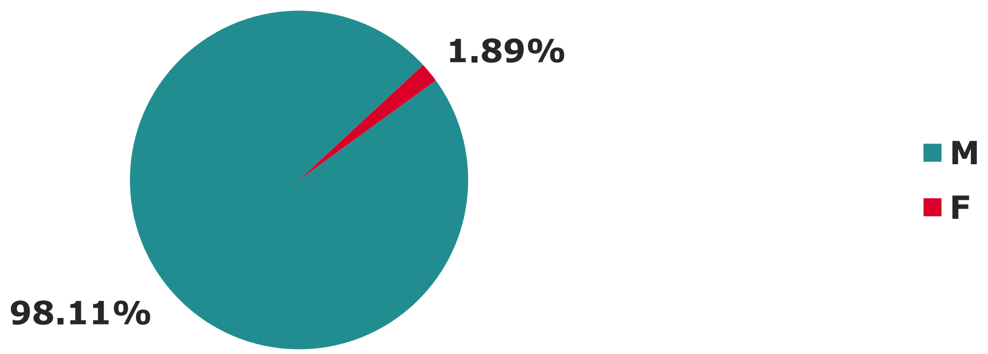

Figure 6. Mafia members by country of birth (%), n=17,388.

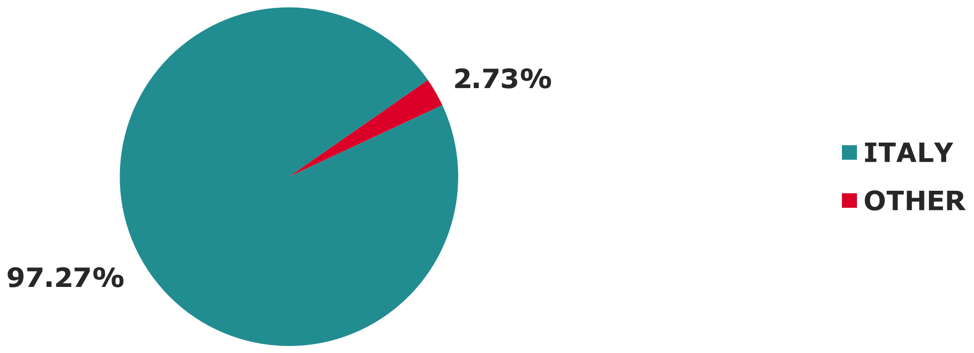

Figure 7. Mafia members by province of birth (%), n=16,913.

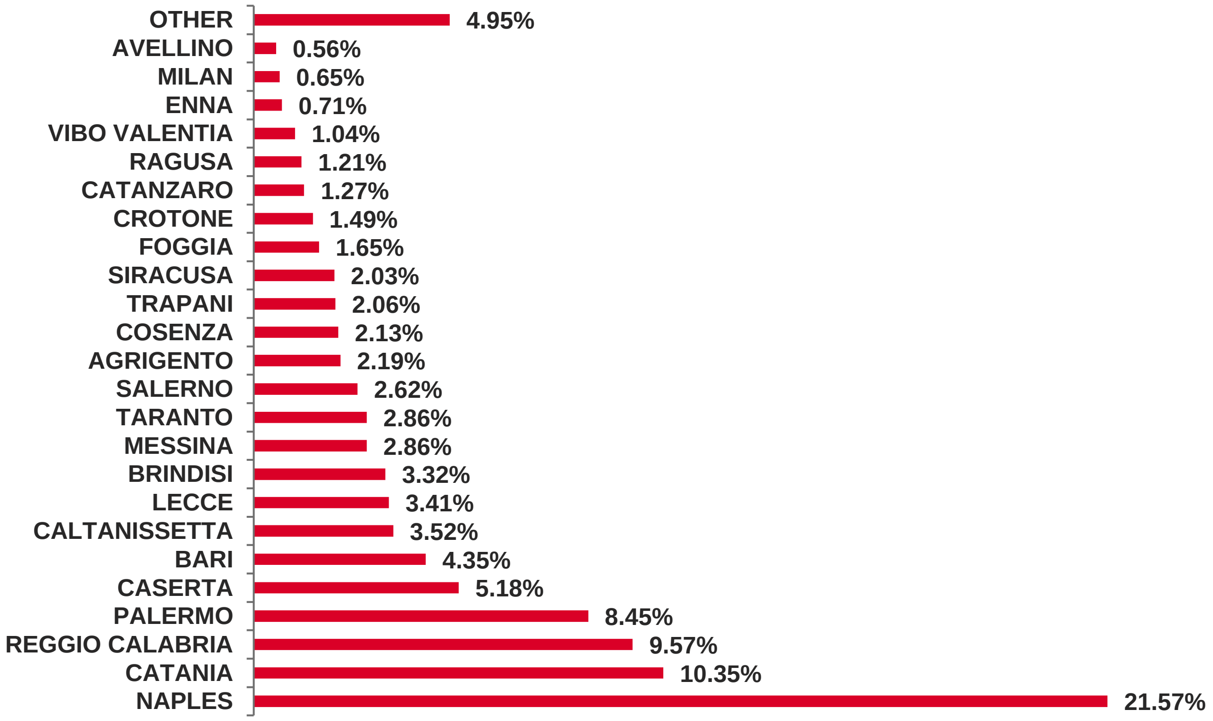

Figure 8. Mafia members by province of domicile (%), n=17,222.

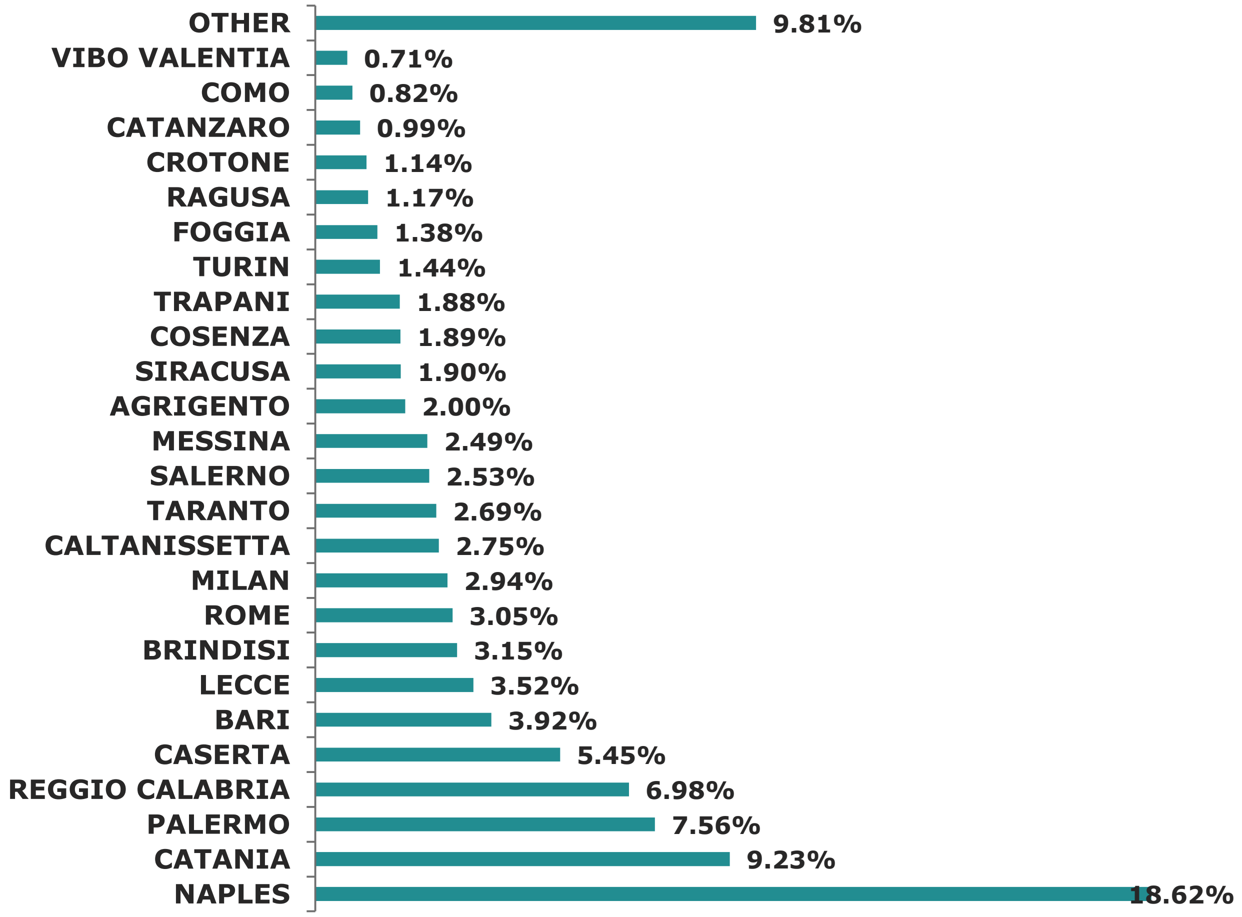

Figure 9. Region of birth of Mafia members (%), n=16,913.

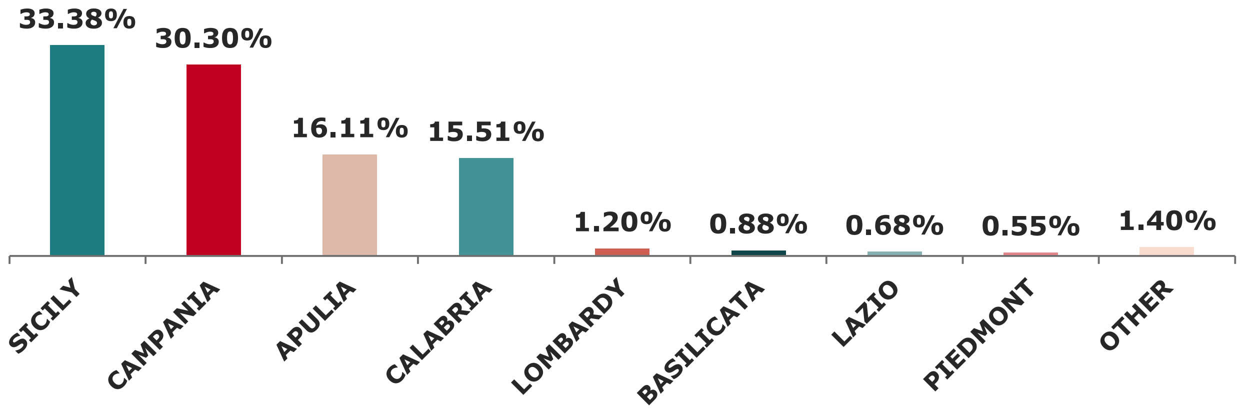

Figure 10. Region of domicile (or residence when domicile is missing) of Mafia members (%), n=17,222.

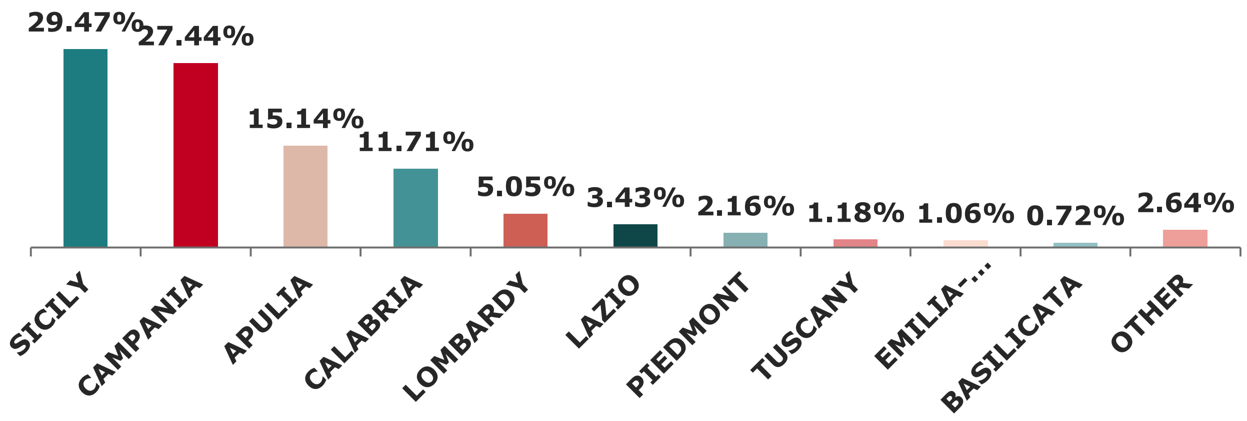

Figure 11. Mafia members by economic sector of employment (%), n=6,925.

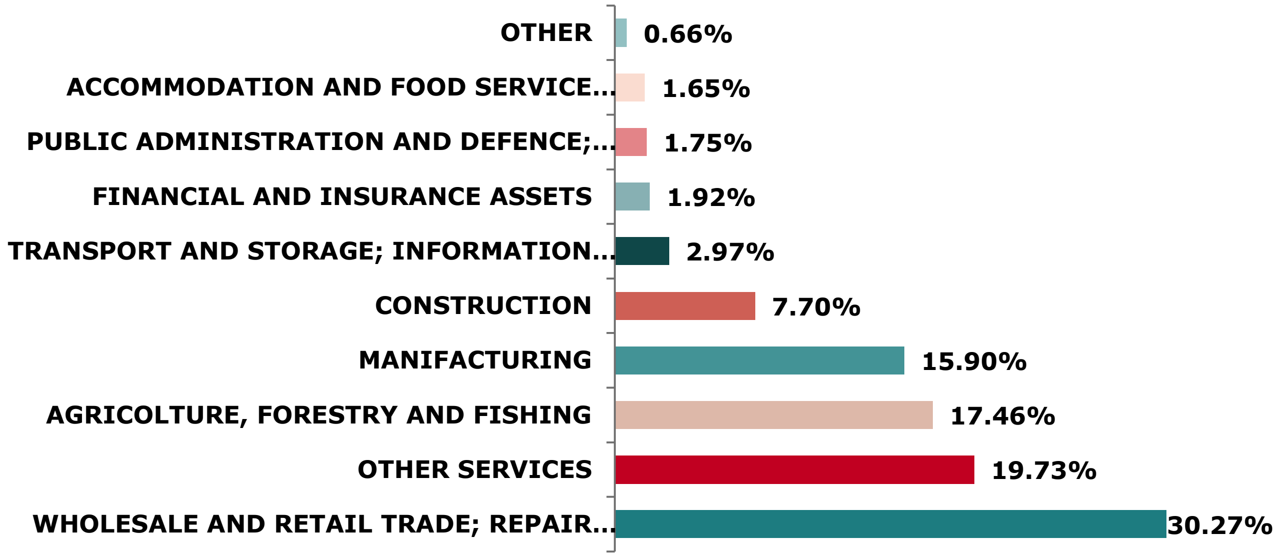

Figure 12. Mafia members by affiliation (%), n=9,141.

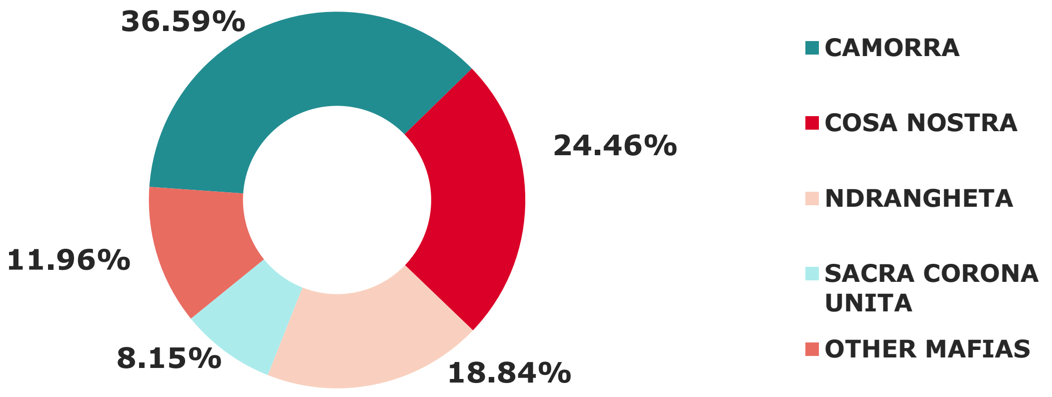

Figure 13. Mafia members by educational background (%), n=13,882.

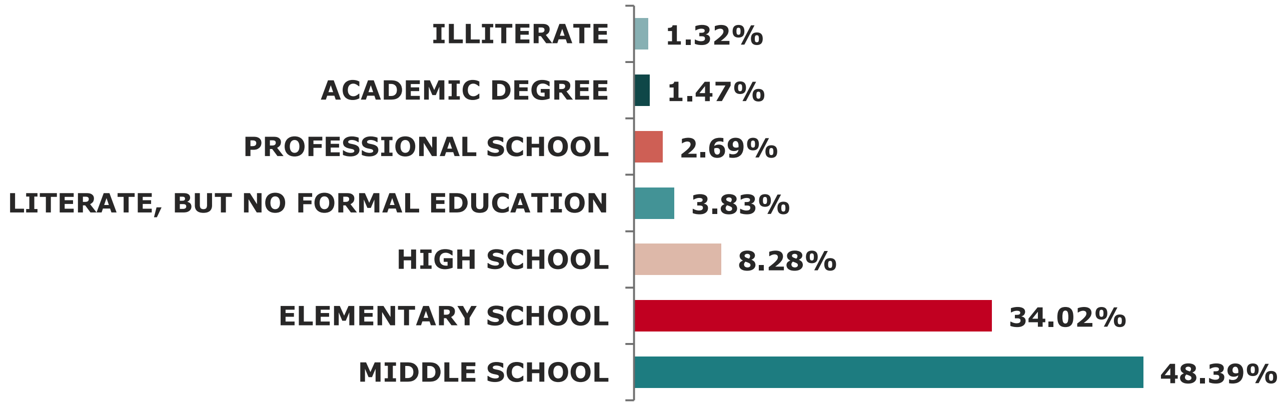

Figure 14. Mafia members by role (%), n=7,058.

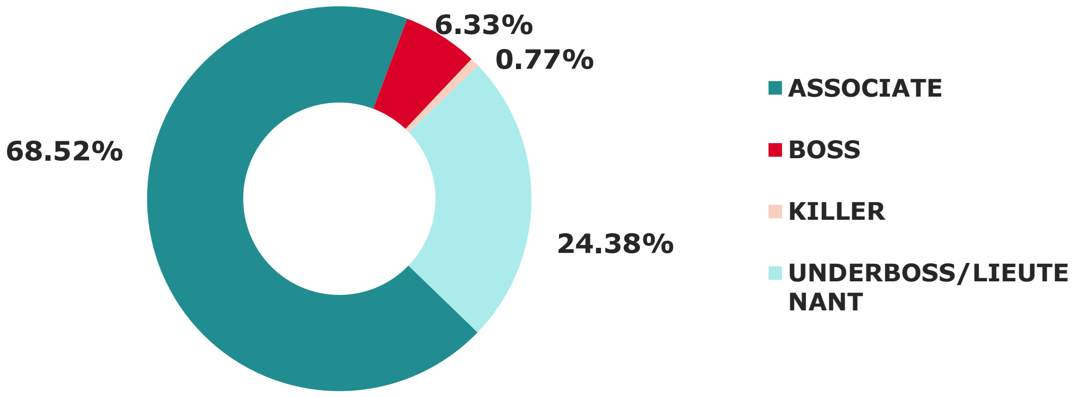

Figure 15. Mafia members by detention status (first arrest) (%), N=17,391.

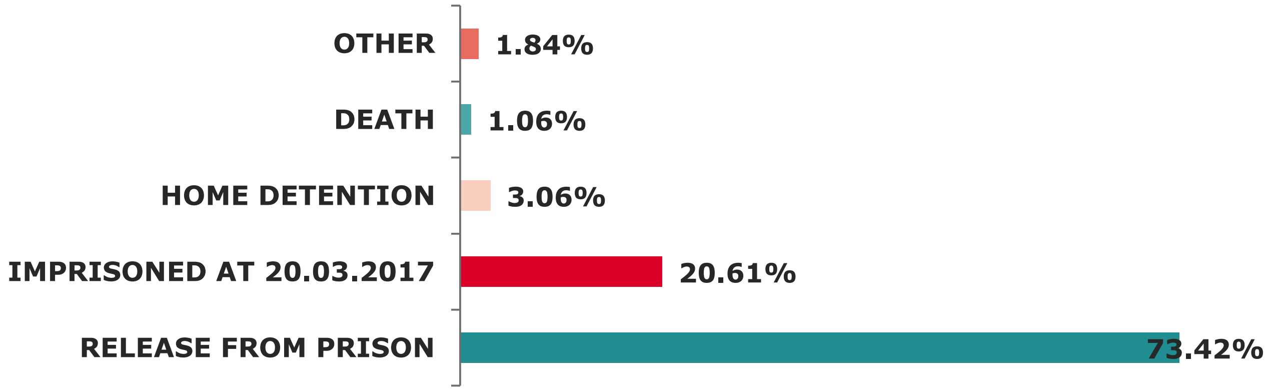

Figure 16. Age of Mafia members at first arrest (%), N=17,931.

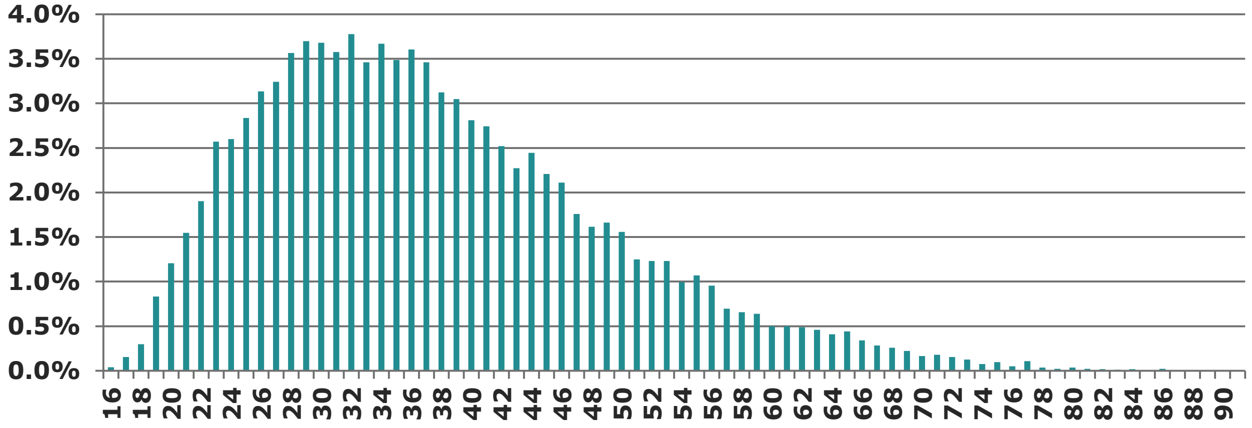

Figure 17. Mafia members by number of years in prison after first imprisonment (%), n=17,390.

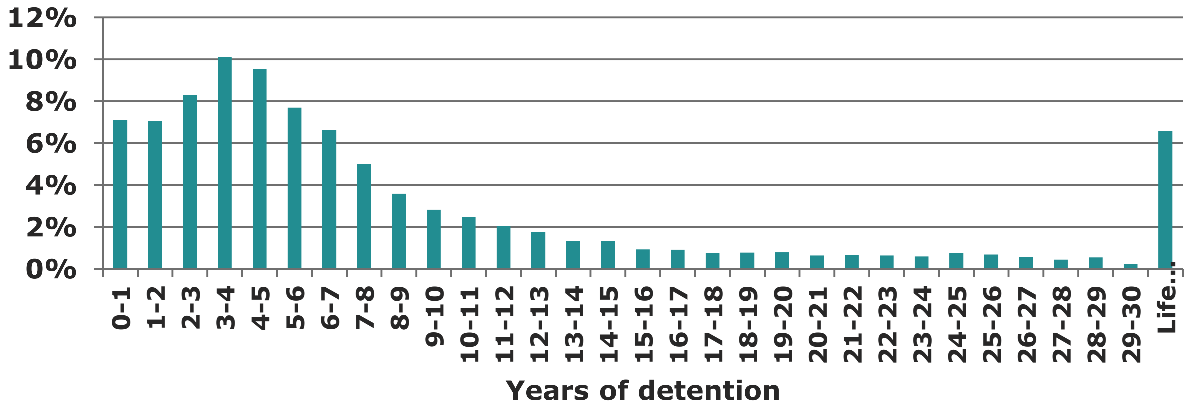

Figure 18. Role of mafia members by mafia type (%), n=6,961.

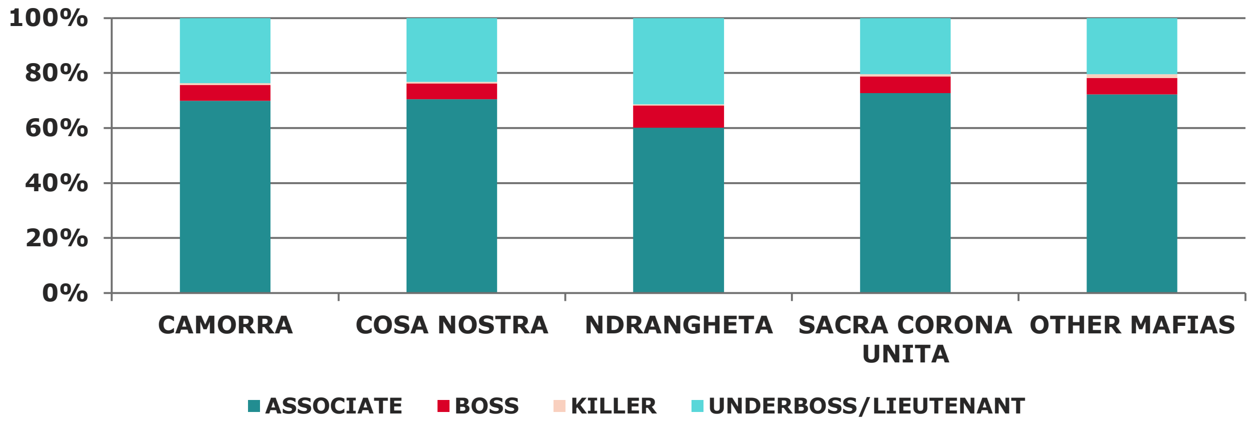

Figure 19. Role of mafia members per sex - Men (%), n=6,920.

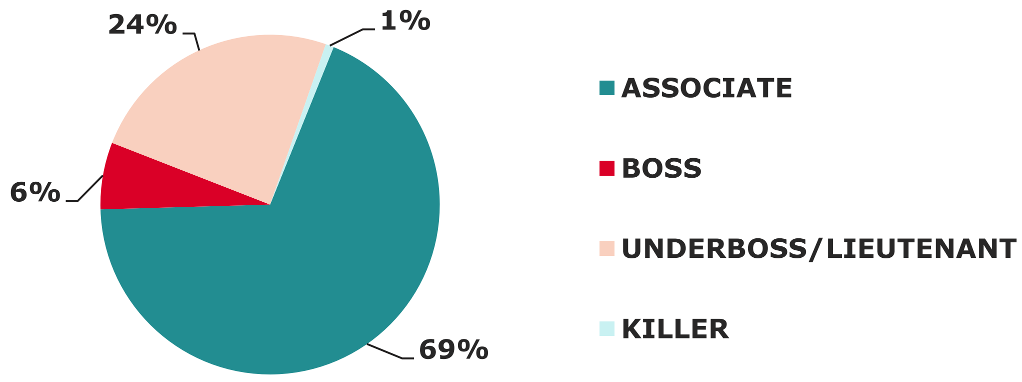

Figure 20. Role of mafia member per sex - Women (%), n=131.

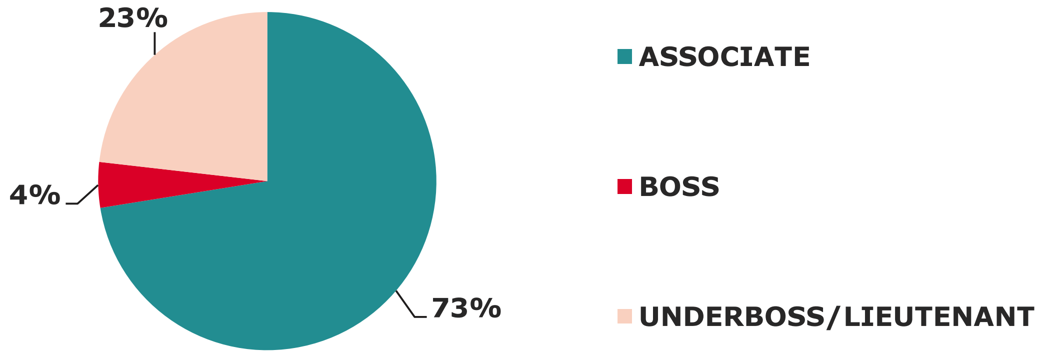

Figure 21. Education of convicted mafia members by role (%), n=5,448.

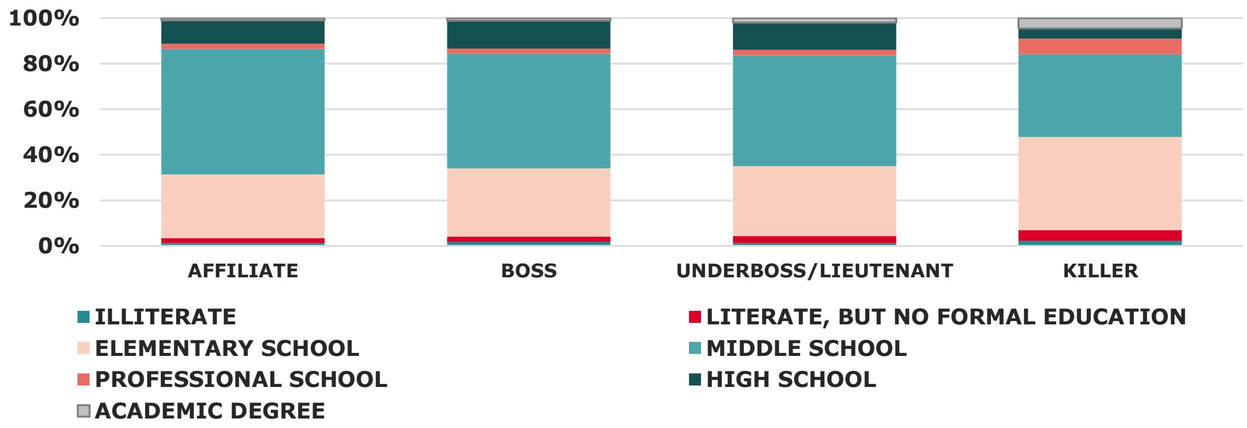

Figure 22. Professional status of convicted Mafia members by profession at first arrest (%), n=6,583.

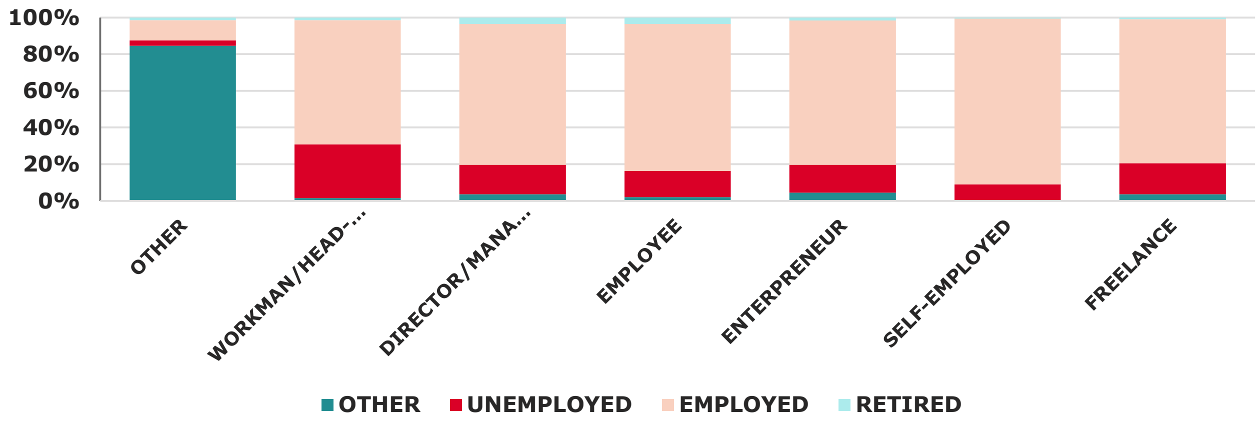

Figure 23. Profession of mafia members at first arrest by role (%), n=3,429.

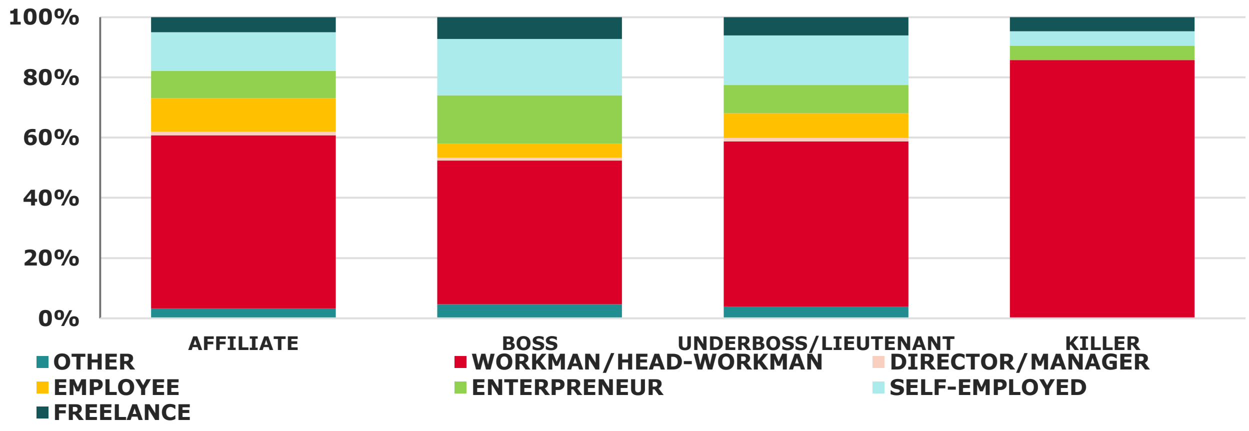

1.1.4 DESCRIPTIVE STATISTICS - MISSING VALUES IN THE DAP DATASET

The missing values are presented for the main variables of the DAP dataset (Figure 24). Missing values for mafia association at first arrest are also presented (Figure 25).

Figure 24. Missing values per variable (%), N=17,391.

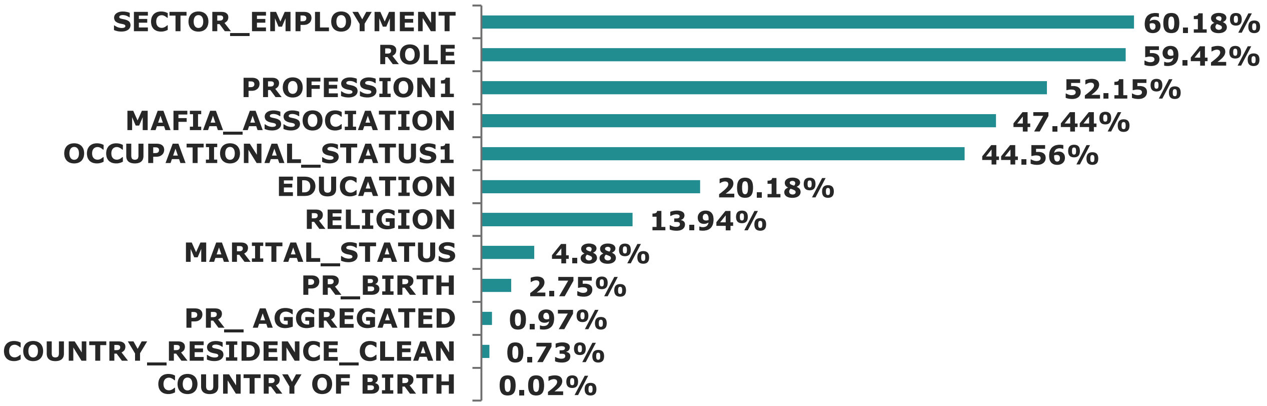

Figure 25. Missing values for mafia association of mafia members at first arrest (%), N=17,391.

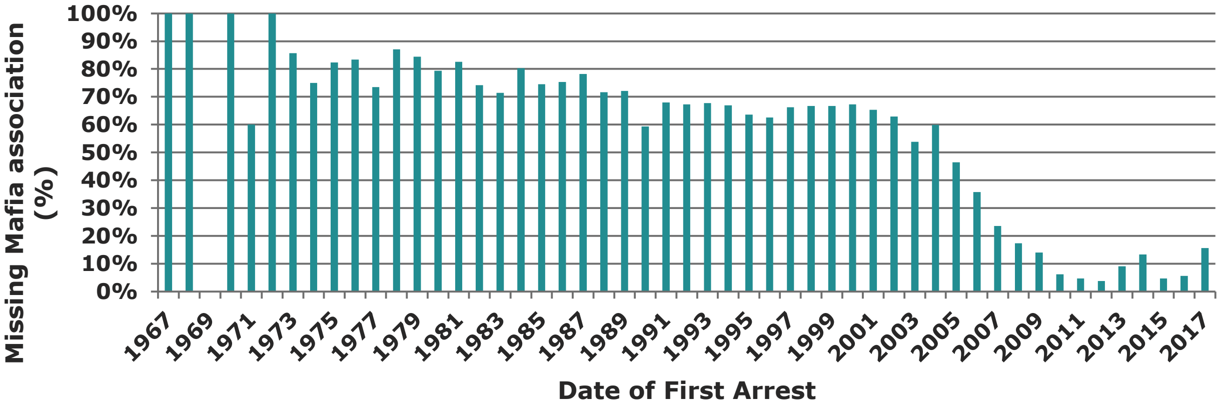

1.2 The Casellario dataset

1.2.1 RESHAPING OF THE CASELLARIO DATASET

The *Casellario* dataset provides information on all the criminal convictions of each individual in the DAP dataset. Unique numeric IDs (N=13,229) enabled to link the *Casellario* dataset to the DAP dataset.

The *Casellario* dataset consisted of two sub-datasets:

- Offences (*Reati*)
- Judgements (Provvedimenti)

The Judgements dataset provided specific information on the judicial authorities that issued the convictions for each offence included in the Offences dataset (Table 9). The PROG_PROV variable was present also in the Judgements dataset and enabled linking the two datasets.

Table 9. Description of variables of the Judgements dataset

**Original name**	**Variable Explanation**
PROG_PROV (N=102,346)	Code assigned to each criminal judgement
ID_SUB (N=13,229)	Identification number for each individual included in the dataset
YEAR_PROV	Year of the judgement
JUD_AUTHORITY	Judicial authority which issued the judgement
PROVINCE_JA	Italian province of the judicial authority

IDs retrieved from the Judgements dataset were included in the Offences dataset. The dataset was subsequently reshaped using the single ID_SUB variable as unit of observation. As a result, each row of the reshaped Offences dataset contained the set of criminal records (and related variables) of a single individual (Table 10).

Table 10. Reshaping of the Offences dataset.

PROG_CRIME	ID_SUB
1	30
2	30
3	50
4	50
5	70

ID_SUB	PROG_CRIME_1	PROG_CRIME_2
30	1	2
50	3	4
70	5	

1.2.2 Classification of offences in the Casellario dataset

The dataset included thousands of different offences, as the Italian Criminal Records Registry provides detailed information on the legislative source, article number, and even paragraph of the violated provisions. While most offences were in the Italian Criminal Code, many others were in special laws. To synthetize the considerable amount of distinct offences committed by the mafia members, the research team classified them into crime categories (Table 11).

Table 11. Number of offences per legislative source in the offences dataset

Legislative source	N	%
Italian Criminal Code	117,692	57.08%
Italian Special Laws	66,608	32.30%
Illegal Drugs Act	12,318	5.97%
Law against bad cheques	5,073	2.46%
New Road Traffic Code	1,510	0.73%
Abrogated Road Traffic Code	1,082	0.52%
Military Criminal Code (in peace time)	974	0.47%
Law on Bankruptcy	429	0.21%
Law against Prostitution	281	0.14%
Naval Code	189	0.09%
Foreign Criminal Codes	29	0.01%
Civil Code	12	0.01%
Military Criminal Code (in war time)	1	0.00%

The classification of offences in the Criminal Code used the Article number, the section, the title, and the book of the Code.[5] As a result, some categories comprised all offences in the same Code title (Table 12).

Table 12. Example of the classification of offences in the Italian Criminal Code

**Article number**	**Topic**	**Book**	**Title**	**Code**	**Category**
368	Slander	2	3	368.2.3	Offences against the criminal justice system

The classification of offences in special laws used the law number and the year, the article and the paragraph of each specific law (Table 13). Most offences in special laws were classified at the paragraph level of detail, as different paragraphs of an article may often include distinct criminal offences.[6] The classification regrouped the offences into similar categories, based on the purpose of special laws and the type of criminal offence (i.e.: felony or misdemeanour).[7]

---

[5] the Italian Criminal Code is organised in three books, titles, and sections. Each title includes a specific class of offences (e.g.: Book II, Title III is on Offences against the criminal justice system).

[6] The research team classified the offences at the paragraph level for 90% of the offences in special laws. The residual 10% included very rare offences which were classified at only at the article level.

[7] The Italian criminal law distinguishes offences between felonies (*delitti)* and misdemeanours *(contravvenzioni)*. Felonies are more serious offences and are punished either by life sentence *(ergastolo)*, imprisonment *(reclusion)* or fine (*multa*). Misdemeanors are less serious offenses and are punished by either detention *(arresto)* or pecuniary penalty (*ammenda*).

Table 13. Example of the classification of offences in special laws.

Law number	Law year	Article number	Topic	paragraph	Code	Category
110	1975	23	Clandestine Weapons	1	110-1975-23-1	Weapons and explosives - felonies
110	1975	4	Use of bats or sticks in public spaces	1	110-1975-4-1	Weapons and explosives - weapons

After the classification of offences in the Criminal Code and special offences, a few relevant and frequent offences were assigned a separate category (e.g. extortion, thefts, mafia-association, and illicit drug production, trafficking and selling). The final classification distributed all 206,198 offences into 31 categories (Table 14).

Table 14. Offences per crime category (N=206,198).

Crime Category	N	%
Weapons and explosives - Felonies	33,139	16.07%
Extortion	17,074	8.28%
Thefts	14,421	6.99%
Mafia association	13,378	6.49%
Illicit Drugs production. trafficking and selling	12,489	6.06%
Fencing	11,219	5.44%
Murder	10,810	5.24%
Other Special Laws	9,625	4.67%
Robbery	8,575	4.16%
Other - Misdemeanours	7,338	3.56%
Offences against the criminal justice system - Felonies	6,941	3.37%
Weapons and explosives - Misdemeanours	6,018	2.92%
Financial Offences	5,474	2.65%
Offences against public officers	5,328	2.58%
Road traffic misdemeanours	4,917	2.38%
Assault and violent offences	4,860	2.36%
Other - Felonies	4,566	2.21%
Threats and intimidation	3,899	1.89%
False documentation and forgery	3,872	1.88%
Arson and damages	3,631	1.76%
Drug Trafficking criminal association	3,584	1.74%
Evasion	2,908	1.41%
Smuggling - Felonies/Misdemeanours	2,278	1.10%
Administrative Violations	2,116	1.03%
Criminal association	1,757	0.85%
Fraud	1,195	0.58%
Offences against the criminal justice system - Misdemeanours	1,147	0.56%
Loan Sharking and Money Laundering	1,012	0.49%
Military Service	974	0.47%
OTHER	940	0.46%
Corruption	713	0.35%

1.2.3 Descriptive Statistics – Casellario dataset

The number of offences committed by mafia members are reported per year (Figure 26), per crime category (Figure 27), per legislative source (Figure 28), and per Italian province (Figure 29). Missing values are also shown

Figure 26. Offences committed by mafia members per year (%), N=179,907.

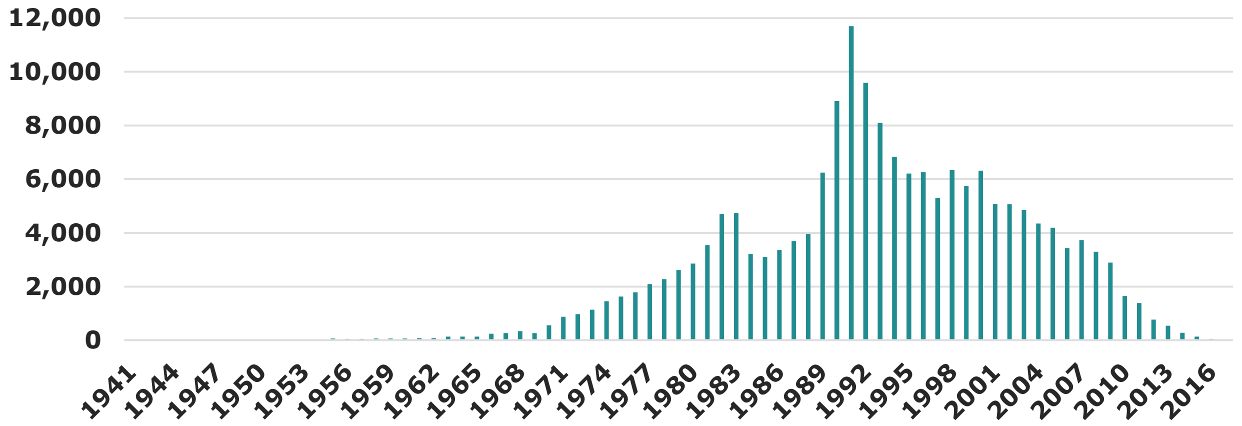

Figure 27. Number of offences committed by mafia members per crime category (%), N=206,198.

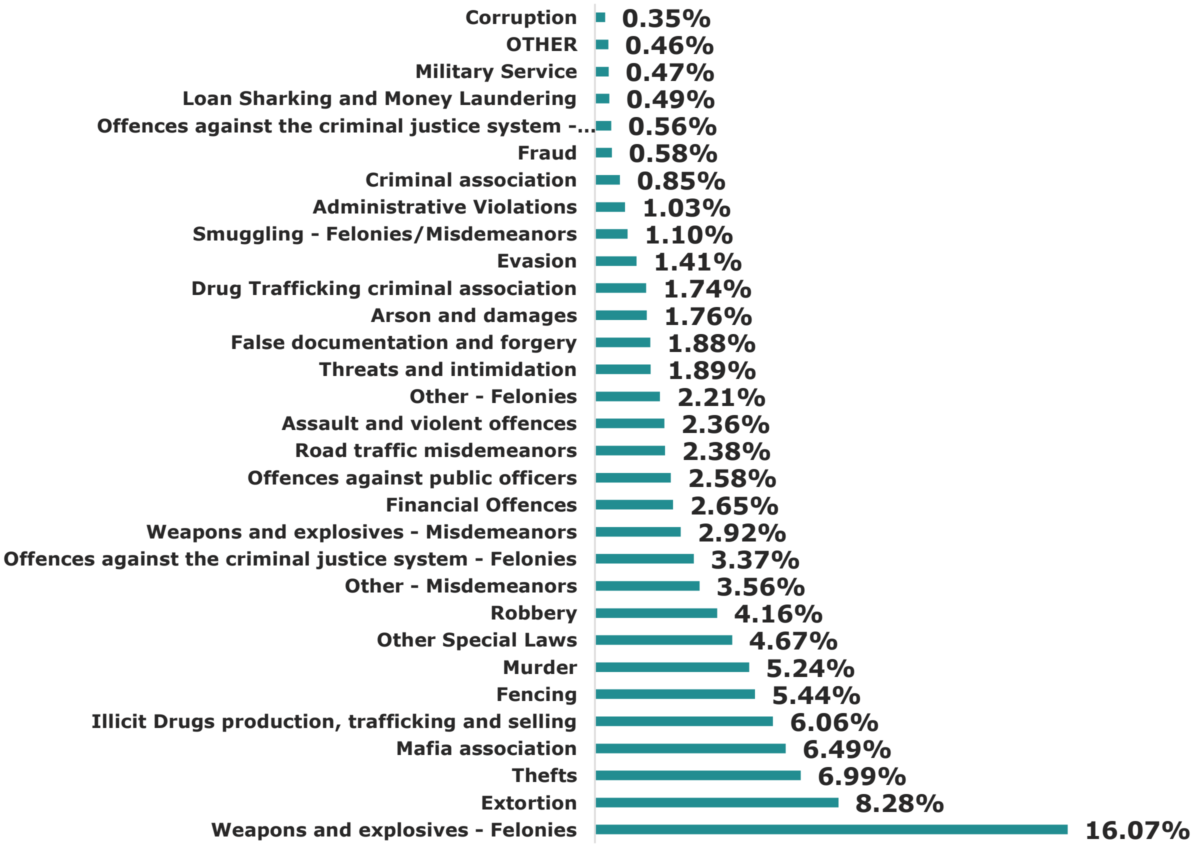

Figure 28. Number of offences committed by mafia members per Legislative source (%), N=206,198

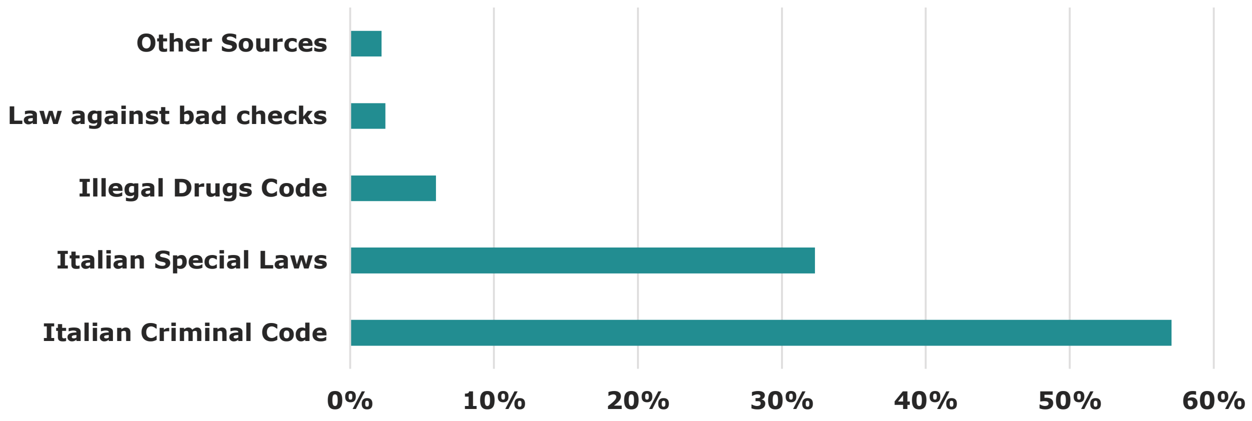

Figure 29. Number of offences committed by Mafia members per Italian provinces (%), N=102,813.

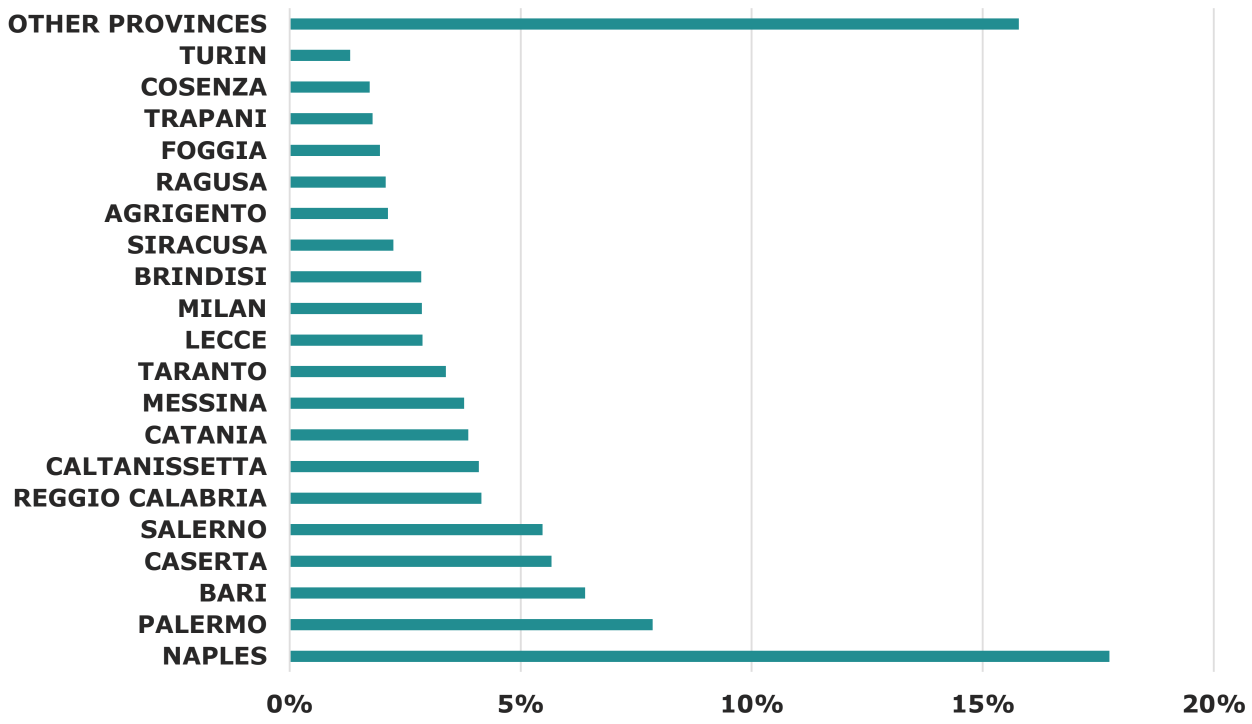

1.2.4 Descriptive statistics – Missing values in the Casellario dataset

The missing values are presented here for the only variables of the *Casellario* dataset for which complete information is not available: crime_year and crime_province (Figure 30).

Figure 30. Missing values per variable (%), N=206,198

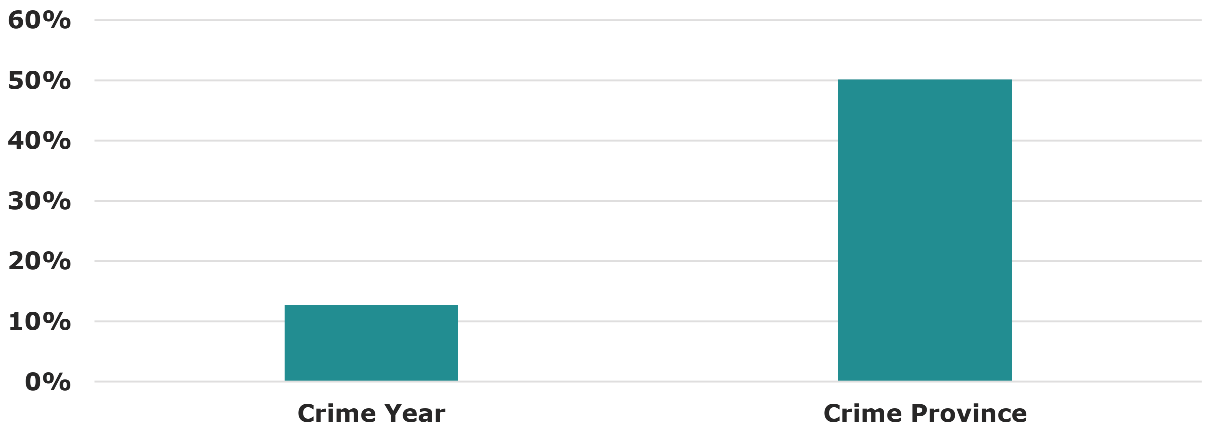

2 Methodology

This research project applies the criminal careers framework to the Italian mafia members. This section focuses on the different methodological strategies adopted across the project. The research procedures applied to the computation of the two parameters "specialisation" and "escalation" are presented in Section 2.1. The propensity of mafia members to commit certain types of crimes application has been studied by performing Principal Component Analysis (PCA), and the research approach is described in Section 2.2. The cleaning procedure to explore the differences in the criminal career of mafia members before and after the recruitment into mafias, together with the creation of the new variable year_recruitment, are presented in Section 3.3.

2.1 Computation of the parameters of criminal career

2.1.1 Specialisation

Investigations on specialisation have mainly used four strategies to model offending specialisation (see Sullivan, McGloin, Ray, & Caudy, 2009):

1. The Diversity Index
2. The forward specialisation coefficient (henceforth FSC)
3. Latent class analysis
4. A multilevel latent variable approach

The present study chose the Diversity Index. In line with previous studies, the diversity index is computed only for mafia members who committed at least two offences during their criminal career (Mazerolle, Brame, Paternoster, Piquero, & Dean, 2000; Piquero et al., 1999; Sullivan et al., 2006). Given the number of crime categories identified in the dataset, the maximum value of the diversity index in this analysis is (31-1)/31=0.97.

Methods 2, 3, and 4 were discarded for different reasons. The FSC, firstly proposed by Farrington et al. (1988), allows only to derive a sample-level aggregate measure of specialisation. The latent class analysis, although it examines the nature of specialisation besides its degree, requires a high degree of subjective judgement in setting the model parameters (Sullivan et al., 2009). The multilevel latent variable approach also provides guidance on the kind of specialisation, but it identifies only whether an offender is specialized on a specific type of crime (e.g. drug offences) that needs to be pre-imposed by the researcher.

2.1.2 Escalation

The literature on escalation revealed three main approaches to assess the escalation in crime seriousness (see Liu et al., 2011):

1. Regression approach
2. Crime-Type Switching Tables
3. Analysis of means

The present study relies on the regression approach to estimate the escalation. This section explains thoroughly the reason for this methodological choice, in particular with regard to the two discarded approaches (i.e. Crime-Type Switching Tables and Analysis of means).

Crime-type switching tables categorize convictions into offending groups with distinct crime severity. To evaluate whether there is evidence of the matrices changing over time, different methodologies can be implemented, including a chi-squared test of homogeneity (Rojek & Erickson, 1982) and quasi-symmetry models (Sobel, 1988). However, this methodology has two main drawbacks. Firstly, it analyses escalation only at the aggregate level, not necessarily providing information on single individuals (Spelman, 1994). Secondly, it compares only offences that are temporally adjacent and ignores information about escalation across other offences (Osgood & Schreck, 2007).

The analysis of means approach calculates mean levels of crime seriousness over the life course and visually compares them (Carrington, Matarazzo, & DeSouza, 2005; Kyvsgaard, 2002). However, this approach fails to provide any measure of escalation.

By relying on regression models, the operationalization process of the escalation parameter is performed in two steps:

1. **Assignment of a seriousness score to each crime.** The literature suggests different approaches:
    a. The determination of seriousness on the basis of the views of criminal justice professionals (Sellin & Wolfgang, 1964);
    b. The use of the average length of prison sentences (Carrington et al., 2005);
    c. The categorization based on the maximum sentence which could be imposed by law (Kyvsgaard, 2002).

The present study measures crime seriousness exploiting a method that combines the approaches b. and c. presented above. Crime seriousness is estimated as the average punishment calculated employing the average

between the maximum and minimum length of the prison sentence[8] for each crime set by the Italian legislation. This methodological choice is supported by the availability of this information. Alternative measures of seriousness, such as the average sentence length actually imposed for each crime by Italian courts, are currently unavailable.

2. **Linear regression** of the seriousness score (as dependent variable) on a temporal measure (independent variable), separately for each individual.

The coefficient of the temporal variable represents the individual-specific measure of escalation. Data availability in the present study allow to employ two different temporal scales:[9]

a. The crime number;
b. The age at crime commission.

These two scales measure different criminological processes: while change in escalation by crime number reflects the effect of increased experience, change in escalation by age can be thought of as a maturational process (Liu et al., 2011). For this reason, the present study will compare results obtained with both temporal scales.

More formally, escalation will be measured through the following individual regressions:[10]

$$Ser_{ij} = \alpha + \beta\ Crime_{ij} + \varepsilon_{ij}$$

$$Ser_{ij} = \alpha + \beta\ Age_{ij} + \varepsilon_{ij}$$

Where:

- Ser_{ij} is the seriousness of the j[th]crime committed by individual *i;*
- $Crime_{ij}$ is the ordinal number of crime *j* committed by individual *i;*
- Age_{ij} is the age at which individual *i* committed the j[th]crime *;*
- ε_{ij} is the individual-specific error term.

[8] The average length of the prison sentence is measured in months of detention.

[9] The dataset provides information on the year of commission of each crime. For both temporal scales, when two or more offences have been committed on the same year, the highest seriousness score was considered. The rationale for this methodological choice lies in viewing an offender as "committing major offences around their personal mean seriousness level together with other minor offences, and taking the worst offence provides a better measure of an individual's mean seriousness level" (Liu, Francis, & Soothill, 2011, p. 182). Francis et al. (2013), who focus on escalation among organised crime offenders, also adopt a similar approach.

[10] Only offenders who committed at least 3 offences during their criminal career have been included in the regressions.

The coefficient on the temporal measure (for either $Crime_{ij}$ or Age_{ij}), β, represents the slope of the linear fit, and it is used as an estimate for the value of the escalation parameter for individual *i*.

2.2 Principal Component Analysis (PCA)

To analyse whether certain mafia members are more prone to commit certain types of crimes, the research team performed a Principal Component Analysis (PCA) on the variables identifying the number of crimes committed in the different crime categories. This analysis aimed at reducing the number of crime categories by grouping them into different components. The examination of the factor loadings of each component derived through the PCA provided guidance on the correlations across the different crime categories and on how to consistently group them.

Table 15 shows the crime categories groupings highlighted by the PCA, along with a label assigned by the research team. Table 16 shows the component correlation matrix obtained through promax (oblique) rotation of the PCA eigenvectors (oblique and orthogonal rotations deliver equivalent results).

Table 15. PCA groupings of crime categories.

**Label**	**Crime categories**
Long conviction crimes	Murder, Weapons and explosives – felonies, extortion
Robbery, theft and related crimes	Fencing, robbery, thefts, weapons and explosives - misdemeanours
Arson and assault crimes	Arson and damages, assault and violent offences, offences against public officers
Drug-related crimes	Drug trafficking criminal association, illicit drugs production, trafficking and selling
Administrative, financial and road traffic crimes	Administrative violations, financial offences, road trafficking misdemeanours
White-collar crimes	False documentation and forgery, money laundering, fraud

Table 16. Promax rotation of PCA eigenvectors.

Variable	Comp 1	Comp 2	Comp 3	Comp 4	Comp 5	Comp 6	Comp 7	Unexp-lained
Administrative violations					0.6621			0.4355
Arson and damages			0.5044					0.5085
Assault and violent offences			0.4537					0.5045
Corruption								0.9188
Criminal association								0.6768
Drug trafficking crim. association				0.6901				0.3344
Illicit drugs prod., traffick. and selling				0.6935				0.3332
Evasion								0.6849
Extortion	0.3211							0.5717
False documentation and forgery							0.5487	0.5295
Fencing		0.3448						0.4955
Financial offences					0.4537			0.6826
Fraud							0.477	0.7138
Loand sharking and money laundering							0.5199	0.6164
Mafia association	0.3922					0.3646		0.5219
Military service offences								0.9549
Murder	0.5494							0.3433
Offences against public officers			0.5472					0.3814
Felonies against the crim. justice system						0.4089		0.6536
Misdem. against the crim. justice system						0.5892		0.5457
Road traffic misdemeanours					0.4672			0.5099
Robbery		0.4521						0.5309
Smuggling								0.9076
Thefts		0.4137						0.52
Threats and intimidations								0.5852
Weapons and explosives: felonies	0.5225							0.2232
Weapons and explosives: misdem.		0.3079						0.6607

Notes: blanks reported for values smaller than 0.3.

2.3 Comparison of criminal careers before and after recruitment

2.3.1 CLEANING PROCEDURE OF MAFIA MEMBERS' IDS

To explore the differences in the criminal career of mafia members before and after the recruitment into mafias, the analysis focused on the subset of individuals with information on the year of the mafia association offence. This subset was created with the following procedure. All the IDs with a number $k = 0$ of mafia association crimes were removed. All the IDs with a number $k > 0$ of mafia association crimes were also removed, if all of their mafia association crimes were missing the year_crime variable. For each of the remaining IDs, three parameters were computed: (i) total number C of committed crimes, (ii) total number Q of committed crimes missing the year_crime variable ($Q \leq C$), and (iii) probability P that one of the Q committed crimes was the first or last committed crime of the particular ID. The IDs with $C \leq 4$ and $P \geq 0.4$ were then filtered out. Of the remaining IDs, only those with at least one crime, and not missing the year_crime variable, were kept (N=5,717 mafia members; N=986 mafia leaders). These IDs were then cleaned of all their crimes without the year_crime variable.

2.3.2 CREATION OF VARIABLE YEAR_RECRUITMENT

For this subset, a new variable (year_recruitment) reported the values of year_crime for the first mafia association. For each ID, the crimes were then split into the two distinct sets *crimes_pre_recruitment* and *crimes_post_recruitment.* The former set contained all the crimes with year_crime strictly less than year_recruitment, while the latter set contained all the remaining crimes with the exception of the mafia association crime which allowed to identify the year_recruitment. This crime was in fact discarded from the *crimes_post_recruitment* set to avoid biases in the computation of the twelve different parameters: duration pre-recruitment (D_{pre}), duration post-recruitment (D_{post}), frequency pre-recruitment (F_{pre}), frequency post-recruitment (F_{post}), specialization pre-recruitment (S_{pre}), specialization post-recruitment (S_{post}), escalation pre-recruitment (E_{pre}), escalation post-recruitment (E_{post}), number of crimes pre-recruitment (N_{pre}), number of crimes post-recruitment (N_{post}), average seriousness pre-recruitment (AS_{pre}), and average seriousness post-recruitment (AS_{post}). Each of these eight parameters was computed (on the *crimes_pre_recruitment* or on the *crimes_pre_recruitment* respectively) as explained in Section 3, except for the two parameters D_{pre} and D_{post}, which were defined as

$$D_{pre} = Y_{recruitment} - T_{pre}^{(F)}$$

$$D_{post} = T_{post}^{(L)} - Y_{recruitment}$$

where:

- $Y_{recruitment}$ is the year_recruitment;
- $T_{pre}^{(F)}$ is the year of the first crime in the set *crimes_pre_recruitment*;
- $T_{post}^{(L)}$ is the year of the last crime in the set *crimes_post_recruitment*.

3 Results: the criminal careers of mafia members

This section presents additional results not presented in the main report. In particular, the results are presented for the entire population of mafia members, following the three different levels of analysis (i.e., macro, meso and micro). The macro level is presented in Section 3.1, while the meso and micro levels are presented in Section 3.2 and Section 3.3, respectively.

3.1 Macro level: the parameters of the criminal careers of mafia members

3.1.1 Trajectories identified through Group Based Trajectory Modelling (GBTM)

A Group Based Trajectory Model (henceforth GBTM) was employed to detect different strata within the mafia population. Several attempts were made to identify the best fitting model. The research team first iterated the model starting from a minimum of one to a maximum of eight groups to select the optimal number that minimized the Bayesian Criterion Information (BIC).[11] The BIC is a standard measure for model selection (Fraley & Raftery, 1998). Afterwards, different polynomial orders were tested to minimise the BIC. The BIC is calculated as follows:

$$BIC = \log(L) - 0.5k \log(N)$$

Where L is the values of the model's maximised likelihood, N is the size of the sample, and k is the number of the parameters included in the model. The number of parameters is determined by the polynomial order of the model.

[11] All the Group Based Trajectory models employed in this work are Zero inflated Poisson (ZIP) due to the particular nature of the data. Indeed, a ZIP model fits with matrices that contain excess zero-count data per unit time. Moreover, population was censored both in terms of timespan and offence frequency. Considering that no complete information on deaths was available, the model censors the trajectory at age 60 (although a fraction of mafia members still commits offences after that age threshold). The maximum number of offences in the population is set to 15 (which is the population average). This choice was necessary in order to perform statistically significant models, considering that initially the matrix had a very high absolute variance (the maximum of offences in a single unit time was higher than 100). Keeping the dataset as it was originally involved problems of high singularity and false convergence.

The best fitting model involved 5 groups with cubic polynomial order for all of them (Table 17).

Table 17. Outcome parameters of Mafia population trajectories.

Group	Parameter	Estimate	Standard Error	T for H0 Parameter= 0	Prob>|T|
1	Intercept<br>Linear<br>Quadratic<br>Cubic	-9.30038<br>0.80662<br>-0.01871<br>0.00011	0.39821<br>0.03719<br>0.00112<br>0.00001	-23.356<br>21.688<br>-16.742<br>10.124	0.000<br>0.000<br>0.000<br>0.000
2	Intercept<br>Linear<br>Quadratic<br>Cubic	-10.86907<br>0.73589<br>-0.01489<br>0.00009	0.43798<br>0.03769<br>0.00103<br>0.00001	-24.816<br>19.527<br>-14.455<br>9.728	0.000<br>0.000<br>0.000<br>0.000
3	Intercept<br>Linear<br>Quadratic<br>Cubic	-16.90065<br>1.86332<br>-0.05894<br>0.00052	0.43102<br>0.04745<br>0.00164<br>0.00002	-39.211<br>39.269<br>-35.939<br>30.399	0.000<br>0.000<br>0.000<br>0.000
4	Intercept<br>Linear<br>Quadratic<br>Cubic	3.55182<br>-0.32869<br>0.01579<br>-0.00023	0.28285<br>0.03084<br>0.00108<br>0.00001	12.557<br>-10.658<br>14.676<br>-18.985	0.000<br>0.000<br>0.000<br>0.000
5	Intercept<br>Linear<br>Quadratic<br>Cubic	2.80965<br>-0.24387<br>0.00861<br>-0.00009	0.29228<br>0.02523<br>0.00069<br>0.00001	9.613<br>-9.665<br>12.486<br>-14.606	0.000<br>0.000<br>0.000<br>0.000
1<br>2<br>3<br>4<br>5	Alpha0<br>Alpha0<br>Alpha0<br>Alpha0<br>Alpha0	0.75748<br>1.93217<br>0.95649<br>1.34746<br>1.28804	0.01514<br>0.01986<br>0.01608<br>0.01786<br>0.01579	50.026<br>97.27<br>59.467<br>75.463<br>81.58	0.000<br>0.000<br>0.000<br>0.000<br>0.000
Group membership					
1<br>2<br>3<br>4<br>5	(%)<br>(%)<br>(%)<br>(%)<br>(%)	21.21738<br>32.78074<br>25.99194<br>10.92052<br>9.08942	0.59122<br>0.6429<br>0.64964<br>0.3652<br>0.34639	35.887<br>50.989<br>40.01<br>29.903<br>26.24	0.000<br>0.000<br>0.000<br>0.000<br>0.000
BIC=-318469.15 (N=621763)		*BIC=-318413.32 (N=13229)*		*AIC=-318304.71 L=-318275.71*	

After this first process, postestimation diagnostics were performed to verify the solidity of the results. The results of the diagnostics confirmed the reliability of the model (Table 18). Three postestimation diagnostics were performed to test model validity: the Average Posterior Probability ($AvePP_j$), the Odds of Correct Classification (OCC) and the difference between the posterior probability of group membership P_j and the probability of group memebership π_j (Nagin, 2005).

The optimal $AvePP_j$ for each group would be 1. Nonetheless, the certainty of group assignments based on the posterior probability rule decreases and so $AvePP_j$ does. As a rule of thumb, each $AvePP_j$ should be at least 0.7 for all groups.

The Odds of Correct Classification is a measure that combines both the $AvePP_j$ and π_j. The OCC is calculated as follows:

$$OCC = \frac{AvePP_j/(1 - AvePP_j)}{\pi_j/(1 - \pi_j)}$$

Literature suggest that an OCC greater than 5 is indicative of an accurate model.

Finally, the difference between P_j and π_j is another estimation aimed at describing the assignment accuracy of the model. A perfect assignment would result in two identical figures. For this reason, the lower is the difference between these two probabilities (converging to 0), the higher is the accuracy.

Table 18. Postestimation diagnostics - Mafia population model.

Group	N	AvePPj	π	Pj	OCC	Pj- π
1	2689	0.84	0.21	0.20	20.02	-0.01
2	4614	0.86	0.32	0.33	12.59	-0.01
3	3403	0.86	0.26	0.26	17.65	0.00
4	1392	0.88	0.10	0.11	64.07	0.01

3.1.2 Comparison of criminal careers before and after recruitment

Correlation results across time-buffers Correlation values are computed for each dimension, before and after recruitment, and across time-buffers. Results show strong correlations on average, implying the robustness of the choice of the variable year_recruitment (Table 19).

Table 19. Table of correlation values for different dimensions, before and after recruitment, and across time-buffers.

	Table of correlation values							
**Escalation (before)**	buffer0	buffer1	buffer3		**Escalation (after)**	buffer0	buffer1	buffer3
buffer1	0.79				buffer1	0.90		
buffer3	0.60	0.71			buffer3	0.75	0.84	
buffer5	0.38	0.45	0.68		buffer5	0.65	0.74	0.81
**Frequency (before)**	buffer0	buffer1	buffer3		**Frequency (after)**	buffer0	buffer1	buffer3
buffer1	0.95				buffer1	0.98		
buffer3	0.81	0.88			buffer3	0.93	0.95	
buffer5	0.73	0.78	0.91		buffer5	0.88	0.90	0.95
**Number of crimes (before)**	buffer0	buffer1	buffer3		**Number of crimes (after)**	buffer0	buffer1	buffer3
buffer1	0.96				buffer1	0.99		
buffer3	0.83	0.91			buffer3	0.95	0.97	
buffer5	0.70	0.81	0.93		buffer5	0.92	0.95	0.98
**Seriousness (before)**	buffer0	buffer1	buffer3		**Seriousness (after)**	buffer0	buffer1	buffer3
buffer1	0.96				buffer1	0.96		
buffer3	0.89	0.94			buffer3	0.92	0.94	
buffer5	0.86	0.90	0.95		buffer5	0.88	0.91	0.97
**Specialization (before)**	buffer0	buffer1	buffer3		**Specialization (after)**	buffer0	buffer1	buffer3
buffer1	0.91				buffer1	0.92		
buffer3	0.82	0.89			buffer3	0.82	0.89	
buffer5	0.82	0.85	0.92		buffer5	0.80	0.85	0.92

3.2 Meso level: the similarities and differences of mafia members across the types of mafias

3.2.1 Trajectories by type of mafia identified through Group Based Trajectory Modelling (GBTM)

Four separate Group Based Trajectory models were carried out to detect latent strata within the different mafia organisations (Table 20).

Table 20. GBTM by mafia type - Description of the models

Mafia type	N of individuals	N of groups	BIC	Polynomial order
Camorra	4046	7	-100608.80	Cubic
Sicilian Mafia	4473	3	-105274.9	Cubic
‘Ndrangheta	2014	6	-39745.88	Cubic
Apulian Mafia	1896	6	-52717.4	Cubic

For each of the models postestimation diagnostics were calculated. All the four models performed well, proving the goodness of the outputs (Table 21).

Table 21. Postestimation diagnostics - Models by mafia type.

Mafia type	Group	N ID	AvePP	π	Pj	OCC	π-Pj
Camorra	1	365	0.85	0.09	0.09	52.28	0.00
	2	1007	0.79	0.23	0.25	12.68	-0.02
	3	950	0.83	0.23	0.23	16.45	-0.01
	4	695	0.80	0.18	0.17	17.72	0.01
	5	557	0.86	0.14	0.14	37.33	0.00
	6	281	0.86	0.07	0.07	76.42	0.00
	7	191	0.85	0.05	0.05	108.32	0.00
Sicilian Mafia	1	1788	0.85	0.38	0.40	9.61	-0.02
	2	713	0.92	0.17	0.16	54.64	0.01
	3	1972	0.89	0.45	0.44	9.56	0.01
‘Ndrangheta	1	894	0.78	0.38	0.44	5.72	-0.07
	2	301	0.82	0.16	0.15	24.47	0.01
	3	176	0.86	0.09	0.09	59.21	0.01
	4	402	0.86	0.24	0.20	18.45	0.04
	5	106	0.86	0.06	0.05	99.01	0.01
	6	135	0.90	0.07	0.07	123.88	0.00
Apulian Mafia	1	369	0.83	0.19	0.19	20.50	0.00
	2	341	0.82	0.18	0.18	20.75	0.00
	3	284	0.85	0.16	0.15	30.15	0.01
	4	503	0.84	0.24	0.27	16.94	-0.02
	5	203	0.93	0.11	0.11	101.19	0.00
	6	196	0.83	0.11	0.10	41.02	0.01

3.3 Micro level: exploring the drivers of the recruitment into the mafias

Table 22 and Table 23 present some additional results investigating the characteristics of early recruits and late recruits, considering different percentiles of the age at recruitment distributions to identify these two groups.

Table 22. Characteristics of early and late recruits (average recruitment age is between the 33rd and the 66th percentile).

	Base outcome is the "average" recruitment age (29-37 years old)			
	Early recruits (<29 years old)		Late recruits (>37 years old)	
Boss	0.490<br>(0.395)	0.883<br>(0.292)***	-0.653<br>(0.335)*	-0.881<br>(0.297)***
Killer	1.248<br>(0.692)*	0.686<br>(0.625)	0.017<br>(0.838)	-0.348<br>(0.706)
Underboss/Lieutenant	0.138<br>(0.207)	0.306<br>(0.156)*	-0.102<br>(0.211)	-0.313<br>(0.170)*
'Ndrangheta	-0.465<br>(0.355)	-0.062<br>(0.262)	0.788<br>(0.404)*	0.381<br>(0.325)
Camorra	-0.402<br>(0.273)	-0.144<br>(0.217)	-0.072<br>(0.336)	-0.370<br>(0.286)
Mafia Lucana	-1.269<br>(0.890)	-0.190<br>(0.689)	-0.860<br>(1.239)	-0.881<br>(0.911)
Sicilian Mafia	-0.570<br>(0.280)**	-0.487<br>(0.209)**	0.286<br>(0.334)	0.043<br>(0.277)
Mean serious. pre MO	-0.006<br>(0.003)*		0.005<br>(0.003)*	
Duration pre MO	-0.419<br>(0.035)***	-0.331<br>(0.019)***	0.284<br>(0.025)***	0.195<br>(0.015)***
Frequency pre MO	-0.027<br>(0.092)		0.105<br>(0.142)	
Escalation pre MO	0.002<br>(0.004)		-0.013<br>(0.006)**	
Diversity index pre MO	1.066<br>(0.717)		-2.076<br>(0.763)***	
N violent crimes pre MO	0.041<br>(0.030)		-0.018<br>(0.031)	
N long conviction crimes pre MO		0.014<br>(0.011)		0.005<br>(0.010)
N robbery, theft and related crimes pre MO		0.155<br>(0.024)***		-0.128<br>(0.025)***
N arson and assault crimes pre MO		-0.005<br>(0.047)		-0.030<br>(0.039)
N drug-related crimes pre MO		-0.036<br>(0.036)		0.061<br>(0.040)
N admin, financial, road traffic crimes pre MO		0.082<br>(0.037)**		-0.015<br>(0.031)
N white-collar crimes pre MO		-0.126<br>(0.097)		0.268<br>(0.086)***
N associative crimes pre MO		-0.019<br>(0.186)		-0.395<br>(0.167)**
Years of education	-0.102<br>(0.037)***	-0.101<br>(0.026)***	0.056<br>(0.035)	0.045<br>(0.027)*
Year of birth	0.097<br>(0.014)***	0.127<br>(0.010)***	-0.121<br>(0.014)***	-0.152<br>(0.012)***
Intercept	-185.751<br>(26.651)***	-245.672<br>(19.853)***	234.364<br>(27.819)***	295.881<br>(22.707)***
Pseudo R^2	0.470	0.462	0.470	0.462
Observations	1,319	2,202	1,319	2,202

*Notes: standard errors reported in parenthesis. *, ** and *** indicate statistical significance at the 10, 5 and 1 per cent level respectively. Early recruits are offenders whose age at recruitment is lower than the 33rd percentile of the age at recruitment distribution, while late recruits' age at recruitment is higher than the 66th percentile. Base outcome for the role is the affiliate role and for the mafia association is the Apulian Mafia.*

Table 23. Characteristics of early and late recruits (average recruitment age is between the 20th and the 80h percentile).

	Base outcome is the “average” recruitment age (25-43 years old)			
	Early recruits (<25 years old)		Late recruits (>43 years old)	
Boss	-0.307<br>(0.560)	0.661<br>(0.310)**	-1.513<br>(0.460)***	-1.696<br>(0.440)***
Killer	1.911<br>(0.887)**	0.855<br>(0.618)	-12.687<br>(492.174)	-14.563<br>(1,033.567)
Underboss/Lieutenant	-0.099<br>(0.264)	0.329<br>(0.164)**	-0.652<br>(0.270)**	-0.510<br>(0.213)**
‘Ndrangheta	-0.145<br>(0.408)	0.099<br>(0.249)	0.565<br>(0.485)	0.200<br>(0.416)
Camorra	0.137<br>(0.309)	-0.031<br>(0.213)	0.017<br>(0.428)	-0.129<br>(0.386)
Mafia Lucana	-0.897<br>(1.234)	0.410<br>(0.800)	-11.752<br>(652.116)	-14.685<br>(1,397.273)
Sicilian Mafia	-0.109<br>(0.326)	-0.099<br>(0.206)	0.249<br>(0.422)	0.062<br>(0.373)
Mean serious. pre MO	-0.014<br>(0.004)***		0.004<br>(0.004)	
Duration pre MO	-0.609<br>(0.061)***	-0.405<br>(0.025)***	0.244<br>(0.025)***	0.174<br>(0.015)***
Frequency pre MO	0.026<br>(0.096)		0.208<br>(0.191)	
Escalation pre MO	0.002<br>(0.003)		-0.019<br>(0.009)**	
Diversity index pre MO	0.369<br>(0.867)		-2.677<br>(0.854)***	
N violent crimes pre MO	0.055<br>(0.040)		-0.059<br>(0.040)	
N long conviction crimes pre MO		-0.009<br>(0.017)		-0.008<br>(0.014)
N robbery, theft and Related crimes pre MO		0.157<br>(0.024)***		-0.145<br>(0.035)***
N arson and assault crimes pre MO		0.025<br>(0.051)		-0.145<br>(0.079)*
N drug-related crimes pre MO		-0.087<br>(0.074)		0.088<br>(0.062)
N admin, financial and road traffic crimes pre MO		0.006<br>(0.081)		-0.006<br>(0.029)
N white-collar crimes pre MO		-0.315<br>(0.122)***		0.140<br>(0.105)
N associative crimes pre MO		0.144<br>(0.236)		-0.504<br>(0.200)**
Years of education	-0.046<br>(0.047)	-0.057<br>(0.028)**	0.071<br>(0.042)*	0.052<br>(0.033)
Year of birth	0.108<br>(0.017)***	0.131<br>(0.010)***	-0.203<br>(0.020)***	-0.217<br>(0.015)***
Intercept	-208.469<br>(33.561)***	-256.692<br>(20.404)***	392.582<br>(38.610)***	420.527<br>(29.360)***
Pseudo R^2	0.534	0.494	0.534	0.494
Observations	1,319	2,202	1,319	2,202

*Notes: standard errors reported in parenthesis. *, ** and *** indicate statistical significance at the 10, 5 and 1 per cent level respectively. Early recruits are offenders whose age at recruitment is lower than the 20th percentile of the age at recruitment distribution, while late recruits' age at recruitment is higher than the 80th percentile. Base outcome for the role is the affiliate role and for the mafia association is the Apulian Mafia.*

4 Results: the criminal careers of mafia bosses

This section presents additional results not presented in the main report. In particular, the results are presented for the subpopulation of mafia leaders, following only two (i.e., macro and meso) of the three different levels of analysis (i.e., macro, meso and micro). The macro level is presented in Section 4.1, while the meso level is presented in Section 4.2.

4.1 Macro level: the parameters of the criminal careers of mafia bosses

4.1.1 Trajectories identified through Group Based Trajectory Modelling (GBTM)

GBTM was used also to detect developmental trajectories within the population of mafia leaders in the dataset (N=1,870). In this case, a five groups model with polynomial order resulted as the most fitting, after different polynomial orders had been tested to minimise the Bayesian Criterion Information (BIC). (Table 24).

Table 24. Outcome parameters of Mafia leaders' trajectories.

Group	Parameter	Estimate	Standard Error	T for H0 Parameter=0	Prob>|T|
1	*Intercept*	-6.85377	0.83985	-8.161	0
	Linear	0.51289	0.07807	6.569	0
	Quadratic	-0.00883	0.00232	-3.798	0.0001
	Cubic	0.00002	0.00002	0.754	0.4508
2	*Intercept*	-6.11898	1.09196	-5.604	0
	Linear	0.35611	0.09328	3.818	0.0001
	Quadratic	-0.0052	0.00253	-2.055	0.0398
	Cubic	0.00001	0.00002	0.493	0.6221
3	*Intercept*	3.88579	0.59928	6.484	0
	Linear	-0.30582	0.05326	-5.742	0
	Quadratic	0.00922	0.00146	6.31	0
	Cubic	-0.00008	0.00001	-6.411	0
4	*Intercept*	-13.23903	0.82846	-15.98	0
	Linear	1.40658	0.08982	15.66	0
	Quadratic	-0.04173	0.00309	-13.513	0
	Cubic	0.00034	0.00003	10.277	0
5	*Intercept*	3.16488	0.61426	5.152	0
	Linear	-0.30716	0.06371	-4.821	0
	Quadratic	0.01561	0.00213	7.333	0
	Cubic	-0.00023	0.00002	-9.896	0
1	Alpha0	0.80899	0.03344	24.192	0
2	Alpha0	2.00021	0.05188	38.551	0
3	Alpha0	1.17092	0.03813	30.712	0
4	Alpha0	0.83844	0.03429	24.452	0
5	Alpha0	1.18237	0.03524	33.555	0
Group membership					
1	(%)	23.04342	1.44349	15.964	0
2	(%)	27.09955	1.52504	17.77	0
3	(%)	8.38533	0.82133	10.21	0
4	(%)	28.67242	1.43423	19.991	0
5	(%)	12.79928	0.92826	13.788	0
BIC=-55217.27 (N=87890)		*BIC=-55161.44 (N=1870)*		*AIC=-55081.20 L=-55052.20*	

Postestimation diagnostics confirmed the reliability of the model. All the estimation measures performed well (Table 25).

Table 25. Postestimation diagnostics - Mafia bosses model.

Group	N	AvePP	π	Pj	OCC	π-Pj
1	425	0.856889	0.23	0.227	20.04539	0.002727
2	527	0.865422	0.27	0.281	17.38652	-0.01182
3	151	0.916907	0.08	0.080	126.8992	-0.00075
4	534	0.886884	0.28	0.285	20.16119	-0.00556
5	233	0.913275	0.12	0.124	77.22511	-0.0046

4.2 Meso level: similarities and differences of mafia bosses across types of mafias

4.2.1 Trajectories of bosses by type of mafia identified through Group Based Trajectory Modelling (GBTM)

GBTM was employed to assess possible differences across leaders within the same mafia type. Different polynomial orders were tested to minimise the Bayesian Criterion Information (BIC). For each mafia organisation, a model was employed using only leaders as unit of analysis (Table 26).

Table 26. GBTM by leaders belonging to different mafia types - Description of the models.

Mafia type	N of individuals	N of groups	BIC	Polynomial order
Camorra	672	3	-20803.24	Cubic
Sicilian Mafia	549	5	-16335.18	Cubic
Ndrangheta	444	3	-11235.19	Cubic
Apulian Mafia	191	3	-6847.92	Cubic

Postestimation diagnostics were tested also for these models (Table 27). The outcomes of these tests confirmed the validity of the results.

Table 27. Postestimation diagnostics - Models by leaders of different mafia types.

Mafia type	Group	ID	Ave PP	π	Pj	OCC	π-Pj
Camorra	1	403	0.94	0.58	0.60	10.58	-0.02
	2	94	0.89	0.15	0.14	49.42	0.01
	3	175	0.94	0.27	0.26	45.78	0.01
Sicilian Mafia	1	63	0.93	0.12	0.11	102.66	0.00
	2	241	0.92	0.42	0.44	15.47	-0.01
	3	151	0.90	0.28	0.28	23.42	0.01
	4	66	0.94	0.12	0.12	101.27	0.00
	5	28	0.94	0.52	0.05	14.88	0.47
‘Ndrangheta	1	252	0.91	0.54	0.57	8.74	-0.11
	2	116	0.89	0.27	0.26	22.82	0.47
	3	76	0.95	0.19	0.17	80.65	0.64
Apulian Mafia	1	33	0.85	0.18	0.17	27.65	0.00
	2	59	0.96	0.32	0.31	57.27	0.01
	3	99	0.93	0.51	0.52	13.86	-0.01

References

Carrington, P. J., Matarazzo, A., & DeSouza, P. (2005). *Court Careers of a Canadian Birth Cohort*. Statistics Canada, Canadian Centre for Justice Statistics.

Farrington, D. P., Snyder, H. N., & Finnegan, T. A. (1988). Specialization in Juvenile Court Careers. *Criminology*, *26*(3), 461–488. https://doi.org/10.1111/j.1745-9125.1988.tb00851.x

Fraley, C., & Raftery, A. E. (1998). How Many Clusters? Which Clustering Method? Answers Via Model-Based Cluster Analysis. *The Computer Journal*, *41*(8), 578–588. https://doi.org/10.1093/comjnl/41.8.578

Francis, B., Humphreys, L., Kirby, S., & Soothill, K. (2013). *Understanding criminal careers in organised crime* (Report). Home Office. Retrieved from http://eprints.lancs.ac.uk/67009/

Kyvsgaard, B. (2002). *The Criminal Career: The Danish Longitudinal Study*. Cambridge University Press.

Liu, J., Francis, B., & Soothill, K. (2011). A Longitudinal Study of Escalation in Crime Seriousness. *Journal of Quantitative Criminology*, *27*(2), 175–196. https://doi.org/10.1007/s10940-010-9102-x

Mazerolle, P., Brame, R., Paternoster, R., Piquero, A. R., & Dean, C. W. (2000). Onset Age, Persistence, and Offending Versatility: Comparisons Across Gender. *Criminology*, *38*(4), 1143–1172. https://doi.org/10.1111/j.1745-9125.2000.tb01417.x

Nagin, D. (2005). *Group-Based Modeling of Development*. Harvard University Press.

Osgood, D. W., & Schreck, C. J. (2007). A New Method for Studying the Extent, Stability, and Predictors of Individual Specialization in Violence. *Criminology*, *45*(2), 273–312. https://doi.org/10.1111/j.1745-9125.2007.00079.x

Piquero, A. R., Oster, R. P., Mazerolle, P., Brame, R., & Dean, C. W. (1999). Onset Age and Offense Specialization. *Journal of Research in Crime and Delinquency*, *36*(3), 275–299. https://doi.org/10.1177/0022427899036003002

Rojek, D. G., & Erickson, M. L. (1982). Delinquent Careers A Test of the Career Escalation Model. *Criminology*, *20*(1), 5–28. https://doi.org/10.1111/j.1745-9125.1982.tb00445.x

Sellin, J. T., & Wolfgang, M. E. (1964). *The Measurement of Delinquency*. Wiley.

Sobel, M. E. (1988). Some Models for the Multiway Contingency Table with a One-to-One Correspondence among Categories. *Sociological Methodology*, *18*, 165. https://doi.org/10.2307/271048

Spelman, W. (1994). *Criminal Incapacitation*. Boston, MA: Springer. Retrieved from http://link.springer.com/10.1007/978-1-4757-4885-7

Sullivan, C. J., Mcgloin, J. M., Pratt, T. C., & Piquero, A. R. (2006). Rethinking the “norm” of offender generality: Investigating specialization in the short-term. *Criminology*, *44*(1), 199–233. https://doi.org/10.1111/j.1745-9125.2006.00047.x

Sullivan, C. J., McGloin, J. M., Ray, J. V., & Caudy, M. S. (2009). Detecting Specialization in Offending: Comparing Analytic Approaches. *Journal of Quantitative Criminology*, *25*(4), 419–441. https://doi.org/10.1007/s10940-009-9074-x

CHAPTER 5: Report on emotional and cognitive determinants of OC involvement (UNIPV)

Authors: Gabriella Bottini, Maria Laura Fiorina, Gerardo Salvato

Table of content

SUMMARY 313

1. MATERIALS AND METHODS 314

1.1 PARTICIPANTS 314
1.2 COGNITIVE AND EMOTIONAL ASSESSMENT 314

2. RESULTS 318

2.1 DEMOGRAPHIC CHARACTERISTICS 318
2.2 NEUROPSYCHOLOGICAL SCREENING 318
2.3 PSYCHOLOGICAL SCREENING 319
2.4 COMPUTERIZED COGNITIVE TESTS 320
2.5 DISCRIMINANT ANALYSES BETWEEN OC AND NOC PRISONERS 327

5. CONCLUSIONS 327

6. REFERENCES 330

Summary

In this report, we described the methodology, results and conclusions of the applicative psychological and neuropsychological study on the Organized Crime (OC) prisoners. One hundred fifty participants were tested and separated into three groups: OC prisoners, non-OC prisoners, and non-prisoners Controls. Each individual was administered with a comprehensive psychological and neuropsychological test battery. Each test is described in detail in the following sections. Statistical analyses and results are presented for each measure.

1. Materials and Methods

1.1 Participants

One hundred fifty males, individuals participated in the study. One hundred inmates were randomly selected from two Italian Prisons and they were divided into two groups of 50 individuals each according to their criminal records. They were classified as Organized Crime prisoners (**OC**) or non-organized crime prisoners (**NOC**). The OC and NOC groups were balanced according to the nature of the crime committed (25 violent and 25 non-violent prisoners per group). We also recruited a group of **Control** including 50 non-prisoners, participants. These participants were matched in age, and years of education with the OC and NOC groups (see Table 1). All participants were native Italian speakers and had normal or corrected-to-normal vision. Participants had no history of neurologic or psychiatric disorders. Informed consent was obtained prior to participation in the experiment according to the Declaration of Helsinki. The experimental protocol had ethical approval from the Ethics Committee of the Department of Brain and Behavioural Sciences of the University of Pavia (*protocol n°010*).

1.2 Cognitive and Emotional assessment

1.2.1 NEUROPSYCHOLOGICAL SCREENING

Following a brief neuropsychological interview, participants were administered with two tests assessing the integrity of the global cognitive functioning and intelligence:

- **Addenbrooke's Cognitive Examination-Revised (ACE-R)** (Mioshi et al., 2006). ACE-R is a brief battery that provides an evaluation of six cognitive domains (orientation, attention, memory, verbal fluency, language and visuospatial ability). The total score indicated the *global cognitive functioning* clinical status.
- **Raven's Coloured Progressive Matrices (CPM)** (Raven, 1965). CPM is a culture-free test for non-verbal *intelligence*. It consists of 36 non-representational coloured designs incomplete in the bottom right end corner. Participants are required to indicate the best completion pattern amongst 6 alternatives.

1.2.2 PSYCHOLOGICAL SCREENING

The psychological sphere has also been explored. We investigated for possible personality disorders, anxiety, depression, and psychopathy. Interoceptive self-awareness has also been measured.

- **The Beck Depression Inventory (BDI)** (Beck et al., 1961). The BDI is a 21-question multiple-choice self-report inventory, one of the most widely used psychometric test for measuring the severity of *depression*.
- **The State-Trait Anxiety Inventory (STAI)** (Spielberger et al., 1983). The STAI is a psychological inventory based on a 4-point Likert scale and consists of 40 questions on a self-report basis. The STAI measures two types of anxiety - *state anxiety*, or anxiety about an event, and *trait anxiety*, or anxiety level as a personal characteristic.
- **Body Perception Questionnaire (BPQ)** (Porges, 1993). The BPQ is a self-report 122-item questionnaire assessing: *Body Awareness* (a measure of general awareness of bodily processes); the *Sympathetic Reactivity* (a measure of the withdrawal of neural circuits promoting social engagement and calm resting state as well as activation of sympathetic "fight or flight" responses); and the *Dorsal Vagal Reactivity* (a measure of reactivity of the unmyelinated vagus nerve that innervates the gut).
- **The Psychopathic Personality Inventory - revised (PPI-R)** (Lilienfeld, & Widows, 2005). The PPI-R is a personality test for traits associated with *psychopathy* in adults. It consists of a series of statements to which subjects respond on how accurately the statement describes them using a 4-point Likert scale ("False, "Mostly False", "Mostly True", "True").
- **Millon Clinical Multiaxial Inventory - Third Edition (MCMI-III**) (Millon et al., 2009). The MCMI-III is a psychological assessment tool intended to provide information on *personality traits and psychopathology*, including specific psychiatric disorders outlined in the DSM.

1.2.3 COMPUTERIZED NEUROPSYCHOLOGICAL TESTS

Eight tests from the Cambridge Automated Neuropsychological Test Battery (CANTAB; Cambridge Cognition 2006; 2008) were used to assess the neuropsychological profile of our participants. Extensive descriptions and interactive demonstrations of the tasks are available at the website of the manufacturer (www.cambridgecognition.com). The tests were administered using a 12.1" tablet with a touchscreen (screen resolution of 1280×800). The following tests were used and administered in a pseudo-randomized order across participants:

- **Emotion Recognition Task (ERT).** The ERT measures the ability to identify six basic *emotions* in facial expressions along a continuum of

expression magnitude. Computer-morphed images derived from the facial features of real individuals, each showing a specific emotion, are displayed on the screen, one at a time. Each face is displayed for 200ms and then immediately covered up to prevent residual processing of the image. The participant must select which emotion the face displayed from 6 options (sadness, happiness, fear, anger, disgust or surprise).

- **Multitasking Test (MTT).** The MTT explores the participant's ability to *manage conflicting information* provided by the direction of an arrow and its location on the screen and to ignore task-irrelevant information. The test displays an arrow, which can appear on either side of the screen (right or left) and can point in either direction (to the right or to the left). Each trial displays a cue at the top of the screen that indicates to the participant whether they have to select the right or left button according to the "side on which the arrow appeared" or the "direction in which the arrow was pointing". In some sections of the task, this rule is consistent across trials (single task) while in others it may change from trial to trial in a randomised order (multitasking). Using both rules in a flexible manner places a higher demand on cognition than using a single rule. Some trials display congruent stimuli (e.g. arrow on the right side pointing to the right) whereas other trials display incongruent stimuli, which require a higher cognitive demand (e.g. arrow on the right side of the screen pointing to the left).
- **Stockings of Cambridge (SOC).** The SOC is used to measure *executive functions* and is analogous to the commonly used Tower of London task. Participants are presented with a horizontally split screen and verbally instructed to move the coloured balls in the lower half, to copy the pattern of coloured balls in the upper half. Difficulty slowly increases from a minimum of two moves to a minimum of five moves.
- **Paired Associates Learning (PAL).** PAL assesses *visual memory and learning*. In the PAL, boxes are displayed on the screen and are "opened" in a randomised order. One or more of them will contain a pattern. The patterns are then displayed in the middle of the screen, one at a time and the participant must select the box in which the pattern was originally located. If the participant makes an error, the boxes are opened in sequence again to remind the participant of the locations of the patterns. Increased difficulty levels can be used to test high-functioning, healthy individuals.
- **Reaction Time (RTI).** The RTI assesses motor and mental *response speeds*, as well as measures of movement time, reaction time, response accuracy and impulsivity. In the RTI, the participant must select and hold a button at the bottom of the screen. Circles are presented above (one for the simple mode, and five for the five-choice mode.) In each case, a yellow dot will appear in one of the circles, and the participant must react as soon as possible, releasing the button at the bottom of the screen, and selecting the circle in which the dot appeared.
- **Rapid Visual Information Processing (RVP).** RVP is a measure of

sustained attention. In this task, a white box is shown in the centre of the screen, inside which digits from 2 to 9 appear in a pseudo-random order, at the rate of 100 digits per minute. Participants are required to detect target sequences of digits (for example, 2-4-6, 3-5-7, 4-6-8). When the participant sees the target sequence they must respond by selecting the button in the centre of the screen as quickly as possible. The level of difficulty varies with either one- or three-target sequences that the participant must watch for at the same time.

- **Spatial Working Memory task (SWM)**. The SWM is used to measure *spatial working memory*. Participants are presented with a number of closed coloured square boxes and are instructed to search for a smaller blue square, that is hidden within one of the closed boxes. All closed boxes will contain a blue square only once; i.e. participants will have to remember in which box they already found a blue square, and in which they did not. Looking inside a closed box that already contained a blue square once, is considered an error (a 'between error'). Looking inside a closed square twice within the same search is also considered an error (a 'within error').
- **Verbal Recognition Memory (VRM)**. The VRM assesses *verbal memory and learning*. It measures the ability to encode and subsequently retrieve verbal information, with recall tapping into frontotemporal networks and recognition assessing hippocampal areas. In the VRM the participant is shown a sequence of words on screen one by one. The participant is then tasked with recalling the words, whilst a rater marks which ones they remembered. In the next phase, the participant is presented with two words, one from the original list and one distractor and is asked to choose which one they have seen before, in a 2-force choice paradigm. The latter recognition phase is then repeated after a delay.
- **Body and Balloon Analogue Risk Task (BARISTA)** (Lejuez et al., 2002). We have developed a new experimental paradigm to measure risk-seeking behaviour associated with body representation. We have modified a previously published experiment, the Balloon Analogue Risk Task (BART) (Lejuez et al., 2012). It models real-world risk behaviour through the conceptual frame of balancing the potential for reward versus loss. In the task, the participant is presented with a figure of a balloon on the screen and offered the chance to earn money by inflating the balloon up by clicking a button. Each click causes the balloon to incrementally inflate and money to be added to a counter up until a pre-defined threshold, which makes the balloon overinflated and explodes. Thus, each pump means greater risk, but also greater potential reward. If the participant chooses to cash-out prior to the balloon exploding then they collect the money earned for that trail, but if the balloon explodes, earnings for that trial are lost. Participants are not informed about the balloon breakpoints; the absence of this information allows for testing both participants' initial responses to the task and changes in responding as they gain experience with the task contingencies. We have

implemented this task, replacing the balloon picture with a human body shape. The procedure remains equal, but participants will not receive money. The two tasks have been administered in a randomized order, by means of a personal computer and were not contained in the CANTAB battery.

1.2.4 STATISTICAL ANALYSES

Data have been treated with SPSS 20 (Statistical Package for Social Science, Windows version, Chicago, Illinois). At first, we explored the distribution of the dependent variables using the Shapiro-Wilks test. If data did not follow a normal distribution, we then performed a log10 transformation in order to analyse them using parametric approaches.
As the first step, we used Generalized Linear Models to study patterns of changes in relation to the experimental Group (OC, NOC, Controls). If necessary, we also inserted covariates. As a second step, in order to take into account for other variables, peculiar of the prisoners' groups, we used Generalized Linear Models to study patterns of changes of our variables in relation to the two experimental groups (OC, NOC).
Alpha level was at $p<0.05$. All the Post-hoc pairwise comparisons were Bonferroni-corrected. For the brevity of the report, here we detailed only statistics on significant effects.
Finally, we use Discriminant Analyses in order to identify the most discriminant variables of the group membership.

2. Results

2.1 Demographic Characteristics

The three groups did not differ in age and education level (see table 1). Furthermore, the two inmates’ groups differed for the duration of detention ($p<0.001$). This result is due to the fact that there is a difference (i) in the duration of the legal trials, and (ii) the severity of the punishment in terms of the years of detention specifically concerning the Organized Crime criminals. Such variable has been considered as a covariate in the statistical analyses in order to control for the possible effect of the condition of detention.

2.2 Neuropsychological Screening

All the participants showed adequate global cognitive functioning and level of intelligence. None of them showed clinically significant scores under the normative sample ranges, at both tasks.

2.3 Psychological Screening

The Beck Depression Inventory (BDI)

A Univariate ANOVA has been performed with depression scores as a dependent variable, and Groups (OC, NOC, Controls) as a between-subjects factor. We found a higher level of depression in both OC and NOC compared to Controls ($F_{(2,147)}$=21.1; p<0.001). There was no difference between the OC and NOC group.

The State-Trait Anxiety Inventory (STAI)

A Multivariate ANOVA has been performed with state and trait Anxiety scores as dependent variables, and Groups (OC, NOC, Controls) as a between-subjects factor. We found higher level of both state and trait Anxiety in both OC and NOC compared to Controls (*state*: $F_{(2,147)}$=11.9; p<0.001; *trait*: $F_{(2,147)}$=5.7; p=0.004). There was no difference between the OC and NOC groups.

The Psychopathic Personality Inventory (PPI-Revised).

A one-way ANOVA was performed with the PPI total scores as a dependent variable, and Group (OC, NOC, C) as a between-subjects factor. Results showed that both OC and NOC groups scored significantly higher than Controls ($F_{(2,147)}$=5.9; p=0.003). There was no difference between OC and NOC prisoners.

Body Perception Questionnaire (BPQ)

A mixed ANCOVA was performed with Awareness (Body Awareness, Sympathetic Reactivity, Vagal Reactivity) as a within-subjects factor and Group (OC, NOC, Controls) as a between-subjects factor. Body awareness mean-values were inserted as the dependent variable. As the subjective Anxiety level may influence self-awareness for body signals, we inserted the STAI values as covariates. Results show a main effect of Awareness ($F_{(2,147)}$=10.2; p<0.001). Interestingly, there was a main effect of Group ($F_{(1,147)}$=13.6; p<0.001), indicating that OC showed a higher level of body awareness compared to NOC (p=0.004) and Controls (p<0.001). There was no significant difference between NOC and Controls. In order to rule out possible effects of traumatic brain injury (TBI) and time of detention on the task performance, we ran a separate ANCOVA contrasting OC and NOC using these variables as covariates. Results confirmed the difference between OC and NOC prisoners ($F_{(1,96)}$=4.8; p=0.030). OC showed a higher level of interoceptive awareness compared to NOC inmates.

MILLON CLINICAL MULTIAXIAL INVENTORY - THIRD EDITION (MCMI-III)

Raw scores for each of the eight clinical personality disorders were coded as 1 (score >75 indicating the presence of a personality disorder) or 0 (score <74 indicating the absence of a personality disorder). The personality disorders examined were: schizoid, avoidant, histrionic, depressive, narcissist, obsessive-compulsive, antisocial, sadist, and masochist. We analyzed the frequency of such personality disorders between the three groups (OC, NOC, Controls) through the Pearson Chi-Square test. We found that:

- Schizoid personality disorder was more frequent in the OC group compared to Controls (chi^2=6.9; p=0.031). There was no difference between Controls and NOC, neither between OC and NOC.
- Narcissistic personality disorder was more frequent in the NOC group compared to Controls (chi^2=7.3; p=0.026). There was no difference between OC and Controls, and between OC and NOC.
- Antisocial personality disorder was more frequent in the OC group compared to NOC and Controls, although we only found a statistical trend for significance (chi^2=4.9; p=0.085). There was no difference between NOC and Controls.
- Negativistic personality disorder was more frequent in the OC group compared to Controls (chi^2=10.5; p=0.005). There was no difference between OC and NOC, neither between NOC and Controls.
- Masochistic personality disorder was more frequent in the OC group compared to NOC and Controls (chi^2=6.8; p=0.032). There was no difference between NOC and Controls.

2.4 Computerized Cognitive Tests

EMOTION RECOGNITION TASK (ERT)

As dependent variables, we used the percentages and numbers correct or incorrect and overall response latencies. In order to explore differences between groups in Emotion Recognition accuracy, we performed a mixed ANOVA with Emotion (Happiness, Anger, Disgust, Sadness, Fear) as a within-subjects factor and Group (OC, NOC, Controls) as a between-subjects factor. Accuracy was inserted as a dependent variable. Results showed a main effect of Emotion ($F_{(4,147)}$=148.2; p<0.001). Importantly, this effect was regulated by the interaction with Group ($F_{(8,147)}$=1.8; p=0.044). Pairwise comparisons Bonferroni corrected showed that OC performed worse than NOC at recognizing Sadness (p=0.044). Other effects were not significant.

In order to explore differences between groups in Emotion Recognition reaction times, we performed a mixed ANOVA with Emotion (Happiness, Anger, Disgust, Sadness, Fear) as a within-subjects factor and Group (OC, NOC, Controls) as a between-subjects factor. Median reaction time was inserted as a dependent variable. Results showed a main effect of Emotion ($F_{(4,147)}$=48.8; p<0.001). There was no main effect of Groups, neither interaction Emotion by Group.

We also analysed the ERT Unbiased Hit Rate for each emotion. The unbiased hit rate ensures that response guessing or response bias effects do not influence recognition accuracy of an emotion. It takes into consideration the joint probability of an individual making a correct response, based on the presentation of the correct stimulus out of the available possibilities. We performed a mixed ANOVA with Emotion (Happiness, Anger, Disgust, Sadness, Fear) as a within-subjects factor and Group (OC, NOC, Controls) as a between-subjects factor. Unbiased Hit Rate was inserted as a dependent variable. Results showed that the three groups did not differ from each other. We followed-up this analysis, exploring differences between the two groups of inmates, as the duration of detention, anxiety, depression and the nature of the crime committed (violent-not violent) could influence the results. We then performed a mixed ANOVA with Emotion (Happiness, Anger, Disgust, Sadness, Fear) as a within-subjects factor and Group (OC, NOC, Controls) as a between-subjects factor. Unbiased Hit Rate was inserted as a dependent variable, and duration of detention, anxiety, depression and the nature of the crime committed (violent-not violent) were inserted as covariates. Results showed that OC prisoners were more accurate in recognising the expression of Fear compared to NOC ($F_{(1,91)}$=4.8; p=0.031), whereas, NOC prisoners were more accurate in recognising the expression of Sadness compared to OC (statistical trend: $F_{(1,91)}$=3.1; p=0.085).

Multitasking Test (MTT)

We compared the Multitasking cost between groups. Multitasking cost was calculated as the difference between the median latency of response (from stimulus appearance to button press) during assessed blocks in which both rules are used versus assessed blocks in which only a single rule is used. We performed a Univariate ANOVA with Multitasking cost as dependent variable and Group (OC, NOC, Controls) as a between-subjects factor. Results showed a difference between groups ($F_{(2,150)}$=5.1; p=0.008). Post-hoc Bonferroni–corrected comparisons indicated that the OC groups showed a statistical trend for higher Multitasking cost compared to NOC. There was no difference between OC vs. Controls, and NOC vs. Controls. A secondary analysis of the two inmates' groups taking into account TBI and the duration of detention confirmed the difference between OC and NOC participants.

Stockings of Cambridge (SOC)

As dependent variables, we used (i) reaction times: the median latency, measured from the appearance of the stocking balls until the first box choice was made by the subject; and (ii) accuracy: the total number of assessed trials where the subject chose the correct answer on their first attempt.

Firstly, we compared the two dependent variables (accuracy and reaction time) between the three groups (OC, NOC, Controls). We performed a Univariate ANOVA with reaction times on correct trials as a dependent variable, and Group (OC, NOC, Controls) as a fixed factor. Results showed a main effect of Groups ($F_{(2,147)}$=4.3; p=0.015). Pairwise Bonferroni-corrected comparisons, showed that the OC group performed slower than NOC (p=0.023). There were

no differences between OC versus Controls and NOC versus Controls (all *ps*>0.05). We performed a second Univariate ANOVA with the number of problems solved as a dependent variable, and Group (OC, NOC, Controls) as a fixed factor. Results showed a main effect of Groups ($F_{(2,147)}$=6.6; *p*=0.002). Pairwise Bonferroni-corrected comparisons, showed that the OC and NOC group were less accurate than Controls (OC vs. Controls: *p*=0.020; NOC vs. Controls: *p*=0.002. There was no difference between OC and NOC. In order to rule out possible effects of traumatic brain injury (TBI) and time of detention on the task performance, we ran two separate ANCOVAs contrasting OC and NOC only using covariates. We performed an ANCOVA with reaction times on correct trials as a dependent variable, and Group (OC, NOC) as a fixed factor, and with TBI and length of time detention as covariates. Results confirmed the difference between OC and NOC, with OC performing slower than the NOC group ($F_{(1,96)}$=5.1; *p*=0.027). We ran an ANCOVA with a number of problems solved as a dependent variable, and Group (OC, NOC) as a fixed factor, and with TBI and time of detention as covariates. Results confirmed that there was no difference in accuracy between the OC and NOC group ($F_{(1,96)}$=0.2; *p*=0.620).

Paired Associates Learning (PAL)

We used the Memory Score as a dependent variable, calculated as the number of times a subject chose the correct box on their first attempt when recalling the pattern locations. We performed a Univariate ANOVA with Memory Score as dependent variable and Group (OC, NOC, Controls) as a between-subjects factor. Results showed a main effect of the Group ($F_{(2,150)}$=3.1; *p*=0.050). Pairwise comparisons indicated that the OC group had a worse memory score compared to the NOC group (*p=0.080)*. There was no difference between OC vs. Controls, and NOC vs. Controls. A secondary analysis of the two inmates' groups taking into account TBI and the duration of detention confirmed the difference between OC and NOC participants.

Reaction Time (RTI)

As a dependent variable, we used the RTI Median Five-Choice Movement Time, calculated as the median time taken for a subject to release the response button and select the target stimulus after it flashed yellow on screen. Calculated across correct, assessed trials in which the stimulus could appear in any one of five locations. We performed a Univariate ANOVA with RTI Median Five-Choice Movement Time as the dependent variable, and Group (OC, NOC, Controls) as a between-subjects factor. Results did not show any differences between groups.

Rapid Visual Information Processing (RVP)

As a dependent variable, we used the "A prime" signal detection measure of a subject's sensitivity to the target sequence (string of three numbers), regardless of response tendency. This metric is a measure of how good the subject is at detecting target sequences. We performed a Univariate ANOVA with "A prime" signal detection as a dependent variable, and Group (OC, NOC,

Controls) as a between-subjects factor. Results did not show any differences between groups.

Spatial Working Memory task (SWM)

As an outcome measure, we used the accuracy calculated as the number of times a subject begins a new search pattern from the same box they started with previously. If they always begin a search from the same starting point we infer that the subject is employing a planned strategy for finding the tokens. Therefore, a low score indicates high strategy use (1 = they always begin the search from the same box), a high score indicates that they are beginning their searches from many different boxes.
We firstly compared accuracy between the three groups (OC, NOC, Controls). We performed a Univariate ANOVA with accuracy as a dependent variable, and Group (OC, NOC, Controls) as a fixed factor. Results showed that the OC group performed the task using a detrimental spatial working memory strategy compared to Controls and NOC ($F_{(2,147)}$=4.4; p=0.014; OC vs. Controls p=0.046; OC vs. NOC p=0.025). There was no difference between NOC and Controls. Importantly, in order to rule out the possibility that the difference between the two inmates' groups could be have been caused by the higher frequency of TBI or longer duration of detention in the OC group, we ran an ANCOVA with accuracy as dependent variable and Group (OC, NOC) as fixed factor, and with TBI and time of detention as covariates. Results confirmed the significant difference between OC and NOC ($F_{(1,96)}$=7.9; p=0.006) ruling out the effect of TBI and duration of detention.

Verbal Recognition Memory (VRM)

This test consisted of three main variables of interest: We used the Total Correct Words score as a dependent variable, calculated as the total number of target words that the subject correctly recognises in the delayed recognition phase, plus the total number of distractor words that the subject correctly rejects. We performed a Univariate ANOVA with Total Correct Words as a dependent variable, and Group (OC, NOC, Controls) as a between-subjects factor. We found that the groups did not differ for the number of correct words recalled in the delayed phase.

We used the Free Immediate Recall score as a dependent variable, calculated as the total number of distinct words that are correctly recalled from the presentation phase by the subject during the immediate free recall stage. We performed a Univariate ANOVA with Free Immediate Recall as a dependent variable, and Group (OC, NOC, Controls) as a between-subjects factor. Results showed a main effect of Group ($F_{(2,150)}$=8.2; p<0.001). The OC group recalled more correct words than the NOC group (p=0.001). The NOC group performed poorly compared to Controls (p=0.001). There was no difference between OC and Controls. A secondary analysis of the two inmates' groups taking into account TBI and the duration of detention confirmed the difference between OC and NOC participants.

Lastly, we used the Immediate Recognition score as a dependent variable,

calculated as the total number of target words that the subject correctly recognises, plus the total number of distractor words that the subject correctly rejects. We performed a Univariate ANOVA with Immediate Recognition as a dependent variable, and Group (OC, NOC, Controls) as a between-subjects factor. We found that the groups did not differ for the number of correct words recognized soon after the learning phase.

Body and Balloon Analogue Risk Task (BARISTA)

As a dependent variable, we calculated the total and an average number of pumps only for trials when the participants cashed out before the balloon exploded (index of risk-taking: "adjusted pumps" Lejuez et al., 2002). We also calculated the total and the average number of cash-out trials, the average sum won on each trial, and the average reaction time (RT) for all pumps, cash-outs, and of the first and last pump from each trial.

We performed a mixed ANCOVA with Stimulus (Body, Balloon) as within-subjects factor, and Group (OC, NOC, Controls) as a between-subjects factor. The adjusted number of pumps was inserted as a dependent variable. As the stress level could impact the risk-taking behaviour, we inserted the STAI scores as a covariate. Results showed a main effect of Group ($F_{(2,146)}$=4.5; p=0.012). Pairwise Bonferroni-corrected post-hoc comparisons indicated that the OC prisoners showed a higher level of risk-taking behaviour compared to NOC (p=0.023) and Controls (p=0.042). There was no main effect of Stimulus, neither interaction Group by stimulus. The secondary analysis performed on the OC and NOC groups taking into account TBI frequency and the duration of detention confirmed the difference between OC and NOC on both the body and the balloon tasks, with OC prisoners showing higher risk-taking behaviour.

Figure 1 – Results

		OC (n=50)		NOC (n=50)		Controls (n=50)		Differences
	DV	***M***	***SD***	***M***	***SD***	***M***	***SD***	
	AGE	48,7	10,3	46,6	13,4	51,5	11,9	OC = Cont<br>NOC = Cont<br>OC = NOC
	EDUCATION	10,7	3,0	10,9	3,3	11,8	3,1	OC = Cont<br>NOC = Cont<br>OC = NOC
	DETENTION DURATION	12,7	9,2	4,9	3,5			OC > NOC
	INCARCERA TION	2,6	2,0	2,5	2,2			OC = NOC
	TRAUMATIC BRAIN INJURY	6/50		1/50		0/50		OC = NOC

**Global cognitive functioning**	ACE-R	92,7	4,7	91,4	5,8	94,0	2,0	Within the norm
**Intelligence**	CPM	31,0	4,3	30,9	3,6	34,5	1,2	Within the norm
**Depression**	BDI	16,1	10,9	13,9	8,7	5,3	5,7	OC > Cont<br>NOC> Cont<br>OC = NOC
**Anxiety**	STAI 1	43,6	12,4	42,4	12,9	34,0	7,0	OC > Cont<br>NOC> Cont<br>OC = NOC
	STAI 2	42,4	12,0	40,8	12,4	35,0	9,4	OC > Cont<br>NOC> Cont<br>OC = NOC
**Psychopathy Personality Inventory**	PPI-R	273,2	30,1	274,5	32,9	255,8	24,9	OC > Cont<br>NOC> Cont<br>OC = NOC
**Body Awareness**	Awareness	2,4	0,7	2,0	0,6	1,7	0,5	OC > Cont<br>NOC = Cont<br>OC > NOC
	Social	2,2	0,9	1,7	0,8	1,4	0,6	OC > Cont<br>NOC = Cont<br>OC > NOC
	Vagal	2,2	0,9	1,7	0,9	1,5	0,7	OC > Cont<br>NOC = Cont<br>OC > NOC
**Emotion Recognition**	ERT - Anger	5,3	2,9	5,3	2,8	5,0	3,1	OC = Cont<br>NOC = Cont<br>OC = NOC
	ERT - Disgust	6,4	2,7	7,0	3,5	7,5	3,0	OC = Cont<br>NOC = Cont<br>OC = NOC
	ERT - Fear	5,6	3,0	5,1	2,8	4,8	3,1	OC = Cont<br>NOC = Cont<br>OC = NOC
	ERT - Happiness	11,5	2,0	11,0	2,1	11,6	1,9	OC = Cont<br>NOC = Cont<br>OC = NOC
	ERT – Sadness	7,9	3,1	9,4	3,1	9,1	2,6	OC = Cont<br>NOC = Cont<br>OC < NOC
**Multitasking Test**	MTT Normalized	262,0	162,9	201,8	168,2	303,3	151,3	OC = Cont<br>NOC = Cont

	values							OC > NOC
**Executive Functions**	SOC Reaction times	15417	7524	11362	8380	14862	6493	OC = Cont<br>NOC = Cont<br>OC < NOC
	SOC Accuracy	8,6	2,7	8,2	4,0	10,4	2,7	OC < Cont<br>NOC < Cont<br>OC = NOC
**Learning**	PAL Accuracy	9,5	4,5	11,5	4,7	11,3	3,9	OC = Cont<br>NOC = Cont<br>OC < NOC
**Reaction Times**	RTI Reaction times	2750400	910061	3035500	860045	3051956	695682	OC = Cont<br>NOC = Cont<br>OC = NOC
**Information Processing**	RVPA A prime	0,9	0,1	0,9	0,1	218,3	1412,8	OC = Cont<br>NOC = Cont<br>OC = NOC
**Spatial Working Memory**	SWM Errors	9,0	1,7	7,9	2,4	8,0	2,3	OC > Cont<br>NOC = Cont<br>OC > NOC
**Verbal Memory**	VMR Immediate Recognition Accuracy	30,0	3,5	28,9	4,7	29,5	2,9	OC = Cont<br>NOC = Cont<br>OC = NOC
	VMR Immediate Free Recall Accuracy	6,5	2,2	4,9	2,4	6,4	1,8	OC = Cont<br>NOC < Cont<br>OC > NOC
	VMR Delayed Recall Accuracy	30,2	3,6	29,3	3,8	30,5	2,7	OC = Cont<br>NOC = Cont<br>OC = NOC
**Risk-taking Behaviour**	BARISTA Body Adjusted Pumps	22,1	13,2	15,5	12,5	17,9	12,8	OC > Cont<br>NOC = Cont<br>OC > NOC
	BARISTA Balloons Adjusted Pumps	23,3	15,7	16,3	14,8	15,6	12,0	OC > Cont<br>NOC = Cont<br>OC > NOC

2.5 Discriminant Analyses Between OC and NOC Prisoners

In line with the aim of the current project, we were interested in determining which psychological and neuropsychological variable, amongst those taken into account, better discriminates differences between prisoners' groups. To this aim, we performed two separate Discriminant Analyses. Discriminant Analysis conducted for predictive purposes formulates a linear discriminant function describing the importance of the independent variables in differentiating observations of known group membership.

Firstly, we ran a discriminant analysis with Membership (OC, NOC) as the grouping variable and the psychological variables such as personality scales, anxiety, depression, psychopathy scores as independent variables. Results revealed one discriminant function, which significantly differentiated the groups, $chi^2_{(1)}$=4.5; p=0.034. Such variable was identified by means of the stepwise procedure, as the **Masochist personality disorder**, which was more frequent in OC prisoners, and was the best predictor of Group membership.

Secondly, we ran a discriminant analysis with Membership (OC, NOC) as the grouping variable and the neuropsychological variables emerged from the cognitive tests as independent variables. Results revealed one discriminant function, which significantly differentiated the groups, $chi^2_{(4)}$=26.5; $p<0.001$. Four discriminant variables were identified by means of the stepwise procedure:

1. The reaction times at the SOC test – **executive functions** - (OC > NOC)
2. The accuracy at the PAL test – **learning** – (OC < NOC)
3. The number of words recalled at the VRM - **verbal memory** - (OC > NOC)
4. The adjusted number of pumps made at the Body version of the BARISTA task – **risk-taking behaviour for biological stimuli** - (OC > NOC).

5. Conclusions

In conclusion, we found a coherent psychological and neuropsychological pattern characterising OC members.

From a psychological point of view, compared to NOC prisoners, the OC members' profile is characterized by higher frequency of Antisocial and Masochistic personality disorders. Specifically, the **Masochistic disorder** is a better predictor of differences between the two groups. Typically, the Masochistic subjects relate to others in an obsequious manner and are always ready for sacrifice. These subjects allow others or even encourage them to take advantage of them. To adapt to the pain and distress that can be perceived as comforting, these subjects live their past misfortunes in an active and repetitive way and expect problematic outcomes even from potentially lucky experiences. These subjects act in a habitual, shy manner, and often

highlight their negative characteristics by appearing in an unfavourable light or in a despicable position. One might hypothesize that this psychological profile could fit with the behavioural adaptation to the several rules characterizing the different OC groups. As known, the OC members are bound by commandments based on mobster guidelines. These rules include "professional" and "private" aspects, such as respecting the others' wives (Salvatore Lo Piccolo, 2007 – Corriere della Sera). In particular, the 5th commandment saying: "Always being available for Cosa Nostra is a duty - even if your wife is about to give birth" indicates that who is obeying to this commandment should be characterized by at least a masochistic tendency. It is extremely interesting to note that these written guidelines have been defined also to control and rein the rebel behaviours of the younger Mafiosi. The rite of Mafia initiation is based on a sort of religious communion defined by obligations and rules of correctness and solidarity. As in the all closed societies, the weakness of these bonds lies in the absence of novelties from the "outside" society, so that the internal cohesion can be considerably weakened due to the absence of a modernization boost especially felt by the youngest members. So that the finding of masochist personality disorder in OC members compared to the NOC, suggests that the choice of a specific criminal behaviour is also influenced by personality traits. In other words, our results suggest that specific personality traits drive the enrolment in specific OC groups. Lastly, our findings expand and substantiate a very recent evidence, in which masochistic personality traits have been found in a small group (10 participants) of the Cosa Nostra members (Craparo et al., 2018). However, in this case, the authors did not compare the OC members with a group of control, as we have done in our study.

From a <u>neuropsychological point of view</u>, compared to NOC prisoners, OC criminals' profile is characterized by **diminished frontal lobe cognitive functions**. In particular, OC criminals showed difficulties in strategic planning and in inhibiting the interfering stimuli. They showed also less efficient working memory. OC prisoners exhibited higher risk-taking behaviour. Coherently with such neuropsychological picture, recognition of faces expressing sadness was also impaired. Interestingly, OC criminals showed **enhanced temporal lobe cognitive functions**. In particular, they were more efficient at the verbal memory retrieval. Compared to NOC prisoners, they were able to recall more words from a list read just once. Furthermore, there was evidence for a better recognition of faces expressing fear, an emotion typically associated with the amygdala activity, a medial region of the temporal lobe.

Concerning the <u>frontal lobe functions</u>, it has been largely demonstrated that neuropsychological deficits in executive functions have been linked to antisocial behaviour and considered to be cardinal to the onset and persistence of severe antisocial and aggressive behaviour (Pera-Guardiola et al., 2016). Here we expected to find such evidence on the prisoners' groups. However, we found that frontal lobe functions are more impaired in OC members compared to members of NOC. The adhesion to a community with several rules that have to be respected whatever happens in your personal life, requires a consistent passive behaviour and a general tendency to be manipulated and dominated by stronger and more authoritarian individuals. Thus, it would not be surprising that OC members appear to be significantly weaker in tests

exploring strategies competences. Furthermore, one of the behavioural features of the frontal lobe syndrome, is perseveration. Perseveration induces subjects to reiterate the same behaviours despite significant changes in the environment (Moltò et al., 2007). The strong and acritical adhesion of OC members to their network might be also driven by such frontal symptom.

Furthermore, we found that risk-taking behaviour for biological stimuli (body) is discriminant of the OC group membership. This finding is in line with the frontal lobe dysfunction previously reported in prisoners (Ostrosky & Ardila, 2017), as these subjects show an impaired competence in the estimation of the cost-benefit ratio. We think that the biological risk-taking component is also modulated by more social factors that explain these differences between OC and NOC members. Firstly, the OC members could feel to be more protected by their strong criminal network, while risking. In case of NOC members the situation changes dramatically as they act as lonely wolves, being bound to be much more cautious while committing crimes. Secondly, the nature of the "taken risk" is different between the two groups. Indeed, being part of an OC group involves higher biological risk not only on the own, but also on the others' body. The rules, in fact, impose also to take care of the other members' lives. Furthermore, being part of an OC group has also implications for family members as retaliations from other OC groups are very frequent. We might thus speak of an extended (non) "altruistic" biological risk-taking as a new interesting social discriminant in criminality.

As known, the frontal lobe syndrome is characterized by productive or defective emotional symptoms such as difficulties in recognizing facial emotion expressions. This is the case of recognition of sadness, which is associated with the integrity of the cingulate cortex (Phan et al., 2002). Interestingly, this cerebral area is also implicated in risk-taking behaviour. Indeed, higher risk-seeking is associated with abnormal activity or lesions to specific parts of the cingulate cortex (Rao et al., 2008). This could explain our findings with OC members being less competent in recognising sadness and showing higher risk-taking behaviour.

Concerning the temporal lobe functions, surprisingly enough, we found evidence for a better performance of OC in verbal memory and recognition of fear. Verbal memory competences are particularly relevant when acting in a structured community characterized by a number of rules and obligations (such as the ten commandments). Furthermore, these rules, may change as they can be variously personalized by different bosses. One relevant aspect of the activity of the OC members is to keep in mind many names of members of other OC groups and also which role they play in such organizations. This social component, precious for the OC members survival, could determine the selection of those who are more skilled in verbal memory.

Fear is one of the most "protective" and adaptive emotion. Our OC members, are also better at recognizing fear. As OC members are more used in acting in a structured group and in interacting with other individuals, their level of attention in recognizing others' fear might be particularly alerted. Finally, verbal threats are part of the usual behaviours of OC members. There is evidence that verbal threats have an augmentative impact on fear perception

(Muris and Field, 2010. Olsson & Phelps, 2007). We thus hypothesize that OC members are better in the recognition of this emotion due to the continuous exposition to verbal threats in their group.

To summarize we have found a number of cognitive and behavioural differences between OC and NOC members. Some of these differences are, in our view, relevant discriminants in determining the enrolment in either the two groups. In particular, less efficient executive functions, better verbal memory together with a masochistic personality trait seem to drive the enrolment in OC groups.

6. References

Beck, A.T., Ward, C. H., Mendelson, M., Mock, J., & Erbaugh, J. (1961) An inventory for measuring depression. Archives of General Psychiatry, 4, 561-571.

Cambridge Cognition. (2006). CANTABeclipse Test Administration Guide. Cambridge, UK: Cambridge Cognition Limited.

Cambridge Cognition. (2008). CANTAB Topic: Test-retest reliabilities and detecting reliable change. Cambridge, UK: Cambridge Cognition.

Craparo, G., David, V., Costanzo, G., & Gori, A. (2018). Cosa Nostra and the Camorra: Assessment of personality, alexithymic traits, and attachment styles. International Journal of Law and Psychiatry, 58, 17-26

Kiehl, K. A., Smith, A. M., Hare, R. D., Mendrek, A., Forster, B. B., Brink, J., & Liddle, P. F. (2001). Limbic abnormalities in affective processing by criminal psychopaths as revealed by functional magnetic resonance imaging. Biological psychiatry, 50(9), 677-684.

Lejuez, C. W., Read, J. P., Kahler, C. W., Richards, J. B., Ramsey, S. E., Stuart, G. L., ... & Brown, R. A. (2002). Evaluation of a behavioral measure of risk taking: the Balloon Analogue Risk Task (BART). Journal of Experimental Psychology: Applied, 8(2), 75.

Lilienfeld, S. O., & Widows, M. R. (2005). Professional manual for the Psychopathic Personality Inventory–Revised (PPI–R). Lutz, FL: Psychological Assessment Resources.

Millon, T., Davis, R., Grossman, S., & Millon, C. (2009). MCMI-III: Millon Clinical Multiaxial Inventory-III Manual, 4th edition. Minneapolis, MN: NCS, Inc.

Mioshi, E., Dawson, K., Mitchell, J., Arnold, R., & Hodges, J. R. (2006). The Addenbrooke's Cognitive Examination Revised (ACE-R): a brief cognitive test battery for dementia screening. International journal of geriatric psychiatry, 21(11), 1078-1085.

Moltó, J., Poy, R., Segarra, P., Pastor, M., & Montanes, S. (2007). Response perseveration in psychopaths: Interpersonal/affective or social deviance traits? Journal of Abnormal Psychology, 116, 632-637.

Muris, P., & Field, A. P. (2010). The role of verbal threat information in the development of childhood fear."Beware the Jabberwock!". Clinical Child and Family Psychology Review, 13(2), 129-150.

Olsson, A., & Phelps, E. A. (2007). Social learning of fear. Nature neuroscience, 10(9), 1095

Ostrosky, F., & Ardila, A. (Eds.). (2017). Neuropsychology of Criminal Behavior. Routledge

Pera-Guardiola, V., Batalla, I., Bosque, J., Kosson, D., Pifarré, J., Hernández-Ribas, R., ... & Cardoner, N. (2016). Modulatory effects of psychopathy on Wisconsin Card Sorting Test performance in male offenders with Antisocial Personality Disorder. Psychiatry research, 235, 43-48.

Phan, K. L., Wager, T., Taylor, S. F., & Liberzon, I. (2002). Functional neuroanatomy of emotion: a meta-analysis of emotion activation studies in PET and fMRI. Neuroimage, 16(2), 331-348.

Porges S. (1993). Body Perception Questionnaire: Laboratory of Development Assessment. Baltimore, MD: University of Maryland.

Rao, H., Korczykowski, M., Pluta, J., Hoang, A., & Detre, J. A. (2008). Neural correlates of voluntary and involuntary risk taking in the human brain: an fMRI Study of the Balloon Analog Risk Task (BART). Neuroimage, 42(2), 902-910.

Raven, J. C. (1965). Guide to Using the Coloured Progressive Matrices: Sets A, Ab, B... HK Lewis.

Spielberger, C. D., Gorsuch, R. L., Lushene, R., Vagg, P. R., & Jacobs, G. A. (1983). Manual for the State-Trait Anxiety Inventory. Palo Alto, CA: Consulting Psychologists Press.

CHAPTER 6: Report on Socio-Economic Inequalities and OC Development (UNIPA)

Authors: Mario Lavezzi, Michele Battisti, Giovanni Bernardo, Andros Kourtellos, Antri Konstantinidi

Table of contents

1. INTRODUCTION 333

2. DATA 334

2.1. ORGANIZED CRIME 334
CALDERONI INDEX 335
LATENT FACTOR 335
2.2. INEQUALITY 337
2.3. SOCIO-ECONOMIC MOBILITY 337
2.4. OTHER COVARIATES 337

3. METHODOLOGY 337

3.1. RESULTS 338
3.2. ORGANIZED CRIME AND INEQUALITY 338
3.3. ORGANIZED CRIME AND SOCIO-ECONOMIC MOBILITY 339
3.4. CONCLUSIONS 340

FIGURES 341

TABLES 343

BIBLIOGRAPHY 355

APPENDIX 357

1. Introduction

In this report we present the results of the research conducted by UNIPA on the relationship between inequality, socio-economic mobility and organized crime (OC). Our research implies two activities: I) assessing the relation between inequality and OC development, through an econometric analysis of the relationship between inequality and OC at regional level, exploiting time and cross-section variation of the variables of interest for the period 1985-2014. II) Assessing the relation between socio-economic mobility and OC development by a cross-section analysis.

Casual observation suggests that inequality is high and social mobility is low in the Italian regions and provinces in which organized crime is widespread, such as those of Southern Italy (see, e.g. Istat, 2010, Acciari et al., 2016, Guell et al., 2017). Our hypothesis is that high inequality and low social mobility have a positive effect on organized crime development. In particular, *direct* channels may be at work, for example if poor individuals find OC attractive for lack of remunerative alternatives in the labour market. Differently, rich individuals, for example members of the economic and political *élite*, may demand OC ``services" to gain or preserve their socio-economic standing. This behaviour was initially pointed out by Franchetti (1877) in his thorough enquiry on Sicily at the time of reunification of Italy, as he noted that the Sicilian upper class benefited from the protection services by the early *mafiosi*. Recent evidence supporting this claim is found for example in Gambetta and Reuter (1995), describing how entrepreneurs can gain monopolistic power by forming cartels enforced by criminal organizations in the adjudication of public contracts,[1] and by De Feo and De Luca (2017), who provide evidence on the support that Mafia can give to local politicians in exchange for economic benefits.

In addition, *indirect* channels can also be at work. This occurs if the expected returns from OC, including possible punishment and opportunity costs, are higher than the expected return of the individuals' legal alternatives. Our conjecture is that there might be more gains from involvement in OC in presence of inequality as the latter is likely to lead to corruption and erosion of the rule of law (Jong-Sung and Khagram, 2005; Sunde et al., 2008).

No previous work has investigated the link between inequality, socio-economic mobility and OC development, although inequality has been studied in relation to corruption (e.g. Jong-Sung and Khagram, 2005), violent crime (e.g. Fajnzylber, 2005) and civil conflict (e.g. Macours, 2010).

Our main finding is that higher inequality can lead to higher organized crime development: in particular, the index that better captures the effect of inequality is P90/P10: the ratio of 90th quintile over the 10th in the distribution. We also find that consumption inequality performs better that income inequality as the relevant inequality measure. Finally, we find that low socio-economic mobility displays a robust association with OC development.

---

[1] See also Gambetta (1993) for an analysis of the Sicilian Mafia as a provider of private p protection and Lavezzi (2014) for a discussion of the concept of "demand for Mafia".

This report is organized as follows. Section 2 contains the description of the dataset; Section 3 describes the methodology employed for the empirical analysis; Section 4 presents the results; Section 5 contains some concluding remarks.

2. Data

Our dataset on organized crime and inequality takes the form of an unbalanced 5-year period panel for 20 Italian regions for the period 1985-2014. A detailed description of the data and our sources is given in the Data Appendix Table A1. While we construct the organized crime variables at the annual frequency we opt to use 5-period averages to reduce measurement error bias and ease the problem of missing observations in several explanatory variables.

Data on income inequality are obtained from the Survey on Household Income and Wealth (SHIW) provided by Bank of Italy, which comprises about 8,000 households (20,000 individuals) per wave. This survey includes information on personal income, household consumption and wealth, education and occupation. Data on socio-economic mobility are from Acciari et al. (2017), and come from Italian tax data at provincial level.

2.1. Organized crime

To measure organized crime we follow the current literature and utilize data on crimes committed by members of criminal organizations. Data on crimes come from Istat and the SDI database, managed by the Italian Ministry of Interiors.[2]

Our analysis distinguishes between two types of crimes measures. Type I includes direct measures, i.e. crimes that unambiguously are related to OC (2 crimes): Homicide by Mafia and Mafia Type Association. Type II includes indirect measures, i.e. crimes that are potentially associated to OC (14 crimes): Criminal Association, Bribery, Corruption for an Act Against Official Duties, Drugs, Extortion, Money Laundering, Prostitution, Smuggling, Threats, Usury, Corruption in Public Acts, Instigation to Corrupt, Judicial Corruption, Kidnapping for Extortion Purposes.[3]

Our organized crime measures are based on the joint consideration of type I and II crimes. We choose to measure OC in this way as the relevance of crimes of Type II is remarked in several reports on the activities of Italian mafias (see, e.g. Dia, 2016), as well as in the literature (see, e.g. Riccardi et al., 2016, and Fioroni et al., 2017). This approach distinguishes us from, e.g., Calderoni (2011) who focuses on type I crimes only.[4] In addition, from a

---

[2] We are grateful to Magg. Domenico Martinelli and to Claudia Di Persio for invaluable help, and to the Department on "Analisi Criminale della Direzione Centrale della Polizia Criminale" at the Italian Ministry of Interiors for releasing the data.

[3] In our empirical analysis, crime numbers are normalized by population.

[4] Crime data in Calderoni (2011) are integrated by data on other direct measures of Mafia activities: the number of city councils dissolved for Mafia infiltration, and a measure of assets

statistical point of view, we see OC as a latent variable, measured with error. In this perspective, the higher the number of crimes we consider to measure OC, the lower the measurement error.

Our organized crime variables are based on two alternative methodologies using 16 crime variables. The first one is based on Calderoni (2011) and the second one on dynamic factor methods (Moench, Ng, and Potter, 2011, and Banbura and Modugno, 2014), which represents an innovative method not used so far for such purposes.

CALDERONI INDEX

Following Calderoni (2011), we employ two alternative procedures to calculate the Mafia Index. In the first one, we first normalize each variable to take values between 0 and 100 with 100 to denote the highest value of Mafia presence. Then, we compute the *Calderoni Mean Index* as the average score of all variables. One problem with the average score is that may overestimate the presence of the mafia in the Southern regions and underestimate it in the other regions. This is because organized crime is prevalent in the areas of southern Italy. To overcome this problem, we also construct an index based on region's rank. For each variable, we rank all the Italian regions in decreasing order. Then we attribute the score of 100 to the region with the highest rank and proportionally lower scores to the other provinces, according to their rank. Then, we compute the *Calderoni Rank Index* as the average score of all variables.

LATENT FACTOR

We employ the dynamic factor model method to extract a latent factor for organized crime at the regional level. This methodology has not been applied to such a purpose before. Let $x_t = (x_{1t}, x_{2t}, \dots, x_{nt})', t = 1,..,T$ denote a stationary *n*-dimensional vector of crime series standardized to mean 0 and unit variance. We assume that the factor model takes the following form:

(1) $$x_t = \Lambda f_t + e_t$$

(2) $$f_t = \mathrm{A} f_{t-1} + \eta_t$$

where f_t is a $r \times 1$ vector of latent common factors of organized crime and $e_t = (e_{1t}, e_{2t}, \dots, e_{nt})'$, is the idiosyncratic component, uncorrelated with f_t at all leads and lags.[5] The errors η_t are assumed to be innovations to the factor. The common component is given by $n \times r$ matrix Λ, which contains factor loadings.

Following Banbura and Modugno (2014) we estimate the dynamic factor model using a modified Expectation Maximisation (EM) algorithm. The idea of the

confiscated to Mafia clans. See the same article for details on other methods to measure OC found in the literature.

[5] The errors were not allowed to be serially correlated, but this assumption can be relaxed later.

algorithm is to write the likelihood as if the data were complete and to iterate between two steps: in the Expectation step the missing data are filled in the likelihood, while in the Maximisation step this expectation is re-optimised. This modification is important in our analysis as it allows us to consider a larger number of time-series that organized crime regardless of whether they have missing observations or not.

In addition to the latent factor at the regional level we extract factors at the macro and Italy level for descriptive purposes. In doing so, we employ two complementary factor analyses. First, we apply the aforementioned methodology separately at different levels of aggregation. Figures 1 and 2 provides the t-plots of the factors for results for Italy-wide and the macro area factors (Centro, Nord and Sud), respectively. It can be observed that there are some large spikes at the beginning of the period, indicating a higher intensity of the phenomenon, while fluctuations are much smaller in the subsequent years.

Table 2 presents the estimated the loadings of the factors and Table 3 the square loadings (as % of total) to illustrate the relative importance of the different crimes.[6] Table 3 suggests that some of the indirect measures of OC may have a high capacity to capture the phenomenon, and that the effect is different across the different Italian macro regions. For example, different corruption crimes stand out (see, e.g. Fioroni et al. 2017, on corruption and organized crime), as well as money laundering in the South (see Barone and Masciandaro, 2011, for an analysis of organized crime and money laundering).

Second, we employ the hierarchical dynamic factor models proposed by Moench, Ng, and Potter (2011) to account for the correlations between the shocks at the different levels of aggregation. In particular, we employ this multilevel factor model using common (Italy level - global), block-specific (macro regions), and sub-block-specific (regions) to capture the within and between-block variations in the measurement system of organized crime. Given that this method does not allow for missing observations we only use a set of five variables (Criminal Association, Drugs, Mafia type Association, Extortions and Prostitution) As it is illustrated by Figures 3 and 4, the common factor exhibits a similar pattern to the one we obtained using the first method in Figures 1 and 2 although it is only based on a much smaller set of variables. Table 4 presents the variance decomposition analysis to document the examine the importance of the variation of aggregate (Italy-wide), block-specific (Macro), and subblock-specific (Regional) components as well as idiosyncratic noise (IdioNoise) relative to the total variation in the data.

The variations due to idiosyncratic shocks dominate other variations, in all cases. Overall, we can see that regarding the other variations, the subblock specific
variations are larger. Notice in particular that in Southern regions (where OC is more widespread), the regional component is relatively high.

[6] The factor loadings (estimated as stationary, non time-varying, values) can be interpreted as weights telling how much the factor intensity depends (loads) on each crime over the period of interest.

2.2. Inequality

We employ six different inequality metrics that capture different aspects of the distribution: (i) the Gini index, (ii) the Atkinson inequality index,[7] - and four percentile ratios - (iii) P90/P10, (iv) P75/P25, (v) P90/P50, and (vi) P75/P50. The Gini and Atkinson indices take value between 0 and 1 as opposed to the percentile ratios that do not have this restriction.

For each inequality metric, we construct 2 different measures of inequality based on total consumption and net income. For inequality measures on consumption and household income, we consider all the observations in which the heads of household are aged from 25 to 65.

Table 5 presents summary statistics of these inequality measures.

2.3. Socio-economic mobility

For socio-economic mobility, we use the measure computed by Acciari et al. (2016), based on Italian tax data. In particular mobility is measured between parents' income in 1998 and children's income in 2012 at provincial level. The three indices we consider are: i) *Relative mobility*: this is the slope of a rank-rank regression between child ranks and parent ranks and measure the difference in outcomes between children from top vs. bottom income families within province (Chetty et al., 2015). A high value indicates low mobility; ii) *Absolute mobility – expected rank*: this index measures the expected rank of children from families at the bottom 25% of the national parent income distribution (Chetty et al., 2015). A high value indicates high mobility; iii) *Absolute mobility – Q1toQ5* measures the probability of rising from the bottom quintile to the top quintile of the income distribution (Corak and Heisz 1999, Hertz 2006). A low value indicates high mobility.

2.4. Other covariates

We considered as covariates in our analysis indicators of the economic conditions and of human capital, under the assumption that bad economic conditions and low levels of human capital can positively affect OC development. In particular, we used secondary education level, the economic activity rate, the growth rate of compensation of employee, the growth rate of total hours worked, the participation in education and training, the long-term unemployment rate, the growth rate of gross fixed capital formation (see Table A1 in the Appendix for the definitions and Table 6 for summary statistics).

3. Methodology

The focus of this paper is to investigate how changes in organized crime (OC) are related to inequality (INEQ).

---

[7] For definitions and details on the Gini and Atkinson index see e.g. Cowell (2011).

Our benchmark model takes form of a dynamic panel model:

$$\boldsymbol{OC_{it} = \rho OC_{it-1} + \beta_I INEQ_{it} + +\gamma' Z_{it} + \xi_t + v_i + u_{it}}, \tag{3}$$

where v_i is the fixed effect, ξ_t is the time effect, and u_{it} is the idiosyncratic error term. Z_{it} includes a constant and other covariates. Following Blundell and Bond (1998), we estimate this model using system 2-step GMM up to 4 lags as instrumental variables and robust standard errors.[8] Given that the impact of inequality on OC development can be not contemporaneous, we also tried specifications with a lagged value of INEQ.

In the case of measurement of OC by the estimated dynamic factor, we had to consider differences in this variable to make the series stationary. This implied considering also INEQ in differences. In this case the estimated dynamic panel takes the form:

$$\boldsymbol{\Delta OC_{it} = \rho \Delta OC_{it-1} + \beta_I \Delta INEQ_{it} + \gamma' \Delta Z_{it} + \xi_t + v_i + u_{it}} \tag{4}$$

As for socio-economic mobility, given that our data are only available as a cross-section we estimate a simple cross-sectional regression of the form:

$$\boldsymbol{OC_i = \beta_I INEQ_i + \beta_M SM_i + \gamma' Z_i + u_i}. \tag{5}$$

The next section contains the results of our econometric analysis.

3.1. Results

This section presents the results of our econometric analysis. In particular, Section 4.1 discusses the results on inequality and OC, while Section 4.2 those on socio-economic mobility and OC.

3.2. Organized Crime and Inequality

Tables 7 and 8 contain the results of regressions of OC on INEQ for both indices based on Calderoni (2011), i.e. *Calderoni Rank Index* and *Calderoni Mean Index*, considering respectively indices of income inequality and consumption inequality.[9] The results show that the coefficients of the effect of inequality on organized crime have positive and significant coefficients in particular when the *Calderoni Mean Index* is utilized in the regressions. Moreover, it appears that consumption inequality better captures the relationship of interest. The inequality index with the highest level of significance is P90/P10. Under the null of joint validity of instruments, the Hansen's test of over-identification provides us enough evidence about the validity of our instruments, in all models (at least at 5% significance level).

[8] We implement this estimation using the Stata package xtabond2 by Roodman (2009).

[9] Regressions are run on data from 19 out of 20 Italian regions. The region of Val d'Aosta is dropped for lack of data.

Table 9 contains the results of specifications in which we added lagged values of inequality indices.[10] Results in Table 8 shows that, with the *Calderoni Mean Index*, all six metrics of inequality measures have positive and significant coefficients: in particular the contemporaneous values of P90/P10, P90/P50 and P175/P50 and the lagged values of the Gini and Atkinson Indices and P//5/P25. In the case of the *Calderoni Rank Index*, the coefficients of the lagged values of the Atkinson index, P90/P10, P75/P25, P90/P50 are positive and significant.

Table 10 contains the results of regressions in which we added other covariates, related to potential economic determinants of OC.[11] Given the results presented in the previous tables we kept as our preferred metric of inequality the P90/P10 index and its lagged value. Table 9 shows that the lagged value of P90/P10 has a positive and significant coefficient in almost all the specifications.

Table 11 presents the results where the measure of OC is based on the innovative method we described in Section 2.1.2, based on the estimation of a dynamic common factor.[12] We can see that the coefficients of the indices of inequality (in this case appearing as differences) have positive and significant coefficients with the exception of P75/P25 and P75/P50.

Table 12 contains the results of the regressions with additional covariates when we consider the effect of consumption inequality on the factor index computed following Banbura and Modugno (2014).[13] We can observe that in almost all the specifications the effect of inequality (in differences) is positive and significant.

3.3. Organized Crime and Socio-economic Mobility

In this section we present the results of cross-section regressions estimating the effect of indicators of socio-economic mobility on OC. The analysis is carried out at provincial level. Table 13 contains the summary statistics.

Table 14 contains simple regressions of our two OC measures (Calderoni Rank Index and Calderoni Mean Index) on the three metrics of socio-economic mobility we considered in this report only and a constant, while Tables 15, 16 and 17 adds the covariates related to economic conditions and human capital

[10] We only report the results for consumption inequality. Results for income inequality return a significant coefficient only with the *Calderoni Mean Index* for the lagged values of the Atkinson index (at 5%) and of P90/P10 (at 1%).

[11] We only report results for consumption inequality. Regressions on income inequality returned positive and significant coefficients for the lagged value of P90/P10 in Models 1,2,4,5 with the *Calderoni Rank Index*.

[12] We only report results for consumption inequality, as regressions on income inequality measures did not return significant results.

[13] We also estimated regressions using the Hierarchical dynamic factors, but the results were not significant. This is likely due to the fact that we used only five variables to estimate them.

levels to regressions with the *Calderoni Mean Index*.[14] The mobility indices' coefficients have the expected signs and are highly significant. The same result holds for Table 18, which reports the results for the correlation of the relative mobility index on the *Calderoni Rank Index*.[15]

3.4. Conclusions

This study investigates the linkages between organized crime and inequality and social mobility. In doing so we construct a novel dataset of organized crime at the regional level based on two complementary approaches. The first one is based on the methodology developed by Calderoni (2011) and the second one employs a dynamic factor model. Additionally, we construct a wide range inequality as well as social mobility measures. Our dataset is of general interest beyond the specific application considered in the present study. Our main finding is that higher inequality can lead to higher organized crime development: in particular, the index that better captures the effect of inequality is P90/P10. We also find that consumption inequality performs better that income inequality as the relevant inequality measure. Finally, we find that low socio-economic mobility displays a robust association with OC development.

These results shed new light on the economic explanations of the rise and spread of organized crime and suggest potential policy interventions. For example, the fact that consumption inequality seems to matter more than income inequality, suggests that what matters are differences in actual standards of living, which are better captured by consumption levels than by income levels as they depend on life-cycle decisions (see e.g. Jappelli and Pistaferri, 2010, for recent trends of income and consumption inequality in Italy). This directly points out a policy response in terms of insuring adequate standard of living to the poor in order to reduce the existing disparities. The same holds for improving the socio-economic mobility, especially by improving the perspective of those lagging behind the social ladder.

[14] The set of covariates used for the regional and provincial analysis do not perfectly match because of data availability. Model 7 in Tables 15, 16 and 17 contains a dummy for the Northern regions as the latter proved to be highly significant in regressions on the mobility indices and macro-region dummies.

[15] The results for the other mobility indices are not significant.

Figures

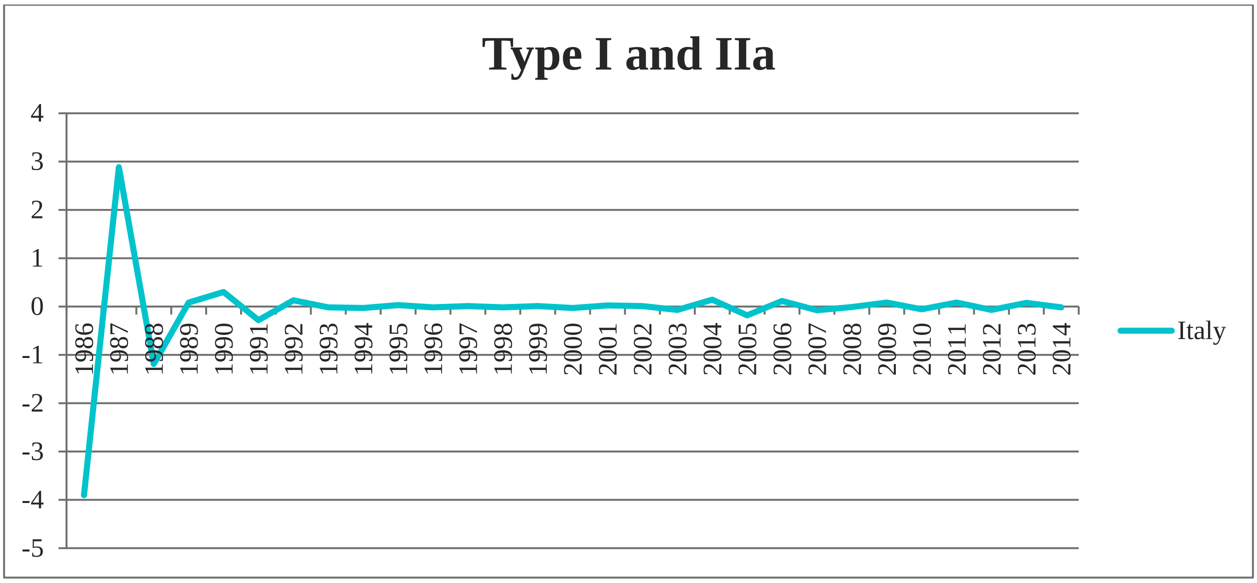

Figure 1: Factor for Organized crime based on Banbura and Modugno (2014) –Italy-wide Factor for Organized Crime

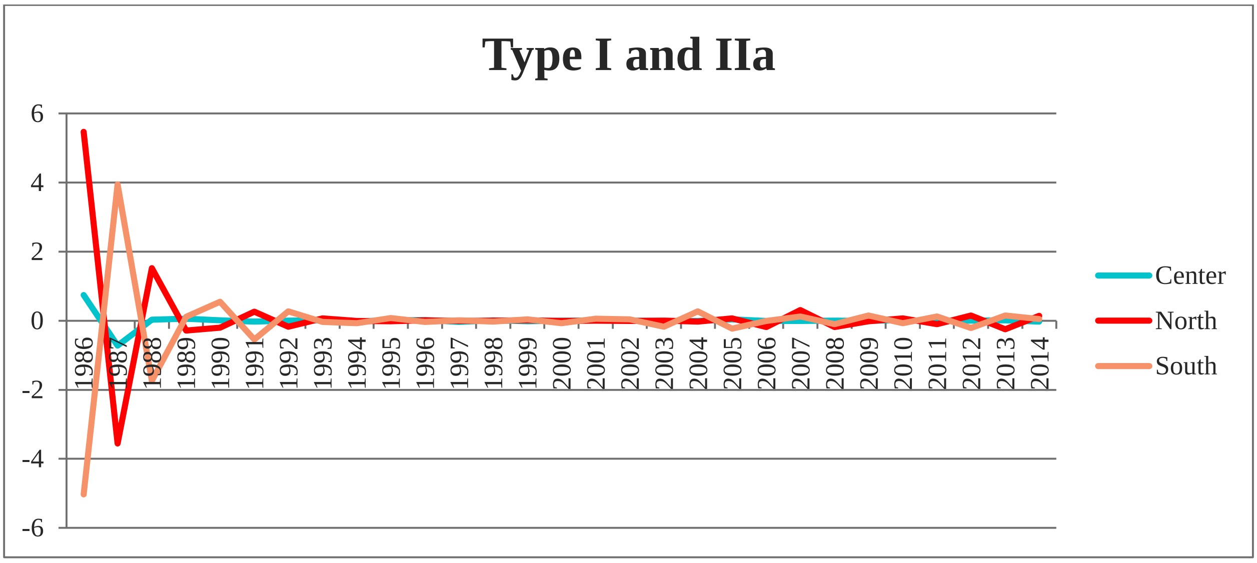

Figure 2: Factor for Organized crime based on Banbura and Modugno (2014) -Center, North, South Factor levels

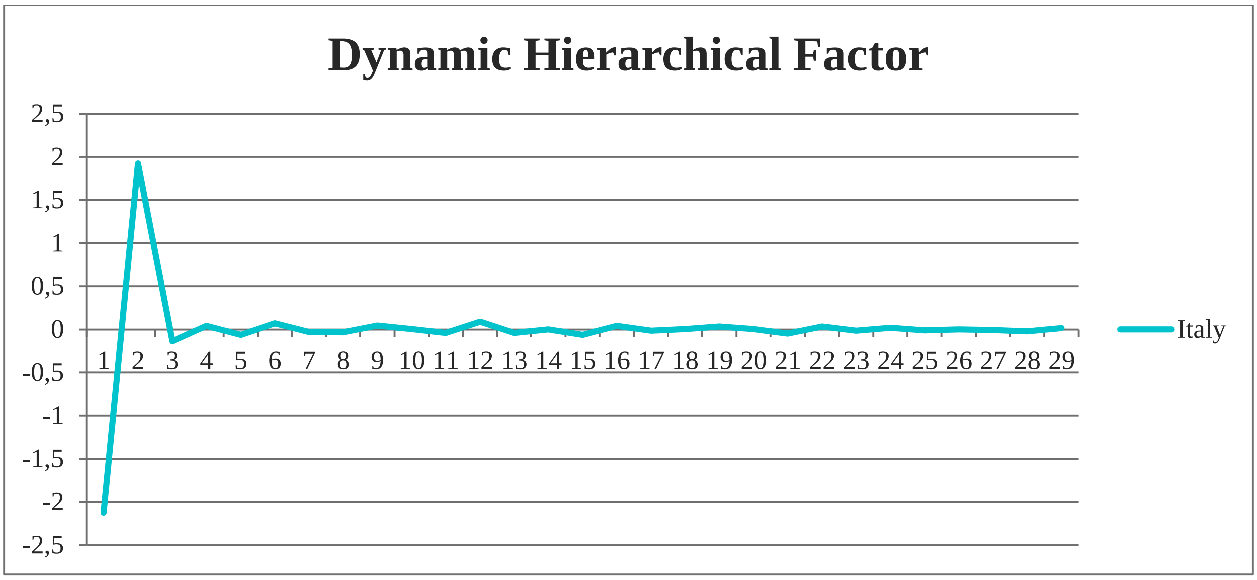

Figure 3: Factor for Organized crime based on Moench, Ng, and Potter (2011) - Italy

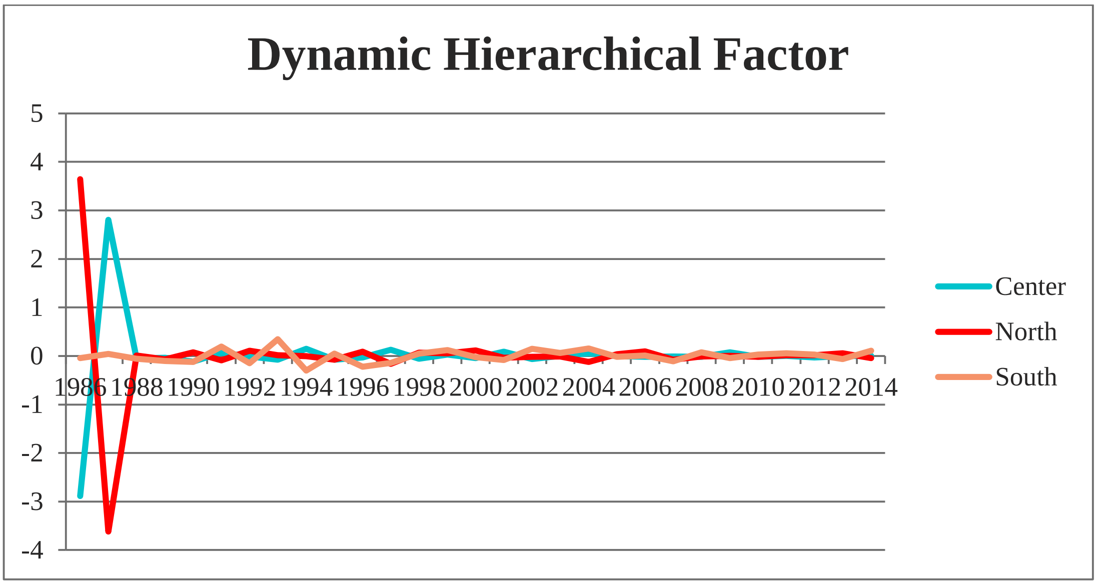

Figure 4: Factor for Organized crime based on Moench, Ng, and Potter (2011) – Macro Regions

Tables

Table 1: Summary statistics of 5-year averages of crime indices

	N	mean	min	max	sd	p25	p50	p75
A.3 Calderoni Mean Index (Type I,II)	120	30,378	9,150	61,367	11,533	21,623	28,965	37,839
A.4 Calderoni Rank Index (Type I,II)	120	52,500	14,813	79,750	13,018	44,125	54,063	60,594

Table 2: Factor Loadings computed following Banbura and Modugno (2014)

	Italy	Center	North	South
Bribery	-3,966	-31,1845	4,2941	-0,25721
Corruption for an action against official duties	-0,00708	-7,9145	-3,0894	-4,077
Criminal association	0,76512	-3,6345	-0,40594	0,50953
Drugs	0,031524	-0,94228	-0,00657	0,018737
Extortions	0,2422	-0,5157	-0,09812	0,21896
Homicide by Mafia	-0,16805	-0,73217	-0,1458	-0,16258
Mafia type Association	-1,0042	5,0596	0,74096	-0,73676
Money laundering	-2,4525	28,0857	-0,37736	-6,7294
Prostitution	-0,08054	0,43824	0,008533	-0,08575
Smuggling	-0,01232	-0,08777	0,012555	-0,00599
Threats	-2,5352	-9,1251	2,0677	-1,4177
Usury	0,68745	-21,5159	0,50321	-0,85019
Corruption in public acts	2,1015	-55,8672	-1,473	1,6959
Instigation to corrupt	-0,397	19,4413	-2,3362	-1,3267
Judicial corruption	9,4225	-26,3304	-5,5945	1,1776
Kidnapping for extortion purpose	-0,22488	-0,75744	0,068116	-0,22035

Table 3: Square of factor loadings computed following Banbura and Modugno (2014), as percentage of the total

	Italy	Centro	North	South
Bribery	12,7%	14,7%	25,5%	0,1%
Corruption for an actic	0,0%	0,9%	13,2%	23,2%
Criminal association	0,5%	0,2%	0,2%	0,4%
Drugs	0,0%	0,0%	0,0%	0,0%
Extortions	0,0%	0,0%	0,0%	0,1%
Homicide by Mafia	0,0%	0,0%	0,0%	0,0%
Mafia type Association	0,8%	0,4%	0,8%	0,8%
Money laundering	4,9%	11,9%	0,2%	63,2%
Prostitution	0,0%	0,0%	0,0%	0,0%
Smuggling	0,0%	0,0%	0,0%	0,0%
Threats	5,2%	1,3%	5,9%	2,8%
Usury	0,4%	7,0%	0,4%	1,0%
Corruption in public ac	3,6%	47,3%	3,0%	4,0%
Instigation to corrupt	0,1%	5,7%	7,5%	2,5%
Judicial corruption	71,7%	10,5%	43,3%	1,9%
Kidnapping for extorti	0,0%	0,0%	0,0%	0,1%

Table 4

		Share F	Share G	Share H	Share Z
Centro	Lazio	**0,10**	**0,09**	**0,10**	**0,71**
	Marche	**0,08**	**0,07**	**0,13**	**0,72**
	Toscana	**0,10**	**0,09**	**0,09**	**0,72**
	Umbria	**0,19**	**0,16**	**0,10**	**0,55**
Nord	Emilia Romagna	**0,17**	**0,07**	**0,07**	**0,69**
	Friuli Venezia Giulia	**0,10**	**0,04**	**0,17**	**0,69**
	Liguria	**0,21**	**0,09**	**0,10**	**0,61**
	Lombardia	**0,20**	**0,09**	**0,03**	**0,68**
	Piemonte	**0,20**	**0,08**	**0,06**	**0,66**
	Trentino Alto Adige	**0,17**	**0,07**	**0,06**	**0,70**
	Valle D'Aosta	**0,15**	**0,06**	**0,09**	**0,70**
	Veneto	**0,19**	**0,08**	**0,06**	**0,66**
Sud	Abruzzo	**0,02**	**0,13**	**0,13**	**0,71**
	Basilicata	**0,00**	**0,02**	**0,25**	**0,72**
	Calabria	**0,03**	**0,14**	**0,15**	**0,69**
	Campania	**0,01**	**0,07**	**0,22**	**0,70**
	Molise	**0,00**	**0,02**	**0,22**	**0,75**
	Puglia	**0,01**	**0,03**	**0,22**	**0,75**
	Sardegna	**0,01**	**0,04**	**0,23**	**0,72**
	Sicilia	**0,01**	**0,06**	**0,21**	**0,72**

ShareF, ShareG, ShareH, ShareZ denote the average variance share across all variables in the block due to aggregate, block-level, subblock-level and idiosyncratic shocks respective

Table 5: Summary statistics of inequality indices

**Variable**	**Obs.**	**mean**	**sd**	**min**	**max**
**A. Net income - Households**					
Gini Index	120	0,287	0,037	0,165	0,396
Atkinson inequality index	120	0,265	0,066	0,095	0,43
P90/P10	120	3,733	0,814	2,111	7,087
P75/P25	120	1,92	0,192	1,567	2,568
P90/P50	120	1,896	0,24	1,405	2,677
P75/P50	120	1,382	0,087	1,224	1,628
**B. Total Consumption - Households**					
Gini Index	118	0,251	0,029	0,138	0,327
Atkinson inequality index	118	0,186	0,036	0,062	0,296
P90/P10	118	3,084	0,419	1,961	4,82
P75/P25	118	1,765	0,125	1,35	2,107
P90/P50	118	1,799	0,146	1,366	2,26
P75/P50	118	1,335	0,053	1,152	1,432

Table 6: Summary statistics for the other covariates

Variable	N	mean	sd	min	max
Gross fixed capital formation	120	0,006	0,040	-0,119	0,078
Compensation of employees	120	0,007	0,019	-0,065	0,050
Primary Education level	58	47,978	7,477	32,500	61,500
Secondary level	58	38,886	4,909	29,56	48,110
Total active population	120	0,005	0,007	-0,02	0,018
Total hours worked	120	0,000	0,011	-0,03	0,030
Economic activity rate	76	53,797	5,570	43,500	63,750
Participation rate in education and training	58	6,06069	1,187057	3,76	9,47
Long-term unemployment rate	71	5,991	4,398	0,780	17,800
Employment	120	0,003	0,010	-0,024	0,028

Table 7: Dynamic panel regressions of Mafia Rank Index and Mafia Mean Index - alternative income inequality indices

	Mafia Rank Index						Mafia Mean Index					
Dependent variable	(1)	(2)	(3)	(4)	(5)	(6)	(7)	(8)	(9)	(10)	(11)	(12)
Regressors												
Lagged Mafia Index	0.60**	0.72***	0.72***	0.74***	0.72***	0.69***	0.87***	0.96***	0.88***	0.95***	0.95***	0.91***
	(0.27)	(0.17)	(0.06)	(0.20)	(0.09)	(0.10)	(0.08)	(0.08)	(0.06)	(0.07)	(0.09)	(0.09)
Gini	45.96						76.51					
	(61.63)						(56.72)					
Atkinson		2.10						33.16				
		(19.49)						(25.84)				
P90/P10			2.47**						3.53***			
			(0.90)						(1.04)			
P75/P25				8.19						19.70**		
				(7.37)						(8.08)		
P90/P50					8.44*						19.80**	
					(4.20)						(8.50)	
P75/P50						17.78**						48.77***
						(8.13)						(16.61)
period3	6.78***	7.56***	6.36***	7.11***	6.45***	7.51***	-0.68	-0.98	-1.08	-0.06	-0.49	0.68
	(1.67)	(1.82)	(1.46)	(1.55)	(1.38)	(1.04)	(0.98)	(1.34)	(1.06)	(1.17)	(1.05)	(1.18)
period4	11.55***	11.48***	10.88***	11.32***	10.07***	11.18***	0.42	0.08	0.77	2.07*	0.64	1.37
	(1.57)	(1.95)	(1.56)	(1.79)	(1.73)	(1.57)	(0.87)	(0.94)	(1.01)	(1.01)	(1.08)	(1.05)
period5	4.85*	4.18	3.85**	3.86*	4.27*	4.64***	0.55	-0.02	0.96	1.22	2.08	1.41
	(2.67)	(2.64)	(1.74)	(2.20)	(2.18)	(1.47)	(2.66)	(2.54)	(2.73)	(2.73)	(2.69)	(2.73)
period6	5.65***	5.44**	4.43**	5.05***	4.93***	6.21***	-0.11	-1.87	-1.01	-0.04	1.12	1.06
	(1.55)	(2.04)	(1.57)	(1.37)	(1.51)	(1.39)	(1.45)	(2.31)	(1.39)	(1.28)	(1.37)	(1.38)
Constant	-6.55	2.46	-5.71	-13.01	-11.58	-20.20*	-15.51	-6.46	-7.02	-35.74**	-35.22*	-63.34**
	(11.44)	(4.88)	(3.59)	(9.30)	(7.07)	(9.81)	(18.42)	(10.30)	(6.28)	(16.38)	(17.73)	(25.81)
Observations	95	95	95	95	95	95	95	95	95	95	95	95
Number of Regions	19	19	19	19	19	19	19	19	19	19	19	19
Hansen test p-value	0.818	0.730	0.925	0.761	0.986	0.749	0.540	0.387	0.446	0.555	0.554	0.511

This tables presents dynamic panel regressions of Mafia Rank Index and Mafia Mean Index based on Calderoni (2011) on alternative income inequality indices. All results are based on the baseline sample of 5-year averages. All models control for fixed effects and time period effects. Estimation is based on system GMM (Blundell and Bond (1998) using instruments up to the 4th lag. Each cell reports the coefficient estimates and robust standard errors in the parenthesis. ***, **, and * denote significance of the regression coefficient at 1%, 5%, and 10%.

Table 8: Dynamic panel regressions of Mafia Rank Index and Mafia Mean Index - alternative consumption inequality indices

	Mafia Rank Index						Mafia Mean Index					
Dependent variable	(1)	(2)	(3)	(4)	(5)	(6)	(7)	(8)	(9)	(10)	(11)	(12)
Regressors												
Lagged Mafia Index	0.80***	0.46***	0.63***	0.75***	0.73***	0.74***	0.91***	0.99***	1.04***	0.98***	0.93**	0.94***
	(0.09)	(0.15)	(0.16)	(0.12)	(0.10)	(0.13)	(0.20)	(0.20)	(0.17)	(0.17)	(0.33)	(0.18)
Gini	41.41						179.06**					
	(42.08)						(71.75)					
Atkinson		82.54*						151.78**				
		(42.77)						(61.62)				
P90/P10			5.65						13.85***			
			(5.74)						(4.51)			
P75/P25				10.26						33.27		
				(14.35)						(19.90)		
P90/P50					2.72						50.19	
					(6.86)						(30.88)	
P75/P50						53.59**						98.06**
						(24.95)						(37.02)
period3	8.30***	6.66***	6.97***	8.28***	7.61***	6.09***	1.62	1.11	0.48	0.42	-0.39	-0.91
	(1.35)	(1.72)	(1.50)	(1.42)	(1.21)	(1.34)	(2.20)	(2.47)	(2.12)	(1.72)	(2.10)	(1.80)
period4	11.94***	10.19***	10.99***	11.98***	11.41***	9.82***	1.51	1.72	-0.29	0.11	0.87	-0.53
	(2.25)	(2.63)	(1.97)	(2.66)	(2.38)	(1.75)	(2.10)	(2.39)	(2.67)	(2.13)	(2.09)	(1.86)
period5	4.38*	7.88***	5.51*	4.67	4.24*	3.33	3.17	4.58	3.98	1.11	4.83	0.47
	(2.35)	(2.61)	(2.67)	(2.72)	(2.07)	(2.21)	(4.70)	(4.27)	(4.71)	(4.51)	(4.64)	(3.68)
period6	5.61***	6.94***	6.00***	5.71**	5.50***	5.36***	2.57	1.44	0.22	0.57	3.64	1.81
	(1.71)	(1.47)	(1.64)	(2.00)	(1.76)	(1.50)	(2.41)	(2.78)	(2.18)	(2.57)	(3.33)	(1.78)
Constant	-9.86	-5.05	-11.31	-16.27	-1.94	-68.27**	-42.68	-30.40	-45.61**	-58.36	-88.77	-128.52**
	(11.01)	(11.76)	(15.53)	(25.23)	(12.39)	(32.19)	(25.42)	(19.12)	(19.59)	(40.67)	(71.94)	(57.21)
Observations	95	95	95	95	95	95	95	95	95	95	95	95
Number of Regions	19	19	19	19	19	19	19	19	19	19	19	19
Hansen test p-value	0.983	0.187	0.979	0.986	0.968	0.998	0.103	0.0729	0.0866	0.0571	0.169	0.232

This tables presents dynamic panel regressions of Mafia Rank Index and Mafia Mean Index based on Calderoni (2011) on alternative consumption inequality indices. All results are based on the baseline sample of 5-year averages. All models control for fixed effects and time period effects. Estimation is based on system GMM (Blundell and Bond (1998) using instruments up to the 4th lag. Each cell reports the coefficient estimates and robust standard errors in the parenthesis. ***, **, and * denote significance of the regression coefficient at 1%, 5%, and 10%.

Table 9: Dynamic panel regressions of Mafia Rank Index and Mafia Mean Index - alternative inequality indices

Dependent variable	Mafia Rank Index						Mafia Mean Index					
	(1)	(2)	(3)	(4)	(5)	(6)	(7)	(8)	(9)	(10)	(11)	(12)
Regressors												
Lagged Mafia Index	0.42**	0.42**	0.35	0.76***	0.50	0.47	0.86***	0.85***	0.92***	0.84***	0.92***	0.84***
	(0.17)	(0.15)	(0.25)	(0.08)	(0.31)	(0.29)	(0.08)	(0.09)	(0.16)	(0.09)	(0.08)	(0.07)
Gini	78.50						32.85					
	(72.39)						(46.96)					
Lagged Gini	41.35						70.80**					
	(31.44)						(33.06)					
Atkinson		57.76						20.39				
		(60.21)						(41.88)				
Lagged Atkinson		48.23*						53.51*				
		(26.09)						(29.15)				
P90/P10			1.25						15.28***			
			(5.22)						(4.30)			
Lagged P90/P10			3.81*						-2.84			
			(2.13)						(3.46)			
P75/P25				-3.80						3.53		
				(13.20)						(15.78)		
Lagged P75/P25				20.19*						14.98*		
				(11.46)						(8.27)		
P90/P50					0.32						21.79**	
					(12.23)						(9.80)	
Lagged P90/P50					13.74*						1.97	
					(7.27)						(8.22)	
P75/P50						31.88						86.02*
						(90.24)						(41.14)
Lagged P75/P50						5.63						-7.93
						(43.36)						(26.16)
period3	5.55**	5.09***	5.00***	5.37**	6.00***	5.48**	-1.43	-1.28	1.05	-0.89	0.06	-1.28
	(2.11)	(1.45)	(1.57)	(2.19)	(1.44)	(2.08)	(1.16)	(1.24)	(1.54)	(1.23)	(1.08)	(1.37)
period4	10.05***	9.98***	10.75***	8.70***	9.72***	10.42***	-0.97	-0.85	1.03	-0.59	-0.06	-0.71
	(1.97)	(1.83)	(1.49)	(1.96)	(2.64)	(2.16)	(1.10)	(1.23)	(2.19)	(1.05)	(1.45)	(1.81)
period5	7.22**	7.15**	6.45**	1.48	4.12	5.84**	-0.47	-0.33	4.16	-0.57	2.31	1.70
	(3.10)	(2.87)	(2.97)	(2.24)	(4.51)	(2.42)	(3.08)	(3.07)	(3.18)	(2.43)	(2.79)	(3.07)
period6	7.85***	7.37***	7.49***	4.04**	6.91**	6.67***	0.54	0.37	1.11	0.20	2.27	0.85
	(1.87)	(1.71)	(1.77)	(1.74)	(3.01)	(1.69)	(1.87)	(1.76)	(1.23)	(1.56)	(1.82)	(1.61)
Constant	-17.60	-6.92	-1.11	-24.51	-15.19	-39.62	-18.61	-5.43	-35.72**	-23.77	-39.73**	-96.18**
	(19.88)	(13.03)	(16.68)	(20.99)	(30.31)	(73.91)	(14.26)	(10.20)	(15.23)	(25.63)	(14.84)	(37.50)
Observations	95	95	95	95	95	95	95	95	95	95	95	95
Number of Regions	19	19	19	19	19	19	19	19	19	19	19	19
Hansen test p-value	0.360	0.405	0.518	0.675	0.549	0.399	0.966	0.961	0.344	0.981	0.910	0.989

This tables presents dynamic panel regressions of Mafia Rank Index and Mafia Mean Index based on Calderoni (2011) on alternative inequality indices. All results are based on the baseline sample of 5-year averages. All models control for fixed effects and time period effects. Estimation is based on system GMM (Blundell and Bond (1998) using instruments up to the 4th lag. Each cell reports the coefficient estimates and robust standard errors in the parenthesis. ***, **, and * denote significance of the regression coefficient at 1%, 5%, and 10%.

Table 10: Dynamic panel regressions of the Mean Mafia Index - P90/P10 ratio and other determinants

Dependent variable	Mafia Rank Index								Mafia Mean Index							
	(1)	(2)	(3)	(4)	(5)	(6)	(7)	(8)	(9)	(10)	(11)	(12)	(13)	(14)	(15)	(16)
Regressors																
Lagged Mafia Index	0.93***	0.92***	0.79***	0.92***	0.67***	0.90***	0.85***	0.75***	0.62***	0.69***	0.62***	0.79***	0.76***	0.68***	0.72***	0.60***
	(0.11)	(0.08)	(0.11)	(0.06)	(0.10)	(0.06)	(0.09)	(0.13)	(0.12)	(0.07)	(0.10)	(0.06)	(0.09)	(0.07)	(0.06)	(0.11)
P90/P10	7.53	1.94	-0.76	-3.34	7.43	-0.97	-5.16	-0.67	0.50	-1.90	-4.09	-3.87	-3.82	-1.47	-2.14	-4.65
	(8.77)	(6.28)	(7.26)	(4.76)	(6.39)	(4.44)	(3.37)	(7.20)	(5.38)	(3.88)	(4.53)	(2.90)	(5.08)	(2.60)	(3.94)	(4.91)
Lagged P90/P10	10.94	10.42**	11.34**	9.03***	17.27***	7.26**	5.14	12.55***	7.69	7.13*	9.50**	5.56	8.49**	6.68**	7.20	9.19**
	(6.69)	(3.87)	(4.26)	(2.48)	(4.65)	(3.13)	(3.79)	(4.04)	(4.47)	(4.09)	(4.48)	(3.38)	(3.89)	(3.15)	(4.31)	(3.48)
Economic activity rate		-0.63***	-0.47	-0.58***	-1.74***	-0.60***	-0.78***	-0.48		-0.16	-0.26	-0.19*	-0.20	-0.17	-0.15	-0.17
		(0.19)	(0.34)	(0.17)	(0.38)	(0.15)	(0.18)	(0.42)		(0.12)	(0.20)	(0.10)	(0.19)	(0.12)	(0.14)	(0.15)
Secondary Education level			-0.39								0.07					
			(0.58)								(0.48)					
Compensation of employees				32.89								-0.80				
				(63.32)								(34.26)				
Long-run unemployment					-3.09***								-0.05			
					(0.56)								(0.52)			
Gross fixed capital formation						34.43								22.63		
						(37.34)								(25.70)		
Total hours worked							-136.27								154.66	
							(153.37)								(105.33)	
Part. in education and training								-1.60								-0.73
								(3.29)								(1.55)
period3	-1.61	-2.87	0.00	-0.92	6.41**	-3.59	-0.20	0.00	4.99**	2.77	0.00	4.26**	4.54*	1.48	1.94	0.00
	(2.57)	(3.11)	(0.00)	(2.50)	(2.46)	(2.94)	(2.73)	(0.00)	(1.93)	(1.73)	(0.00)	(1.60)	(2.23)	(2.06)	(1.71)	(0.00)
period4	-1.37	-1.60	-6.60**	-0.10	2.48	-2.25	1.63	0.00	8.91***	6.23**	2.60	7.36***	7.23***	5.03**	4.93*	1.15
	(2.06)	(3.56)	(2.48)	(3.06)	(2.10)	(3.04)	(2.96)	(0.00)	(1.79)	(2.36)	(2.46)	(2.11)	(1.87)	(2.10)	(2.44)	(2.78)
period5	0.24		-4.45					2.77	2.94		-4.02**					-4.45**
	(4.95)		(2.89)					(4.06)	(3.36)		(1.40)					(1.75)
period6	2.07	2.78	0.00	3.97	11.95***	3.64	0.77	7.24	5.86**	3.30*	0.00	3.33**	4.24***	3.97**	5.32**	0.00
	(2.53)	(2.52)	(0.00)	(2.30)	(2.81)	(2.75)	(3.28)	(5.18)	(2.05)	(1.76)	(0.00)	(1.31)	(1.41)	(1.65)	(2.17)	(0.00)
Constant	-53.54**	-0.25	22.97	16.54	45.97	17.77	49.22*	8.20	-17.45	0.70	8.70	9.59	1.26	2.00	-0.38	15.09
	(24.53)	(30.68)	(46.48)	(19.34)	(35.64)	(20.83)	(27.06)	(36.97)	(14.26)	(18.03)	(33.12)	(15.41)	(19.06)	(14.87)	(21.30)	(25.66)
Observations	95	72	55	72	70	72	72	55	95	72	55	72	70	72	72	55
Number of Regions	19	19	19	19	19	19	19	19	19	19	19	19	19	19	19	19
Hansen test p-value	0.439	0.424	0.323	0.901	0.970	0,925	0,926	0,358	0.633	0.565	0.167	0.919	0.600	0.981	0.944	0.411

This table presents dynamic panel regressions of the Mean Mafia Index, which is the Mafia Rank Index based on Calderoni (2011), on P90/P10 ratio and other determinants. All results are based on the baseline sample of 5-year averages. All models control for fixed effects and time period effects. Each cell reports the coefficient estimates and robust standard errors in the parenthesis. All regression models include fixed effects and time effects. Estimation is based on system GMM (Blundell and Bond (1998) using instruments up to the 4th lag. ***, **, and * denote significance of the regression coefficient at 1%, 5%, and 10%.

Table 11: Regressions of two latent factors on alternative inequality of consumption indices

Dependent variable	Hierarchical Dynamic Factor Analysis						EM algorithm					
	(1)	(2)	(3)	(4)	(5)	(6)	(7)	(8)	(9)	(10)	(11)	(12)
Regressors												
Lagged Mafia Index	-0.43	-0.41	-0.37	-0.37*	-0.32*	-0.26*	-0.09*	-0.11	-0.16*	-0.12	-0.11	-0.12**
	(0.25)	(0.25)	(0.32)	(0.19)	(0.17)	(0.15)	(0.05)	(0.08)	(0.08)	(0.08)	(0.07)	(0.05)
ΔGini	1.42**						2.08*					
	(0.64)						(1.02)					
ΔAtkinson		1.16**						1.51*				
		(0.53)						(0.77)				
ΔP90/P10			0.15*						0.14**			
			(0.08)						(0.07)			
ΔP75/P25				0.08						0.23		
				(0.16)						(0.26)		
ΔP90/P50					0.34**						0.24***	
					(0.14)						(0.07)	
ΔP75/P50						-0.52						1.18
						(0.40)						(1.19)
period3	0.01	0.00	-0.00	-0.03*	-0.00	-0.04	0.06	0.09	0.07	0.02	0.03	0.03
	(0.02)	(0.02)	(0.03)	(0.02)	(0.03)	(0.02)	(0.04)	(0.06)	(0.05)	(0.07)	(0.04)	(0.08)
period4	0.01	0.01	0.00	-0.02	0.01	-0.03	0.04	0.06	0.04	0.01	0.02	-0.01
	(0.03)	(0.04)	(0.04)	(0.03)	(0.04)	(0.03)	(0.05)	(0.07)	(0.05)	(0.07)	(0.06)	(0.07)
period5	0.03	0.03	0.04	-0.02	0.04	-0.05***	0.09	0.10	0.10*	0.05	0.06	0.06
	(0.04)	(0.04)	(0.06)	(0.02)	(0.04)	(0.02)	(0.05)	(0.06)	(0.05)	(0.07)	(0.04)	(0.08)
period6	0.01	0.00	-0.01	-0.01	0.02	-0.02	0.03	0.02	0.01	-0.02	0.01	0.00
	(0.03)	(0.02)	(0.03)	(0.02)	(0.03)	(0.02)	(0.03)	(0.03)	(0.04)	(0.04)	(0.03)	(0.04)
Constant	-0.02	-0.01	-0.01	0.01	-0.01	0.03*	-0.05	-0.06	-0.05	-0.01	-0.03	-0.02
	(0.02)	(0.02)	(0.03)	(0.01)	(0.02)	(0.01)	(0.04)	(0.05)	(0.04)	(0.05)	(0.04)	(0.06)
Observations	95	95	95	95	95	95	95	95	95	95	95	95
Number of Regions	19	19	19	19	19	19	19	19	19	19	19	19
Hansen test p-value	0.280	0.297	0.574	0.445	0.553	0.307	0.624	0.219	0.987	0.210	0.404	0.461

The tables present regressions of two latent factors on alternative inequality of consumption indices. In Panel A, we have the results of a factor constructed using a hierarchical dynamic factor methodology, while in Panel B we show the results of a dynamic factor estimated by using a modified Expectation Maximisation (EM) algorithm. Given that the latent factors are constructed on transformed data to endure stationarity (growth rates) we also transform the regressors into first differences, too. All results are based on the baseline sample of 5-year averages. Each cell reports the coefficient estimates and robust standard errors in the parenthesis. All regressions control for fixed effects and time period effects. ***, **, and * denote significance of the regression coefficient at 1%, 5%, and 10%.

Table 12: Dynamic panel regressions of the Mafia Factor Index on P90/P10 ratio and other determinants

Dependent variable	Mafia Factor Index							
	(1)	(2)	(3)	(4)	(5)	(6)	(7)	(8)
Regressors								
Lagged Mafia Index	-0.16*	-0.48***	-0.48***	-0.22**	-0.47***	-0.13	-0.13*	-0.43*
	(0.08)	(0.16)	(0.05)	(0.08)	(0.11)	(0.08)	(0.07)	(0.25)
ΔP90/P10	0.14**	0.07*	0.02	0.10**	0.07**	0.10*	0.12**	0.05
	(0.07)	(0.04)	(0.04)	(0.04)	(0.03)	(0.05)	(0.05)	(0.07)
ΔEconomic activity rate		-0.03						
		(0.03)						
ΔSecondary Education level			-0.00					
			(0.02)					
ΔCompensation of employees				-0.08				
				(0.70)				
ΔLong-run unemployment					-0.02			
					(0.02)			
ΔGross fixed capital formation						-0.52		
						(0.41)		
ΔTotal hours worked							0.03	
							(1.91)	
ΔPart. in education and training								-0.02
								(0.09)
period3	0.07	0.00	0.00	0.06	0.00	0.11**	0.05	0.00
	(0.05)	(0.00)	(0.00)	(0.06)	(0.00)	(0.05)	(0.08)	(0.00)
period4	0.04	0.00	0.00	0.01	0.00	0.05	0.04	0.00
	(0.05)	(0.00)	(0.00)	(0.05)	(0.00)	(0.04)	(0.05)	(0.00)
period5	0.10*	0.03	0.02	0.07	0.03	0.09*	0.08*	0.00
	(0.05)	(0.05)	(0.05)	(0.04)	(0.04)	(0.04)	(0.04)	(0.00)
period6	0.01	-0.06	-0.02	-0.02	0.05	0.02	0.00	-0.06
	(0.04)	(0.05)	(0.05)	(0.03)	(0.09)	(0.02)	(0.03)	(0.11)
Constant	-0.05	0.04	0.00	-0.02	-0.03	-0.07**	-0.05	0.04
	(0.04)	(0.03)	(0.00)	(0.04)	(0.05)	(0.03)	(0.04)	(0.13)
Observations	95	53	36	95	51	95	95	36
Number of Regions	19	19	19	19	19	19	19	19
Hansen test p-value	0.987	0.688	0.564	1.000	0.635	1.000	1.000	0.838

This table presents dynamic panel regressions of the Mafia Factor Index on P90/P10 ratio and other determinants. All results are based on the baseline sample of 5-year averages. All models control for fixed effects and time period effects. Given that the latent factors are constructed on transformed data to endure stationarity (growth rates) we also transform the regressors into first differences, too. Each cell reports the coefficient estimates and robust standard errors in the parenthesis. All regression models include fixed effects and time effects. Estimation is based on system GMM (Blundell and Bond (1998) using instruments up to the 4th lag. ***, **, and * denote significance of the regression coefficient at 1%, 5%, and 10%

Table 13: Summary statistics on socio-economic mobility and OC measures at provincial level

Variable	N	mean	min	max	sd	p25	p50	p75
**A. Organized Crime Index**								
**A.3 Calderoni Mean Index (Type IIa)**	95	2,003	0,453	100	10,163	0,820	0,970	1,051
**A.4 Calderoni Rank Index (Type IIa)**	95	50,526	15,855	93,816	13,005	42,039	50,724	59,474
**B. Social Mobility**								
**B.1 Absolute Upward Mobility: Expected Rank**	95	0,463	0,371	0,625	0,059	0,410	0,457	0,523
**B.2 Absolute Upward Mobility: Q1Q5**	95	0,131	0,042	0,389	0,064	0,077	0,112	0,188
**B.3 Relative Mobility**	95	0,168	0,095	0,222	0,028	0,148	0,169	0,190

Table 14: Cross-sectional regressions of three alternative socio-economic mobility measures

Dependent variable	Mafia Mean Index (Type IIa)			Mafia Rank Index (Type IIa)		
Regressors	(7)	(8)	(9)	(10)	(11)	(12)
Absolute Upward Mobility: Expected Rank	-60.35***			-53.19**		
	-9,776			-20,72		
Absolute Upward Mobility: Q1Q5		-50.32***			-39.19**	
		-9,324			-18,56	
Relative Mobility			116.7***			179.2***
			-23,62			-42,37
Constant	48.81***	27.42***	1,25	75.18***	55.65***	20.44***
	-4,778	-1,471	-3,901	-9,849	-2,842	-7,253
Observations	95	95	95	95	95	95
R-squared	0,269	0,218	0,235	0,064	0,041	0,17

The table presents cross-sectional regressions of three alternative socio-economic mobility measures. All results are based on the baseline sample of 5-year averages. Each cell reports the coefficient estimates and robust standard errors in the parenthesis. ***, **, and * denote significance of the regression coefficient at 1%, 5%, and 10%.

Table 15: Cross-sectional regressions (1)

Dependent variable	Mafia Mean Index						
Regressors	(1)	(2)	(3)	(4)	(5)	(6)	(7)
Absolute Upward Mobility: Expected Rank	-35.06**	-42.90**	-41.25**	-38.57**	-42.77**	-44.09**	-47.97*
	(17.03)	(16.94)	(17.38)	(19.29)	(17.68)	(16.80)	(26.64)
Economic activity rate	-0.524**	-0.376*	-0,205	-0.330*	-0,193	-0,211	-0,201
	(0.218)	(0.207)	(0.174)	(0.196)	(0.173)	(0.170)	(0.181)
Primary Education level	-0.380**						
	(0.154)						
Secondary Education level		0,355					
		(0.232)					
Compensation of employees			-18,89				
			(41.57)				
Employment				164.8**			
				(75.34)			
Gross fixed capital formation					-17,71		
					(20.16)		
Total hours worked						-124,7	
						(84.73)	
Nord							0,928
							(2.246)
Constant	82.39***	46.82***	51.10***	58.27***	50.60***	51.32***	53.79***
	(13.86)	(6.382)	(5.614)	(6.595)	(5.489)	(5.480)	(7.850)
Observations	95	95	95	92	95	95	95
R-squared	0,327	0,301	0,284	0,284	0,287	0,303	0,284

The table presents cross-sectional regressions. All results are based on the baseline sample of 5-year averages. Each cell reports the coefficient estimates and robust standard errors in the

parenthesis. ***, **, and * denote significance of the regression coefficient at 1%, 5%, and 10%.

Table 16: Cross-sectional regressions (2)

Dependent variable	Mafia Mean Index						
Regressors	(1)	(2)	(3)	(4)	(5)	(6)	(7)
Absolute Upward Mobility: Q1Q5	-22.64**	-26.69**	-25.22**	-25.35*	-25.87**	-27.93**	-22,88
	(11.32)	(11.43)	(11.83)	(15.19)	(11.82)	(11.60)	(13.81)
Economic activity rate	-0.638***	-0.500***	-0.331**	-0.436***	-0.326**	-0.339**	-0.332**
	(0.180)	(0.178)	(0.134)	(0.157)	(0.132)	(0.131)	(0.136)
Primary Education level	-0.399**						
	(0.153)						
Secondary Education level		0,347					
		(0.231)					
Compensation of employees			-14,94				
			(41.43)				
Employment				166.8**			
				(75.80)			
Gross fixed capital formation					-14,01		
					(19.68)		
Total hours worked						-123,4	
						(83.71)	
Nord							-0,296
							(1.780)
Constant	76.21***	37.63***	42.30***	49.59***	41.67***	41.64***	42.36***
	(14.75)	(7.295)	(6.690)	(7.991)	(6.505)	(6.426)	(6.889)
Observations	95	95	95	92	95	95	95
R-squared	0,32	0,288	0,271	0,275	0,273	0,291	0,27

The table presents cross-sectional regressions. All results are based on the baseline sample of 5-year averages. Each cell reports the coefficient estimates and robust standard errors in the parenthesis. ***, **, and * denote significance of the regression coefficient at 1%, 5%, and 10%.

Table 17: Cross-sectional regressions (4)

Dependent variable	Mafia Mean Index (Type IIa)						
Regressors	(1)	(2)	(3)	(4)	(5)	(6)	(7)
Relative Mobility	59.58**	68.66**	72.71**	69.59**	81.25***	75.93***	64.63**
	(28.38)	(27.51)	(27.91)	(31.12)	(28.36)	(26.86)	(27.25)
Economic activity rate	-0.606***	-0.473**	-0.311***	-0.427***	-0.287***	-0.330***	-0.260**
	(0.189)	(0.185)	(0.113)	(0.125)	(0.109)	(0.112)	(0.124)
Primary Education level	-0.361**						
	(0.167)						
Secondary Education level		0,295					
		(0.239)					
Compensation of employees			-28,05				
			(41.35)				
Employment				164.9**			
				(68.36)			
Gross fixed capital formation					-30,5		
					(20.35)		
Total hours worked						-131	
						(80.99)	
Nord							-1,478
							(1.545)
Constant	59.89***	23.27**	25.51**	34.04***	21.75**	24.63**	25.01***
	(20.26)	(9.017)	(9.999)	(11.02)	(9.851)	(9.667)	(9.440)
Observations	95	95	95	92	95	95	95
R-squared	0,335	0,308	0,299	0,3	0,308	0,319	0,302

The table presents cross-sectional regressions. All results are based on the baseline sample of 5-year averages. Each cell reports the coefficient estimates and robust standard errors in the parenthesis. ***, **, and * denote significance of the regression coefficient at 1%, 5%, and 10%.

Table 18: Cross-sectional regressions (5)

Dependent variable	Mafia Rank Index						
Regressors	(1)	(2)	(3)	(4)	(5)	(6)	(7)
Relative Mobility	151.5***	163.3***	161.8***	189.8***	170.4***	174.9***	179.4***
	(53.89)	(53.84)	(54.22)	(57.37)	(55.22)	(53.24)	(53.92)
Economic activity rate	-0,479	-0,365	-0,107	-0,344	-0,0845	-0,118	-0,301
	(0.332)	(0.328)	(0.193)	(0.210)	(0.186)	(0.186)	(0.229)
Primary Education level	-0,475						
	(0.364)						
Secondary Education level		0,506					
		(0.533)					
Compensation of employees			18,45				
			(85.92)				
Employment				406.0***			
				(147.4)			
Gross fixed capital formation					-15,81		
					(40.67)		
Total hours worked						-207,5	
						(145.3)	
Nord							4,972
							(3.109)
Constant	71.94*	22,33	29,48	40.55**	25,9	24,97	34.91*
	(36.30)	(18.93)	(18.28)	(19.85)	(18.16)	(17.78)	(18.40)
Observations	95	95	95	92	95	95	95
R-squared	0,193	0,184	0,172	0,236	0,173	0,19	0,194

The table presents cross-sectional regressions. All results are based on the baseline sample of 5-year averages. Each cell reports the coefficient estimates and robust standard errors in the parenthesis. ***, **, and * denote significance of the regression coefficient at 1%, 5%, and 10%.

Bibliography

Acciari, P., Polo, A. Violante, G. (2017), *"And yet, it moves': Intergenerational mobility in Italy"*. Mimeo NYU.

Arellano M., and S. Bond (1991), *"Some tests of specification for panel data: Monte Carlo evidence and an application to employment equations"*. Review of Economic Studies 58: 277–97.

Bańbura M., and Modugno M. (2014), *"Maximum Likelihood Estimation Of Factor Models On Datasets With Arbitrary Pattern Of Missing Data"*. Journal of Applied Econometrics, John Wiley & Sons, Ltd., vol. 29(1), pages 133-160, 01.

Barone, R., & Masciandaro, D. (2011). Organized crime, money laundering and legal economy: theory and simulations. *European journal of law and economics*, 32(1), 115-142.

Blundell R., and S. Bond (1998), *"Initial conditions and moment restrictions in dynamic panel data models"*. Journal of Econometrics 87: 11–143.

Calderoni F. (2011), *"Where is the Mafia in Italy? Measuring the presence of the Mafia across Italian Provinces"*. Article published in Global Crime.

Chetty, R., Hendren, N., Kline, P., & Saez, E. (2014). Where is the land of opportunity? The geography of intergenerational mobility in the United States. *The Quarterly Journal of Economics*, 129(4), 1553-1623.

Corak, M., & Heisz, A. (1999). "The intergenerational earnings and income mobility of Canadian men: Evidence from longitudinal income tax data". *Journal of Human Resources*, 504-533.

Cowell, F. (2011). *Measuring Inequality* - Oxford University Press

DIA (2016), *"Relazione del Ministro dell'interno al Parlamento sull'attività svolta e sui risultati conseguiti dalla Direzione Investigativa Antimafia"*, Roma. Available at: http://direzioneinvestigativaantimafia.interno.gov.it/page/relazioni_semestrali.html

Fioroni, T., Lavezzi, AM and Trovato, G. (2017), "Organized Crime, Corruption and Poverty Traps", in progress

Fajnzylber, P., Lederman, D., & Loayza, N. (2002), "Inequality and violent crime". The journal of Law and Economics, 45(1), 1-39.

Franchetti, L. (1877). Condizioni politiche e amministrative della Sicilia (Vol. 1). G. Barbera.

Gambetta, D., & Reuter, P. (1995). Conspiracy among the many: the mafia in legitimate industries. In The Economic Dimensions of Crime (pp. 99-120). Palgrave Macmillan UK.

Güell, M., Pellizzari, M., Pica, G., & Rodríguez Mora, J. V. (2017), "Correlating social mobility and economic outcomes". Forthcoming Economic Journal.

Hertz, T. (2006). Understanding mobility in America. Center for American Progress Discussion Paper.

Istat (2010), "Distribuzione del reddito e condizioni di vita in Italia".

Lavezzi, A. M. (2014). Organised crime and the economy: a framework for policy prescriptions. Global Crime, 15(1-2), 164-190.

Jappelli, T., & Pistaferri, L. (2010). Does consumption inequality track income inequality in Italy?. Review of Economic Dynamics, 13(1), 133-153.

Jong-Sung, Y., & Khagram, S. (2005), "A comparative study of inequality and corruption". American Sociological Review, 70(1), 136-157.

Macours, K. (2010), "Increasing inequality and civil conflict in Nepal". Oxford Economic Papers, 63(1), 1-26.

Moench E., Ng S. and Potter S. (2013), "Dynamic Hierarchical Models". Review of Economics and Statistics, 95:5.

Riccardi, M., Soriani, C., & Giampietri, V. (2016). 8 Mafia infiltration in legitimate companies in Italy. Organised Crime in European Businesses, 119.

Roodman, D. (2009). How to do xtabond2: An introduction to difference and system GMM in Stata. Stata Journal, 9(1), 86-136.

Stock, J. H., & Watson, M. (2011), "Dynamic factor models". Oxford Handbook on Economic Forecasting.

Sunde, U., Cervellati, M., & Fortunato, P. (2008), "Are all democracies equally good? The role of interactions between political environment and inequality for rule of law". Economics Letters, 99(3), 552-556

Appendix

Table A1: variables' definition and sources

Variable	Source
Bribery	Concussione - Offences reported by the police to the prosecution service (2003-2015) - SDI (acronym for System of Investigation)
Compensation of employees	Cambridge Econometrics (€2005m)
Corruption for an act against Official Duties	Corruzione per un atto contrario ai doveri d'ufficio - Offences reported by the police to the prosecution service (2003-2015) - SDI (acronym for System of Investigation)
Corruption in public acts	Corruzione per un atto d'ufficio - Offences reported by the police to the prosecution service (2003-2015) - SDI (acronym for System of Investigation)
Criminal_association	Associazione a delinquere - reported by the police forces to the judicial authority (1985-2015) - ISTAT
Drugs	Produzione, commercio, ecc. di stupefacenti - reported by the police forces to the judicial authority (1985-2015) - ISTAT
Economic Activity rate - total	Percentage of total population, both employed and unemployed, that constitutes the manpower supply of the labor market - Eurostat
Employment	Cambridge Econometrics (000s)
Extortions	Estorsioni - reported by the police forces to the judicial authority (1985-2015) - ISTAT
Gross fixed capital formation	Cambridge Econometrics (€2005m)
Homicide_Mafia	omicidi per motivi di mafia, camorra o 'ndrangheta - Murder committed by Mafia reported by the police forces to the judicial authority (1985-2015) - ISTAT
Homicide_terrorism	Omicidi a scopo terroristico (art. 28- C.P.) - Murders committed by Terrorists reported by the police forces to the judicial authority (1985-2015) - ISTAT
Inequality - Net Income	Gini and other inequality measures for Net Income - Bank of Italy (SHIW)
Inequality - Total consumption	Gini and other inequality measures for total consumption- Bank of Italy (SHIW)
Instigation to corrupt (Istigazione alla corruzione)	Instigation to corrupt - Offences reported by the police to the prosecution service (2003-2015) - SDI (acronym for System of Investigation)
Intergenerational educational mobility	Mobility mesuares based on the change of educational status between father and children - Bank of Italy (SHIW)
Intergenerational occupational mobility	Mobility mesuares based on the change of occupational status between father and children - Bank of Italy (SHIW)
Judicial corruption	Corruzione in atti giudiziari - Judicial corruption - Offences reported by the police to the prosecution service (2003-2015) - SDI (acronym for System of Investigation)
Kidnappings for extortion purpose	Sequestro di persona a scopo estorsivo - kidnappings for extortion purpose reported by the police forces to the judicial authority (1985-2003) - ISTAT
Kidnappings for terrorist purposes	Sequestro di persona a scopo terroristico - kidnappings for terrorist purposes reported by the police forces to the judicial authority (1985-2003) - ISTAT
Long-term uneployment rate	Eurostat
Mafia_type_Association	Associazione a delinquere di stampo mafioso - Mafia criminal association reported by the police forces to the judicial authority (1985-2015) - ISTAT
Money laundering	Riciclaggio di denaro - Money laundering - Offences reported by the police to the prosecution service (2003-2015) - SDI (acronym for System of Investigation)
Participation rate in education and training	Participation rate in education and training (last 4 weeks). Population aged 25-64 - Eurostat
Primary Education level	Percentage of people aged 25-64 with less than primary, primary and lower secondary education attainment (levels 0-2) - Eurostat
Prostitution	Prostituzione - Prostitution reported by the police forces to the judicial authority (1985-2015) - ISTAT
Robberies	Rapina - Robberies reported by the police forces to the judicial authority (1985-2015) - ISTAT
Secondary Education level	Percentage of people aged 25-64 with upper secondary and post-secondary non-tertiary education attainment (levels 3 and 4) - Eurostat
Smuggling	Contrabbando - Smuggling reported by the police forces to the judicial authority (1985-2015) - ISTAT
Theft	Furto - Theft reported by the police forces to the judicial authority (1985-2015) - ISTAT
Threats	Minacce - Threats - Offences reported by the police to the prosecution service (2003-2015) - SDI (acronym for System of Investigation)
Total hours worked	Cambridge Econometrics (millions)
Usury	Usura - Usury - Offences reported by the police to the prosecution service (2003-2015) - SDI (acronym for System of Investigation)

CHAPTER 7: Policy Makers' Contribution (UCSC-Transcrime)

Table of contents

1 INTRODUCTION 358
2 SUMMARY OF POLICY MAKERS' CONTRIBUTIONS 359
FEEDFORWARD PROCESS..359
FEEDBACK PROCESS 359
3 CONCLUSIONS...359

1 Introduction

UCSC-Transcrime promoted the interaction between policy makers and PROTON researchers to target on the needs and requirements of policy authorities at different levels. For doing so, scientific and policy partners collaborated to establish a common approach to the analysis of the factors leading to recruitment in OC networks, and to integrate policy makers' perspectives into the final outputs of the studies.

In particular, representatives of Brå, DPPS, EUCPN, EUROPOL, UNODC and WODC received the outlines of activities and project proposals (feedforward process, M3-M4) and the draft reports on innovative studies (feedback process M11-M12) of WP1, and they provided multidisciplinary feedback and recommendations on task T1.3 to T1.6.

Moreover, policy makers commented and discussed the reports on preliminary finding of WP1 during the First Consortium meeting, hosted by The Hebrew University of Jerusalem on October 16th-17th 2017. Afterwards, they submitted a summary of their recommendations.

Feedback and feedforward comments were gathered in a single document by the project Coordinator and they were shared with WP1's tasks leaders. The following section briefly summarises policy makers' main contributions to the innovative studies of WP1; the original comments can be consulted in the abovementioned document.

2 Summary of policy makers' contributions

Feedforward process

Brå (comments submitted on December 22nd 2016):

- Researchers should evaluate the applicability of results on mafia members and mafia bosses to the European context;
- Definitions of networks for organised crime and terrorism should be separated due to the peculiarities of each phenomenon.

WODC (comments submitted on December 22nd 2016)

- Partners should focus on current knowledge on the possibility to mitigate determinant factors leading to OC involvement.

UCSC-Transcrime sent clarifications to policy makers. Pertinent comments and recommendations were considered and integrated into the research.

Feedback process

EUROPOL (summary of recommendations submitted on October 26th 2017):

- Studies on the criminal career paths should be translated into observations/questions over the recruitment phase;
- Researchers should reconsider the applicability of the ABM to a wider, not country- or region-specific context;
- The dynamic nature of OC should be considered and captured in the ABM.

Brå (summary of recommendations submitted on November 13th 2017):

- Researchers should emphasize how the results will contribute to the ABM and to the data simulations;
- The results from the different studies should not be generalised as a complete overview on recruitment factors of OC.

EUCPN (summary of recommendations submitted on November 13th 2017):

- Researchers should create different or separated ABMs instead of a unique model, due to the distinctive features of OC and terrorism;
- The ABMs should be applicable to the European context.

WODC *(summary of recommendations submitted on November 17th 2017):*

- Some preliminary results of WP1 may not reflect the Netherlands socio-economic context.

3 Conclusions

Task 1.7 established a systematic process of interaction between policy and scientific partners and allowed policy makers' views and requirements to be properly considered and integrated into D1.1.
