# Supplementary material for: Grassroots community actors leading the way in the prevention of youth violent radicalization
Source: PLoS One. 2020 Oct 12;15(10):e0239897. doi: 10.1371/journal.pone.0239897 (PMC7549796; doi:10.1371/journal.pone.0239897)
Supplement: S2 File — (PDF) [file pone.0239897.s002.pdf]

PROTON

Modelling the processes leading
to organised crime and terrorist networks

Work package 2– Final report

December 2017

WP2 - OC Networks: Social, Psychological & Economic Factors

Tasks T2.1 (Michael Wolfowicz, Yael Litmanovitz, Badi Hasisi, David Wesiburd), T2.2, T2.3 (Badi Hasisi, Tomer Carmel, David Weisburd, Michael Wolfowicz), T2.4 (Justice Tankabe), T2.5 (Gary LaFree, Michael Jensen, Patrick A. James Aaron Safer-Lichtenstein,), T2.6 (Friedrich Lösel, Sonja King, Doris Bender, Irina Jugl) T2.7 (Frank Weerman, Vanja Ljujic, Fabienne Thijs, Inge Versteegt, Fatima El Bouk

Authors: HUJI, UB-CREA, UCAM, USMF, FAU, VU/VUmc and WODC

Modelling the PRocesses leading to Organised crime and TerrOrist Networks
FCT-16-2015

This project has received funding from the European Union's Horizon 2020 research and innovation programme under grant agreement N° 699824.

Technical References

Project Acronym	PROTON
Project Title	Modelling the PRocesses leading to Organised crime and TerrOrist Networks
Project Coordinator	Ernesto Savona<br>Università Cattolica del Sacro Cuore<br>ernesto.savona@unicatt.it
Project Duration	October 2016 – September 2019 (36 months)

Deliverable No.	
Dissemination level [1]	PP
Work Package	WP2
Task	T2.1, T2.2, T2.3, T2.4, T2.5, T2.6, T2.7, T2.8
Lead beneficiary	2 (HUJI), 7 (VU/VUmc), 8 (UB-CREA), 9 (UCAM), 10 (FAU),11 (USMF), 21 (WODC)
Contributing beneficiary(ies)	
Due date of deliverable	31 December 2017
Actual submission date	31 December 2017

[1] PU = Public
PP = Restricted to other programme participants (including the Commission Services)
RE = Restricted to a group specified by the consortium (including the Commission Services)
CO = Confidential, only for members of the consortium (including the Commission Services)

This project has received funding from the European Union's Horizon 2020 research and innovation programme under grant agreement N° 699824.

Security Advisory Board Review	
Comments	No security sensitivity issues
Recommended Distribution	It is therefore suitable for public dissemination as required by the DoA
Date	21 December 2017

This project has received funding from the European Union's Horizon 2020 research and innovation programme under grant agreement N° 699824.

0 Summary

This deliverable report presents the findings and final reports of all tasks contained within WP2. The report centres around T2.1, the first quantitative systematic review of risk factors for radicalization and recruitment to terrorism. This task includes the results of T2.3, T2.5 and T2.7. The report continues with T2.4, a systematic review of protective factors. The report follows with the individual reports for T2.3, T2.5, T2.6 and T2.7. This report will also focus on the progress and development of the collaborative approach and structure of WP2 in achieving its objectives for months 1-18. The report also discusses how the tasks complemented each other and will filter in to the development of WP5. The report also provides some ideas on possible policy implications stemming from the results of WP2.

Table of contents

0 SUMMARY 4
1 INTRODUCTION TO WP2 8
2 T2.1 SYSTEMATIC REVIEW OF RISK FACTORS OF RADICALIZATION AND RECRUITMENT TO TERRORISM 12
2.1 INTRODUCTION 12
2.2 WHAT IS RADICALIZATION? OPERATIONALIZING EU POLICY 14
2.3 RISK FACTORS 17
2.3.1 CHALLENGES: CONTRADICTORY EVIDENCE 18
2.4 THE IMPORTANCE AND TIMING OF T2.1 20
2.5 OBJECTIVES 21
2.6 INCLUSION AND EXCLUSION CRITERIA 22
2.6.1 POPULATION 22
2.6.2 TYPES OF SETTINGS 24
2.6.3 SEARCH STRATEGY 26
2.7 INITIAL SEARCHES, REVIEW AND QUERYING 28
2.7.1 TRENDS IN RESEARCH, APPROACHES AND FINDINGS 30
2.7.2 TYPES OF STUDIES: 31
2.8 META ANALYSIS 34
2.8.1 AGE AND SEX 36
2.8.2 MARITAL STATUS 38
2.8.3 INDIVIDUAL SOCIO-ECONOMIC STATUS 39
2.8.4 EMPLOYMENT STATUS 41
2.8.5 EDUCATION 44
2.8.6 IMMIGRANT STATUS 47
2.8.7 POOR INTEGRATION 50

This project has received funding from the European Union's Horizon 2020 research and innovation programme under grant agreement N° 699824.

2.8.8 Institutional trust and legitimacy 51
2.8.9 Differential associations 52
2.8.10 Mental health 55
2.8.11 Racism experience 58
2.8.12 Experienced violence 60
2.8.13 Social media usage 62
2.8.14 Criminal history 65
2.8.15 Collective relative deprivation 69
2.8.16 Politics 71
2.8.17 West vs. Islam 73
2.8.18 Religious identity 75
2.8.19 Religiosity 77
2.8.20 Frequency of attendance at place of worship 79
2.8.21 prayer frequency 82
2.8.22 Religious fundamentalism 83
2.8.23 Other risk factors: 86
2.8.24 quantifying the two pyramid model 87
2.9 Heterogeneity and publication bias 92
2.10 Conclusions 94
2.11 List of included studies 97

3 T2.6- - Protective factors against extremism and violent radicalization: A systematic review of research 106

3.1 Abstract 106
3.2 Introduction 106
3.3 Method 109
3.3.1 Eligibility criteria 109
3.4 Results 110
3.4.1 Descriptive data of included studies 110
3.5 Discussion 116
References 122

4 Individual risk factor studies (T2.3, T2.5, T2.7) 130

4.1 T2.3 Recidivism of security offenders: Examining the crime-terror nexus 130
4.1.1 Summary 130
4.1.2 Introduction 131
4.1.3 Recidivism among terrorists and the crime-terror nexus 132
4.1.4 Methodology 135
4.1.5 Results 138
4.1.6 Discussion 142
4.1.7 Conclusions 144
4.1.8 References 145
4.2 T2.4: Counter-terrorism legitimacy and recruitment into terrorism 146
4.2.1 Summary 146
4.2.2 Summary of preliminary findings: 147
4.2.3 BACKGROUND 147
4.2.4 METHODS 148
4.2.5 PRELIMINARY FINDINGS 149

This project has received funding from the European Union's Horizon 2020 research and innovation programme under grant agreement N° 699824.

4.2.6 COUNTER-TERRORISM AS RADICALISATION AND RECRUITMENT TOOL? 152
4.2.7 PLANS FOR QUANTITATIVE DATA 154
4.2.8 REFERENCES 155
4.3 T2.5 CORRELATES OF VIOLENT POLITICAL EXTREMISM IN THE UNITED STATES 156
4.3.1 SUMMARY 156
4.3.2 RESEARCH OBJECTIVE 157
4.3.3 INDIVIDUAL-LEVEL CORRELATES 159
4.3.4 PIRUS DATASET 163
4.3.5 METHODOLOGY 165
4.3.6 UNIVARIATE AND BIVARIATE RESULTS 165
4.3.7 MULTIVARIATE ANALYSIS 169
4.3.8 REFERENCES 172
4.4 T2.7: SOCIO-ECONOMIC BACKGROUND OF TERRORISM SUSPECTS IN EUROPE 180
4.4.1 SUMMARY AND CONCLUSION 180
4.4.2 THEORETICAL FRAMEWORK 181
4.4.3 EXPLORATORY STUDY USING OPEN SOURCES 181
4.4.4 QUANTITATIVE STUDY ON DUTCH TERRORIST SUSPECTS 182
4.4.5 QUALITATIVE STUDY ON DETAINED TERRORIST OFFENDERS 186
4.4.6 REFERENCES 188

5 CONCLUSIONS OF WP2 189

5.1 EU SPECIFIC CONTEXT 190

6 POLICY IMPLICATIONS 192

7 REPORT ON ETHICAL AND SECURITY IMPLICATIONS 196

INTRODUCTION 196

1. METHODOLOGY AND SCOPE 197

2. FINDINGS 203

3.1. LITERATURE REVIEW 203
2.2. RESULTS FROM THE FIELDWORK: ETHICAL AND SOCIETAL IMPACT 215

4. CONTRIBUTIONS TO INFORM THE OPERATIONALISATION OF THE INPUT FOR ABM SIMULATIONS 241

5. FINAL REMARKS 242

6. REFERENCES 244

ANNEX 1. FIELDWORK STUDY PARTICIPANTS 246

ANNEX 2. GUIDELINES FOR QUALITATIVE FIELDWORK 252

8 POLICY MAKERS' CONTRIBUTION 281

SUMMARY 281

This project has received funding from the European Union's Horizon 2020 research and innovation programme under grant agreement N° 699824.

CONCLUSIONS **283**

List of Tables

1 Included studies by type 31
2 Meta-analysis on "Age" 36
3 Meta analysis on "Marital status" 38
4 *Meta-analysis on "SES"* 39
5 Meta-analysis on "SES" moderated by region 40
6 Meta-analysis on "Employment" 41
7 Meta-analysis on "Employment" (beliefs group only) 42
8 Meta-analysis on "Employment" (Actions groups only) moderated by region 42
9 Meta-analysis on "Education" 44
10 Meta-analysis on "Education" (Beliefs group only) moderated by region 45
11 Meta-analysis on "Education" (Actions group only) moderated by region 46
12 Meta-analysis on "Immigrant status" 47
13 Meta-analysis on "Immigrant status" (Beliefs group only) moderated by region .. 48
14 Meta-analysis on "Poor integration" 50
15 Meta-analysis on "Institutional trust" 51
16 Meta-analysis on "Differential associations" 52
17 Meta-analysis on "Differential Associations" (Actions group only) 53
18 Meta-analysis on "Mental health" 56
19 Meta-analysis on "Experienced racism" (Beliefs group only) moderated by region 59
20 Meta-analysis on "Experienced violence" (Beliefs group only) 60
21 Meta-analysis on "Experienced violence" EU only 61
22 Meta-analysis on "Social media usage" (Beliefs group EU only) 63
23 Meta-analysis on "Criminal history" 66
24 Meta-analysis on "Religiosity" 77
25 Meta-analysis on "Religiosity" (Beliefs group only) 78
26 Meta-analysis on "Place of worship attendance" 80
27 Meta-analysis on "Place of worship attendance frequency" (Beliefs group only) moderated by region 81
28 Meta-analysis on "Prayer frequency" 82
29 Meta-analysis on "Religious fundamentalism" 83
30 Radicalization of belief pyramid 88
31 Radicalization of action pyramid 89
32 T2.1 risk factor matrix 91
Table 34 166
Table 35 168

This project has received funding from the European Union's Horizon 2020 research and innovation programme under grant agreement N° 699824.

1 Introduction to WP2

The primary objective of WP2 was to identify the social, psychological and economic factors leading to the radicalisation and recruitment of individuals to terrorism. The secondary objective of WP2 was to classify the identified factors in a way that would enable them to contribute to the definitions and construction of variables and rules for the outcomes of PROTON (WPs 4-5).

WP2 was led by HUJI and included 6 tasks, each led by a specific participant as well as a report on ethical and societal impacts. With this structure (see figure below), WP2's objective was to ensure a comprehensive coverage of the current knowledge while pushing beyond the existing knowledge with state-of-the-art studies in the field of radicalisation and recruitment to terrorism. Each of the studies, as well as the WP as a whole, were conceptualized and implemented, and altered as needed, in order to ensure that they would contribute effectively to the Agent Based Modelling (ABM) of processes of radicalization and recruitment in WP5. This posed a unique challenge which required that task leaders adjusted their research plans in order to conform to the same approach. This approach greatly improved the integrative and collaborative nature of the work package.

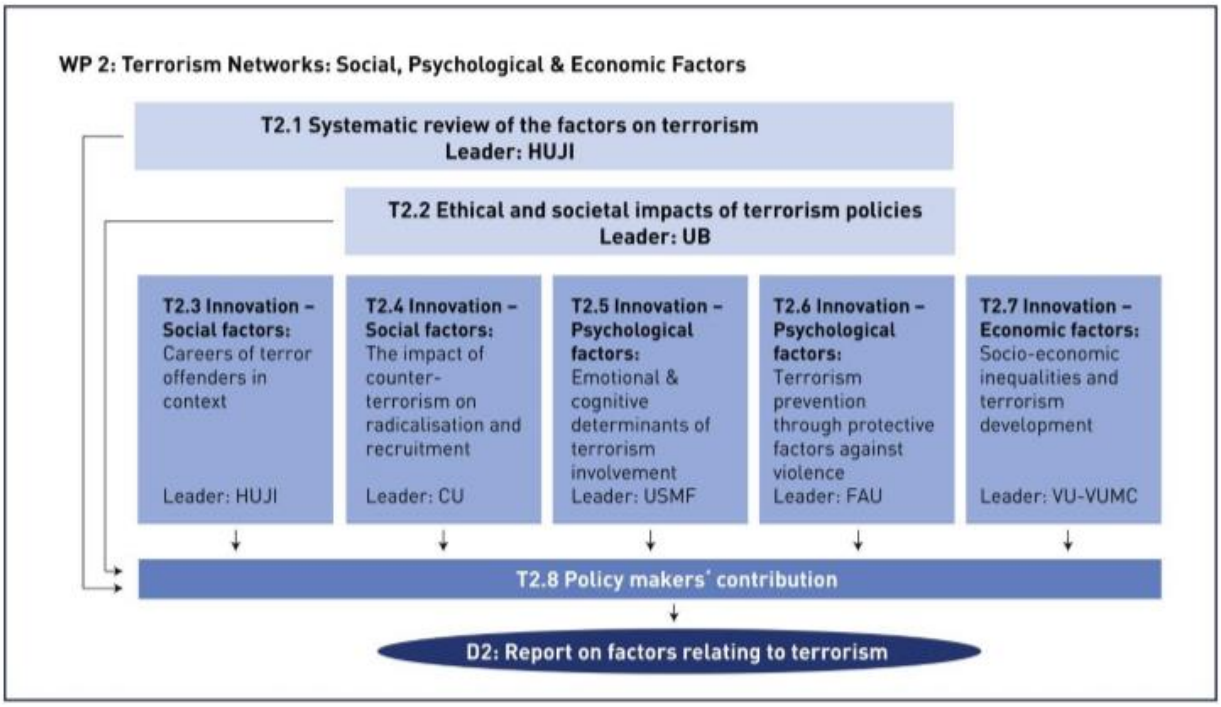

Figure 1 Original structure of WP2

This project has received funding from the European Union's Horizon 2020 research and innovation programme under grant agreement N° 699824.

This report's structure reflects the results of this approach by presenting the synthesis of the tasks as well as their individual and collective results. This report's is therefore centred around the findings of T2.1 (which incorporates the results of T2.3, T2.5, T2.7). The results of T2.1 are subsequently contrasted with the results of T2.6 in order to contextualize the crossover between risk and protective factors. The report proceeds with the individual reports of T2.3, T2.4, T2.5, T2.7, which each focus on specific risk factors and contexts. Each of the studies examines some of factors identified in T2.1. This structure provides a better contextualization of the results, conclusions, and policy implications.

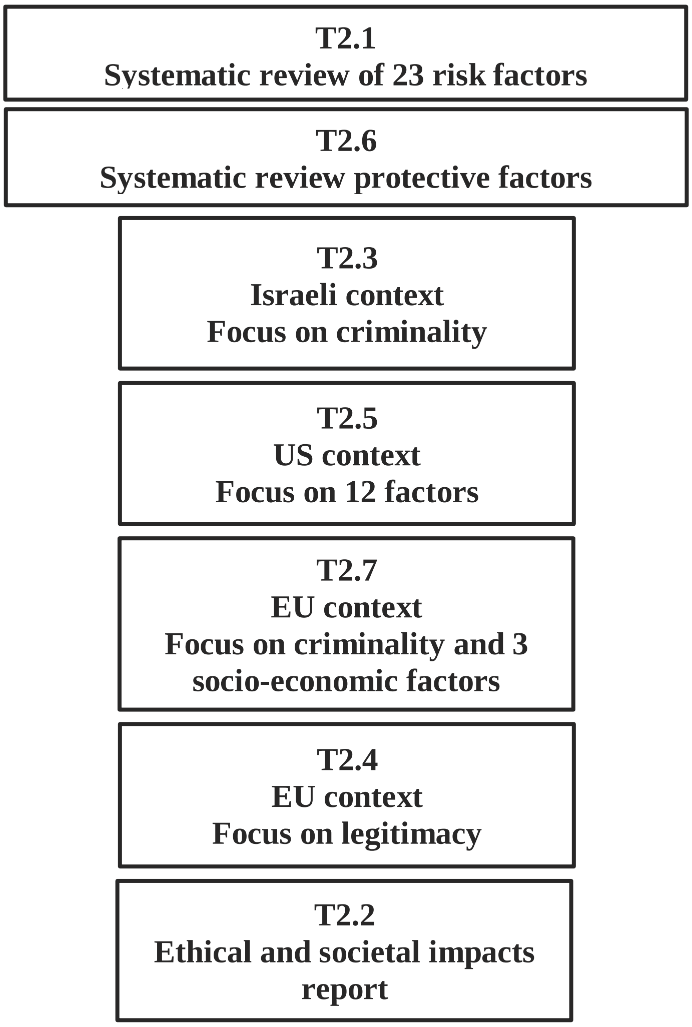

The results of WP2 have also fulfilled the original objectives of identifying and classifying risk factors for radicalization and recruitment and in providing actionable data for the development of WP5. The rules and inputs for the ABMs on terrorism will be directly derived from the Odds

This project has received funding from the European Union's Horizon 2020 research and innovation programme under grant agreement N° 699824.

Ratios provided by T2.1, which include the results of T2.5 and T2.7 as noted above. A risk matrix which summarizes the quantitative findings of T2.1 provides the basis for this (see section 2.8.24).

The tasks conducted in the framework of WP2 sought to go beyond the state-of-the-art by employing both quantitative and qualitative approaches to examine new and original data sets and examining a range of risk factors on a deeper level. To this end, each task focussed on a specific set of factors in a specific context. Additionally, while T2.1 demonstrates that much of the current evidence relates to radicalization, WP2 focussed more on the outcome of recruitment.

WP2's comparative approach

PROTON was conceptualized and designed to be oriented towards the EU context and policymakers in the framework of the Horizon2020 project. As such, WP2's goal was to identify factors of radicalization and recruitment that may be specific to the EU context. It is an axiom of scientific investigation that it is impossible to assess the bias in a sample by looking at the sample itself (Rosenbaum, 1987). It follows from this that it is not possible to identify factors that are unique to a specific context without having comparison groups from other contexts. Such an approach is already widely recognized in the study of health and violence related risk factors (Wilson et-al, 2011; Tsendsuren, Li & Liu, 2016). WP2 took the approach that since democratic states are similar in many fundamental ways, it makes methodological sense to compare between them (GTI, 2016; Shenderovich et al, 2016).

Most of the earlier terrorism research has already demonstrated that democracies are unique compared to non-democracies in the macro factors that predict terrorism (e.g. Piazza, 2004, 2006, 2008a, 2008b, 2009, 2010, 2011; Enders and Sandler 1992, 1999, 2000, 2004; Krueger and Laitin, 2003; Freytag, Krüger, Meierrieks & Schneider, 2011). However, little is known about the individual level factors as they have not been quantified in the way that macro socio-economic factors have been. In order to identify risk factors unique to the EU, WP2 included two tasks specific to the EU, and two additional tasks from other democratic contexts, namely the US and Israel. This theory led approach enabled comparisons to be made which led to WP2 being able to extrapolate those risk factors which may be uniquely significant to the EU context.

This project has received funding from the European Union's Horizon 2020 research and innovation programme under grant agreement N° 699824.

The issue of radicalization and recruitment as a research framework has been approached by the PROTON consortium from the point of view that ABM's represent the best possible chance of experimenting the effects of different anti-radicalization policies that seek to inhibit risk factors or improve protective factors. The meta-analysis conducted in T2.1 provides actionable data upon which distributions and rules can be built in to the ABMs. As such, this report focusses primarily on the summary statistics identified by T2.1.

This project has received funding from the European Union's Horizon 2020 research and innovation programme under grant agreement N° 699824.

2 T2.1 Systematic review of risk factors of radicalization and recruitment to terrorism

2.1 Introduction

Terrorism continues to be one of the most serious challenges in the world today, especially for democratic countries which have experienced recent increases in terrorism and the threat of terrorism. Not only has the frequency of terrorism events and related deaths increased, but there has been a growth in specific types of threats, such as home-grown and lone-wolf terrorism, and foreign fighters, particularly those returning (EUROPOL, 2017; European Commission, 2016; GTI, 2016; UN, 2015; Wright, 2016). In light of these issues, most democratic states have recognized that military power, intelligence and policing alone are unable to counter the multitude of threats posed by terrorism. Rather, an effective approach to combatting terrorism is one which balances active security with efforts to stymie those factors which lead to radicalization and recruitment to terrorism (EU, 2005, 2014; White House, 2011; OSCE, 2014). There has therefore been an increased focus on efforts aimed at counter-radicalization, de-radicalization, identification of at-risk individuals and prevention of recruitment. This approach has led to the development of a range of socio-economic interventions, community and education initiatives, and online interventions under the umbrella of counter violent extremism (CVE) policies. Such an approach seeks to tackle radicalization and recruitment in all their forms in order to curb the development of a new generation of terrorists and the threat that an increased prevalence of radicalized individuals increases the likelihood of terrorism (EU, 2015, 2016, 2017; Korn, 2016; Harris-Horgan et-al, 2016; OSCE, 2014).

Unfortunately, many of the current approaches, as well as specific policies and initiatives are not evidence based. The fact that there is little concrete information upon which policies and interventions can be developed, relying instead on assumptions, means that they are unlikely to have the desired impact (Davis, 2014; Christmann, 2012; Romaniuk, 2015; Mastroe & Szmania, 2016). For example, the EU 's counter-terrorism strategy focussed specifically on socio-economic interventions at home and abroad (EU, 2005), even though economic development has not been shown to impact on reducing terrorism (Berrebi, 2007; Reich, 2008). Additionally, the spillover effects of terrorism that such policies seek to prevent may not actually exist (LaFree et-al, 2017). In

This project has received funding from the European Union's Horizon 2020 research and innovation programme under grant agreement N° 699824.

the UK, the leading terror prevention strategy 'PREVENT' has been called a failure by former MI-5 chief Eliza Manningham-Buller (Holehouse, 2015). PREVENT was partly informed by one study on risk factors that specifically states that "It is not an actuarial measure of risk", and that "It must be followed up by longitudinal evaluation studies in order to empirically validate its theoretical bases". Despite admitting that it is merely intended to "provide and enhance practitioners' knowledge of factors that are theoretically linked to violent extremism", this study and others like it have provided the basis and framework for informing important policies (Cole et-al, 2013:2; Munton et-al, 2011).

The nature of the research on terrorism, radicalization and risk factors has lagged behind for a number of years (Sageman, 2014) and despite the growth in the quantity of such research, empirical studies still only accounts for a small percentage overall (Lum, Kennedy & Shirley, 2006; Christmann, 2012; Silke, 2008). Some researchers have pointed out that it is this lack of systematic investigation that has left policy makers to develop policies and strategies that are not only not evidence based but are based on purely theoretical assumptions (Victoroff, 2005; Neumann & Kleinmann, 2013). A systematic review and synthesis of the data on risk factors therefore marks an important first step to developing a better understanding of what are the individual micro-level risk factors of radicalization and recruitment to terrorism, and which of these are the most significant. Only once a better understanding of risk factors is developed can the development of more effective and scientifically based policies and interventions be achieved (Hawkins et al., 1998; Blum & Ireland, 2004; Piquero et al., 2009; Heath-Kelley, 2013).

This project has received funding from the European Union's Horizon 2020 research and innovation programme under grant agreement N° 699824.

2.2 What is radicalization? Operationalizing EU policy

The EU has defined radicalization as "the phenomenon of people embracing opinions, views and ideas which could lead to acts of terrorism" (EU, 2005), whereas recruitment is where an individual has somehow been convinced to act on these opinions and ideas and provide actual support for, facilitate or engage in violent actions (EU, 2014). In this regard, the EU defines recruitment as meaning "to solicit another person to commit or participate in the commission of a terrorist offence, or to join an association or group, for the purpose of contributing to the commission of one or more terrorist offences by the association or the group" (EU, 2005) and both "Recruitment to carry out terrorist offences" as well as actual "recruitment into a terrorist group" (EU, 2014). It follows that radicalization to action, is a natural developmental outcome of radicalization of belief, which occurs in some small percentage of the population that have radicalized beliefs. Radicalization to action, or recruitment to terrorism is ultimately fulfilled by:

the unlawful use of violence or threat of violence against persons, as well as serious damage or threats to property, critical infrastructure or systems, carried out by non-state actor organizations, members or supporters of such organizations, small groups or individuals who are motivated by religious, political, or other ideological beliefs, and aim to instil fear in and coerce governments or societies in pursuit of the furtherance, advancement or promotion of goals that are usually political, social, religious or ideological (PROTON).

Radicalization and recruitment to terrorism are certainly two different but inherently interrelated processes. A host of different risk factors may increase an individual's propensity for radicalization but not necessarily for recruitment. While not every recruited individual has necessarily been radicalized, most have (Horgan, 2009; ICCT, 2016). This means that radicalization is the single greatest risk factor for recruitment. In turn, many of the risk factors for both radicalization and recruitment are therefore likely to be the same, at least indirectly. However, other risk factors may be able to be used to differentiate between those who are radicalized and those who go on to be recruited (Horgan & Taylor, 2001). This is important in contexts like the EU, where as little as 1%

This project has received funding from the European Union's Horizon 2020 research and innovation programme under grant agreement N° 699824.

of Muslims for example may hold radical views (Rabasa & Benard, 2014). According to this logic model, from this small pool of individuals, an even smaller percent are at risk of recruitment.

The current literature on radicalization and recruitment to terrorism is characterized by the multiple, competing, and often contradicting theoretical frameworks that seek to explain pathways to radicalization and recruitment. Many of these frameworks explain specific types of terrorism, such as suicide bombings (Gill, 2007), or utilize only specific case studies of significance, such as that of the Hamburg cell to develop their models (Taarnby, 2005). Other models may only address radicalization and recruitment to a terror organization and therefore they do not address home-grown or lone wolf terrorism (Wiktorowicz, 2004). Still others have treated radicalization to terrorism in the same way as all political radicalization (Moskalenko, 2008), which can be criticized for many reasons. Most of these frameworks focus explicitly on individual or societal factors, and few have sought to incorporate both. Overall, none of these models have provided the type of integration described by Velhuis and Staun (2009) as needing both the "micro (individual) and macro (societal/cultural) examination of context-specific variables to explain causation". Such an approach could account for the different factors leading to different radicalization trajectories; these include trajectories of 'no radicalization', and radicalization without recruitment (Veldhuis & Staun, 2009; Borum, 2011; Bertelsen, 2016; Monahan, 2015; Borum, 2015).

Based on such appreciations, the research team adopted the two pyramid approach of McCauley and Moskalenko (2014, 2017) and which differentiates between radicalization of belief and radicalization of action. In this model, radicalization of action is only one step above radicalization of belief, although radicalization of belief is not a necessary factor for radicalization of action or behavior. Nevertheless, radicalization of belief is usually a strong risk factor for radicalization of action, meaning involvement in terrorism (Neumann, 2013; Khalil, 2014; Hafez & Mullins, 2015). Additionally, by being able to categorize different types of beliefs and actions, it may be easier to systemize and organize the evidence. For example, activists engage in essentially legal and non-violent political actions, however radicals engage in illegal and violent political actions. Similarly, terrorists are clearly differentiated by having carried out an illegal, violent act (Moskalenko and McCauley, 2009). These distinctions enable sub-group analysis that can differentiate the effect sizes of the typologies.

This project has received funding from the European Union's Horizon 2020 research and innovation programme under grant agreement N° 699824.

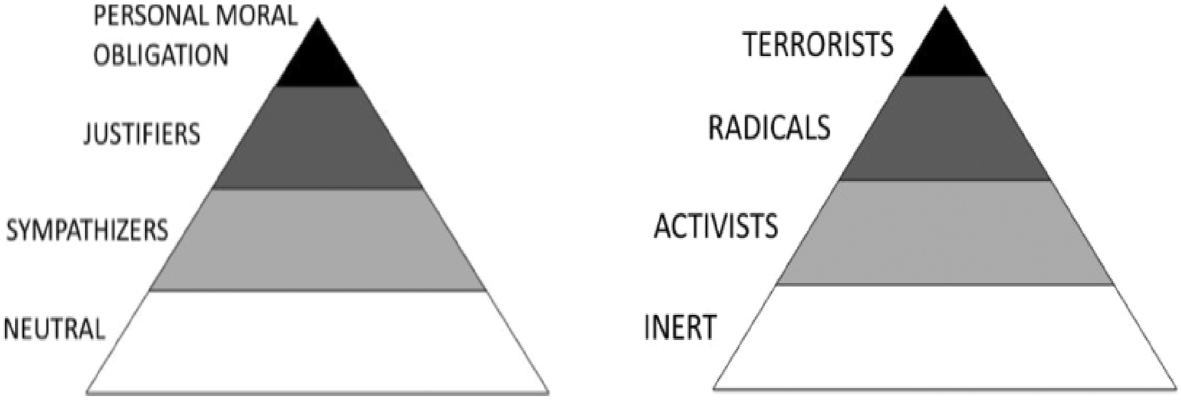

Figure 1. Opinion radicalization pyramid. **Figure 2.** Action radicalization pyramid.

Figure 2 The 'two pyramid' model

This project has received funding from the European Union's Horizon 2020 research and innovation programme under grant agreement N° 699824.

2.3 Risk factors

As stated above, while existing policies and strategies aim to tackle risk factors, there is often contradicting information about what the risk factors are and the extent to which they are significant. Within the current body of literature can be found a host of proposed risk factors, however the extent to which these risk factors have any degree of validation, or demonstrate statistical significance remains unknown (Bhui et-al, 2012; Coppock & McGovern, 2014).

Risk factors are used as predictors for involvement in many types of offending behaviours, such as gang involvement (Higginson et-al, 2016; Hill et-al, 1999) and general violent offending (Herenkohl et-al, 2000). They can also be used to identify differences between different types or categories of offending (Esbensen et-al, 2009; Horgan et-al, 2016), as well as recidivism (T2.3). As is the case with all types of offending, most individuals possessing even all the risk factors will never actually offend. Risk factors only increase the propensity of offending and do not predict offending as a given. However, risk factors can help to identify and differentiate the types of individuals who may be at a greater risk for offending behaviour (Shader, 2001; Kazden et-al, 1997; Murray & Thomson, 2010). By systematically collating and analysing risk factors, it is also possible to identify how their magnitude may be associated with different types of offending outcomes (Esbensen et-al, 2009; Horgan et-al, 2016). In the context of radicalization and recruitment to terrorism, a risk factor approach may be able to reconcile some of the differences between those acting on different radical doctrines (e.g. Islamist, right-wing, left-wing etc.) for which different factors may be relevant (Mohanan, 2015; Jensen et-al, 2016), as well as between different contexts. It has been noted that among violent offenders in general, there is great heterogeneity and attempts to approach the issue with "one-size-fits-all" solutions ought to be avoided (Widom, 2014).

A "risk factor" is a variable that a) statistically correlates with the outcome (in this case radicalization and/or recruitment to terrorism), and which b) precedes the outcome in time (Kraemer et-al, 1997; Farrington & Loeber, 2000). By defining risk factors in this way a distinction can also be made from 'causal risk factors', which are quite difficult to determine for processes such as radicalization and recruitment (Mohanan, 2015). This is especially the case in our field of study,

This project has received funding from the European Union's Horizon 2020 research and innovation programme under grant agreement N° 699824.

since researchers have mostly been prevented access to incarcerated terrorists and extremists (Mohanan, 2012, 2015). This has resulted in the current body of literature containing few validated risk factor measures; most of the identified risk factors remain as putative, or proposed. As such, in order for a study to have been included in the review (as will be described in the 'inclusion criteria' section), the outcome variable(s) must have directly represented or related to radicalization of belief or radicalization of action as per the definitions adopted by the review in the context of terrorism/violent extremism.

2.3.1 Challenges: contradictory evidence

In the course of the ever expanding research into causes of radicalization and recruitment to terrorism, a great deal of work has been done to examine the correlation with economic factors, both at the national, community and individual levels. With terrorism research having initially been the purview of political science, it was widely believed that individual depravation was a significant factor leading to radicalization and recruitment to terrorism. However, despite a plethora of research that sought to examine this correlation, many studies found evidence that indicated that poverty was not connected to terrorism (e.g. Krueger & Malečková, 2003; Piazza, 2006). Rather, it was illuminated that in general, and especially in democratic countries, most terrorists come from middle and upper-middle class economic settings (Russell & Miller, 1977; Livingstone, 1982; Berrebi, 2007). Additionally, those of the poorest settings appear the least likely to be involved in or support terrorism (Bhui, 2014; Lee, 2011). In this same context it has been regularly noted that the poorest countries and populations in the world, especially those in Africa, suffer little terrorism and produce few terrorists (Bloom, 2005; Clarke, & Newman, 2006; Gambetta, 2005; Hafez, 2006; Pape, 2005). Both the United States and the EU have devoted significant attention and resources to foreign economic development as part of their counter-terrorism strategies. However, there is significant doubt, based on empirical findings, that such development has any effect on terrorism (Berrebi, 2007; Reich, 2008). The Global Terrorism Index (GTI) (2016) indicates that correlations with economic status differ significantly between democratic and non-democratic countries as well. However, not all the evidence is in agreement on these issues, with some having found that whilst economic factors may not be relevant at the individual level, macro-level economic gaps and relative depravation may be (Coid et-al, 2016; Piazza, 2011; Freytag et-al, 2011; Mousseau, 2011).

This project has received funding from the European Union's Horizon 2020 research and innovation programme under grant agreement N° 699824.

Similarly, the evidence regarding psychological factors is quite mixed. Early notions that radicalized individuals and terrorists *must* be 'crazy', or that suicide bombers *must* be suffering from mental illness, have traditionally not been supported by the evidence. Several studies have claimed that most of the evidence points to an overwhelming 'normality' among terrorists as being the only psychological constant (Atran, 2003; Pape, 2005; Post et-al, 2009; Gill, 2012; Horgan, 2005; Kruglanski & Golec, 2004; Merari, 2004; Sageman, 2004). However, between different types of terrorism some differences can be found and some researchers have claimed that such assertions were made to early and before enough evidence had been gathered (Victoroff, 2006; Lankford, 2016). For example, many suicide bombers in Afghanistan sent by the Taliban were individuals suffering from mental illness (Sheehan, 2014); suicide bombers in Chechnya and Israel have also been found to suffer from psychological illness, sometimes as a result of traumatic incidents such as the loss of a relative (Speckhard & Akhmedova, 2006; Merari et-al, 2009). According to Sheehan (2014) there is strong evidence that many suicide terrorists are in fact suicidal, contrary to the prevailing conception. With respect to lone wolf terrorists, as many as 35% of attackers in Europe between 2000 and 2015 may have been suffering from some form of mental health issues (de Roy van Zuijdewijn & Bakker, 2016). Additionally, with the rise of the internet and social media as an important factor in radicalization and recruitment, other types of psychological conditions are now being examined as possibly relevant, such as the online disinhibition effect, deindividuation (Silke, 2010), and narcissistic personality disorder (Manne, 2014). In recent years, more studies have begun to identify some psychosomatic and mental health correlations with terrorist offending, support for extremism, and propensity for radicalization. Among a sample of foreign fighters from the Netherlands, a significant number were found to have had pre-existing mental health conditions, both diagnosed and undiagnosed (Weenink, 2015). As is the case with economic factors, the body of evidence on psychological factors needs to be better synthesized in order for a more grounded understanding to be developed.

This project has received funding from the European Union's Horizon 2020 research and innovation programme under grant agreement N° 699824.

2.4 The importance and timing of T2.1

As discussed, there are two dangers inherent in policies and strategies that may be based on misidentified risk factors. Namely, the failure to properly identify those most at risk, and the inclusion of those who pose an insignificant threat (Bertelsen, 2016; Scarcella et-al, 2016). Recently Scarcella et-al (2016) conducted a systematic review on methodologies used to perform risk assessments of individual radicalization. The focus of this review was on the different instruments and tools used to conduct risk assessments. The examination of actual risk factors was beyond the scope of this review, although the authors do note than in their review it was apparent that for many of the tools they examined, the risk factors included in them were not based on empirical evidence. They further noted that there was little transparency with regards to how and why specific risk factors were included or omitted. As an example, this review specifically pointed out that the ERG22+ tool, which was developed for and represents one of the main products of the UK's 'PREVENT' strategy, lacked transparency as to how their set of risk factors was developed. Indeed, ERG22+ has come under scrutiny for having resulted in the exact problems noted above (CAGE, 2016).

To date, only a few systematic reviews have been carried out with respect to the broader topic of terrorism. In the first review, Kennedy, Lum and Shirley (2006) reviewed counter-terrorism policies. It was also found that a 'rapid evidence review' conducted by Munton et-al (2011) for Office for Security and Counter-Terrorism, UK Home Office, examined only theoretical and qualitative studies. Christmann's (2012) review on 'pathways to terrorism', only had a small section devoted to "individual risk factors". The review notes the lack of an individual profile of terrorists but does so only in the context of its discussion, without referring to any quantitative evidence. More recently, the ICPC (2015) review of processes of radicalization in western countries was conducted. While this review examined empirical evidence, it did not examine risk factors specifically nor did it provide any quantitative synthesis that could be operationalized in a meaningful way.

This project has received funding from the European Union's Horizon 2020 research and innovation programme under grant agreement N° 699824.

2.5 Objectives

The primary objectives of this systematic review were to provide information that could help in answering important questions regarding the risk factors associated with radicalization and recruitment to terrorism. Namely, the primary objectives of this review were to identify and collate the different risk factors which are grounded in empirical research and for which there is evidence to support their characterization as risk factors, and for which there is evidence regarding the extent to which they are significant. As such, this review sought to explore and identify:

1) What are the social, economic, psychological and environmental risk factors associated with radicalization?

2) What are the social, economic, psychological and environmental risk factors associated with recruitment?

3) What are the shared and differentiating risk factors for the two outcomes of radicalization and recruitment?

4) To what extent are identified risk factors significant and what are their effect sizes?

5) What risk factors differentiate between different contexts and different ideologies?

In addition, the review's secondary objectives were to:

6) Provide guidance for a secondary systematic review that will focus on anti-radicalisation interventions that mitigate these risk factors.

7) Provide data and inputs in the form of effect sizes (such as risk ratios) to be used in the development of PROTONS's agent based modelling section.

This project has received funding from the European Union's Horizon 2020 research and innovation programme under grant agreement N° 699824.

2.6 Inclusion and exclusion criteria

The known literature is characterized by studies representing theoretical discussions or literature reviews, with only a small percentage of studies being empirically based (King & Taylor, 2011; Sageman, 2011; Christmann, 2015; Sikes, 2008). As such this review sought to extract only the most relevant studies by excluding those studies which are purely theoretical, provide theoretical models, or which represent literature reviews, opinion pieces, purely qualitative approaches and those studies which are based on purely descriptive statistics. As Jolliffe et al., (2012) explain, when conducting systematic reviews on risk factors—as opposed to interventions—there is a need, or at least a justification, for lowering the number of variables relating to the inclusion quality threshold. Nevertheless, only quantitative studies were considered for inclusion as earlier reviews relied almost exclusively on the type of excluded studies noted above.

While randomized controlled trials (RCT) would provide the best causal risk factor data, only very few studies exist which have employed such methods (e.g. Amjad & Wood, 2009; Dechesne, 2009). It has often been explained that the topics of terrorism, radicalization and recruitment inherently preclude the use of RCTs on account of security concerns (Laycock, 2012). The review therefore prioritized the second most ideal study designs as being quasi-experimental designs that may be based on case controlled analysis (e.g. Kruglanski et-al, 2017), longitudinal studies (e.g. Feddes et-al, 2015), and cross-sectional study designs (e.g. Bhui et-al, 2014, 2015; Coid et-al, 2016). With regards to these observational studies, including pre and post evaluation studies, in order to have been considered for inclusion, they must have demonstrated that attempts had been made to control for potential confounding factors by implementing multivariate regressions or other relevant procedures. For these types of studies, the review only included those studies which had an N of >50.

2.6.1 Population

In general, there are significant difficulties and challenges in studying populations of radicalized individuals and terrorists more specifically. Namely, access to such individuals has been very difficult for researchers to obtain (Mohanan, 2015). Following the definitions outlined in sections 2.2. and 2.3, this review needed to take a broader approach in order to ensure that the most relevant and

This project has received funding from the European Union's Horizon 2020 research and innovation programme under grant agreement N° 699824.

important studies would be identified and captured by the systematic searches. The review therefore opted to include studies whose examinations dealt with any of the following categories:

a) Individuals who are radicals; hold radical beliefs; support terrorist groups or actions; in the process of radicalization; experienced radicalization and/or de-radicalization; considered to be at risk of radicalization; are from a pool of people from a wider population considered to be at a greater risk of radicalization.

b) Individuals who have been recruited to terrorism; attempted to have been recruited; self-recruited; attempted to self-recruit; who are in the process of recruitment; who have returned from recruitment; who are at risk of recruitment; are from a pool of people from a wider population considered to be at a greater risk of recruitment

c) Individuals who carried out acts of terrorism; who were caught whilst planning of preparing to carry out acts of terrorism; who attempted but failed to carry out an act of terrorism; who were arrested as part of a terrorist plot; who were criminally charged as being involved in or a part of a terrorist plot; who subsequently reneged on carrying out an act of terrorism

d) Individuals used as part of control or comparison groups to any of the above.

The review set no limitations based on studies based on age, sex, race, religion and ethnicity of the objects of study. Additionally, the review set no limitations based on guiding doctrines and sought to include all doctrines, such as; religious, right-wing, left-wing, ethno-nationalist and single issue etc. There was no theoretical or empirical base to justify the differentiation between the doctrines, but we hoped the review would further the understanding of existing similarities and differences.

This project has received funding from the European Union's Horizon 2020 research and innovation programme under grant agreement N° 699824.

2.6.2 TYPES OF SETTINGS

It is often pointed out in the literature that the 'driving factors' of terrorism are quite different between democratic and non-democratic countries. Additionally, trends in terrorism, as well as radicalization processes appear to be quite different between these countries governed by these different regime types (GTI, 2016). Taking such issues into consideration, it is important for researchers examine to examine terrorism and violence related issues separately between high-income and low-income countries (Enders & Hoover, 2012). Terrorism in democratic countries appears to be more prevalent and more stable than it is elsewhere. Furthermore, the recent rise in the threat of terrorism in such countries demands further development of our understanding of risk factors leading to radicalization and recruitment to terrorism that are specific to such places (Blomberg et-al, 2004; Sandler & Enders, 2006; GTI, 2016). Moreover, much of the literature on radicalization and terrorism focusses on non-democratic countries in Africa and the Middle-East and lessons learned from such studies may be less applicable to democratic contexts. Lastly, there is added methodological value in the separating of searches based on country and country type, including with regards to the databases which should be searched and also with respect to the synthesis of the data identified (Shenderovich et al, 2016). This review therefore limited its inclusion criteria to only those studies which in the settings of countries that have been categorised by the Democracy Index as being either *full democracies* or *flawed democracies* and where they were categorized as such during the period relevant to a study's analysis. Additionally, when a study included more than one country, and only one or more of these countries were in the inclusion criteria but other countries were not, then these studies were considered to meet the inclusion criteria where all other criteria had been met.

Indeed, a lot of the literature which appeared in this review's systematic searches was ultimately excluded on this basis. For example, Hasan (2015) found that the interaction between education and unemployment is an important risk factor for radicalization of belief and radicalization of action in Egypt and Bangladesh. Additionally, according to Fair (2014), Pakistani militants from LET and HM were statistically more likely to hold higher than average education compared to the general population. While some of these findings may provide important and even exportable lessons, other studies identified in this review corroborate the understandings of spatial differences. For example, one included study by Zhirkov et-al (2014) found that relative deprivation is much more important in European countries compared to Muslim majority countries. More specifically, Spain, the location of a recent attack (on 18 August 2017) had the highest correlation with collective relative deprivation.

This project has received funding from the European Union's Horizon 2020 research and innovation programme under grant agreement N° 699824.

Clearly there can be important differences between contexts. This review sought out to identify such differences by comparing the EU with the US and other democratic settings such as Israel.

**Full democracies**	**Flawed democracies**
Norway, Iceland, Sweden, New Zealand, Denmark, Canada, Ireland, Switzerland, Finland, Australia, Luxembourg, Netherlands, Germany, Austria, Malta, United Kingdom, Spain, Mauritius, Uruguay	*Japan, United States, Italy, Cape Verde, France, South Korea, Costa Rica, Botswana, Portugal, Israel, Estonia, Czech Republic, India, Taiwan, Chile, Belgium, Cyprus, Slovenia, Lithuania, South Africa, Jamaica, Latvia, Slovakia, Timor-Leste, Greece, Panama, Trinidad and Tobago, Bulgaria, Indonesia, Argentina, Philippines, Brazil, Poland, Suriname, Croatia, Ghana, Hungary, Dominican Republic, Colombia, Peru, El Salvador, Romania, Mongolia, Lesotho, Serbia, Malaysia, Sri Lanka, Mexico, Hong Kong, Tunisia, Singapore, Namibia, Paraguay, Guyana, Senegal, Papua New Guinea, Moldova*

Figure 3 List of included countries

This project has received funding from the European Union's Horizon 2020 research and innovation programme under grant agreement N° 699824.

2.6.3 Search strategy

The review set out to search for both published and unpublished literature based on the above inclusion criteria and search terms that were developed and selected after many pilot searches. When dealing with systematic reviews, there is a delicate balance between sensitivity and specificity, and this varies greatly between different databases. It was decided that none of the outcomes would be part of our searches in order to avoid limiting the number of relevant studies that may come from other fields, such as psychology. As has been noted in the literature on systematic reviews, reviews dealing with risk factors may have limited search tiers due to the need to balance sensitivity and search precision (Hammerstrøm et-al, 2009; Shenderovich et-al, 2016). The research team also consulted with librarians and academics with expertise in systematic searches, especially in the social and behavioural sciences.

Below is a list of the 4 search tiers used by this review:

1. Radical* OR recruit* OR extrem* OR violent extrem* OR violent radical* OR foreign fighter* OR Terror* or Lone wol* or lone-wol* or homegrown or home-grown or home grown or sympath* OR support OR facilitate OR engage* OR activis*
2. Jihad* OR Islam* OR Salaf* OR right-wing OR neo-Nazi OR far-right OR nationalist OR white-supremacist OR left wing OR extreme left OR anarchist OR single-issue
3. *Risk* OR *factor* OR predict* OR propensity OR likelihood OR predispose* OR predisposition OR vulnerab* OR causal OR putative OR determinant OR Root OR correlate*
4. *democra* or West* or Europ* or Norw* or Iceland or Swed* or New Zealand or Denmark or dutch or Canad* or Ireland or Irish or Switzerland or swiss or Finland or Finnish or Australia* or Luxembourg or Netherlands or holland or German* or Austria* or Malta or United Kingdom or England or British or Spain or Spanish or Mauritius or Uruguay or Japan* or United States or USA or America* or Ital* or Cape Verde or France or french or South Korea* or Costa Rica or Botswana or Portugal or Israel* or Jew* or Palestin* or Arab or Estonia or Czech Republic or India* or Taiwan* or Chile* or Belgi* or Cyprus or Slovenia or Lithuania or South Africa* or Jamaica* or Latvia* or Slovakia* or Timor-Leste or Greece or Greek or Panama or Trinidad and Tobago or Bulgaria* or Indonesia* or Argentina* or Philippin* or Brazil* or Poland or Suriname or Croatia* or Ghana or Hungar*

This project has received funding from the European Union's Horizon 2020 research and innovation programme under grant agreement N° 699824.

or Dominican Republic or Colombia* or Peru* or El Salvador or Romania* or Mongolia or Lesotho or Serbia* or Malaysia* or Sri Lanka* or Mexic* or Hong Kong or Tunisia* or Singapor* or Namibia or Paraguay or Guyana or Senegal or Papua New Guinea or Moldova

The search strings were applied in the following databases:

Campbell library	Journal of de-radicalization	Sociological abstracts
ISI Science	Google Scholar	Bibsys
PsycINFO	SSRN	START
PubMed	SCO	Perspective on Terrorism
Campbell library	Journal of de-radicalization	Sociological abstracts

This review sought to follow the most recognized standards for a systematic review of this scope. To this end a title registration and protocol has been submitted to the Campbell Collaboration. The title registration was approved and published on in June 2016. The Protocol will be published in the coming months, followed by a full report based largely off of the current report. This methodology also allows for updating the review on a continual basis to include new studies from this fast-developing field.

This project has received funding from the European Union's Horizon 2020 research and innovation programme under grant agreement N° 699824.

2.7 Initial searches, review and querying

The results from the initial searches led to the collection of 9006 indexed items which were indexed in a shared reference manager library. Automated duplicate detection and removal resulted in 6445 original items. These included 150 items from Google Scholar searches. A team of trained reviewers then performed the initial screening based on abstract and title, and considering the established inclusion and exclusion criteria. This first screening resulted in 374 itsems which were subjected to a secondary screening led by the senior reviewers who subsequently reduced this to 135 items. Each of these items was subsequently read in its entirety and extracted by the review team. Following this stage, a further 36 items were removed due to falling outside of the inclusions criteria, primarily due to the settings in which the studies were carried out, or because they did not provide a quantiative analysis which met the inclusion criteria. Of the remaining items, 84 items examined micro level factors, while 11 studies examine macro level factors and 4 studies examines meso level factors.

In addition, the reviewers carried out a search of grey literature and also contacted internationally regarded researchers and research institutions together with the list of identified studies. The research team also contacted two authors in order to attempt to include their studies where the studies were inaccessible or missing information. The researchers were able to include one dissertation study that among the 18 relevant dissertations identified.

In addition, the three PROTON studies of T2.3, T2.5 and T2.7 were included. The addition of these studies, which examined terrorists and compared them with comparison groups, added two important studies to the review and their inclusion enabled many of the meta-analyses presented in the foregoing sections.

This project has received funding from the European Union's Horizon 2020 research and innovation programme under grant agreement N° 699824.

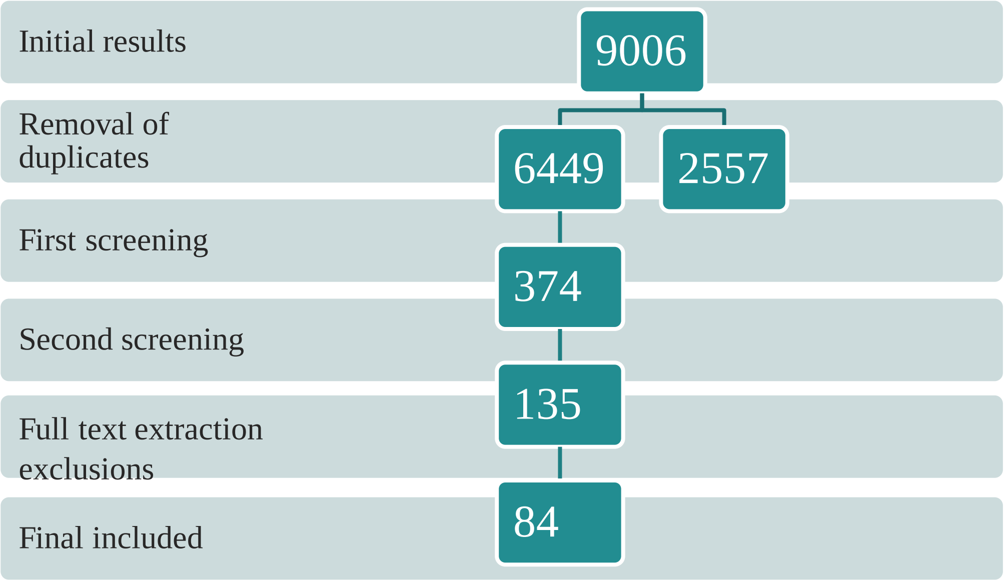

Figure 4- Flow chart following the approach of Moher et-al (2009).

The included studies covered a broad range of approaches to examining either radicalization of belief or radicalization of action. Different methodologies and approaches dictated how the studies were categorized, coded, and synthesized in the review. The below table presents this division:

30 studies coded for meta-analysis	-19 studies examining beliefs (including support/willingness etc.)<br>-12 studies examining actions (including terrorists, convicted terrorist, self-reported radical violence etc.)
44 un-coded studies	-15 studies using SEM or other methods that could not be synthesized in meta-analysis.<br>-15 quantitative studies that compared different types of offenders and could not be synthesized in meta-analysis.
9 studies examining RWA and SDO research	9 studies examining Right-wing Authoritarianism and/or Social Dominance Orientation in the context of radical belief or radical action.

Figure 5 Types of studies included in T2.1

This project has received funding from the European Union's Horizon 2020 research and innovation programme under grant agreement N° 699824.

Some of these studies have employed comparison groups, and compared violent terrorist offenders with other types of violent offenders, or even non-violent terrorist offender groups (n=5). A few of the studies focussed exclusively on lone-wolf terrorists, home-grown terrorists, or foreign fighters. Overall, the trend seems to be towards more research examining radical attitudes, beliefs, justifications, support and willingness or intentions.

2.7.1 Trends in research, approaches and findings

The results of the systematic searches show that there is a marked increase in useable, quantitative radicalization 2016 to 2017. More than 50% of the studies included were published in the last three years alone. The trends towards more quantitative research in 2016-2017 offers promise for the production of more high quality empirical studies that can in turn lead to better understanding regarding risk factors of radicalization and recruitment to terrorism.

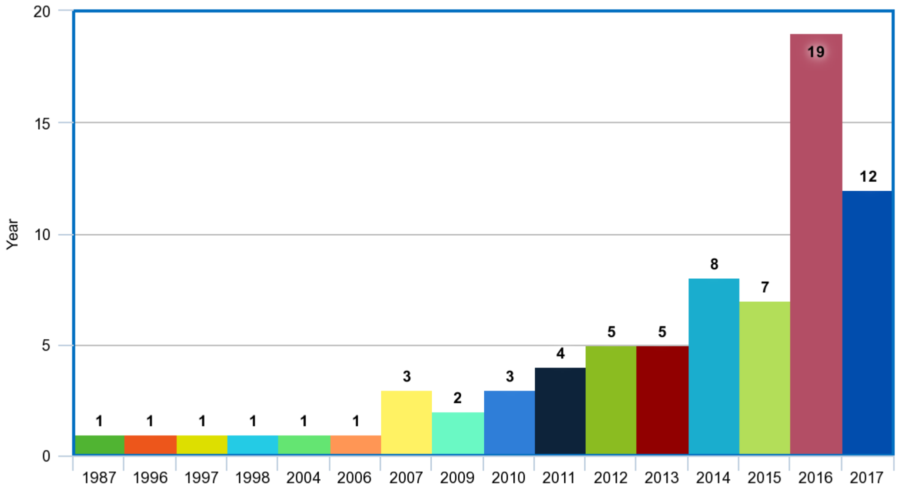

Figure 6:Histogram including un-coded studies

There is also a move towards researching radical attitudes and beliefs, and comparing radical actors with other types of offenders. Overall, most studies examined radical attitudes and beliefs in the form of support or justification of terrorism.

This project has received funding from the European Union's Horizon 2020 research and innovation programme under grant agreement N° 699824.

2.7.2 Types of studies:

The studies included in this review were primarily based on cross-sectional data, however there were some longitudinal, experimental, and quasi-experimental studies.

Type	***N***
Cross sectional	75
Longitudinal	2
Experimental	2
Quasi design	1
Descriptive	2

1 Included studies by type

In terms of analytic methodologies, most studies employed some form of regression analyses, most commonly in the form of various logistic regression models. However, a number of studies have employed some form of Path analysis/Confirmatory Factor Analysis/Structural Equation Modelling. While many consider these to be strong methodologies for examining this type of data, especially when generated from self-report cross-sectional surveys, there are critics. Some not that with these types of methodologies, there are problems with inferences. Additionally, the way in which such methods combine variables and create dependent variable contructs meant that they could not be used in meta-analysis. In the future however, there is scope for such studies to be synthasized together, using new methods that have only recently been tested in other fields.

2.7.2.1 Experimental, quasi-experimental and longitudinal research

Experimental research is rare but is ultimately the direction that researchers are hoping to take the study of radicalization. Some of this work is being carried out as part of doctoral studies and available only in thesis format. This shows that there is promise for creative approaches to achieving the 'gold standard' in research. For example, this review identified three studies which use an experimental methodology to examine radicalization.

This project has received funding from the European Union's Horizon 2020 research and innovation programme under grant agreement N° 699824.

In one study, Dechesne (2009) employed a computer based experiment using recognition and reaction latencies to examine the Struggle-Violence Link and specific risk factors associated with it. The experiment combined the results with the results from a survey which participants also completed. As the authors note, those who reported negative social youth experiences exhibited were more likely to have a greater strength of association between struggle and violence. Additionally, greater self-concept instability was found to be marginally associated with stronger association between struggle and violence. Although obtained in laboratory situation that is by all accounts far removed from the real situation in which terrorism is enacted, the findings nonetheless appear to converge with some insights into the psychological dynamics at play in the world of terrorism. In another experimental study, Kalmoe (2014) used random survey samples and introduced different violent texts pertaining to political parties. He then used the survey instruments to assess participants' support for political violence. This research found that those with general trait aggression were pre-disposed to being 'radicalized' by the violent messages in the form of expressing support for political violence. While these two studies were both conducted in a laboratory type setting and may only be indirectly connected to radicalization to violent extremism, their creative approaches could inform future research. Additionally, the risk factors identified by these studies should be considered by policy makers as important.

Another experimental approach is that of Dornschneider (2016) who created a computer based model profile of radical violent murderers. In this book, "Whether to Kill: The Cognitive Maps of Violent and Nonviolent Individuals", he attempts to model differences between violent and non-violent. This book follows all the steps that one would like to see in moving this type of research forward. Computer based simulations and experiments can help make up for the lack of real world experiments, which are difficult for a number of reasons, with security and ethical concerns topping the list.

As will be discussed below, two additional quasi-experimental studies were identified from the field of Right-wing Authoritarianism and Social Dominance Orientation research. Both these studies, carried out by well known terrorism researchers, sought to adapt these validated instruments in a radicalization context. In the first study, Lemieux and Asal (2010) used an experimental approach to examine support for terrorism over legitimate political means, a form of radical beliefs. In this study, the researchers manipulated levels of grievance of 2932 US adult participants. A vignette

This project has received funding from the European Union's Horizon 2020 research and innovation programme under grant agreement N° 699824.

tool was employed and participants then indicated their likelihood of engaging in any form of political action as a result. Participants were able to choose between violent actions and peaceful protest. They were also given the opportunity to indicate the extent to which they justified each form of action. The authors found that those who were in the high grievance condition were more likely to favor taking action, and felt that both forms of action were more justified. Those who were higher on both SDO and RWA generally indicated less likelihood of taking any action, however those higher on only SDO were more likely to both choose the terror attack and indicate that it was more justified.

In another study, Kruglanski et-al (2017) have employed a single-group before-after quasi-experimental design in a Philippines prison setting to examine prison radicalization and risk factors. Whilst this is not the only quantitative study examining prison radicalization identified in this review, it is the only longitudinal quasi-experimental design. In this study the authors found evidence of prison radicalization, however only 29 of the original 82 participants were included in Wave 2. Whilst the authors found that none of the risk factors predicted changes in Islamic extremism, they did find that time in prison did. The authors should be commended for publishing the study given the issues of attrition and a lack of significant findings. Nevertheless, the issue of prison radicalization remains as one that is not well understood.

One other study employing a before-after quasi experimental design is Feddes' (2015) longitudinal study which employed resilience training to increasing self-esteem and empathy to reduce radicalization of belief. This design study, and quality of the data and results, could add to the argument that mental health issues are becoming more important, more apparent, or both, something which will be discussed in the foregoing sections of this report.

This project has received funding from the European Union's Horizon 2020 research and innovation programme under grant agreement N° 699824.

2.8 META ANALYSIS

In total the review identified 22 risk factors upon which meta-analyses could be conducted on account of their being a minimum of two studies and two different effect sizes to input. Some of these variables were labeled differently but based on their descriptions we were able to group some of them together with accuracy. There were an additional five (5) variables for which there was only one study, however the importance of these findings will be discussed in its proper context in the appropriate sections of this report.

Some variables were grouped following a careful examination of the studies' description of variables and their constructs. For example, there were 4 studies that examined whether being an immigrant is a risk factor for developing radical belief and/or action, whereas 2 studies examined whether being a citizen, or non-citizen had an impact. Whereas we cannot assume that being a citizen is equal to being a non-immigrant, we can assume that most non-citizans are immigrants. It happened that the studies, such as Krueger (2008), the "citizen" variable, which was highly significant was defined as being in opposition to the notion that "immigrants are much less likely to be incarcerated than are native born americans". Additionally, another variable, "idle" was described as being someone who is "neither working nor attending school". We grouped this together with unemployment. If there would have been other similar variables in other studies perhaps we would have made a separate variable, however at the least we can be sure that it means unemployed.

Of the 30 studies coded for meta-analysis, 8 of them study data on those who have committed acts of radical action, 2 studies examine willingness, and another two studies look at both willingness and beliefs together. In total, 20 of the studies examine radical beliefs by looking at beliefs, support and/or willingness. Since these are conceptually part of the same radicalization model, we have run the primary analyses with all studies combined. Where relevant, the analyses have been conducted with removing studies and performing sub-group analyses. We have included the two in publication studies by our partners, (T2.5) LaFree et-al (2017) & (T2.7) Weerman et-al (2017). We feel these add interesting data to unravel similarities and differences that exist between the US and Europe.

This project has received funding from the European Union's Horizon 2020 research and innovation programme under grant agreement N° 699824.

Two of the studies which looked at radical action presented two models, one for radical action against property and the other for radical action against persons. In sticking with the definitions laid out in the protocol for this review, we included only the results from the violence against persons. In all analogous cases in which more than one model was presented by a study, the more radical models were chosen or those models which included the most control variables.

Our analyses on age and gender only confirm that which is already known regarding the likelihood of violent offenders and those with propensity of support for violence generally being young males. We find important differences in the magnitudes, both actual and relative between radical belief and radical action, as well as between regions.

All analyses were carried out using Biostat's Comprehensive Meta-Analysis (CMA) software (Borenstein et-al, 2009) for performing the meta-analyses, creating forest plots, publication bias analyses and heterogeneity tests. The review followed standard procedures for converting and standardizing effect sizes from observational and correlational studies. Since calculating of a single measure is preferable to conducting multiple separate meta-analyses for each risk factor split by effect size measurement type all measures were standardized to Fisher's Z for final input (Borenstein et al, 2009). All analyses were carried out using the Random Effects (Mixed effects) models. These models are the most appropriate for the large homogeneity that is characteristic of correlational data from the types of observational studies identified and included in this review. This is in part because risk factor studies generally examine more heterogeneous populations than interventions do, as has been noted by analyses of risk factors for gang involvement (Higginson et-al, 2014) and juvenile delinquency (Lanza et-al, 2016).

This project has received funding from the European Union's Horizon 2020 research and innovation programme under grant agreement N° 699824.

2.8.1 AGE AND SEX

As is the case with criminal and violent offenders in general, radical offenders tend to be mostly young males, as attested to by almost all of the studies included in this review. However, the overall relevance of age in the context of other risk factors is not well understood. Additionally, while most radical attackers may be young, many of those who hold radical belief may span an wider age range. Additionally, different types of radicals in different contexts will be of different age brackets. The results of the below meta-analysis are indicative of these complexities. Despite age being a statistically significant correlate in most of the included studies, the pooled OR is not significant (p=0.147), nor is age significant for the beliefs (p=0.472) or actions groups (p=0.141). Whilst it is enough to know that offenders are most likely to be young males, age is not a good predictor of radical belief or radical action, with both results being non-significant and displaying quite small effect sizes relative to other factors.

Risk factor: Age

Group by Group	Study name	Statistics for each study				
		Odds ratio	Lower limit	Upper limit	Z-Value	p-Value
Actions	Jasko 2016	1.022	0.850	1.228	0.232	0.817
Actions	Sela Shayovitz (2007)	1.022	0.595	1.755	0.079	0.937
Actions	LaFree et-al (2017)	1.029	0.855	1.239	0.307	0.759
Actions	Krueger	1.037	0.834	1.290	0.326	0.745
Actions	Altunbus 2011	1.095	0.908	1.321	0.947	0.343
Actions	Berrebi 2007	1.423	1.374	1.473	19.776	0.000
Actions	Pauwells & Schils (2016)	3.476	3.155	3.829	25.194	0.000
Actions	Pauwells et-al	4.267	3.699	4.922	19.915	0.000
Actions		1.511	0.873	2.617	1.473	0.141
Beliefs	Victoroff et-al (model2)	0.010	0.007	0.014	-25.422	0.000
Beliefs	Cherney & Povey (2013)	1.000	0.919	1.089	0.000	1.000
Beliefs	Beller & Kröger (2017)	1.004	0.919	1.096	0.081	0.935
Beliefs	LaRue (2012)	1.007	0.809	1.255	0.065	0.948
Beliefs	Coid et-al (2016)	1.022	0.909	1.149	0.364	0.716
Beliefs	Victoroff et-al (2012)	1.041	0.872	1.242	0.443	0.658
Beliefs	Littler	1.075	0.898	1.288	0.789	0.430
Beliefs	Acevedo et-al (2015)	1.111	0.867	1.423	0.833	0.405
Beliefs	Cherney & Murphey (2017)	1.115	0.867	1.434	0.847	0.397
Beliefs	Pedersen et-al (2017)	1.152	1.062	1.250	3.411	0.001
Beliefs	MaCauley et-al (2012)	1.199	0.962	1.494	1.617	0.106
Beliefs	Bhui et-al (2016)	2.170	1.615	2.917	5.138	0.000
Beliefs	Delia-Deckard & Jacobson (2015)	2.715	2.194	3.360	9.185	0.000
Beliefs	Zhirkov et-al (2014)	7.042	6.379	7.774	38.712	0.000
Beliefs	Bhui et-al (2014)	9.983	7.090	14.059	13.175	0.000
Beliefs		1.158	0.776	1.728	0.719	0.472
Overall		1.270	0.919	1.755	1.449	0.147

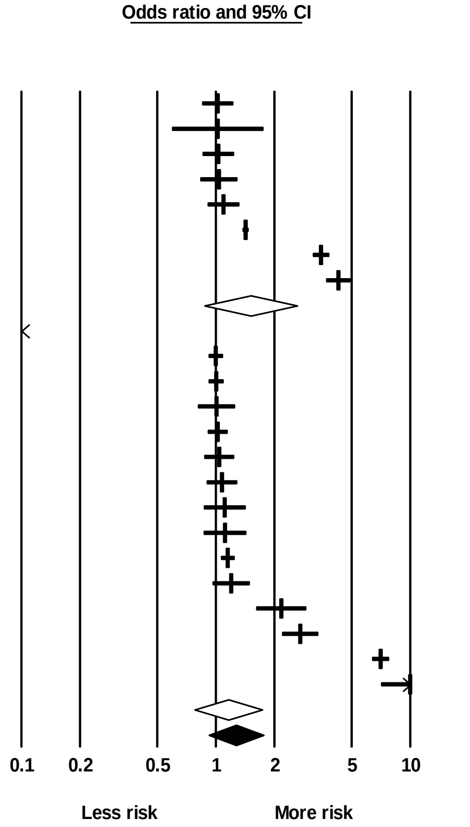

2 Meta-analysis on "Age"

This project has received funding from the European Union's Horizon 2020 research and innovation programme under grant agreement N° 699824.

It should be intuitive that those carrying out radical violence are generally older than school shooters for example (Lankford, 2013). Klausen et-al (2016) have identified that the terror age crime curve is demonstrably different from the general age-crime curve. Similar findings were identified by T2.3 which found that terrorism offenders were much older than ordinary criminals, specifically with the age at time of first incarceration. Terrorism offending peaks at a much later age and occurs over a larger age range compared with regular criminal offending. There may also be important differences between different types of actors acting on behalf of different ideological doctrines. For example, Gill et-al (2014) found that Al Qaeda inspired terrorists were an average of 10 years younger than right-wing and other types of terrorists. According to Gill and Young (2011), Palestinian suicide terrorists were older than non-suicide terrorists of any persuasion in the US, as also reflected in the finding that such terrorists were more likely to have been married. In many cases, younger and older individuals may be quite likely to hold radical views. Nevertheless, males in their 20's are the most likely to turn from belief to action.

This project has received funding from the European Union's Horizon 2020 research and innovation programme under grant agreement N° 699824.

2.8.2 Marital status

There are conflicting conceptions regarding the role of marital status in radicalization and recruitment to terrorism. While many consider it to be a protective factor, being married is often a significant positive correlate with both radical beliefs and radical actions. Part of the confusion is because marital status is also closely associated with age. For example, Gill and Young (2011) found that Palestinian suicide bombers were also much more likely to be married than terrorists of any persuasion in the US. Additionally, many of the communities from which radicals may emerge may hold values and norms that encourage marriage at a younger age than that of the majority population. As noted above, radical offenders are also older than regular criminals, and an increased prevalence of being married may simply be a reflection of this fact.

In this review, there were a total of 7 studies which provided effect sizes for marital status. A cumulative model found that marital status was not a statistically significant risk factor for radicalization and recruitment to terrorism (p=0.149). The results of the analysis showed that marital status' importance for radicalization of action was even less significant (p=0.876), whereas its effect on radicalization of belief, albeit a small effect, is statistically significant at the .05 level (p=0.03).

Risk factor: Marital status

Group by Group	Study name	Statistics for each study: Odds ratio	Lower limit	Upper limit	Z-Value	p-Value
Actions	Berrebi (2007)	0.403	0.270	0.603	-4.428	0.000
Actions	LaFree (2017)	0.796	0.661	0.958	-2.411	0.016
Actions	Altunbus (2011)	1.208	1.001	1.457	1.969	0.049
Actions	SelaShayovitz (2007)	2.203	1.267	3.830	2.798	0.005
Actions		0.948	0.482	1.865	-0.155	0.876
Beliefs	Bhui et-al (2014)	0.480	0.357	0.645	-4.878	0.000
Beliefs	Cherney (2013)	2.528	2.316	2.760	20.720	0.000
Beliefs	Acevedo (2014)	2.665	2.062	3.443	7.496	0.000
Beliefs	Delia Deckard (2015)	5.653	4.502	7.099	14.908	0.000
Beliefs		2.084	1.074	4.045	2.171	0.030
Overall		1.417	0.882	2.276	1.442	0.149

Odds ratio and 95% CI

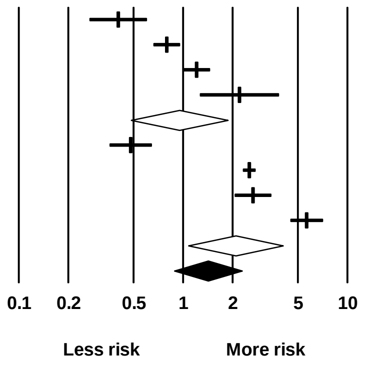

3 Meta analysis on "Marital status"

It would seem from these results that being married is a stronger risk factor for radicalization of beliefs than it is for radicalization of action and this could be connected to age. This seems to be what was found by T2.3 as well. The direction of the non-significant effects would indicate towards a possible protective effect against radical actions, as found in T2.5

This project has received funding from the European Union's Horizon 2020 research and innovation programme under grant agreement N° 699824.

2.8.3 INDIVIDUAL SOCIO-ECONOMIC STATUS

The debate about the role of real individual socio-economic status with respect to radicalization and terrorism is ongoing. While many studies specifically seek out to investigate the connection between socio-economic status and radicalization outcomes, most of the studies in this review find that there is an inverse association and that higher socio-economic status is positively associated with radicalization of belief (e.g. Delia-Deckard & Jacobson, 2016; Bhui et-al, 2014a). The meta-analyses provide quantitative evidence to support the position that individual SES is not related to radicalization. As can be seen below, a cumulative model found that SES was an insignificant risk factor (p=0.998) with an Odds Ratio (OR) only slightly above 1 (1.001). A moderator analysis was conducted in which the studies were moderated by region (EU Vs. US only). While the OR for the EU was larger (1.558) than it was for the US (1.023), both results were statistically insignificant, as was the overall effect (1.023). Additionally, there were no differences between these two groups (p=0.899). This risk factor remained the lowest and least significant of all of those examined in this review.

Risk factor: SES

Study name	Statistics for each study				
	Odds ratio	Lower limit	Upper limit	Z-Value	p-Value
Pedersen et-al (2017)	0.045	0.041	0.050	-60.347	0.000
MaCauley et-al (2012)	0.441	0.352	0.552	-7.129	0.000
Nivettee (2017)	0.865	0.705	1.061	-1.391	0.164
Cherney & Murphey (2017)	1.075	0.836	1.383	0.565	0.572
Coid et-al (2016)	1.169	1.039	1.314	2.605	0.009
Bhui et-al (2016)	1.671	1.248	2.237	3.445	0.001
Delia Deckard (2015)	2.278	1.845	2.812	7.656	0.000
Acevedo et-al (2015)	2.391	1.854	3.083	6.714	0.000
Bhui et-al b (2014b)	5.260	3.828	7.228	10.235	0.000
	1.001	0.299	3.358	0.002	0.998

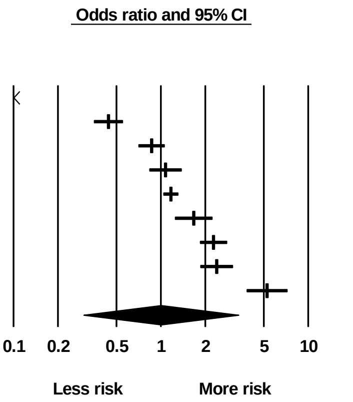

4 *Meta-analysis on "SES"*

This project has received funding from the European Union's Horizon 2020 research and innovation programme under grant agreement N° 699824.

Risk factor: SES

Group by Region	Study name	Odds ratio	Lower limit	Upper limit	Z-Value	p-Value
EU	Nivettee (2017)	0.865	0.705	1.061	-1.391	0.164
EU	Cherney & Murphey (2017)	1.075	0.836	1.383	0.565	0.572
EU	Coid et-al (2016)	1.169	1.039	1.314	2.605	0.009
EU	Bhui et-al (2016)	1.671	1.248	2.237	3.445	0.001
EU	Bhui et-al b (2014b)	5.260	3.828	7.228	10.235	0.000
EU		1.558	0.846	2.869	1.424	0.155
US	MaCauley et-al (2012)	0.441	0.352	0.552	-7.129	0.000
US	Acevedo et-al (2015)	2.391	1.854	3.083	6.714	0.000
US		1.023	0.390	2.684	0.047	0.963
Overall		1.381	0.825	2.314	1.228	0.219

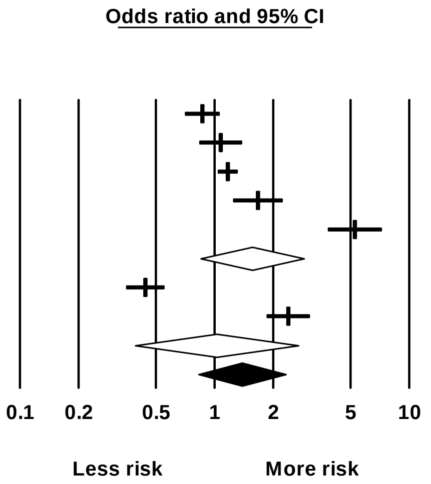

5 Meta-analysis on "SES" moderated by region

This analysis provides strong quantitative evidence to support the notion that individual real SES is not a significant risk factor for radicalization and/or recruitment to terrorism. This does not mean that policy makers should cease their attempts to increase the SES of their residents and citizens. Rather, it means that conceptions about terrorists being more likely to come from poverty are not rooted in evidence. To the contrary, most of the research finds the opposite and most prominent researchers tend to be an agreement on this. Indeed, the results of this analysis provide evidence in support of this.

This project has received funding from the European Union's Horizon 2020 research and innovation programme under grant agreement N° 699824.

2.8.4 EMPLOYMENT STATUS

There is much discussion in the literature about the role of employment as a risk factor. Generally speaking, it is believed that unemployment, or under-employment is a risk factor for radicalization, whereas employment is a protective factor. There are some issues of interpretation here which warrant additional research. For example, employment may be related to age, and marital status, whereas unemployment may be related to age, education, and being a full-time student. Only a few studies disaggregate the employment/unemployment variable in such a way so as to account for these other sources of unemployment. Nevertheless, a moderator analysis was conducted on "employment" and found that overall, it has a weak albeit significant effect (OR=1.589, p=0.025). When looking at the different groups separately however, employment is not significant for radical beliefs (p=0.065) or radical actions (p=0.197).

Risk factor: Employment

Group by Group	Study name	Odds ratio	Lower limit	Upper limit	Z-Value	p-Value
		Statistics for each study				
Actions	LaFree (2017)	0.610	0.506	0.735	-5.179	0.000
Actions	Krueger (2008)	1.111	0.893	1.382	0.944	0.345
Actions	Altunbus (2011)	2.409	1.986	2.921	8.927	0.000
Actions	Lujic et-al (2017)	2.984	2.717	3.277	22.868	0.000
Actions		1.491	0.813	2.733	1.291	0.197
Beliefs	Cherney (2013)	1.199	1.101	1.305	4.178	0.000
Beliefs	Bhui et-al (2014)	1.470	1.099	1.966	2.598	0.009
Beliefs	Bhui et-al (2016)	1.519	1.136	2.032	2.816	0.005
Beliefs	Acevedo (2014)	1.824	1.420	2.344	4.700	0.000
Beliefs	Delia Deckard (2015)	2.705	2.186	3.348	9.153	0.000
Beliefs		1.674	0.969	2.892	1.849	0.065
Overall		1.589	1.059	2.385	2.238	0.025

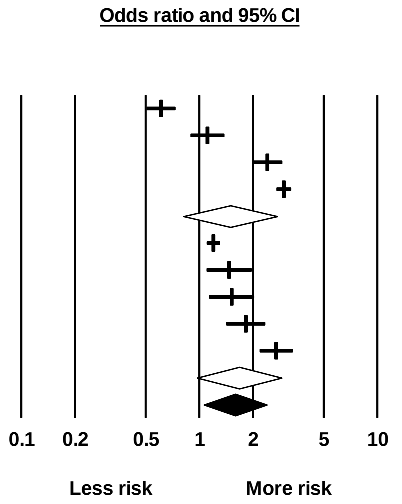

6 Meta-analysis on "Employment"

These results may be related to the model used, since a moderator analysis model uses a shared TAU. In an attempt to look deeper into the issue, a separate analysis was conducted for both groups and found that employment status has a small, albeit significant effect on radicalization of belief. That is, being employed is a positive risk factor for radicalization of belief (OR=1.392, p=0.003).

This project has received funding from the European Union's Horizon 2020 research and innovation programme under grant agreement N° 699824.

Risk factor: Employment (Beliefs only)

Study name	Odds ratio	Lower limit	Upper limit	Z-Value	p-Value
Cherney (2013)	1.199	1.101	1.305	4.178	0.000
Bhui et-al (2014)	1.470	1.099	1.966	2.598	0.009
Bhui et-al (2016)	1.519	1.136	2.032	2.816	0.005
Acevedo (2014)	1.824	1.420	2.344	4.700	0.000
Delia Deckard (2015)	2.705	2.186	3.348	9.153	0.000
	1.672	1.196	2.337	3.005	0.003

Statistics for each study

Odds ratio and 95% CI

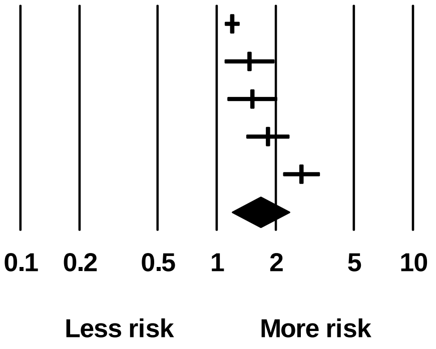

7 Meta-analysis on "Employment" (beliefs group only)

Wherever possible, this review attempted to identify differences between radicalization of action and belief, as well as between the EU and US (or elsewhere). With respect to employment, it was found that overall there was a small but significant effect on radicalization of action and that this effect was considerably larger for the EU (OR=2.695, p=0.000) than it was for the US (OR 0.818) which was statistically insignificant (p=0.322).

Risk factor: Employment (Actions only)

Group by Region	Study name	Odds ratio	Lower limit	Upper limit	Z-Value	p-Value
EU	Altunbus (2011)	2.409	1.986	2.921	8.927	0.000
EU	T2.7	2.984	2.717	3.277	22.868	0.000
EU		2.695	1.833	3.960	5.046	0.000
US	LaFree (2017)	0.610	0.506	0.735	-5.179	0.000
US	Krueger (2008)	1.111	0.893	1.382	0.944	0.345
US		0.818	0.550	1.217	-0.991	0.322
Overall		1.512	1.147	1.994	2.933	0.003

Statistics for each study

Odds ratio and 95% CI

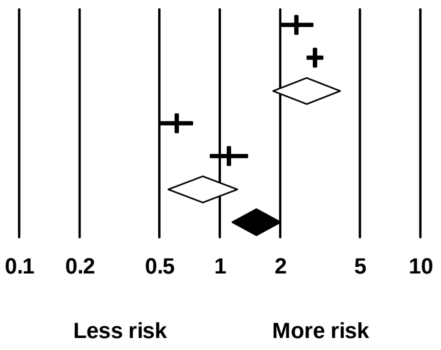

8 Meta-analysis on "Employment" (Actions groups only) moderated by region

This project has received funding from the European Union's Horizon 2020 research and innovation programme under grant agreement N° 699824.

These findings demonstrate the value of looking for differences between not only types of radicalization but also differences specific to regions.

There are some additional considerations however when interpreting the true effects of employment and unemployment on radicalization outcomes. As mentioned above, in some studies unemployment may be due to being a full-time student or not needing to work. In the case of the former there may be an important interaction between unemployment when coupled with high education. In other cases, unemployment may be associated with job loss. Especially in the case of recent job loss, some studies have found that this could be a possible risk factor and trigger. For example, T2.7's second model found a significant effect of recent job loss on the odds of engaging in radical action in the Netherlands (OR 2.32). Additionally, Bhui et-al (2016) found that recent job loss had a positive correlation with support for terrorism. However, Bhui et-al (2014b) found that retired, ill or housewife respondents were more likely to hold radical beliefs as well (RR 8.81). Studies which examine the effects of unemployment should focus on this type of disaggregation and on differences between various sources of unemployment.

This project has received funding from the European Union's Horizon 2020 research and innovation programme under grant agreement N° 699824.

2.8.5 Education

One of the most emphasized and regularly examined risk factors in the literature is education. It is often theorized that poor education—often together with poverty—is a serious risk factor for radicalization. Others believe that it is those who are better educated who are at a greater risk, pointing to the fact that many terrorists had university degrees and that those holding radical beliefs tend to come a more affluent and educated class. Many earlier studies in radicalization and terrorism research found that the university itself was a breeding ground for radicalization Russell and Miller Captain (1977) found that the interaction between being a young male from a relatively well to do family and starting university was the strongest combination of risk factors for radicalization. Lee (2011) found that the "terrorist profile" of Bengali political "terrorists" from the early 1900's included higher education.

The relevance of education as a risk factor for radicalization is reflected in the number of studies that include it as a variable (19) and which explore it explicitly. When pooled together, higher education does have a degree of significant risk, although the overall effect size is only modest (OR 2.092). This analysis identifies that higher education also has a stronger effect for radicalization of belief (OR 2.425) than for radicalization of action (1.607), with such differences being significant (*p<0.1)*.

Risk factor: Education

Group by Group	Study name	Statistics for each study: Odds ratio	Lower limit	Upper limit	Z-Value	p-Value
Action	Krueger (2008)	1.060	0.852	1.318	0.521	0.602
Action	T2.5	1.060	0.880	1.276	0.613	0.540
Action	Altunbus 2011	1.140	0.945	1.375	1.364	0.173
Action	Berrebi (2007)	1.628	1.098	2.414	2.427	0.015
Action	Jasko (2016)	1.900	1.576	2.290	6.731	0.000
Action	Sela Shayovitz (2007)	2.186	1.258	3.801	2.773	0.006
Action	T2.7	3.358	3.055	3.691	25.099	0.000
Action		1.607	1.126	2.295	2.613	0.009
Belief	Victoroff et-al (2012b)	1.041	0.835	1.296	0.356	0.722
Belief	Larue (2012)	1.327	1.065	1.655	2.519	0.012
Belief	Zhirkov et-al (2014)	1.611	1.475	1.758	10.642	0.000
Belief	Bhui et-al (2016)	1.921	1.432	2.577	4.356	0.000
Belief	Acevedo et-al (2015)	2.463	1.909	3.178	6.931	0.000
Belief	Victoroff et-al (2012a)	2.463	2.054	2.954	9.718	0.000
Belief	Beller et-al (2017)	2.519	2.301	2.758	20.009	0.000
Belief	Delia-Deckard (2015)	2.576	2.083	3.186	8.733	0.000
Belief	Littler (2017)	3.345	2.767	4.044	12.466	0.000
Belief	Coid et-al (2016)	3.476	3.070	3.934	19.692	0.000
Belief	Cherney (2013)	3.598	2.755	4.699	9.396	0.000
Belief	Bhui et-al (2014b)	6.229	4.506	8.610	11.074	0.000
Belief		2.425	1.857	3.167	6.506	0.000
Overall		2.092	1.689	2.590	6.773	0.000

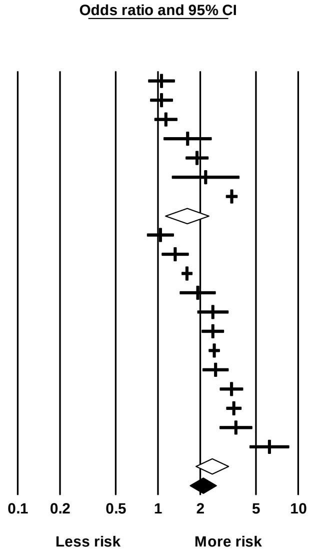

9 Meta-analysis on "Education"

This project has received funding from the European Union's Horizon 2020 research and innovation programme under grant agreement N° 699824.

Moderator analyses were subsequently performed for both groups. When looking at the radicalization of beliefs group only, the overall OR remains relatively stable. However, important differences are apparent between regions. The effect of education on radicalization of belief is strongest in the EU (OR 2.929) where it is also statistically significant (p=0.000). The effects for the US and Other countries are not significant.

Risk factor: Education (Beliefs group only)

Group by Region	Study name	Odds ratio	Lower limit	Upper limit	Z-Value	p-Value
EU	Zhirkov et-al (2014)	1.611	1.475	1.758	10.642	0.000
EU	Bhui et-al (2016)	1.921	1.432	2.577	4.356	0.000
EU	Victoroff et-al (2012a)	2.463	2.054	2.954	9.718	0.000
EU	Littler (2017)	3.345	2.767	4.044	12.466	0.000
EU	Coid et-al (2016)	3.476	3.070	3.934	19.692	0.000
EU	Cherney (2013)	3.598	2.755	4.699	9.396	0.000
EU	Bhui et-al (2014b)	6.229	4.506	8.610	11.074	0.000
EU		2.929	2.131	4.027	6.620	0.000
Other	Beller et-al (2017)	2.519	2.301	2.758	20.009	0.000
Other	Delia-Deckard (2015)	2.576	2.083	3.186	8.733	0.000
Other		2.547	1.418	4.574	3.128	0.002
US	Victoroff et-al (2012b)	1.041	0.835	1.296	0.356	0.722
US	Larue (2012)	1.327	1.065	1.655	2.519	0.012
US	Acevedo et-al (2015)	2.463	1.909	3.178	6.931	0.000
US		1.498	0.920	2.440	1.625	0.104
Overall		2.423	1.901	3.088	7.150	0.000

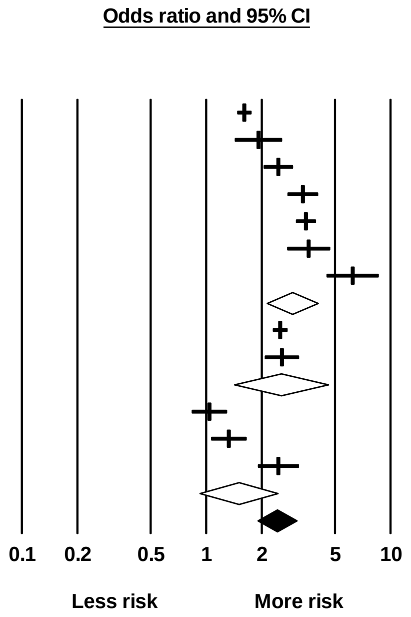

10 Meta-analysis on "Education" (Beliefs group only) moderated by region

When we analyzed the effects of education on the radicalization of actions group, whilst the overall effect size was significant at the .05 level (p=0.029), when moderating by region, there were no significant findings for the EU (p=0.090), US (p=0.440) or Israel (p=0.146). Overall, education does not appear to be a significant risk factor for radicalization of action.

This project has received funding from the European Union's Horizon 2020 research and innovation programme under grant agreement N° 699824.

Risk factor: Education (Action group by region)

Group by I	Study name	Odds ratio	Lower limit	Upper limit	Z-Value	p-Value
EU	Altunbus 2011	1.140	0.945	1.375	1.364	0.173
EU	Weerman et-al (2017)	3.358	3.055	3.691	25.099	0.000
EU		1.967	0.900	4.303	1.695	0.090
IL	Berrebi (2007)	1.628	1.098	2.414	2.427	0.015
IL	Sela Shayovitz (2007)	2.186	1.258	3.801	2.773	0.006
IL		1.872	0.804	4.360	1.454	0.146
US	Jasko (2016)	1.900	1.576	2.290	6.731	0.000
US	Krueger (2008)	1.060	0.852	1.318	0.521	0.602
US	LaFree et-al (2017)	1.060	0.880	1.276	0.613	0.540
US		1.288	0.677	2.451	0.771	0.440
Overall		1.610	1.049	2.471	2.178	0.029

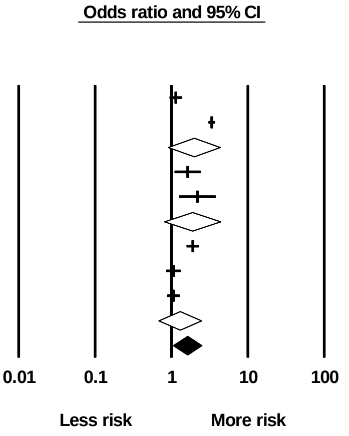

11 Meta-analysis on "Education" (Actions group only) moderated by region

Education's relevance in radicalization is not straightforward. According to Pyrooz et-al (2017), compared to gang members, lone-wolf terrorists are better educated. Additionally, there may be important differences between different types of actors. For example, while 54% of single issue terrorists had university experience, Gill et-al (2014) found that it was only a significant risk factor for right-wing and AQ inspired terrorists. Being a full-time student was also significant for these groups (Gill et-al, 2014). According to Gill and Young (2011), Palestinian suicide terrorists in Israel had overall higher education than non-suicide terrorists of all persuasions in the US. Gill et-al (2014) also found that successful attackers were more likely than unsuccessful attackers to have higher education. Additionally, in Jenkins (2011) set of 82 cases of home-grown terrorists in the U.S. from 2002 through 2010, many had been enrolled or in full-time education but never graduated, perhaps due to their attacks or arrest.

This project has received funding from the European Union's Horizon 2020 research and innovation programme under grant agreement N° 699824.

2.8.6 IMMIGRANT STATUS

The issue of immigration is in part so contested because of the fear of radicalization and recruitment to terrorism. Beyond this, already radicalized returning foreign fighters may come with immigrant waves. Nevertheless, an examination of this variable cannot give insights into how to deal with this aspect of the wider immigration issues. However, in this analysis, immigrant status was found to have a small, albeit significant effect at the .05 level (OR=1.602, p=.043). However, the effect on radicalization of action (OR 2.765) was greater than the effect on radicalization of belief (OR 1.034), with the latter not being significant (p=0.915).

Risk factor: Immigrant status

Group by Group	Study name	Statistics for each study: Odds ratio	Lower limit	Upper limit	Z-Value	p-Value	Region
Actions	Krueger (2008)	1.576	1.264	1.963	4.049	0.000	US
Actions	Altunbus (2011)	2.427	2.000	2.943	8.999	0.000	EU
Actions	Jasko (2016)	2.917	2.408	3.534	10.937	0.000	US
Actions	T2.7	5.177	4.688	5.716	32.518	0.000	EU
Actions		2.765	1.393	5.488	2.909	0.004	
Beliefs	Acevedo (2014)	0.239	0.183	0.312	-10.549	0.000	US
Beliefs	McCauley (2012)	0.670	0.537	0.836	-3.545	0.000	US
Beliefs	Pederson et-al (2017)	1.022	0.942	1.109	0.525	0.600	EU
Beliefs	Delia Deckard (2015)	2.635	2.130	3.259	8.927	0.000	Other
Beliefs	Coid et-al (2016)	2.645	2.342	2.986	15.701	0.000	EU
Beliefs		1.034	0.560	1.910	0.107	0.915	
Overall		1.602	1.014	2.530	2.020	0.043	

Odds ratio and 95% CI

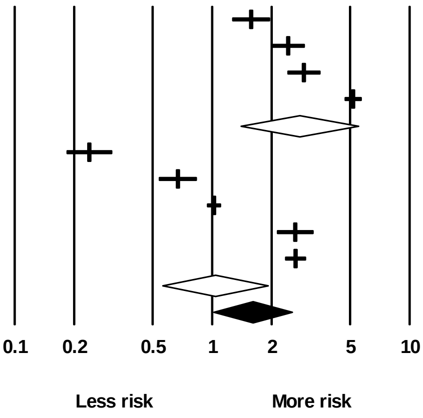

12 Meta-analysis on "Immigrant status"

In the above analysis, of the three action group studies, two examine the issue in the context of the US (Krueger, 2008; Jasko et-al, 2016) and two in the context of the EU (Altunbus, 2011; T2.7). In the beliefs group there are also two US based studies and two EU based studies (and one other). As

This project has received funding from the European Union's Horizon 2020 research and innovation programme under grant agreement N° 699824.

such, additional analyses were carried out on each outcome while moderating for region. In the beliefs group, the analysis found that immigrant status was not significant for any of the groups.

Risk factor: Immigrant status (beliefs group)

Group by Region	Study name	Odds ratio	Lower limit	Upper limit	Z-Value	p-Value
EU	Pederson et-al (2017)	1.022	0.942	1.109	0.525	0.600
EU	Coid et-al (2016)	2.645	2.342	2.986	15.701	0.000
EU		1.642	0.640	4.214	1.032	0.302
Other	Delia Deckard (2015)	2.635	2.130	3.259	8.927	0.000
Other		2.635	0.686	10.118	1.411	0.158
US	Acevedo (2014)	0.239	0.183	0.312	-10.549	0.000
US	McCauley (2012)	0.670	0.537	0.836	-3.545	0.000
US		0.402	0.154	1.044	-1.872	0.061
Overall		1.144	0.346	3.782	0.220	0.826

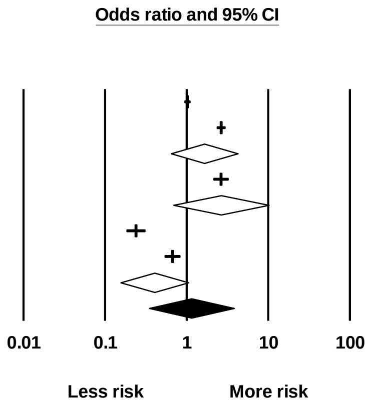

13 Meta-analysis on "Immigrant status" (Beliefs group only) moderated by region

For the actions group, a moderator analysis found that overall there was little change in the overall effect (OR= 2.773). However, region specific effects show that while significant for both regions, immigrant status has a larger OR for the EU (OR=3.563) compared to the US (OR=2.148).

Risk factor: Immigrant status

Group by Region	Study name	Odds ratio	Lower limit	Upper limit	Z-Value	p-Value	Region
EU	Altunbus (2011)	2.427	2.000	2.943	8.999	0.000	EU
EU	T2.7	5.177	4.688	5.716	32.518	0.000	EU
EU		3.563	1.781	7.131	3.590	0.000	
US	Krueger (2008)	1.576	1.264	1.963	4.049	0.000	US
US	Jasko (2016)	2.917	2.408	3.534	10.937	0.000	US
US		2.148	1.066	4.328	2.138	0.032	
Overall		2.773	1.694	4.541	4.056	0.000	

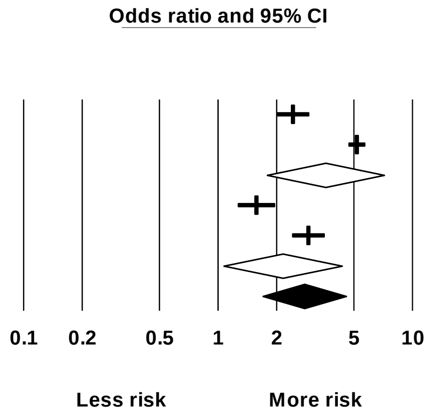

This needs to be better understood in context however. In a recent study, Schils & Pauwells (2014) examined the issue of immigrant status in conjunction with age and gender. They find that immigrant males have a lower propensity for radical action than non-immigrant males, although immigrant females have a higher propensity than non-immigrant females. They stress the importance of better understanding the complexities of immigrant status as a risk factor. According to Simon et-al's (2013) study, immigrant status is a significant correlate of radical belief, especially

This project has received funding from the European Union's Horizon 2020 research and innovation programme under grant agreement N° 699824.

when coupled with issues of dual identity that many immigrants seem to struggle with (Schils & Pauwells, 2014; Lyons, 2016). It is in this context that the risk factors of immigrant status and integration are likely to have a strong interaction with each other. While the EU has seen a recent flood of immigration, whose effects will remain unknown for some time, overall, being an immigrant is not a significant risk factor on its own for predicting radical beliefs. However, of those that turn to radical action, a significant percentage are likely to come from immigrant background. However, more needs to be done to examine the risk associated with second generation. In T2.7, it was found that while being a first generation immigrant was a risk factor for being involved in radical actions in the EU (OR=5.17), being second generation had slightly higher odds ratio (5.98). However, Delia-Deckard and Richardson (2015) found that each generation removed from immigration had an increasingly smaller chance of developing religious fundamentalism. This finding could also be relevant to the below analysis and discussion on integration, for which immigrant status may have an important interaction effect and differ in its manifestations among those with radical beliefs and those engaging in radical actions (Schils & Pauwells, 2014; Lyons, 2016).

This project has received funding from the European Union's Horizon 2020 research and innovation programme under grant agreement N° 699824.

2.8.7 POOR INTEGRATION

It has been pointed out that many radicals are actually 2nd and 3rd generation immigrants, leading some to suggest that poor integration may be an important factor in their radicalization. In this analysis two studies for radicalization of beliefs were compared with two studies of self-reported radical action. Overall, the effect of poor integration is quite large and significant (OR 3.145). The effect is even larger for self-report radical action in an EU context (OR 3.626). The effect on radical belief is small, albeit significant (OR 1.555) based on two studies, one from the EU and one on Somali immigrants in the US.

Risk factor: Poor integration

Group by Group	Study name	Odds ratio	Lower limit	Upper limit	Z-Value	p-Value
Actions	Pauwells & Schils (2016)	3.436	3.119	3.785	24.986	0.000
Actions	Pauwells et-al (2017)	3.887	3.374	4.478	18.796	0.000
Actions		3.626	3.139	4.189	17.490	0.000
Beliefs	Feddes et-al (2015)	0.748	0.252	2.219	-0.523	0.601
Beliefs	Bhui et-al b (2014b)	1.664	1.243	2.229	3.421	0.001
Beliefs		1.555	1.128	2.144	2.692	0.007
Overall		3.145	2.757	3.588	17.057	0.000

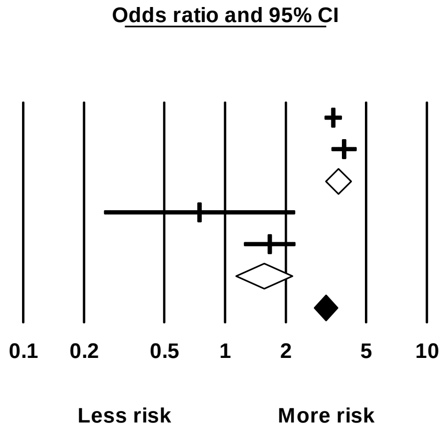

14 Meta-analysis on "Poor integration"

In Feddes et-al's (2015) study, social disconnectedness—while not significant--had a negative effect on radical beliefs but a positive effect direction on violent intentions. According to Lyons (2016), poor integration is a significant risk factor for radicalization of belief. However, it must be understood that the effect of poor integration are exacerbated by actual discrimination and marginalization. Each context is likely to have unique interactions between these risk factors. Pauwells et-al (2014) found that both poor social integration and perceived discrimination had a stronger correlation with radical behaviours than religious authoritarianism did.

This project has received funding from the European Union's Horizon 2020 research and innovation programme under grant agreement N° 699824.

2.8.8 INSTITUTIONAL TRUST AND LEGITIMACY

Another important risk factor that is brought up in the literature is institutional trust. Often studies examine this in the context of trust in procedural justice, or trust in the police. One study which examined the issue most explicitly did so in the context of a sample from the Australian Muslim community. Cherney & Murphey (2017) found that lower legitimacy of the law and trust of the police both predicted radical beliefs. The below analysis found that overall, institutional trust has a small but significant effect (OR=1.657). However, moderator analysis shows that effect is larger for self-reported radical action (OR=2.420) than it is for radical belief (OR 1.353).

Risk factor: Institutional trust

Group by Group	Study name	Odds ratio	Lower limit	Upper limit	Z-Value	p-Value
		Statistics for each study				
Actions	Pauwells et-al (2017)	2.053	1.793	2.350	10.430	0.000
Actions	Pauwells & Schils (2016)	2.798	2.544	3.078	21.169	0.000
Actions		2.420	1.995	2.935	8.965	0.000
Beliefs	Bhui et-al b (2014b)	1.221	0.914	1.631	1.351	0.177
Beliefs	Littler (2017)	1.248	1.042	1.495	2.405	0.016
Beliefs	Cherney & Murphey (2017)	1.289	1.002	1.660	1.973	0.048
Beliefs	Pedersen et-al (2017)	1.449	1.341	1.565	9.440	0.000
Beliefs	Nivettee (2017)	1.492	1.214	1.832	3.813	0.000
Beliefs		1.353	1.175	1.558	4.195	0.000
Overall		1.657	1.478	1.857	8.678	0.000

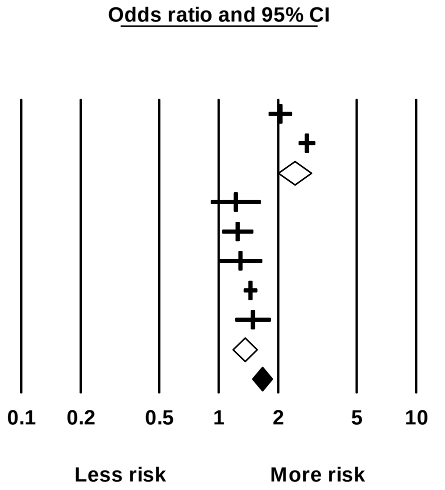

15 Meta-analysis on "Institutional trust"

According to Doosje et-al's (2012) study of right-wing radical belief among dutch youth, respect for the dutch authorities had a strong correlation with respondents' own violent intentions. In another study examining Muslim youth in the Netherlands, Doosje et-al (2013) also identified that legitimacy of the authorities predicted supportive attitudes towards radical violence. Together with the findings of the above meta-analysis, institutional trust seems to have a stronger effect on radical actions compared to radical beliefs.

This project has received funding from the European Union's Horizon 2020 research and innovation programme under grant agreement N° 699824.

2.8.9 DIFFERENTIAL ASSOCIATIONS

Differential associations, including peers, family members and community members, are considered to be especially strong factors in determining one's beliefs and behaviours, especially with respect to issues of conformity and deviance. Social learning theory has widely been used to examine and theorize radicalization processes (Pauwells and Schills, 2016). Some studies, like those of Jasko et-al (2016) and LaFree et-al (2017) and Pauwells & Schills (2016) examine different types of differential associations, separating between relationships with family members and peers. As LaFree et-al (2017) point out, social learning theory perspectives on criminal behaviour are evidently applicable to radicalization and recruitment to terrorism in the US. We see similar findings in the EU, where having radical and deviant peers figures as a strong correlate of radicalization and self-reported radical action (Pauwells & Schills, 2016; Pauwells et-al, 2014).

While the review identified 5 studies that provided correlates for differential associations, the two studies by Pauwells and Schills (2016) and Pauwells and his colleagues (2014) examine self-reported radical action. Not only are these self-reports but both studies provide two models, one for self-reported radical action against property, and the other for radical action against persons. This review's protocol dictated that we code the violent action against persons' effect size only. When pooled we find that differential associations have a mid-range positive effect (OR 2.367). However, we also find that the overall effect on radical action (OR 2.794) is greater than the effect on radical beliefs (OR 1.647) and the differences between the mean effects of these groups is significant (*p* <0.1).

Risk factor: Differential associations

Group by Group	Study name	Statistics for each study: Odds ratio	Lower limit	Upper limit	Z-Value	p-Value
Actions	LaFree et-al (2017)	1.993	1.650	2.408	7.156	0.000
Actions	Jasko 2016	2.269	1.879	2.740	8.513	0.000
Actions	Pauwells et-al (2014)	3.476	3.021	3.999	17.418	0.000
Actions	Pauwells & Schils (2016)	3.768	3.418	4.155	26.641	0.000
Actions		2.794	2.022	3.861	6.225	0.000
Beliefs	Nivettee (2017)	1.156	0.942	1.419	1.391	0.164
Beliefs	Bhui et-al b (2014b)	2.436	1.809	3.280	5.862	0.000
Beliefs		1.647	1.020	2.657	2.043	0.041
Overall		2.367	1.810	3.094	6.301	0.000

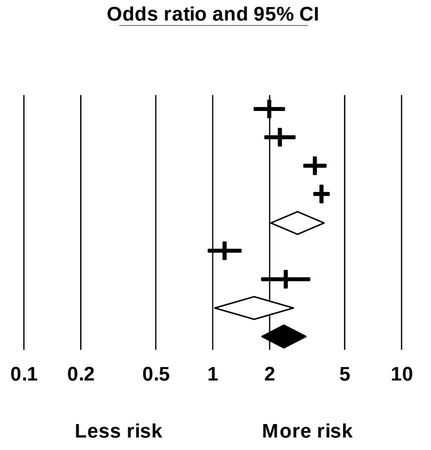

16 Meta-analysis on "Differential associations"

This project has received funding from the European Union's Horizon 2020 research and innovation programme under grant agreement N° 699824.

An additional analysis was carried out on only the actions group and moderating for region. It is important to note that the two EU studies here are based on self-reported radical action, whereas the two US studies are based on convicted terrorists. Each of these represent different categories on the radicalization of action pyramid, namely "activist" and "terrorist" respectively. In this analysis we see that the effect of differential associations is significantly greater (*p*<*.000)* for the EU "activist" actions group (OR 3.670) than it is even for the US "terrorist" actions group (OR 2.127). The results should however be taken with caution, especially given that the EU studies, whilst using different data sets, were carried out by the same group of authors. Similarly, the US studies examine the same dataset (PIRUS) and come from the same research institute (START). Nevertheless, the results of these analyses provide evidence to support social learning perspectives on radicalization.

Risk factor: Differential associations

Group by Region	Study name	Statistics for each study: Odds ratio	Lower limit	Upper limit	Z-Value	p-Value
EU	Pauwells et-al (2014)	3.476	3.021	3.999	17.418	0.000
EU	Pauwells & Schils (2016)	3.768	3.418	4.155	26.641	0.000
EU		3.670	3.388	3.976	31.817	0.000
US	LaFree et-al (2017)	1.993	1.650	2.408	7.156	0.000
US	Jasko 2016	2.269	1.879	2.740	8.513	0.000
US		2.127	1.861	2.431	11.081	0.000
Overall		3.176	2.966	3.402	32.983	0.000

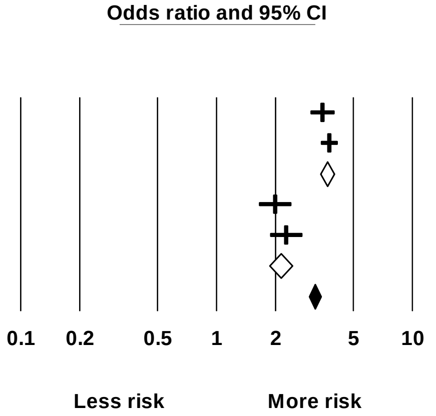

17 Meta-analysis on "Differential Associations" (Actions group only)

These findings appear to be supported by Sawyer & Heinz's (2013) chapter in the Handbook of Criminology of Terrorism. Most of their set of terrorists (55%) were part of a "clique" of some sort. Jensen et-al (2016) also found that membership in a clique was a significant predictor of involvement in terrorism, especially for left-wing terrorism. According to Gill et-al's (2014) study of 119 lone-actor terrorists Al Qaeda inspired and single-issue terrorists were highly likely to have been subjected to pressures from wider groups.

This project has received funding from the European Union's Horizon 2020 research and innovation programme under grant agreement N° 699824.

Differential associations, while clearly having a stronger effect on behaviours than beliefs, are still important for the latter group. According to the findings of Bhui et-al (2014a), having more homogenous social networks is positively correlated with radical beliefs and attitudes. In another study, Van Bergen et-al (2015) has found that that having out-group friendships/associations is a protective factor. These differential associations had a significant impact on connectedness to society, reduced in-group superiority, and ultimately less of a willingness to use violence in defence of the in-group. Tausch (2009) also found similar correlations in that contact with non-Muslims reduced the likelihood of radicalization. On a community level, the less penetration of Muslims in the area, the less the extent of radical belief in the form of supporting terrorism against home country.

In another study, Van Bergen et-al (2015) found that social and egalitarian socialization, especially parental socialization, is a protective factor against attitudes and willingness to use violence in in-group defence. While these studies used path analysis and the findings are difficult to interpret, they are informative, especially in the context of contradictory findings regarding the effect of radical family members. In this regard, three separate examinations of the PIRUS database have found that while radical peers are a significant factor, radical family members are not (Jensen et-al, 2016; Jasko et-al, 2016; LaFree et-al, 2017). Slootman and Tillie's (2006) study used path analysis to examine longitudinal data and found that social isolation, from both family and friends, was a predictor of radical belief.

Overall, the effects of differential associations appear to play an important role in differentiating those from radical belief and radical actions groups and may be an important risk factor for those who may shift from radical belief to radical action.

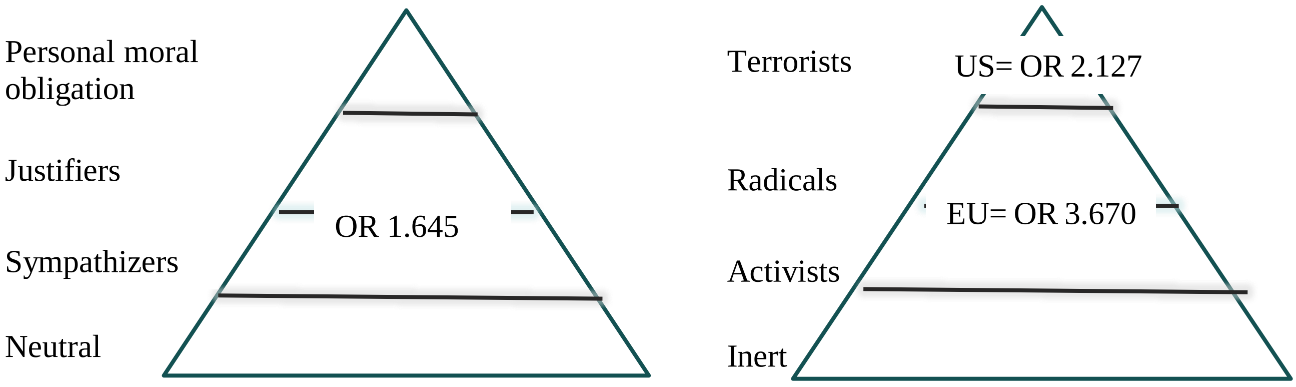

Figure 7 Effect of differential associations on radical beliefs and radical actions between groups

2.8.10 Mental health

Perhaps one of the most recently and debated issues in terrorism and radicalization research is that of the role of mental health. Some of the more well-known literature on terrorism developed a near consensus over the years that not only was mental health not a risk factor for radicalization but that the only psychological trait shared by terrorists was an overwhelming normality (Gill et-al, 2014). Early studies found that terrorists were overwhelmingly normal and absent of psychosomatic issues. For example, Lyons & Harbinson's (1986) comparison of IRA murderers with regular murderers found that the IRA offenders were less likely to suffer from mental illness or a family history of the same. However, a number of scholars now believe that such assumptions and statements were made too early and were founded upon anecdotal and observational evidence (Gill et-al, 2014).

In today's environment of relatively low-intensity lone wolf attacks, the issue of mental health has once again come into the spotlight. Many of the most recent cases of lone wolf terrorism are followed by reports in the media that the attackers' families and peers report mental health issues of the attacker. Such observations appear to be supported by the evidence. In this review, a total of 6 coded studies were identified which provide effect sizes for mental health in the context of radicalization of belief. One set of scholars, led by Bhui (2014, 2014b, 2016) and who come from the field of psychiatry in the UK psychiatrist, have sought to explore the issue of mental health and radicalization of belief explicitly. In their first study they found that, in opposition to their original hypothesis, mental health issues were negatively associated with radical beliefs in the form of support for radical actions. In a secondary study on the same sample, depression was found to be positively associated with support for violent, radical protest and terrorism (Bhui et-al, 2014b). These contradictory findings appear to be representative of the general debate on the issue. However, T2.5 (LaFree et-al, 2017) found that mental health did have a positive correlation with radical action in the US and   Additionally, T2.7 found in their qualitative component, through interviews with some convicted terrorists that self-reported mental health issues figured prominently.

Using moderator analysis and a random effects model, overall, a modest and significant effect size was found for mental health (OR 1.835). There were no studies however that examined the effect of mental health for radical action against a group with which to synthesize the effect size from T2.5.

This project has received funding from the European Union's Horizon 2020 research and innovation programme under grant agreement N° 699824.

Nevertheless, a moderator analysis found that the effect size of mental health on radical belief is higher for the EU (OR 1.798) than it is for the US (OR 1.927)

Risk factor: Mental health

Group by Region	Study name	Odds ratio	Lower limit	Upper limit	Z-Value	p-Value
EU	Bhui et-al b (2014b)	1.029	0.771	1.375	0.197	0.844
EU	Coid et-al (2016)	1.541	1.370	1.735	7.181	0.000
EU	Bhui et-al (2016)	2.586	1.918	3.486	6.232	0.000
EU	Feddes et-al (2015)	6.153	1.830	20.690	2.937	0.003
EU		1.798	1.183	2.733	2.746	0.006
US	Ellis et-al (2016)	1.581	1.090	2.294	2.414	0.016
US	Ellis et-al (2015)	2.798	1.199	6.533	2.379	0.017
US		1.927	1.002	3.708	1.965	0.049
Overall		1.835	1.289	2.611	3.372	0.001

Statistics for each study

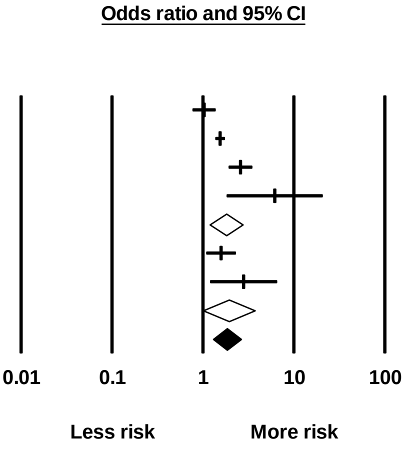

18 Meta-analysis on "Mental health"

It is important to note that the first 3 studies from the EU group were carried out by the same group of authors, and that the two Bhui et-al studies (2014a & 2016) used the same samples. Additionally, the two US based studies were conducted by the same set of authors, although different samples were used. More work needs to be done on disaggregating and examining different types of mental health issues that may present varying and differing effects (Horgan et-al, 2016).

While the meta-analysis cannot inform on the effects of mental health on radical action, the literature indicates that there may be important differences between different types of actions. For example, Bakker and de Bont (2016) found that only 2% of their sample of European foreign fighters suffered from known mental health issues. However, Corner and Gill (2015) found that the odds of a lone actor having mental illness were 13.49 times higher than that of group/organizational based terrorist. According to Gruenwald et-al (2013), right-wing loner attackers are also more likely to suffer from mental illness than group based right-wing offenders. According to Gill et-al (2014) Al Qaeda inspired terrorists were less likely than all other types of terrorists to suffer from mental illness. Such differences are important to take into consideration for how mental health is approached as a risk factor for radicalization and recruitment to terrorism. Additionally, according to Gill et-al (2014), successful attackers are more likely to suffer from mental illness than failed attackers.

This project has received funding from the European Union's Horizon 2020 research and innovation programme under grant agreement N° 699824.

Nevertheless, overall, those engaging in radical action are apparently less likely to have mental health issues than ordinary homicide offenders. In this respect, both Lyons and Harbinson's (1986) study of IRA actors and Horgan et-al's (2016) study of lone wolves found that they were less likely to have mental health issues than their regular homicide offender counterparts.

Mental health: Terrorists Vs. Murderers

Study name	Statistics for each study				
	Odds ratio	Lower limit	Upper limit	Z-Value	p-Value
Lyons & Habrinson (1986)	0.151	0.060	0.378	-4.032	0.000
Horgan et-al (2016)	0.710	0.390	1.295	-1.117	0.264
	0.341	0.075	1.553	-1.391	0.164

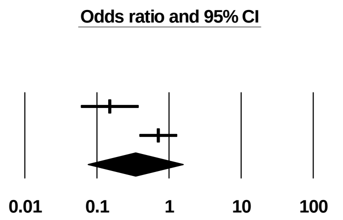

According to Gill et-al (2016), there are very few temporal differences between terrorist cohorts, however mental health may be one of those factors and it is currently evidencing itself in its relationship to radicalization of belief.

This project has received funding from the European Union's Horizon 2020 research and innovation programme under grant agreement N° 699824.

2.8.11 RACISM EXPERIENCE

Racism and discrimination are often pointed to as exacerbating factors which can lead to radicalization and recruitment to terrorism. Only one of the 7 studies which examined being a victim of racism examined the issue in the context of self-reported radical action (Pauwells & Schils, 2016). The analysis shows that this risk factor has a significant moderate effect (OR 1.958). This effect is considerably larger for the EU (OR 2.852) than it is for the US (OR 1.106) where it is statistically insignificant (p=0.559). This is an interesting finding that may provide an important distinction between the experiences of at risk populations in different contexts and regions.

Risk factor: Experienced racism

Group by Region	Study name	Statistics for each study				
		Odds ratio	Lower limit	Upper limit	Z-Value	p-Value
EU	Pedersen et-al (2017)	2.178	2.014	2.356	19.487	0.000
EU	Bhui et-al b (2014b)	2.203	1.639	2.961	5.232	0.000
EU	Pauwells & Schills (2016	3.064	2.784	3.373	22.881	0.000
EU	Victoroff et-al (2012a)	4.437	3.667	5.370	15.312	0.000
EU		2.852	2.170	3.750	7.510	0.000
US	MaCauley et-al (2012)	1.075	0.863	1.340	0.647	0.518
US	Victoroff et-al (2012b)	1.095	0.879	1.364	0.809	0.419
US	Ellis et-al (2016)	1.160	0.802	1.679	0.789	0.430
US		1.106	0.789	1.549	0.584	0.559
Overall		1.958	1.583	2.421	6.200	0.000

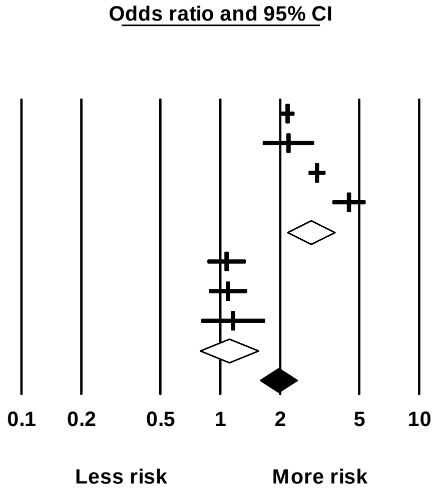

Figure 8 Experienced racism

An additional analysis which excluded the Pauwells and Schils (2016) study found that this study's removal had little effect on the results, with only minor decreases in overall effects (OR 1.781) and EU only effects (OR 2.777). The differences between the groups remained significant (*p=.002).*

This project has received funding from the European Union's Horizon 2020 research and innovation programme under grant agreement N° 699824.

Risk factor: Experienced racism

Group by Region	Study name	Odds ratio	Lower limit	Upper limit	Z-Value	p-Value
EU	Pedersen et-al (2017)	2.178	2.014	2.356	19.487	0.000
EU	Bhui et-al b (2014b)	2.203	1.639	2.961	5.232	0.000
EU	Victoroff et-al (2012a)	4.437	3.667	5.370	15.312	0.000
EU		2.777	1.861	4.144	5.000	0.000
US	MaCauley et-al (2012)	1.075	0.863	1.340	0.647	0.518
US	Victoroff et-al (2012b)	1.095	0.879	1.364	0.809	0.419
US	Ellis et-al (2016)	1.160	0.802	1.679	0.789	0.430
US		1.107	0.732	1.675	0.481	0.631
Overall		1.781	1.335	2.375	3.929	0.000

Statistics for each study

Odds ratio and 95% CI

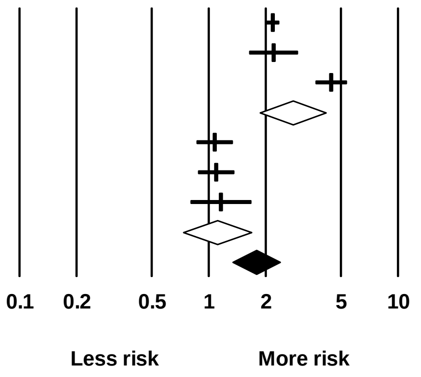

19 Meta-analysis on "Experienced racism" (Beliefs group only) moderated by region

Experiencing racism is only one form of discrimination, albeit one that is experienced on the individual and personal level. While this analysis cannot explain how at risk populations perceive discrimination more broadly and how it impacts their beliefs, the effects of this issue do differ considerably when comparing the EU to the US. In this context, Bhui et-al (2014a) did not find that having been a victim of discrimination had a significant correlation with radical beliefs. It is important to maintain a separation and distinction between individual experience of racism and other forms of discrimination, and perceived discrimination. In the case of the latter, perceptions of discrimination are more suited to be examined within the context of relative deprivation (e.g. Doosje et-al, 2012, 2013) (see below).

This project has received funding from the European Union's Horizon 2020 research and innovation programme under grant agreement N° 699824.

2.8.12 Experienced violence

Many violent radicals are believed to have come from troubled background where violence was an issue. Whether experiences of violence have occurred in the context of the home, school, gangs, encounters with the police, civil war or insurgency, experiencing and being a victim of violence and violence related trauma is considered to be a risk factor for violent offending in general. Only one study in this review examined having experienced violence in the context of radical actions (Jasko et-al, 2016). The lack of evidence on this issue is understandable given that self-reports of having been a victim of violence are more likely to come from the surveys used to analyse radical beliefs than from open sourced data on terrorist characteristics. An overall model l found a relatively strong effect (OR 3.205) and this remained quite stable after removing the Jasko et-al (2016) study (OR 3.131).

Risk factor: Experienced violence

Study name	Statistics for each study				
	Odds ratio	Lower limit	Upper limit	Z-Value	p-Value
Pedersen et-al (2017)	2.463	2.276	2.665	22.395	0.000
Coid et-al (2016)	3.668	3.238	4.154	20.448	0.000
Ellis et-al (2015)	4.284	1.779	10.314	3.245	0.001
	3.131	2.182	4.493	6.194	0.000

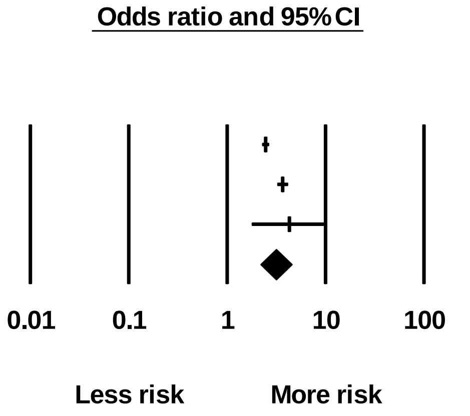

20 Meta-analysis on "Experienced violence" (Beliefs group only)

Given that the Ellis et-al (2015) study was carried out on a relatively small and homogenous group of immigrants in the US, whereas the Pedersen et-al (2017) and Coid et-al (2016) studies focussed on larger samples from the EU, an additional analysis examining only these two studies was carried out. The new pooled OR of 2.997 remains significant and probably provides a better estimate of the average effect of having experienced violence on radicalization of beliefs in an EU context.

This project has received funding from the European Union's Horizon 2020 research and innovation programme under grant agreement N° 699824.

Risk factor: Experienced violence

Study name	Odds ratio	Lower limit	Upper limit	Z-Value	p-Value
Pedersen et-al (2017)	2.463	2.276	2.665	22.395	0.000
Coid et-al (2016)	3.668	3.238	4.154	20.448	0.000
	2.997	2.029	4.427	5.513	0.000

Odds ratio and 95% CI

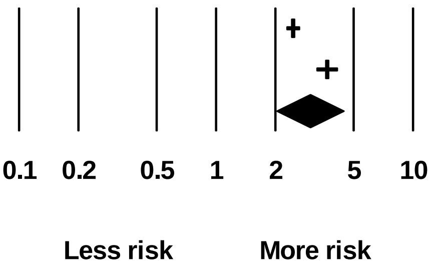

21 Meta-analysis on "Experienced violence" EU only

This project has received funding from the European Union's Horizon 2020 research and innovation programme under grant agreement N° 699824.

2.8.13 Social media usage

The effects of social media on radicalization have been hotly debated in recent years. Nevertheless, while social media has been widely accused of being complicit in creating more radicals, and in accelerating radicalization processes, many researchers point out that even in cases where the internet is known to have played a role in radicalization and terrorism, offline associated were more important. While the study of the effects of social media increases, there are still surprisingly few quantitative studies examining direct correlations. The one study to examine the topic most explicitly was carried out by Pauwells and Schills (2016) who examined different types of radical social media usage and their effects on self-reported radical actions. Other studies have tended to examine only overall internet or social media use. In the first analysis an overall effect of 2.223 was identified, representing a moderate and significant effect (p=0.000), with a smaller effect for radicalization of beliefs (OR=1.739, p=0.005) and a larger effect for the single actions group study (OR=4.284).

Risk factor: Social media

Group by Group	Study name	Odds ratio	Lower limit	Upper limit	Z-Value	p-Value
Actions	Pauwells & Schils (2016)	4.284	3.881	4.728	28.873	0.000
Actions		4.284	2.290	8.013	4.553	0.000
Beliefs	Nivettee (2017)	1.156	0.942	1.419	1.391	0.164
Beliefs	Pedersen et-al (2017)	1.599	1.480	1.727	11.914	0.000
Beliefs	Ellis et-al (2016)	3.257	2.209	4.802	5.962	0.000
Beliefs		1.739	1.185	2.551	2.828	0.005
Overall		2.223	1.603	3.082	4.789	0.000

Statistics for each study

Odds ratio and 95% CI

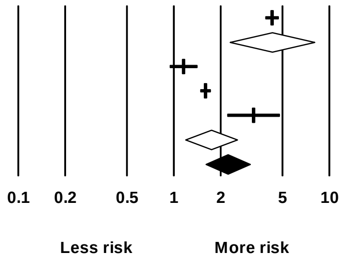

Figure 9 Social media

In order to provide a more accurate estimate, a second analysis was carried out without the actions group study. In this analysis the pooled effects dropped to OR 1.739, a small but still significant effect..

This project has received funding from the European Union's Horizon 2020 research and innovation programme under grant agreement N° 699824.

Risk factor: Social media usage

Study name	Odds ratio	Lower limit	Upper limit	Z-Value	p-Value
Nivettee (2017)	1.156	0.942	1.419	1.391	0.164
Pedersen et-al (2017)	1.599	1.480	1.727	11.914	0.000
Ellis et-al (2016)	3.257	2.209	4.802	5.962	0.000
	1.739	1.185	2.551	2.828	0.005

Odds ratio and 95% CI

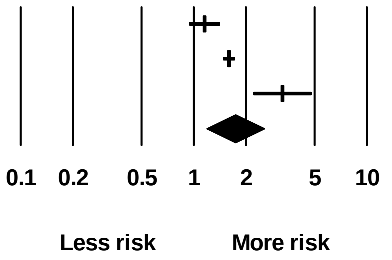

Due to the fact that the Ellis et-al (2016) study focussed on a small homogenous group of immigrants in America, whereas the other two studies focussed on larger samples drawn from EU populations, and on account of the apparent heterogeneity, another analysis was carried out only on Nivette et-al (2017) and Pedersen et-al (2017). Nivette et-al (2017) found insignificant correlations between violent media consumption, which included violent and extremist social media content and radical online peers. Pedersen et-al (2017) found a significant correlation between using social media for political expression and radical beliefs. The results of this analysis show that social media has a fairly weak, albeit significant effect on radicalization of opinion in the EU context (OR 1.379).

Risk factor: Social media usage

Study name	Odds ratio	Lower limit	Upper limit	Z-Value	p-Value
Nivettee (2017)	1.156	0.942	1.419	1.391	0.164
Pedersen et-al (2017)	1.599	1.480	1.727	11.914	0.000
	1.379	1.005	1.893	1.992	0.046

Odds ratio and 95% CI

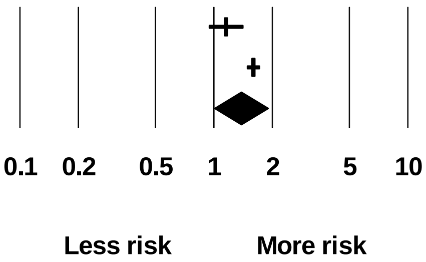

22 Meta-analysis on "Social media usage" (Beliefs group EU only)

With social media being a relatively recent phenomenon in and of itself, it can be expected that new studies that will be published over the coming years and this should improve our understanding of

This project has received funding from the European Union's Horizon 2020 research and innovation programme under grant agreement N° 699824.

the role of social media. While overall social media appears to be important, relative to other factors it has a weak effect on radicalization of opinion. However, as evidenced by the approach taken by Pauwells and Schills (2016), the type of activities and behaviours taken online, as well as frequency of use, can strongly predict differences in the effects of social media on individual beliefs and actions. As shown in Ellis et-al's (2016) study of Somalian immigrants in North America, while hours spent on the internet was not a significant factor in predicting radical beliefs, importance of online community was. In fact, whilst not statistically significant, the most frequent internet usage was associated with a negative effect. In Pedersen et-al's (2017) study, using social media to discuss and post politics was a significant predictor of supporting political violence and for supporting foreign fighters. Interestingly, it was not a significant factor for the third model which predicted support for humanitarian travel to Syria. Only Nivette (2017) found no correlation or effect between consumption of violent media. These are not the only studies which have examined the issue of social media and radicalization however. For example, Gill et-al (2014) and Meloy and Gill (2016) both found that social media usage is more significant for Islamic terrorists than right-wing or other terrorists. According to Horgan et-al (2016b), lone actors are more likely to engage in virtual interactions with informants and receive encouragement from a wider network compared to those working in groups. According to Gill et-al (2014), unsuccessful attackers are much more likely to have used the internet more than successful attackers, especially since their internet usage may lead to their arrest and prevention of their possible plans.

This project has received funding from the European Union's Horizon 2020 research and innovation programme under grant agreement N° 699824.

2.8.14 Criminal history

One of the objectives of PROTON was to investigate possible links between criminality and terrorism. It was to this end that T2.3 explored the incarceration histories of charged and convicted terrorists in Israel. This study found that previous incarcerations were one of the best predictors of future offending. This finding is similar to what has been known for some time in the criminology literature, that the single greatest risk factor for future offending is prior offending. In recent years there has been much written about a possible nexus between criminality and radicalization. Primarily this came in the form of a large qualitative based literature that focussed on the idea of prison radicalization. Yet, while this literature certainly demonstrated that radicalization does occur in prisons in western countries, we have yet to witness cases of terrorism where it is clear that the individual attacker's radicalization was largely related to their stints in prison.

In examining the profiles of some European foreign fighters, Bakker (2009) found that some 25% of European jihadists had recorded criminal histories and suspected that the number was actually much larger on account of missing data. According to the German Federal Office for the Protection of the Constitution (2014) report, some 30% of German foreign fighters had criminal records prior to radicalization, with this number growing to a whole 65% by the time of their departure overseas. This is very similar to what has been found by T2.7, that over 29% of terrorism offenders in the Netherlands dataset had criminal records for other offences.

Previous criminality has recently come to light as a potentially important risk factor for radicalization but one that is not properly understood. PROTON and WP2 sought to further our understanding of this crime-terror nexus by examining the correlation of criminal history and terrorism offending in the US (T2.5) and in the context of the EU (T2.7). Both these studies found strong and significant correlations. Especially in the case of the Netherlands, T2.7, an exceptionally strong Odds Ratio of criminal history among terrorism offenders when analysed against the general population.

While the findings of T2.3, T2.5 and T2.7 clearly show strong correlations between previous criminality and terrorism offending, this still does not say much about the impact on radicalization processes. To this end, this review (T2.1) identified two studies which also examine the criminal

This project has received funding from the European Union's Horizon 2020 research and innovation programme under grant agreement N° 699824.

history variable in the context of radical beliefs in the form of support for terrorism. Both studies were carried out in the UK by the same group of psychiatric researchers, however different samples were used. Only the Coid et-al (2016) study moves in the direction that crime-terror research should be focussing by trying to disaggregate the criminality variable. Here they examine any self-reported criminal violence in the last 5 years, any convictions in the last 5 years, and whether or not the respondents had ever been imprisoned. For the purposes of the meta-analysis only imprisonment was used—which the study found to be significant on its own—as this was the same way that criminality was measured by the other studies. The findings of the moderator analysis are quite informative, however must also be taken with some caution given the large confidence intervals. Nevertheless, the direction of the effects indicates that regardless of where the true effect lies, it would likely only be even greater than what is found here.

Risk factor: Criminal History

Group by Group	Study name	Odds ratio	Lower limit	Upper limit	Z-Value	p-Value	Region	Group
Actions	LaFree (2017)	1.759	1.458	2.122	5.892	0.000	US	Actions
Actions	Jasko (2016)	3.710	3.051	4.512	13.134	0.000	US	Actions
Actions	Weerman (2017)	13.090	11.727	14.612	45.833	0.000	EU/IL	Actions
Actions	Sela-Shayovitz (2007)	21.096	10.412	42.744	8.463	0.000	EU/IL	Actions
Actions		6.321	2.304	17.337	3.582	0.000		
Beliefs	Bhui et-al (2014)	4.839	3.531	6.632	9.805	0.000	EU	Beliefs
Beliefs	Coid et-al (2016)	6.104	5.354	6.958	27.056	0.000	EU	Beliefs
Beliefs		5.441	1.324	22.355	2.350	0.019		
Overall		6.009	2.643	13.659	4.280	0.000		

Odds ratio and 95% CI

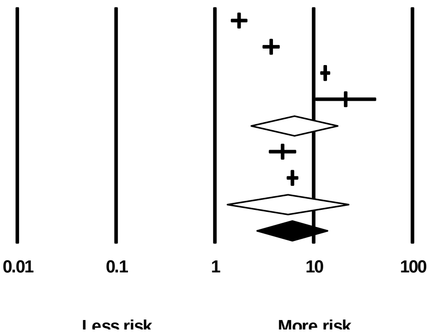

23 Meta-analysis on "Criminal history"

As can be seen, the overall effect for criminal history is both large and significant (OR 6.009) and represents the single largest effect size of all the meta-analyses carried out in T2.1. The effect on radicalization of action is even stronger (OR 6.321). The effect on radicalization of belief remains quite strong, although is slightly smaller than the overall effect (OR 5.441). These findings are quite interesting and indicate that previous criminality is associated with both radicalization of belief and radicalization of action but with a greater effect on radicalization of action. Given the nature of the data and the large heterogeneity identified by this analysis, additional moderator analyses were carried out by region. The only study which could not be easily grouped was the T2.7 study as no

This project has received funding from the European Union's Horizon 2020 research and innovation programme under grant agreement N° 699824.

other included studies provide effect sizes for radicalization of action in the EU. However, given that the Odds Ratio was so drastically different than all others and quite close to that from the Israeli study (Sela-Shayovitz, 2007), they were grouped together as "other" in order to separate out the two studies from the US.

Risk factor: Criminal history (by group and region)

Group by Region	Study name	Odds ratio	Lower limit	Upper limit	Z-Value	p-Value	Region	Group
EU	Bhui et-al (2014)	4.839	3.531	6.632	9.805	0.000	EU	Beliefs
EU	Coid et-al (2016)	6.104	5.354	6.958	27.056	0.000	EU	Beliefs
EU		5.473	3.070	9.760	5.761	0.000		
EU/IL	Weerman (2017)	13.090	11.727	14.612	45.833	0.000	EU/IL	Actions
EU/IL	Sela-Shayovitz (2007)	21.096	10.412	42.744	8.463	0.000	EU/IL	Actions
EU/IL		15.544	8.256	29.263	8.500	0.000		
US	LaFree (2017)	1.759	1.458	2.122	5.892	0.000	US	Actions
US	Jasko (2016)	3.710	3.051	4.512	13.134	0.000	US	Actions
US		2.552	1.443	4.514	3.221	0.001		
Overall		5.642	4.009	7.941	9.925	0.000		

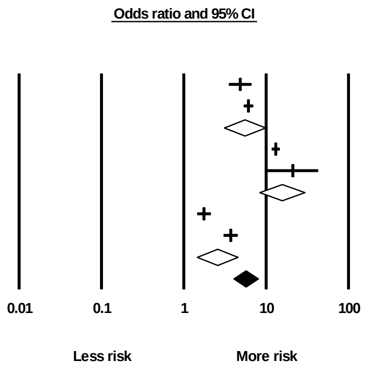

Figure 10 Criminal history 2

Overall, the pooled OR (5.642) is quite large, dropping only slightly from the previous analysis (OR 6.002) and it remains significant. However, the confidence limits tighten up considerably, making this a more reliable figure. Looking at the first group, the EU beliefs only studies' overall effect remains almost the same (OR 5.473). However, the most drastic differences exist between the action groups. For the two US studies, the OR drops to 2.552. While still significant, it is evident that for the US group, criminal history has a weaker effect than the overall pooled effect. The strong and similar effects from the EU and Israeli studies are evidently and significantly different. Combined, they have a pooled OR of 15.544, which is only slightly larger than the T2.7 study alone and which represents the single largest effect size identified anywhere in WP2.

In order to test the sensitivity here, an additional moderator analysis was carried out after having completely removed the two beliefs group studies as there was concern that their heterogeneity may have impacted the pooled TAU that is used to calculate the pooled OR. Nevertheless, the differences in the ORs were entirely insignificant and only miniscule.

This project has received funding from the European Union's Horizon 2020 research and innovation programme under grant agreement N° 699824.

Risk factor: Criminal history

Group by Region	Study name	Odds ratio	Lower limit	Upper limit	Z-Value	p-Value
Other	Lujic et-al (2017) (T2.7)	13.090	11.727	14.612	45.833	0.000
Other	Sela-Shayovitz (2007)	21.096	10.412	42.744	8.463	0.000
Other		15.821	7.456	33.572	7.194	0.000
US	LaFree (2017)	1.759	1.458	2.122	5.892	0.000
US	Jasko (2016)	3.710	3.051	4.512	13.134	0.000
US		2.553	1.275	5.111	2.647	0.008
Overall		5.906	3.546	9.836	6.823	0.000

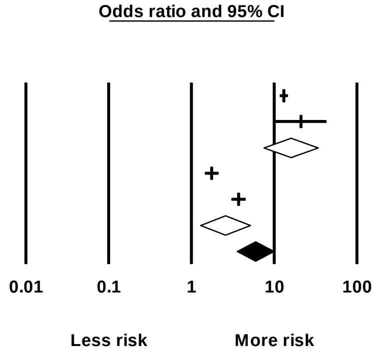

Horgan et-al (2016) found that 58% of terrorists in their sample had had criminal histories. While this was not significantly different to the comparison group, including between criminal offense type, it is important in the context of the findings of the meta-analysis. Nevertheless, Gruenwald (2016) found that unsuccessful attackers were more likely to have a criminal record compared to successful attackers. This may be the result of the fact that criminal records mean that potentially violent radicals are already on the radar of security forces as a result of having passed through the criminal justice system. Gill et-al (2014) found that Al Qaeda inspired and single issue terrorists were more likely than right-wing terrorists to have a criminal record. Overall, of 119 Lone Actors from the US and EU between 1990 and 2012, 40% of them had prior criminal records (Gill et-al, 2014).

This project has received funding from the European Union's Horizon 2020 research and innovation programme under grant agreement N° 699824.

2.8.15 Collective relative deprivation

In this analysis we found a moderate and significant effect overall. For radicalization of beliefs, the effect is somewhat smaller, albeit still significant (OR 1.792). However, for the two studies on self-reported radical action, collective relative deprivation had a strong and significant pooled effect (OR 3.609). The differences between these groups was also significant (*p <.05)*.

Risk factor: Collective relative deprivation

Group by Group	Study name	Odds ratio	Lower limit	Upper limit	Z-Value	p-Value
Actions	Pauwells & Schils (2016)	2.809	2.554	3.090	21.241	0.000
Actions	Pauwells & De Waele (2014)	4.670	4.043	5.394	20.970	0.000
Actions		3.609	2.293	5.679	5.548	0.000
Beliefs	Nivette et-al (2017)	1.156	0.942	1.419	1.391	0.164
Beliefs	Beller & Kröger (2017)	1.428	1.307	1.559	7.920	0.000
Beliefs	Zhirkov et-al (2014)	1.942	1.607	2.348	6.857	0.000
Beliefs	Feddes et-al (2015)	2.015	0.668	6.081	1.244	0.214
Beliefs	Ellis et-al (2014)	3.668	2.478	5.429	6.496	0.000
Beliefs		1.792	1.295	2.478	3.524	0.000
Overall		2.271	1.744	2.956	6.094	0.000

Odds ratio and 95% CI

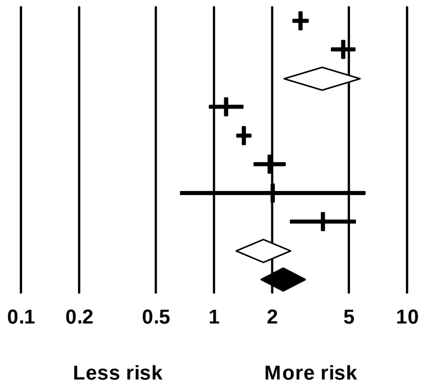

Figure 11 Collective relative deprivation

In examining only the beliefs group, an additional analysis was carried out in which the moderator was region. While the overall effects for the beliefs group were not so different to the first analysis (OR 1.718), there was a significant difference (*p=.004)* between the EU (OR 1.475) and US (OR 3.294) beliefs groups.

Risk factor: Collective relative depirvation

Group by Region	Study name	Odds ratio	Lower limit	Upper limit	Z-Value	p-Value
EU	Nivette et-al (2017)	1.156	0.942	1.419	1.391	0.164
EU	Beller & Kröger (2017)	1.428	1.307	1.559	7.920	0.000
EU	Zhirkov et-al (2014)	1.942	1.607	2.348	6.857	0.000
EU		1.475	1.161	1.874	3.186	0.001
US	Feddes et-al (2015)	2.015	0.668	6.081	1.244	0.214
US	Ellis et-al (2014)	3.668	2.478	5.429	6.496	0.000
US		3.294	2.009	5.403	4.723	0.000
Overall		1.718	1.385	2.131	4.924	0.000

Odds ratio and 95% CI

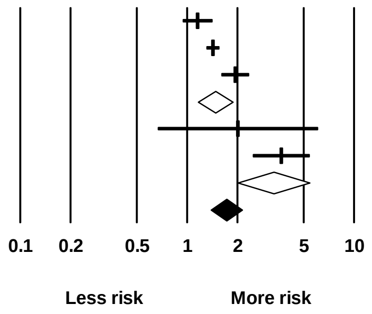

This project has received funding from the European Union's Horizon 2020 research and innovation programme under grant agreement N° 699824.

Collective relative deprivation is figures as one of the most prominent risk factors examined in the wider literature. Van Bergen et-al (2015, 2016) examined the risk factors for willingness to use violence for in-group defence. Collective relative depravation was the only significant direct path in this study (*$p<.001$).* It is important to consider however that in two of the studies (Van Bergen et-al, 2016; Zaidise, 2009) the constructs used for "collective relative deprivation" consisted of a combination of variables that have otherwise been examined separately in the literature. For example, while "objective deprivation" was a composite measure of education, personal, and household income, "subjective deprivation" was a composite of being a recipient of marginalization and discrimination.

Nevertheless, these studies also provide important evidence and do so in the context of comparing two different religious groups. They found that being religious Muslim and objectively deprived was the strongest pathway to supporting political violence. Conversely, the path from religiosity to subjective deprivation was quite weak in comparison, both directly and indirectly. Doosje et-al (2012) and Doosje et-al (2013) found that collective relative deprivation was one of the strongest correlates for both right-wing and Muslim youth in the Netherlands with respect to radical belief and willingness to use violence. In another study, Van der veen (2016) examined a US (179) sample and one EU (n=183) sample. Collective relative deprivation was found to be an especially important and significant correlate for the EU study.

This project has received funding from the European Union's Horizon 2020 research and innovation programme under grant agreement N° 699824.

Religion and politics

This review identified a total of five (5) variables that could be categorized as being "religious" variables in nature and definition and an addition two (2) variables related to "politics". In many studies identified by T2.1 these variables were grouped together and in general they are closely linked conceptually. Indeed, many survey based studies find evidence to support this approach as it is a widely held position that radicalization is inherently linked with religious identity and observance on the one hand, and local and foreign politics on the other. Perhaps not surprisingly, studies that examine such variables are almost entirely from the radicalization of beliefs group only, since it is quite difficult for information to be gathered on issues of identity and practice from deceased or incarcerated individuals who have engaged in radical actions.

2.8.16 Politics

As such, with regards to politics T2.1 identified a total of four (4) studies which examined questions pertaining to politics in the sense of perceptions of foreign involvement in external conflicts. For example, in Cherney and Povey's (2013) study, the 'politics' variable was constructed by questions pertaining to Barak Obama's conduct in the arena of foreign policy. In Acevedo and Chaudery's (2015) study, foreign policies, such as US involvement in Iraq and Afghanistan were used to construct the independent variables. Similarly, McCauley's study of the pew report examined opinions of American Muslims with respect to US involvement in Afghanistan and how such perceptions were correlated with radical belief in the form of support for terrorism. In the fifth study, Zhirkov et-al (2015) examined the issue by constructing a variable that examined respondents' attribution of responsibility for conflict on the Western world. It is clear that these studies examined the issue of 'politics' in a conceptually similar and fairly consistent manner that also reflects the general debate over the role of politics and foreign interventions in radicalization in western and democratic countries.

Of the studies included here and coded for meta-analysis there are some important differences however. For example, Cherney and Povey (2013) examine 8,003 respondents to the Pew survey rom 7 predominantly Muslim countries. However, T2.1's review protocol pre-determined that studies which examine multiple countries but which include at least one democratic country would be

This project has received funding from the European Union's Horizon 2020 research and innovation programme under grant agreement N° 699824.

considered to have met the inclusion criteria where they also meet all other criteria. As such, this study's inclusion of Indonesia would have it meet such criteria. Both the studies by Acevedo and MaCauley (2012) also use the Pew report data in their examinations of the issue but limit their analyses to the US only. The Zhirkov (2014) study also uses data from the 2006 Pew report but compares between 15 countries, grouping the countries into either "Muslim" or "Western Europe" categories. Whilst all studies included in this analysis thus use the same primary data source, they examine the results from different years, different countries, and utilize different statistical methods. Only the Zhirkov et-al (2014) study explicitly sets out to examine how perception of a variety of political issues impact radicalization of belief. The overall effects of this risk factor were both moderate and significant (OR 2.328). However there are apparently much stronger effects for the US group than the "other" countries group and these differences are significant (*p*<.05).

Risk factor: Politics

Group by Region	Study name	Statistics for each study				
		Odds ratio	Lower limit	Upper limit	Z-Value	p-Value
Other	Cherney & Povey (2013)	1.530	1.405	1.667	9.741	0.000
Other	Zhirkov et-al (2014)	2.038	1.865	2.227	15.733	0.000
Other		1.766	1.237	2.519	3.135	0.002
US	MaCauley et-al (2012)	2.269	1.811	2.842	7.129	0.000
US	Acevedo et-al (2015)	4.801	3.667	6.286	11.408	0.000
US		3.257	2.202	4.817	5.913	0.000
Overall		2.328	1.790	3.029	6.296	0.000

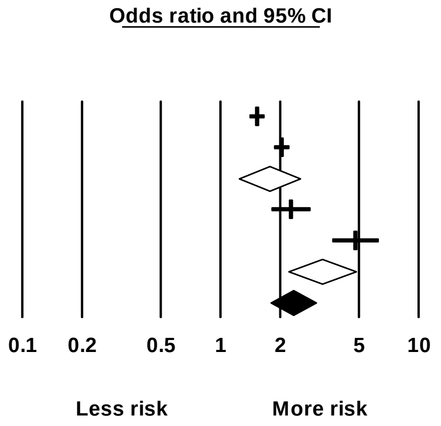

Figure 12 Politics

Despite the potential bias that arises from the four studies all having used the same set of datasets, the results of the random effects model meta-analysis provide potentially important direction. Between the two studies examining other countries, Zhirkov et-al's (2014) study, which examines Western European countries, has an effect size (OR 2.038) that is much closer to the overall effect, while the Cherney and Povey (2013) study examining Muslim majority countries reports the smallest effect. The issue of "politics" needs to be understood as something specific to different groups in different contexts who may feel differently towards a variety of foreign conflicts in which their home countries (or countries of residence) will be involved in in different ways. For example, the

This project has received funding from the European Union's Horizon 2020 research and innovation programme under grant agreement N° 699824.

involvement of the US in a greater number of conflicts, over a greater period of time and for which greater total casualties and damages have arguably resulted, could explain the larger impact of politics of radicalization of opinion of American Muslims compared to their European counterparts as well as those of Muslims in countries who are not directly affected by such conflicts.

2.8.17 West vs. Islam

The issue of holding a belief that there is a perpetual and ongoing war between the West and Islam may not necessarily be connected to the actual ongoing conflicts between Western and Islamic countries. For example, right-wing extremists may believe that there is a war being waged by the liberal governments against the white race or culture. Nevertheless, such beliefs may be distinct from views on how home countries may be operating overseas. For example, although two (2) of the studies examined here also provided effect sizes for the above variable of "politics", West Vs. Islam was examined separately and distinctly in these studies. In one of the additional studies examined here, Beller and Kroeger (2017) examined the issue specifically and explicitly, findings a strong correlation between beliefs in an ongoing war between West Vs. Islam and radicalization of beliefs. They used data pertaining to a number of countries from Eastern Europe and the Islamic world. In the other additional study, Pedersen et-al (2017) examine the issue through a survey of youth in Norway. One of the advantages of their analysis is that their data includes responses from both Muslims and Christians (as well as 'no religion' individuals).

Unfortunately, there were no two studies which specifically examined this risk factor in the same country or grouping of countries that would enable moderator analyses to be conducted. Nevertheless, the results are still informative. The overall effect size is both moderate and significant (OR 2.238). It is interesting to note the closeness of the pooled OR between this variable and the "politics" variable, especially where they place relative to other variables.

This project has received funding from the European Union's Horizon 2020 research and innovation programme under grant agreement N° 699824.

Risk factor: West Vs. Islam

Study name	Odds ratio	Lower limit	Upper limit	Z-Value	p-Value
Cherney & Povey (2013)	1.123	1.032	1.223	2.675	0.007
Pedersen et-al (2017)	2.156	1.993	2.331	19.238	0.000
Beller et-al (2017)	3.159	2.881	3.463	24.505	0.000
Acevedo et-al (2015)	3.358	2.587	4.358	9.103	0.000
	2.238	1.360	3.680	3.172	0.002

Odds ratio and 95% CI

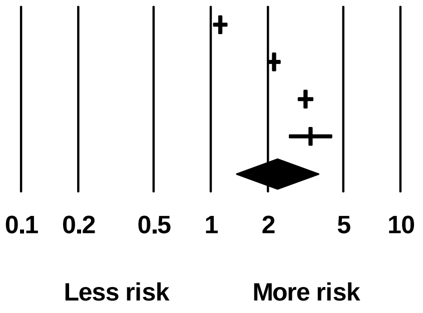

Figure 13 Meta-analysis on "West Vs. Islam"

This project has received funding from the European Union's Horizon 2020 research and innovation programme under grant agreement N° 699824.

2.8.18 RELIGIOUS IDENTITY

Religious identity, belief and practice is not always connected to political beliefs, nor with each other. Many individuals may firstly identify as Muslim, Christian, Jewish or other but not actually be overly religious in terms of their practice of beliefs. It is for this reason that identity is such an interesting variable. In the study by Simon et-al (), dual identity can be shown to be an important determinant of radical belief. In this analysis, 5 studies examine religious identity explicitly and separately from other religious related variables. Only of them, Pauwells and Schills (2016) examines self-reported actions. The fact that this study's reported effect size is quite different and larger than those of the other four (4) included studies demonstrates some of the important differences that risk factors play between beliefs and actions. Nevertheless, the overall OR is 1.611, represents a small but significant effect which appears to be more in line with the individual effect sizes observed from the beliefs group studies.

Risk factor: Religious identity

Group by Group	Study name	Statistics for each study				
		Odds ratio	Lower limit	Upper limit	Z-Value	p-Value
Actions	Pauwells & Schils (2016)	3.397	3.083	3.742	24.777	0.000
Actions		3.397	2.462	4.686	7.449	0.000
Beliefs	Cheryney & Murphey	1.037	0.806	1.334	0.282	0.778
Beliefs	Cherney & Povey (2013)	1.160	1.066	1.263	3.427	0.001
Beliefs	Pederson et-al (2017)	1.459	1.351	1.576	9.623	0.000
Beliefs	Zhirkov et-al (2014)	1.581	1.449	1.726	10.240	0.000
Beliefs		1.319	1.117	1.558	3.257	0.001
Overall		1.611	1.390	1.868	6.318	0.000

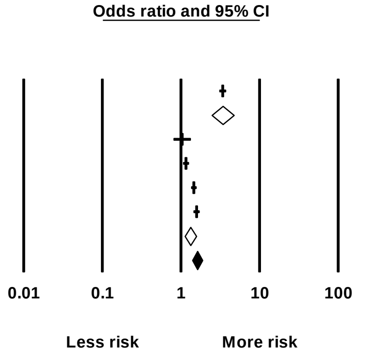

Figure 14 Religious identity

An additional analysis analysing only the beliefs group found only a slight change and decrease in the pooled OR (1.319).

This project has received funding from the European Union's Horizon 2020 research and innovation programme under grant agreement N° 699824.

Risk factor: Religious identity

Study name	Odds ratio	Lower limit	Upper limit	Z-Value	p-Value
Cheryney & Murphey	1.037	0.806	1.334	0.282	0.778
Cherney & Povey (2013)	1.160	1.066	1.263	3.427	0.001
Pederson et-al (2017)	1.459	1.351	1.576	9.623	0.000
Zhirkov et-al (2014)	1.581	1.449	1.726	10.240	0.000
	1.319	1.117	1.558	3.257	0.001

(Columns Odds ratio to p-Value: Statistics for each study)

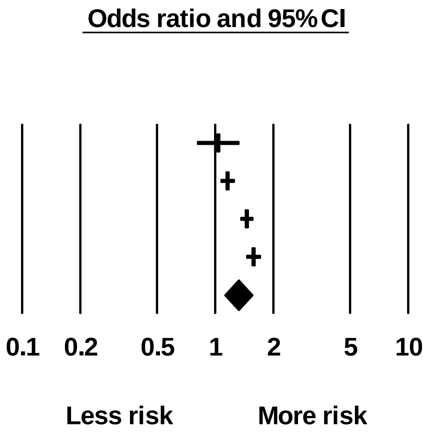

Figure 15 Religious identity 2

This project has received funding from the European Union's Horizon 2020 research and innovation programme under grant agreement N° 699824.

2.8.19 Religiosity

With the rise of religious inspired attacks in recent years, there has been a growing debate about the extent to which "religiosity" plays a role in radicalization. While this may seem intuitive to some that it does play an important role, it is important to note that indeed, many terrorists were not overly religious by the time of their attacks nor in the time leading up to them. There is however an issue with examining religiosity per se, specifically with respect to issues of measurement and construction of the variable. Self reported religiosity may or may not be primarily related to frequency of attendance of services and frequency of prayer. However, there are many other religious practices and forms of religious observance that may be used to determine or measure religioisity. Indeed, as will be seen in this section, many of the same studies examine both religiosity, as well as other forms of practice, such as frequency of attendance at places of worhip, and prayer.

Only two of the studies carried over from the above examination of religious identity and examine these as serparate individual variableds. In the current analysis of five (5) studies, the overall pooled OR is quite large and significant (OR 3.603). Again, the inclusion of an actions group study by Pauwells et-al (2017) represents a clear and identifable outlier which once again demonstrates the differing effects that risk factors may have between belies and actions.

Risk factor: Religiosity

Group by Group	Study name	Odds ratio	Lower limit	Upper limit	Z-Value	p-Value
Actions	Pauwells et-al (2017)	13.207	11.224	15.541	31.088	0.000
Actions		13.207	6.652	26.224	7.375	0.000
Beliefs	MaCauley et-al (2012)	1.611	1.290	2.010	4.215	0.000
Beliefs	Beller et-al (2017)	2.364	2.160	2.587	18.709	0.000
Beliefs	Acevedo et-al (2015)	2.950	2.279	3.819	8.214	0.000
Beliefs	Cherney (2013)	3.195	2.922	3.494	25.507	0.000
Beliefs	Pedersen et-al (2017)	4.437	4.085	4.820	35.284	0.000
Beliefs		2.779	2.045	3.777	6.531	0.000
Overall		3.603	2.723	4.768	8.973	0.000

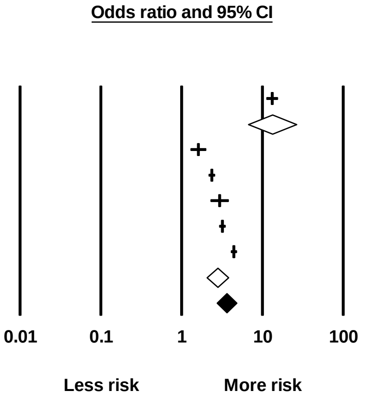

24 Meta-analysis on "Religiosity"

This project has received funding from the European Union's Horizon 2020 research and innovation programme under grant agreement N° 699824.

As such, an additional analysis was carried out after having removed the Pauwells et-al (2017) study and instead, moderating for region. In this case, only the Pederson et-al (2017) study examined the variable in an EU context and was unable to be grouped with any other studies. However, two studies, McCauley (2012) and Acevedo (2015) both examine in the US. Whilst grouping the non-US studies together is not ideal, it does provide for some degree of disaggregation. The removal of the actions group study does cause a significant decrease in the pooled OR (2.781), although it still represents a strong-moderate and significant effect. The effect for the US only studies is slightly smaller (OR 2.167). However, the other countries group's pooled OR (3.225) is considerably larger and not so different than the overall OR of the first analysis.

Risk factor: Religiosity

Group by Region	Study name	Statistics for each study				
		Odds ratio	Lower limit	Upper limit	Z-Value	p-Value
Other	Beller et-al (2017)	2.364	2.160	2.587	18.709	0.000
Other	Cherney (2013)	3.195	2.922	3.494	25.507	0.000
Other	Pedersen et-al (2017)	4.437	4.085	4.820	35.284	0.000
Other		3.225	2.232	4.660	6.237	0.000
US	MaCauley et-al (2012)	1.611	1.290	2.010	4.215	0.000
US	Acevedo et-al (2015)	2.950	2.279	3.819	8.214	0.000
US		2.167	1.344	3.494	3.174	0.002
Overall		2.781	2.078	3.723	6.878	0.000

Odds ratio and 95% CI

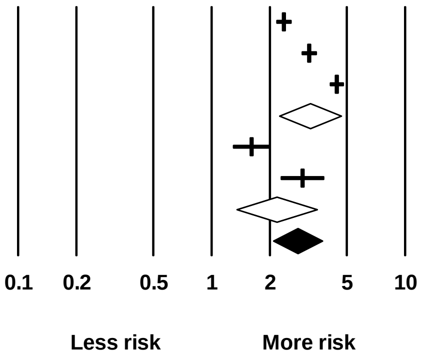

25 Meta-analysis on "Religiosity" (Beliefs group only)

The results of this analysis are also difficult to interpret. While on face value they may seem to indicate that religiosity is less of a risk factor in the US than it is elsewhere in the context of radicalization of belief, the apparent differences between these groups are actually not significant (p= .196).

This project has received funding from the European Union's Horizon 2020 research and innovation programme under grant agreement N° 699824.

2.8.20 Frequency of attendance at place of worship

It is of great importance to understand the nature and impact of attendance to places of worship, especially in light of the ongoing debates and protests that surround the construction of new facilities. It is of course important to distinguish frequency of attendance from prayer frequency since not all prayers are conducted at places of worship and not all visits to places of worship are necessarily for the purpose of prayer. Religiously observant and devoted individuals may have to pray multiple times a day and at specific times and so they are unlikely to attend a place of worship on each occasion. Additionally, visits to places of worship may be for reasons of community meetings or other communal activities or non-prayer related groups and activities, including lectures and other events. It is perhaps with such understandings that most studies examine frequency of attendance on a weekly, monthly, or more seldom basis (as opposed to daily).

In this analysis a total of six (6) studies were identified which examine the effects of frequency of attendance on radical beliefs and a further two (2) studies that examine the effects on self-reported radical action. Half of the belief groups studies examined the issue with regards to Muslims exclusively, whereas the others studies examine multiple religions. Of those studies examining radical actions, for example, multiple religions were included, as well as respondents who reported themselves as having no religion. As can be seen in the below analysis, the overall effects of frequency of attendance at places of worship have a moderate-strong and statistically significant effect (OR 2.458). The overall effect is only slightly smaller for the beliefs only group (OR 2.274) and marginally larger for the self-reported actions group (3.087), however there is no statistically significant differences between the groups (p=.427).

This project has received funding from the European Union's Horizon 2020 research and innovation programme under grant agreement N° 699824.

Risk factor: Place of worship attendance (frequency)

Group by Group	Study name	Odds ratio	Lower limit	Upper limit	Z-Value	p-Value
Actions	Pauwells & Schills (2016)	2.053	1.870	2.254	15.086	0.000
Actions	Pauwells et-al (2017)	4.670	4.043	5.394	20.970	0.000
Actions		3.087	1.609	5.923	3.391	0.001
Beliefs	Cherney & Murphey (2017)	1.075	0.836	1.383	0.565	0.572
Beliefs	MaCauley et-al (2012)	1.387	1.112	1.729	2.904	0.004
Beliefs	Ginges et-al (2009)	2.203	1.981	2.449	14.584	0.000
Beliefs	Bhui et-al b (2014b)	2.950	2.182	3.989	7.030	0.000
Beliefs	Beller et-al (2017)	3.064	2.796	3.359	23.914	0.000
Beliefs	Coid et-al (2016)	4.472	3.939	5.077	23.140	0.000
Beliefs		2.274	1.553	3.329	4.220	0.000
Overall		2.458	1.769	3.417	5.355	0.000

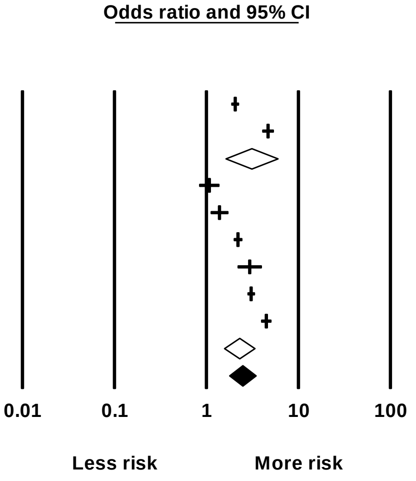

26 Meta-analysis on "Place of worship attendance"

Although most of the studies examining this issue tend to focus on Muslims, some of the studies include examining the variable in the context of up to 7 different religious (IE Coid et-al, 2016). In a study of 2787 Britons of different religious and ethnic backgrounds, Oskooii & Dana (2017) found that attendance frequency was negatively associated with participation in and support for violent processes. Unfortunately, the methodology used in this study prohibited its inclusion in the meta-analysis. Nevertheless, it is evident that the character of each individual place of worship will have a different impact. For example, Cherney & Murphey's (2017) first model found that frequency of attendance had a negative effect on radical beliefs but in their final model there was a positive effect, although it was not statistically significant. A secondary analysis examining only the radical beliefs group found that the overall effect size remained relatively unchanged (OR=2.281), however important differences were found between region. The OR of 3.679 for the EU is considerably larger than the 1.809 for the "other" countries, with these differences being significant ($p < 0.05$).

This project has received funding from the European Union's Horizon 2020 research and innovation programme under grant agreement N° 699824.

Risk factor: Place of worship attendance (frequency)

Group by Region	Study name	Odds ratio	Lower limit	Upper limit	Z-Value	p-Value
EU	Bhui et-al b (2014b)	2.950	2.182	3.989	7.030	0.000
EU	Coid et-al (2016)	4.472	3.939	5.077	23.140	0.000
EU		3.679	2.119	6.389	4.626	0.000
Other	Cherney & Murphey (2017)	1.075	0.836	1.383	0.565	0.572
Other	MaCauley et-al (2012)	1.387	1.112	1.729	2.904	0.004
Other	Ginges et-al (2009)	2.203	1.981	2.449	14.584	0.000
Other	Beller et-al (2017)	3.064	2.796	3.359	23.914	0.000
Other		1.809	1.232	2.656	3.026	0.002
Overall		2.281	1.664	3.126	5.126	0.000

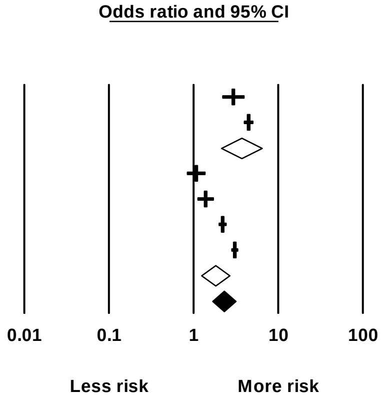

27 Meta-analysis on "Place of worship attendance frequency" (Beliefs group only) moderated by region

The overall effect size drops only slightly to 2.281. However, the pooled effects from the two EU based studies are quite large and significant (OR 3.679), especially in comparison to the much smaller effects of the combined studies from "other" countries (OR 1.809), with such differences being statistically significant at the .05 level (p=.039). Both studies in this group (Bhui et-al, 2014b; Coid et-al, 2016) were carried out by the same group of authors using different samples from the UK. Overall, in the context of the EU, there is a similar effect size for place of worship attendance on radicalization of belief and radicalization of action.

This project has received funding from the European Union's Horizon 2020 research and innovation programme under grant agreement N° 699824.

2.8.21 PRAYER FREQUENCY

As discussed above, there is certainly a difference in prayer frequency and attendance at places of worship. Additionally, prayer frequency is only something which can be truly gauged through self-reports, and this is reflected by the fact that none of the studies included here examine radicalization of action. Indeed, we lose 5 of the studies which reported effect sizes for the previous risk factor and are only able to add one new study in this analysis.

Overall, the pooled OR shows a large and significant effect of prayer frequency on radicalization of beliefs (3.609), similar to the identified effect of overall religiosity, of which prayer frequency is most likely a part. The overall OR is also considerably larger than it is for frequency of attendance, perhaps underscoring the fact that personal engagement in prayer is not always associated with attendance at a place of worship.

Risk factor: Prayer frequency

Study name	Statistics for each study				
	Odds ratio	Lower limit	Upper limit	Z-Value	p-Value
Beller et-al (2017)	2.767	2.526	3.031	21.900	0.000
Acevedo et-al (2015)	3.739	2.874	4.865	9.820	0.000
Coid et-al (2015)	3.932	3.469	4.457	21.409	0.000
Cherney (2013)	4.217	3.849	4.621	30.843	0.000
	3.609	2.856	4.561	10.750	0.000

Odds ratio and 95% CI

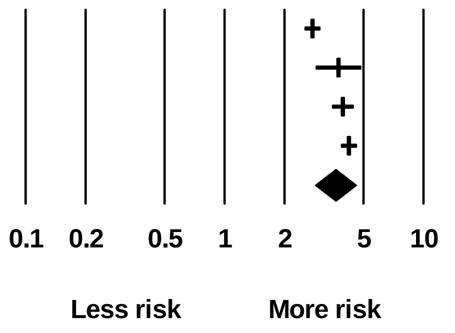

28 Meta-analysis on "Prayer frequency"

It is important to note that notions about prayer frequency and attendance need to be understood in context, since the most frequent attendees and engagers in prayer are most commonly not radicals and will never become terrorists. Nevertheless, actual frequency of engagement in religious rites and practices appears to have a stronger effect on radicalization of belief than only religious identity.

This project has received funding from the European Union's Horizon 2020 research and innovation programme under grant agreement N° 699824.

2.8.22 Religious fundamentalism

Religious fundamentalism is arguably the most important of the religious related variables. This is also because in many respects, religious fundamentalism and radical beliefs may be indistinguishable. Nevertheless, religious fundamentalism doesn't necessarily justify, support or engage in violence. Most traditionalists who would score high in religious fundamentalism are still unlikely to ever engage in radical action and violence. In this analysis religious fundamentalism was found to have a strong and significant overall effect (OR 4.020). The effect was stronger for radicalization of beliefs (4.384) and only slightly less strong for self-reported radical action (OR 3.251) but the differences were not significant (p=0.562).

Risk factor: Religious fundamentalism

Group by Group	Study name	Statistics for each study				
		Odds ratio	Lower limit	Upper limit	Z-Value	p-Value
Actions	Pauwells & Schills (2016)	2.928	2.661	3.222	22.028	0.000
Actions	Pauwells et-al (2014)	3.612	3.138	4.157	17.896	0.000
Actions		3.251	1.386	7.625	2.710	0.007
Beliefs	Acevedo et-al (2015)	1.407	1.097	1.804	2.694	0.007
Beliefs	Beller et-al (2017)	2.139	1.956	2.340	16.632	0.000
Beliefs	Cherney (2013)	4.995	4.551	5.481	33.900	0.000
Beliefs	Cherney & Murphey (2017)	9.647	7.168	12.983	14.960	0.000
Beliefs	Delia Deckard (2015)	11.569	9.029	14.824	19.355	0.000
Beliefs		4.384	2.542	7.560	5.317	0.000
Overall		4.020	2.540	6.362	5.939	0.000

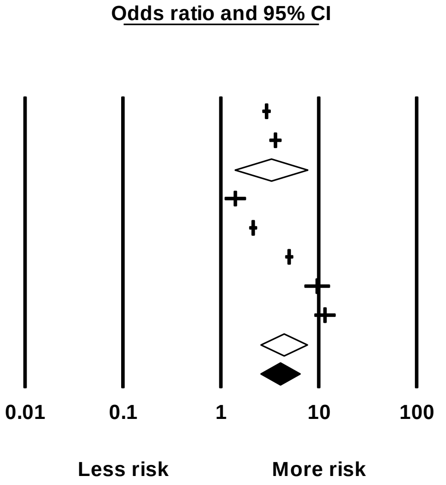

29 Meta-analysis on "Religious fundamentalism"

Understanding the nature and effect of religious fundamentalism may be one of the most important areas that research should focus on. Fundamentalism is inherently a "radical" ideology according to most western interpretations and perspectives and it is therefore not surprising that the effect on beliefs is so strong. However, as demonstrated by Delia-Deckard (2015), a number of the risk factors identified in this review as being relatively weaker risk factors for radicalization itself, such as socio-economic and demographic factors, may be strong predictors for religious fundamentalism. As such, some of these factors may have indirect effects on radical outcomes. In independent studies by Muluk et-al (2013) and Mushuri et-al (2016), religious fundamentalism was found to be the single greatest predictor of belief in violent jihad among two samples from Indonesia. Whilst Indonesia is a

This project has received funding from the European Union's Horizon 2020 research and innovation programme under grant agreement N° 699824.

democratic country, it is also a predominantly Muslim country, and the largest one for that matter. So whilst Indonesia may not be comparable to other democratic states, the similarities in findings with regards to religious fundamentalism are nevertheless informative regarding this risk factor.

2.8.22.1 Right-wing authoritarianism, social dominance orientation, and religious fundamentalism

During the course of this systematic review, a number of items were identified from the field of Social Dominance Orientation (SDO) and Right Wing Authoritarianism (RWA) research. It was identified that some researchers have used the RWA and SDO instruments to examine religious fundamentalism and radicalization. For example, Limieux et-al (2010), examined extremism using the RWA and SDO framework. In this quasi-experimental study, high SDO, personal grievance, and gender predicted willingness to engage in terrorism. Support for terrorism was also predicted by SDO, RWA, grievance and age. The most recent study, Kruglanski et-al (2017) use SDO in their most recent research into radicalization and de-radicalization in prisons. Given that the review pointed to religious fundamentalism as being one of the strongest risk factors, examining the relevance of RWA and SDO research may provide important insights and direction for future research. Some SDO and RWA instruments have been used to assess extremist ideologies that lead to support for extremist violence and propensity for involvement in both Palestinians and Israelis. In this regard, Rubinstein (1996) found that such support (radicalization of belief) was connected with a religious extremism that was assessed with these instruments. According to Besta et-al (2014), one can also approach the issue from the point of Left Wing Authoritarianism (LWA), as in the case with identity fusion and violence.

Crossing the line from RWA research to religious fundamentalism research is not new however. In one of the more interesting studies, Jakubowska & Oniszczenko (2010) conducted a Twins study on "religious fundamentalism". They found that religious fundamentalism was only 38% inheritable and that overall it was the result of environmental factors. However, the single greatest predictor of fundamentalism was trait anxiety, and trait anxiety itself is 60% heritable. Nevertheless, the other predictor was 'anxiety and beliefs about the nature of the social world.

This project has received funding from the European Union's Horizon 2020 research and innovation programme under grant agreement N° 699824.

> "*This finding confirms the hypothesis that individual, unique personal experience acquired in the family, as well as in peer groups, religious organisations etc., help to shape the individual level of religious fundamentalism"* (p. 260).

These findings appear to be in line with much of what we know about the effects of differential associations, attendance of religious establishments etc. and as described by the above meta-analyses. Additionally, quasi experimental and longitudinal studies in RWA and SDO research may have important implications for radicalization and counter-radicalization research. For example, Asbrock et-al (2010) and Asbrock et-al (2012) have found that RWA and SDO predict out-group prejudice, whereas contacts with members of outgroups reduce RWA and SDO. These findings appear to be in line with those of Bhui et-al regarding the differential effects of in-group and out-group peers. Additionally, other studies in radicalization research, including those included in this review, find out-group interactions and friendships to be important protective factors.

Additionally, Corson et-al (1997) found that education and socio-economic status among other variables strongly predict RWA and SDO. In a more recent study, Muranyi (2011) found that lower socio-economic status was also associated with greater RWA and SDO. This is in line with the findings of Delia-Deckard (2015) as mentioned above. Croson (1997) reports that verbal intelligence predicts the likelihood of youth scoring high on RWA and SDO.

Based on a review of these studies there appears to be good validity to the notion that RWA research can be used to approach at the least, an equivalent to religious fundamentalism, which is itself one of the strongest predictors of radicalization of belief. With much discussion over the years about so called "root causes" having been taken over by "drivers", and now with the shift to "risk factors of radicalization", the roots of religious fundamentalism and its psychological components may have been overlooked out of political concerns. It seems that the underpinning of RWA and SDO research have direct relevance to radicalization research and could assist in providing more validated instruments for use in survey and experiment based research, especially longitudinal designs for which it is well suited.

This project has received funding from the European Union's Horizon 2020 research and innovation programme under grant agreement N° 699824.

2.8.23 OTHER RISK FACTORS:

While the above meta-analyses cover a wide range of those risk factors most commonly examined, there are of course additional factors which need to be taken into consideration. A number of studies have identified that symbolic and real threat may be important risk factors for radicalization of belief (Van der Veen, 2016a; Doosje et-al, 2012, 2013; Mushuri et-al, 2016). There is also the issue of in-group superiority, which may be closely related to religious identity in many cases (Van Bergent et-al, 2015, 2016), as well as justice and significance seeking (Van der veen, 2016a; Doosje et-al, 2012, 2013).

Other studies have identified that personal grievances, especially when they include anger and contempt (Tausch et-al, 2009) have an influence on radicalization of beliefs. Additionally, a range of different life events such as personal loss, or personal grievance could be important risk factors for radicalization (Bhui et-al, 2014), or even important triggers that may distinguish violent from non-violent radicals (Jasko et-al, 2016).

This project has received funding from the European Union's Horizon 2020 research and innovation programme under grant agreement N° 699824.

2.8.24 QUANTIFYING THE TWO PYRAMID MODEL

The quantitative findings of any meta-analysis are open to much interpretation. Nevertheless, this is the first attempt to provide some form of quantitative reconciliation of the evidence and to systemise a large number of risk factors under a theoretical framework. The results from the meta-analyses are able to be used to quantify the two pyramid model of radicalization.

This project has received funding from the European Union's Horizon 2020 research and innovation programme under grant agreement N° 699824.

2.8.24.1 Radicalization of belief

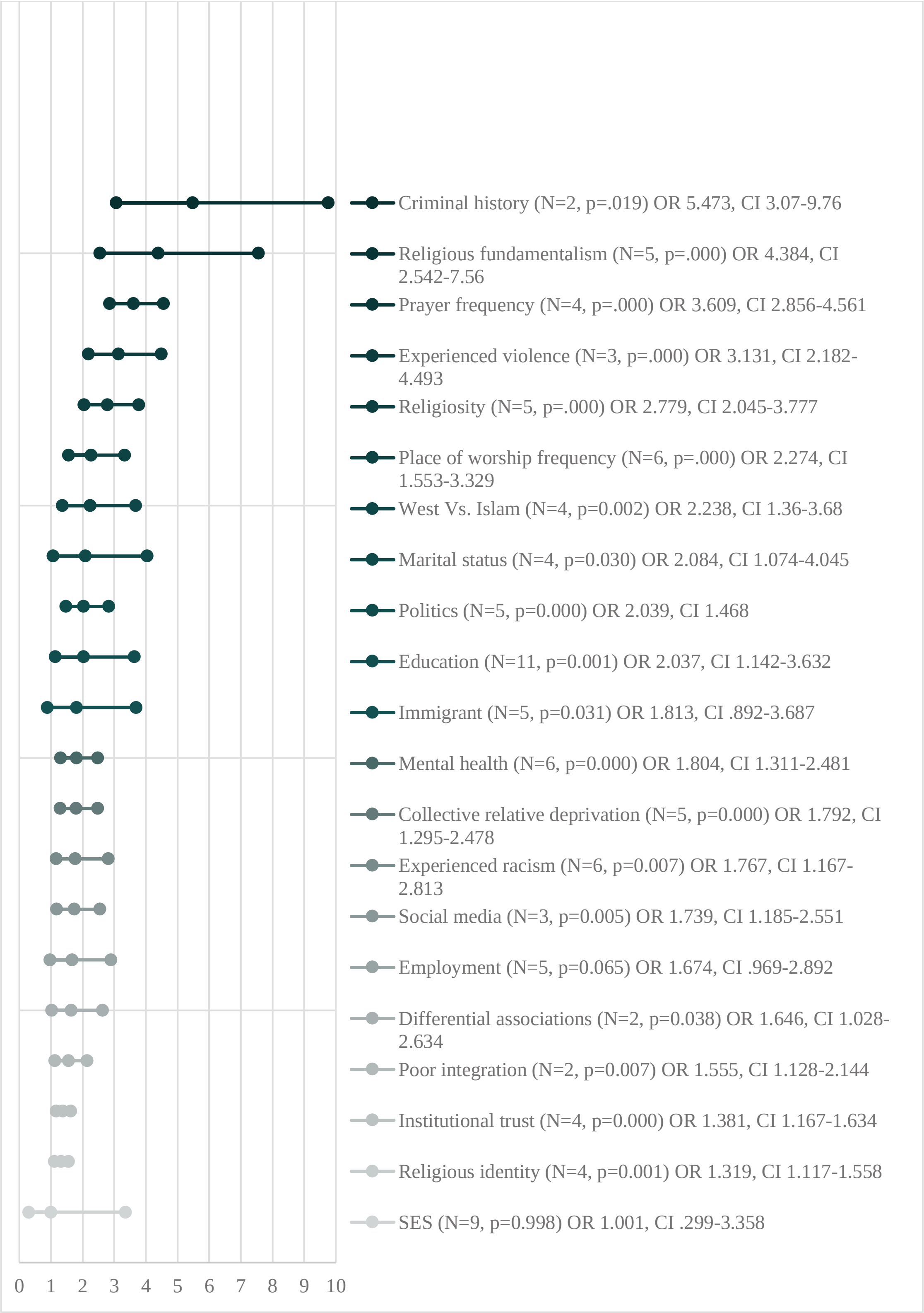

30 Radicalization of belief pyramid

This project has received funding from the European Union's Horizon 2020 research and innovation programme under grant agreement N° 699824.

2.8.24.2 Radicalization of action

For radicalization of action moderator effect sizes produced an 11 factor model. The model differs in important respects from the radicalization of action pyramid, identifying poor integration, collective relative deprivation, differential associations and institutional trust as having greater effects on radicalization of action.

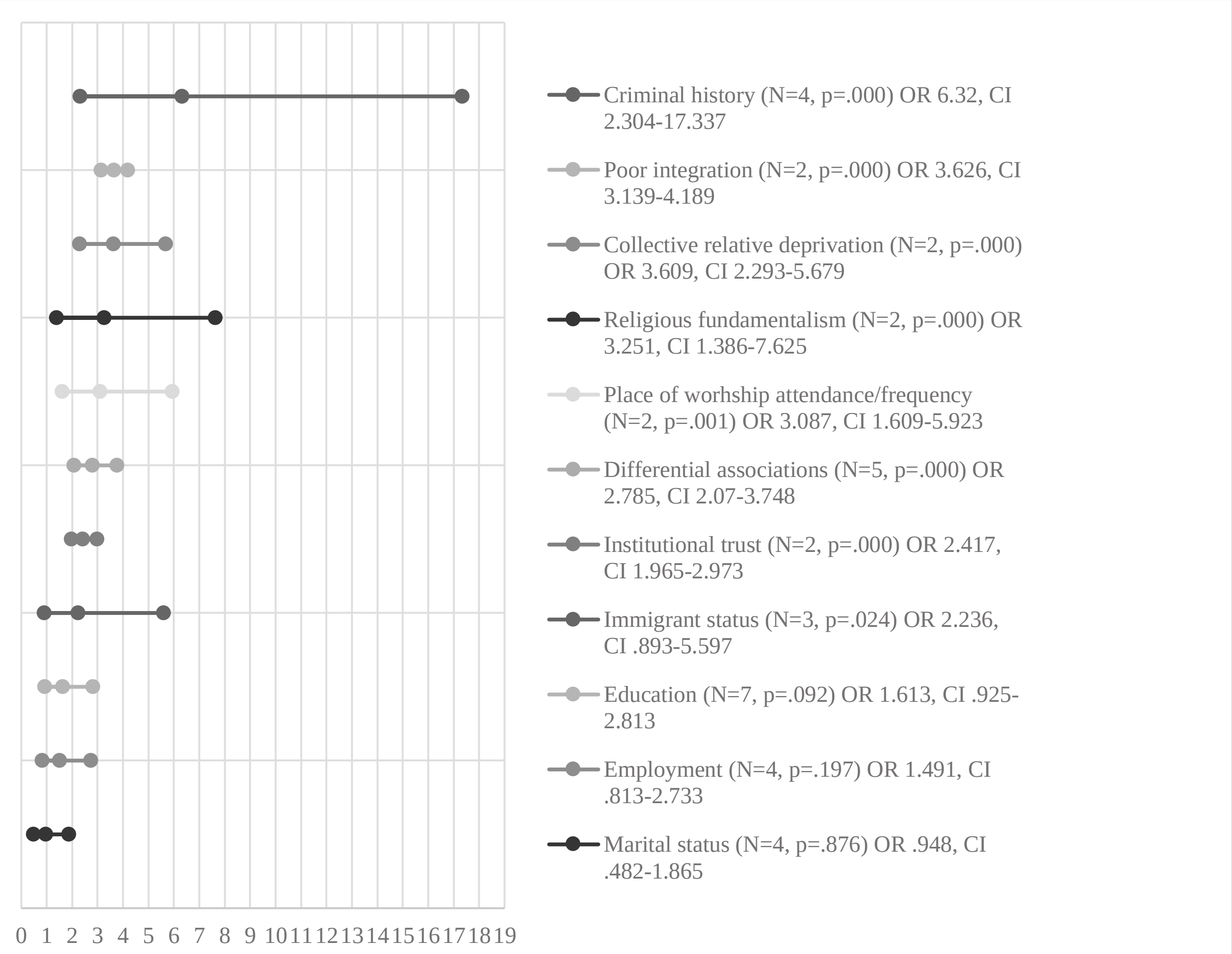

31 Radicalization of action pyramid

Other factors such as religious fundamentalism and criminal history appear as strong risk factors across all radical groups.

This project has received funding from the European Union's Horizon 2020 research and innovation programme under grant agreement N° 699824.

Below is a risk factor matrix that classifies the above data.

Risk Factor	Overall	Beliefs	Actions
Age	1.270	1.158	1.511
SES	1.001	Overall=1.381<br>US=1.023<br>EU=1.538	
Marital status	1.417	Overall= 2.084**	Overall=0.948
Employment	1.674	Overall=1.674**	Overall= 1.491<br>US=0.818<br>EU=2.695***
Education	2.092***	Overall=2.425***<br>US= 1.498<br>EU=2.929***<br>Other=2.547**	Overall= 1.607**<br>US= 1.288<br>EU= 1.967*<br>Other= 1.872
Immigrant	1.961**	Overall= 1.813**<br>US= 0.402<br>EU= 1.642	Overall= 2.236**
Poor integration	3.145***	Overall=1.555**	Overall= 3.626***
Institutional trust	1.725***	Overall=1.381***	Overall=2.417***
Differential associations	2.367***	Overall=1.647**	Overall=2.794***<br>US= 2.127***<br>EU=3.670***<br>Other=3.176***
Mental health	1.835***	US=1.927**<br>EU=1.798**	
Experienced racism	1.958***	Overall= 1.781***<br>US=1.107<br>EU=2.777***	
Experienced violence	3.131***		

This project has received funding from the European Union's Horizon 2020 research and innovation programme under grant agreement N° 699824.

Social media	2.223***	EU=1.379**	
Criminal history	6.002***	Overall=5.441**<br>US=2.552***<br>EU=5.473***	Overall=6.321***<br>US=2.552***<br>EU/IL= 15.544***
Collective relative deprivation	2.271***	Overall= 1.792***<br>US= 3.924***<br>EU= 1.475***	Overall= 3.609***
Politics		Overall= 2.328***<br>US= 3.257***<br>Other=1.766**	
West Vs. Islam		Overall= 2.238**	
Religious identity	1.611***	Overall=1.319***	Overall (EU)=3.397***
Religiosity	3.603***	Overall= 2.779***<br>US= 2.167**<br>Other= 3.225***	Overall=13.207***
Attendance frequency	2.458***	Overall=2.274***<br>Other=1.809**<br>EU= 3.679***	Overall= 3.087***
Prayer frequency	3.609***		
Religious fundamentalism	4.020***	Overall=4.384***	Overall (EU)= 3.251**

32 T2.1 risk factor matrix

This project has received funding from the European Union's Horizon 2020 research and innovation programme under grant agreement N° 699824.

2.9 Heterogeneity and publication bias

The results the meta-analyses presented here must be interpreted correctly and with a perspective that includes consideration of heterogeneity and publication bias. Rather than describe the heterogeneity and bias of each study, it is more worthwhile to report that overall, heterogeneity was quite high for all variables, ranging between 80%-99%. Heterogeneity was evaluated using the $\chi 2$ test and I2 statistic, with p values lower than. 05 indicating heterogeneity with higher I2 values indicating greater variability than would be expected by chance alone (range: 0–100%). As mentioned above, high heterogeneity is expected among these type of correlational based studies (E.g. Xue et-al, 2015). In other areas researching different types of criminal and violent beliefs and behaviours similar degrees of heterogeneity have been found. For example, in a systematic review and meta-analysis risk factor for gang involvement in low income countries, Higginson et-al (2014) found high heterogeneity for about half the factors examines. However, unlike the Higginson et-al (2014) study, this review was able to carry out moderator analyses and focus on specific sources of heterogeneity in the variables. Moderator analyses helped to identify sources of heterogeneity and thus also determine relative differences in effect sizes. However, even with the use of moderator analyses, the high heterogeneity characteristic of observational studies means that while pooled effects provide important evidence, they must be approached with some caution (Lipsey, 2003).

In a recent review of violence related meta-analyses, Hockenhull et-al (2015) specifically explore the issue of heterogeneity and state that "It could be argued that violence, broadly defined, is an inherently heterogeneous phenomenon and the heterogeneity of the literature merely reflects this" (p.23). While the authors make suggestions for introducing different types of studies in order to increase homogeneity, they also are considering only experimental studies. While such approaches have suggested that it may turn out that meta-analyses are ill-suited for violence related research, meta-analysis experts don't necessarily consider heterogeneity to be a bad thing, especially with respect to correlational risk factor work. This position may even hold that it is in fact on account of the heterogeneity among at risk populations that meta-analysis provides the best possible approach for identifying risk factor effects (Borenstein et-al, 2009).

The probability of publication bias was assessed using Egger's regression test. If publication bias was present, attempts were made to evaluate the effects by using the trim and fill method. Only in

This project has received funding from the European Union's Horizon 2020 research and innovation programme under grant agreement N° 699824.

the case of a few risk factors was this method found to identify any significant changes. Other sources of potential bias stem from the use of the same datasets across multiple studies. For example, three studies used the PIRUS dataset and another three studies used the 608-person sample of Bhui et-al (2014, 2014a, 2016). A total of four studies used Pew survey data, although only two studies overlapped in year and countries examined. Two studies had relatively small sample sizes, with the smallest sample size being 46 (Feddes et-al, 2015) and 79 (Ellis et-al, 2015) and the largest studies having between 6000 and 7659 (Pedersen et-al, 2017).

This project has received funding from the European Union's Horizon 2020 research and innovation programme under grant agreement N° 699824.

2.10 Conclusions

It emerges from this review that the quantitative findings of the meta-analysis are overwhelmingly supported in the direction and the magnitude they demonstrate by the un-coded studies. Nevertheless, this study should only be viewed as a first step towards developing more reliable data upon which evidence based policies can become more informed. The literature still remains underdeveloped and more effect sizes are needed in order to produce more reliable statistics. Specifically, the proportion of the literature that offers any quantitative data is within the estimated bracket of 1-5%. This review and its results should hopefully encourage additional quantitative analysis of radicalization.

It seems that its not a matter of the literature being wrong in any way but rather a matter of disaggregating and synthesizing often divergent and contradictory findings. Risk factors differ in their effect on radicalization and recruitment in many ways. There are also important differences between the US and the EU, just as there are between these countries and non-democratic countries in the Middle East, Asia, Africa and South America. Further disaggregation of risk factors at the stage of initial inquiry and analysis will enable more specific and focussed examinations of the issue. More efforts also need to be made to carry out experiments, especially quasi-experiments in the form of evaluations of interventions.

In terms of individual risk factors, it emerges from this review that at the demographic level there appears to be great temporal stability. In the 1977 article "Profile of a terrorist" Russell and Miller Captain found that the interaction between being a young male from a relatively well to do family, who was starting university and who may possibly drop out or fail to find employment quickly, was the most likely to be radical in belief and action[1]. As early as the 1960's, the university campus was identified as a hotbed of radicalization, and its students the most likely to take part in radical action. In other contexts, this phenomena of student radicalization was apparent much earlier on. Lee (2011) examined the "terrorist profile" of Bengali political "terrorists" from the early 1900's. The largest percentage of those who participated in the radical violence were full-time students—and the

[1] This was the first article in Issue 1 of "Terrorism", which would later become the "Terrorism and violence" journal.

This project has received funding from the European Union's Horizon 2020 research and innovation programme under grant agreement N° 699824.

unemployed—and overall, higher education was a strong predictor of being a Bengali terrorist. Over the years however there has been a growing tendency to believe that higher education ought to be associated with a lower risk of radicalization. While there is some evidence that this may be the case in the non-democratic world, it may not be so for democratic settings such as the US and the EU. In his thesis, Larue (2012) specifically examines the university campus as a possible risk node of radicalization. Enrolment in university was a weak, although significant predictor of radicalization of belief. However, age and educational attainment were much stronger and more significant predictors.

In addition, in another early study, Lyons and Harbinson (1986) compared characteristics between political (terrorist) and non-political murderers in Ireland. The perpetrators of political murders were less likely to be alcoholic, have mental health issues, or a family history of these issues than were their traditional murderer counterparts. In another early study, Weinberg & Eubank (1988) examined characteristics of Italian leftist and neo fascist terrorists. They found that local, meso-level factors such as community size (urbanity) were important predictors of becoming a terrorist. Individual characteristics related to age, marital status, and having a family member in one of the respective groups were also identified as important factors. A more recent study of 1960-70's left-wing radicals had similar findings regarding urbanity and differential associations. They also found that radicals were more likely to come from the working middle-class rather than the lower class (Forland et-al, 2010). In this review, differential associations was found to be a strong risk factor for radicalization of action, especially in the EU context.

The apparent temporal stability of these risk factors is in line with Gill et-al's (2016) findings that only two risk factors (military experience and face-to-face interactions) differentiate between temporal cohorts. According to Post et-al (2006), the demographic who is at risk of radicalization is the same demographic involved in radical actions that would fall under the category of "activist" and which may be protected by law, such as protesting. Even in contexts as far flung as the Balkans, those at risk of radicalization are more likely to have higher education or currently be in higher education (Richardson et-al, 2017). Additionally, foreign fighters are overwhelmingly university educated, as are jihadist leaders (Dawson & Amarasingam, 2017; Gambetta & Hertog, 2016). Currently, one of the fears, after the recent fall of ISIS' administrative control in Syria and Iraq, is that FFs will return to the western countries where they hold citizenship and recruit others.

This project has received funding from the European Union's Horizon 2020 research and innovation programme under grant agreement N° 699824.

Those currently in full time education in university appear to be from a demographic that may be at an even higher risk of radicalization than poor, unemployed and uneducated individuals.

While the rise of religious inspired terrorism has led to increased polarization, fear, and in many cases ad-hoc policies, religiosity in and of itself is not a good predictor of radicalization. The overwhelming majority of even the most religious individuals do not display radicalization of belief and certainly would never engage in violent radical action. Nevertheless, certain interpretations of religious doctrine, or political doctrine for that matter, can and do lead to radicalization of belief and action. Whether its religious fundamentalism or right-wing/left-wing authoritarianism, the fundamentalist belief that violence is necessary to effect desired change is a significant risk factor. It must be understood however that such beliefs are not learned or acquired in a vacuum. Rather, such beliefs may be brought from foreign countries, learnt in schools, religious institutions and places of worship, in small peer networks, at home, and on the internet. The learning and adoption of such ideas and ways of thinking may also be attractive to those with low attachment, or who have low institutional trust or legitimacy. Those suffering from poor integration may attach themselves more to their religious based identities, which can ultimately lead them through a course of learning that ultimately brings them to fundamentalist thinking and subsequently to radicalization.

Additionally, a range of important social factors appear to be the most identifiable variables that differentiate between radicalization of belief and radicalization of action, especially in the context of the EU; Poor integration, institutional trust and collective relative deprivation. On the level of individual characteristics, those with criminal histories and who score high in religious fundamentalism, and who have radical peers, should be considered to be at the greatest risk of radicalization of action where they already have high radicalization of belief.

This project has received funding from the European Union's Horizon 2020 research and innovation programme under grant agreement N° 699824.

2.11 List of included studies

Acevedo, G. A. and A. R. Chaudhary (2015). "Religion, cultural clash, and Muslim American attitudes about politically motivated violence." Journal for the Scientific Study of Religion **54**(2): 242-260.

Altunbas, Y. and J. Thornton (2011). "Are homegrown Islamic terrorists different? Some UK evidence." Southern Economic Journal **78**(2): 262-272

Bakker, E. (2006). "Jihadi terrorists in Europe." Den Haag: Netherlands Institute of International Relations.

Bakker, E. and R. de Bont (2016). "Belgian and Dutch Jihadist Foreign Fighters (2012-2015): Characteristics, Motivations, and Roles in the War in Syria and Iraq." Small Wars and Insurgencies **27**(5): 837-857.

Beller, J. and C. Kroger (2017). "Religiosity, Religious Fundamentalism, and Perceived Threat as Predictors of Muslim Support for Extremist Violence." Psychology of Religion and Spirituality:

Berrebi, C. (2007). "Evidence about the link between education, poverty and terrorism among Palestinians." Peace Economics, Peace Science and Public Policy **13**(1).

Bhatia, K. and H. Ghanem (2017). "How do education and unemployment affect support for violent extremism?" Brookings Global Working Paper Series.

Bhui, K., et al. (2014). "Might Depression, Psychosocial Adversity, and Limited Social Assets Explain Vulnerability to and Resistance against Violent Radicalisation?" PLoS One **9**(9): 1-10.

Bhui, K., et al. (2014). "Is Violent Radicalisation Associated with Poverty, Migration, Poor Self-Reported Health and Common Mental Disorders?" PLoS One **9**(3): 1-10.

This project has received funding from the European Union's Horizon 2020 research and innovation programme under grant agreement N° 699824.

Bhui, K., et al. (2016). "Pathways to sympathies for violent protest and terrorism." The British Journal of Psychiatry: bjp. bp. 116.185173.

Boehnke, K., et al. (1998). "Right-wing extremism among German adolescents: Risk factors and protective factors." Applied Psychology-an International Review-Psychologie Appliquee-Revue Internationale **47**(1): 109-126.

Cherney, A. and K. Murphy (2017). "Support for Terrorism: The Role of Beliefs in Jihad and Institutional Responses to Terrorism." Terrorism and Political Violence: 1-21.

Cherney, A. and J. Povey (2013). "Exploring support for terrorism among Muslims." Perspectives on Terrorism **7**(3).

Coid, J. W., et al. (2016). "Extremism, religion and psychiatric morbidity in a population-based sample of young men." The British Journal Of Psychiatry: The Journal Of Mental Science **209**(6): 491-497.

Corner, E. and P. Gill (2015). "A false dichotomy? Mental illness and lone-actor terrorism." Law and Human Behavior **39**(1): 23.

Delia Deckard, N. and D. Jacobson (2015). "The prosperous hardliner: Affluence, fundamentalism, and radicalization in Western European Muslim communities." Social Compass **62**(3): 412-433

Doosje, B., et al. (2013). "Determinants of Radicalization of Islamic Youth in the Netherlands: Personal Uncertainty, Perceived Injustice, and Perceived Group Threat." Journal of Social Issues **69**(3): 586-604.

Doosje, B., et al. (2012). "“My in-group is superior!”: Susceptibility for radical right-wing attitudes and behaviors in Dutch youth." Negotiation and Conflict Management Research **5**(3): 253-268.

Dornschneider, S. (2016). Whether to kill: The cognitive maps of violent and nonviolent individuals. Baltimore, MD, US, University of Pennsylvania Press.

This project has received funding from the European Union's Horizon 2020 research and innovation programme under grant agreement N° 699824.

Ellis, B. H., et al. (2015). "Trauma and Openness to Legal and Illegal Activism Among Somali Refugees." Terrorism and Political Violence **27**(5): 857-883.

Ellis, B. H., et al. (2016). "Relation of Psychosocial Factors to Diverse Behaviors and Attitudes Among Somali Refugees." American Journal of Orthopsychiatry **86**(4): 393-408.

Feddes, A. R., et al. (2015). "Increasing self-esteem and empathy to prevent violent radicalization: a longitudinal quantitative evaluation of a resilience training focused on adolescents with a dual identity." Journal of Applied Social Psychology **45**(7): 400-411.

Gill, P., et al. (2016). "Indicators of lone actor violent events: The problems of low base rates and long observational periods." Journal of Threat Assessment and Management **3**(3-4): 165.

Gill, P., et al. (2014). "Bombing alone: tracing the motivations and antecedent behaviors of lone-actor terrorists." J Forensic Sci **59**(2): 425-435.

Gill, P. and J. K. Young (2011). "Comparing role-specific terrorist profiles."

Ginges, J., et al. (2009). "Religion and support for suicide attacks." Psychol Sci **20**(2): 224-230.

Gruenewald, J., et al. (2013). "Distinguishing “loner” attacks from other domestic extremist violence." Criminology & Public Policy **12**(1): 65-91.

Gruenewald, J., et al. (2016). "American jihadi terrorism: A comparison of homicides and unsuccessful plots." Terrorism and Political Violence: 1-20.

Horgan, J., et al. (2016). "Across the Universe? A Comparative Analysis of Violent Behavior and Radicalization Across Three Offender Types with Implications for Criminal Justice Training and Education." US Department of Justice, June.

This project has received funding from the European Union's Horizon 2020 research and innovation programme under grant agreement N° 699824.

Horgan, J., et al. (2016). "Actions speak louder than words: A behavioral analysis of 183 individuals convicted for terrorist offenses in the United States from 1995 to 2012." Journal of forensic sciences **61**(5): 1228-1237.

Ianchovichina, E. and Y. Kiendrebeogo (2016). "Who Supports Violent Extremism in Developing Countries? Analysis of Attitudes Based on Value Surveys."

Jasko, K., et al. (2016). "Quest for significance and violent extremism: The case of domestic radicalization." Political Psychology.

Jensen, M., et al. (2016). Empirical assessment of domestic radicalization (EADR), National Consortium for the Study of Terrorism and Responses to Terrorism (START) College Park, MD.

Kerodal, A. G., et al. (2016). "Commitment to extremist ideology: Using factor analysis to move beyond binary measures of extremism." Studies in Conflict & Terrorism **39**(7-8): 687-711.

Klausen, J., et al. (2016). "The Terrorist Age-Crime Curve: An Analysis of American Islamist Terrorist Offenders and Age-Specific Propensity for Participation in Violent and Nonviolent Incidents." Soc Sci Q **97**(1): 19-32.

Krueger, A. B. (2008). "What makes a homegrown terrorist? Human capital and participation in domestic Islamic terrorist groups in the USA." Economics Letters **101**(3): 293-296.

Kruglanski, A. W., et al. (2016). "What a difference two years make: Patterns of radicalization in a Philippine jail." Dynamics of Asymmetric Conflict **9**(1-3): 13-36.

Lankford, A. (2013). "A Comparative Analysis of Suicide Terrorists and Rampage, Workplace, and School Shooters in the United States From 1990 to 2010." Homicide studies **17**(3): 255-274.

LaRue, F. B. (2012). Birthplace or Meeting Place? An Analysis of Muslim Student Radicalization in American Universities, Lehigh University.

This project has received funding from the European Union's Horizon 2020 research and innovation programme under grant agreement N° 699824.

Leuprecht, C., et al. (2011). "Home-Grown Islamist Radicalization in the West: Using Survey Evidence to Model the Structure of Radical Attitudes."

Littler, M. (2017). "Rethinking democracy and terrorism: a quantitative analysis of attitudes to democratic politics and support for terrorism in the UK." Behavioral Sciences of Terrorism and Political Aggression **9**(1): 52-61.

Loza, W. (2010). "The Prevalence of Middle Eastern Extremist Ideologies Among Some Canadian Offenders." J Interpers Violence **25**(5): 919-928.

Lyons, H. A. and H. J. Harbinson (1986). "A comparison of political and non-political murderers in Northern Ireland, 1974–84." Medicine, Science and the Law **26**(3): 193-198.

Lyons, S. L. (2016). The psychological foundations of homegrown radicalization: An immigrant acculturation perspective. US, ProQuest Information & Learning. **76**

Makkawi, I. A. (2000). Collective identity development and related social psychological factors among Palestinian student activists in the Israeli universities. US, ProQuest Information & Learning. **61:** 464.

Mashuri, A., et al. (2016). "You Are the Real Terrorist and We Are Just Your Puppet: Using Individual and Group Factors to Explain Indonesian Muslims' Attributions of Causes of Terrorism." Eur J Psychol **12**(1): 68-98.

McCauley, C. (2012). "Testing theories of radicalization in polls of US Muslims." Analyses of Social Issues and Public Policy **12**(1): 296-311.

Meloy, J. R. and P. Gill (2016). "The lone-actor terrorist and the TRAP-18." Journal of Threat Assessment and Management **3**(1): 37-52.

Merari, A., et al. (2009). "Personality characteristics of “self martyrs”/“suicide bombers” and organizers of suicide attacks." Terrorism and Political Violence **22**(1): 87-101.

This project has received funding from the European Union's Horizon 2020 research and innovation programme under grant agreement N° 699824.

Mieriņa, I. and I. Koroļeva (2015). "Support for far right ideology and anti-migrant attitudes among youth in Europe: A comparative analysis." The Sociological Review **63**(Suppl 2): 183-205.

Muluk, H., et al. (2013). "Jihad as justification: National survey evidence of belief in violent jihad as a mediating factor for sacred violence among Muslims in Indonesia." Asian Journal of Social Psychology **16**(2): 101-111.

Nivette, A., et al. (2017). "Developmental Predictors of Violent Extremist Attitudes: A Test of General Strain Theory." Journal of Research in Crime and Delinquency: 0022427817699035.

Oskooii, K. A. and K. Dana (2017). "Muslims in Great Britain: the impact of mosque attendance on political behaviour and civic engagement." Journal of Ethnic and Migration Studies: 1-27.

Pauwels, L., et al. (2014). "Explaining and understanding the role of exposure to new social media on violent extremism: an integrative quantitative and qualitative approach." Science and Society.

Pauwels, L. and M. De Waele (2014). "Youth involvement in politically motivated violence: Why do social integration, perceived legitimacy, and perceived discrimination matter?" International Journal of Conflict and Violence **8**(1): 134.

Pauwels, L. and N. Schils (2016). "Differential online exposure to extremist content and political violence: Testing the relative strength of social learning and competing perspectives." Terrorism and Political Violence **28**(1): 1-29.

Pauwels, L. J. and R. Svensson (2017). "How Robust Is the Moderating Effect of Extremist Beliefs on the Relationship Between Self-Control and Violent Extremism?" Crime & Delinquency: 0011128716687757.

Pedersen, W., et al. (2017). "At risk for radicalization and jihadism? A population-based study of Norwegian adolescents." Cooperation and Conflict: 0010836717716721.

This project has received funding from the European Union's Horizon 2020 research and innovation programme under grant agreement N° 699824.

Perliger, A., et al. (2016). "The Gap Between Participation and Violence: Why We Need to Disaggregate Terrorist 'Profiles'." International Studies Quarterly **60**(2): 220-229.

Perry, S., et al. (2017). "Who is the lone terrorist? A study of Vehicle-Borne attackers in Israel and the West Bank." Studies in Conflict & Terrorism(just-accepted): 00-00.

Plutzer, E. (1987). "DETERMINANTS OF LEFTIST RADICAL BELIEF IN THE UNITED-STATES - A TEST OF COMPETING THEORIES." Social Forces **65**(4): 1002-1019.

Pyrooz, D. C., et al. (2017). "Cut from the Same Cloth? A Comparative Study of Domestic Extremists and Gang Members in the United States." Justice Quarterly: 1-32.

Russell, C. A. and B. H. Miller (1977). "Profile of a terrorist." Terrorism **1**(1): 17-34.

Sawyer, J. P. and J. Hienz (2016). "What Makes Them Do It? Individual-Level Indicators of Extremist Outcomes." The Handbook of the Criminology of Terrorism: 47.

Schbley, A. (2004). "Religious Terrorism, the Media, and International Islamization Terrorism: Justifying the Unjustifiable." Studies in Conflict & Terrorism **27**(3): 207-233.

Schils, N. and L. Pauwels (2014). "Explaining violent extremism for subgroups by gender and immigrant background, using SAT as a framework." Journal of Strategic Security **7**(3): 27.

Sela-Shayovitz, R. (2007). "Suicide Bombers in Israel: Their Motivations, Characteristics, and Prior Activity in Terrorist Organizations." International Journal of Conflict and Violence **1**(2): 161-168.

Simi, P., et al. (2016). "Recruitment and Radicalization among US Far-Right Terrorists Recruitment and Radicalization among US Far-Right Terrorists.

Simon, B., et al. (2013). "When dual identity becomes a liability: identity and political radicalism among migrants." Psychol Sci **24**(3): 251-257.

This project has received funding from the European Union's Horizon 2020 research and innovation programme under grant agreement N° 699824.

Slootman, M. and J. Tillie (2006). "Processes of radicalisation." Why some Amsterdam Muslims become Radicals. Amsterdam: University of Amsterdam.

Szlachter, D., et al. (2012). "Radicalization of Religious Minority Groups and the Terrorist Threat-Report from Research on Religious Extremism among Islam Believers Living in Poland." Internal Security **4**(2): 79.

Tausch, N., et al. (2009). "Religious and national identity as predictors of attitudes towards the 7/7 bombings among British Muslims: An analysis of UK opinion poll data." Revue Internationale de Psychologie Sociale **22**(3-4): 103-126.

van Bergen, D. D., et al. (2016). "Turkish-Dutch youths' attitude toward violence for defending the in-group: What role does perceived parenting play?" Peace and Conflict: Journal of Peace Psychology **22**(2): 120-133.

van Bergen, D. D., et al. (2015). "Collective identity factors and the attitude toward violence in defense of ethnicity or religion among Muslim youth of Turkish and Moroccan Descent." International Journal of Intercultural Relations **47**: 89-100.

van der Veen, J. (2016). "Predicting susceptibility to radicalization: An empirical exploration of psychological needs and perceptions of deprivation, injustice, and group threat."

Victoroff, J., et al. (2012). "Psychological Factors Associated with Support for Suicide Bombing in the Muslim Diaspora." Political Psychology **33**(6): 791-809.

Zaidise, E., et al. (2007). "Politics of god or politics of man? The role of religion and deprivation in predicting support for political violence in israel." Political Studies **55**(3): 499-521.

Zhirkov, K., et al. (2014). "Perceptions of world politics and support for terrorism among Muslims: Evidence from Muslim countries and Western Europe." Conflict Management and Peace Science **31**(5): 481-501.

This project has received funding from the European Union's Horizon 2020 research and innovation programme under grant agreement N° 699824.

Freilich, J., et al. (2017). "Comparing Extremist Perpetrators of Suicide and Non-Suicide Attacks in the United States." Terrorism and Political Violence: 1-23.

Hafez, M., & Mullins, C. (2015). The Radicalization Puzzle: A Theoretical Synthesis of Empirical Approaches to Homegrown Extremism. *Studies in Conflict and Terrorism, 38*(11), 958-975.

Khalil, J. (2014). Radical beliefs and violent actions are not synonymous: How to place the key disjuncture between attitudes and behaviors at the heart of our research into political violence. *Studies in Conflict & Terrorism, 37*(2), 198-211.

McCauley, C., & Moskalenko, S. (2008). Mechanisms of political radicalization: Pathways toward terrorism. *Terrorism and political violence, 20*(3), 415-433.

McCauley, C., & Moskalenko, S. (2014). Toward a profile of lone wolf terrorists: What moves an individual from radical opinion to radical action. *Terrorism and Political Violence, 26*(1), 69-85.

McCauley, C., & Moskalenko, S. (2017). Understanding political radicalization: The two-pyramids model. *American Psychologist, 72*(3), 205.

Neumann, P. (2013). The Trouble with Radicalization. *International Affairs, 89*(4), 873-893.

This project has received funding from the European Union's Horizon 2020 research and innovation programme under grant agreement N° 699824.

3 T2.6- - Protective factors against extremism and violent radicalization: A systematic review of research

3.1 Abstract

Extremism, radicalization and terrorism are urgent topics in many countries. Numerous research projects are carried out, most of which focus on risk factors and interventions. In contrast, this article contains a systematic review of international research on protective factors. After screening of more than 2000 documents, we found 17 reports containing 21 analyses that provided quantitative data on potential protective effects. Most studies addressed religious extremism/radicalization, far-right, far-left, and mixed forms were less frequent. Thirty different protective factors showed significant effects. Many were assessed in single analyses, but there were various replicated factors such as self-control, adherence to law and police legitimacy, illness, positive parenting behavior and non-violent significant others, good school achievement, non-violent peers, contact to foreigners, and a basic attachment to society. Most findings are similar to what we know from more general research on youth violence. Therefore, it is recommended not to separate the topic of extremism and violent radicalization from other fields of developmental and life course criminology.

3.2 Introduction

Political and other extremism, radicalization towards violence and recruitment for terrorism are currently urgent topics of crime policy in many countries. Accordingly, numerous research projects and prevention programs on these topics have recently been launched. However, the empirical basis in these fields is still rather thin, which is a core problem of systematic evidence-based approaches to prevention and control (Jensen et al., 2016; Sageman, 2014; Silke, 2001). This situation is due to a number of issues that can only be mentioned briefly in this article:

This project has received funding from the European Union's Horizon 2020 research and innovation programme under grant agreement N° 699824.

First, there is no generally shared terminology; ‘extremism’ or ‘radicalization’ often refers to more or less gradual differences in attitudes and behavior (Bartlett & Miller, 2012; Kruglanski, Chen, Dechesne, Fisman, & Orehek, 2009). Second, the field is very heterogeneous and contains groups that are far-right, far-left, religiously motivated, nationalistic/separatist or focus on special issues such as animal protection or anti-abortion (Doosje, Moghaddam, Kruglanski, Wolf, de Mann & Feddes, 2016). Third, although various phases of radicalization have been proposed, the respective processes that may start from ideological interest and proceed over group affiliation to violent action are neither deterministic nor uniform (Borum, 2011; Jensen et al., 2016). Fourth, whereas the influence of group membership plays a core role, there are also cases of ‘self-radicalization’ or ‘lone wolf’ terrorism (e.g. Meloy & Genzman, 2016). Fifth, the theoretical approaches to explanation are heterogeneous and range from social learning and reinforcement over social bonding and control to ambiguity tolerance and mental health issues. Sixth, many research projects are cross-sectional or retrospective case analyses that contain various methodological problems (Scarcella, Page, & Furtado, 2016). Seventh, although there are sound evaluations of programs against intergroup prejudice (e.g. Beelmann & Heinemann, 2014) much less controlled evaluations address projects against extremism and violent radicalization (e.g. Armborst & Kober, 2017).

Within this context research on risk factors for radicalization plays an important role. A number of risk assessment instruments have been developed for use in practice, for example, VERA-2R, ERG-22+, TRAP-18, MLG, IVP, RAT, IAF, and RADAR-ItE (for brief overviews see King, Bender, & Lösel, 2017; Rettenberger, 2016). Some measures that are used by secret services are not published for plausible reasons. The risk assessment instruments are mainly based on structured clinical judgment that is widespread in other areas of violence risk prediction. Depending on the specific focus of radicalization and institutional contexts, the measures vary, but many constructs and items are similar. Due to low base rates of the outcome criteria, partial unwillingness of target persons to cooperate, and a lack of large-scale prospective studies risk assessments cannot yet claim proven validity. Similar to the risk assessment in other domains of violence an accumulation of risk factors (Lösel & Bliesener, 2003) seems to be most indicative (Jensen et al., 2016). However, one must bear in mind that even when 100 percent of known terrorists have a specific pattern of risk factors as (LaFree, Jensen, Atwell-Seate, Pisoiu, Stevenson & Tinsley (2017) showed, the vast majority of ordinary people with such characteristics do not become radicalized or carry out violent acts. This

This project has received funding from the European Union's Horizon 2020 research and innovation programme under grant agreement N° 699824.

problem of a large number of false positives led to concerns about problems of stigmatization (Royal College of Psychiatrists, 2016; Sarma, 2017).

Against this background it is necessary for both theoretical and practical reasons to widen the perspective from risk factors to protective factors and processes. Doosje et al. (2016), for example, refer to ‘shields of resilience’ that interrupt the development from extremist orientation to membership in radical groups and to violent action (or contribute to de-radicalization). There is, however, much less research on protective factors against extremism and radicalization than on risk factors. This situation is similar to the broader literature on violence of young people where research on risk factors is also more frequent than on protective factors (Lösel & Farrington, 2012). This is partially due to conceptual and methodological issues. Protective factors are not simply the other ‘side of the coin’ of a risk factor (which would be tautological), but require more differentiated research methods (Lösel & Bender, 2003, 2017). For example, one has to investigate curvilinear relations between quantitative variables of direct protective/promotive factors and, in particular, study buffering effects in interaction analyses and hierarchical regressions (Loeber & Farrington, 2012; Lösel & Farrington, 2012).

Studies on protective factors have often been carried out within the context of resilience research. Conceptual and methodological issues in this field have been addressed by Luthar, Cicchetti and Becker (2000), Lösel and Bender (2003), Masten (2016) and others. Resilience refers to a “reduced vulnerability to environmental risk experiences, the overcoming of a stress or adversity or a relatively good outcome despite risk experiences” (Rutter, 2012, p. 336). Within this framework the present article contains first results of a systematic review that concentrates on studies of potential protective factors against extremism, radicalization and recruitment to violent acts such as terrorism. The review has been carried in the research consortium ‘Modelling the processes leading to organized crime and terrorist networks’ (PROTON; funded by the European Commission). Originally, we aimed to focus on violent behavioral outcomes only, but our knowledge of the field and a pilot search suggested a scarcity of behavioral studies that led us to include research on violence-oriented extremist attitudes as well. Due to the assumed low number of relevant empirical studies we included systematic research on all kinds of extremism and radicalization. This approach was justified as there are not only differences, but also similarities in the ideologies and the background of the different groups (Ebner, 2017; Jensen et al., 2016).

This project has received funding from the European Union's Horizon 2020 research and innovation programme under grant agreement N° 699824.

3.3 Method

3.3.1 ELIGIBILITY CRITERIA

Topic of the study: We examined research on far-right, far-left, religiously motivated, nationalistic/separatist, and special issue groups (see Doosje et al. 2016). The main focus was on factors that reduce the likelihood of involvement in violent acts, but also outcomes that are closely related to violent behavior like sympathies for radical violence, the willingness to use violence or a mind-set explicitly favoring, supporting or justifying violence. Studies examining broader underlying attitudes or propensities (e.g., right-wing authoritarianism or social dominance) were not included. In accordance with general research on resilience (e.g., Lösel & Bender, 2003; Rutter, 2012) and desistance from crime (Farrall, Hough, Maruna, & Sparks, 2011; Shapland, Bottoms, & Grant, 2012) we decided to concentrate not only on factors against radicalization but also included the reverse, namely de-radicalization and disengagement (e.g., Barelle, 2014; Bjorgo, 2011; Horgan, 2009).

Publication characteristics: For reasons of research-economy we focused on publications in English and German language. There was no restriction regarding time and mode of publication. ‘Unpublished’ work such as dissertations or ‘grey’ literature was also included. All countries of origin were eligible. There was also no restriction with regard to the scientific discipline of authors and research institutions (e.g. sociology, psychology, political science, history, and cultural science).

Design of the primary studies: The main goal of the present systematic review was to integrate empirical studies with quantitative results on protective factors against extremism and radicalization towards violence and potential terrorism. The study design could be both cross-sectional and longitudinal. Beyond correlational designs we included intervention studies targeting extremism if they provided data on quantitative evaluations on a specified factor that could be interpreted as being protective. We also searched for studies with a qualitative design which

This project has received funding from the European Union's Horizon 2020 research and innovation programme under grant agreement N° 699824.

investigated more than single cases and reported systematic findings, however this part of our project will not be reported here.

Search strategy

The results of meta-analyses and systematic reviews dependent on the search terms used in databases. In a pilot test of potential search terms we used a rather general string, e. g., “protect* AND “terroris*”. This led to an unmanageable number of often irrelevant results. We also tested a very narrow search string, e.g., “protective factor* AND terroris*’ This reduced the number of usable results too much. We thus created a few rather complex search strings including the terms *right-wing, left-wing, islamis*, salafi*, radicali*, jihad*, extremis** and *terror** combined WITH *protect*, buffer*, resilien*, risk factor*, disengag*, deradical*, moderat*, reject*, desist**.

Using these strings we searched 15 databases (Cochrane Library, Campbell Reviews, Dissertation Abstracts, MEDLINE, PubMed, EMBASE, ERIC, German National Library, PsycINFO, Psyndex, Science Direct, Scopus, Sociological Abstracts, Sociological Collection, and World Cat). After the elimination of duplicates the final output resulted in 354 titles/publications. The subsequent stepwise screening process (see Lösel, King, & Bender, 2017) reduced the number to a total of only eight relevant papers. In accordance with other experiences in systematic reviews and meta-analyses (e.g. Schmucker & Lösel, 2011) we also screened the references of articles and used contacts of experts in the field. This ‘snowball method’ led to a screening of more than 2,000 titles. Together with the studies from the databases we finally retrieved 28 documents that met our eligibility criteria. Seventeen reports provided quantitative data. Some of these contained more than one different data set or analysis so that we could finally include 21 ‘studies’ in the present systematic review.

3.4 Results

3.4.1 DESCRIPTIVE DATA OF INCLUDED STUDIES

Not surprisingly, the selected publications were very heterogeneous concerning the type of extremism or ideological background, the design, regional context, target group, method of

This project has received funding from the European Union’s Horizon 2020 research and innovation programme under grant agreement N° 699824.

analysis, measures and outcomes. We assigned the types of extremism to four categories of the ideological spectrum, namely ethnic/religious[2], far-right (right-wing), far-left (left-wing) and nationalist/separatist. The types of extremism/radicalization and the respective research designs are shown in Table 1.

Table 1 Design of studies (analyses) and type of extremism and radicalization

	Right-wing	Left-wing	Ethnic/ religious	Nationalist/ separatist	Mixed	Total
Cross-sectional	4	2	7	1	2	16
Longitudinal	1	0	0	0	0	1
Intervention	0	0	4	0	0	4
Qualitative	0	0	5	2	0	7
Total	5	2	16	3	2	28

More than half of the studies (*n* = 16) addressed religious/ethnic extremism and radicalization. Protective factors against right-wing extremism/radicalization ranked second, other orientations were less frequent. Most of the studies had a quantitative cross-sectional design (*n* = 12) with one measurement. Four studies reported effects of an intervention with a pre-post-design, of which only one included a control group. The longitudinal correlational study had a follow-up of one year. Seven studies had a qualitative design, one of which examined the process of radicalization in a diaspora community, while the remaining six studies investigated disengagement and de-radicalization.

Most quantitative studies were carried out in Europe (*n* = 10), three in the Middle East, two in North America and one each in Asia and Africa. The samples mainly covered young people and young adults up to the age of 30 (*n* = 11) which included students at school and in further education (*n* = 6). Six studies examined samples with an age from adolescence to adulthood. Two studies referred to convicted radical offenders. For details of the age ranges and sample sizes see Table 2.

With regard to the different methods of analysing protective factors the studies were heterogeneous. Many studies addressed both risk and protective factors. The most frequently used methods were various regression models (*n* = 9), whereas in the remaining analyses path models (*n*

[2] This combination was chosen because it occurred that some authors referred to “Islamic extremism” while in fact hostility towards the majority society (e.g. “German-hostile” attitudes and behaviors) was assessed.

This project has received funding from the European Union’s Horizon 2020 research and innovation programme under grant agreement N° 699824.

= 6) and group comparisons (*n* =2) were applied. These designs could at least reveal the respective protective factor as a non-confounded variable (e.g. by negative beta weights or path coefficients with regard to extremist/radical outcomes). Only one study carried out moderator analyses on interactions of the protective factors with a present risk (Pauwels & Svensson, 2017).

Regarding the outcome criteria eight studies addressed violent behavior, ten violence-prone attitudes and five a willingness to use violence. Two analyses formed a composite score of violent behavior and attitudes. Most data based on self-reports of the participants (*n* = 13), two studies analysed free reports on scenarios, and two studies used data from a special database (PIRUS) where various information was collected (including file data of convicted offenders).
Results on protective factors

The following results of our review focus on the *quantitative* studies. Details of the qualitative studies will be reported in our more comprehensive project report next year. Most quantitative studies investigated a wide range of variables. For reasons of parsimony and clarity we only present those variables that showed a protective effect. Since a few studies had rather small samples and our review is exploratory and not theory-testing we accepted $p < .10$ as a threshold, but most significant effects were at $p < .05$ or lower. We found 30 different variables with an effect that could be interpreted as being protective. These factors were grouped according to the more general classification of Lösel and Farrington (2012): individual, family, school, peer group and community/society factors. As in similar classifications there is some overlap between the categories. We mainly used the same terms for the variables as in the respective primary studies. More details will be provided in our final project report. A brief overview of our main findings is presented in Table 2.

2. Identified protective factors against different types of extremism and radicalization

Protective factors	Type of extremism	No. of effects	Outcome criteria	Sample *N*	Age range or *M (SD)*	Country	Authors
Individual factors							

This project has received funding from the European Union's Horizon 2020 research and innovation programme under grant agreement N° 699824.

Self-control	rw, lw, e/r	3[a]	Behavior	6020	16-24	Belgium	Pauwels & Svensson (2017)
Empathy	e/r	1	Attitude	46	14-23	Netherlands	Feddes et al. (2015)
Value complexity	e/r	1	Behavior	81	19.48 (2.14)	UK	Liht & Savage (2013)
Anxiety about getting incarcerated	n/s	1	Willingness	600	18-30	Palestine	Cragin et al. (2015)
Acceptance of police legitimacy	rw, rw	2	Behavior	2879	<18=>22	Belgium	Pauwels & De Waele (2014)
Adherence to law	rw, lw, e/r, lw	4[b]	Attitude, behavior	11003	9th graders	Germany	Baier et al. (2016)
Political disinterest	n/s	1	Attitude	600	18-30	Palestine	Cragin et al. (2015)
Low importance of religion	e/r, e/r	2	Attitude	608	18-45	UK	Bhui et al. (2014)
Intensive religious practice	e/r	1	Attitude & behavior	934	39.90 (13.40)	Indonesia	Muluk et al. (2013)
Employment	mixed	1	Behavior	1473	34.18 (13.22)	USA	Jensen et al. (2016)
Perceived personal discrimination	rw	1	Behavior	2879	<18=>22	Belgium	Pauwels & De Waele (2014)
Subjective deprivation (= negative evaluation of own SES)	rw	2	Attitude	4367	14-21	Germany	Fuchs (2003)
Dissatisfaction with quality of life	n/s	1	Attitude	600	18-30	Palestine	Cragin et al. (2015)
Illness, depression	e/r, e/r, e/r	3	Attitude	608	18-45	UK	Bhui et al. (2014)
Threatening life events	e/r	1	Attitude	608	18-45	UK	Bhui et al. (2014)

This project has received funding from the European Union's Horizon 2020 research and innovation programme under grant agreement N° 699824.

Family factors							
Appreciative parenting behavior	lw<br>e/r	2	Behavior<br>Willingness	11003<br>133	9th graders<br>14-18	Germany<br>Netherlands	Baier et al. (2016)<br>Van Bergen et al. (2016)
Ownership of residential property	e/r, e/r	2	Attitude	141	Families	Pakistan	Asal et al. (2008)
Family members not involved in violence	n/s	1	Willingness	600	18-30	Palestine	Cragin et al. (2015)
Significant other not involved in violence	mixed, mixed	2	Behavior	1496	34.24 (13.30)	USA	Jasko et al. (2016)
Incarceration of a family member	n/s	1	Attitude	600	18-30	Palestine	Cragin et al. (2015)
Membership in militant religious groups	e/r	1	Attitude	141	Families	Pakistan	Asal et al. (2008)
School factors							
Higher educational level	e/r	1	Attitude & behavior	934	39.90 (13.40)	Indonesia	Muluk et al. (2013)
Good school achievement	rw, lw;<br>rw<br>rw	4	Attitude;<br>behavior<br>Attitude	11003<br>590	9th graders<br>12-25	Germany<br>Germany	Baier et al. (2016)<br>Boehnke et al. (1998)
Bonding to school	rw, lw	2[b]	Attitude	11003	9th graders	Germany	Baier et al. (2016)
Peer group factors							
Non-violent peers	n/s<br>mixed, mixed	3	Attitude<br>Behavior	141<br>1496	Families<br>34.24 (13.30)	Pakistan<br>USA	Cragin et al. (2015)<br>Jasko et al. (2016)

This project has received funding from the European Union's Horizon 2020 research and innovation programme under grant agreement N° 699824.

More social contacts / social network	e/r	1	Attitude	608	18-45	UK	Bhui et al. (2014)
Contact to foreigners	rw, rw	2	Attitude; attitude & behavior	4367	14-21	Germany	Fuchs (2003)
Community/society factors							
Basic attachment to society	e/r, e/r<br>e/r	3	Attitude & willingness<br>Attitude	398<br>133	14-18<br>14-18	Netherlands<br>Netherlands	Van Bergen et al. (2015)<br>Van Bergen et al. (2016)
Low social capital	e/r	1	Attitude	608	18-45	UK	Bhui et al. (2014)
Migrant of the first generation	e/r	1	Attitude	608	18-45	UK	Bhui et al. (2014)

Various variables were identified as protective factors against more than one kind of extremism or radicalization. At the *individual level*, good self-control reduced violence in groups with far-right, far-left and ethnic/religious extremist orientation, particularly in young people holding highly extremist beliefs (Pauwels & Svensson, 2017). Similarly, adherence to law was a replicated protective factor against extremist attitudes in the ethnic/religious, far-right as well as far-left spectrum and reduced reduced violent left-wing extremist behavior as well (Baier et al., 2016). Acceptance of police legitimacy (Pauwels & De Waele, 2014) and illness/disease (Bhui et al., 2014) also had a protective effect in more than one analysis. It should, however, be kept in mind that these findings based on several comparisons within single projects and were not replications across different studies.

At the *family level* an appreciative/positive parenting behavior (Baier et al., 2016), non-violent significant others (Jasko et al., 2016) and ownership of residential property (Asal et al., 2008) had a protective effect on different kinds of extremism/radicalization.

This project has received funding from the European Union's Horizon 2020 research and innovation programme under grant agreement N° 699824.

There were also replicated and more general protective effects at the *school level*. Both, good school achievement and bonding to school reduced far-right and far-left extremist attitudes and behavior (Baier et al., 2016; Boehnke et al., 1998).

With regard to *peer group* influences contact with non-violent peers had a protective effect against mixed types of extremism and nationalist/separatist orientations in more than one study (Cragin et al., 2015; Jasko et al., 2016). More specific was the finding that contact to foreigners buffered against far-right extremism (Fuchs, 2003).

The latter result could also be subsumed under factors at the *community/society* level, where a basic attachment to society appeared as being protective against ethnic/religious extremism (Van Bergen et al., 2015, 2016). That only very few other protective factors were found in this domain is insofar plausible as all primary studies gathered data from individuals that may indirectly reflect influences at the meso- and macro-level.

Most results in our review only based on one study, one sample, one cultural context, and one type of extremism/radicalization respectively (see Table 2). Some of these findings do make sense, e.g. a protective function of employment, concern about getting incarcerated, empathy with non-Muslims, becoming politically disinterested, value complexity, incarcerated or non-violent family members, higher education, and being a first generation migrant. Other results seemed to be somewhat contradictive, such as intensive religious practice versus low importance of religion or having a wider social network versus low social capital. With regard to such findings it needs to be emphasized that they are rather specific and may not be generalized across countries, topics and samples. Some of these results may be counter-intuitive at first glance and need to be addressed in the following discussion.

3.5 Discussion

Most studies on extremism, radicalization and recruitment into terrorism focus on risk factors. Although these are key topics for research and practice they ignore that the majority of individuals with a 'risky' profile do not start and/or not proceed on a pathway toward violent radicalization. This issue is similar to what we know from research on users of 'hard' illegal drugs: Many often used to consume cannabis or other 'soft' drugs, but only a minority moved on to heroin and other

This project has received funding from the European Union's Horizon 2020 research and innovation programme under grant agreement N° 699824.

‘harder’ drugs. Accordingly, it is highly important to understand the factors and influences that protect and/or buffer against radicalization and violent extremism. Whereas such questions are important in research on resilience, they are yet rarely addressed with regard to extremism and radicalization.

To our knowledge our study is the first *systematic* review with a particular focus on protective factors against extremism and violent radicalization. We aimed to establish a broad evidence-base, but a pilot search suggested that there is very limited quantitative research on this topic. We therefore defined rather broad eligibility criteria and included data from intervention studies as well as qualitative research that went beyond mere case descriptions. For reasons of space the latter data are not presented here. We did not carry out a meta-analysis on the quantitative studies in our systematic review because the topics, variables, samples, contexts and data analyses of the primary studies were extremely heterogeneous. Under these conditions the calculation of effect sizes and means is not meaningful and would increase the risk of artificial or non-replicable findings (Lösel, 2017). We concentrated on factors that had at least one significant protective effect (see Table 2). Our findings showed an array of factors that seemed to have a protective function against extremism and radicalization towards violence. Various individual, family, peer group and school factors were remarkably similar to what has been reported in studies of protective effects against more general violence of young people (Lösel & Bender, 2017; Lösel & Farrington, 2012).

With regard to *individual* protective factors, self-control (low impulsivity) was one of the characteristics that are consistent to research in other fields of crime and violence (Lösel & Bender, 2017). Another protective factor was employment. This is consistent to North American research on radicalization (e.g. Jensen et al., 2016) and also known from criminological research that has shown that having a job helps to desist from a pathway of crime (e.g. Sampson & Laub, 2003). Although our synthesis (like other studies) subsumed good school achievement under school factors, it is also an indirect indicator of above-average intelligence. This personal resource has repeatedly shown a protective effect against crime and violence (Ttofi, Farrington, Piquero, Lösel, DeLisi & Murray, 2016). Our finding of a protective function of anxiety about being caught and incarcerated is also in accordance with the broader literature on violence of young people (Lösel & Farrington, 2012). Beyond the specific content of such consequences an anxious mood goes along with higher physiological arousal and may thus reduce engagement in risky behavior and crime (Raine, 2013).

This project has received funding from the European Union’s Horizon 2020 research and innovation programme under grant agreement N° 699824.

The finding on empathy has less equivalence in other research on violence. This may partially be due to the heterogeneity of this construct (Joliffe & Murray, 2012) and to differences between types of violence. It must therefore be noted that the result of Feddes, Mann, & Doosje (2015) explicitly refers to empathy towards non-Muslims. This protective function can be explained by processes of social information processing (Dodge & Pettit, 2003; Lösel, Bliesener, & Bender, 2007). Within this context perspective taking should counteract black-and-white thinking and hostility biases in violence-prone cognitive schemata. In contrast, empathy may be a risk factor if it is only shown for the own radical Muslim group members (Rahimullah, Larmar, & Abdalla, 2013). The protective impact of illness that has been found in a sample of individuals with Pakistani or Bangladeshi origins in England (Bhui et al., 2014) does not seem to make sense at first sight, but in a study of ‘hard core’ football hooligans this issue has also been relevant for those who desisted from violence (Lösel & Bliesener, 2006).

In contrast to these factors, our findings on subjective experiences of deprivation, discrimination and dissatisfaction with own quality of life seem to deviate from other fields of violence. One could have expected that these factors have a risk instead of a protective effect. Therefore we recommend caution in interpretations of these heterogeneous studies (Bhui et al., 2014; Cragin et al., 2015; Fuchs, 2003). They addressed attitudes towards suicide attacks in the West Bank, far-right attitudes of school students in Germany, and attitudes on religious extremism of young adults in Belgium. We assume rather different mechanisms behind these findings. For example, the far-right attitudes in the German study were related to higher self-esteem and authoritarian attitudes as risk factors that may counteract feelings of discrimination and deprivation.

Some other findings may also be counter-intuitive at first sight. For example, in a British sample of people with Pakistani or Bangladeshi roots, “being a migrant” was connected to more condemnation of violent extremism (Bhui et al., 2104), but this may be plausible when one takes cultural tensions and the generation of migration into account (Mansour, 2016). Another example of context-specific findings in Table 2 relate to religious orientation. Whereas Muluk, Sumaktoyo, and Ruth (2013) found that intensive religious practice was a protective factor against committing extremist violence in Indonesia, less identification with religion was protective against violence-oriented attitudes in Muslim individuals from Pakistani or Bangladeshi families in England (Bhui et al., 2014). These

This project has received funding from the European Union’s Horizon 2020 research and innovation programme under grant agreement N° 699824.

examples suggest that the cultural and religious framing conditions play a role for the specific motivation for radicalization and de-radicalization in different contexts.

On the *family* level parenting behavior seems to have a protective effect. Although the terminology and measures are different, the significant finding on an 'egalitarian' parenting style is similar to the protective effects of positive parenting in other fields of juvenile violence (Lösel & Bender, 2017). Another family factor that showed a protective effect was ownership of residential property. This is similar to a protective effect of the family's socio-economic status in violence research (Lösel & Farrington, 2012). More complex are the findings that non-violent family members (Cragin et al., 2015) as well as family members of militant religious groups (Asal et al., 2008) or in custody (Cragin et al., 2015) may have a protective influence. Such results are not inconsistent when different contexts and mechanisms are taken into account. On the one hand, they may indicate vicarious reinforcement and on the other hand they may involve a more or less conscious deterrent effect when family members faced serious negative consequences.

With regard to *school* factors the findings were clearly consistent to research in other criminological fields (Lösel & Bender, 2017): Bonding to school, good school achievement and a higher educational level were protective factors against extremism and radicalization in several studies. As mentioned above, good school achievement may indicate above-average intelligence as a protective factor (Ttofi et al., 2016).

Peer relations showed also similarities to the more general research on protective factors against violence. Having contact with non-deviant peer groups on the one hand, but also some social isolation seem to reduce the risk of extremism and/or moving on a pathway to violent radicalization. This is in accordance with the literature on protective effects against youth violence (Lösel & Farrington, 2012). Similarly, the somewhat contradicting finding on a protective function of both a wider social network and low social capital may be due to different underlying mechanisms. When more social contacts include non-extremist people and when a lack of social capital means also less involvement in extremist networks the respective results are plausible.

At the *community/society* a basic attachment to or integration into society seems to be a potential source of not moving on a pathway of radicalization. This is in agreement with the theory and

This project has received funding from the European Union's Horizon 2020 research and innovation programme under grant agreement N° 699824.

findings on informal social control and social bonding (Hirschi, 1969). Social bonding may also enhance the above-mentioned protective function of self-control (Hirschi, 2004). Another protective factor seems to be specifically relevant for ethnic-religiously oriented extremism. The study of Bhui et al. (2014) suggests that first generation immigrants are less vulnerable to extremism/radicalization than later generations. This is in accordance with more general findings on a particular risk for youth violence in later generations of migrants (Walburg, 2014). Whereas first generation immigrants often aim to adapt to the society, some groups of their offspring may become deviant due to problems of integration, parenting and lower education (Baier, Pfeiffer, Simonson, & Rabold, 2009).

The above-mentioned results show that many protective factors are similar to what we know from other fields of violence of young people. Therefore, extremism, radicalization, and recruitment into terrorism should not be seen as being too isolated from the more general research on youth violence and developmental psychopathology. School bullying is an example for that. Although this has often been investigated as a school phenomenon only, research showed clear longitudinal relations to violent behavior in other contexts (Bender & Lösel, 2011; Ttofi, Farrington, & Lösel, 2012). Accordingly, prevention programs against youth violence may be promising (but not sufficient) against extremism and violent radicalization.

In addition to the more general findings our review revealed protective factors and processes that seem to be more specific. Our findings suggest that individuals who have extremist attitudes did less often develop radical violence, but decided to desist when they have a basic non-rejecting attitude towards law and society and accept police legitimacy. This relation was mainly found in studies of extremism where it is plausible because these groups emphasize law and order. Their development into radicalization may therefore involve ambivalence between conformism to state authority on the one hand and the feeling of a lack of law and order on the other hand. Such a mechanism is also suggested by the study that found an acknowledgement of value complexity as a protective influence (Liht & Savage, 2013).

Unfortunately, only a few of the above-mentioned quantitative studies investigated the same constructs and variables. This relates to the more general need of more replications in criminological research (Lösel, 2017). However, there was some consistency across studies that can

This project has received funding from the European Union's Horizon 2020 research and innovation programme under grant agreement N° 699824.

be used in practice. This may be complemented by our preliminary analyses of qualitative studies which showed that disappointment with leaders or methods of the respective extremist groups play a protective role for desistance (Lösel, King, & Bender, 2017). Of course, it cannot definitely be said whether these individuals became less radical or just left the extremist group without an attitude change. However, this question relates to the above quantitative findings: Extremists or members of radical groups do not seem not be motivated by political or religious attitudes only; they also seem to be characterized by a lack of protective resources on the individual, family, school and peer group level. Such processes are similar to what we know about other violent subcultures such as street gangs or hooligans (Lösel & Bliesener, 2006).

Our systematic review showed similarities between protective factors against extremism and radicalization with research in other fields of social deviance and violence of young people. This may help to reduce and integrate the many theoretical approaches that have been applied to explain the phenomena and accordingly develop prevention and intervention measures. The above findings suggest a number of dynamic protective factors that can be strengthened in prevention and treatment programs. The respective approaches should be based on theories of social-cognitive learning, social bonding, group dynamics, intergroup prejudice and ambiguity tolerance. More controlled and theory-based evaluation is necessary (Beelmann & Heinemann, 2014). Similar to risk factors most single protective factors have small effects and need to be accumulated according to dose-response-relationships (Lösel & Farrington, 2012). This requires multi-modal approaches to prevention (Lösel & Bender, 2012; Lösel, Stemmler, & Bender, 2013).

Limitations: Although our systematic review revealed findings that seem to be replicable and useful for practice, we need to emphasize its limits: To include a reasonable number of primary studies we had to widen the focus beyond violent behavior and included extremist and radical attitudes and propensities. Most of the studies were cross-sectional and thus do not allow a causal interpretation. The topics and contexts were very heterogeneous. Most frequent were studies on religious/ethnic extremism and radicalization, but the samples and degrees of extremism were heterogeneous and mostly did not address the particularly serious forms of violent radicalization and terrorism. In addition, the definition and operationalization of constructs make generalization difficult. For example, Baier et al. (2016) operationalized “Islamic extremism” as “German-hostile” and surveyed “Muslim” adolescents, but the study may not have captured religious extremism per se. Mixing

This project has received funding from the European Union's Horizon 2020 research and innovation programme under grant agreement N° 699824.

culture and religious “belonging” bears the risk of a bias because many people from Turkish descent, for example, are counted as “Muslims“ although they may not be much attached to the religion after all (Blume, 2017). Therefore, the study may partially not deal with religion-oriented hostility, but perhaps with a diffuse non-religious anger or limited integration into society.

Problems like these are not unique. As the broader literature shows there is not a clear red line between the different motivations and ideological backgrounds of extremism and radicalization. Accordingly, Doosje et al. (2016) assign some terror groups to more than one type of extremism. For example, they subsume ISIS not only to the religious type, but also include it in the nationalist-separatist category because they claim territory. It can also be doubted that the “Islamic Jihad Movement in Palestine” is motivated mainly by religious aspirations and not by demands for territory or revenge for the loss of territory after the establishment of the state of Israel in 1948. These examples suggest that “religious” extremism may be a melting pot for different kinds of motivation and perhaps combines different concepts too casually. Similar problems of clear demarcations exist with regard to other types of extremism and radicalization. For example many right-wing extremists may also belong to thrill-seeking violent subcultures (e.g. Lösel & Bliesener, 2006) and not be as politically motivated at it appears on first glance.

Conclusion: The heterogeneity of the types and levels of extremism/radicalization, samples, research methods, and results suggests caution with regard to generalization and simple recommendations for prevention. However, our review showed a number of plausible and partially replicated findings that were similar to the broader research on protective factors, mechanisms and programs against violence of young people. Therefore, the currently very actual topic of extremism and radicalization should not be too much separated from the more general criminological and developmental research on crime and violence.

References*,†

* quantitative studies included in the systematic review

† qualitative studies not included in the main findings

This project has received funding from the European Union's Horizon 2020 research and innovation programme under grant agreement N° 699824.

*Amjad, N., & Wood, A. M. (2009). Identifying and changing the normative beliefs about aggression which lead young Muslim adults to join extremist anti-Semitic groups in Pakistan. Aggressive behavior, 35(6), 514–519. https://doi.org/10.1002/ab.20325

Armborst, A., & Kober, M. (2017). Effekte von Ansätzen zur Prävention Islamistischer Radikalisierung: Systematische Übersichtsarbeit zu den Methoden und Ergebnissen von Studien zur Evaluation von Präventionsansätzen im Bereich Islamismus. *Berichte des Nationalen Zentrums für Kriminalprävention, Nr. 1*. Bonn: NZK

*Asal, V., Fair, C. C., & Shellman, S. (2008). Consenting to a child's decision to join a Jihad: Insights from a Survey of militant families in Pakistan. Studies in Conflict & Terrorism, 31(11), 973–994. https://doi.org/10.1080/10576100802400201

Backes, U. (2007). Meaning and forms of political extremism in past and present. *Středoevropské politické studie*, *9*(4), 242–262.

*Baier, D., Manzoni, P., & Bergmann, M. C. (2016). Einflussfaktoren des politischen Extremismus im Jugendalter: Rechtsextremismus, Linksextremismus und islamischer Extremismus im Vergleich. *Monatsschrift für Kriminologie und Strafrechtsreform/Journal of Criminology and Penal Reform, 99(3),* 171-198. https://doi.org/10.1515/mkr-2016-0302

Baier, D., Pfeiffer, C., Simonson, J., & Rabold, S. (2009). *Jugendliche in Deutschland als Opfer und Täter von Gewalt.* Hannover: Kriminologisches Forschungsinstitut Niedersachsen.

†Barrelle, K. (2014). Pro-integration: Disengagement from and life after extremism. *Behavioral Sciences of Terrorism and Political Aggression*, *7(2)*, 129–142. https://doi.org/10.1080/19434472.2014.988165

Bartlett, J., & Miller, C. (2012). The edge of violence: Towards telling the difference between violent and non-violent radicalization. *Terrorism and Political Violence*, *24*(1), 1–21. https://doi.org/10.1080/09546553.2011.594923

Beelmann, A., & Heinemann, K. (2014). Preventing prejudice and improving intergroup attitudes. A meta-anaylsis of child and adolescent training programs. *Journal of Applied Developmental Psychology*, *35(1)*, 10-24.

Bender, D., & Lösel, F. (2011). Bullying at school as predictor of delinquency, violence and other antisocial behaviour in adulthood. *Criminal Behaviour and Mental Health*, *22*, 99-106.

*Bhui, K., Everitt, B., Jones, E., & Correa-Velez, I. (2014). Might depression, psychosocial adversity, and limited social assets explain vulnerability to and resistance against violent radicalisation? PloS one, 9(9), e105918. https://doi.org/10.1371/journal.pone.0105918

This project has received funding from the European Union's Horizon 2020 research and innovation programme under grant agreement N° 699824.

Bjørgo, T. (2011). Dreams and disillusionment: Engagement in and disengagement from militant extremist groups. *Crime, Law and Social Change, 55*(4), 277–285. https://doi.org/10.1007/s10611-011-9282-9

Blume, M. (2017). Islam in der Krise: Eine Weltreligion zwischen Radikalisierung und stillem Rückzug. Ostfildern: Patmos Verlag.

*Boehnke, K., Hagan, J., & Merkens, H. (1998). Right-wing extremism among German adolescents: Risk factors and protective factors. Applied Psychology, 47(1), 109–126. https://doi.org/10.1111/j.1464-0597.1998.tb00016.x

Borum, R. (2011). Radicalization into violent extremism II: A review of conceptual models and empirical research. *Journal of Strategic Security, 4(4),* 37-62.

†Chernov Hwang, J. (2017). The disengagement of Indonesian jihadists: Understanding the pathways. *Terrorism and Political Violence, 29(2),* 277–295. https://doi.org/10.1080/09546553.2015.1034855

†Chernov Hwang, J., Panggabean, R., & Fauzi, I. A. (2013). The disengagement of Jihadis in Poso, *Indonesia. Asian Survey, 53(4),* 754–777. https://doi.org/10.1525/as.2013.53.4.754

*Cragin, R. K., Bradley, M. A., Robinson, E., & Steinberg, P. S. (2015). What factors cause youth to reject violent extremism? Results of an exploratory analysis in the West Bank. RAND Corporation
http://www.rand.org/content/dam/rand/pubs/research_reports/RR1100/RR1118/RAND_RR1118.pdf

Dodge, K. A., & Pettit, G. S. (2003). A biopsychosocial model of the development of chronic conduct problems in adolescence. Developmental Psychology, 39, 349-371.

Doosje, B., Moghaddam, F. M., Kruglanski, A. W., Wolf, A. de, Mann, L., & Feddes, A. R. (2016). Terrorism, radicalization and de-radicalization. *Current Opinion in Psychology, 11*, 79–84. https://doi.org/10.1016/j.copsyc.2016.06.008

Ebner, J. (2017). *The rage: The vicious circle of Islamist and far right extremism.* London: Tauris.

Farrington, David P. (1997): Early prediction of violent and non-violent youthful offending. In: *European Journal on Criminal Policy and Research, 5, 2,* pp. 51– 66.

Farrall, S., Hough, M., Maruna, S., & Sparks, R. (Eds.) (2011). *Escape routes: Contemporary perspectives on life after punishment*. Milton Park, UK: Routledge.

*Feddes, A. R., Mann, L., & Doosje, B. (2015). Increasing self-esteem and empathy to prevent violent radicalization: A longitudinal quantitative evaluation of a resilience training focused on

This project has received funding from the European Union's Horizon 2020 research and innovation programme under grant agreement N° 699824.

adolescents with a dual identity. *Journal of Applied Social Psychology, 45(7)*, 400–411. https://doi.org/10.1111/jasp.12307

†Ferguson, N. (2016). Disengaging from terrorism: A Northern Irish experience. *Journal for Deradicalization. 6*, 1–23.

*Fuchs, M. (2003). Rechtsextremismus von Jugendlichen. Zur Erklärungskraft verschiedener theoretischer Konzepte. *Kölner Zeitschrift für Soziologie und Sozialpsychologie, 55(4)*, 654–678. https://doi.org/10.1007/s11577-003-0116-3

Hanson, R.K., & Bussière, M.T. (1998). Predicting relapse: A meta-analysis of sexual offender recidivism studies. Journal of Consulting and Clinical Psychology, 66, 348-362.

Hirschi, T. (1969). *Causes of delinquency*. Berkeley, CA: University of California Press.

Hirschi, T. (2004). Self-control and crime. In R.F. Baumeister & K.D. Vohs (Eds.), *Handbook of self-regulation: Research, theory, and applications* (pp. 537-552). New York: Guilford.

Horgan, J. (2009). Individual disengagement: A psychological analysis. In J. Horgan & T. Bjørgo (Eds.), *Cass series on political violence. Leaving terrorism behind. Individual and collective disengagement* (pp. 17–29). Oxon: Routledge.

*Jasko, K., LaFree, G., & Kruglanski, A. (2016). Quest for significance and violent extremism: The case of domestic radicalization. *Political Psychology, 1(2)*, 195. https://doi.org/10.1111/pops.12376

*Jensen, M., LaFree, G., James, P. A., Atwell-Seate, A., Pisoiu, D., Stevenson, J., & Picarelli, J. (2016). Final Report: Empirical assessment of domestic radicalization (EADR). *Report to the National Institute of Justice, Office of Justice Programs.* Washington, DC: U.S. Department of Justice.

Jolliffe, D., & Murray, J. (2012). Low empathy and offending. In R. Loeber & B. C. Welsh. (Eds.), *The future of criminology* (pp. 62-70). Oxford, UK: Oxford University Press.

King, S., Bender, D., & Lösel, F. (2017). Instrumente zur Beurteilung extremistisch motivierter Straftäter: Eine Synopse. *Paper presented at the 17th Meeting of the Division of Psychology and Law of the German Society of Psychology*. 22.09.2017, Jena, Germany.

Kruglanski, A. W., Chen, X., Dechesne, M., Fishman, S., & Orehek, E. (2009). Fully committed: Suicide bombers motivation and the quest for personal significance. *Political Psychology*, *30*(3), 331–357. https://doi.org/10.1111/j.1467-9221.2009.00698.x

La Free, G., Jensen, M., Atwell-Seate, A., Pisoiu, D., Stevenson, J., & Tinsley, H. (2017). Empirical results on risk of domestic radicalization and terrorism. *Presentation at the first PROTON Consortium Meeting, 15-17 October 2017*. Hebrew University Jerusalem, Israel.

This project has received funding from the European Union's Horizon 2020 research and innovation programme under grant agreement N° 699824.

*Liht, J., & Savage, S. (2013). Preventing Violent Extremism through Value Complexity: Being Muslim Being British. *Journal of Strategic Security*, *6*(4), 44–66. https://doi.org/10.5038/1944-0472.6.4.3

Loeber, R., & Farrington, D. P. (2012). Advancing knowledge about direct protective factors that may reduce youth violence. *American Journal of Preventive Medicine, 43* (2S1), 24-27.

Lösel, F. (2017). Evidence comes by replication, but needs differentiation: The reproducibility issue in science and its relevance for criminology. *Journal of Experimental Criminology,* in press. Online: DOI 10.1007/s11292-017.9297-z .

Lösel, F., & Bender, D. (2003). Protective factors and resilience. In D. P. Farrington & J. Coid (Eds.), *Early prevention of adult antisocial behaviour* (pp. 130–204). Cambridge, UK: Cambridge University Press.

Lösel, F., & Bender, D. (2012). Child social skills training in the prevention of antisocial development and crime. In D.P. Farrington & B. C. Welsh (Eds.), *Handbook of crime prevention* (pp. 102-129). Oxford, UK: Oxford University Press.

Lösel, F., & Bender, D. (2017). Protective factors against crime and violence in adolescence. In P. Sturmey (Ed.), *The Wiley handbook of violence and aggression*. New York: Wiley.

Lösel, F., & Bliesener, T. (2003). *Aggression und Delinquenz unter Jugendlichen: Untersuchungen von kognitiven und sozialen Bedingungen* [Aggression and delinquency in adolescence: Studies on cognitive and social origins]. Neuwied: Luchterhand.

Lösel, F., & Bliesener, T. (2006). Hooliganismus in Deutschland: Verbreitung, Ursachen und Prävention. [Football hooliganism in Germany: Prevalence, origins, and prevention]. *Monatsschrift für Kriminologie und Strafrechtsreform/Journal of Criminology and Penal Reform, 89*, 229-245.

Lösel, F., Bliesener, T., & Bender, D. (2007). Social information processing, experiences of aggression in social contexts, and aggressive behavior in adolescents. *Criminal Justice and Behavior, 34*, 330-347.

Lösel, F., & Farrington, D. P. (2012). Direct protective and buffering protective factors in the development of youth violence. *American Journal of Preventive Medicine*, *43*(2S1), 8-23. https://doi.org/10.1016/j.amepre.2012.04.029

Lösel, F., King, S., & Bender, D. (2017). Protective factors against extremism, radicalisation and recruitment for terrorism. Interim report to the PROTON Research Consortium. Erlangen: Friedrich-Alexander-University Erlangen-Nürnberg.

Lösel, F., Stemmler, M., & Bender, D. (2013). Long-term evaluation of a bimodal universal prevention program: Effects from kindergarten to adolescence. *Journal of Experimental Criminology, 9,* 429-449. DOI 10.1007/s11292-013-9192-1

Luthar, S. S., Cicchetti, D., & Becker, B. (2000). The construct of resilience: a critical evaluation and guidelines for future work. *Child Development*, *71*, 543–562. doi:10.1111/1467-8624.00164

Mansour, A. (2016). Generation Allah: Warum wir im Kampf gegen religiösen Extremismus umdenken müssen (5. Auflage). Frankfurt am Main: S. Fischer.

Masten, A.E. (2016). Resilience in developing systems: the promise of integrated approaches. *European Journal of Developmental Psychology*, *13*, 297-312.

McGilloway, A., Ghosh, P., & Bhui, K. (2015). A systematic review of pathways to and processes associated with radicalization and extremism amongst Muslims in Western societies. *International Review of Psychiatry*, *27*(1), 39–50. https://doi.org/10.3109/09540261.2014.992008

Meloy, J. R., & Genzman, J. (2016). The clinical threat assessment of the lone-actor terrorist. *The Psychiatric Clinics of North America*, *39*(4), 649–662. https://doi.org/10.1016/j.psc.2016.07.004

*Muluk, H., Sumaktoyo, N. G., & Ruth, D. M. (2013). Jihad as justification: National survey evidence of belief in violent Jihad as a mediating factor for sacred violence among Muslims in Indonesia. *Asian Journal of Social Psychology, 16(2),* 101–111. https://doi.org/10.1111/ajsp.12002

Pantucci, R., & Ellis, Clare, Chaplais, Lorien. (2015). *Lone-Actor terrorism. Literature Review* (Countering Lone-Actor Terrorism Series No. 1). Retrieved from Royal United Services Institute for Defence and Security Studies (RUSI) website: https://rusi.org/sites/default/files/201512_clat_literature_review_0.pdf

*Pauwels, L., & De Waele, M. (2014). Youth involvement in politically motivated violence: Why do social integration, perceived legitimacy, and perceived discrimination matter? *International Journal of Conflict and Violence, 8(1)*, 134–153. https://doi.org/10.4119/UNIBI/ijcv.379

*Pauwels, L. J. R., & Svensson, R. (2017). How robust is the moderating effect of extremist beliefs on the relationship between self-control and violent extremism? *Crime and Delinquency, 63(8),* 1000–1016. https://doi.org/10.1177/0011128716687757

Pressman, D.E., & Flockton, J. (2012). Calibrating risk for violent political extremists and terrorists: the VERA 2 structured assessment. *The British Journal of Forensic Practice, 14*, 237-251.

Rahimullah, R.H., Larmar, S., & Abdalla, M. (2013). Understanding violent radicalization amongst Muslims: A review of the literature. *Journal of Psychology and Behavioral Science, 1* (1), 19-35.

This project has received funding from the European Union's Horizon 2020 research and innovation programme under grant agreement N° 699824.

Raine, A. (2013): *The anatomy of violence: The biological roots of crime*. New York: Pantheon Books.

†Reinares, F. (2011). Exit from terrorism: A qualitative empirical study on disengagement and deradicalization among members of ETA. *Terrorism and Political Violence, 23(5),* 780–803. https://doi.org/10.1080/09546553.2011.613307

Rettenberger, M. (2016). Die Einschätzung der Gefährlichkeit bei extremistischer Gewalt und Terrorismus. *Kriminalistik, 8-9*, 532-537.

Royal College of Psychiatrists (2016). *Counter-terrorism and psychiatry*. London: Royal College of Psychiatrists.

Rutter, M. (2012). Resilience as a dynamic concept. *Development and Psychopathology, 24*, 335-344.

Sageman, M. (2014). The stagnation in terrorism research. *Terrorism and Political Violence*, *26*(4), 565–580. https://doi.org/10.1080/09546553.2014.895649

Sampson, R.J., & Laub, J.H. (2003). *Shared beginnings, divergent lives: Delinquent boys to Age 70.* Cambridge, MA: Harvard University Press.

Sarma, K.M. (2017). Risk assessment and the prevention of radicalization from nonviolence into terrorism. *American Psychologist, 72*, 278-288.

*Savage, S. (2014). Preventing violent extremism in Kenya through value complexity: Assessment of Being Kenyan Being Muslim. *Journal of Strategic Security, 7(3),* 1–26. https://doi.org/10.5038/1944-0472.7.3.1

Scarcella, A., Page, R, & Furtado, V. (2017). Terrorism, radicalisation, extremism, authoritarianism and fundamentalism: A systematic review of the quality and psychometric properties of assessments. *PLoSONE 11(12): e0166947.* doi:10.1371/journal.pone.0166947

Schmucker, M., & Lösel, F. (2011). Meta-analysis as a method of systematic reviews. In D. Gadd, S. Karstedt & S.F. Messner (Eds.), *The Sage handbook of criminological research methods* (pp. 425-443). Thousand Oaks, CA: Sage.

Shapland, J., Bottoms, A., & Grant, M. (2012). Perception of the criminal justice system among young adult would-be desisters. In F. Lösel, A.E. Bottoms & D.P. Farrington (Eds.) (2012). *Young adult offenders: Lost in transition?* (pp. 128-145). Milton Park, UK: Routledge.

Silke, A. (2001). The devil you know: Continuing problems with research on terrorism. *Terrorism and Political Violence*, *13*(4), 1–14. https://doi.org/10.1080/09546550109609697

Topalli, V. , Brezina, T. , & Bernhardt, M. (2013). With god on my side: The paradoxical

This project has received funding from the European Union's Horizon 2020 research and innovation programme under grant agreement N° 699824.

relationship between religiosity and criminality among hardcore street offenders. Theoretical Criminology, 17, 49-69.

Ttofi, M.M., Farrington, D.P., & Lösel, F. (2012). School bullying as a predictor of violence in later life: A systematic review and meta-analysis of prospective longitudinal studies. *Aggression and Violent Behavior, 17*, 405-418.

Ttofi, M.M., Farrington, D.P., Piquero, A.R., Lösel, F., DeLisi, M., & Murray, J. (2016). Intelligence as a protective factor against offending: A meta-analytic review of prospective longitudinal studies. *Journal of Criminal Justice, 45*, 4-18.

*van Bergen, D. D., Ersanilli, E. F., Pels, T. V. M., & Ruyter, D. J. de. (2016). Turkish-Dutch youths' attitude toward violence for defending the in-group: What role does perceived parenting play? *Peace and Conflict: Journal of Peace Psychology, 22(2),* 120–133. https://doi.org/10.1037/pac0000173

*van Bergen, D. D., Feddes, A. F., Doosje, B., & Pels, T. V. (2015). Collective identity factors and the attitude toward violence in defense of ethnicity or religion among Muslim youth of Turkish and Moroccan Descent. *International Journal of Intercultural Relations, 47,* 89–100. https://doi.org/10.1016/j.ijintrel.2015.03.026

†van der Heide, L., & Huurman, R. (2016). Suburban bliss or disillusionment: Why do terrorists quit? *Journal for Deradicalization, (8),* 1–24. Retrieved from http://journals.sfu.ca/jd/index.php/jd/article/view/64

Walburg, C. (2014). Migration und Jugenddelinquenz: Mythen und Zusammenhänge. Ein Gutachten im Auftrag des Mediendienst. Retrieved from Institut für Kriminalwissenschaften, Universität Münster website: https://mediendienst-integration.de/fileadmin/Dateien/Gutachten_Kriminalitaet_Migration_Walburg.pdf

Weine, S., & Ahmed, O. (2012). *Building Resilience to Violent Extremism Among Somali - Americans in Minneapolis - Building resilience to violent extremism among Somali-Americans in Minneapolis-St.Paul: Final report to human factors/behavioral sciences division, science and technology directorate, U. S. department of Homeland Security*. Final report to human

This project has received funding from the European Union's Horizon 2020 research and innovation programme under grant agreement N° 699824.

factors/behavioral sciences division, science and technology directorate, U. S. Department of Homeland Security.

†Weine, S., Henderson, S., Shanfield, S., Legha, R., & Post, J. (2013). Building community resilience to counter violent extremism. *Democracy and Security*, *9(4)*, 327–333. https://doi.org/10.1080/17419166.2013.766131

4 Individual risk factor studies (T2.3, T2.5, T2.7)

4.1 T2.3 Recidivism of security offenders: Examining the crime-terror nexus

4.1.1 SUMMARY

T2.3, led by HUJI, sought out to improve existing knowledge on the social factors leading to individuals' involvement in terror activity and terror groups. The objective of T2.3 was to analyse the factors of the careers of terror offenders by analysing their criminal and terrorist careers. Information regarding criminal career was provided only by the Israel Prison Service, although West Bank residents might have committed crimes and incarcerated in the prisons of the Palestinian Authority (PA). Due to the lack of co-operation on the behalf of the PA, it would be impossible to know the true criminal histories of each individual. This lack of identification of criminal records in the West Bank in areas under the authority of the PA invalidates any inferences we would draw in regards to this population.

However, the research team did identify that by singling out the data of "security prisoners" from Jerusalem, it was reasonable to ensure that all possible data and event history would be included therein. This is because residents of Jerusalem are under full Israeli jurisdiction both administratively and in terms of their physical place of residence. This was identified as being the only way in which

This project has received funding from the European Union's Horizon 2020 research and innovation programme under grant agreement N° 699824.

valid conclusions could be drawn on the crime-terror nexus from the data. Therefore, the following analyses were carried out on the Jerusalem data set (N=1557).

4.1.2 Introduction

With the growth of terrorism since 9/11, Western democracies have been faced with the challenge of incapacitating growing numbers of security prisoners. In some countries such prisoners have been kept in special facilities which have distinct capacities and methods of control and interrogation, while other nations have incarcerated security prisoners in ordinary penal system institutions (Hamm, 2009; Trujillio et. al, 2009). The problem of the incarceration of such offenders is not a new one of course, but the impending release into the community of potentially hundreds of radical offenders has as yet unknown consequences. The extent to which security prisoners are found to get back to terrorist actions after being released from prison will increasingly affect western countries in the coming years. This is a particularly salient and important issue in the US and the UK, since many prisoners or detainees are gradually being released after their terms of incarceration or due to other legal requirements.

While there are yet to be a large number of post-released terrorism offenders in Europe, in the coming years the number is expected to increase. Additionally, the potential to recidivate among those who have been released after incarceration for less serious terrorism offenses, such as low level violence or non-violent offences, is an important consideration for the European context. There is an ongoing debate in the US for example about the effects of incarceration for radical offences and how incarceration for low level offences may impact the likelihood of future offending. In this case, it is still unknown as to what types of incarceration factors, such as age and sentence length may be associated with recidivism propensities (Ahmed, 2016). The Israeli case

This project has received funding from the European Union's Horizon 2020 research and innovation programme under grant agreement N° 699824.

thus provides an outside case from a democratic setting which can provide a possible window into the future for the EU context. It is already well documented that criminal histories are symptomatic of European terrorists (Basra & Neumann, 2016; German Office for the Protection of the Constitution, 2014; Bakker, 2009). Additionally, many recent terrorists are known to security services, often due to their involvement in radical activity more generally, emphasizing the importance of the issue.

According to the Almagor (2009) organization that represents Israeli victims of Palestinian terrorism, of the 6,912 convicted Palestinian terrorists released from Israeli jails between 1993 and 1999, 12% were re-arrested for murder or attempted murder, in addition to those arrested for other offences. In Europe as well, there is strong evidence to suggest that terrorists were known to security services and have previously been incarcerated for a range of offences (Nesser, 2014). Former State Department intelligence analyst, Dennis Pluchinsky has identified that although there is sufficient anecdotal evidence that suggests that the tendency for released imprisoned terrorists is to return to terrorist activity, there are no comprehensive statistics terrorist recidivism. He advocates that research on the recidivism rate for terrorist offenders is an imperative (Pluchinsky, 2008).

The current study pushed beyond the state-of-the-art by providing the first analysis of recidivism among terrorism offenders and the nexus between radical and non-radical type offences and future terrorism related offending. This study provides an in depth look at the types of criminal histories and backgrounds that characterize both first time and repeating terrorism offenders.

4.1.3 Recidivism among terrorists and the crime-terror nexus

Terrorist and criminal groups have traditionally been seen as two distinct categories of offenses (Clarke & Newman, 2006; Lafree & Dugan,2004:53-57), with the former seeking to effect some sort of change, while the latter pursues *personal goals (Hoffman, 1998).* Nevertheless, this traditional division between terrorism and crime appears to have been overstated. In reality, not all terrorists commit their acts for a general political cause. Many of them, especially those in the lower ranks in the organization, aim to achieve other goals such as status, group support or economic welfare (Clarke &Newman, 2006). Terrorist groups may also be especially attractive to criminals

This project has received funding from the European Union's Horizon 2020 research and innovation programme under grant agreement N° 699824.

because in many ways they satisfy criminal aims and objectives. Perhaps on account of representing the same demographic of individuals with specific criminogenic tendencies, or for other reasons, it seems that terror and criminal groups seem to recruit from the same pool (Laqueur 1977; McCauley & Segal 1987; Basra & Neumann, 2016).

Some scholars have argued that terrorist groups actively recruit criminal offenders, perhaps because they possess some transferable skill set or behavioural attributes that are considered especially useful. It could also be the case that criminal offenders also happen to be generally inclined towards political violence on account of their overall criminogenic propensities (Horgan & Taylor, 2003; Clarke & Newman, 2006; Basra & Neumann, 2016). This possibility is supported by terrorists' tendency to commit common crimes such as murders and kidnappings (Clarke & Newman, 2006a, 2006b; Hamm & Van de Voorde ,2005).

Some have argued that in many cases, radical action may be an attempt to make amends or repentance for their criminal pasts and selves in a 'born again' styled act. However, there is evidence that terrorists may engage in a variety of crimes ranging from motor vehicle violations (Hamm & Van de Voorde ,2005), robberies and extortion (Horgan & Taylor, 2003; Schmid, 2002), arms smuggling (Amir,1993; Curtis and Karacan, 2002; Gheordunescu, 2000), drug trade (Amir,1993; Berry Curtis, Hudson & Kollars, 2002) and transnational organized crime (Makarenko, 2004; Schmid, 1996). Such involvement in crime might provide means for financing terrorism (Basra & Neumann, 2016). Regardless of the reasons, it is clear now that criminal histories are an increasingly key feature of the profile of radical offenders, especially in Europe (Ljujic, Prooijen, & Weerman, 2017; Basra & Neumann, 2016). Although research on the crime-terror nexus has recently grown, it is still rarely evaluated with empirical methods or with official databases.

There is another aspect to the crime-terror nexus which remains under-appreciated and has received little attention. As McCauley and Moskalenko (2008,2012,2017) explain, there are different types of radical actors. Activists and radicals may engage in illegal behaviours that are inherently connected to radicalism but which fall outside the purview of jurisdiction of terrorism laws. Activists and radicals may be incarcerated for a range of related crimes such as public disturbance, malicious damage, racially or hate motivate assault, or assault of a peace officer. According to the two pyramid model's pyramid of radical action (McCauley and Moskalenko, 2008,2012,2017) some

This project has received funding from the European Union's Horizon 2020 research and innovation programme under grant agreement N° 699824.

activists may escalate to becoming radical as a result of progression and involvement in increasingly more serious offences. Escalating from one type of criminal behaviour to more serious behaviours is a tendency that has been noted with regards to other types of offenders. With respect to terrorism, in 2001 Spanish judge Juan Cotino noted that ETA (Basque) youth "start out throwing rocks, then molotov cocktails, and eventually pick up a pistol or wire a car-bomb". Many previous studies have noted that those involved in radical violence often start out with more legitimate and legal forms of radical activity (Post et-al, 2006; Richardson et-al, 2017; Rodríguez, 2013).

The nature of offences and offence type are generally not examined by the literature in a disaggregated way (Coid et-al, 2016; T2.7). Rather, studies that do examine criminal history as an independent variable tend to examine it as a binary representation (e.g. Jasko et-al, 2016; Bhui et-al, 2014). Since it is now known that criminal history more generally is an important risk factor in the EU context as it is in Israel's, T2.3 attempted to advance the knowledge and understanding by disaggregating criminal history.

This study was aimed to look into three aspects of terrorist recidivism:

1) to describe recidivism of a large group of incarcerated terrorists over a long period for the first time;

2) to test the importance of various risk factors known to affect terrorism and criminal recidivism in the context of released terrorists;

3) To test how criminal and terrorist history of repeat offenders affects their risk to commit future terrorist acts.

To achieve these goals, we analysed longitudinal data of all individuals who were held in prisons of the Israel Prison Service collected over 13 years. Static and dynamic risk factors were tested using their personal information. Information regarding previous incarcerations allowed us to probe the effect of criminal and terrorist history, disentangled from other risk factors.

This project has received funding from the European Union's Horizon 2020 research and innovation programme under grant agreement N° 699824.

4.1.4 METHODOLOGY

4.1.4.1 THE DATA

The data for this study was obtained by agreement from the Israel Prison Service (IPS). The data focussed on prisoners who had been charged with terrorist activity in Israel and who are classified as "security prisoners" by the IPS. Security prisoners are defined as those "committed and obvious attack on national security or other illegal activities related to nationalistic ideology." (IPS, 2014). The distinction and classification between criminals and terrorists is made according to the conclusions of the Israeli Security Agency. Prior to 2004, security prisoners were incarcerated by the Israeli Military Police while ordinary criminal prisoners were incarcerated by the Israel Prison Service (IPS). Since 2004, authority over all security prisoners was transferred to the IPS, and data collection under a central and uniform system commenced. Therefore, the IPS records of security prisoners is limited and unreliable.

Most Israeli security prisoners are residents of either the West Bank or Jerusalem district. Only 150,000 Arab residents of the West Bank live under full Israeli jurisdiction (UNOCHA, 2013), which constitutes 5 percent of the Arab population in the West Bank (according to estimates of the Palestinian Authority, PCBS, 2016). The other 95 percent are under the jurisdiction of the *Palestinian* Security Services (PSS), which are responsible for general policing in their places of residence. Due to this dual system, the criminal records in possession of the Israeli authorities for West Bank security offenders is incomplete and perhaps even unknown. However, the residents of Jerusalem are under full Israeli jurisdiction. Therefore, in this study, to ensure most the comprehensiveness of records in the sample, the data included only prisoners registered by the IPS as living in Jerusalem district.

The data covers the period of January 2004 until February 2017 and includes all Jerusalem residents who were incarcerated during this time. In order to ensure the complete history of all prisoners, prisoners whose first incarcerations were indicated to have occurred prior to the start date (2004) were removed from analysis, leaving the full dataset to include 1557 offenders and 2310 incarcerations.

This project has received funding from the European Union's Horizon 2020 research and innovation programme under grant agreement N° 699824.

The data received from the IPS included individual risk factors that are known to affect criminal recidivism such as age at time of incarceration, age at first incarceration, marital status, place of residence, ethnicity and education. Since only 28 female security prisoners were recorded (1.8% of the data) and they were unlikely to have a criminal history, they were removed from the dataset. Similarly, only 20 Jewish security prisoners were incarcerated during the study period (1.3%). While they were included in the analysis, ethnicity had no effect over the outcomes and was not included as a final parameter.

The data included information regarding each incarceration for each individual. The data included start and release date, length of full sentence and crime type (national security, public disorder, assault, murder and manslaughter, sex, vice, property, fraud, fiscal and licensing). Age and marital status were also coded.

Many security prisoners claim affiliation to one of the organizations linked to the Israeli-Palestinian conflict, partly to gain protection and support from their organization. Although most prisoners claimed affiliation to one of the organizations, 36.8% remained unaffiliated. In this study, only the two main Palestinian organizations, Fatah and Hamas, and their affiliated minor organizations were coded. The number of prisoners affiliated with small organizations was negligible (14 prisoners, 0.9%).

This project has received funding from the European Union's Horizon 2020 research and innovation programme under grant agreement N° 699824.

4.1.4.2 Analysis

Most security prisoners in the data had only one security-related incarceration (n=864, 61.1%). Although they were involved in terrorism, they did not have a continuous involvement in terrorist activity over the data's time period. In order to differentiate the one-time offenders from the continuous and repeat offenders, a logistic regression over the first incarceration of every prisoner was conducted. The dependent variable was "security recidivism" (whether the individual was re-incarcerated as a security prisoner). The independent variables included age upon release, sentence length served, marital status, organizational affiliation and type of violation.

In the second analysis, security prisoners with more than one incarceration, criminal or security, were examined using a proportional hazards model to assess recidivism. Testing the effect of criminal and security history, the number of prior security and criminal incarcerations were the key independent variables. Other risk factors tested in the first model were also included with the addition of age at first incarceration. To correct for the dependence between incarcerations of the same person, a mixed effects correction was applied to the model. The first incarceration was excluded in order to disentangle dependency with the first regression model. 20 outlier individuals with unusually high number of criminal or security incarcerations (7 and 4 prior incarcerations, respectively; a total of 20 individuals) were removed from analysis of repeated offenders due to their effects on model stability.

This project has received funding from the European Union's Horizon 2020 research and innovation programme under grant agreement N° 699824.

4.1.5 Results

4.1.5.1 Recidivism of released security prisoners

In the first analysis it was identified that security offenders had a significantly higher recidivism rate than ordinary criminal offenders. While the security prisoners' five-year recidivism rates were found to be 50%, 67% and 94%, for first, second and third incarceration, respectively (See Figure 1), the Israeli criminal recidivism is only 23.4%, 38.8% and 48.9%, respectively (Walk & Berman, 2016). The Israeli criminal recidivism rate is 42.8%. It should be noted that this rate is similar to Germany and France where the rate is around 50% as well.

Figure 1 The rate of security prisoners re-incarceration for new security offenses by number of prior incarcerations

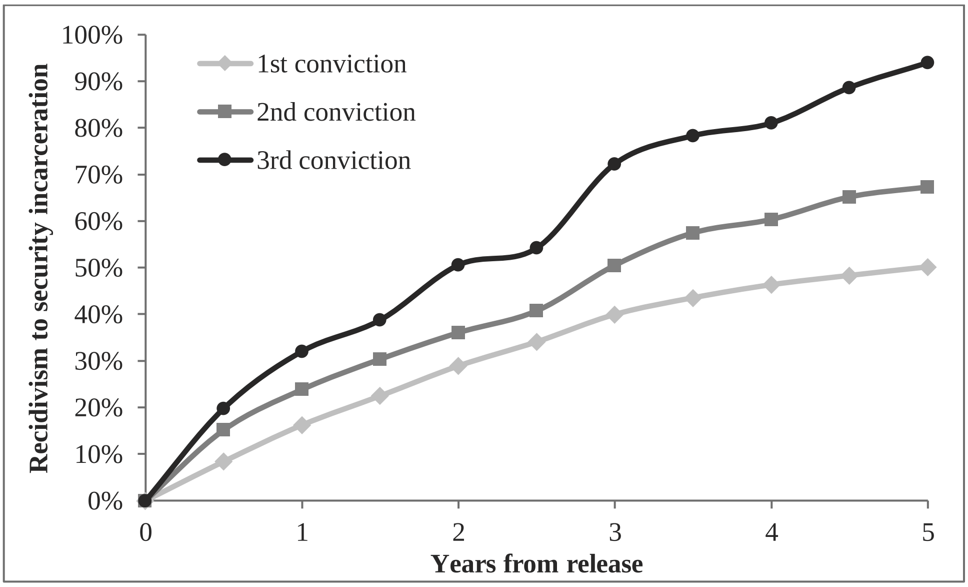

The most common incarceration classification was for "National security" offences, for which all of the dataset had at least one registered offence (46.8%) as described above. The second most common offence was "Public disorder" offences (45.7%). "Murder and manslaughter" offences (18.5%) were greater than the proportion of "General violence" offences (11.9%). Property offences accounted for 20.9% of the offences.[3]

---

[3] Charges leading to incarceration could have included more than one category of violations, leading to the percentages to exceed 100%.

This project has received funding from the European Union's Horizon 2020 research and innovation programme under grant agreement N° 699824.

4.1.5.2 Risk factors of security recidivism

In order to test various risk factors that were found to be indicative of re-offending in previous studies, we used two separate models: first, we tested the recidivism of first-timers (prisoners with no prior incarcerations). We tested the effect of dynamic risk factors focusing on repeating offenders.

One-timers, or offenders that were only incarcerated once for security offenses, represented the majority of cases in the data (61.1%). Logistic regression was used to assess how well different risk factors differentiate between the one-timers and the repeating offenders. As can be seen in Table 1, the risk of repeat offending decreases with age. Similarly, affiliation to organization significantly facilitated offending. Being married had a positive effect. Sentence length has a negative effect on repeat offending. Of all offence types, only public disorder significantly predicted one-time security offending.

Table 1 Logistic regression of recidivism of first-time offenders.

	Log(OR)	OR	SE	z	p	Sig
**Age upon release**	-0.09	0.92	0.02	-3.85	0.0001	***
**Sentence length (weeks)**	-0.02	0.98	0.00	-6.35	0.0001	***
**Terrorist affiliation**	1.75	5.75	0.18	9.52	0.0001	***
**Marital Statatus**[1]						
**Divorced/Widower**	-11.64	0.00	349.57	-0.03	0.9734	
**Married**	1.00	2.72	0.33	3.02	0.0025	**
**Type of violation**						
**Public disorder**	-0.65	0.52	0.19	-3.44	0.0006	***
**Violence**	0.34	1.41	0.31	1.11	0.2672	
**Against person**	0.02	1.02	0.23	0.10	0.9216	
**Property**	0.18	1.20	0.21	0.88	0.3795	
**National security**	-0.29	0.75	0.19	-1.51	0.1319	

A cox regression model of the time to recidivism corrected for repeated measures of repeating offenders was used to examine the risk factors of repeated security offending. Similar to what is observed in the previous analysis, sentence length contributed significantly to reducing recidivism, while terrorist affiliation increased the risk. Conversely, age was only a marginally significant inhibitor of recidivism in repeat offenders, and age at first incarceration was found non-significant. Only national security offenses (e.g., possession and manufacture of explosives, unlawful possession of firearms, affiliation with terrorist organizations, or contact with foreign agents)

This project has received funding from the European Union's Horizon 2020 research and innovation programme under grant agreement N° 699824.

demonstrated a significant suppression of recidivism. Other types of offenses did not significantly affect recidivism to security re-incarceration (Table 2).

Similar to what is observed in the previous analysis, sentence length contributed significantly to reducing recidivism, while terrorist affiliation increased the risk. Conversely, age was only a marginally non-significant inhibitor of recidivism in repeat offenders, and age at first incarceration was found non-significant. Only national security offenses (e.g., possession and manufacture of explosives, unlawful possession of firearms, affiliation with terrorist organizations, or contact with foreign agents) demonstrated a significant suppression of recidivism. Other types of offenses did not significantly affect recidivism to security re-incarceration (Table 2).

Table 2 Mixed effects survival analysis on repeating offenders

	Log(PH)	PH	SE	z	p	Sig
**Number of Security incarcerations**	**0.22**	**1.24**	**0.06**	**3.35**	**0.001**	*******
**Number of Criminal incarcerations**	**-.027**	**0.76**	**0.12**	**-2.32**	**0.020**	*****
Age upon release	-0.07	0.93	0.04	-1.84	0.065	.
**Sentence length (weeks)**	-0.03	0.97	0.00	-7.60	0.000	***
**Age at first incarceration**	0.01	1.01	0.05	0.20	0.840	
**Terrorist affiliation**	1.34	3.82	0.22	6.22	0.000	***
**Marital Statatus[1]**						
**Divorced/Widower[2]**	-19.31	0.00	20311.13	0.00	1.000	
**Married**	0.43	1.53	0.36	1.18	0.240	
**Type of violation**						
**Public disorder**	-0.01	0.99	0.16	-0.08	0.940	
**Violence**	-0.04	0.96	0.33	-0.13	0.900	
**Against person**	0.08	1.08	0.21	0.38	0.700	
**Property**	-0.02	0.98	0.20	-0.08	0.940	
**National security**	-0.38	0.68	0.18	-2.14	0.032	*

[1] – Reference level of marital status: singles.
[2] – Only 14 widowers in the data, none re-incarcerated.

This project has received funding from the European Union's Horizon 2020 research and innovation programme under grant agreement N° 699824.

4.1.5.3 Effect of criminal and terrorist history on security recidivism

The current analysis aimed to explore the nature of the relationship of involvement in terror or criminal activity and its influence on recidivism to terror activity. Predictably, the number of prior security incarcerations increased the risk of security recidivism. Contrarily, the number of prior ordinary criminal incarcerations reduced recidivism.

These effects are consistent across the range of the number of prior incarcerations, with higher recidivism rates correlating with shorter Time-To-Recidivism times (See Figure 2). The effects of prior incarcerations by type are significant even after controlling for other risk factors (See Table 2).

Figure 2 Recidivism and time until re-incarceration by the number of prior criminal incarcerations (upper panels) and security incarcerations (lower panels)

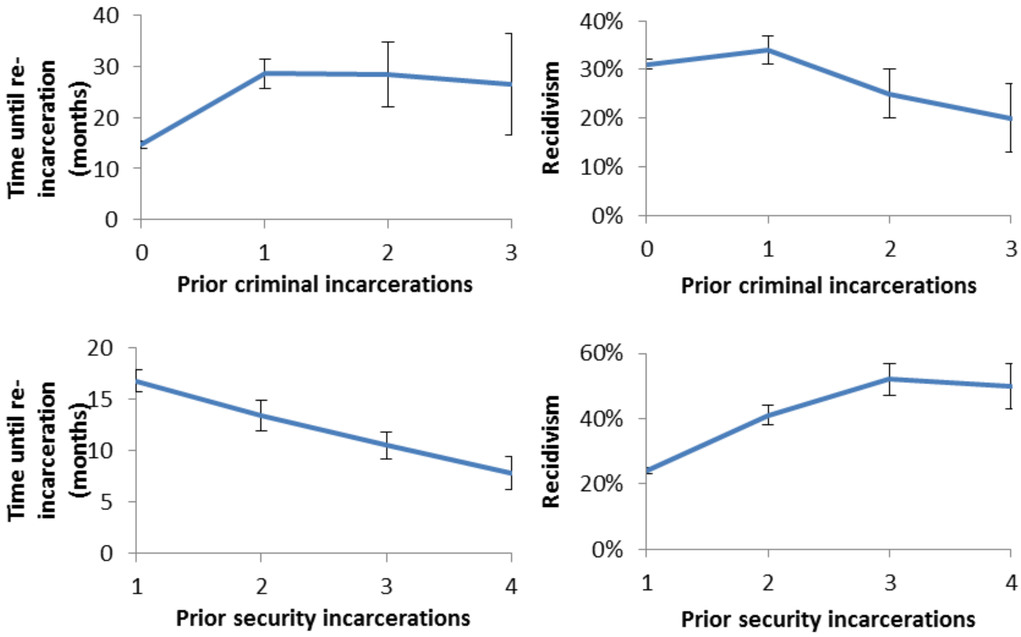

This project has received funding from the European Union's Horizon 2020 research and innovation programme under grant agreement N° 699824.

4.1.6 DISCUSSION

The current study was aimed to answer three questions that were not previously explored using quantitative analysis of large samples. First, although criminal recidivism is thoroughly studied and measured over the world, knowledge of recidivism of terror activists was only limited. Second, risk factors for criminal and terrorist offenders was identified in the literature, but was not previously tested over longitudinal career data. Third, while the involvement of terrorist organizations in crime was described before, the effect of criminal behavior over desistence of terrorists was not tested previously, missing an important aspect of the link between terrorism and criminality. Here, we report for the first time the recidivism rates of a large sample of released prisoners whose crimes were linked to terrorism, and the effects of their criminal and terrorist history while seeking to validate other risk factors as well.

The question of recruitment to terrorism is analogous to models describing social infections of disease. In that context, the question of desistance is a major issue for recruitment, since as ex-terrorists the should be considered as resilient against recurring recruitment ("immune"), and are supposedly also desisted from recruiting others actively or by example ("no longer infecting").

Our analysis suggests that about 60% of the security prisoners are first timers who didn't return to the cycle of terrorism in later stage. We also found that the recidivism rates among security prisoners are about 60%, higher that the average recidivism rates among Israeli criminals in prisons. This is an important finding since to date we couldn’t find a reliable data on terrorist recidivism.

In the next stage we estimated the influence of several risk factor and the way they affected recidivism to terror activity. Age has a negative influence on criminals' careers, this seem to be true also for terrorists' careers. Our analysis show that an increase of terrorist age reduced the odds to recidivate for terrorist offences. Length of sentence seem to have a negative influence on future terror recidivism. Unlike ordinary criminals, terrorists' recidivism is more sensitive to sentence length, which has a reverse effect compared to ordinary criminals for whom it is associated with greater recidivism.
Criminologists are divided as to whether prisons are rehabilitative or criminogenic for regular criminal and terrorist. Our results suggest that the imprisonment reduce future recidivism of terrorists.

This project has received funding from the European Union’s Horizon 2020 research and innovation programme under grant agreement N° 699824.

Several studies on radicalization and involvement in terrorism considered risk factors of marital status. Overall, single males were at a higher risk to be involved in terror activities. However, our analysis found that with regards to recidivism on terrorism offences, married individuals had higher odds. These findings also confirm earlier findings regarding differences between terrorists and ordinary criminals (e.g. Klausen et-al, 2016). Terrorists were more likely to be older than ordinary criminals and therefore also more likely to be married, at least at the time of first offence.

Why would being married be a risk factor for repeated terrorism offences? One interpretation to this finding is that in the Palestinian context, security prisoners are considered by the PA as prisoners of war and their families receive significant financial benefits. This incentivization may be more relevant for married individuals in their persistence in terrorism related activities. However, it could also be that marriage patterns and the relationship between age and marriage among this population is uniquely different (PA, 2008; Jarallah, 2008).

Our analysis showed that for security offenders who had been released from one or more terms of incarceration for security offences, only their security offence history significantly predicted future security offending (terrorism recidivism). We also found that the affiliation of terrorists to an organization increase his likelihood of future incarceration for additional terror offences. One possible explanation for this could be related to high motivations and commitment to the ideology of the organization and/or other peer groups and associations.

Public order offences were negativity associated with future recidivism to terror, indicating that those terrorists who were convicted on public order offences were less likely to be involved in future terror activity. This finding suggests that these prisoners, who had previously practiced non-violent or low level violent offences, were less likely to recidivate. Those who were incarcerated for national security offences however, which could include murder, had a greater risk of recidivating than those with non-security related murder histories.

In the third stage the effect of criminal behavior over desistence of terrorists was analyzed. The findings show that current terrorist offending facilitated later terrorist activity by 1:1.25 per prior security incarceration. But perhaps one of the more interesting findings was that criminal offending

This project has received funding from the European Union's Horizon 2020 research and innovation programme under grant agreement N° 699824.

after involvement in terrorism offenses seems to inhibit future terror offending by 1:1.3 per prior criminal incarceration. This finding suggests that involvement of terrorists in criminal activity may divert them from future terror offending. The question is why? While involvement in both criminal activity and terrorism can be explained by utilitarian motives, still, terror activity can be justified by the actors as a deed for a higher cause while criminal activity is a deed for "lower causes". Hence, involvement of terrorists in criminal activity might decrease their motivation for future involvement in terror activity and may also change their peer group orientation.

4.1.7 Conclusions

The current study examined three main outcomes related to terrorist recidivism. First, we estimated the recidivism rate among the security prisoner which is higher than the criminal offenders. Second, we identified that general risk factors for criminal recidivism, namely age at first incarceration and marital status, differ in important ways for terrorist offenders. While criminal histories may increase overall propensity for radical offending, there may be other factors at play in how criminal histories are relevant for future radical offending. In many cases, it could be that the categories and laws under which offenders are processed play an important role in affecting our understandings. However, of all types of offending, previous low-level radical offending is the strongest risk factor for future radical offending that can be classified as such. In this case, those who have been incarcerated for security/terrorism related offences, are at a heightened risk of re-offending compared to those without such histories and compared with those with only ordinary criminal backgrounds. Finally, our third outcome focused on the relationship between criminal and terrorist activities and showed how criminal involvement reduce the odds for future terror incarceration. Our results are based on the data of Jerusalem; we hope that future access to the PA criminal records will enable validation of these finding among the rest of the security prisoners population

This project has received funding from the European Union's Horizon 2020 research and innovation programme under grant agreement N° 699824.

4.1.8 REFERENCES

Adriano Rodríguez, P. (2013). Violence resistance in Xinjiang (China): tracking militancy, ethnic riots and “knife-wielding” terrorist (1978-2012). *Historia Actual Online, 30*, 135-149.

Ahmed, S. (2016). Is History Repeating Itself: Sentencing Young American Muslims in the War on Terror. *Yale Law Review, 126*, 1520.

Bakker, E. (2006). *Jihadi terrorists in Europe their characteristics and the circumstances in which they joined the jihad: An exploratory study.* The Hague: Netherlands Institute of International Relations.

Bakker, E., & De Bont, R. (2016). Belgian and Dutch jihadist foreign fighters (2012–2015): Characteristics, motivations, and roles in the War in Syria and Iraq. *Small Wars & Insurgencies, 27*(5), 837-857.

Basra, R. a. (2016). Criminal Pasts, Terrorist Futures: European Jihadists and the New Crime-Terror Nexus. *Perspectives on Terrorism, 10*(6).

Clarke, R. V. (2006). *Outsmarting the terrorists.* Greenwood Publishing Group.

de Roy van Zuijdewijn, J., & Bakker, E. (2016). *Lone-Actor Terrorism: Policy Paper 1: Personal Characteristics of Lone-Actor Terrorists.* The Hague : International Centre for Counter-Terrorism (ICCT).

Klausen, J. e.-a. (2016). The Terrorist Age-Crime Curve: An Analysis of American Islamist Terrorist Offenders and Age-Specific Propensity for Participation in Violent and Nonviolent Incident. *Social Science Quarterly, 97*(1), 19-32.

Ljujic, V. v., & Weerman, F. (2017). Beyond the crime-terror nexus: socio-economic status, violent crimes and terrorism. *Journal of Criminological Research, Policy and Practice, 3*(3), 158-172.

Ministry of the Interior. (2014). *2014 ANNUAL REPORT ON THE PROTECTION OF THE CONSTITUTION:FACTS AND TRENDS .* Berlin: Federal Ministry of the Interior, Germany.

Nesser, P. (2014). Toward an increasingly heterogeneous threat: a chronology of jihadist terrorism in Europe 2008–2013. *Studies in Conflict & Terrorism, 37*(5), 440-456.

Post, J. M. (2002). The radical group in context: 1. An integrated framework for the analysis of group risk for terrorism. *Studies in conflict and terrorism, 25*(2), 73-100.

W., F. F. (1990). *Ideology and Repentance: Terrorism in Italy, Origins of Terrorism: Psychologies, Ideologies, Theologies and States of Mind.* Cambridge: Cambridge University Press.

This project has received funding from the European Union's Horizon 2020 research and innovation programme under grant agreement N° 699824.

4.2 T2.4: Counter-terrorism legitimacy and recruitment into terrorism

4.2.1 SUMMARY

Terrorist campaigns involve a triad of "strategic actors – the group, the government, and the audience" (Cronin 2009). A feature of the dynamic relationship between the State and terrorist groups is a struggle to claim legitimacy among local communities. It is sometimes speculated that counter-terrorism strategies create resentment and act as a 'recruiting sergeant', making people radicalised. Understanding how people experience counter-terrorism policing and perceive its legitimacy can therefore be vital not only in encouraging public collaboration, but in preventing radicalisation and recruitment into terrorism.

The key research questions for this study are:
How do Muslims in London understand and perceive terrorism?
What are their experiences of counter-terrorism strategies, and how do they perceive these strategies?
What experiences are believed to put people at risk of radicalisation and recruitment into terrorism?
How can legal authorities build and sustain legitimacy for counter-terrorism practices?
What do they consider to be the role of local communities in preventing terrorism and how can that role be facilitated?

Data for the study come from in-depth interviews of Muslims in London and a national survey of Muslims in England. The fieldwork for the interviews was conducted between March 2017 and September 2017, but the household surveys are still ongoing and expected to be completed in December 2017. This reported in based on preliminary analysis of interview data. A total of 159 interviews were completed, lasting between 30 minutes and 120 minutes.

This project has received funding from the European Union's Horizon 2020 research and innovation programme under grant agreement N° 699824.

4.2.2 SUMMARY OF PRELIMINARY FINDINGS:

Participants expect police officers to keep them safe, to make efforts to understand them and their local communities, and to treat them fairly. However, it also emerged that a history of difficult personal and vicarious experiences has conditioned some participants to develop what might be called *adaptive expectations* of police harassment and disrespect.

Many positive observations were noted about police efforts at preventing terrorism. Participants believed the security services were effective in dealing with terrorism but suggested ambiguity in assessing effectiveness as well as tensions between efforts to prevent terrorist attacks and protecting other societal values.

All participants believed the quality of counter-terrorism practices was the proximate factor that placed people at risk of radicalisation and recruitment into terrorism. Specifically, feelings of being targeted, harassed and treated with disrespect were believed to reinforced a distrust and a sense of alienation, which then made people vulnerable to recruitment. Differences among participants concerned merely the *strength* of their belief about the relevance of counter-terrorism for recruitment.

Participants identified discussed ways to build legitimacy for counter-terrorism practices, and therefore curtailing their unintended consequences. They highlighted engagement with community and religious leaders to develop strategies that identify and support vulnerable people at risk of radicalisation and terrorism, and an effective communication strategy to promote greater 'cultural sensitivity and understanding' of Muslim communities.

4.2.3 BACKGROUND

In an important work security and counter-terrorism, Lowe and Innes observed that:

If violent radicalisation can, at least in part, be driven by disaffection with and insecurity within an individual's immediate social milieu, it would appear that, in some regards, national security is contingent on levels of neighbourhood security. Understanding local perceptions and experiences of crime, security and policing thus becomes a salient counter-terrorism tool (Lowe and Innes 2008: 6).

This project has received funding from the European Union's Horizon 2020 research and innovation programme under grant agreement N° 699824.

The central focus of my work package is, therefore, to understand how Muslims in London (UK) experience and perceive counter-terrorism policing, and how far people believe certain experiences may make place some people at risk of radicalisation and recruitment into terrorism. Terrorist campaigns involve a triad of "strategic actors – the group, the government, and the audience" (Cronin 2009). A feature of the dynamic relationship between the State and terrorist groups is a struggle to claim legitimacy among local communities. It is sometimes speculated that counter-terrorism strategies create resentment and sometimes act as a 'recruiting sergeant', pushing people into terrorism and related offences. Understanding how people experience counter policing and perceive its legitimacy can therefore be vital not only in encouraging public collaboration, but in preventing radicalisation and terrorism.

The key research questions are: How do Muslims in London understand and perceive terrorism? What are their experiences of counter-terrorism strategies, and how do they perceive these strategies? What experiences are believed to put people at risk of radicalisation and recruitment into terrorism? How can legal authorities build and sustain legitimacy for counter-terrorism practices? What do they consider to be the role of local communities in preventing terrorism and how can that role be facilitated?

4.2.4 METHODS

The research design combines qualitative and quantitative methods. Fieldwork for the former took place in London between March 2017 and September 2017. It involved semi-structured interviews with Muslim – and a small number of non-Muslim – Londoners. Three research assistants were recruited and trained for the interviews. The primary objective was to capture experience and perceptions of everyday policing and counter-terrorism. The study was particularly interested understanding those experiences that participants considered important for recruitment and radicalisation. The choice of participants was not random. However, we strove to ensure they were diverse in terms of gender, locality, and nationality. A total of 159 interviews were completed, lasting between half an hour and two hours. the origins and/or nationalities of the participants included: Asian (Pakistani, Bangladeshi, Indian, Iraqi); African (Nigerian, British Sierra Leonean,

This project has received funding from the European Union's Horizon 2020 research and innovation programme under grant agreement N° 699824.

Somali, and Egyptian); Afro-Caribbean (Trinidad and Jamaican); Middle-Eastern (Turkish and Lebanese); South-East Asian (Malaysian); and European (Irish and Albanian).

The quantitative component involves a national household surveys of Muslims. This is ongoing and expected to be completed by December 2017.

4.2.5 PRELIMINARY FINDINGS

Data transcription is 90% completed and coding is ongoing. The preliminary findings reported in August 2017 remain unchanged. I focus here on four key themes: normative expectations; quality and nature of counter-terrorism; role of local communities in counter-terrorism; counter-terrorism as a push factor; and building and sustaining legitimacy for counter-terrorism strategy.

Normative Expectations

Normative expectations matter because they point to mutual (moral) obligations of social actors. For security agencies, building and sustaining legitimacy require accurate reading and effective response to public expectations within the parameters of the rule of law (Beetham 1991; Williams 2005; Bottoms and Tankebe 2017). It follows that conflicting and unfulfilled expectations threaten the formation of police-community relations to promote social order. Participants expected the police to protect them and to make them feel safe in their local communities, to try to understand them, and to treat them fairly:

To do their job and to provide protection and be that individual that we can always turn to. Someone that—police officers that we can have trust in and know they won't discriminate against us. […] I should be feeling more safe and secure around them, not the other way around. I almost feel as if, like, I'm guilty of some crime that I haven't committed and they will pin something on me (Interview F5).

[People] expect to be safe, to be treated fair. Not be pinpointed as a troubled community. They want fairness, they want transparency (Interview MG 20)

This project has received funding from the European Union's Horizon 2020 research and innovation programme under grant agreement N° 699824.

However, a history of negative vicarious and personal experiences appears to have conditioned some participants to expect over-enforcement. This is linked not only to religion but also to socio-economic conditions (cf. Natapoff 2006). The following illustrates such adaptive expectations:

Not very much. I expect [people] – I think they expect to get hassled, and they get it… like I know that the police have, I've seen the police go in houses quite near to me quite a few times, and I've not usually really known what it's about. But there are cameras all over our community as well, like we're an estate right in the middle of an area that's getting increasingly gentrified, and so I think that we get quite, maybe we get more focus. So I think they expect crap from the police, and they get it, frankly. (Interview F34)

Nature and Quality of Counter-Terrorism

Participants believed the security services were effective but suggested ambiguity in assessing effectiveness as well as tensions between efforts to prevent terrorist attacks and protecting other societal values:

Well, I guess relatively speaking the intelligence services in this country are actually quite on the ball and to be honest it's unfortunately a result of the fact that there's a very powerful surveillance system, so in a sense that they're efficient in that way but has its own costs. But I think that the question of what police do well is difficult to answer because there isn't a coherent response or a coherent idea of what should be done. So there isn't a kind of, a standard by which to measure them by. So, in terms of pure effectiveness, if you like, I think that the intelligence services in the UK do a good, if you like, job, according to their own measurements (Interview F11).

I am sure they are doing a good job in preventing terrorism and protecting the public through…you know…the monitoring and surveillance on certain individuals which might be potential terrorists. They need to do this to protect the public and we do understand, but this is different from what we were talking about…arresting people or shooting people. So it's quite different. But I think they are so far…if they have the resources they can do a good job (Interview MG19).

This project has received funding from the European Union's Horizon 2020 research and innovation programme under grant agreement N° 699824.

However, they were critical of certain elements of counter-terrorism practices that they believed impeded police efforts. They believed those elements constituted shortcomings which should be avoided completely if counter-terrorism policies are to be effective. These include dangers of false positives and false negatives arising from seeing ‘non-violent extremism’ as a risk factor in violent extremism and terrorism:

I think the idea of non-violent extremism has become a very dangerous idea and very, very detrimental and, if you like, it’s actually getting in the way of the work that the police could and should be doing. And the reason why I say that is because a lot of the time, individuals who do end up undertaking these activities don’t actually express the kinds of ideas that in policy circles are called, or described as non-violent extremism; these ideas essentially being strong political and social views (Interview F11)

A component of UK counter-terrorism is *Prevent*, which seeks to “respond to the ideology of extremism and terrorism and […] to prevent people from being drawn into terrorism and ensure that they are given appropriate advice and support” (Home Office 2015: 15). Some respondents raised concerns about unevenness in the enforcement of this strategy and questioned the legitimacy of local organisations with whom the government seek to implement Prevent:

In my opinion, PREVENT needs to be rebranded. It needs to challenge all forms of extremism, whether it’s right-wing and needs to be something…not only that but the police need to engage with a lot more with grass roots organisations. They’re [currently] engaging with a lot of bourgeoisie organisations who are not on the ground. So a lot of them don’t have a clue. So that’s one thing they can change (Interview MG20).

Local Communities and Counter-Terrorism Strategy

Our preliminary data show multiple ways that participants believe local communities may contribute to preventing terrorism. These range from acting as critical sources of information for legal authorities and active intervention to prevent young people from becoming radicalised by neutralising the ideological roots of terrorism.

This project has received funding from the European Union’s Horizon 2020 research and innovation programme under grant agreement N° 699824.

I guess the biggest role is to keep an eye out for like aggressive radicalization of their young people. So, that's the best role. If you have a community institution like a mosque or a hub or a center or whatever and you notice the signs of radicalization like yeah, you need to nip that in the bud like immediately, right? You need to deal with it. That's the role that they can play. If someone is given a platform to speak and it seems like they are abusing that platform they should stop that (Interview F2).

The leaders in this community - the imams - they need to structure a programme for Muslims where the can talk about this issue on a weekly basis, if not, every other week, okay? To talk about what Islam says about jihad, okay? What Islam says about living in the West? What Islam says about how you should treat non-Muslims? All of these things you need to educate them, because they have been brainwashed, so what you need to do is de-radicalise these things, de-radicalise these ideologies, this mentality (Interview M34).

4.2.6 Counter-Terrorism as Radicalisation and Recruitment Tool?

A key question for this work package was to explore how counter-terrorism can become counter-productive, pushing people into terrorism rather than preventing terrorism. Previous quantitative studies show the quality of counter-terrorism policing predict willingness to collaborate with the authorities (Tyler et al 2010). Every participant responding to this question indicated the quality of counter-terrorism had consequences for radicalisation and terrorism. The difference was merely in the strength of their belief:

Yeah, I think if - like I said, if one police officer mistreats someone, or abuses his power specifically to a Muslim, this will make Muslims angry. So, for me, radicalisation is more of anger than religion […] Muslims, for example, have seen a lot of their brothers and sisters who died in Iraq, and these people are innocent people, innocent civilians who died […] A lot of Muslims will use this and retaliate against non-Muslims in anger ... but that does not mean that retaliation is the right thing to do in Islam, in that sense (Interview M34)

Absolutely, without a doubt. […] Many people have been pushed out of society because of counterterrorism means and methods, and strategies over the years. Whether it's control orders, or

This project has received funding from the European Union's Horizon 2020 research and innovation programme under grant agreement N° 699824.

security service harassment, or Prevent, or whether it's Schedule 7, people feel that they're being pushed out of society. And some people who have had it worse, they have potentially been manufactured or engineered to be kicked out of the country. And maybe they've gone off to a particular conflict zone, because they were doing some work there, and their passport was revoked, and, as a result, they had nowhere to go in the conflict zone, like Turkey, for example. (Interview F41)

Building and Sustaining Legitimacy Counter-terrorism Strategy

What should counter-terrorism policy look like? It is a crucial question for governments and security services because it concerns how to build and sustain legitimacy for counter-terrorism strategies, with legitimacy defined as power that is lawful, fair and effective (Bottoms and Tankebe 2017; Tankebe 2013). We invited research participants to assume reflective on priority issues in formulating counter-terrorism policy. The data show a diversity of views.

I suggest that a lot more communication with the affected communities are needed to find out what the current issues are. I mean, I'm sure a lot of them police are already aware of, and how the police can further try to build trust with the affected, with the communities that are most affected […] *You know, even if you don't want to go the route of, going to the most obvious, kind of, community leaders, like going to the mosques and so forth. I mean, in some communities, all you'd have to do is perhaps just walk the beat, and just speak to people, speak to the people who run the local shops, who, I don't know, go speak to parents at a local school, I mean, it's not, I don't think it's hard to speak to local people and to try to work out what the issues are* (Interview F10).

Some responses had connotations of what will constitute an effective communication strategy to promote greater 'cultural sensitivity and understanding' of Muslim communities:

A lot more training about cultural sensitivity and understanding the communities in which they operate, and how to communicate to people without being patronising and without being overbearing either (interview F34)

Participants expressed strong objections to various elements of Prevent strategy, and discussed scope for reforms. More positively, they highlighted engagement with community and religious

This project has received funding from the European Union's Horizon 2020 research and innovation programme under grant agreement N° 699824.

leaders to develop strategies that identify and support vulnerable people at risk of radicalisation and terrorism:

Get rid of Prevent. It's a joke. Excuse me. Excuse me, engage community leaders and mosque leaders to think of things [...] Initiatives and areas to better equip them within the local community to deal with young adults, to deal with vulnerable adults, to deal with people that might be slighted towards that way. So, for example, they might have those kind of tendencies a lot of the times these people and these recruits that get involved in terrorism they have mental health issues and the system has failed them [...] and this is their only outlet, unfortunately. I think more engagement in our communities via mosques [...] The thing is there is a lot of distrust with the Muslim and Arab community, that's the problem. So, as the police come and, you know, there's a lot of distrust and but at the same times the Muslims haven't done themselves any favours, especially being secluded and not involved in the community. So, yeah, I think if there was to be more openness, more trust, more honesty (Interview F7).

4.2.7 PLANS FOR QUANTITATIVE DATA

The updated final report will have two objectives: first, to complete and report fully the findings from the qualitative data. The results reported above already point to experiences and perceptions that participants believe to be relevant to recruitment and radicalisation. The second objective will be to incorporate results of quantitative analysis on the experiences that predicted radicalisation and recruitment into terrorism. The fieldwork will be completed this month and results reported not later than January 7, 2018.

This project has received funding from the European Union's Horizon 2020 research and innovation programme under grant agreement N° 699824.

4.2.8 REFERENCES

Beetham, D. (1991). *The legitimation of power*. Palgrave Macmillan.

Bottoms, A., & Tankebe, J. (2017). Police legitimacy and the authority of the state. In M. Ulväng, A. du Bois- Pedain, & P. Asp (Eds.), Criminal law and the authority of the state. Oxford: Hart Publishing

Cronin, A. K. (2009). *How terrorism ends: Understanding the decline and demise of terrorist campaigns*. Princeton University Press.

Home Office. (2015). *CONTEST: The United Kingdom's strategy for countering terrorism* (Cm 9310): Annual Report. London: The Stationery Office.

Natapoff, A. (2006). Underenforcement. *Fordham L. Rev.*, *75*, 1715.

Tankebe, J. (2013). Viewing things differently: The dimensions of public perceptions of police legitimacy. *Criminology*, *51*(1), 103-135.

Tyler, T. R., Schulhofer, S., & Huq, A. Z. (2010). Legitimacy and deterrence effects in counterterrorism policing: A study of Muslim Americans. *Law & Society Review*, *44*(2), 365-402

Williams, B. A. O. (2005). *In the beginning was the deed: Realism and moralism in political argument*. Princeton University Press.

This project has received funding from the European Union's Horizon 2020 research and innovation programme under grant agreement N° 699824.

4.3 T2.5 CORRELATES OF VIOLENT POLITICAL EXTREMISM IN THE UNITED STATES

4.3.1 SUMMARY

Drawing on a database of 1,473 United States-based violent and non-violent extremists, this report provides a preliminary test of the relationship between 12 individual-level attributes and violent extremism. The results show that individuals who are married and have achieved stable employment and advanced educations are significantly less likely to engage in violent extremism. Conversely, we find that individuals who are embedded in social networks of radical peers or family members and have military experience, criminal histories, and suspected mental illness are more likely to plan, prepare for, or engage in acts of political violence. Being young and male is also positively correlated with violent extremism. Future research will utilize multivariate regression analysis in order to better assess the extent to which these attributes can improve our understanding of violent extremism. Moreover, future efforts will include a number of a robustness tests to minimize concerns arising from missing data.

This project has received funding from the European Union's Horizon 2020 research and innovation programme under grant agreement N° 699824.

4.3.2 Research Objective

Research on violent extremism represents one of the major growth areas in social science scholarship over the past two decades. During this period, important research has been conducted that provides a clearer picture of the mechanisms by which individuals and groups come to adopt extreme views (McCauley and Moskalenko 2011), and valuable insights have been generated on the processes that drive extremist organizations to abandon non-violent protest in favor of violent attacks (Bloom 2004; McCauley and Moskalenko 2011; Kydd and Walter 2006). However, scholarship that has sought to identify the factors that are most commonly correlated with violent action has been inconsistent and inconclusive (Neumann and Kleinmann 2013; Gill 2015). The inability to generate cumulative knowledge on the factors that are associated with violent radicalization has been the product of three principal shortcomings in the ways researchers have conceived of radicalization and the designs that they have used to study it.

First, researchers that study radicalization often conflate attitudes and behaviors despite decades of social psychological research showing only weak connections between extreme beliefs and extreme actions (Jones and Harris 1967; Sabini 1995). Survey and experimental research has consistently found strong support for radical ideologies across the political spectrum—and even for the use of violence in support of them—but very few people actually engage in such behavior (McCauley and Moskalenko 2011; Lemieux and Asal 2010). Likewise, there are examples of individuals who have committed serious acts of political violence with relatively weak ideological justifications; most famously Abu Nidal and Carlos the Jackal (Borowitz 2005).

Second, despite more than a decade of intense interest in the issue of radicalization, there is only weak empirical grounding for the current understanding of the characteristics of those who radicalize to the point of violence (Githens-Mazer and Lambert 2010; Borum 2011; Della Porta and LaFree 2012). The main concern here is that most empirical studies of radicalization concentrate only on those who have actually used violence for an extremist cause but do not consider those who may share similar ideological convictions but have not used violence in their pursuit (Borum 2011).

Finally, while excellent work has been done to trace and analyze the radicalization pathways of individuals and small groups, variously highlighting the role of psychological processes (Horgan

This project has received funding from the European Union's Horizon 2020 research and innovation programme under grant agreement N° 699824.

2008; Horgan 2009; Kruglanski et al. 2009), small group dynamics (Sageman 2004), and social movement catalysts (Wiktorowicz 2004; Wiktorowicz 2005), most of these studies rely on limited case evidence to support their claims. There have been few attempts to utilize systematically collected and coded data to generate robust inferences about causes and effects. The large-scale empirical projects that have been conducted have tended to focus exclusively on providing a cross-sectional analysis of individual attributes that at least theoretically can be compared to known population averages. While these studies provide some insight into the factors that increase an individual's susceptibility to radicalization, they do not explain which individuals are more or less likely to engage in violence, nor do they assess when interventions might have been effective.

In short, there have been few quantitative empirical analyses of the characteristics of those who engage in politically-motivated violence in the United States. This report will seek to address this lacuna by assessing the relationship between a number of individual-level characteristics and the risk of violent extremism. In particular, drawing on influential criminological perspectives and extant research on political extremism, we identify 12 individual-level attributes and explore the extent to which they are correlated with violence among United States extremists. These conditions are analysed for a broad spectrum of extremists adhering to far right, far left, Islamist, and single-issue ideologies.

This project has received funding from the European Union's Horizon 2020 research and innovation programme under grant agreement N° 699824.

4.3.3 Individual-level Correlates

The individual-level correlates that are reviewed in this report were drawn from a set of influential perspectives in criminological and political violence research. These include social control, social learning, outbidding, and crime-related background studies.

Social control

Social control perspectives generally hold that deviation from conforming behaviors is more likely to occur when bonds to family, friends, and conventional society are weak (Hirschi 1969; Gottfredson and Hirschi 1990; Laub and Sampson 1993). Overwhelmingly, extant empirical research on those who have committed crime has been aimed at testing whether key life events, such as marriage, employment, or military service, have the potential to alter criminal trajectories, with findings largely supportive of the substantial impact of these positive "turning points" (Laub and Sampson 1993).

From social control perspectives, we assess the relationship of marriage, education, employment, and military service to violent extremism.

Social learning

In contrast to the social control perspective, social learning theory emphasizes that small-group interactions and communication are the primary drivers of deviant and criminal behavior. This perspective emphasizes the impact of social influences, particularly family members and peers (Warr and Stafford 1991). Within this context there is an evolving learning process that involves the transmission of defining behaviors as right or wrong through imitation, modeling, conditioning, and reinforcement (Akers 2009). Put simply, the social networks in which individuals are embedded—family, mentors, and especially peers—will influence them in important ways. This can occur through mechanisms, such as fear or ridicule and loyalty, which produce compliance and status enhancement (e.g., acceptance, reward systems) in adolescent peer networks (Warr 2002). People will be influenced according to the frequency, intensity, duration, and priority of their relationships with others, who in turn help create and mold definitions of behaviors (Akers 2009).

The social learning/peer effects perspective has some similarities to group dynamics models of violent extremism, which suggests that the in-group/out-group biases that form in small cliques can often

This project has received funding from the European Union's Horizon 2020 research and innovation programme under grant agreement N° 699824.

lead to extreme forms of violent expression and groupthink (Bion 1961; Allison 1971; Janis 1972; McCauley 1989; Post 1998). The intense bonds experienced within cliques, and the weak bonds tying individual members to those outside cliques, eventually change the calculus of conformity and remove a brake on the individuals' participation in violent extremism.

From social learning, we assess the relationship of radical family members and radical peers to violent extremism.

Outbidding

Researchers that study how non-violent political organizations transition into violent extremist groups often stress the important role that rivals play in the escalation of extremist behaviors (Bloom 2004; Bloom 2007; Kydd and Walter 2006; Findley and Young 2012). Competition with rival groups often compels individuals to abandon non-violent forms of political expression in favor of violent acts, which are increasingly viewed by the group as a more effective way to garner attention, obtain resources, and establish leadership within a community. The process of outbidding has been to shown to play an important role in the adoption of extreme forms of political violence, including suicide terrorism (Bloom 2004; 2007) and the deliberate targeting of children (Biberman and Zahid 2016).

Similarly, the pull towards increasingly extreme behaviors is common in organizations that experience significant in-fighting over organizational leadership, vision, and routines (Cronin 2011). Such competition within organizations often leads to dangerous splintering, where individuals who were once allies find themselves in opposing factions that are vying for support from a common constituency. Opposing factions are often pulled toward increasingly extreme forms of political behavior in order to outdo each other and establish a hierarchy within the political movement. Following the logic of outbidding theory, extremists will be more likely to engage in violence when they are members of groups or cliques that are engaged in competition with rival organizations or are members of groups that are suffering from internal splintering.

From outbidding theory, we assess the relationship of rival groups to violent extremism.

This project has received funding from the European Union's Horizon 2020 research and innovation programme under grant agreement N° 699824.

Crime-related background studies

Finally, we consider several background characteristics that are staples of criminological predictions about criminal etiology.

Prior criminal record. Having a prior criminal record has long been regarded by criminologists as one of the single best predictors of future criminal behavior (Blumstein, Cohen, and Farrington, 1988; Loeber and Le Blanc, 1990). Indeed, most actuarial assessments of sentencing, parole, and probation decisions rely on prior criminal record (Hoffman and Beck, 1974; Monahan and Skeem, 2015). An influential meta-analysis of the predictors of adult offender recidivism by Gendreau, Little, and Goggin (1996) examined 131 prior studies and found criminal history to be among the strongest predictors of future criminal activity. Similarly, DeLisi et al. (2013) found that prior police contact and arrest were predictive of future violent crime.

Gender. The conclusion that men are disproportionately responsible for violent crime is probably the best supported assertion in all of criminology (Braithwaite, 1989; DeLisi et al., 2013; Gendreau, Little, and Goggin, 1996). Arrest, court, and prison statistics for the United States all show massive overrepresentation of men. There is also evidence that men are overrepresented in acts of violent terrorism (Laqueur, 1977; McCauley and Segal, 1987; however, see Bloom, 2012; Cunningham, 2003; Zedalis, 2004 for studies of women in terrorism). For example, Bakker (2006) found that only 5 of 242 jihadi terrorists operating in Europe since 2001 (2.1%) were women.

Age. The idea that violent crime is closely associated with young people is also one of the most widely held empirical claims in criminology (Farrington, 2003; Marvell and Moody, 1991; Sweeten, Piquero, and Steinberg, 2013). The relationship between age and crime (known as the age-crime curve) is perceived to be so strong in criminology that Matza (1964) equates crime desistance with “maturational reform.” The age-crime curve also implies that the relationship between age and crime does not have a linear functional form (Hirschi and Gottfredson, 1983) and involvement in crime declines rapidly from its peak.

Although there is far less research, there is evidence that participation in political violence may also disproportionately be the work of young people (Berrebi, 2007; Silke, 2008). For example, Pape (2005) found that the average age of offenders in his study of suicide terrorists ranged from a low of 21.1 years for the Lebanese Hezbollah to 29.8 years for Chechens. However, in a study of 600

This project has received funding from the European Union's Horizon 2020 research and innovation programme under grant agreement N° 699824.

American Islamist extremists, Klausen, Morrill, and Libretti (2016) found that the median age for commission of violence was older and occurred across a broader age range than was the case for offenders who had committed more typical violent crimes (see also Pyrooz et al., 2017).

Mental illness. Much work in criminology has concluded that there is a positive relationship between previous mental illness and propensity to crime. For example, Cocozza, Melick, and Steadman (1978) compared the arrest rates of those suffering from mental illness, ex-offenders, and the general population and found that individuals with mental illnesses had a higher rate of arrest for violent offenses than the general population, but a lower rate of arrest than ex-offenders. The group differences remained even when prior arrest histories and age, the two variables most closely associated with recidivism, were controlled. Terrorism researchers have studied mental illness for several decades, but have thus far failed to reach a consensus on its role in shaping violent political behavior (see Gill and Corner [2016] for a review). For example, studies by McCauley and Moskalenko (2011) and Nijboer (2012) conclude that terrorist perpetrators are psychologically stable. However, recent research suggests that while extremists may not exhibit psychopathy, mental illness may indeed be related to extremism as an important causal factor that combines with others to produce complex pathways to political violence (Gill and Corner, 2016). Indeed, several recent studies have found empirical links between mental illness and violent political behavior, especially for unaffiliated loners (Bakker and de Graaf, 2010; Gill, Horgan, and Deckert, 2014; Spaaij, 2011). Gruenewald, Chermak, and Freilich (2013) compared homicides committed by far-right loner extremists to homicides committed by other types of far-right extremists in the United States. The authors found that 40% of far-right loners had a reported history of mental illness compared to only 8% for other far-rightists.

From crime-related background studies, we assess the relationship of criminal history, gender, age, and mental illness to violent extremism.

This project has received funding from the European Union's Horizon 2020 research and innovation programme under grant agreement N° 699824.

4.3.4 PIRUS Dataset

Data are drawn from the newly available individual-level database, Profiles of Individual Radicalization in the United States (PIRUS; Jensen et al., 2016), which is based on publicly available sources and contains background, demographic, group affiliation and contextual information for 1,473 individuals who radicalized in the United States from 1948 to 2013. The individuals in the database were included for committing ideologically motivated illegal violent or non-violent acts, joining a designated terrorist organization, or associating with an organization whose leader(s) has/have been indicted of an ideologically motivated violent offense. We define these cases as "domestic radicalization" in that most or all of the individuals' radicalization occurred while they were residing in the United States. All data were collected using publicly available sources, including court documents, online news articles, newspaper archives, open-source non-government reports (e.g., the Southern Poverty Law Center), unclassified government reports (e.g., annual FBI terrorist reports), and existing terrorism-related datasets (e.g., the Global Terrorism Database).

The dependent variable (violent/non-violent) is coded "1" for individuals whose earliest public exposure, that is, the action they undertook that resulted in their initial identification in public sources as an extremist, involved an act of violence or the intention to commit an act of violence. Likewise, the dependent variable is coded "0" for individuals whose earliest public exposure involved only non-violent acts. We treat as violent those cases where there is strong evidence that individuals were conspiring to kill or injure even if they failed to do so. We treat as non-violent all cases where it is clear from source documents that individuals did not intend to harm others, including acts of vandalism, illegal protest, fraud, and acts of property destruction where the perpetrators took measures to ensure that no one was injured or killed.

Stable employment is coded "1" for individuals who worked regularly prior to engaging in acts of extremism and "0" for individuals who were unemployed, alternated between periods of employment and unemployment, or habitually changed careers in the years leading up to their involvement in extremism.

Education is an ordinal measure that captures the highest level of education attained by individuals at the time they engaged in illegal acts. Individuals were coded "3" if they received a college degree

This project has received funding from the European Union's Horizon 2020 research and innovation programme under grant agreement N° 699824.

or higher, “2” if they attended college but did not graduate, “1” if they finished high school, or “0” if they did not graduate high school.

Marital status is coded “1” for individuals with a legally-recognized spouse or domestic partner and “0” for divorced or widowed individuals and those who never married.

Individuals who were once active in the military, but were discharged before they engaged in extremism were coded “1” for *past military experience* and “0” otherwise. Those on active military duty at the time of their public exposure were coded “1” for *active in military* and “0” otherwise.

We coded individuals who were part of close-knit, insular cliques with others who share similar extremist views as “1” for *radical peers* and “0” otherwise. We coded individuals who have immediate or extended family members who participated in illegal non-violent or violent ideologically-motivated behavior as “1” for *radical family* and “0” otherwise.

We coded *mental illness* as “1” if there is evidence that an individual suffered from mental illness of any kind, including schizophrenia, bipolar disorder, post-traumatic stress disorder, or major depression and “0” otherwise. We coded mental illness as positive based on either clinical diagnosis or self-reports and testimony by family or friends.

To test the argument that individuals are more likely to engage in violence when they are members of competing groups (e.g., Bloom, 2004), we coded *rival groups* as “1” for individuals who were members of an extremist group that suffered from internal splintering or competed with like-minded organizations for status, prestige, or resources and “0” otherwise. Evidence of internal competition included fractionalization within the group, acts or threats of violence between group members, and leadership turnover due to disagreements about group behavior or goals. We also coded this variable positively if there was evidence of violence between rival groups.

Prior criminal activity is an ordinal measure coded “1” for participation in violent crime, “0.5” for participation in non-violent crime, and “0” for no criminal participation prior to involvement in extremist activities. We relied primarily on official records for evidence of criminal behavior prior to

This project has received funding from the European Union’s Horizon 2020 research and innovation programme under grant agreement N° 699824.

involvement in extremist activities, but also treat self-reporting of criminal activities that did not lead to arrest as positive evidence of criminal behavior.

Gender is coded as a dichotomy ("1" = men; "0" =women) and *age* is a continuous measure recorded at the time at which the individual's extremist activities first became public knowledge (e.g., they were arrested, their plot materialized). To control for the possibility that the effects of age are curvilinear, we will include an *age-squared* term in the regression models. To control for violence-justifying beliefs that may be present across the political spectrum we also include several ideology measures. These measures are binary variables that categorize individuals along far right, far left, radical Islamist, or single-issue ideological milieus (the categories are mutually exclusive). We also include a series of dichotomous variables that capture the decade in which the individuals in the database came to public attention.

4.3.5 METHODOLOGY

This report contains a preliminary descriptive analysis of 12 variables, including univariate distributions and bivariate correlations between the variables and violent extremism. We also control for the effects of ideology and exposure year in our analysis. The final report will build on this analysis and feature a series of multivariate regression models run across four versions of the PIRUS dataset, each of which are based on different techniques to compensate for missing data.

4.3.6 UNIVARIATE AND BIVARIATE RESULTS

Descriptive statistics and information on missing values for all variables included in the analysis are shown in Table 1. While our dependent variable—violent/non-violent—and several other variables (gender, ideology, and exposure year) include no missing values, the remaining variables have a wide range of missing data. Our strategy for how we will deal with this issue is discussed in Section 4.

This project has received funding from the European Union's Horizon 2020 research and innovation programme under grant agreement N° 699824.

Table 33

Coding details and descriptive statistics for independent variables

Variable	Code	Distribution/Mean (SD)	Valid N	% missing values
Violent	No (0)<br>Yes (1)	48.3%<br>51.7%	1473	0.0%
Stable employment history	No (0)<br>Yes (1)	13%<br>87%	571	61.2%
Education	Did not complete high school (0)<br>High school diploma (1)<br>Some college, no college degree (2)<br>College degree or higher (3)	15.8%<br>21.4%<br>19.6%<br>43.2%	547	62.9%
Married	No (0)<br>Yes (1)	57%<br>43%	722	51.0%
Past military experience	No (0)<br>Yes (1)	85.7%<br>14.3%	856	41.9%
Active military	No (0)<br>Yes (1)	95.5%<br>4.5%	856	41.9%
Radical peers	No (0)<br>Yes (1)	44.3%<br>55.7%	867	41.1%
Radical family member(s)	No (0)<br>Yes (1)	41.5%<br>58.5%	294	80.0%
Mental illness	No (0)<br>Yes (1)	56.3%<br>43.7%	284	80.7%
Rival groups	No (0)	64.2%	542	63.2%

This project has received funding from the European Union's Horizon 2020 research and innovation programme under grant agreement N° 699824.

	Yes (1)	35.8%		
Prior criminal activity	No (0)<br>Prior, non-violent minor crime (0.5)<br>Prior non-violent serious crime or violent crime (1)	45.9%<br>24.3%<br>29.8%	678	54.0%
Gender	Female (0)<br>Male (1)	10.0%<br>90.0%	1473	0.0%
Age	Numerical	34.18 (13.22)	1395	5.3%
Islamist ideology	No (0)<br>Yes (1)	84.9%<br>15.1%	1473	0.0%
Far right ideology	No (0)<br>Yes (1)	56.5%<br>43.5%	1473	0.0%
Far left ideology	No (0)<br>Yes (1)	79.4%<br>20.6%	1473	0.0%
Single Issue ideology	No (0)<br>Yes (1)	79.2%<br>20.8%	1473	0.0%
Exposure 1950s	No (0)<br>Yes (1)	99.1%<br>0.9%	1473	0.0%
Exposure 1960s	No (0)<br>Yes (1)	92.5%<br>7.5%	1473	0.0%
Exposure 1970s	No (0)<br>Yes (1)	86.9%<br>13.1%	1473	0.0%
Exposure 1980s	No (0)<br>Yes (1)	83.6%<br>16.4%	1473	0.0%
Exposure 1990s	No (0)<br>Yes (1)	79.8%<br>20.2%	1473	0.0%
Exposure 2000s	No (0)<br>Yes (1)	69.4%<br>30.6%	1473	0.0%
Exposure 2010s	No (0)<br>Yes (1)	88.9%<br>11.1%	1473	0.0%

This project has received funding from the European Union's Horizon 2020 research and innovation programme under grant agreement N° 699824.

Note: % Missing values refers to PIRUS dataset untreated by imputation techniques.

ABBREVIATIONS: N = sample size; SD = standard deviation.

In an analysis of bivariate correlations between each variable of interest and involvement in violent extremism (as opposed to non-violent activity), we find that 10 out of 12 (83.3%) of the variables have statistically significant bivariate relationships (see Table 2). The results show that individuals who are married and have achieved stable employment and advanced educations are significantly less likely to engage in violent extremism. Conversely, we find that individuals who are embedded in social networks of radical peers or family members and have military experience, criminal histories, and suspected mental illness are more likely to plan, prepare for, or engage in acts of political violence. Being young and male is also positively correlated with violent extremism. The only two variables that did not produce a statistically significant relationship with violent extremism were those related to past military records and rival groups. Overall, the bivariate results indicate that the majority of the independent variables included in this study are correlated with individual-level violence in a way that supports further investigation using multivariate regression techniques.

Table 34

**Bivariate correlations between dependent variable (violence) and independent variables**	
**Variable**	**Correlation coefficient**
Stable employment history	–.125***
Education	–.118**
Married	–.074*
Past military experience	.019
Active military	.057*
Radical peers	.141***
Radical family	.096**
Mental illness	.107***
Rival groups	–.003
Prior criminal activity	.104***

This project has received funding from the European Union's Horizon 2020 research and innovation programme under grant agreement N° 699824.

Gender	.104***
Age	–.078**
Age (squared)	–.072**
Islamist	.118***
Far Right	.092***
Far Left	–.171***
Single issue	–.046
Exposure 1950s	.009
Exposure 1960s	.000
Exposure 1970s	.029
Exposure 1980s	.047
Exposure 1990s	–.082***
Exposure 2000s	–.051
Exposure 2010s	.090***

Note: Pooled results of bivariate correlations using the Expected Maximization model (N=1,473). *p≤.05; **p≤.01; ***p≤.001

4.3.7 MULTIVARIATE ANALYSIS

In the next stage, the research team conducted Hierarchical logistic regression

Models. A total of four different models were carried based on differing approaches to missing data:

- *expected maximization*
- *multiple imputation*
- *subgroup mean*
- *fixed value substitution*

As reported in Table 1, the variables in this study have a wide range of missing data. At the low extreme, variables such as gender, ideology, exposure year, and age have almost no missing data. At the other extreme, radical family and mental illness are missing data in more than 80% of the cases. Although this situation is common in studies of political violence based on open-source data (Chermak et al., 2012; Dugan and Distler, 2016; Safer-Lichtenstein, LaFree, and Loughran, 2017), it raises obvious concerns.

This project has received funding from the European Union's Horizon 2020 research and innovation programme under grant agreement N° 699824.

Nevertheless, as can be seen in the following models, the four different approaches produced very similar results.

Variable	Expected Maximization model			Regression-based multiple imputation model			Subgroup mean substitution model			Fixed value substitution model		
	B		SE	B		SE	B		SE	B		SE
Stable employment history	***–.555***	**	***(.206)***	***–.527***	**	***(.188)***	***–.637***	**	***(.207)***	***–.494***	**	***(.185)***
Education	–.086		(.097)	–.100		(.103)	–.091		(.090)	–.059		(.075)
Married	–.326		(.184)	–.366		(.200)	–.196		(.146)	–.230		(.149)
Past military experience	.024		(.229)	.000		(.234)	.184		(.218)	.115		(.233)
Active in military	.452		(.322)	.408		(.319)	.413		(.318)	.061		(.308)
Radical peers	***.878***	***	***(.167)***	***.819***	***	***(.135)***	***.803***	***	***(.155)***	***.689***	***	***(.128)***
Radical family	–.492		(.389)	–.408		(.350)	–.285		(.299)	–.005		(.199)
Mental illness	***.750***	**	***(.237)***	***.747***	***	***(.231)***	***.776***	***	***(.229)***	***.666***	**	***(.224)***
Rival groups	–.246		(.251)	–.100		(.293)	–.177		(.206)	–.143		(.177)

This project has received funding from the European Union's Horizon 2020 research and innovation programme under grant agreement N° 699824.

Prior criminal activity	***.471***	**	***(.176)***	***.428 ****	*	***(.194)***	***.550***	***	***(.169)***	***.564***	***	***(.170)***
Gender	.341		(.213)	.330		(.225)	.317		(.204)	.281		(.209)
Age	–.024		(.025)	– .019		(.025)	– .045		(.023)	– .030		(.024)
N	1473			1473			1473			1395		

The results indicate that there are four significant predictors of engaging in violent political extremism:

- Unstable employment history
- Radical peers
- Record of mental illness
- Criminal record

This project has received funding from the European Union's Horizon 2020 research and innovation programme under grant agreement N° 699824.

4.3.8 References

Akers, Ronald L. 2009. *Social Learning and Social Structure: A General Theory of Crime and Deviance*. New Brunswick, NJ: Transaction Publishers.

Allison, Graham T. 1971. *Essence of Decision: Explaining the Cuban Missile Crisis*. Boston, MA: Little, Brown.

Andridge, Rebecca R., and Roderick J. A. Little. 2010. A Review of Hot Deck Imputation for Survey Non-Response. *International Statistical Review* 78:40–64.

Bakker, Edwin. 2006. "Jihadi Terrorists in Europe, Their Characteristics and the Circumstances in Which They Joined the Jihad: An Exploratory Study." *Clingendael Security Paper*, No. 2, The Hague, the Netherlands: Netherlands Institute of International Relations.

Bakker, Edwin, and Beatrice de Graaf. 2010. "Lone Wolves: How to Prevent This Phenomenon?" The Hague, the Netherlands: International Centre for Counter-Terrorism. Retrieved January 9, 2017 from https://openaccess.leidenuniv.nl/bitstream/handle/1887/16557/ICCT%2520EM%2520Lone%2520Wolves%2520Paper.pdf?sequence=2.

Berrebi, Claude. 2007. "Evidence about the Link between Education, Poverty and Terrorism among Palestinians." *Peace Economics, Peace Science and Public Policy* 13:1–36.

Biberman, Yelena, and Farhan Zahid. 2016. "Why Terrorists Target Children: Outbidding, Desperation, and Extremism in the Peshawar and Beslan School Massacres." *Terrorism and Political Violence*: 1–16.

Bion, Wilfred Ruprecht. 1961. *Experiences in Groups: And Other Papers*. New York, NY: Tavistock Publications.

Bloom, Mia. 2004. "Palestinian Suicide Bombing: Public Support, Market Share, and Outbidding." *Political Science Quarterly* 119:61–88.

This project has received funding from the European Union's Horizon 2020 research and innovation programme under grant agreement N° 699824.

Bloom, Mia. 2007. *Dying to Kill: The Allure of Suicide Terror*. New York, NY: Columbia University Press.

Bloom, Mia. 2012. *Bombshell: Women and Terrorism*. Philadelphia, PA: University of Pennsylvania Press.

Blumstein, Alfred, Jacqueline Cohen, and David P. Farrington. 1988. "Criminal Career Research: Its Value for Criminology." *Criminology* 26:1–35.

Borowitz, Albert. 2005. *Terrorism for Self-Glorification: The Herosratos Syndrome*. 1st edition. Kent, Ohio: Kent State University Press.

Borum, Randy. 2011. "Radicalization into Violent Extremism I: A Review of Social Science Theories." *Journal of Strategic Security* 4: 7–36.

Braithwaite, John. 1989. *Crime, Shame and Reintegration*. New York, NY: Cambridge University Press.

Chermak, Steven M., Joshua D. Freilich, William S. Parkin, and James P. Lynch. 2012. "American Terrorism and Extremist Crime Data Sources and Selectivity Bias." *Journal of Quantitative Criminology* 28:191–218.

Cocozza, Joseph J., Mary Evans Melick, and Henry J. Steadman. 1978. "Trends in Violent Crime Among Ex-Mental Patients." *Criminology* 16: 317–34.

Cronin, Audrey Kurth. 2011. *How Terrorism Ends: Understanding the Decline and Demise of Terrorist Campaigns*. Princeton, NJ: Princeton University Press.

Cunningham, Karla J. 2003. "Cross-Regional Trends in Female Terrorism. *Studies in Conflict & Terrorism.*" 26:171–95.

This project has received funding from the European Union's Horizon 2020 research and innovation programme under grant agreement N° 699824.

DeLisi, Matt, Tricia K. Neppl, Brenda J. Lohman, Michael G. Vaughn, and Jeffrey J. Shook. 2013. "Early Starters: Which Type of Criminal Onset Matters Most for Delinquent Careers?" *Journal of Criminal Justice* 41:12–17.

Della Porta, Donatella, and Gary LaFree. 2012. "Guest Editorial: Processes of Radicalization and De-Radicalization." *International Journal of Conflict and Violence* 6 (1): 4–10.

Dugan, Laura, and Michael Distler. 2016. "Measuring Terrorism." In *The Handbook of the Criminology of Terrorism*, eds. Gary LaFree and Joshua D. Freilich, New York, NY: Wiley-Blackwell.

Farrington, David P. 2003. "Developmental and Life-Course Criminology: Key Theoretical and Empirical Issues-The 2002 Sutherland Award Address." *Criminology* 41:221–25.

Findley, Michael G., and Joseph K. Young. 2012. "More Combatant Groups, More Terror?: Empirical Tests of an Outbidding Logic." *Terrorism and Political Violence* 24:706–21.

Gendreau, Paul, Tracy Little, and Claire Goggin. 1996. "A Meta-Analysis of the Predictors of Adult Offender Recidivism: What Works!" *Criminology* 34:575–608.

Gill, Paul. 2015. "Toward a Scientific Approach to Identifying and Understanding Indicators of Radicalization and Terrorist Intent: Eight Key Problems." *Journal of Threat Assessment and Management*, *Terrorism in Europe*, 2: 187–91.

Gill, Paul, and Emily Corner. 2016. "Lone-Actor Terrorist Target Choice." *Behavioral Sciences & the Law* 34:693–705.

Gill, Paul, John Horgan, and Paige Deckert. 2014. "Bombing Alone: Tracing the Motivations and Antecedent Behaviors of Lone-Actor Terrorists." *Journal of Forensic Sciences* 59:425–35.

Githens-Mazer, Jonathan, and Robert Lambert. 2010. "Why Conventional Wisdom on Radicalization Fails: The Persistence of a Failed Discourse." *International Affairs* 86: 889–901.

This project has received funding from the European Union's Horizon 2020 research and innovation programme under grant agreement N° 699824.

Gottfredson, Michael, and Travis Hirschi. 1990. *A General Theory of Crime*. 1st edition. Stanford, CA: Stanford University Press.

Gruenewald, Jeff, Steven Chermak, and Joshua D. Freilich. 2013. "Distinguishing 'Loner' Attacks from Other Domestic Extremist Violence." *Criminology & Public Policy* 12:65–91.

Hirschi, Travis. 1969. *Causes of Delinquency*. Berkeley, CA: University of California Press.

Hoffman, Peter B., and James L. Beck. 1974. "Parole Decision-Making: A Salient Factor Score." *Journal of Criminal Justice* 2:195–206.

Honaker, James, and Gary King. 2010. "What to Do About Missing Values in Time-Series Cross-Section Data." *American Journal of Political Science* 54:561–81.

Horgan, John. 2008. "From Profiles to Pathways and Roots to Routes: Perspectives from Psychology on Radicalization into Terrorism." *The ANNALS of the American Academy of Political and Social Science* 618: 80–94.

Horgan, John. 2009. "Individual Disengagement: A Psychological Analysis." In *Leaving Terrorism Behind: Individual and Collective Disengagement*, 17–29. Political Violence. New York, NY: Routledge.

Janis, Irving Lester. 1972. *Victims of Groupthink: A Psychological Study of Foreign-Policy Decisions and Fiascoes*. Boston, MA: Houghton Mifflin Company.

Jensen, Michael, Patrick James, Gary LaFree, Anita Atwell-Seate, Daniela Pisoiu, and John Stevenson. 2016. "Empirical Assessment of Domestic Radicalization (EADR)." Final Report National Institute of Justice, Award Number 2012–ZA–BX–0005. National Consortium for the Study of Terrorism and Responses to Terrorism (START), College Park, MD.

This project has received funding from the European Union's Horizon 2020 research and innovation programme under grant agreement N° 699824.

Jones, Edward E., and Victor A. Harris. 1967. “The Attribution of Attitudes.” *Journal of Experimental Social Psychology*, 1–24.

King, Gary, James Honaker, Anne Joseph, and Kenneth Scheve. 2001. “Analyzing Incomplete Political Science Data: An Alternative Algorithm for Multiple Imputation.” *American Political Science Review* 95:49–69.

Klausen, Jytte, Tyler Morrill, and Rosanne Libretti. 2016. “The Terrorist Age-Crime Curve: An Analysis of American Islamist Terrorist Offenders and Age-Specific Propensity for Participation in Violent and Nonviolent Incidents.” *Social Science Quarterly* 97:19–32

Kruglanski, Arie W., Xiaoyan Chen, Mark Dechesne, Shira Fishman, and Edward Orehek. 2009. “Fully Committed: Suicide Bombers’ Motivation and the Quest for Personal Significance.” *Political Psychology* 30: 331–57.

Kydd, Andrew H., and Barbara F. Walter. 2006. “The Strategies of Terrorism.” *International Security* 31:49–80.

Laqueur, Walter. 1977. *Terrorism*. Boston, MA: Little, Brown.

Laub, John H., and Robert J. Sampson. 1993. “Turning Points in the Life Course: Why Change Matters to the Study of Crime.” *Criminology* 31:301–25.

Lemieux, Anthony F., and Victor H. Asal. 2010. “Grievance, Social Dominance Orientation, and Authoritarianism in the Choice and Justification of Terror versus Protest.” *Dynamics of Asymmetric Conflict* 3: 194–207.

Loeber, Rolf, and Marc Le Blanc. 1990. “Toward a Developmental Criminology.” *Crime and Justice* 12:375–473.

Marvell, Thomas B., and Carlisle E. Moody. 1991. “Age Structure and Crime Rates: The Conflicting Evidence.” *Journal of Quantitative Criminology* 7:237–73.

This project has received funding from the European Union’s Horizon 2020 research and innovation programme under grant agreement N° 699824.

Matza, David. 1964. *Delinquency and Drift: From the Research Program of the Center for the Study of Law and Society, University of California, Berkeley*. New York, NY: Wiley.

McCauley, Clark. 1989. “The Nature of Social Influence in Groupthink: Compliance and Internalization.” *Journal of Personality and Social Psychology* 57:250–60.

McCauley, Clark, and Sophia Moskalenko. 2011. *Friction: How Radicalization Happens to Them and Us*. New York, NY: Oxford University Press.

McCauley, Clark R., and M. E. Segal. 1987. “Social Psychology of Terrorist Groups.” In *Group Processes and Intergroup Relations: Review of Personality and Social Psychology*, eds. Dominic Abrahms and Michael A. Hogg, Newbury Park, CA: Sage.

Monahan, John, and Jennifer L. Skeem. 2015. “Risk Assessment in Criminal Sentencing.” *Virginia Public Law and Legal Theory Research Paper*, No. 53.

Neumann, Peter, and Scott Kleinmann. 2013. “How Rigorous Is Radicalization Research?” *Democracy and Security* 9: 360–82.

Nijboer, Matthijs. 2012. “A Review of Lone Wolf Terrorism: The Need for a Different Approach. Article.” *Social Cosmos*, 3.

Pape, Robert. 2005. *Dying to Win: The Strategic Logic of Suicide Terrorism*. New York, NY: Random House Publishing Group.

Post, Jerrold M. 1998. “Terrorist Psycho-Logic: Terrorist Behavior as a Product of Psychological Forces.” In *Origins of Terrorism: Psychologies, Ideologies, Theologies, States of Mind*, ed. Walter Reich, Washington, DC: Woodrow Wilson Center Press.

This project has received funding from the European Union's Horizon 2020 research and innovation programme under grant agreement N° 699824.

Pyrooz, David, Gary LaFree, Scott Decker, and Patrick A. James. 2017. “Cut from the Same Cloth? A Comparative Study of Gang Members and Domestic Extremists in the United States.” *Justice Quarterly* (May): 1-32.

Rubin, Donald B. 2004. *Multiple Imputation for Nonresponse in Surveys*. Hoboken, NJ: Wiley-Interscience.

Sabini, John. 1995. *Social Psychology*. Norton.

Safer-Lichtenstein, Aaron, Gary LaFree, and Thomas A. Loughran. 2017. “Studying Terrorism Empirically: What We Know about What We Don’t Know.” Unpublished.

Sageman, Marc. 2004. “Joining the Jihad.” In *Understanding Terror Networks*, 99–135. Philadelphia, PA: University of Pennsylvania Press.

Silke, Andrew. 2008. “Holy Warriors Exploring the Psychological Processes of Jihadi Radicalization.” *European Journal of Criminology* 5:99–123.

Spaaij, Ramon. 2011. *Understanding Lone Wolf Terrorism: Global Patterns, Motivations and Prevention*. Springer Science & Business Media.

Sweeten, Gary, Alex R. Piquero, and Laurence Steinberg. 2013. “Age and the Explanation of Crime, Revisited.” *Journal of Youth and Adolescence* 42:921–38.

Tsikriktsis, Nikos. 2005. “A Review of Techniques for Treating Missing Data in OM Survey Research.” *Journal of Operations Management* 24:53–62.

Warr, Mark, and Mark Stafford. 1991. “The Influence of Delinquent Peers: What They Think or What They Do?” *Criminology* 29:851–66.

Warr, Mark. 2002. *Companions in Crime: The Social Aspects of Criminal Conduct*. Cambridge, UK; New York, NY: Cambridge University Press.

This project has received funding from the European Union’s Horizon 2020 research and innovation programme under grant agreement N° 699824.

Wiktorowicz, Quintan, ed. 2004. *Islamic Activism: A Social Movement Theory Approach*. Bloomington, Ind: Indiana University Press.

Wiktorowicz, Quintan. 2005. *Radical Islam Rising: Muslim Extremism in the West*. Oxford: Rowman & Littlefield.

Zedalis, Debra D. 2004. *Female Suicide Bombers*. Carlisle, PA: Strategic Studies Institute, U.S. Army War College.

This project has received funding from the European Union's Horizon 2020 research and innovation programme under grant agreement N° 699824.

4.4 T2.7: Socio-economic background of terrorism suspects in Europe

4.4.1 SUMMARY AND CONCLUSION

The project aims at identifying economic drivers of terrorism involvement. The objective is to develop and apply an innovative theory to study the relation between indicators of (perceived and real) economic adversity and involvement in terrorism, radicalization and extremism in general. The project consists of quantitative and qualitative analyses. The results of the quantitative analyses suggest that terrorist offenders are relatively often low educated, unemployed, from ethnic minorities, and involved in other offenses than the general population. It appears that socio-economic variables, in particular being low educated and losing a job, increase the chance to become suspected of a terrorist offense, net of demographic variables and previous involvement in criminal behavior. Qualitative data suggest that suspects of terrorist activities perceive their adversities more as injustice and discrimination than other offenders. This may be related to traumatic youth experiences and broken families which may have boosted the impact of discrimination and adversity on attitudes and behaviour.

This project has received funding from the European Union's Horizon 2020 research and innovation programme under grant agreement N° 699824.

4.4.2 THEORETICAL FRAMEWORK

We use a top to bottom threat model to understand which socio-economic factors correspond to radicalization and why they correlate to individual involvement in terrorism (described in Ljujic et al., 2017). The model (Figure 1) builds upon psychological and criminological notions of perceived threat (Stephan, 2014; Schmid & Muldoon, 2015); strain (Agnew, 2016) and significance loss (Kruglanski, et al., 2014).

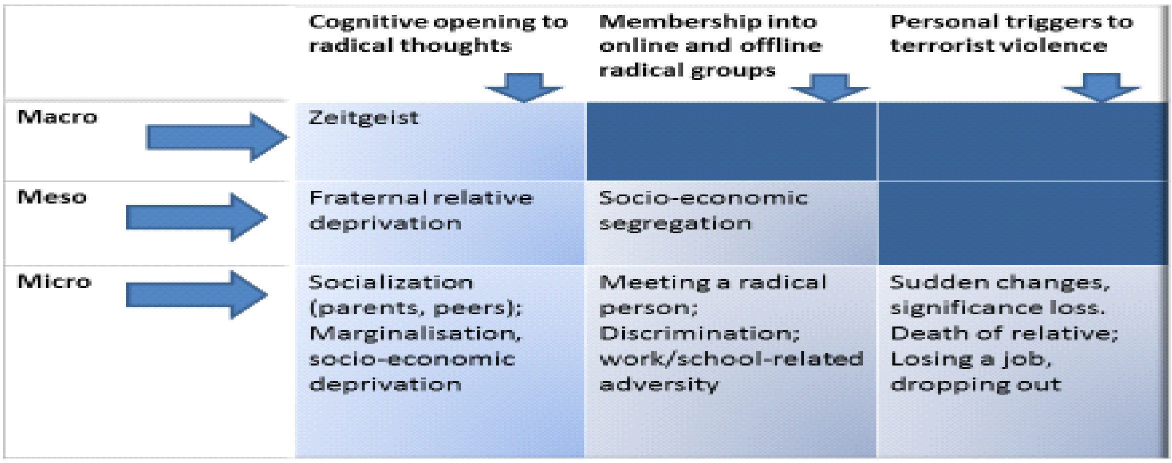

Figure 16 Top to bottom threat model of radicalization to terrorism

4.4.3 EXPLORATORY STUDY USING OPEN SOURCES

We started with exploratory analyses using secondary data from open-source data on recent European terrorist attacks. These findings can be summarized as follows:

- 100% of perpetrators were men
- 70% belong to second-generation immigrants.
- 50% completed only secondary education or less. Less than 15% attended university or vocational university / college.
- 70% were unemployed or at school at the time of attack.
- 50% were previously involved in violent crimes or had joined foreign fighters' jihadi groups abroad.
- 33% of the sample radicalized in prison.

This project has received funding from the European Union's Horizon 2020 research and innovation programme under grant agreement N° 699824.

4.4.4 QUANTITATIVE STUDY ON DUTCH TERRORIST SUSPECTS

To get a more detailed and robust insight into the socio-economic drivers of terrorism, an anonymized register database on Dutch terrorist suspects have been retrieved and combined with relevant population data from Statistics Netherlands (CBS). The 'terrorist' sample (*n*=279) consists of Dutch suspects of terrorist activities since 2004, when the Criminal Law Code on terrorist acts was introduced. The control sample (*n*=279) consists of non-terrorist suspects of other offenses (that are comparable to terrorist suspects along the categories of age and gender), and of a sample (n=279) from the general population that is comparable with regard to age and gender.

Table 1 Demographics, SES and previous involvement in crime among terrorist suspects, general suspects and general population (samples are matched on gender and age)

		Terrorist suspects (n=279)	Other offenders (n=279)	General population (n=279)
Origin	Non-immigrant<br>1st generation<br>2nd generation	18.6 % (52)<br>41.2 % (115)<br>40.1 % (112)	58.1 % (162)<br>24.7 % (69)<br>17.2 % (48)	70,3 % (196)<br>21.9 % (61)<br>7.8 % (22)
Education level	 Low<br>Middle<br>High	(n=170)<br>62.4 % (106)<br>33.5 % (57)<br>4.1 % (7)	(n=190)<br>64.7 % (123)<br>31.6 % (60)<br>3.7 % (7)	(n=182)<br>34.1 % (62)<br>42.3 % (77)<br>23.6 % (43)
Employed	 Yes<br>No	(n=250)<br>44.0 % (110)<br>56.0 % (140)	 49.8 % (139)<br>50.2 % (140)	 60.6 % (169)<br>39.4 % (110)
Lost job in year before terrorist activities	Yes<br>No change / unknown	11.5 % (32)<br>88.5 % (247)	4.7 % (13)<br>95.3 % (266)	2.9 % (8)<br>97.1 % (271)

This project has received funding from the European Union's Horizon 2020 research and innovation programme under grant agreement N° 699824.

Criminal offenses in the year before	Yes<br>No	(n=233)<br>29.2 % (68)<br>70.8 % (165)	20.4 % (57)<br>79.6 % (222)	1.8 % (5)<br>98.2 % (274)
Multiple criminal offenses until the year before	Yes<br>No	(n=233)<br>12.9 % (30)<br>87.1 % (203)	8.2 % (23)<br>91.8 % (256)	0.0 % (0)<br>100 % (279)

The table shows that suspects of terrorist activities have a migrant background more often than the general population and other offenders. They have followed lower levels of education than the general population. Suspects from terrorist offenses are also more often unemployed or still in education than the general population and also lost a job more often in the year before they became suspect. Their economic position is even worse than that of the general offender sample. Finally, a substantial portion of the terrorist suspects were involved in general types of offending in the year before they became involved in terrorism, much more than the general population and even slightly more than the general offender sample.

In a next analysis, we analysed to which extent major socio-economic variables had an independent and significant relation with being suspect of terrorism. To enable this more complex analysis, we selected a larger control sample from the population data from Statistic Netherlands, again matched on gender and age. This control sample consists of 6,004 persons. We included 204 suspects from terrorism in this analysis for whom we had enough information. Using this sample, we conducted a logistic regression analysis that included the major demographic and socio-economic variables. We also included previous criminal involvement and previous detention in this regression analysis. The results of this analysis are presented in table 2.

This project has received funding from the European Union's Horizon 2020 research and innovation programme under grant agreement N° 699824.

Table 2 Logistic regression analysis: independent effects of demographics, SES and previous involvement in crime on becoming a terrorism suspect

		B (SE)	Adjusted Odds ratio	95% Confidence Interval for the Odds Ratio
Constant		-4.096 (.117)***	.02	
Origin	1st generation<br>2nd generation	1.643 (.207)***<br>1.789 (.208)***	5.17<br>5.98	[3.45, 7.76]<br>[3.98, 8.99]
Education level	Low<br>Middle<br>No info (dummy)	1.694 (.441)***<br>1.084 (.446)*<br>.847 (.447)	5.44<br>2.96<br>2.33	[2.29, 12.92]<br>[1.23, 7.09]<br>[.97, 5.60]
Employed	Not in a job	.163 (.185)	1.18	[.82, 1.69]
Lost job in year before terrorist activities	Yes	.842 (.283)**	2.32	[1.33, 4.04]
Criminal offenses until the year before	Yes	1.950 (.248)***	7.03	[4.32, 11.43]
Multiple criminal offenses until the year before	Yes	.420 (.374)	1.52	[.73, 3.17]
Detention in the year before	Yes	1.081 (.381)**	2.95	[1.40, 6.22]

Nagelkerke's R^2: .240

Table 2 shows that most of the demographic, socio-economic and offending history variables are

This project has received funding from the European Union's Horizon 2020 research and innovation programme under grant agreement N° 699824.

independently related to becoming a suspect of terrorist activities. The results confirm that terrorist suspects more often have a migrant background, which can be explained by the jihadi background of most terrorist cases in the Netherlands. Interestingly, the socio-economic variables are also related to becoming a terrorism suspect, independently from demographic background (ethnicity and age/gender, for which the sample was matched), and criminal offending history. In particular, having followed a lower type of education increases the chance to become involved in terrorist activities (with an estimated odds ratio of more than 5). Somewhat contrary to the expectation, not having a job (which means being unemployed or still in school) did not have a significant effect. However, *losing* a job in the year before being a terrorism suspect did have significant effect, with an estimated odds ratio of more than 2. Further, criminal history was related to becoming a suspect of terrorism. In particular, being previously registered for any offense other than terrorism strongly increased the chance of becoming a terrorism suspect (estimated odds ratio of 7). Also having spent some time in detention before becoming a terrorism suspect enhances the chance of becoming a terrorism suspect.

This project has received funding from the European Union's Horizon 2020 research and innovation programme under grant agreement N° 699824.

4.4.5 QUALITATIVE STUDY ON DETAINED TERRORIST OFFENDERS

Qualitative data have been collected among 5 offenders/suspects in custody who are detained in special prison units for terrorist offenders, and among 12 key informants (warders, mentors, psychologists, defence lawyers) that are working with terrorism offenders. We also draw from secondary biographic material on Dutch terrorism offenders, and from information about non-terrorist offenders. The qualitative interviews addressed the individual life-course of terrorism suspects and other offenders, using 'life history calendars', focused on perceived or real economic adversities, and previous criminal involvement. We are still analysing this data, but the first impressions from the qualitative material suggest the following:

- Generally, the quantitative findings are supported in the interviews: terrorist suspects were often previously involved in offending (in petty crimes, not in organised or serious crime), were low educated and some of them lost their job. However, more factors should be taken into account. There seems to be a difference between 'leaders' and 'followers' in prison: the former can be relatively well educated, but latter often have a low IQ and are easy to manipulate.
- One factor that should be taken into account is the *perception* of discrimination and injustice, sometime triggered by explicit experiences of overt discrimination. Terrorist suspects seem to blame western society in general for their problems. They perceive a lot of injustice in their situation, also in their situation in detention. Their socio-economic life histories have parallels with the life histories of other offenders in detention (who are also often from a minority background). But terrorism suspects seem to perceive their adversities more as a result of injustice and discrimination.
- Often, terrorism suspect had a difficult youth with sometimes traumatic experiences. There are also several reports of losing contact with parents and the occurrence of broken families before suspects became radicalized. This seems to be happening relatively often among terrorist suspects, and may have boosted the *impact* of discrimination & adversity on their attitudes and behaviour.
- There are reports of psychological problems and disorders among terrorism suspects: e.g., depression, personality problems, anxiety. However, many other offenders also have a history of psychological and social problems. But terrorism suspects are also very active in

This project has received funding from the European Union's Horizon 2020 research and innovation programme under grant agreement N° 699824.

searching for meaning in life and for belonging (‘brotherhood’). This enhances the need for recognition and belonging, at the same time distrust towards others and society.

- There is no indication that jihadi radicalization is related to a strict orthodox religious education at home. On the contrary, it seems that the jihadi terrorist suspects found their way in religion on their own through internet and contacts with peers, while their parents often did not endorse strict religious beliefs. This is reflected by reprots that part of the terrorist jihadi suspects do not have a deep knowledge of their religion.

This project has received funding from the European Union’s Horizon 2020 research and innovation programme under grant agreement N° 699824.

4.4.6 REFERENCES

Agnew, R. (2016). *General Strain Theory and Terrorism*. In: LaFree, G., & Freilich, J. D. (eds.), The Handbook of the Criminology of Terrorism.

De Bie, J. (2016). *How Jihadist Networks Operate*. Leiden: University of Leiden.

Kruglanski, A. W., Gelfand, M. J., Bélanger, J. J., Sheveland, A., Hetiarachchi, M., & Gunaratna, R. (2014). The psychology of radicalization and deradicalization: How significance quest impacts violent extremism. *Political Psychology*, 35(1), 69-93.

Ljujic, V., van Prooijen, J. W., & Weerman, F. (2017). Beyond the crime-terror nexus: socio-economic status, violent crimes and terrorism. *Journal of Criminological Research, Policy and Practice*, online before print.

Schmid, K., & Muldoon, O. T. (2015). Perceived threat, social identification, and psychological well-being: The effects of political conflict exposure. *Political Psychology*, 36(1), 75-92.

Stephan, W. G. (2014). Intergroup anxiety: Theory, research, and practice. *Personality and Social Psychology Review*, 18(3), 239-255.

This project has received funding from the European Union's Horizon 2020 research and innovation programme under grant agreement N° 699824.

5 Conclusions of WP2

The original structure of PROTON as a collaborative model has successfully achieved its original objectives of identifying the social, economic and psychological risk factors for radicalization and recruitment to terrorism and in providing information needed for the development of the ABMs in WP5. In this WP, T2.1 synthesized the current literature together with the original PROTON studies from WP2 and provided the first quantitative classification of the primary social, economic and psychological risk factors for radicalization and recruitment to terrorism. WP2 also included a systematic review on protective factors, which provides corroboration for the identification of many of the risk factors. Of the four individual risk factor studies, two studies focused on the EU context. One study applied a qualitative approach to examining the effects of legitimacy and trust in the UK context (T2.4). The other study applied a mixed quantitative and qualitative examination of terrorism offenders in the Netherlands. In addition, WP2 included one study from Israel and another from the US. These studies provided for important comparisons which enabled moderator analysis to be carried out in T2.1 and the extrapolation of similarities and differences in risk factor effect sizes.

The inclusion of the innovative studies of T2.3, T2.5 and T2.7 enabled additional meta-analyses to be carried out and proved to be a successful integration of the collaborative structure that PROTON was originally designed and intended for. There is much that can and should be learned from this. Proper planning and identification of gaps in the body of scientific knowledge first informed the planned structure of PROTON. By following this plan as closely as possible, with ongoing and frequent contact with the task leaders, enabled all partners to maintain and stay on the same page. As the results of WP2 demonstrate, there is significant benefit in using such an approach when executed properly. For example, the evidence from the T2.6 report on protective factors appears to line up quite well with T2.1's findings on risk factors. For example, T2.1 found that institutional trust and legitimacy were important risk factors, together with poor integration, for moving from radicalization to recruitment. T2.6 found that increasing legitimacy and trust were protective factors. T2.4 found strong qualitative evidence to support these findings. The project's focus on the crime-terror nexus has identified that across all democratic settings, criminality and prior criminal involvement is arguably the single strongest risk factor for radicalization and recruitment to terrorism. Such evidence has potentially important implications for policy making.

This project has received funding from the European Union's Horizon 2020 research and innovation programme under grant agreement N° 699824.

5.1 EU specific context

The original structure of PROTON as a collaborative model that included studies specific to the US and Israel was challenged by many initially and its relevance has been questioned by many throughout. In science it is a well-known precept that one cannot tell how bias their sample is by looking at the sample alone. It is to this end that western countries continue to try and learn hard learned lessons from each other when it comes to terrorism. One of the challenges has to do with low even counts. It is quite difficult to carry out studies with a lack of incidents. It is to this end that Israel and the US provided good contexts for extrapolating region specific differences. In the framework of PROTON and the integration of the findings from the studies we can report the following risk factors as being specific to the EU context in ways that are apparently stronger or weaker than they are in other places.

This does not mean that necessarily education is not a risk factor in the EU because it has a stronger effect in the US. Nor does it mean that criminality is not a risk factor in the US because it has a stronger effect in the EU. Rather, by comparing different risk factors with each other and between settings we can create an evidence based list of priorities that is context specific and which understands the relative effect of different risk factors.

As has been identified, criminal history is one of the single greatest risk factors across all contexts. However, criminal history is stronger for recruitment to terrorism than it is for radicalization of recruitment in the US. The effect of criminal history is only now starting to be unravelled. The innovative studies of T2.3 and T2.7, combined with the findings of T2.1 show the value of disaggregating criminal histories. It could be that the EU has a lot to learn from the Israeli context in which a growing number of radicals are being arrested for low-level non-terrorism violence and other offences. The results identified in WP2 indicate that the EU and US contexts differ significantly on a number of factors and criminal history is one of those. The magnitude of the effect of prior offending in the EU is far more similar to what is seen in Israel than in the US. In both these studies, disaggregating criminal history points to an important direction in understanding what type of individuals start with criminal histories and move on to terrorism. In the case of the EU, recent arrestees, who have been detained in the last year were at the greatest risk of being re-incarcerated for terrorism related offences. In Israel, T2.3 uncovered evidence that those involved

This project has received funding from the European Union's Horizon 2020 research and innovation programme under grant agreement N° 699824.

in public disorder offences, which contextually is often linked to conflict based violence, were most likely to be subsequently incarcerated for terrorism related offences. In this respect, it has been observed that many foreign fighters and lone wolves from Europe have prior criminal histories and have been on the radar of intelligence services for some time. These similar findings stress the importance of monitoring and surveillance on known radicals, especially those previously incarcerated for radical related activities. Those recently released from incarceration stints appear to be at a heightened risk of offending.

In addition, a number of risk factors display important differences between the EU and other contexts. For example, with respect to radicalization of beliefs, education, being a victim of racism, and frequency of attendance of place of worship all have a stronger effect in the EU compared to other contexts. Conversely, mental health and social media usage, while important, have smaller effect sizes in the EU context compared to others. With respect to radicalization of actions, employment, differential associations, religious identity and collective relative deprivation all have stronger effects in the EU compared to other contexts. While the EU is certainly unique in many ways and demonstrates important differences in the effects of a range of risk factors, there are also many similarities with the US, Israel, and other western-democratic contexts. As such, while it is important to identify EU specific risk factors, important lessons remain to be learned from democratic partners.

This project has received funding from the European Union's Horizon 2020 research and innovation programme under grant agreement N° 699824.

6 Policy implications

To date, many anti-radicalization policies have been based on the conception that inhibiting radicalization of belief will invariably lead to a lessening of the risk that some small percentage cognitive radicals will inevitably turn to violence. This appears to be an intuitive and useful approach, however a more evidence based approach would leverage such policies to focus on those factors that have a greater effect on the move from radicalization of belief to radicalization of action in a very specific and focussed way. For example, whilst socio-economic, employment and anti-racism policies may seem intuitive, they will not necessarily lead to less radicalization in a significant way. The evidence from this review does however point to a few potential key areas in which policies and interventions may better be directed.

Education and employment

More opportunistic interventions based on the current evidence may consider how the university campus and experience can be leveraged to fight against radicalization. While not every campus may be equally radical or home to an equal number of radical individuals, the general demographic from which radicals are likely to emerge, and the time at which they are likely to emerge.

Since many studies point to under-employment rather than unemployment as a risk factor, especially combined with higher education, efforts should be made to reduce under-employment. One option could be to increase partnerships between employers and educational institutions, perhaps through government initiatives, to provide full-time students with immediate job opportunities and legitimate career paths without the downtime in between graduation and hiring. Internships and fellowships in government institutions could also help to increase integration and institutional trust. Increased focus should be placed overall on developing of employment skills during the university experiences. As T2.5 identifies, higher education when coupled with steady employment has a protective effect.

This project has received funding from the European Union's Horizon 2020 research and innovation programme under grant agreement N° 699824.

Immigration, Integration and legitimacy

Whilst being an immigrant is not one of the stronger risk factors for radicalization of belief, it does have a moderate effect for radicalization of actions. Additionally, it seems that immigrants and minority community members who may also be suffering from poor integration and a poor perception of state institutions may be at an especially high risk of radicalization of action. While EU and member states' policies include a focus on encouraging minorities to maintain their communities, customs and practices, more efforts need to be made to integrate such people into the wider community and state(s). Close, positive contacts with community workers and governmental employees may encourage institutional trust and legitimacy. Additionally, whilst social media may not be a significant risk factor in and of itself, it may be useful to leverage social media to promote minority language based outreach, services, and programming. There is likely also an important interaction between the education and employment outcomes and achievements for immigrants that may differ substantially compared to non-immigrants. As such, policies should seek to address employment and education opportunities bearing this in mind.

Collective relative deprivation

Whilst individual real socio-economic status is not a good predictor of radicalization or belief or the move to radical action, collective relative deprivation and collective economic discrimination should be targeted as sources of risk of radicalization. In this regard it should be understood that there are different types of relative deprivation. For some, relative deprivation is not a matter of what one's real situation is, but what one's imagines situation ought to be. For others, it is not a matter of real individual standing, but the generally perceived situation of a larger community or population with which one identifies. Yet for other, it is not a matter of what one or others have, but a matter of attaining goals that are perhaps unattainable through financial means, such as respect, standing and power. Relative deprivation is overwhelmingly dictated by perceptions as opposed to that which is actual and real.

Additionally, legitimacy and relative deprivation may be inter-related on many levels. Low institutional trust and legitimacy may blame those same institutions for the perceptively poor situation of an individual or community. Even in cases where the actual situation may not be so different or worse off than comparable groups, these perceptions can be reinforced by the situation of other communities in other cities, countries or regions. In this sense, relative deprivation may

This project has received funding from the European Union's Horizon 2020 research and innovation programme under grant agreement N° 699824.

inherently be linked to perceptions of collective discrimination and institutional discrimination. Policy makers therefore need to adopt an extra sense of sensitivity when designing policies at the community level. The potential for negative impacts on collective socio-psychological states of minority communities should be at the forefront of policy makers' considerations. At this level, individual experiences and encounters with different arms of the state—especially when individual encounters receive media attention—can have consequences that spread to the wider communities. It is for this reason that approaches to improving collective relative deprivation should be made in conjunction with policies seeking to improve institutional trust, legitimacy and integration.

Religious fundamentalism

More needs to be done to tackle religious fundamentalist teachings and the spreading of such ideas in communities. The support for such beliefs by a wider community encourages its further development. While policies can continue to encourage involvement and engagement with religious institutions and centres, more needs to be done to prevent the spreading of radical ideologies. The adoption of religious fundamentalist ideologies may be attached to the frequency or amount of time that individuals spend at places of worship, although not necessarily connected to the time they spend there for the specific purposes of routine prayer.

Policies will need to leverage moderate and respected religious leaders' positions and influence in order to combat the spread of the type of fundamentalist ideas that are associated with increased risk for radicalization and recruitment.

Criminal histories

While this review has not identified any strong evidence that radicalization in prisons is a significant factor, individuals with general criminal histories and records of arrest and incarceration are at an elevated risk of radicalization. As such, more should be done at the level of incarceration services to identify those at risk at the pre and post imprisonment stages. This approach would enable assessment of possible in-prison radicalization as well as the ability to effectively monitor high risk individuals post-release. Additionally, those recently released from periods of incarceration, especially related to radical activities that fall outside the purview of terrorism specific laws, are also likely to be at an elevated risk of offending.

This project has received funding from the European Union's Horizon 2020 research and innovation programme under grant agreement N° 699824.

Other issues that intelligence and security services should be especially aware of when attempting to identify those at risk of moving from radicalization of belief to radicalization of action include Differential associations. That is, small groups, organizations and networks of radical individuals increase the likelihood that one or more of them will move to radical action.

Policy makers should be aware of these factors in considering possible alternatives to standard incarceration. Rehabilitative and treatment based approaches may provide alternative avenues to reducing the likelihood that those with criminal backgrounds and incarceration histories will turn to radical actions. The identification of criminality as one of the strongest risk factors for predicting involvement in radical actions, means that policy makers can hopefully lead prevention efforts and diversion programs, especially among young offenders, at an early stage.

This project has received funding from the European Union's Horizon 2020 research and innovation programme under grant agreement N° 699824.

7 Report on ethical and security implications

INTRODUCTION

This report presents the main findings of Task 2.2., conducted with the general aim of *analysing the societal and ethical impact of policies against terrorist networks to make sure that PROTON supports the development of better evidence-based policies.* Countering terrorism is one of the priorities in the EU and its Member States and many efforts have been employed to pursue this aim. As a first reaction to the policies implemented by the EU and the MSs is the controversy about the freedom of human rights, especially data privacy versus national security. This is an important aspect, because it evidences a negative societal and ethical impact of CT policies. In the present research we have explored not only the negative societal and ethical impact of the CT policies implemented in the EU, but we have focused on the possibilities of implementing these policies obtaining a positive societal and ethical impact. Without ignoring the very relevant negative impacts that require urgent actions to preserve the fundamental human rights especially for the Muslim community which is most affected by the negative impact of CT policies, the present report informs about crucial elements, actions that can be taken in the frame of the CT policies, and insights into improving the existing policies to respond to the challenge of terrorism and at the same time promote a positive societal and ethical impact.

As for T.1.2., in order to embrace this endeavour three main activities have been conducted: desk research on scientific literature and grey literature about the *impact of policies against terrorist networks* (hereinafter, CT); desk research for policy mapping on the policies and protocols in the EU member states regarding terrorism, and qualitative fieldwork conducted jointly for T.1.2 and T.2.2. in six EU countries (Germany, Italy, The Netherlands, Romania, Spain, and UK).

The methodology used was the communicative methodology of research (Gómez, Puigvert, Flecha, 2011), which focuses on social transformation and thus distinguishes between analysing the 'transformative' (those elements that contribute to transform a given reality) and 'exclusionary dimension' (those elements that perpetuate a condition of inequality or/and hindrance of transformation) of data. In order to analyse the 'societal and ethical impacts' and set a standard for a definition of what was understood by 'societal impacts' and 'ethical impacts' we have drawn on the EC guidelines 'Better regulation: guidelines and toolbox' for impact analysis of research (European Commission, 2017). The substantial lack of research on the societal and ethical impact assessment of CT policies called for a qualitative research to get insights into the realities of the

This project has received funding from the European Union's Horizon 2020 research and innovation programme under grant agreement N° 699824.

diverse social agents involved in defining policies, in their implementation and the very end-users affected by these policies. Thus, qualitative research was conducted, mainly with in-depth interviews, focus groups and daily life stories providing insight in the very diverse perspective and especially in the common elements involved in preventing recruitment into terrorist networks.

Similar as for the analysis of policies to fight organised crime, counter terrorism policies place greater emphasis on the disruption of terrorist networks rather than on the mechanisms of recruitment. Thus, in the implementation of these policies, oftentimes, potential suspects are targeted with repressive methods. Yet, prevention efforts have to be targeted at society as a whole to be aware of the mechanisms of recruitment and able to resist recruitment or provide people at risk of falling prey to terrorist networks with the necessary support to choose otherwise. The present report provides novel insight to respond to this need.

1. METHODOLOGY AND SCOPE

The methodology used has been the communicative methodology of research, an approach characterised for its dialogic orientation that has been widely recognized by its transformative potential (Mertens & Sordé, 2014). Scientific publications have analysed in-depth the reasons why this methodology is effective in contributing to the scientific, social and political impact of the research' results, outlining the suitability of this method to conduct studies with those social groups most of the time excluded from scientific research such as ethnic minorities (Flecha, 2014; Gómez, 2011; Mertens & Sordé, 2014). These publications have pointed at the own premises on which the communicative methodology stands (for instance, the adoption of a dual conception of reality, which includes *systems* and *lifeworld* [Habermas, 1984]), as well as the methodological strategies that it uses in order to include the voices of the 'researched' subjects as key elements to illuminate new knowledge for informing social change.

Collected data has been analysed according the dimensions defined by the communicative methodology: the transformative and exclusionary dimension. This way of analysing data was what defined the transformative scope of the investigation, as the aim of the study was not only to analyse and describe reality identifying the exclusionary components, but also identifying the transformative effects of those actions implemented in the prevention of both recruitment into Organised Crime (OC) and Terrorist Networks (TN) that have demonstrated to be successful in order to overcome this problem. This said, as *transformative dimension* we considered all those elements that foster positive social and ethical impacts of the policies in place in each of the countries, and as *exclusionary dimension* those elements that hindered the positive social and ethical impact.

This project has received funding from the European Union's Horizon 2020 research and innovation programme under grant agreement N° 699824.

Defining 'ethical impacts' and 'social impacts'

As mentioned in the Introduction, the 'social impacts' and 'ethical impacts' have been defined drawing on the existing *'Better regulation: guide lines and toolbox'* of the European Commission. This regulation sets out the principles that the European Commission follows when preparing new initiatives and proposals and when managing and evaluating existing legislation. The advice of using as a departing point this Toolbox derived from the work of the *Expert Group on Evaluation Methodologies for the Interim and Ex-post evaluations of Horizon 2020*, whose members were in dialogue with the research team of the FP7 IMPACT-EV project, of which Prof Lidia Puigvert is member. The Expert Group and the IMPACT-EV project were commissioned for the analysis of impacts of research projects, whereas the T.1.2. of the PROTON project was oriented to the analysis of the impacts of policies. However, we relied on advice derived from these consortiums for the definition of impacts (to set a methodological criteria) rather than generating these definitions or new understandings of both 'social and ethical impacts' for T.1.2 and T.1.2..

This clarification done, the #Tool 19 of the Better regulation 'Toolbox'[4] was used for the definition of 'ethical impact' and 'social impacts'. For the 'ethical impacts', we included the 'Fundamental rights impacts' of the #Tool 19, which capture the different dimensions of the fundamental rights:

1. General; 2. Dignity; 3. Individuals, private and family life, freedom of conscience and expression; 4. Personal data; 5. Asylum and protection of removal, expulsion or extradition; 6. Property rights and the right to conduct a business; 7. Gender equality, equality treatment and opportunities, non –discrimination, and rights of persons with disabilities; 8. Rights of the child; 9. Good administration / Effective remedy/ Justice.

According to these dimensions of ethical impacts and in line with the communicative methodology, we distinguish between two dimensions for our analysis:

- *Transformative dimension:* Elements that contribute to protect and promote the fundamental rights of a person
- *Exclusionary dimension:* Elements that affect the protection and promotion of the fundamental rights of a person

For the 'social impacts', we included the 'social impacts' identified by the #Tool 19, which are indeed elements linked to advancing the realisation of economic, social and cultural rights:

[4] This Toolbox complements the better regulation guidelines presented in SWD(2017)350. It should be mentioned here that 'Tool 19 #Identification/screening of impacts' of Chapter 3: Identifying Impacts in IAs, Evaluations and Fitness Checks" [SWD(2017)350, corresponds to the "Tool 16 #Identification/screening of impacts' of Chapter 3 of the previous version of this guideline: for the complementation of Better Regulation Guideless presented in SWD(2015).

This project has received funding from the European Union's Horizon 2020 research and innovation programme under grant agreement N° 699824.

1. Employment; 2. Working conditions; 3. Effects on income, distribution, social protection and social inclusion; 4. Governance, participation and good administration; 5. Public health and safety health systems; 6. Crime, terrorism and security; 7. Education & training and education & training systems; 8. Culture; 9. Social impacts in third countries

According to these dimensions of social impacts and in line with the communicative methodology, we distinguish between two dimensions for our analysis:

- *Exclusionary dimension:* Elements that advance the social impacts
- *Transformative dimension:* Elements that hampers the social impacts

Data collection techniques

1) Desk research on scientific and grey literature about the impact of policies that fight against TN

The scientific literature reviewed was conducted considering the following criteria: articles in the database Web of Science and published after 2012 (within the 5-years period 2012-2017). We have used keywords targeting TN and policy impact. Scientific research projects funded by the EU have been also revised.

Using this criterion a total of 25 articles were found, which were classified as (1) useful, (2) partly useful and (3) not useful for the project's goal. Of these 25 articles, 23 were considered useful or partly useful. A great number of the articles we revised about Counter terrorism are focused on preventive measures, and in particular, to the negative impact of these policies which contribute to discrimination, creating the so-called *suspect communities or profiling*.

However, the majority of articles considered as useful analyzed the impact of policies for the case of UK, and many of them focused on the case of Islamic communities. Thus, although the actions tackled by this literature can be also identified in other European contexts, scientific articles are limited to the UK reality, what can be a bias.

Other projects funded by the European Commission, such as the 7th FP Project SECILE have been considered too. This project was useful for the following tasks: better elaborate the legal framework of EU in relation to terrorism; understand the methodologies used by the EU to assess the policies; differentiate which EU countries adopt binding legislations.

This project has received funding from the European Union's Horizon 2020 research and innovation programme under grant agreement N° 699824.

2) Desk research for policy mapping on the policies and protocols in the EU member states regarding TN

We have done a mapping of the policies and protocols in the European Union (EU hereinafter) Member States (MSs hereinafter) regarding CT policies, for the areas of: legal framework (gathering the penal code, the procedural criminal code and other important legislations), strategic plans of each country in relation to terrorism; contrasting if the counter-terrorist policies of the 28 MS follow the EU Strategic Plan.

All our data regarding European policies and measures were of public access through different web searchers such as http://eur-lex.europa.eu/, while the information regarding policies and strategies of the EU MSs were gathered through public sources (the corresponding Ministry in the field of CT for each country).

3) Qualitative fieldwork

Qualitative fieldwork was conducted in six EU countries: Italy, Germany, The Netherlands, Romania, Spain and The United Kingdom. The fieldwork was conducted for OC and TN together (extensive literature and legal documents have already recognised the straight linked among both).[5]

We selected experts, stakeholders and end-users of different domains, which were considered relevant for the analysis of the impact of policies in OC, TN and in both. These domains are *media, prison, migration, religion, education and neighborhoods.*

In order to reach the subjects we followed the coming criteria. First, in the case of experts, we tracked persons with sound evidence of their expertise in the subject. For the majority of cases, these are people who have a long scientific record in the field, publications in top-ranked journals, and as evidence of their knowledge on the topic, they have participated in European and national research projects. Besides, many of these experts are also people who have been involved in the elaboration of policies, participating directly in the process of policy-making (policy-makers) or indirectly being asked to contribute with their academic expertise to assess policy-makers (academics).

[5] This explains why this section “Qualitative fieldwork” is the same one for T.1.2. and for T.2.2.

This project has received funding from the European Union's Horizon 2020 research and innovation programme under grant agreement N° 699824.

A snowball strategy was followed, contacting first experts and stakeholders and later on, end-users. Regarding stakeholders, persons active at the public sphere in relation to OC were reached. For instance, NGOs or associations specifically created with the aim of preventing some type of OC, either public or private organisms which with their task develop a preventive work in OC (e.g.: entities developing education programs located in the most vulnerable neighbourhoods, NGO focused on media literacy, NGOs carrying out prison voluntary work, etc), or working in the field of terrorism and violent extremism prevention.

Finally, end-users were contacted as individuals recommended by the own stakeholders in the six countries or chosen for being recognised through social media networks and among the research team networks for being very active at the grassroots level developing a task in preventing the recruitment in organised crime networks. The majority of end-users selected to participate in the fieldwork are lay individuals that due to their background (belong to ethnic and cultural minorities, are involved in neighbourhood associations, migrants associations, and others) was considered that provide key information on the impact of the policies and programmes in their daily lives.

Fieldwork involved mainly semi-structured interviews, and in a less extent focus groups, which were conducted jointly with relevant stakeholders and end-users, and daily life stories with end-users.

The guidelines [6] used for the interviews are based on the #Tool 19 of the European Commission 'Identification/screening of Impacts'. Nevertheless, every guideline referred to the specificities of the country being studied, and was modified depending on the profile of the expert or stakeholder in order to obtain as much information as possible regarding ethical and societal impacts (for instance, if an interviewee could provide more information regarding OC, the guidelines was adapted to this issue and taking into account the country legislation; the same for the case of counter-terrorism). Moreover, each interviewee was provided with a consent form giving detailed information about the PROTON project and his/her participation in the fieldwork.
The following table summarises the overall techniques done in the qualitative fieldwork:[7]

**December 2017**	
Interviews with experts	34
Interviews with stakeholders (not foreseen in the initial fieldwork)	29
Daily life stories with end-users	8
Focus groups with stakeholders	7
Focus groups with end-users	
**Overall**	**77**

[6] Examples of the guidelines used is annexed. See Annex 2: Country Samples of a general guideline for qualitative fieldwork.
[7] In order to see the entire list of participants in the fieldwork see *Annex 3.Fieldwork study participants*

This project has received funding from the European Union's Horizon 2020 research and innovation programme under grant agreement N° 699824.

ESPANYA	
Interviews with experts	7
Interviews with stakeholders	1
Daily life stories with end-users	6
Focus groups with stakeholders and end-users:	3
**FINAL Nr. OF TECHNIQUES DONE: 17**	

ITALY	
Interviews with experts	5
Interviews with stakeholders	3
Daily life stories with end-users	0
Focus groups with stakeholders and end-users:	0
**FINAL Nr. OF TECHNIQUES DONE: 9**	

UNITED KINGDOM	
Interviews with experts	6
Interviews with stakeholders	5
Daily life stories with end-users	0
Focus groups with stakeholders and end-users:	2
**FINAL Nr. OF TECHNIQUES DONE: 13**	

GERMANY	
Interviews with experts	7
Interviews with stakeholders	5
Daily life stories with end-users	1
Focus groups with stakeholders and end-users:	2
**FINAL Nr. OF TECHNIQUES DONE: 15**	

ROMANIA	
Interviews with experts	3
Interviews with stakeholders	7
Daily life stories with end-users	0
Focus groups with stakeholders and end-users:	0
**FINAL Nr. OF TECHNIQUES DONE: 10**	

NETHERLANDS	
Interviews with experts	6
Interviews with stakeholders	7
Daily life stories with end-users	1
Focus groups with stakeholders and end-users:	0
**FINAL Nr. OF TECHNIQUES DONE: 14**	

Barriers encountered to conduct the fieldwork foreseen

As mentioned, all the profiles initially contacted were looked for according if they were representative of at least one of the six domains of our analysis. This provided in many cases very concrete information for the analysis of the impact of policies in specific domains, ensuring that experts and stakeholders would contribute with new information from new perspectives. However, this categorisation also delayed the fieldwork as when someone (expert, stakeholder), from a specific domain declined to participate we needed to proceed to find a similar profile (for instance, expert in the field of religious affairs with expertise in terrorism and organised crime) to fill the gap.

Another barrier encountered was for conducting focus groups with Stakeholders and end-users. In order to overcome this difficulty and do not delay the entire work, we decided to conduct interviews with stakeholders. Thus, through a different technique we were able to respond to our final aim. Stakeholders provided extremely useful information, sending grey literature of their organisations, reports that they have themselves either collected, participated in their elaboration or which considered that would be of our interest. However, as it is understandable, in many cases they were reluctant to put us in contact with end-users as their concerned that this would involve approaching sensitive issues for them.

Finally, more than 200 people were invited to participate in the fieldwork (some of them accepted, other declined, and some of them did not reply to the petition), what can be also considered for the dissemination of

This project has received funding from the European Union's Horizon 2020 research and innovation programme under grant agreement N° 699824.

the existence of PROTON (the email sent had the most relevant information of the project, as well as its website).

2. FINDINGS

3.1. Literature review

3.1.1. Mechanisms of the European Union to measure the impact of Counter-Terrorism policies

The SECILE project in its report called "How the EU assess the impact, legitimacy and effectiveness of its counterterrorism laws" (Hayes and Jones, 2013) provides a well-defined scheme about the mechanisms that the EU has for the assessment of the impact of the CT policies. However, the research is focused primarily on the utilisation of an application of the mechanisms rather than the substance of the assessment they produce. Moreover, they evaluate only the assessment of binding legislation, hard law, in three different stages: pre-legislative assessments, assessments during the policy-making process and ex-post assessments. Regarding the pre-legislative assessments, public consultation and impact assessment are considered. In the first case, of the 88 binding CT measures analysed by the SECILE project, public consultation was held only on three (retention of telecommunications traffic data; establishment of the Visa information system and the EU framework for critical infrastructure protection). Of these three pre-legislative consultations, only in the one concerned with the VISA information system considered the impact from a human rights perspective (Hayes and Jones, 2013). In the second case, regarding the impact assessment, a tool called *general-purpose impact analysis to be implemented gradually from 2003 and applied to all initiatives undertaken under the Commission's programme work* is considered. However, the conclusion of the SECILE project is that no legislative proposal that originated in the Council has ever been subjected to an impact assessment.

The assessments during the decision-making process take into account the involvement of the European Parliament and also of advisory bodies. In the first case, the SECILE project concludes that after the Lisbon Treaty the majority of primary EU legislation may be subject to co-decision procedures, under which Parliament and Council are obliged to reach an agreement by negotiating new legislation. However, the authors affirm that the European Parliament has been excluded from the EU decision-making process. Regarding the involvement of advisory bodies, the article 300 of the Lisbon Treaty states that European Parliament, the Council and the Commission shall be advised by an Economic and Social Committee and a Committee of the

This project has received funding from the European Union's Horizon 2020 research and innovation programme under grant agreement N° 699824.

regions exercising advisory functions. However, their advice is not binding and it is merely advisory. Some observers, such as Statewatch had hoped that the EU fundamental Rights Agency would also have the option to issue opinions and recommendations. Nevertheless, it has not been the case.

Regarding the ex-post assessment procedures, according to SECILE, once the legislation has been adopted, it must be implemented by the member states, EU bodies or private sector. Depending on the legislative instrument in question, the implementation or transposition of the legislation may be subject to a review. However, Hayes and Jones (2013) affirm that *the assessment measures if the member states have implemented the legislation rather than how effective that legislation has been in respect to its aim*. The authors also stress that *of the 88 legally binding CT measures identified in their research, 59 contain provision for review by the European Commission and nine of these provide for further review by the Council. In conclusion, one third of the EU legally binding CT measures contain no provision for review, suggesting little or no concern for their impact*.

Finally, the EU also has substantive reviews of the CT policy. First, the peer review mechanism. The Council wanted to receive an evaluation report by the end of 2002, but the report was not issued until 2005 with the aim to provide analysis of member states' structures and capabilities, highlight good practices and give recommendations. According to Hayes and Jones (2013) *Council's final report does not raise any questions about whether such changes may have an impact on fundamental rights or democratic control*. Another, peer evaluation was completed for the period 2007- 2010 regarding preparedness and consequences management in the event of a terrorist attack.

A different substantive review mechanism is the network of independent experts on fundamental rights created by the European Commission in response to a recommendation in the European Parliament's report on the state of fundamental rights in the European Union in 2000. According to SECILE Project:

> *The network was established in 2002 and produced four Annual reports covering the period 2002-2005, four thematic comments including one on the Balance between Security and Freedom in the European Union and 15 opinions including reports on the Requirements of fundamental rights in the framework of the measures of prevention of violent radicalisation and recruitment of potential terrorists, the human rights responsibilities of the EU Member States and ethnic profiling*.

While these reports were extremely detailed of the way specific EU measures impacted on the fundamental rights of citizens. There was no meaningful consideration of their reports by either the Council of the EU or

This project has received funding from the European Union's Horizon 2020 research and innovation programme under grant agreement N° 699824.

the European Commission, and neither any attempt to act upon their findings. Instead, in 2006 the network was disbanded to make way for the Fundamental Rights Agency, which was denied any competence at all in regard to EU Justice and Home Affairs policy.

The European Council in 2004 established the EU Counter Terrorism Coordinator (CTC) with the aim of *monitoring the implementation of the EU counter-terrorism strategy (...) fostering better communication between the EU and third countries (...) ensuring that the EU plays an active role in the fight against terrorism*.[8] The CTC issued regular reports, but without mentioning aspects of impact and legitimacy of these policies.

Finally, the European Commission published a Communication called "The EU CT policy: main achievements and future challenges". However, this report is mainly descriptive and essentially listing the measures introduced and the areas where it needed to improve. In response to this document, the European Parliament in a report of 2011 stressed, "little has been done to assess (…) whether the measures (…) have been evidence-based (and not based) on assumptions".

The Statewatch report for the SECILE project concluded that:

> *There are ample mechanisms and there is certainly no shortage of expertise available to the EU to properly assess the impact, legitimacy and effectiveness of its counter-terrorism policies. But our research suggests that these resources are at best underutilised and at worst applied in a manner that ultimately ignores crucial issues of civil liberties and human rights, necessity and proportionality, accountability and democratic control. Such an approach is fundamentally at odds with the values espoused by the EU Treaties.*

3.1.2. Conclusions of other reports measuring the impact of Counter Terrorism policies

a. Fundamental Rights Agency. Embedding fundamental rights in the security Agenda (2015).

As it has been affirmed, the EU has enough mechanisms and resources to measure the impact of its policies in counter terrorism, but these are barely used or taken into account. However, other agencies such as the European Union Agency for fundamental rights has been elaborating reports such as the report called

[8] For more information see: http://www.consilium.europa.eu/en/policies/fight-against-terrorism/counter-terrorism-coordinator/

This project has received funding from the European Union's Horizon 2020 research and innovation programme under grant agreement N° 699824.

embedding fundamental rights in the security Agenda (2015). The FRA affirms that the policy response at the national level has tended toward the adoption of tighter security measures to counter terrorism and radicalisation. It is clear that the EU Member States have the duty to protect their population and the EU is uniquely placed to assist them. Moreover, the FRA also emphasises the fact that the security measures and policies should reduce the risk of alienating entire communities by measures that could be perceived as discriminatory. The internal security strategy should pursue the respect of Fundamental rights in planning and implementing internal security policies and actions. For this reason, the agency calls to the implementation of more sustainable and effective policies capable of passing the necessity and proportionality tests. Some of the measures, which are stressed by the FRA as controversial are the EU Passenger Name Record (PNR) as disrespectful of the fundamental rights because it is linked to profiling and data protection violations. This measure can lead to discrimination and false positive matches. The FRA published a complete report about this particular measure in 2011[9], and a set of fundamental rights safeguards in 2014[10], which introduce clear and strict limitations on purpose, personal data safeguards or increased transparency of the system towards passengers. Another measure mentioned in the report is the confiscation of travel documents, which is possible as long as is necessary to protect national security. However, it cannot target people based on criteria such as religion or ethnicity.

The same occurs with the systematic checks of EU nationals at the external borders of the Schengen area in order to intercept potential foreign fighters travelling to Middle East. According to the FRA, this measure should be predefined and regularly reviewed assuring that the selective checks are based on evidence and did not constitute discriminatory profiling.

The FRA also states that profiling is a legitimate preventive instrument. However, surveillance of a specific group or profiling of potential suspects based solely or mainly on ethnicity or religion creates the risk of unacceptable discriminatory treatment. The FRA also affirms that a high number of the measures proposed at EU level, and others in the Member States may have an adverse effect on the rights of individuals, giving rise to a sense of discrimination and potentially contributing to the alienation of some groups and in consequence, undermining their trust in the authorities. In consequence, the FRA published a guide with the aim of assisting law enforcement authorities to identify legitimate and illegitimate uses of profiling in 2010[11]. An important FRA remark is that the prevention of radicalisation may support social inclusion and participation because a

[9] See FRA (2011), Opinion on the proposal for a Directive on the use of Passenger Name Record (PNR) data for the prevention, detection, investigation and prosecution of terrorist offences and serious crime, FRA Opinion 01/2011, Vienna, https://fra.europa.eu/en/opinion/2011/

[10] FRA (2014), twelve operational fundamental rights considerations for law enforcement when processing Passenger Name Record (PNR) data, http://fra.europa.eu/en/news/2014/ fra-provides-guidance-member-states-setting-national-pnr-systems.

[11] FRA (2010), Towards more effective policing, understanding and preventing discriminatory ethnic profiling: A guide, http://fra.europa.eu/en/publication/2012/ towards-more-effective-policing-understanding-and-preventing-discriminatory-ethnic.

This project has received funding from the European Union's Horizon 2020 research and innovation programme under grant agreement N° 699824.

previous FRA research shows that a sense of social marginalisation is closely linked to the experience of discrimination[12]. The EU Minorities and discrimination survey showed that 40% of Muslim respondents who had been previously stopped by the police believed that this was specifically due to their minority or immigrant status[13]. The FRA remarks the fact that poorly designed security policies that are perceived as targeting an entire community rather than individual suspects can exacerbate the problem. In order to avoid this problem, the FRA in 2013 published a fundamental rights based police training manual to provide guidance on how law enforcement can carry out its duties in a fundamental rights consistent manners[14].

The FRA appoints the Radicalisation Awareness Network (RAN) as a useful platform to share the best practices on combating radicalisation, overall concerning radicalisation in prisons and probation. This network provides the dissemination of suitable exit strategies such as alternative means of civic engagement.

The FRA also stresses the need to promote tolerance and non-discrimination, affirming that people of Muslim faith are not portrayed as a monolithic group conducting to radicalisation. It is important to reiterate that radicalisation in Europe is not limited to specific ethnic or religious groups. For this reason, it is important to dialogue with Muslim communities and ensure their representation across different domains. As well as, the EU and its Member States should avoid legitimising xenophobic reactions and hate crimes.

b. International Commission of Jurists. Urging Action: Report of the eminent jurist panel on terrorism, counter-terrorism and human rights (2009)

The International Commission of Jurists has a report called Assessing Damage, Urging Action: Report of the eminent jurist panel on terrorism, counter-terrorism and human rights. This report is mainly legal discussing ICJ affirms “many States have fallen into a trap set by the terrorists. Ignoring lessons from the past, they have allowed themselves to be rushed into hasty responses, introducing an array of measures, which undermine the values and the international legal framework developed since the Second World War”. This report is mainly legal, making reference to the dilemma between security and human rights, and analysing the effect of this measures on human rights. However, there is a part dedicated to the social impact of CT measures based in the evidence of the past, affirming *CT in the past were said to be counter-productive*. The ICJ states that *the disappointing aspect of this knowledge is that present governments are not using it*.

---

[12] See FRA (2010), Experience of discrimination, social marginalization and violence: A comparative study of Muslim and non-Muslim youth in three EU Member States, Luxembourg, Publications Office, http://fra.europa.eu/en/publication/2012/ experience-discrimination-social-marginalisation-and-violence-comparative-study.

[13] FRA (2009), EU-MIDIS Data in Focus Report 2: Muslims, Luxembourg, Publications Office, http://fra.europa.eu/en/publication/2010/ eu-midis-data-focus-report-2-muslims.

[14] FRA (2013), Fundamental rights-based police training – A manual for police trainers, Luxembourg, Publications Office, http://fra.europa.eu/en/publication/ 2013/fundamental-rights-based-police-training-manual-police-trainers.

This project has received funding from the European Union’s Horizon 2020 research and innovation programme under grant agreement N° 699824.

Experience suggest that neither CT law nor operations, nor indeed military alone, can prevent or resolve the problems created by terrorism. Comprehensive solutions, including political, social and economic approaches are necessary. Hearings said that the notions of terrorist and terrorism obfuscated, rather than helped. The use of such language tended to hinder rather than facilitate the search for solutions. Therefore, as a short-term solution, this may seem attractive; over the longer term, experience showed that such labelling makes dialogue, negotiation and resolution of conflict extremely difficult.

In this part, it is mentioned suspect communities in particular, affirming "past experiences suggest that States target particular communities with counter terrorism laws and other measures. The discriminatory targeting or marginalising of certain groups is both legally wrong and has often proved counter-productive because it stigmatize whole groups and even impede the ability of the authorities to gather crucial intelligence information".

The ICJ report also includes provisions on how to avoid or limit the counter-productive effects of CT measures with examples from the past such as the hearings in Northern Ireland where the witnesses averred that if some or some safeguards had existed, a lot of violations might be avoided. For instance, codes of conduct, stronger equality legislation, creation of domestic bodies to oversee and guide government on human rights and equality measures, integration of human rights and equality considerations into all government policies, strong civic oversight of the police and a range of measures to ensure recruitment from across different communities, (…).

c. **Project TERRA (Terrorism and Radicalisation). European Network based prevention and learning program. Inventory of the best practices on de-radicalisation from different Member States of the EU.**

The project TERRA is a European project supported by the European Commission DG Home Affairs with the aim of reinforcing the positive role victims and former terrorists can play in relation to the prevention of radicalisation and providing practical guidance to specific target groups. Target groups and beneficiaries include victims, (potential) terrorists, EU member states and frontline-workers in the field of law-enforcement, rehabilitation, teaching, welfare and social workers, journalists, policy makers, and religious leaders. TERRA stimulates knowledge synthesis and exchange throughout the European Union, between groups and between member states. The project builds on the work of the Network of Victims of Terrorism (NAVT) and is complementary to and provides input for the Radicalization Awareness Network (RAN) as a 'network of networks'.

This project has received funding from the European Union's Horizon 2020 research and innovation programme under grant agreement N° 699824.

Moreover, they have a document called Inventory of the best practices on de-radicalization from the different Member States of the EU with the aim to gather the best practices on de-radicalization from different European Member States.

d. **Organization for Security and Co-operation in Europe (OSCE). Preventing Terrorism and Countering Violent Extremism and Radicalization that lead to Terrorism: a community-policing approach (2014).**

This report is focused on the community-policing approach to prevent terrorism and counter Violent Extremism and Radicalization that lead to Terrorism (VERLT, hereinafter). According to OSCE (2014) many counterterrorism policies and practices have been "community-targeted". These practices have focused on tactics such as the use of police stop-and-search powers, covert operations and intelligence-gathering methods to detect suspected terrorists and thwart their activities, especially active plans for attacks. However, these tactics usually are a necessary part of any effective strategy for countering terrorism, but these are carried out without a particular and well-defined framework based upon the rule of law and respect for human rights. The OSCE report (2014) also affirms that there is a difference between community-targeted approach and community-oriented approach. In the last one, the methods are used with strong partnership between the affected community and the police, with a high degree of mutual trust on both sides and based on transparent information on the goals and scope of such practices. OSCE concludes that "the state can diminish the risk of stigmatizing particular communities by engaging with a broader number of people and/or engaging them on a diverse range of issues not limited to empowerment against terrorism".

OSCE (2014) confirms that the Community Policing approach cannot be an isolated strategy.
In conclusion, this document revises the community-policing approach to tackle terrorism and radicalization. It includes a section to evaluate the impact of this method to increase effectiveness. Nevertheless, the report provides examples of possible indicators to evaluate progress in achieving benefits of community policing in preventing terrorism and countering VERLT, but no actual results. However, the document gives important insights about the community policing approach, with examples of previous experiences. The main conclusion is that "community policing may incidentally benefit counterterrorism depending on the level of trust and co-operation that already exists between the police and the public".

e. **International Commission of Jurists. Press release: European Union Directive on Counterterrorism is Seriously Flawed. November 2016.**

This project has received funding from the European Union's Horizon 2020 research and innovation programme under grant agreement N° 699824.

The international Commission of Jurist together with a group of human rights organisations released a press statement on November 2016 affirming that:

> *The overly broad language of the new EU Directive on Combating Terrorism could lead to criminalising public protests and other peaceful acts, to the suppression of the exercise of freedom of expression protected under international law, including expression of dissenting political views and to other unjustified limitations on human rights. The directive's punitive measures also pose the risk of being disproportionately applied and implemented in a manner that discriminates against specific ethnic and religious communities.*

Therefore, the organisations called the Member States to transpose this Directive in a respectful way to ensure human rights and liberties according to international human rights obligations,. Moreover, these organisations also claim the fact that the legislative process for adopting the directive was not transparent, and that there was no impact assessment of the proposal.

This press release is one of the multiple statements made by civil society and other human rights organisation to stress the need to further enquiries about the creation and implementation of Counter-terrorism legislation. It is obvious the need to consider civil society movements in the creation of legislation.

f. Council of Europe. Evaluation of National Anti-Terrorist arrangements.

Every Member State of the EU has a report of the Council of Europe evaluating the counter- terrorism arrangements. However, some of the information of these reports is classified and not publicly accessible. Moreover, the information is mainly descriptive, without considering the legitimacy or impact of the policies.

3.1.3. Scientific literature review on the impact of Counter Terrorism policies

a. Preventive measures and negative impact

The vast majority of literature about the impact of Counter Terrorism policies is focused on the negative effect that preventive measures or community policing have, alienating communities and ethnic minorities. In the analysis done by Sentas (2015), the author concluded that *the counter-terrorism policy of 'disruption' is the primary mechanism of criminalization in this study, reliant on both direct and indirect forms of MI5*

This project has received funding from the European Union's Horizon 2020 research and innovation programme under grant agreement N° 699824.

intimidation and to a lesser extent the Metropolitan Police. In other words, the author affirms that repeated visits within a community, over years and of multiple people within a confined period of time, function as collective harassment of the Kurdish people. In this study, the harassment of Kurds relies on regenerating experiences of persecution in Turkey by use of deportation threats. Disruption also relies on communicating to those targeted that they are subject to extensive surveillance, using knowledge of targeted people's former Kurdish names in order to heighten fear.

Hickman, et al. (2011) arrive to the same conclusion explaining that *the recent linking of extremist ideas that are non-violent with violent extremism, on the one hand reinforces the notion of a 'suspect' community and on the other hand makes explicit what has always been implicit in the idea of 'the enemy within' and the injunction that dangerous people are lurking 'in our midst'.* It is another argument in line with the negative and indirect impact that some measures have on some communities.

Alam and Husband's (2013) research is affirming that the introduction of counter-terrorism policy in the form of "Prevent" was met with a wide range of strong resistance. Many councillors responsible for the implementation of this policy saw it as discriminatory in its unambiguous targeting of Muslim communities. At the same time, Muslims saw the measures related to "Prevent" as an assault on their integrity as law-abiding citizens.

Guru (2012) also arrived to similar conclusions about prevention and intervention measures with special focus on the effect that it has on their families. Guru (2012) affirms that the political effects of 9/11 and 7/7 have unfolded in ways that have tyrannised Muslim communities through counter-terrorism legislation. Amidst the widespread concerns about Islamophobia, there remains a hidden population of women and children who suffer the repercussions of the punishment meted out to their men-folk. This has received little attention, particularly in social work research and literature. However, the experiences of the women and the children testify to their isolation, police brutality, undignified treatment, financial hardship, and emotional and psychological difficulties. Whilst more extensive and rigorous research in this area is necessary, these examples highlight how state policies manipulate the intersections of gender, 'race', religious and class divisions and the ways in which these processes are continuously renewed.
Fischbacher-Smith (2016) stresses a new perspective in the "Prevent" section of the CT measures. He states that the current policy puts the responsibilities for identifying early-stage radicalization on organizations for whom it is not their primary concern (and for which they are ill-equipped), and there is an insufficient research evidence base available to them to show how effective the various forms of intervention might be. Recent concerns expressed by those who will now be charged with implementing PREVENT have pointed to this lack of an evidence base and the challenges associated with addressing extremist views, especially without

This project has received funding from the European Union's Horizon 2020 research and innovation programme under grant agreement N° 699824.

appropriate training for the task. In conclusion, what Fischbacher-Smith (2016) is affirming is that the prevention strategy is not working because the institutions/staff who has to deal with that is not well prepared. Thus it has a negative operational impact.

Ahmed (2015) stresses that the discussions around the lack of human rights in counter-terrorism legislation were found to exacerbate feelings of fear, insecurity, vulnerability and helplessness. The ‘war on terror’ is perceived to have introduced mechanisms of control which have made risk a much more dominant feature of the criminal justice system, undermining the values of society and indeed the state’s commitment against repression (McCulloch and Pickering, 2009 in Ahmed, 2015). Therefore, these feelings transcended to perceptions of the police with participants believing that counter-terrorism policing incorporates religious profiling and therefore the placement of British Muslims within the constructed binary of ‘criminal’ as opposed to ‘victim’. Within this context, participants framed themselves as vulnerable ‘victims’ and this was due to their perceptions of injustice, vulnerability and powerlessness. The introduction of pre-crime counter-terrorism legislation marks the intrusion of the construction of the enemy with anger, disgust, fear and risk (McCulloch and Pickering, 2009 in Ahmed, 2015).

Lennon (2013) makes an important contribution in this perspective about the stop and search measure. He affirms that adopting precautionary measures in the context of street policing, and criminal law more generally, creates a number of inter-related problems. Designed to operate in conditions of uncertainty, they must be capable of deployment when there is no reasonable suspicion. This requires vague drafting in the governing statute and the granting of substantial discretion to the police. As well as being demanded by the logic of precaution, the grant of broad discretion flows directly from the imprecise statutory drafting. These high levels of police discretion increase the risk of the arbitrary exercise of power and may ‘lead to intensified politicization of policing and law’ (McCulloch and Pickering, 2009 in Lennon, 2013). If the police act, or are perceived to act, in a procedurally unfair manner this weakens police legitimacy and reduces the likelihood of people assisting the police. Given that intelligence is the life-blood of counter-terrorism, this may serve to undermine the overall effectiveness of the counter-terrorist measure. Broad discretion coupled with vague statutory drafting undermines core aspects of the rule of law, making it difficult to satisfy the requirement that a measure, which infringes a human right be prescribed by law.

Isakjee and Allen (2013) also make an important contribution with the evaluation of a project in Birmingham called Project Champion. They affirm that for some Muslims interviewed for this project, Project Champion was part of a singular political and military project that was targeting and oppressing Muslims as much in Birmingham as indeed in Afghanistan and Iraq.

This project has received funding from the European Union's Horizon 2020 research and innovation programme under grant agreement N° 699824.

Jarvis and Lister (2013) also contribute to this effect stating that citizens of distinct ethnic and geographical demographics perceive a diminishment of citizenship that stems from anti-terrorism measures.

Eijkman, Lettinga, & Verbossen, (2012) conducted research in the Netherlands where an evaluation study into the effectiveness of preventive stop-and-search operations was conducted in ten municipalities including Rotterdam and Amsterdam. The study also considered the impact on public opinion. Only four percent of respondents were negative about the instrument and half said that their sense of security had improved. However, the outcome differs for ethnic minorities; almost one in four respondents of minority descent in Rotterdam disagreed or strongly disagreed with the statement that preventive searches increased public safety. Minorities also felt that they were subjected to such searches more frequently than the general population.

Finally, Bossong (2014) also stresses that the EU's strategy to combat radicalisation and recruitment into terrorism has also underlined the limits to further integration. EU-sponsored research has highlighted the diversity and complexity of the terrorist challenge, but concrete policies and pilot projects have largely accentuated repression policies over early prevention.
The SECILE Project does not have specific conclusions about “Prevention” measures like the ones analysed above because SECILE is focused on different measures such as the European Arrest Warrant, Counter Terrorism finance and border control. However, one of the contributions of SECILE is that CT measures have indirect impacts. As stated by Kalliopi Koufa (a UN Special Rapporteur on Terrorism and Human Rights) this situation can stimulate an atmosphere of weakened resistance to very harsh anti-terrorism measures and generalised racism and religious intolerance with broad politico-legal consequences. The construction of a hierarchy of rights within a security context means that tolerance and acceptance of diversity can become overlooked as values, leaving minority groups in precarious position. Moreover, in the SECILE document D4.4 one of the participants expressed concerns in relation to the impact of the measure thus related primarily to the risk of discrimination through profiling and the targeting of NGOs, which were both considered negative impacts (P.19).

b. Data transfer and data retention

Maras (2012) affirms the Data Retention Directive will result in considerable monetary costs; despite that European Community institutions claim they had taken appropriate steps to ensure that the costs of this measure would be limited. Regardless of whether or not service providers are reimbursed by Member States for implementing the Directive, citizens may end up paying to have all of their communications data stored for a period of up to 2 years (with the possibility of extension). The Directive may also negatively impact

This project has received funding from the European Union's Horizon 2020 research and innovation programme under grant agreement N° 699824.

competition and other economic policies in the European Union. The adverse economic effects of data retention might lead consumers to use international webmail services (that is, non- EU providers) and new (and even existing) market participants to take their businesses elsewhere. In short, the economic advantages of pursuing EU-wide data retention were found to be more than outweighed by its economic disadvantages. The Data Retention Directive is, therefore, a disproportionate measure.

SECILE project (D2.4) also contributes to this measure stating that the implementation of the Directive has had a tremendous impact on fundamental rights and other constitutional protections. By establishing the principle that data must be retained for long periods in case police and security agencies need to access it later, the EU has crossed the Rubicon into mass surveillance. Moreover, the Directive has failed to harmonise data retention regimes for law enforcement purposes because of the wide margin of discretion given to member states when transposing its provisions. By giving Member States the scope to choose their own retention periods and to decide which authorities should have access under what type of authorisation, and by failing to include a list of crimes for which retained data can be retrieved or specific data protection provisions, the legislative situation is arguably now only slightly less uneven than before the Directive was adopted.

The SECILE project (D3.5) also contributes theoretically providing an analysis of the dilemma security vs. liberty or surveillance vs. privacy. The authors affirm that privacy has also come under duress as a result of counter-terrorist (discursive and non-discursive) practices. Privacy has since increasingly been disregarded as a luxury that must be dealt away when political times become stringent, as a hurdle to the work of the police, and as that which allows terrorist groups to thrive, away from prying eyes. Consequently, privacy has continuously been eroded in the face of new surveillance mechanisms, as the recent PRISM affair amply demonstrates. The information thus collected is then mined by the means of probabilistic association rules, which are supposed to allow for the thwarting of unknown terrorists and disrupting of their plots. These routine practices of massive electronic surveillance enable illiberal practices such as blacklisting and financial-assets freezing. Although they yield drastic punitive effects affecting the lives of the targeted individuals, these measures are extra-legal. They may therefore easily slip through the protections afforded by the regular legal order of a liberal and democratic polity.

c. Counter terrorism finance law and policy

Guild (2008) affirms that targeted' financial sanctions have been relatively ineffective. They have often been imposed on people and entities selected on non-transparent or dubious grounds. The Government should look for ways to incorporate a proper degree of transparency and due process into targeting procedure (House of

This project has received funding from the European Union's Horizon 2020 research and innovation programme under grant agreement N° 699824.

Lords, 2007 in Guild, 2008). The SECILE Project D4.2 (2013) based on the perspectives garnered in a focus group with eleven counter-terrorist finance operatives from nine member states affirms the difficulty in determining the impact of counter-terrorist finance law and policy given the absence of a means to measure the risk of terrorist financing and the complexity of the system. Participants felt counter-terrorism measures in this area were more likely to serve a disruptive function rather than ending terrorism per se. Some participants noted the role of asset-freezing sanctions and the challenges of effectiveness, particularly in relation to a lack of political agreement as to which organisations should be blacklisted and the use of litigation to oppose such decisions. Participants disagreed on the question of whether measures should aim for prosecution or prevention.

2.2. Results from the Fieldwork: ethical and societal impact

The results from the fieldwork conducted to identify the ethical and social impact of CT policies provide insight into those aspects that can help us to improve policies and their implementation in order to lead to a positive ethical and social impact improving the life of EU citizens, and especially minority groups in our societies. In line with the communicative methodology, the results distinguish between two dimensions: the exclusionary and the transformative dimensions, highlighting those elements that either hinder or promote the positive achievement of ethical and social impact of counter terrorism policies. These two dimensions gather a variety of aspects that have been pointed at by previous research, especially highlighting the negative impact that CT policies have. The results presented here shed light on diverse aspects that need to be taken into account when implementing CT policies to prevent the negative impact, while at the same time providing insight into the implementation of CT policies that have achieved a favourable impact on society and especially on vulnerable groups.

2.2.1. Exclusionary dimension: elements that hinder the positive achievement of social impact and protection of fundamental rights of counter terrorism policies

In line with the results obtained in previous research on the impact of CT policies highlighting the negative impact especially on vulnerable groups, the Muslim community when it comes to discussing terrorism in Europe (Ahmed, 2015; Alam and Husband, 2013; Guru, 2012; Hickman et al., 2011), we have found further evidence of the widespread dimension of these negative impacts that are all related to a stigmatisation and criminalisation of the Muslim community. Thus there are effects of police interventions or the direct

This project has received funding from the European Union's Horizon 2020 research and innovation programme under grant agreement N° 699824.

implementation of policies such as deportation and extradition, the second order harassment that affects family and other members of the Muslim community, as well as the perpetuation of the stigmatisation of the Muslim community in media as well as in education. This is especially striking considering that several interviewees – experts, stakeholders and end-users – highlight that the real problem in extremism is not religiously based terror, but rather the increasing far right extremism. In the following we will delve into the diverse dimensions of the effects that existing policies in the EU Member States analysed here have on the Muslim community as a suspect community underlying the implementation of these policies in ethnocentric societies.

1. **Criminalisation and/or stigmatisation of the Muslim community vs. national security during intervention**

As observed in previous research police interventions with measures such as the stop and search methods to identify potential terrorists in diverse settings lead to greater stigmatisation if these mechanisms are especially put in practice in particular neighbourhoods or with a specific ethnic or religious community. We have found evidence for that in the UK especially with the PREVENT Strategy, in Germany and in the Netherlands. In this regard, some interviewees have listed the fact that young adults pertaining to the Muslim community are more likely to be stopped and searched by the police than the average population. This is explained by the fact that preventive measures in the legal sense are mainly having the opposite effect: Muslim communities feel attacked, because of the prevalence of stop and search actions in particular neighborhoods.

For instance an expert in the field expresses how Dutch legislation and policies, which are mainly centered on prevention rather than on disruption or repression, are affecting the Muslim community:

> "What we see in general, (the same for ct and oc) is an increase of preventive stop and search powers for law-enforcement officials. And in the Netherlands we see that these preventive searches very often do not require a reasonable suspicion of a crime being committed. So it is a pro-active stop and search powers: there is no requirement for a reasonable suspicion, so it is a very broad discretionary power… for instance to ask identification requirement" (Expert, The Netherlands)

> "Many of the Dutch counter-terrorism laws that have been implemented since 2001, have been criticized extremely by Dutch Legal Scholars due to their difficult position in the light of human-rights. Especially one particular law that dealt with investigative powers and police power in general: seen as highly problematic as it would give a lot of power to the prosecutor, to police officers to stop and search people in the street, to keep people detained for very long periods of time without having

This project has received funding from the European Union's Horizon 2020 research and innovation programme under grant agreement N° 699824.

to disclose their files to their lawyers…. Problematic in the light of the process and human rights in general… There have been debates among legal scholars and there have been demands being placed to our government for an evaluation of these CT laws and policies, especially in the light of their difficult position towards human rights and towards the impact that they would have predominantly on certain minority groups in the countries. Politicians showed concern that predominantly Muslim community would fall victim (...)" (Expert, The Netherlands]

These concerns raised by politicians and experts in the Netherlands are confirmed by end-users. To provide only two examples, here the comments of a female Muslim end-user in Spain as well as a female Muslim end-user in Germany:

"Yes a feeling of criminalization exists… Especially among young people… They are constantly stopped without being given any particular explanation" (End-user, end-user, Spain]

"For example a friend of mine was at the airport, because she wanted to travel somewhere, I don´t remember where she was going. So she was asked to step aside and take off her veil, to see whether she was hiding something there. I mean, then you would also have to ask women who do not wear a veil to step aside and have their bellies searched or something. I think this is a pity, there is no equal treatment." (End-user, Germany).

Several examples were also explained in the focus group in UK were the PREVENT strategy and the actions derived from its implementation were discussed. A Muslim female participant explained that this strategy has generated fear and distrust among the Muslim community, as instead of being a measure of safeguarding, has spread a discourse which associations Islam and Muslims to terrorism, thus criminalising not only to adults Muslims but also to children. Participants also emphasised that if this Strategy had been elaborated and implemented really counting with the community, such environment would not have been created.

Another example of police intervention that contributes to the sense of a limitation of the right of conscience is the forceful intrusion into Mosques, following in some specific cases the introduction of secret agents in the Mosques or even censorship of opening Mosques at all. One stakeholder in Spain explains that secret agents were being introduced in the Mosques and the consequences this had on the Muslim community as impacting the freedom of conscience, expression and religion:

"Measures are necessary for the protection of society in general. Protocols work well on paper, but they do not work well when applied to the whole population. It can be understood differently

This project has received funding from the European Union's Horizon 2020 research and innovation programme under grant agreement N° 699824.

depending on how they are implemented. In the police bodies, laws are applied negatively. For instance, in 2009 there was an Imam who had no regular documents. He was arrested on a casual Friday when the Mosque was full (600-700 people), with weapons, with shoes, etc. This is an erroneous application of the protocols, without taking into account sensitive issues; so protocols can be applied in different ways. Islam is associated with terrorism, in the media, and this fact causes huge damage. Protection is always treated from the national security perspective. More integral and integration solutions are needed if we live in a multicultural society; we cannot continue talking about social integration, living in coexistence and mutual respect. All measures are only on security, including schools' protocols. More social policies should be implemented. The school curricula should deeper consider diversity." (End-user, Spain)

In a similar vein, an expert from the UK mentions the deeply entrenched structures that are involved in contributing to negative outcomes of the implementation of CT policies in policing that are affecting ethnic minorities. He thus highlights the persistent racism present in our society and the resulting misuse of the mechanisms put in place to effectively implement CT policies. Also, he points to another recurrent issue in the interviews regarding some counterterrorism policies across countries, such as the PREVENT Strategy in the UK, the 'Netherlands Comprehensive action Programme to combat Jihadism' in the Netherlands, or other laws in Germany, which is an increased perception of surveillance of the Muslim community or ethnic minorities which goes beyond the institutions of law enforcement.

"Ethnic minorities are most impacted by government policies around CT but also at the same time it is argued that there is the problem of policing, and the assumption of criminalization (…) On the other hand you have the idea that the police is unable or unwilling to carry out their function because they are confirmed that they are perceived to be racist.... So you have the existence of racism within the police plus the idea that the police can't do their job properly ... it is a kind of paradox here. And the main result of this is that policing around ethnic minorities does not help ethnic minorities to be more secure, less vulnerable and less isolated... This is a part of a larger democratic deficit in how ethnic minorities are integrated into citizenship (…) I think that here the issue is more clear around the issue of counter extremism when the elaboration of the strategy has been in the hand of, not professionals ... but of ideologues in many cases… The governments many times have an idea of what they call "radicalisation" for example.... So what they do is find evidence for that. First they have the idea and then they look for the evidence, often relying on very dubious or very weak research, which is not open to public scrutiny. (…) But you have also some companies, for instance Thomson Reuters which are producing a list, a software which tells you of whether any speaker if ever been in any problem of extremism... So you have a kind of White list, an approved speakers

This project has received funding from the European Union's Horizon 2020 research and innovation programme under grant agreement N° 699824.

> because if you are a bureaucrat, and you see that your college, your university or your Department is an invited speaker who is not on this list you may feel that is maybe too dangerous for you (…) So you can see the impact that this can have on freedom of expression and freedom of speech, but also on intellectual activity" (Expert, UK)

Further concern mentioned by the interviewees is the fact that people suspected of relations to terrorist networks are more likely to encounter problems to obtain a passport or make progress in their immigration status procedures. This is due to the implementation of certain security laws. As already mentioned by the interviewees the implementation of these laws in some countries relies heavily on the person in charge of law enforcement. Thus, we cannot guarantee some fundamental human rights if there is a risk of the protection of personal data and of the right to privacy. The consequences of this measure affects a great variety of social spheres in that person's life. An expert from Germany explains some of these implications:

> "The consequences if you have your ID removed are huge… You can't rent a car, access to accommodation, even look for a job, so there huge stigmatisation. However, this is the debate about what should be prioritised, if national security or the fundamental rights" (Expert, Germany)

Moreover in some countries, such as the Netherlands or Italy, CT policies contemplate extradition related to the immigration status and put people at an especially vulnerable position as they are at risk of suffering torture or ill treatment in their countries of origin. One of the experts from Italy notices in this regards that since the Decreto-legge 18 febbraio 2015, n. 7, which contains urgent measures for counter terrorism, an increase of deportation of migrants especially to Morocco and Tunisia has been perceived though not analysed as such. However, deportation of migrants to these countries due to radicalisation implies putting them at risk for torture.

2. Second order of harassment of the families

In relation to the impact of the policies on the families of potential suspects and or proven members of terrorist networks, Guru (2012) already highlighted myriad facets of harassment directed at these people, including isolation, police brutality, undignified treatment and further consequences such as on the family economy, emotional and psychological wellbeing. This sort of harassment against family members and children is a second order of harassment, punishing not only the suspect or convict, but the people around him regardless of their involvement in the radicalisation or not. This kind of second order harassment is crucial to keep in mind because it heavily affects the fundamental human rights oftentimes of innocent people, who see their

This project has received funding from the European Union's Horizon 2020 research and innovation programme under grant agreement N° 699824.

lives fall apart not only due to the potential or real involvement of a beloved person in a terrorist network but also because of police intervention against them. The examples evidence that most of the previous negative aspects of police intervention are not only applicable to the suspect or terrorist him or herself but also to their families. Examples of this second order of harassment against families can be found in several of the interviews and focus groups conducted. Here we will provide only two quotations to state the widespread effect it can have on the families lives, ranging from a compromise of data protection, lack of social services or support systems leading for instance to younger siblings follow their older siblings path, harassment or termination of their contracts in their workplaces, to severe implications on health. The first quotation comes from a mother who as lost her son in Syria after he joined Daesh, she is part of the Mothers for Life international association founded to provide some sort of support to families in the same situation due to the lack of institutional assistance.[15]

> "Often families are targeted and treated as criminals as well. Because it is a very delicate situation, they are scrutinized by the authorities, the laws are still not consistent all the way through, policies are vague, there is nothing indicating how anybody is getting any support or assistance. It's more about a top level, about jail sentences, and even then it is unclear how the trial is to proceed. It depends very much on the case, and the lawyer, the department, the area. There is no programme after the judiciary system, so when they are going out of prison there is no programme about how they are going to integrate back into normal society. It is very tough laws, but it's missing the humanity. That's where we need to put the focus on. We need to work in teams, with community members on the side with authorities and politicians making the decisions. (…) quite often we find under various victim services policies that are out there they are very specific that because these are terrorist incidents that you have taken part in or they have travelled to another country and that the family doesn't qualify for the typical victim service benefits, so they get cut out of what's supposed to support them. A big concern and worry in families is when there are younger siblings its very easy for them to follow in their older siblings footsteps, so unless we are supporting them adequately its quite often that you will see a brother or sister follow along. Because the supports aren't there. So, they are treated differently than other victims of crime, in their jobs there is nothing protecting them, at their jobs quite often people are getting pushed out, get fired because of the discrimination and the stigma that they carry. Psychological services are much more difficult to access, there are not a lot of people with training in this type of area. So we are limited there. We have watched a lot of families fall apart, illnesses, cancers, mental breakdowns, medications, cant work anymore, heart attacks, high blood pressure, alcoholism, drug use, gambling, because of the stress, and so it creates this triple effect, because they

[15] Mothers for Life, a unique global network of mothers who have experienced violent jihadist radicalization in their own families. For more information see: http://girds.org/mothersforlife/mothers-for-life-network

This project has received funding from the European Union's Horizon 2020 research and innovation programme under grant agreement N° 699824.

can't access any supports it grows into a larger problem." (Female End-User, Mothers for Life Association)

"It becomes problematic, we now have a case of a girl whose brother has gone to AL Qaeda in Syria, and she receives counselling from us, we guide her to keep the contact with her brother to influence him and to find out what is going on. She participates in our Parents group. And suddenly the Criminal Police came and confiscated her mobile phone, with all kind of data on it, and the police officer requested all the laptops arguing that she was still in contact with her brother, and she told him about the counselling, and he just said they don´t care about counselling. We try to get to their superiors now. These were the comments of a small police officer, and this is not how it works! And there is data that they are not even allowed to analyse. In order to judge somebody they need evidence, and sometimes they send these videos or pictures, but the families have the right to silence and the right of refusal, so sometimes they just confiscate the mother's mobile phone to get the evidences. We have seen that quite some times. This completely destroys the trust. (…) We have a parents' group on whatsapp and that is all on this girls' mobile phone. We hope that… because that's not the way it goes, this is confidential. We have contacted the security forces because this case was in counselling, but they will have to get in touch with us. And this case only came to them, because the son has left to Syria without the police taking any notice, and because we encouraged the family to go to the police and take them on to the boat, and we will support them, so the family made a report that their son was missing. Although the family made that report, they never heard anything from the police for 2 years and now that!" (Stakeholder, Germany).

3. Second order of harassment of Muslim people working in prevention or deradicalisation

Second order of harassment is not only limited to family members of the potential real terrorist, but it also extends to all those people working in relation to these suspects in prevention and or deradicalisation. Especially deradicalisation implies working with people who are or have been radicalised and have been at least close to terrorist networks. Thus, proximity of the workers to terrorist networks is close, due to the crucial work they are doing to help those who want to get out of these networks and those who are at risk of becoming further involved in these networks to actually achieve their goal of pursuing a different path. It is noteworthy that this second order harassment as far as we have learned from the interviews only affects the Muslim community. In our fieldwork no non-Muslims have suffered from second order of harassment due to their work in prevention or deradicalisation and their potential ties to suspects or terrorists. This does not mean that it could not be the case, but it is at least a less present reality so far.

This project has received funding from the European Union's Horizon 2020 research and innovation programme under grant agreement N° 699824.

Some cases that the people we have interviewed explain evidence the impact that policies have especially on the treatment with Muslim community that is working in the field of prevention or deradicalisation. They are much more affected by rough measures and public defamation than a non-Muslim. In this sense we will briefly summarize one case reported by an expert working on deradicalisation with parents.

It is the case of an Imam who lived in Germany and then moved to Spain. According to the expert, he fulfils the typical stereotype of a Muslim who does not live according to European standards; he is physically very alike to 'Osama bin Laden' (as he explained in the interview), has 4 wives and 15 children. He was reported by one of his wives for domestic violence, which has been archived, because it was a false accusation. He was working against radicalization and is a crucial partner to Hayat. Even Daesh has made a call to kill him. However, law enforcement in Spain arrested him using anything that could evidence a relation to terrorist networks against him by accusing him of promoting terrorist networks and recruitment and so does the media. The expert explains the situation and outlines some of the measures that have been implemented due to the CT legislations in Spain and Germany:

> "He does deradicalisation in Barcelona and all along the coast, and he is supported by the Emir of Kuwait who also sponsors Real Madrid, but now they say he is funded by terrorists, but when it comes to sponsoring Real Madrid he is accepted but not for deradicalisation work. Horrible. I know the accusations and this is nonsense, he questions Al Qaeda and Saudi Arabia and whoever else, and now has to travel with bodyguards because his life is in danger. Spain accuses him of being a terrorist, and argues that he went there with guns, and this is violence and means that he is radicalised. I told them that they would need a very good lawyer, and they found one, but he first wanted a lot of money, so they sent money from Germany, more than 10.000€, so the Criminal Police came to check this for money laundering, so the bank transferred the money but then cancelled all the accounts of his family. They have no accounts in Germany anymore, they cancelled them all! They didn´t even want to talk to me because they had wired his phone and now I´m involved, too. And the accusations can be easily verified. (…) The lawyer couldn't go to talk to him for 2 months, and they know nothing. Now that he has a lawyer in Madrid they transferred him to a prison in Cadiz, so it is even more expensive. (…) If he wasn't Muslim, if that was me, he wouldn´t be in prison, but sitting on tribunes as an expert." (Stakeholder, Germany).

This expert from Germany also explains another case of Muslim deradicalisation workers and the consequences that disruptive measures have not only on the person involved and their families but also on the work that they are doing and are forced to stop doing because of the suspicions towards them. This is crucial

This project has received funding from the European Union's Horizon 2020 research and innovation programme under grant agreement N° 699824.

considering that these people are working with radicalised persons who are trying to get out of terrorist networks. A disruption of these ties is counterproductive in this process of deradicalisation.

> "There was this case recently of VPN in Hessen and the two colleagues who suddenly were suspended from work, because a journalist and a self identified Islamism expert in Hessen, in Frankfurt, said that these counsellors who were Muslim, were undercover islamists. And even the Ministry for Interior was impressed by that and they made a huge story out of it, so they suspended them, but of course they were no islamists, one of them was at a conference with islamists, well with people who are… are they only conservative or islamists, they had previously been islamists, well they were absolutely in their competences and after the investigation they could go back to work, but one of them then explained the loss of confidence that this implied for the clients. They are doing work by going towards the people, projects with youth in prison and then they couldn´t go there anymore, and you can imagine, they are in conversations with someone who is considering leaving this, leaving this ideology and to integrate into society, but knowing that they will never be truly accepted. And then suddenly his counsellor is being suspended because he is being defamed as an islamist, that is a huge loss of confidence.(…) that affects especially Muslims more than anybody else, obvious. They always have the feeling to be potential suspects. There are many Muslims working in this field, and it implies a certain mistrust which is why such a report has much stronger waves for a Muslim than for non-Muslims." (Expert, Germany).

4. Perpetuating stigmatisation in diverse social spheres such as science, media and education

The stigmatisation of the Muslim community as a suspect community is not only result of police intervention or law enforcement procedures. It is further enhanced from diverse social spheres, such as science, the media and education. In the following we will display some of the implications and effects of how society contributes from these spheres to the negative impact of CT policies.

In the first place, Mustafa Cimsit and Misbah Arshad, both working in prevention of radicalisation and deradicalisation of Muslim youth in youth retention centres, emphasize the need to eradicate anti-Muslim racism from science. A common phenomenon of science on certain collectives rather than with these collectives is to reach conclusions that are not accurate and contribute to making these social groups even more vulnerable. As Muslims, they both work in science to contribute with evidences on how to overcome this perpetuation of stigmatisation in science, media and education.

This project has received funding from the European Union's Horizon 2020 research and innovation programme under grant agreement N° 699824.

“We are both working on our dissertations and we see how things are communicated, how populist things are communicated, and when non-Muslims, self-identified experts in Islam elaborate theories on Muslims and about the religion Islam. Central terms of our faith are used in science in a very diffused manner. We can evidence this with three terms that have become catchwords for international terrorism, these words are: Islamism, Salafism, and Jihadism. We have to provide our own and corrective theories, and draw on our tradition to clarify this before the youth. In Islam the word Islamism contains Islam, which for Muslims is a positive word. In the word Salafism is the word Salaf, the emulators, the descendants of the Prophet Mohammed, thus for Muslims a positive term. The word Jihadism contains Jihad, which is a central term of the religious life practice of Muslims. To pray is Jihad, what we do in this interview is Jihad, that I am smiling at you is Jihad. So all these central terms become synoym for terrorism; they are again and again thrown at the youth it is a catchword. We have to clarify this to them. But we experience that these terms are worldwide used as catchwords, although they are wrong. We just put a suffix in the end and use it as a synonym for terror, but we don’t do this for Christianity, or for Judaism, for Buddhism, we do this in the Islam. (…) To be aware of this and be over it means that you say ok, if I am in a hotel and there is an old lady at the reception saying ‘Oh you are going to the islamist event’ then I know that she had been manipulated, she cannot distinguish one from the other, because she constantly hears islamist she thinks it is Islamic and confuses it. I know that and I don't blame her or get angry and say ‘Who do you think you are to tell me I am going to an islamist event, Hello?!’ (…) Science is nurturing this, so we are working ourselves scientifically to evidence these problems in science” (End-user, Germany)

Second, the media marked by the ethnocentrist nature of European societies is contributing to islamophobic discourse and actions instilling a greater sense of fear, vulnerability, insecurity and helplessness of the Muslim community. Anti-Muslim discourse in the media is no news, but still we will provide to examples to evidence the importance that every single headline has on the lives of people. Mustafa Cimsit, gives a brief example of the media while he is talking about the importance to eradicate stigmatisation from science as we have seen in the previous section.

“When we see that, if a father of a family kills the family, and if he is non-Muslim, if it is a Bavarian family, then we call it a family drama, but if it is an Islamic family, if they are Turkish we call it an honor killing, due to his tradition, his faith. This anti-Muslim racism, because this is clearly anti-Muslim racism, we have to eradicate this from science (…)” (End-user, Germany)

Another example is provided by another stakeholder in Germany regarding the previously mentioned case of an imam working in deradicalisation accused for connections to terrorist networks. It is noteworthy that the

This project has received funding from the European Union’s Horizon 2020 research and innovation programme under grant agreement N° 699824.

media had a great impact on the life of this person and enhancing the implementation of policies in a detrimental manner to the suspect who was falsely accused of domestic violence. The yellow press would call him ‘Prügelimam’ [Beating Imam], even after the case was archived. The expert states:

> “Until today [7years later] when he was now arrested, the Bildzeitung and the Bayrischer Rundfunk publishes headlines like: the Beating Imam is a terrorist now. The headlines were outrageous.” (Stakeholder, Germany).

A young Muslim man explains that he feels victimised by the media and even feels blame for terrorist attacks although he has nothing to do with it.

> “Dignity... I think they stigmatize us, they put us in the In the eye of the storm , I have often felt victimized (...) and even I blamed myself to a certain point (...) You are afraid let's say, because you know that you are responsible, I think that this idea is also well portrayed from the discourses that come out on television, from the media. (...) the effects derived from this fact [CT policies] are often expressed through media, yes, this is rather for me, I am going to use a term to express it, so that it is a kind of good theatre, it is used more in political terms than in real terms, in many theatres.... There is a lot of show but the base is very thin. That is why I say that if there are plans or whatever, if they are implemented and whatever and it is done carefully, I believe that community will be the most interested in the fact that these people do not go out and that they are detected and the maximum collaboration being provided to authorities”. (End-user, Spain)

In spite of ethical regulations of the media and guidelines to not perpetuate stigmatization of specific ethnic minorities in the media, we can see that in general, there is a spread of negative discourse that can affect the integrity of the person, as well as foster the feelings of exclusion, discrimination and criminalization. The expert interviewed in Spain for the media dimension quoted a research which showed that from the film production of Hollywood, 86% of them represent Muslims like terrorist or criminals. We have to be aware that these kinds of racist reporting in the media can be developed with no consequences for the journalists or the newspaper itself, while enormously contributing to a racist discourse and to the creation of a suspect community.

Finally, stigmatisation of the Muslim community also takes place in education. Some of the interviewees especially mention the PREVENT Strategy in UK or the PODERAI in Catalonia as the reason for this negative outcome, thus underlining the impact of CT policies on different social spheres. These policies foresee that teachers should be sensitized to the problem of radicalisation in order to report potential signs of radicalisation

This project has received funding from the European Union's Horizon 2020 research and innovation programme under grant agreement N° 699824.

among their students. In line with the results of previous research (Fischbacher-Smith, 2016) however, this is highly controversial, because no thorough training is provided to the teachers. Thus, the identification of early signs of radicalisation relies on the sole criteria of every single teacher. In this regard, a high-school teacher in the United Kingdom, explained that with the PREVENT strategy, students who used to talk about their concerns stopped doing it because they didn't feel with the trust of not being reported. In the UK, the report edited by the Open Society "*Eroding Trust: The UK's Prevent Counter-Extremism Strategy in Health and Education*" (2016), denounces this type of situations. More evidence of this impact is given by stakeholders interviewed in Spain, in this case the Director of a primary school in Spain, committed with an educational Project that promotes Inclusion rather than segregation:

> "(…) the legal issue does not arrive here, instead real problems arrive. The crisis has affected generating many tensions that causes a general disease. They do not care if laws take into account social integration and cohesion; the only thing that they consider is that the view towards them is exclusionary. Although an attempt has been made to include them, it has not been achieved (...) From the department of education and the Mossos [local police in Catalonia] teachers were called to explain the PRODERAI CE protocol, and also to explain the indicators that could be considered as risk of radicalization. At the end of the course, in Terrassa, all the schools' principals were called for a meeting, but just those from the school with higher diversity. They were offered to hear the explanation of the PRODERAI protocol, and to be trained. It is not only necessary to know that there is a protocol, that school principals absorb the information and transmits it to the teachers of their schools. This is not prudent, because principals do not know the teachers' sensitivity once receiving such information. They may have prejudices/stereotypes and misapply those indicators creating more stigmatization (a teacher can come in his/her personal comfort zone, and distinguishing between his/her society and others). It also seems that the Education Department only applies this protocol to those centres with greater multiculturalism, and that is not the focus of the problem, but rather it is a "problem" / situation that affects everyone, not only those schools with more cultural diversity. In addition, they have not been told who was in the design of it, nor have they been given training to help the integration of students." (Stakeholder, Spain)

> "If you have a look at the schoolbooks and how the Islam or Muslims are presented, this is always in the same direction, that it is incompatible with democracy, with the human rights, it is underdeveloped, it is always on that level. I think if Muslim adolescents hear that in school, how do we expect them to identify with that, with the constitution and democracy and whatever. We only convey the same image that, let's say so, the Muslim extremists on their part set forth: Islam is incompatible with democracy, there is always this gap, and this is also what we can find in schoolbooks. We need to be aware of the damage this causes." (End-user, Germany)

This project has received funding from the European Union's Horizon 2020 research and innovation programme under grant agreement N° 699824.

Therefore, stigmatisation resulting from the implementation of some CT actions is affecting to the same extent those who are radicalised and those who are not. Due to these actions, all Muslims end-up being portrayed as an homogenic social group, associated with being ‘backwards’ and violent:

“I totally agree, I believe that the profile we have found in many attacks in Paris, in England or so one, we always have a profile of someone who is using drugs or is involved in robberies with violence, who goes to discotheques, who consumes prostitution… and they commit all those acts that are forbidden by Islam, but they still have the Muslim identity and that remorse. To compensate, they go to the other extreme and it is a totally erroneous and fascist interpretation of religion. And for me it is the biggest blasphemy that could exist, to use religion to commit an attack and they do it to calm their feeling of guilt. For me, what you have said is key, it is key that crime circuit, where they came from, and all those circuits, why do they get there? Because of the lack of opportunities, due to the environment or other reasons, right? From there they go to an extreme, because they are already trained to say it in some way, they already know how to move and then move on to another action is quite easy. For me those people are very…, you have to control them a lot, because those people are more dangerous profiles, at any moment they can change sides" (End-user, Spain)

2.2.2. Transformative dimension: elements that facilitate the positive achievement of social impact and protection of fundamental rights of counter terrorism policies

The review of scientific and grey literature has evidenced that special emphasis was placed on highlighting the negative impact of CT policies on society. As we have seen in the previous section on the exclusionary dimension and those elements that hinder the achievement of social impact and the protection of fundamental rights, this analysis is crucial, as relevant elements can be identified and need to be taken into account when considering revisions or the implementation of these policies. Yet, the positive impact of these policies has so far not been further analysed, but is equally important, because it sheds light to those elements that we can actually foster to further promote positive social impact and make counter terrorism policies effective. The present section is dedicated to this endeavour and provides insight into these elements that we can identify across countries that are helpful in effectively translating CT policies into prevention measures of terrorist radicalisation.

1. **Police intervention as a positive security measure**

This project has received funding from the European Union's Horizon 2020 research and innovation programme under grant agreement N° 699824.

In contrast to the negative impacts of policing in the previous section, here we evidence the positive impact of police intervention. While police intervention can have a very limited impact if this intervention is directed against a whole social group, it can also convert this negative effect into a positive intervention, when instead of stigmatising or criminalising ethnic minorities and especially the Muslim community it counts on their collaboration. In this regard, one of the interviewees explains how they transformed the relation between the Muslim community and the police to join forces against terrorism. He highlights that egalitarian dialogue is crucial to have a good collaboration and values this from the police president.

> "Our relationship is good now because of certain escalations in the past. About 20 years ago there was this case of an association of Mosques, where I was the person in charge of public relations and the police wanted to get in there, and wanted to catch somebody fresh. And we blocked that, we did not allow this. We said 'Dear Police, dear Operational Forces, if you do this, without our permission, well you can do this with your weapons and because you have legal power to do so, but this will have consequences.' Then some strong words were exchanged and then they asked the head of operations, and finally the police retrieved their forces. We sent an angry letter and I clearly told them 'Dear Police president, if a stranger had seen the scene and your forces hadn´t been wearing the uniform, it could have seemed as if right-wing extremists were attacking the mosque.' But since we talked straight, and both sides were willing to learn from the mistakes a different culture came up in which we discuss things and solve issues before they become a problem. The migration director developed a protocol with our help on how police has to act in these cases. So, cooperation on equal terms is fundamental." (End-user, Germany)

Another example of positive police intervention leading to preventing radicalisation is that explained by a Dutch police commissioner for the CT unit in his city. He emphasizes and explains the implementation of preventive police intervention rather than repressive methods.

> "An important issue is balance between repression and prevention. Counterterrorism activity can have bad consequences, for instance if migrant people feel welcome in the Netherlands, that's why we invest in alliances with all kind of groups in the society. Allies in the neighbourhoods, in Muslim groups, Turkish people, Jewish people, on all levels, on the neighbourhood, with police officers, team level in the police, and the city. Three levels that we work in. We do: Collecting information; Know what's going on in the societies, how people feel, how they experience relation with governments and police, to detect. We communicate, we are an open organization, every citizen is equal, we are interested in our communities. (…) The focus on recruiters, it is important to get signals from society about recruitment. We collect a lot of signals from allies, so we can act immediately when radicalisation is

This project has received funding from the European Union's Horizon 2020 research and innovation programme under grant agreement N° 699824.

> beginning. We cannot guarantee that we pick up every signal, we have missed a lot, and a lot of young people have left, about 400 young people left to outreaches of ISIS in Syria. We have a lot of examples of successful interventions, it is important to get the signal, if you don't get it things happen behind your back and then its too late. We have a case discussion, every signal is discussed, police prosecutor and institutions that cooperate in these networks, to get the right intervention, and sometimes it is a repressive intervention with arrests, but we also have a targeted approach, relying on the aspects of the case. For instance providing help, we see a lot that young Muslim boys who are confused about religious issues, and a few imams will help us counteract that problem." (Stakeholder, the Netherlands).

The Police commissioner further explains how this theoretical explanation of the CT police interventions is implemented by providing the example of one case:

> "About a year ago we got strong signals of radicalisation of a young man 19 years old at that time, the signal was discussed in our case meeting, one of my police officers had a good relation with that boy, and invested in that relation with the pains, and with the help of the therapy, because he had a job and he was helped with his studies, and for his religious questions he was with an imam. And this intervention, the whole package led to disengagement of this boy about a year and a half. We are still monitoring him because this disengagement might not be the end stage, so he will be monitored for a few years to see what's happening and during that time there is contact with him now and then. He is aware that he is monitored, we are always totally open. And when possible we go together with parents and with other people of the surroundings of the subject." (Stakeholder, Netherlands).

2. Interfaith dialogue and cooperation among religious leaders

Stakeholders from the diverse countries where fieldwork was conducted have implemented actions that promote interfaith dialogue or the collaboration of religious leaders from different religions. These initiatives promote a positive understanding of religion as it evidences to the participants that their religion does not stand in contrast with other beliefs or the human rights. It helps to strengthen the participants' self-perception as a religious person in secular societies or in societies where a different religion is prevalent.

One of the experts interviewed from Italy explained about the existence of different non-state directed programmes developed in prisons to promote interfaith dialogue and prevent radicalisation. Some of these programmes are taking place in the city of Milano and Bolonya. Besides, he explained that the Faculy of

This project has received funding from the European Union's Horizon 2020 research and innovation programme under grant agreement N° 699824.

Architecture in Milano is working on thinking how it is possible to conceive spaces within prisons that encourage diverse religious interactions between inmates as well as between inmates and prisons' stuff.

In Catalonia there are programmes fostered by the Department of Religious Affairs that promote interfaith dialogue at the grassroots level. In this line a Rabbi who participated in a focus groups about religious diversity and immigration in relation to radicalisation explained:

> "We work with Religious Affairs Department and the City Hall of Barcelona. They promote visits of public and private schools to different religious institutions. Institutionally there is a lot of backup in different sectors (prisons, schools, etc.) so that people can practice their faith. Finally there is a topic that is confused, we need to distinguish between a person's needs, for instance not to find a job because you do not have the necessary training or you do not fulfil the requirements, and the discrimination because of religion (…) " (Stakeholder, Spain).

In Germany, different programs have used this approach to foster integration and prevention of radicalization among youth. For instance, Kompass[16] has developed several interfaith sessions with a very diverse group of Muslim youth. The participants were from diverse national backgrounds, speaking different native languages but sharing the same religion with differing traditions and practices. The group was held in German, the only common language between all participants and their second native language. They have done sessions inviting Christian pastors to discuss the similarities in faith or sessions on religious-based extremism, antimuslim racism and the conflict in the Middle East:

> "It gives you strength, we have discussed different topics. And the feeling that you…. I don't know why I am crying now… (…) Well Kompass has given me a lot, all the topics we discussed, were topics we didn't discuss anywhere, in most spaces they were not discussed, topics such as homosexuality, this is almost never addressed anywhere, but we experience this in our everyday life, and in this regard Kompass gave me very much and a variety of things. To be open minded, it all starts with being in German, in most communities they use the native language, Bosnian or Arabic or Turkish or whatever, and thus the relation to the society we are living in is missing. (…) well prevention of extremism or the fact that you are able to respond to someone who verbally attacks you. For instance when someone says oh you are wearing a veil, that you can be above that and that you can better respond to certain questions." (End-user, Germany).

[16] For more information about the Kompass project, see: http://muslimische-jugendbildung.de/

This project has received funding from the European Union's Horizon 2020 research and innovation programme under grant agreement N° 699824.

To provide an example of the results obtained in these sessions, the youth elaborated posters claiming for “ideals instead of ideologies” (Documentation of their sessions provided by Kompass.

Gangway,[17] another organization of social workers addressed to working with youth on the streets of Berlin, also fosters interfaith dialogue when they identify racist attitudes based on religion. One of the social workers we have interviewed explained that in one case of Arab and Turkish youth they identified anti-Semitic comments and attitudes and thus organized a trip to Turkey and Israel visiting different religious institutions in which they practiced interfaith dialogue:

> “A group of Arabic Turkish adolescents from a specific neighbourhood, who were very anti-Semitic due to the situation in Palestine and the West bank and Gaza. This situation was 2-3 years ago, when the situation was at a peak and the anti-Semitism was on the rise. Some colleagues decided in a longer process to travel with a group to Turkey, well the west bank and visit specific projects and then go over to Israel all with these adolescents. They were so sustainably impressed, because in Israel it is not that every Israeli shoots Palestinians, which was their idea that every Israeli was a potential assassin of Palestinians which guides this radical thinking. The constellation of projects they visited, with some Israeli and Palestinian projects that do many things also on a cultural level and Israeli mothers who take care of Palestinian orphans. They visited several projects and then after that trip they were very impressed, they were irritated in the first place, because it doesn't mean that they are pro-Israel now, but they were irritated because their ideas and prejudices were wrong. They got to the conclusion that ‘I will have to think about what kind of person is in front of me’. Diversity at it’s best so to say.” (Stakeholder, Germany)

3. Prevention and deradicalisation strategies developed in the community and with the community fostering counter-narratives that do not glamourize, but reject violence and radicalization

The European Security Strategy (2015) mentions that “exit strategies generally rely on individual mentoring consisting of psychological support and counselling (…) these strategies should engage with families and communities which are often best placed to contribute to deradicalisation”. Also mentioned in the ESS, we have identified projects in which victims tell their stories, which indeed is part of their personal recovery and part of the effort to create new counter-narratives. It should be noted, however, that the RAN has elaborated

[17] For more information about Gangway, visit: http://gangway.de/

This project has received funding from the European Union's Horizon 2020 research and innovation programme under grant agreement N° 699824.

guidelines in order to take into account not only the opportunities but also the challenges that involves working with ‘former extremists’.[18]

We have identified that Associations working at the grassroots level in the UK, Germany and in the Netherlands are already developing these types of counter-narrative actions, having a positive social impact on those who have been radicalised or might be under risk of radicalisation. This type of actions needs a cross-sector collaboration between different stakeholders.

In the case of UK, the Active Change Foundation works with people who have been radicalised, implementing programmes of de-radicalisation. The interviewee explains that they will count with the support of people who had been in Syria, to promote programmes of prevention with children and youth, and they also work together with families, a key ally in the process of identification of when someone is radicalising:

> “(…) A lot of our referrals come from the family. The family is the first to note whether their son or daughter is becoming extremist, or is getting into organized crime (…) When the family comes and asks for help is because the boy or the girl is flirting with violence… they know that they are changing their friends, they know other things and do not know how to cope with that. Always there is something (…) Once you got the cooperation of families, we can either intervene directly or we can ask the parents to intervene: so we ask the parents to use their bonding capital to intervene. If they don’t feel capable of doing that, we ask the parents to do introductions and to endorse us to intervene. We will then continue with the engagement with the young person. I say to them “I am not here to change your point of view… just to explain you. I cannot change your views if you don’t want to change”… It consists in building trust with them (…)” (Stakeholder, UK)

In a similar sense, the international association Mothers for Life, which came into being due to the lack of assistance of families who had suffered the loss of their child to Daesh. They not only support families who have lost a beloved person, but collaborate with schools and communities to prevent other families to go through the same process. In spite of the difficulties that this mother mentions that they encounter, she also explains that they have a major impact on youth because they speak in egalitarian terms with them and they provide a safe space in which these issues can be discussed. Some of the stakeholders working at the ground, specially in the UK and Germany, explained that many times there is limited space among school settings in which Muslim youth would no longer feel secure to open up about their concerns for fear of being reported:

[18] The RAN has also issued an ex-post brief document (2017) in which provides guidelines for those employing formers as coaches/mentors/practitioners in exit work. For more information see: https://ec.europa.eu/home-affairs/sites/homeaffairs/files/what-we-do/networks/radicalisation_awareness_network/ran-papers/docs/dos_and_donts_involving_formers_in_pve_cve_work_bordeaux_27_06_2017_en.pdf

This project has received funding from the European Union’s Horizon 2020 research and innovation programme under grant agreement N° 699824.

> "Families are not worked with but are pushed out. We are seen as nobody, we don't count, so we're cut out. With our prevention work in schools with youth, it is very difficult to get into schools because of the political factors, we struggled, unless we have some very motivated teachers that take the initiative to push it forward and to fight to get us into the schools to speak with the youth it's very difficult because of the hierarchy, directors are afraid, they are busy, picking the part of political correctness but then don't want to get into trouble, who is gonna pay for it (…) It has to be on the community level, there needs to be more mentoring and support, empowering youth to find more positive methods to express their dissatisfactions, or have open and safe areas for open dialogue to be supported and not ridiculed or pushed away. Quite often there are a lot of waitlists in the state driven programs, because the funding not necessarily are there as well as the prevention in schools, give an opportunity to educate the parents who are going to educate the youth, educate the youth, we are not looking at one type of extremism, we see rightwing extremism is growing as well, so what we need to do is start educate the youth at various places, how to connect on emotional levels, how to find positive outlets to express themselves, and we need to put the support into place". (Female End-User, Germany)

In Germany also Hayat is working with families to help in deradicalisation of youth. Families can contact them when they see signs of radicalization and they will help them deal with their children to prevent them from definitely becoming radicalized and engage in terrorist networks as well as in deradicalisation if it is already to late for prevention. One of the representatives explains the current status of their work in numbers:

> "Since 1.1.2012 to today we have had 386 cases. 170 of them are still active, the rest have been closed, but not all cases had a positive resolution. There are very easy cases, when parents call us very early when they see changes in their children, one or two meetings are enough to solve them. But then there are cases when parents can't take it, and abandon and deny any intervention. Other cases are those in which we have made counselling for a long period of time and in which we have stopped or reversed the radicalisation that had taken place. Then there are cases in which the person has radicalised but does not represent any danger to anybody, not to herself and neither to others. We don't mind how conservatively religious this person might be we distinguish between security level cases and those that are not relevant for security. About one third oft he cases are relevant to security. This means there is a certain danger for traveling to a jihadist group, or someone has already traveled there, someone has come back or someone is in certain social circles in which we have to suspect that there is preparation of terror attacks. We have 90 cases that fall into this category, 15 have been solved positively, this means they are no longer a threat (...) we have 36 cases that are still ongoing, and 11

This project has received funding from the European Union's Horizon 2020 research and innovation programme under grant agreement N° 699824.

> cases that were closed because the clients have died (...) and 24 cases where we have no news from the children" (Stakeholder, Germany)

In the case of the UK, an interviewee mentioned the activities that the *Connect Future* association[19] is doing, for instance, with former radicalized youth who are explaining their stories. The interviewee mentioned the programme: BRAVE. Building resistance against violence and extremism (Connect Futures and St Giles Trust):

> "What they show to young people is that there are huge similarities in the ways to access to gangs and the way you access to extremism, and the way they work (…) The idea is to go to schools and to de-construct both… Maybe they are quite tempted by enter…" (Stakeholder, UK)

One of the issues of concern raised in relation to this aspect is the importance of intervening at the very right moment: before the person has completely radicalized. The challenge here is to identify this moment, and act, otherwise extremist groups make the person to 'burn down the bridge'. This was the way how a former radicalised person interviewee explained the process of radicalization and how people end-up breaking up with the networks of friends and with the family.

The different participants in the fieldwork mention a variety of elements that influence the effectiveness of intervention, which has to be done taking into account the complexities of each situation. Thus, can exist common pull and push factors leading youth to engage in terrorist networks and violent extremism, but not a single one. Three factors are crucial and are also in line with previous research on preventive socialization developed by Lidia Puigvert (Free Teens Desire project, 2015-2016): the peers, the perpetuation of dominant violent masculinities in the model of false heroes, the mirage of upward social mobility of girls who are victims of grooming and bid, for instance, on travelling to Syria to fall prey to the abusive practices of terrorist networks.

Although there are only few programs that actively respond to these social phenomena, the research participants agree on the importance to take them into account identifying the given realities in their contexts. The interviewee from The Netherlands, with a background of having been involved in an extremist Salafist group highly insisted in the need of doing more prevention:

[19] For more information about Connect Future, visit: https://www.connectfutures.org/

This project has received funding from the European Union's Horizon 2020 research and innovation programme under grant agreement N° 699824.

“There’s not one thing in prevention, I think that what we have to do in Europe is building a big network of prevention, and we should do it in learning people who are in contact with former… and also learning how to deal with extremist organizations… we can do it schools, at sport clubs, at TV… We can do much more in preventing” (End-user, The Netherlands)

a. **Peer groups: trustworthy friendships**

In order to prevent radicalisation some programmes, such as Active Change Foundation emphasize the importance of disrupting networks of friends who might be negative for the person in question.

“(…) peer to peer is the most powerful bond you can have, you are stronger with your friends than with your mother or father. You have a stronger bonds with your friends than what you do with your parents when you are a teenager. Therefore, the influence in fact is …. You follow what your friends do, this is how you keep your bonds (…) The very first person that you’re going to recruit is the very close friend to you.” (Stakeholder, UK)

Also a stakeholder and end-user, both from the Netherlands emphasize the importance of peers who can either recruit or save lives. The End-user interviewed who had been radicalised and then de-radicalised also mentions the importance of doing prevention programmes specifically promoting that children and youth think critically: about the elements that can lead to radicalisaton, about networks of ‘friends’. When interviewed, he explained the key role that played some people whom he knew, in bringing him into the Salafist group:

“[At that point: ‘I was more fighting the educational system than studying’. So at that moment I had lot of interest in Islam, and only mosques had very good information about Islam and how to convert... lot of books, lots of materials. I knew some Moroccan guys who brought me there. I knew them from schools… we were in the same class... You go to have something to drink with them, you talk about religion, faith… And they brought me there (…) and that put me into the Salafist movement (…) But they were not really religious, they found interested finding a white guy wanting to convert. This was before 9/11, when if you said that you were interested in Islam was totally different from now (…) [Talking about the importance of friends] for those radicalised, for some of them friends can be important, for others no. A lot of them just break up with friends… Many time they talk about ‘burning down the bridge behind you. And I think that this is a beautiful way of describing it: you go through an extremist group, and when you burn down the bridge behind you, you could not go back any more, to your friends, to you family… and that’s the same with all different radical groups. So if you want to

This project has received funding from the European Union’s Horizon 2020 research and innovation programme under grant agreement N° 699824.

> leave but you don’t have connections is very difficult, so many people just stay inside the group”. (End-user, The Netherlands)

In a similar vein and talking about the end-user, the stakeholder mentions the powerful role of friends in preventing and deradicalisation.

> “He radicalized in the Medina, but he has one friend: he was very closed from early childhood and he kept contact with his friends. He says that his friend saved him. He explains that while he was living in Saudi Arabia, his friend contacted him, texted him… And the Dean of the Islamic University at some point told him: you have to give in your telephone, and your content” And then he discussed that with his friend, and that was the moment when he thought to get away, to flight.” (Stakeholder, The Netherlands)

For the case of the person interviewed who was radicalised, he explained that he realized about that and he ‘started his process of de-radicalisation’ thanks to keeping just one friend, who while he was in Medina, continued talking to him via mobile phone.

b. Challenge the social perception of violent subjects [false ‘heroes’]

Contrary to the declarations that many persons who knew the adolescents who carried out the terrorist attack on The Ramblas in Barcelona in August 2017, who expressed that they 'cannot believe how such type of guys could do it’ (justifying that these were not violent young adults), the police officer who was in the area remembers the situation and the attack with the van as follows:

> “He zigzagged looking for the stands and kiosks where people were distracted and unable to react. He wanted to cause as much damage as possible." (Declarations of one of the police officers who saw the Barcelona terrorist attack, in August 2017)

Later on, we knew that the terrorist who was driving the van loved the videogame ‘Grand Theft Auto’, in which the incentive is to run over pedestrians and the player “recharges lives” by having sex with street prostitutes.

This project has received funding from the European Union’s Horizon 2020 research and innovation programme under grant agreement N° 699824.

c. Tackling grooming and promoting healthy relationships

Some interviewees mentioned the importance of working on 'healthy relationships' with both male and female adolescents. They emphasized how for the case of girls, they are first victims as for the case of some types of organized crimes, recruiters use grooming on them. The presence and pressure of a dominant model of attraction that links violence and attraction has been identified as an element that is playing a role in the way recruitment takes place:

> "(…) They were groomed online, and they were groomed online by women who in Syria, who has already gone to Syria, who were making contact with them and grooming them. And one of the things that the girls wanted is, if you looked at their lives in London, they were from Bangladeshi, Pakistani, Somali communities, and their future was going to be: "you gonna get marriage to some guy". That was the future that they were looking forward to in their communities. And going to Syria offered them the opportunity to marry someone different. So they wanted to get marriage to converts to Islam… So they had like a 'poster-boy': a good looking-blond, blue-eyes, Australian, Jihadi, who they were using these images saying [the groomers] "This is the one who you are going be meeting, you are not going to be meeting a Bengali, o Pakistani man. These are special men who come here and we need to find wives for them"." (Stakeholder, UK)

In a similar line, another stakeholder working at the ground in the prevention of gang violence and extremism explained:

> "If you think about how young people learn, they learn by stories and symbols. And they have access to a library of stories and symbols: the videogames, the you tube, the music that you listen to…. A lot of these have the fundamental underline of conflict and violence. This is something in what you wake up everyday. As a result of that you become that. It is not abnormal to talk about violence, your language is aggressive in nature… In certain communities is the norm to consider violence in your conversations. Then it just has to be exploited by organized crime or extremism…. But is already in your everyday life… is subliminal… you learn it while you go along. You have to think that in here every young boy knows what a gang is, what they do, everything…. It is also in the music (…)" (Stakeholder, UK)

This project has received funding from the European Union's Horizon 2020 research and innovation programme under grant agreement N° 699824.

d. Alternative grassroots movements to counteract violent extremist positions

There is the perception that there is a lack of countermovement against extremist positions, but there are individuals and communities working against these discourses. Examples of this are youth movements working at the grassroots level, vindicating that Islam is not Deash. After the terrorist attacks of Barcelona (as also occurred after other terrorist attacks perpetrated in Europe), these type of movements have re-emerged with even more strength, positioning against violence and defending both their religion and their identity. The ‘Association of Muslim Students of Tarragona’ (Spain) is an example of this grassroots movement. The Muslim student interviewed for this study vindicated the defense of democracy and freedom.

Besides, one of the experts from Germany explains the case of Seyran Atesh who wants to open a reformist Mosque.

> “An example of counter-movement: Seyran Atesh. She is a lawyer in Germany and she is going to open a Mosque, a reformist Mosque. This is necessary and clear counter-movement. Women and men, Sunnis and shih praying in the same room, open to homosexuals, etc. but at the same time, is a small and insignificant proposal because it will be based in Berlin, the most liberal city in Germany.” (Expert, Germany)

4. Promoting democracy: teaching democracy and channels for civil participation

Another very prominent aspect in elements that facilitate the social impact of CT policies are the actions and interventions that promote democracy, especially among vulnerable groups for radicalisation. One of the interviewees from the Netherlands emphasized in this regard, that “democracy is not self-evident” and that it has to be taught.

Also Gangway, an organisation in Germany approaching youth on the street work for the youth to be aware of their rights and duties and empower them as citizens. The stakeholder participating in the fieldwork explained that a neighbourhood conflict evolving around a group of youth playing with a ball late at night on the streets. The problem here was that they city had closed the sports place where they usually played, because of the neighbours’ complaints about noise. The intervention in this situation involved convincing the youth to explain their situation and the need for a public space for them and their activities. As a result of the letter they then sent to the city hall, they regained access to the space under certain conditions both parties agreed on, to keep the place quiet after a certain schedule and to close it when they leave. The youth learned that they actually

This project has received funding from the European Union’s Horizon 2020 research and innovation programme under grant agreement N° 699824.

count and are taken into account if they use the formal mechanisms to dialogue about their concerns, rather than cutting car tires to manifest their discontent. They felt empowered by their actions and achievements.

Further experiences in this regard, is Kompass. As mentioned before, this project developed a series of sessions on diverse topics that affect youth, such as sexuality, religion, citizenship, etc. with Muslim youth from different migratory or national backgrounds. Their activities were held in German, the common language of all participants. After discussing a variety of issues, participants became increasingly aware of discriminating experiences, for instance, the negative representation of Islam in schoolbooks. They discussed the potential ways to raise this concern in schools emphasizing the use of dialogue before complaints or other measures. Thus, students raised this issue and managed to involve teachers to open up a space for discussion about discriminatory representations of Islam in these schoolbooks.

All these experiences evidence the lesson learned to engage in civic participation, by stating a certain problem or issue in an attempt to find solutions together with the different social actors involved.

5. Programmes oriented to prevent radicalisation within prisons, enhancing inmates knowledge of and access to their rights

In terms of interventions in prisons to promote the achievement of positive social impact we can see that a variety of programmes aim at guaranteeing access to services to reassure fundamental human rights. One representative of Violence Prevention Network explains that they help their clients in youth detention centres to know and access their rights.

> "We try to take the pressure away and to contribute to taking their rights. Even if this is a person that on moral grounds I would judge him for what he did, it is my task to provide this client with help in accessing their rights. And even if this only implies to accompany that person to the lawyer, or to say 'we are at a point where only a lawyer can help you and I will help you find a lawyer'." (Stakeholder, Germany)

Similar experiences have been identified in the UK, where some stakeholders are counting also with the participation of formers.

This project has received funding from the European Union's Horizon 2020 research and innovation programme under grant agreement N° 699824.

6. Positive role models which reject violence in the media

Contrary to the findings regarding the role of media in the exclusionary dimension, there are also positive examples of how media can contribute to providing positive role models that reflect the ethnic and religious diversity of our current multicultural European societies and also reject violence. In this regard, one female Muslim end-user gave the following example:

> "On TV there was a woman a gynecologist and she was wearing the veil, she was libanese, and her boss, the chief in the hospital, told her to become his assistant, but please put the veil down. And she said 'NO' and opened her own clinic and she was interviewed by a camera team, I don´t remember which one, and she said her environment where she was accepted her as she was and nobody complained. So it is possible if you fight for it." (End-user, Germany).

Finally, the following table summarises the evidence-based elements identified in the results, which advance the societal and ethical impact for CT policies

**TABLE 1. Elements that advance the societal and ethical impact of CT policies**	
1.	Police intervention as a positive security measure
2.	Interfaith dialogue and cooperation among religious leaders
3.	Prevention and deradicalisation strategies developed in the community and with the community fostering counter-narratives that do not glamourize, but reject violence and radicalisation
3.1	Peer groups: trustworthy friendships
3.2.	Challenge the social perception of violent subjects [false 'heroes']
3.3.	Tackling grooming and promoting healthy relationships
3.4.	Alternative grassroots movements to counteract violent extremist positions
4.	Promoting democracy: teaching democracy and channels for civil participation
5.	Programmes oriented to prevent radicalisation within prisons, enhancing inmates knowledge of and access to their rights
6.	Positive role models which reject violence in the media

This project has received funding from the European Union's Horizon 2020 research and innovation programme under grant agreement N° 699824.

4. Contributions to inform the operationalisation of the input for ABM simulations

The following table gathers the factors identified as relevant in the recruitment into OC and TN; they inform the operationalisation of the input for the ABM simulations

**TABLE 2. Contributions to start the operationalisation of the input for ABM simulations**	
1	The more policies encourage and support those not paying extortion money = the less income for the mafias.
2	The more policies support job opportunities targeted to those at risk (based on school drop out, low attendance, low income, health and not only migrant profile) = less risk factors
3	The more active labour market polices and other employment opportunities which capitalise the entrepreneurial skills and leadership capacity of those young people who have been involved or are in the process of engaging in organised crime networks = major social impact
4	The less glamour is put on the narrative discourses about violence in the campaigns against OC and TN = less popularity among youth
5	The more policies focused on evidence-based inclusive education = less risk factors
6	The more training on critical thinking which includes unveiling attraction to violence (=) more success in rejecting fake jobs, promises related to OC or violent extremism
7	The more obligation on coordinating actions among different professionals working in the same territory (+) and better defined competences of each of them (=) the better are chances for effective implementation of their work)
8	The stronger the network of 'protective' community agents (+) the higher the links of solidarity (truly trust) (=) more role models for rejecting OC and TN
9	The better the language skills, the better inclusion and deeper engagement with the community
10	The more programmes that promote 'healthy relationships' challenging the association between violence and attractiveness, the weaker are pull factors
11	The more critical thinking about who are friends and who are not, as they play a capital role in recruitment to OC and TN, the less attraction to them
12	No policies oriented to resocialisation and integration means, more risk situations for human rights abuse
13	The less stigmatising or criminalising ethnic minorities (+) higher counts on their collaboration (=) more in favour of stop and search prevention
14	The more policies identifying second order of harassment of the families and people working in prevention or deradicalisation (+) more bystander intervention = higher support from community members and professionals
15	The more policies that promote interfaith dialogue and cooperation among religious leaders = less disengagement from "real" religion
16	The more prevention and deradicalisation strategies developed in the community and with the community fostering counter-narratives that do not glamourise, but reject violence and radicalisation = less radicalisation
17	The more focus given to critical thinking based on the identification of real friendship and the dominant discourse on violent masculinities = less pull factors
18	The more policies based on healthy relationships (+) less mirage of upward mobility among victims of grooming and therefore = less pull factors
19	The more support to grassroots movements to counteract violent extremist positions = less radicalisation

This project has received funding from the European Union's Horizon 2020 research and innovation programme under grant agreement N° 699824.

5. Final Remarks

Terrorism is one of the main priorities in the security strategy of the European Union. For this reason special efforts need to be done to effectively respond to this phenomenon. While efforts need to be done in myriad facets of society, the present study focused on analysing the societal and ethical impact of counter terrorism policies in six European countries (Germany, Italy, the Netherlands, Romania, Spain and the United Kingdom). Specifically, it aims at contributing with insights into those elements that hinder and those that facilitate the achievement of a positive societal and ethical impact. The, communicative methodology made it possible to not only state the negative impacts, which had already been highlighted in the few previous assessments of the impact of CT policies, especially on certain social groups, but the aim of social transformation of this methodology made it possible to delve into those elements that facilitate a positive societal and ethical impact. Thus, it contributes with novel insight into how policies need to be defined and implemented in order to truly be at the service of the European people.

The framework strategy from where we departed is the so-called EU-Counter terrorism strategy (2005) and its revisions as well as the European Security Strategy (2015). This framework attempts to encompass prevention efforts in CT across the Member States (MS), yet the primary responsibility for combating terrorism lies on the MSs themselves. Almost every MS is following the recommendations of the EU, with some exceptions (for example, Italy which is in the process of developing a complete package of prevention measures) incorporating acts of terrorism in their penal codes and developing a specific strategic plan regarding the prevention of terrorism following the indications of the EU and promoting measures in different areas (schools, migration and social cohesion/ integration, religion, prisons, media, among others) and not only in the law enforcement area. This progress is crucial to tackle the presence of terrorist networks and at the same time poses challenges: to what extent do these policies – implemented in strategies, programmes and measures - respect the human rights and individual liberties and promote positive societal changes? Many institutions and organizations are speaking out about the importance to consider these impacts. For instance, the report of the Council of Europe called On Counter- Terrorism measures and human rights (2010) was elaborated to outline the most recurring issues, which have arisen regarding Counter- Terrorism policies and Human Rights. Additionally, the OSCE (2014) has also made observations in this regard, highlighting how many CT policies are ‘community-targeted’, implementing tactics such as the use of police-stop-and-search powers, which although necessary, are being carried out without a specific framework based upon the rule of law and the respect for human rights.

The analysis evidences that the risk for these policies to limit some of the fundamental human rights is a reality in these six European countries, and especially for some specifically vulnerable social groups that are being

This project has received funding from the European Union's Horizon 2020 research and innovation programme under grant agreement N° 699824.

stigmatised in the effort to prevent terrorism and contribute to a hostile environment in Europe. When thinking of terrorism in Europe, the first thought is religious terror putting other types of violent extremism in the back, whereas the increasing far right movements as the participants in this study outline are as real a threat as the former. Nonetheless, the analysis further emphasises and adds to the current state of the art the potential of these policies and specific programmes to actually make a difference in countering terrorism. While current prevention efforts evolve around preventing terrorism, the results obtained from our analysis evidence the need to specifically prevent people from falling into terrorist networks. We, as a society, need to get to the potential people falling into TN before recruiters reach them and engage them. Actions that are being employed on the grassroots level by very diverse stakeholders and institutions are responding to this need. They enforce democracy, the inclusion of all citizens in our societies and promote the civic participation of some of the most vulnerable groups –youth, ethnic minorities, as well as prison population. One of the crucial elements observed in the recruitment into terrorist networks is the exploitation of the predominant model of socialisation which portrays violence as attractive. Since very early ages the youth are exposed to violence through social media, peer interactions, videogames, and many others. Terrorist groups successfully take advantage of this socialisation to recruit vulnerable people for their purposes. A key issue manifested in the analysis conducted is that whereas most stakeholders and end-users acknowledge the importance of the link between attraction to violence to understand the mechanisms of falling for TN, only few prevention strategies particularly address this specific aspect for the CT efforts.

More research and more efforts need to be done to disentangle these mechanisms and provide greater insight into *dialogic evidence-based policies* to tackle the challenge of terrorist networks, thus including stakeholders and end-users in the design of such measures, as well as in their implementation and evaluation.

This project has received funding from the European Union's Horizon 2020 research and innovation programme under grant agreement N° 699824.

6. REFERENCES

Ahmed, S. (2015). The ‘emotionalization of the “war on terror”’: Counter-terrorism, fear, risk, insecurity and helplessness. *Criminology and criminal justice*, *15*(5), 545-560. DOI: 10.1177/1748895815572161

Alam, Y., & Husband, C. (2013). Islamophobia, community cohesion and counter-terrorism policies in Britain. *Patterns of Prejudice*, *47*(3), 235-252. DOI: 10.1080/0031322X.2013.797779

Council of the European Union. Declaration on combating terrorism, 29 March 2004. Available at: http://data.consilium.europa.eu/doc/document/ST-7906-2004-INIT/en/pdf
Council of the European Union. Evaluation of National Anti-Terrorist arrangements: Improving national machinery and capability for the fight against terrorism, 18 november 2005. Available at: http://register.consilium.europa.eu/doc/srv?l=EN&f=ST%2012168%202005%20REV%203

Eijkman, Q., Lettinga, D., & Verbossen, G. (2012). Impact of Counter-Terrorism on Communities: Netherlands Background Report. *Open Society Foundations, Institute of Strategic Dialogue*.

European Union Agency for fundamental rights *(2015) Embedding fundamental rights in the security Agenda. Available at: http://fra.europa.eu/en/publication/2015/embedding-fundamental-rights-security-agenda*

European Parliament resolution, ‘EU Counter-Terrorism Policy: main achievements and future challenges’, 14 December 2011. Available online at http://www.europarl.europa.eu/sides/getDoc.do?type=TA&reference=P7-TA-2011-0577&language=HR 103 Ibid. 104 Ibid

Flecha, R. (2014). Using Mixed Methods From a Communicative Orientation: Researching With Grassroots Roma. *Journal of Mixed Methods Research*, *8*(3), 245–254. doi:10.1177/1558689814527945

Fischbacher-Smith, D. (2016). Framing the UK's counter-terrorism policy within the context of a wicked problema. *Public money & management*, *36*(6), 399-407. DOI: 10.1080/09540962.2016.1200801

Gomez, A., Puigvert, L., & Flecha, R. (2011). Critical Communicative Methodology: Informing Real Social Transformation Through Research. *Qualitative Inquiry*, *17*(3), 235–245. doi:10.1177/1077800410397802

Guild, E. (2008). The Uses and Abuses of Counter‐Terrorism Policies in Europe: The Case of the ‘Terrorist Lists’*. *JCMS: Journal of Common Market Studies*, *46*(1), 173-193.
Guru, S. (2012). Under siege: Families of counter-terrorism. *British Journal of Social Work*, *42*(6), 1151-1173. DOI: 10.1093/bjsw/bcs089

Habermas, J. (1984). *The Theory of Communicative Action, Vol. 1: Reasons and the Rationalization of Society*. Boston: Beacon Press.

Hayes, B. Jones, C. (2013) Report on how the EU assess the impact, legitimacy and effectiveness of its counterterrorism laws. In SECILE project: Securing Europe through Counter-Terrorism – Impact, Legitimacy & Effectiveness

Hickman, M. J., Thomas, L., Silvestri, S., & Nickels, H. (2011). 'Suspect Communities'?: Counter-terrorism Policy, the Press, and the Impact on Irish and Muslim Communities in Britain. London Metropolitan University.

This project has received funding from the European Union's Horizon 2020 research and innovation programme under grant agreement N° 699824.

Isakjee, A., & Allen, C. (2013). 'A catastrophic lack of inquisitiveness': A critical study of the impact and narrative of the Project Champion surveillance project in Birmingham. *Ethnicities*, *13*(6), 751-770. DOI: 10.1177/1468796813492488

Jarvis, L., & Lister, M. (2013). Disconnected citizenship? The impacts of anti-terrorism policy on citizenship in the UK. *Political studies*, *61*(3), 656-675. DOI: 10.1111/j.1467-9248.2012.00993.x

Lennon, G. (2013). Precautionary tales: Suspicionless counter-terrorism stop and search. *Criminology and criminal justice*, *15*(1), 44-62. DOI: 10.1177/1748895813509637

Maras, M. H. (2012). The economic costs and consequences of mass communications data retention: is the data retention directive a proportionate measure?. *European Journal of Law and Economics*, *33*(2), 447-472. DOI. 10.1007/s10657-011-9245-8

Mertens, D. M., Sordé, T. D. M. (2014). Mixed Methods Research With Groups at Risk New Developments and Key Debates. *Journal of Mixed Methods Research*, *8*(3), 207–211. Doi:10.1177/1558689814527916

Open Society Foundations (2016) *Eroding Trust: The UK's Prevent Counter-Extremism Strategy in Health and Education.* New York: Open Society Foundations. Available online at: https://www.opensocietyfoundations.org/sites/default/files/eroding-trust-20161017_0.pdf

Organization for Security and Co-operation in Europe (OSCE) (2014). Preventing Terrorism and Countering Violent Extremism and Radicalization that lead to Terrorism: a community-policing approach.

Sentas, V. (2015). Policing the diaspora: Kurdish Londoners, MI5 and the proscription of terrorist organizations in the United Kingdom. *British Journal of Criminology*, *56*(5), 898-918. DOI: 10.1093/bjc/azv094

This project has received funding from the European Union's Horizon 2020 research and innovation programme under grant agreement N° 699824.

ANNEX 1. FIELDWORK STUDY PARTICIPANTS

Fieldwork study participants

Country	Institution	Profile
Germany	University not identified due to confidentiality reasons	Expert, female. Collaborates with FP projects. Expert on terrorism and radicalization
Germany	University not identified due to confidentiality reasons	Expert, male. Head of a research center related to religious issues
Germany	University not identified due to confidentiality reasons	Expert, male. Managing director of a center related to Islam and law
Germany	University not identified due to confidentiality reasons	Expert, male. Research interests related to migration, integration and intercultural conflicts
Germany	University not identified due to confidentiality reasons	Expert, male. Director of a center on police and security research
Germany	University not identified due to confidentiality reasons	Expert, male. Research interest related to Arab studies
Germany	University not identified due to confidentiality reasons	Expert, male. Expert in de-radicalization
Germany	NGO working on violence prevention	Stakeholder, male. Expert on prevention of online radicalization
Germany	Institution working in the field of de-radicalization	Stakeholder, female. Works with families on de-radicalization
Germany	NGO working on street work	Stakeholder, male. Social street worker on violence prevention
Germany	Mothers for Life Association	End-user, female. Lost her son in Syria, member of the association
Germany	Federal Ministry of Interior	Stakeholder, female.
Germany	Kompass	Misbah Arshad, female. Works on prevention of radicalization and deradicalisation in youth detention centres
Germany	Kompass	Mustafa Cimsit, male. Imam and works on prevention of radicalization and deradicalisation in youth detention centres

This project has received funding from the European Union's Horizon 2020 research and innovation programme under grant agreement N° 699824.

Germany		End-user, female. Young Muslim participated in prevention programme
Germany	Religious Association	Stakeholder, male. Representative of Muslim associations
Italy	Non profit research centre not identified due to confidentiality reasons	Expert, male. founder and senior researcher of a research center. Producer of documentaries for national and international media.
Italy	University not identified due to confidentiality reasons	Expert, male. Researcher involved in Religious Affairs.
Italy	Institute of Research at the National Research Council	Expert, male. His main research interests concern educational organizations, professional learning and educational policies.
Italy	University not identified due to confidentiality reasons	Expert, female. Her research focuses on criminal law.
Italy	University not identified due to confidentiality reasons	Expert, male. His research interests include terrorism and political violence, radicalisation, and international migration and security.
Italy	Addiopizzo	Stakeholder, male. Director. Addiopizzo is one of the most famous association in Sicily to build a community of businesses and consumers who refuse Mafia extortion money
Italy	School not identified due to confidentiality reasons	Stakeholder, female. She is an institute professor.
Italy	Association Antigone	Stakeholder, male. Work in prisons with association for rights and guarantees in the criminal system.
Netherlands	University not identified due to confidentiality reasons	Expert, female. Senior-Researcher and Lecturer at a Dutch university. Expertise in Justice, policy and Media. Formerly involved with responsibilities in Amnesty International Dutch section.
Netherlands	University not identified due to confidentiality reasons	Expert, female. Researcher involved in several European projects. Expertise in communities and neighborhoods.
Netherlands	University not identified due to confidentiality reasons	Expert, female. Her research focuses on migrations, migration control and terrorism.
Netherlands	University not identified due to confidentiality reasons	Expert, female. Researcher involved in several European projects. Expertise in criminal justice and social work.
Netherlands	Ministry of Justice and Security	Expert, male. Policy maker at Ministry of Justice and Security
Netherlands	Ministry of Justice and Security	Expert, female. Policy maker. Head of unit
Netherlands	City council	Stakeholder, female. Worker in a city council

This project has received funding from the European Union's Horizon 2020 research and innovation programme under grant agreement N° 699824.

Netherlands	Dutch National Police	Stakeholder, male. Chief of police district.
Netherlands	Dutch National Police	Stakeholder, female. Responsible of Organized Crime in a police district.
Netherlands	Dutch National Police	Stakeholder, female. Involved in the work of Trafficking on Human Beings
Netherlands	Foundation for Intercultural Participation and Integration (SIPI) in Amsterdam.	Stakeholder, female. Project manager.
Netherlands	Critical Mass Foundation	Stakeholder, female. Project leader
Netherlands	Foundation Peace Education Projects	Stakeholder, male. Responsible of projects development
Netherlands	Any specific institution	End-user, male. He was radicalised and now is de-radicalised.
Romania	University not identified due to confidentiality reasons	Expert, male. He conducts research in the area of probation and prison fields. He works with recognized agencies in relation to criminology.
Romania	University not identified due to confidentiality reasons	Expert, female. Researcher involved in international security affairs.
Romania	Media Wise Society	Stakeholder, female. Involved in media education.
Romania	Active Watch	Stakeholder, male. He works in this NGO specialized in media monitoring and interventions for combating discrimination.
Romania	Asociatia Impotriva Crimei Organizate si Consiliere Antidrog	Stakeholder, male, neighborhood. This association works to combat violence at the national level.
Romania	Fundatia de Sprijin Comunitar	Stakeholder, male. Responsible of projects. The foundation supports disadvantaged groups of community and development of the local communities.
Romania	Association for Dialogue, Employment and Migration CONECT	Stakeholder, male. President of the association.
Romania	Ofensiva Tinerilor	Stakeholder, male. Member of this association that works with young people belonging to different ethnic groups. He is also parliamentary advisor at the Romanian Parliament.
Romania	Asociatia Eliberare	Stakeholder, female. Director of this association working on human trafficking.
Spain	University not identified due to confidentiality reasons	Expert, male. Researcher in a group on public policies

This project has received funding from the European Union's Horizon 2020 research and innovation programme under grant agreement N° 699824.

Spain	University not identified due to confidentiality reasons	Expert, male. Part of the Advisory Council for Religious Diversity of a regional government
Spain	Catalan government/Regional government not identified due to confidentiality reasons	Expert, female. Head of security of the penitentiary services of the government
Spain	University not identified due to confidentiality reasons	Expert, male. Has worked on racism, anti-Semitism and jihadism issues
Spain	University not identified due to confidentiality reasons	Expert, female. Member of an observatory on human rights and the penal system
Spain	Catalan government/regional government not identified due to confidentiality reasons	Expert, male. Head of Commission for the Attention to the Family and the Community in the Catalan Department of Education
Spain	Catalan government/regional government not identified due to confidentiality reasons	Expert, male. Field of rehabilitation of penitentiary services of the government
Spain	Regional TV	Stakeholder, male. Vice-president of the table for diversity of the TV program
Spain	Catalan primary school	End-user/female. Director of a primary school in Spain organized as a Learning Community, located in a deprived neighborhood.
Spain	Catalan primary school	End-user/female. Teacher of a primary school in Spain organized as a Learning Community, located in a deprived neighborhood.
Spain	Catalan primary school	End-user/female. Teacher of a primary school in Spain organized as a Learning Community, located in a deprived neighborhood.
Spain	Jewish synagogue	End-user/male. Rabbi from a Jewish synagogue in Barcelona
Spain	Mosque	End-user/male. Migrant, Imam of a Spanish mosque.
Spain	Roma Association	End-user/male. Roma, community leader
Spain	Roma activist	End-user/male. Roma, community leader
Spain	Pakistani Neighboorhood Association	End-user/male. Migrant, recognized community leader
Spain	Badalona Neighboorhood Association	End-user/male. Activist. President of the Neighborhood Association

This project has received funding from the European Union's Horizon 2020 research and innovation programme under grant agreement N° 699824.

Spain	Department of Social Affairs of Badalona (Consorci Badalona Sud)	End-user/male. Worker in the Department of Social Affairs of Badalona (Consorci Badalona Sud)
Spain	Women's association	End-user/female. Member of a Women's association from Badalona
Spain	Juvenile Detention Center	Stakeholder/female. Social worker and teacher in a Catalan Juvenile Detention Center
Spain	Juvenile Detention Center	Stakeholder/female. Responsible of the Education in a Catalan Juvenile Detention Center
Spain	Unit for Child and Teenagers Attention, Barcelona City Council	Stakeholder/female. Professional from the Unit for Child and Teenagers Attention, Barcelona City Council].
Spain	University	End-user, male. Community leader collaborating with integration issues
Spain	Muslim Students Association	End-user, female. Muslim student, member of the Association of Muslim Students in Tarragona
Spain	Cooperative of Senegalese Workers	End-user, male. Sub-Saharan member of the DIOMCOOP Cooperative of Senegalese Workers
Spain	Neighbor	End-user, female. Muslim mother, volunteer at a Learning Community
Spain	Migrants association	End-user, male. Pakistani senior community leader from the city of Badalona
United Kingdom	Social Platform on Cities and Social Cohesion	Expert, male. Partner of the Social Platform on Cities and Social Cohesion. Expertise in the neighbourhood domain.
United Kingdom	University not identified due to confidentiality reasons	Expert, male. Head of the Department of Social Science. Expertise in religious affairs and attitudes of and towards ethno-religious minorities.
United Kingdom	University not identified due to confidentiality reasons	Expert, male. International expert in human rights and Muslim studies. Partner of the TOLERANCE Project. Integration of Ethnic Minorities
United Kingdom	University not identified due to confidentiality reasons	Expert, male. Senior Lecturer in Criminology & Criminal Justice. Expertise in prisons.
United Kingdom	University not identified due to confidentiality reasons	Expert, female. Senior Lecturer. Expertise in Education, Communication and Language.
United Kingdom	University not identified due to confidentiality reasons	Expert, male. Lecturer in Psychology. Expertise in Countering radicalisation.
United Kingdom	Active Change Foundation (ACF)	Stakeholder, male. Chief Executive ACF. Expertise in the neighbourhood domain.
United Kingdom	Primary School	Stakeholder, male. Professor at primary school. Interested in the CT strategy in the educational spectrum.

This project has received funding from the European Union's Horizon 2020 research and innovation programme under grant agreement N° 699824.

United Kingdom	Cambridgeshire Race Equality & Diversity Service (CREDS)	Stakeholder, female. Worker at the Race Equality and Diversity Service.
United Kingdom	St Giles Trust organization	Stakeholder, male. Expertise in organized crime. Experiences of prison, homelessness, unemployment, addiction.
United Kingdom	Safer London	Stakeholder, female. Expertise in organized crime. Experiences in youth, gangs, exploitation and crime.
United Kingdom	The Roma Project	Stakeholder, male. Director of the project.
United Kingdom	The Roma Project	Stakeholder, male. Member of the Board.
United Kingdom	Race Equality & Diversity Service (CREDS)	Stakeholder, female. Social worker.
United Kingdom	Race Equality & Diversity Service (CREDS)	Stakeholder, male. Worker
United Kingdom	Race Equality & Diversity Service (CREDS)	End-user, female. Muslim woman
United Kingdom	Prevent Safeguarding Team	Stakeholder, female. Lead trainer for Prevent in schools

This project has received funding from the European Union's Horizon 2020 research and innovation programme under grant agreement N° 699824.

ANNEX 2. GUIDELINES FOR QUALITATIVE FIELDWORK

QUESTIONNAIRE GERMANY

TERRORISM

INTRODUCTION: Considering European legislation against terrorism, and the current waves of terrorist acts, which have led to the implementation of measures and policies in many EU Member states. The aim of the interview is to go further in the implementation of measures and programs against terrorism in Germany and measure their impact. Terrorism is a recurrent issue in the European Security Strategy and one of the main priorities of the Union in the field of freedom, security and justice. Several EU documents deal with the prevention of radicalization and recruitment to terrorism, such as the EU Strategy for combating radicalization and recruitment to Terrorism of the Council of the European Union (5643/5/14) or the COM (2013)941 final, Preventing Radicalization to Terrorism and Violent Extremism: strengthening the EU's Response. This last communication affirms that several Member States have already implemented measures to prevent radicalization, both internally and externally.

In the case of Germany, the most recent and important measure we have knowledge about is the:

Strategie der Bundesregierung zur Extremismusprävention und Demokratieförderung, One of the aims of the strategy is the prevention of extremism including measures that prevent and counteract the order of values of the Basic Law and the democratic constitutional state. Preventive measures are aimed at **endangered people or groups, their environment and their networks, and, where appropriate, potential perpetrators**, in order to prevent and to interrupt (violent) actions.

While another measure of the strategy in 2004 was the establishment of the **GTAZ (Joint Counter-Terrorism Centre)**, which is not an autonomous authority but a joint co-operation and communication platform used by 40 **internal security agencies**.

1. **National tools used in the fight against terrorism in Germany (in particular about prevention of radicalization)**

- Do you have knowledge about any other policy or measure that is being applied in Germany regarding the prevention of radicalization or recruitment in general? (For example, the EU have recommended

This project has received funding from the European Union's Horizon 2020 research and innovation programme under grant agreement N° 699824.

that the EU Member states apply protocols to detect radicalization in schools/ prisons; or tools to counteract on-line terrorist propaganda and hate speech)

- And in particular regarding the areas of? :
  - Media
  - Religion
  - Neighbourhoods and community development
  - Prisons
  - Education
  - Migration
- Is there any other measure of the aforementioned areas not related to TN in particular, but which could have an effect to? For example, the EU have recommended that the EU Member states enhance social inclusion trough education and non-formal learning or trough renovating plans for deprived neighbourhoods.
- Which is the main strategy followed by the police in Germany? We have knowledge that after the attacks against the United States on 11 September 2001, the German police decided to identify individuals on the basis of demographic and socio-economic criteria derived from the profile of the 9/11 terrorists, namely: being male, Muslim, aged between 18 and 40, a student or former student, and a native or national of a specified country with a predominantly Muslim population. As a result, data on up to 300,000 individuals was screened, and a total of 32,000 people were identified as "sleepers" and subject to closer scrutiny. However, no one was ever charged with a criminal offence (OSCE Report, 2014).

- Do the prevent strategy in Germany provide training to the agencies in charge of identifying early-stage radicalization? *According to the lit review: Fischbacher-Smith (2016) stresses that the current policy puts the responsibilities for identifying early-stage radicalization on organizations for whom it is not their primary concern (and for which they are ill-equipped), and there is an insufficient research evidence base available to them to show how effective the various forms of intervention might be.*

2. Impact of the measures/policies in the fight against terrorism

- Does the strategy have received comments of disagreement? Or controversial discussions? (In the affirmative case, what kind of comments or discussions?) *According to the lit review in prevention measures: Alam & Husband (2013) affirms that the introduction of counter-terrorism policy in the form of PREVENT was met with a wide range of strong resistance. Many councillors responsible for the implementation of this policy saw it as discriminatory in its unambiguous targeting of Muslim communities. At the same time, Muslims saw PREVENT as an assault on their integrity as law-abiding*

This project has received funding from the European Union's Horizon 2020 research and innovation programme under grant agreement N° 699824.

citizens. The introduction of pre-crime counter-terrorism legislation marks the intrusion of the construction of the enemy with anger, disgust; fear and risk (see McCulloch and Pickering, (2009)).

- Does the strategy foresee tools or mechanisms to evaluate the impact? (In the affirmative case: how is the impact measured?; how are these tools applied?...)
- To what extent is the strategy affecting fundamental rights?

- Does the strategic plan affect the right to life privacy (including their home and communications)? *The literature reviews that CT measures foresee special powers of secret surveillance of citizens/ special search and seizure powers.*

- Does some measures against CT involve the processing of personal data?
  - Do you know who is responsible for processing personal data?
  - Is the security of data processing activities provided for from a technical and organisational point of view?
  - Are any safeguards which render the interference into the right of data protection proportionate and necessary provided for?

- How does the strategic plan entail any different treatment of groups or individuals directly on grounds of racial or ethnic origin? Or could it lead to indirect discrimination? *The lit review affirms that some preventive measures such as Stop and Search or excessive patrolling in some regions can lead to discrimination and the creation of suspect communities. On the other hand, according to McCulloch and Pickering (2009) high levels of police discretion increase the risk of the arbitrary exercise of power and may 'lead to intensified politicization of policing and law.*

<u>ORGANISED CRIME</u>

INTRODUCTION: The EU has been elaborating action plans, programs and strategies either targeting organised crime in general or dealing with its particular forms. The problem has been addressed within the EU's foreign policy and accession strategy, but most of the relevant actions have been undertaken as part of police and judicial cooperation in criminal matters (EU Response to Organised Crime, 2013). OC is also a priority in the European Agenda on Security. However, the main achievements followed by this strategy are anti-money laundering packages such as financial intelligence units, asset recovery offices, among others.

This project has received funding from the European Union's Horizon 2020 research and innovation programme under grant agreement N° 699824.

Nevertheless, Germany does not include Organized crime as one of the priorities in the *White Paper on German security policy and the future of the Bundeswehr.*

Germany has transposed the **Council Framework decision 2008/841/JHA**. In Germany, the jurisprudence has consistently applied a restrictive interpretation of **Article 129 of the Criminal Code on criminal organization**. In particular, it required that the members of the association pursue a common goal and feel part of a common union, where the individual will is submitted to the common will of the group.

Some other controversies regarding this FD 2008/841/JHA are the following:

- The use of **special investigative means** inherently carries a potential risk for abuse. Few are the European states that have not been affected by a nationwide scandal involving leakage of information collected through covert techniques. For example, **the government Trojan horse controversy in Germany** (Study on paving the way for future policy initiatives in the field of fight against organised crime: the effectiveness of specific criminal law measures targeting organised crime, Final Report, 2015. European Commission).
  - Do you think that these measures are applied indiscriminately? Or mainly to specific communities?

- **Do you have knowledge about any other policy or measure that is being applied in Germany regarding the prevention of organised crime groups?**
- **And in particular regarding the areas of? :**
  - Media
  - Religion
  - Neighbourhoods and community development
  - Prisons
  - Education
  - Migration
- **Is there any other measure of the aforementioned areas not related to OC in particular, but which could have an effect to?** For example, renovating plans for deprived neighbourhoods.
- **Does the strategy foresee tools or mechanisms to evaluate the impact? (In the affirmative case: how is the impact measured?; how are these tools applied?...)**

This project has received funding from the European Union's Horizon 2020 research and innovation programme under grant agreement N° 699824.

2. National specialist legal and investigative tools

Information about the implementation of legal and investigative tools used in the fight against organised crime. For instance: surveillance, interception of communication, covert investigations, controlled deliveries, informants, among others.
Perception about:

- Groups against which tools are most used
- Impact → whether the national legislation foresees tools to measure the impact
- Possible ways to improve each tool in order to achieve greater impact

3. Impact of the measures/policies in the fight against organised crime

- Do the last measures/ policies regarding OC receive comments of disagreement? Or controversial discussions? (In the affirmative case, what kind of comments or discussions?)
- Do the measures in OC foresee tools or mechanisms to evaluate the impact? (In the affirmative case: how is the impact measured? how are these tools applied?...)
- To what extent are the measures against Organised Crime affecting fundamental rights? (For example, wiretapping, protective custody, confiscation of mafia assets.

This project has received funding from the European Union's Horizon 2020 research and innovation programme under grant agreement N° 699824.

QUESTIONNAIRE ITALY

INTRODUCTION: Considering European legislation against terrorism, and the current waves of terrorist acts, which have led to the implementation of measures and policies in many EU Member states. The aim of the interview is to go further in the implementation of measures against terrorism in Italy. Terrorism is a recurrent issue in the European Security Strategy and one of the main priorities of the Union in the field of freedom, security and justice. Several EU documents deal with the prevention of radicalization and recruitment to terrorism, such as the EU Strategy for combating radicalization and recruitment to Terrorism of the Council of the European Union (5643/5/14) or the COM (2013)941 final, Preventing Radicalization to Terrorism and Violent Extremism: strengthening the EU's Response. This last communication affirms that several Member States have already implemented measures to prevent radicalization, both internally and externally. However, comprehensive approaches under the strand of the EU CT Strategy aimed at addressing radicalization and recruitment are not widely used.

In the case of Italy, the most recent and important measure we have knowledge about is the: **Decreto-legge 18 febbraio 2015, n. 7,** which contains urgent measures for counter-terrorism. This decree establishes changes in the penal code such as including the act of travelling to foreign territory as well as the increase of the penalty for many terrorist acts. Moreover, other changes are included such as the preservation of the acquired data and telematic traffic, when they are indispensable for the continuation of the activity aimed at the prevention, or the increase in expenses for the protection of national interests (in particular, for the upgrading of the aeronautical surveillance equipment and security in the central Mediterranean), among others.

Previous to the *decreto legge of 2015*, there is also **the Law no. 155 "Urgent measures to fight international terrorism"** of 2005**.** However, the aforementioned decrees are focused mainly in penal and procedural changes, or the protection of critical facilities.

1. **National tools used in the fight against terrorism in Italy (in particular about prevention of radicalization)**

   1.1. **Do you have knowledge about any other policy or measure that is being applied in Italy regarding the prevention of radicalization or recruitment**

This project has received funding from the European Union's Horizon 2020 research and innovation programme under grant agreement N° 699824.

in general? (For example, the EU have recommended that the EU Member states apply protocols to detect radicalization in schools/ prisons; or tools to counteract on-line terrorist propaganda and hate speech)

1.2. And in particular regarding the areas of…?

- Media
- Religion
- Neighborhoods and community development
- Prisons
- Education
- Migration

1.3. Is there any other measure of the aforementioned areas not related to TN in particular, but which could have an effect to? For example, the EU have recommended that the EU Member states enhance social inclusion through education and non-formal learning or through renovating plans for deprived neighborhoods.

2. Impact of the measures/policies in the fight against terrorism

2.1. Do the last Decreto-legge 18 febbraio 2015, n. 7, which contains urgent measures for the counter-terrorism, have received comments of disagreement? Or controversial discussions? (In the affirmative case, what kind of comments or discussions?)

2.2. Do the new measures of the last Decreto-legge 18 febbraio 2015 foresee tools or mechanisms to evaluate the impact? (In the affirmative case: how is the impact measured? How are these tools applied?...)

2.3. To what extent is the last Decreto-legge 18 febbraio 2015 affecting fundamental rights?

2.4. Do the last Decreto-legge 18 febbraio 2015 have beneficial and negative impacts (Conflict of interests)? Which are these rights in conflict?

2.5. Does the last Decreto-legge 18 febbraio 2015 make the public better informed about radicalization/ recruitment in terrorist groups?

2.6. Does the last Decreto-legge 18 febbraio 2015 affect the public's access to information?

2.7. Does the last Decreto-legge 18 febbraio 2015 affect political parties or civic organisations?

2.8. Is the last Decreto-legge 18 febbraio 2015 affecting on particular risks groups? (social

This project has received funding from the European Union's Horizon 2020 research and innovation programme under grant agreement N° 699824.

groups, mobility, region…)

ORGANISED CRIME

INTRODUCTION: The EU has been elaborating action plans, programs and strategies either targeting organised crime in general or dealing with its particular forms. The problem has been addressed within the EU's foreign policy and accession strategy, but most of the relevant actions have been undertaken as part of police and judicial cooperation in criminal matters (EU Response to Organised Crime, 2013). OC is also a priority in the European Agenda on Security. However, the main achievements followed by this strategy are anti-money laundering packages such as financial intelligence units, asset recovery offices, among others.

Italy does not have a National Security Strategy in the strict sense of the word. However, there are some measures in this sense such as the art. 146 of the Italian Penal Code; some preventive measures such as wiretapping, protective custody, confiscation of mafia assets (Law 646 (1982)), special penalties and prison regimes for persons convicted for Mafia style crimes (the offence of 'mafia-type association' (Article 416 bis c.c.); and the 'hard prison regime' (Article 41 bis p.a.a.); but also some indirect policies such as the **security for development 2007-2013**.

Finally, the EU is enforcing *special legal and investigative tools* such as surveillance; interception of communication; covert investigations; controlled deliveries; informants, among others. However, once more these measures are not specifically focused in the prevention of recruitment in organized crime groups.

1. National tools used in the fight against organised crime

The aim is to gather information about:

- The tools used in the fight against organised crime, we can talk about the transposing of the Framework Decision 2008/841/JHA. However, this information has been analysed in several occasions.
- Other relevant national laws/measures.
- Perceived barriers and facilitators to the implementation of the aforementioned tools
- Frequency of the use of national legislative provisions, their use in practice, and their impact.

1.1. Do you have knowledge about any other policy or measure that is being applied in

This project has received funding from the European Union's Horizon 2020 research and innovation programme under grant agreement N° 699824.

Italy regarding the prevention of organised crime groups?

1.2. And in particular regarding the areas of? :

- Media
- Religion
- Neighborhoods and community development
- Prisons
- Education
- Migration

1.3. Is there any other measure of the aforementioned areas not related to OC in particular, but which could have an effect to? For example, renovating plans for deprived neighbourhoods.

2. National specialist legal and investigative tools

Information about the implementation of legal and investigative tools used in the fight against organised crime. For instance: surveillance, interception of communication, covert investigations, controlled deliveries, informants, among others.
Perception about:

- Groups against which tools are most used
- Impact whether the national legislation foresees tools to measure the impact
- Possible ways to improve each tool in order to achieve greater impact

3. Impact of the measures/policies in the fight against organised crime

- Do the last measures/ policies regarding OC receive comments of disagreement? Or controversial discussions? (In the affirmative case, what kind of comments or discussions?)
- Do the measures in OC foresee tools or mechanisms to evaluate the impact? (In the affirmative case: how is the impact measured? how are these tools applied?...)
- To what extent are the measures against Organised Crime affecting fundamental rights? (for example, the art. 146 of the Italian Penal Code; some preventive measures such as wiretapping, protective custody, confiscation of mafia assets (Law 646 (1982)), offence of 'mafia-type association' (Article 416 bis c.c.); or the 'hard prison regime' (Article

This project has received funding from the European Union's Horizon 2020 research and innovation programme under grant agreement N° 699824.

41 bis p.a.a.).

- To what extent are the measures against organised crime aforementioned before have social impact?
  - Does the measure impact on poverty rates? Or severe material deprivation?
  - Does the measure impact on cultural diversity? Are all actors treated on equal footing? Are there specific effects on particular risk groups?
  - Does the measure affect the right to take collective action?
  - Do the policies improve security? Do the policies impact on crime rates?
  - Do the policies impact more on a specific type of crime than others?

This project has received funding from the European Union's Horizon 2020 research and innovation programme under grant agreement N° 699824.

QUESTIONNAIRE NETHERLANDS

EXPERT IN MIGRATION AND HUMAN RIGHTS [TERRORISM]

INTRODUCTION: Considering European legislation against terrorism, and the current waves of terrorist acts, which have led to the implementation of measures and policies in many EU Member states. The aim of the interview is to go further in the implementation of measures and programs against terrorism in The Netherlands and measure their impact. Terrorism is a recurrent issue in the European Security Strategy and one of the main priorities of the Union in the field of freedom, security and justice. Several EU documents deal with the prevention of radicalization and recruitment to terrorism, such as the EU Strategy for combating radicalization and recruitment to Terrorism of the Council of the European Union (5643/5/14) or the COM (2013)941 final, Preventing Radicalization to Terrorism and Violent Extremism: strengthening the EU's Response. This last communication affirms that several Member States have already implemented measures to prevent radicalization, both internally and externally.

In the case of The Netherlands, the most recent and important protocol we have knowledge about is the:

The Netherlands comprehensive action programme to combat jihadism: overview of measures and actions.

1. **IMPACT OF THE MEASURES/POLICIES IN THE FIGHT AGAINST TERRORISM IN THE FIELD OF MIGRATION**

In the last months, due to the increase of refugees in many EU States the Schengen Area has been modified. For example, there have been an increase in the police checks inside E.U. Member States. As you stress in your paper called SEARCHING FOR ''ILLEGAL'' JUNK IN THE TRUNK: UNDERLYING INTENTIONS OF (CR)IMMIGRATION CONTROLS IN SCHENGEN'S INTERNAL BORDER AREAS: *This is likely to cause new tensions within states, as the already heightened concerns about terrorism by Islamic State sympathizers is giving rise to increased identity controls on Muslims.*

Crimmigration, the growing merger of crime control and immigration control. As far as Europe is concerned, the debate has usually focused on securitization at the level of political and policy discourses. In most instances the focus is on criminalization rather than on crime and concrete

This project has received funding from the European Union's Horizon 2020 research and innovation programme under grant agreement N° 699824.

attempts to deal with it, which is unsatisfactory from the perspective of criminology as a discipline (Killias, 2011).

1.1. ETHICAL IMPACT (Fundamental Rights Impact)

- In general, what is your opinion about the strategy in terms of fundamental rights affected by these measures (database- register; profiling stop and search; alleged terrorists, etc.)
- Do you think that the application of these measures is promoting the social effect of suspect-communities?
- Did these measures (related to border control and crimmigration) receive comments of disagreement? Or controversial discussions? (In the affirmative case, what kind of comments or discussions?)
- Do the measures foresee tools to evaluate the impact? (In the affirmative case: how is the impact measure? How are these tools applied?...)
- To what extent is the strategy affecting fundamental rights?
- Do the strategy have beneficial and negative impacts (Conflict of interests)? Which are these rights in conflict?
- Do the prevent strategy in The Netherlands provide training to the agencies in charge of border control? *According to the lit review: Fischbacher-Smith (2016) stresses that the current policy puts the responsibilities for identifying early-stage radicalization on organizations for whom it is not their primary concern (and for which they are ill-equipped), and there is an insufficient research evidence base available to them to show how effective the various forms of intervention might be.*
- To what extent, are communities considered in the strategy?
- Did the government take into account the opinion (not only of minorities or organizations, but also of experts)?
- Effectivity of the plan → does the strategy have a real effect on terrorism risks?
- Perception of the police or other official agents

1.2. <u>SOCIETAL IMPACT.</u> Preventive measures should enforce not only security measures such as stop and search, but also a prevention from a more comprehensive perspective. For example, measures which enforce the social integration trough employment or education programs. Do you know if the government is working on these kind of measures?

  - Employment: new jobs created, labour market mobility…
  - Improvement of working conditions

This project has received funding from the European Union's Horizon 2020 research and innovation programme under grant agreement N° 699824.

- Vocational training/ education
- Distribution of incomes/ social protection schemes
- Participation of the communities
- Public health

Role that play the media:

- If media content fuels the crimmigration process. Examples

This project has received funding from the European Union's Horizon 2020 research and innovation programme under grant agreement N° 699824.

QUESTIONNAIRE ROMANIA

INTRODUCTION: Considering European legislation against terrorism, and the current waves of terrorist acts, which have led to the implementation of measures and policies in many EU Member states. The aim of the interview is to go further in the implementation of measures against terrorism in Romania. Terrorism is a recurrent issue in the European Security Strategy and one of the main priorities of the Union in the field of freedom, security and justice. Several EU documents deal with the prevention of radicalization and recruitment to terrorism, such as the EU Strategy for combating radicalization and recruitment to Terrorism of the Council of the European Union (5643/5/14) or the COM (2013)941 final, Preventing Radicalization to Terrorism and Violent Extremism: strengthening the EU's Response. This last communication affirms that several Member States have already implemented measures to prevent radicalization, both internally and externally. However, comprehensive approaches under the strand of the EU CT Strategy aimed at addressing radicalization and recruitment are not widely used.

In the case of Romania, the main law contributing to the coordination of the implementation of the agreements and arrangements related to preventing and combating terrorism are:

Law no. 535 / 2004 on preventing and combating terrorism. According to the provisions of art. 6, "the prevention and suppression of terrorism is organized and conducted in a unitary manner". The cooperation between national competent authorities is conducted within the National System for Preventing and Countering Terrorism. However, the Romanian Intelligence Service is the national authority in the field of countering terrorism that technically coordinates the cooperation between 20 public authorities and institutions within the System. The approval of the lists containing persons suspected of committing or financing terrorist acts is made through Government Decision (information retrieved from the answer to the questionnaire on the code of conduct on politico-military aspects of security FSC (DEC/2/09) 2016)**.**

The National Strategy on Preventing and Countering Terrorism (2002): the Strategy identifies the main aspects of the terrorist phenomenon, as a threat to Romania, defines the objectives for preventing and countering terrorism and establishes the main directions for the implementation of the National System for Preventing and Countering Terrorism.

This project has received funding from the European Union's Horizon 2020 research and innovation programme under grant agreement N° 699824.

There are further measures and instruments in the field of financing terrorism for instance, the *Order no 9 /2005 of the President of the Romanian Financial Supervisory Authority on the approval of the Instructions no 4 / 2005 on preventing the financing of terrorist acts; Government Emergency Ordinance no 135 / 2005 on the amendment of the Law no 656 / 2002 on preventing and sanctioning money laundering and on measures to prevent and combat financing terrorist acts*, among others.

2. National tools used in the fight against terrorism in Romania (in particular about prevention of radicalization)

1.4. Do you have knowledge about any other policy or measure that is being applied in Romania regarding the prevention of radicalization or recruitment in general? (For example, the EU have recommended that the EU Member states apply protocols to detect radicalization in schools/ prisons; or tools to counteract on-line terrorist propaganda and hate speech. However, in the national plan and in the Law 535/2004 these areas are not considered).

1.5. And in particular regarding the areas of? :

- Media
- Religion
- Neighborhoods and community development
- Prisons
- Education
- Migration

1.6. Is there any other measure of the aforementioned areas not related to TN in particular, but which could have an effect to? For example, the EU have recommended that the EU Member states enhance social inclusion through education and non-formal learning or through renovating plans for deprived neighborhoods.

2. Impact of the measures/policies in the fight against terrorism

This project has received funding from the European Union's Horizon 2020 research and innovation programme under grant agreement N° 699824.

2.1. Do the Law no. 535 / 2004 or/ and the National Strategy received comments of disagreement? Or controversial discussions? (In the affirmative case, what kind of comments or discussions?)

2.9. Do the new measures of the Law no. 535 / 2004 or/ and the National Strategy foresee tools or mechanisms to evaluate the impact? (In the affirmative case: how is the impact measured? How are these tools applied?...)

2.10. To what extent is the Law no. 535 / 2004 or/ and the National Strategy affecting fundamental rights?

2.11. Do the Law no. 535 / 2004 or/ and the National Strategy have beneficial and negative impacts (Conflict of interests)? Which are these rights in conflict?

2.12. Do the Law no. 535 / 2004 or/and the National Strategy make the public better informed about radicalization/ recruitment in terrorist groups?

2.13. Do the Law no. 535 / 2004 or/and the National Strategy affect the public's access to information?

2.14. Do the Law no. 535 / 2004 and/ or the National Strategy affect political parties or civic organisations?

2.15. Are the Law no. 535 / 2004 or/ and the National Strategy affecting on particular risks groups? (social groups, mobility, region…)

ORGANISED CRIME

INTRODUCTION: The EU has been elaborating action plans, programs and strategies either targeting organised crime in general or dealing with its particular forms. The problem has been addressed within the EU's foreign policy and accession strategy, but most of the relevant actions have been undertaken as part of police and judicial cooperation in criminal matters (EU Response to Organised Crime, 2013). OC is also a priority in the European Agenda on Security. However, the main achievements followed by this strategy are anti-money laundering packages such as financial intelligence units, asset recovery offices, among others.

Romania has a National Defense Strategy (2015-2019) in the strict sense of the word, where organized crime groups are one of the priorities. However, the measures to combat this kind of criminal groups are not written in detail.

This project has received funding from the European Union's Horizon 2020 research and innovation programme under grant agreement N° 699824.

Finally, the EU is enforcing *special legal and investigative tools* such as surveillance; interception of communication; covert investigations; controlled deliveries; informants, among others. However, once more these measures are not specified in the national strategic plans.

4. National tools used in the fight against organised crime

The aim is to gather information about:

- The tools used in the fight against organised crime, we can talk about the transposing of the Framework Decision 2008/841/JHA. However, this information has been analysed in several occasions.
- Other relevant national laws/measures.
- Perceived barriers and facilitators to the implementation of the aforementioned tools
- Frequency of the use of national legislative provisions, their use in practice, and their impact.

1.4. Do you have knowledge about any other policy or measure that is being applied in Romania regarding the prevention of organised crime groups?

1.5. And in particular regarding the areas of? :

- Media
- Religion
- Neighborhoods and community development
- Prisons
- Education
- Migration

1.6. Is there any other measure of the aforementioned areas not related to OC in particular, but which could have an effect to? For example, renovating plans for deprived neighbourhoods.

5. National specialist legal and investigative tools

Information about the implementation of legal and investigative tools used in the fight against organised crime. For instance: surveillance, interception of communication, covert investigations, controlled deliveries, informants, among others.
Perception about:

- Groups against which tools are most used
- Impact whether the national legislation foresees tools to measure the impact
- Possible ways to improve each tool in order to achieve greater impact

6. Impact of the measures/policies in the fight against organised crime

- Do the last measures/ policies regarding OC receive comments of disagreement? Or controversial discussions? (In the affirmative case, what kind of comments or

This project has received funding from the European Union's Horizon 2020 research and innovation programme under grant agreement N° 699824.

discussions?)

- Do the measures in OC foresee tools or mechanisms to evaluate the impact? (In the affirmative case: how is the impact measured? how are these tools applied?...)
- To what extent are the measures against Organised Crime affecting fundamental rights?
- To what extent are the measures against organised crime aforementioned before have social impact?
    - Does the measure impact on poverty rates? Or severe material deprivation?
    - Does the measure impact on cultural diversity? Are all actors treated on equal footing? Are there specific effects on particular risk groups?
    - Does the measure affect the right to take collective action?
    - Do the policies improve security? Do the policies impact on crime rates?
    - Do the policies impact more on a specific type of crime than others?

This project has received funding from the European Union's Horizon 2020 research and innovation programme under grant agreement N° 699824.

ENTREVISTA ESPAÑA : EXPERTO RELIGIÓN

TERRORISMO

INTRODUCCIÓN: considerando la legislación de la Unión Europea contra el terrorismo y las atentados terroristas de los últimos meses, los cuales han llevado a la implementación de medidas y políticas de emergencia en muchos países miembro de la UE. El objetivo de la entrevista es profundizar en el efecto que han tenido estas medidas y políticas en España. El terrorismo es un tema recurrente en la Agenda Europea de Seguridad y también uno de las principales prioridades de la UE en el área de libertad, seguridad y justicia. Muchos documentos de la UE intentan dar pautas para la prevención del terrorismo como sería el caso de la Estrategia de la UE para combatir la radicalización y el reclutamiento para el terrorismo del Consejo de la UE (5643/5/14) o la COM (2013)941 Previniendo la radicalización al terrorismo y al extremismo violento: reforzando la estrategia de la UE.

En el caso de España, las medidas/estrategias sobre las que tenemos conocimiento son las siguientes:

- **Plan estratégico nacional de lucha contra la radicalización violenta. (PEN-LCRV). "Un marco para el respeto y el entendimiento común".** Este plan, siguiendo el modelo de la UE contiene 3 áreas:
    - **Prevenir (antes):** destinado a asegurar la integración y la convivencia social, especialmente de las comunidades o colectivos más vulnerables o en situación de riesgo, interviniendo desde la detección de situaciones de falta de integración social o conflictividad, tratando de impedir procesos de radicalización violenta.
    - **Vigilar (durante):** funciones de observación, vigilancia y tratamiento.
    - **Actuar (después):** seguir a comunidades/ colectivos o individuos en los que ya se han producido los procesos de radicalización violenta.

En el área de prevenir del plan se especifica que:

- En el marco de la educación y la sensibilización social: se asegurará por parte de la Administración el conocimiento de la formación y educación de los diferentes actores educativos, en materias directamente relacionadas con la problemática tratada, incluyendo a grupos vulnerables o en riesgo de radicalización violenta. **Se regulará la**

This project has received funding from the European Union's Horizon 2020 research and innovation programme under grant agreement N° 699824.

formación específica de formadores en aquellas cuestiones ideológicas, culturales o religiosas especialmente conflictivas, garantizándose el conocimiento del marco constitucional español y las iniciativas especificadas sobre integración, exclusión y segregación social y radicalización violenta en el "área de prevenir".

El plan también especifica que deberían incorporarse en el plan comunidades religiosas:

- Se trata de Incorporar al seno del GN-LCRV no sólo a personas, organismos e instituciones relevantes o con influencia en la comunidad, sino también a otros actores potenciales del sector privado, como organizaciones no gubernamentales, seguridad privada, personal académico, docente y educativo, **religioso**, sanitario y de asistencia social, entre otros.

¿Tiene conocimiento que las estrategias/ medidas que se prevén en el plan se están llevando a cabo? ¿El plan prevé mecanismos de evaluación de impacto en temas de religión?

¿Hay medidas/ legislación concreta en temas de religión que se esté usando para la prevención de la radicalización, aunque ese no sea su objetivo principal?

¿Ha recibido comentarios de desacuerdo el plan estratégico en temas de religión? Según la literatura científica, la estrategia de Prevenir produce el estigma de ciertas comunidades religiosas y ha recibida numerosas críticas.

¿Se tuvo en cuenta el diálogo con diferentes comunidades religiosas para crear el protocolo?

¿Tiene conocimiento si se está incluyendo a las comunidades religiosas actualmente en temas de protocolos anti-radicalización?

¿Hay evidencia que el programa está trabajando para que haya una mejor información sobre procesos de radicalización? ¿Y sobre la confusión entre religión islámica y terrorismo islamista?

1. IMPACTO ÉTICO (Impacto en los derechos humanos)

1.1. General

This project has received funding from the European Union's Horizon 2020 research and innovation programme under grant agreement N° 699824.

- ¿El plan estratégico contra el terrorismo está afectando los derechos individuales de las personas que pertenecen a comunidades religiosas?
- ¿El plan estratégico tiene impactos negativos y a la vez también positivos? ¿Cuáles son los derechos en disputa? Por ejemplo, la literatura científica indica que las políticas anti-terroristas presentan la disputa entre privacidad vs seguridad.

1.2. Libertad de expresión y de consciencia

- ¿El plan estratégico está afectando la libertad de pensamiento, conciencia o religión?
- ¿Está el plan estratégico afectando la libertad de expresión e información? ¿y de asamblea y asociación?

1.3. Igualdad de género, de oportunidades, de no discriminación.

- ¿Los planes estratégicos están dando un trato diferente a determinados grupos o individuos debido a su orientación religiosa?

1.4. Datos personales

- ¿Tiene conocimiento de si se están reteniendo datos personales de sospechosos por “terrorismo” según su participación en actos/ comunidades religiosas? ¿hay garantías? ¿se tienen en cuenta mecanismos de revisión y supervisión?

2. IMPACTO SOCIAL

2.1. Gobernanza y participación

- ¿El programa estratégico tiene en cuenta organizaciones sociales o entidades religiosas?
- ¿hay un trato diferente dependiendo de si las organizaciones o entidades tienen vínculos religiosos?
- ¿El plan promueve una mejor información sobre los procesos de radicalización?
- ¿el plan estratégico afecta organizaciones cívicas, sociales o entidades religiosas?

2.2. Seguridad pública

- ¿La estrategia tiene un impacto en la seguridad de los individuos?
- ¿La estrategia produce efectos predominantemente sobre grupos en riesgo?

2.3. Terrorismo y seguridad

This project has received funding from the European Union’s Horizon 2020 research and innovation programme under grant agreement N° 699824.

- ¿La estrategia tiene un efecto en la seguridad? ¿en concreto, en delitos de terrorismo?
- ¿La estrategia aumenta la probabilidad de detectar casos de radicalización?
- ¿La estrategia aumenta las capacidades de los cuerpos policiales para contrarrestar el terrorismo?

DELINCUENCIA ORGANIZADA

INTRODUCCIÓN: la UE ha elaborado planes de acción, programas y estrategias ya sean para combatir la delincuencia organizada en general como para lidiar con sus formas específicas. La delincuencia organizada es una prioridad en la Agenda Europea de Seguridad. No obstante, los máximos logros seguidos por esta estrategia son medidas de lavado de dinero o creación de unidades de inteligencia financiera o recuperación de activos. De modo que no existen medidas concretas relacionadas con asuntos religiosos. Son básicamente cambios en el código penal y estrategias de inteligencia para impedir el asentamiento de los grupos criminales organizados y poner a disposición de la justicia a los que ya operan dentro de nuestras fronteras.

¿Tiene conocimiento sobre protocoles específicos en materia de religión que se estén aplicando para evitar reclutamiento en bandas de delincuencia organizada?
¿Hay medidas/ protocolos concretos en temas de religión que se esté usando para la prevención de la delincuencia organizada, aunque ese no sea su objetivo principal?

This project has received funding from the European Union's Horizon 2020 research and innovation programme under grant agreement N° 699824.

QUESTIONNAIRE UK – EXPERT for the field of IMMIGRATION

The document contains:

a) Background information about organised crime and terrorism policies in the United Kingdom
b) Questionnaire to the expert for the field of immigration

BACKGROUND INFORMATION ABOUT ORGANISED CRIME AND TERRORISM POLICIES IN THE UNITED KINGDOM

1. Terrorism policies and legal framework

1.1. Criminal and procedural code

The United Kingdom has a long tradition in Terrorism strategies, protocols and legislation, before and since the attacks of the 9/11 in the United States.

The firsts acts defining terrorism as a criminal offence were expressed in the Prevention of Terrorism (Temporary Provisions) Act 1989, which was one of the Prevention of Terrorism Acts of the United Kingdom related to the troubles in Northern Ireland. Subsequently, further provisions were created, and some of them, such as the Act 2000 and following received some controversial discussions and statements for being against the European Convention on Human Rights.

The Act 2000 has been described as the centrepiece of the United Kingdom's legislation on terrorism, but at the same time, some measures such as the stop-and-search powers under section 44 of the Act have been ruled illegal by the European Court of Human Rights. In response to the terrorist attacks on 11 September 2001, further counter terrorism powers were introduced under the Anti-Terrorism, Crime and Security Act 2001 ("the ATCS Act"). However, the introduction of some procedural changes received many controversial discussions. The most recent legislation is the Act 2015[20], which introduced once more a critical point: the statutory duty of reporting cases of radicalization to school staff, local authorities, prison personnel and NHS trusts[21].

1.2. Strategic Plans

[20] http://www.legislation.gov.uk/ukpga/2015/6/part/1/chapter/2
[21] This statutory duty is very controversial for the fact that teachers and other staff have not the training or knowledge enough about radicalization to report suspects.

This project has received funding from the European Union's Horizon 2020 research and innovation programme under grant agreement N° 699824.

The Prevent strategy, published by the Government in 2011, is part of the overall counter-terrorism strategy, CONTEST[22]. The aim of the Prevent strategy is to reduce the threat from terrorism by stopping people becoming terrorists or supporting terrorism. In the Act this has simply been expressed as the need to "prevent people from being drawn into terrorism".

The most recent strategy in the United Kingdom is the Counter-Extremism Strategy 2015. The strategy response is focused in four areas. First, countering extremism ideologies with the aim of confronting and challenging extremist propaganda and ensuring no space goes uncontested.

Second, building a partnership with all those opposed to extremism standing with and building the capacity of mainstream individuals, community organisations and others in our society who work every day to challenge extremists and protect vulnerable individuals. This point has received many critiques because includes the *statutory Prevent duty, so that all local authorities, schools, universities and colleges, NHS Trusts and Foundation Trusts, police, probation services and prisons are clear that they must take action to prevent people being drawn into terrorism*[23].

Thirdly, disrupt extremism creating new mechanisms or revising existing ones. For example, in areas of asylum, citizenship and immigration; helping the public to report extremism;
Finally, promoting more cohesive communities through measures such as the National Citizen Service helping young people to become more active and responsible citizens[24]; providing English language training; enforcing the disappearance of illegal cultural practices. For example, tackling Violence against women and girls delivering comprehensive programmes to prevent Female Genital Mutilation or forced marriage. Finally, one of the cohesive measures is the promotion of more opportunities in terms of housing, health and education. These measures will rely on the Casey review[25].

In this guide it is also introduced the controversial definition of extremism:

> *"vocal or active opposition to fundamental British values, including democracy, the rule of law, individual liberty and mutual respect and tolerance of different faiths and beliefs. We also include in our definition of extremism calls for the death of members of our armed forces".*

[22] https://www.gov.uk/government/uploads/system/uploads/attachment_data/file/97994/contest-summary.pdf
[23] The prevent duty applied in schools has been one of the most controversial points of the strategy. There are several documents addressing this. For example : Preventing education ? Human rights and CT in schools (Rights Watch UK).
[24] In the strategy is affirmed that there is an evaluation of this service called: Evaluation of National Citizen Service, July 2013, NatCen Social Research, Office of Public Management and New Philanthropy Capital.
[25] https://www.gov.uk/government/uploads/system/uploads/attachment_data/file/575973/The_Casey_Review_Report.pdf

This project has received funding from the European Union's Horizon 2020 research and innovation programme under grant agreement N° 699824.

What is understand as fundamental British values is subjective and leading to confusion. It has been criticized by many academics such as the expert in education interviewed for the fieldwork in UK.

Another polemic point in the prevent strategy is definition as a factor to detect individuals in risk of being drawn into terrorism. This includes not just violent extremism but also non-violent extremism, which can create an atmosphere conducive to terrorism and can popularise views which terrorists exploit.

In the prevent strategy is detailed the process to be followed by the local authorities to prevent cases of extremism. From the training to the report of the cases, as well as the Office for Standards in Education, Children's Services and Skills (Ofsted) in charge of inspecting that schools are accomplishing with fundamental British values.

There is another document focused on the training of the local authorities aimed at this goal. It is the Channel Duty Guidance: Protecting vulnerable people from being drawn into terrorism. Channel is the program to which the people suspected of being radicalised are referred to. The Channel strategy was first piloted in 2007 and rolled out across England and Wales in April 2012. Channel is a programme which focuses on providing support at an early stage to people who are identified as being vulnerable to being drawn into terrorism. The programme uses a multi-agency approach to protect vulnerable people by: a. identifying individuals at risk; b. assessing the nature and extent of that risk; and c. developing the most appropriate support plan for the individuals concerned. The Channel duty guide lists the functioning of the program and also the authorities' eligible to take part of the program. The Channel program is important to consider as one of the main measures of the counter-extremism strategy because it includes the cooperation between many local actors and allies in a grassroots level. For instance, organization, which work with the most vulnerable population.

There is another document reviewing the actions and methodology to be followed in cases of detection of extremism. This document is called Counter-terrorism local profiles: an updated guide[26]. In this document is affirmed that integration is an important part in the prevention of terrorism.

> *'Creating the Conditions for Integration' which outlines the Government's approach for creating an integrated society. The report highlights the role of local authorities in promoting integration and challenging extremism. However, at the same time, it seems like the Channel program is a government intelligence strategy, gathering intelligence from different sources.*

2. ORGANISED CRIME POLICIES AND LEGAL FRAMEWORK

[26] https://www.gov.uk/government/uploads/system/uploads/attachment_data/file/118203/counter-terrorism-local-profiles.pdf

This project has received funding from the European Union's Horizon 2020 research and innovation programme under grant agreement N° 699824.

2.1. Criminal and procedural code

Offences committed by joint offenders in prosecution of common purpose 20. When two or more persons form a common intention to prosecute an unlawful purpose in conjunction with one another and in the prosecution of such purpose, an offence is committed of such a nature that its commission was a probable consequence of the prosecution of such purpose, each of them is deemed to have committed the offence.

In the Serious and Organised Crime Strategy (2013) organised crime is explained that there is no legal definition of organised crime in England and Wales. However, in this strategy, organised crime is serious crime planned, coordinated and conducted by people working together on a continuing basis. Their motivation is often, but not always, financial gain.

2.2. Strategic Plan

The United Kingdom is the only country for our fieldwork offering a specific strategy to combat organised crime, which is called serious and Organised Crime Strategy (2013). The strategy is following the framework developed for Counter- terrorism, focusing in four areas: pursue (Prosecuting and disrupting serious and organised crime), prevent (Preventing people from engaging in serious and organised crime), protect (Increasing protection against serious and organised crime) and prepare (Reducing the impact of serious and organised crime).

This strategy introduces for the first time Prevent programmes for serious and organised crime, in England and Wales. According to the strategy achieving success in PREVENT would mean that: fewer people engage in serious and organised criminal activity; and to reduce reoffending by people convicted for serious and organised crime. The specific objectives are the following:

i. Deter people from becoming involved in serious and organised crime by raising awareness of the reality and consequences
ii. Use interventions to stop people being drawn into different types of serious and organised crime
iii. Develop techniques to deter people from continuing in serious and organised criminality
iv. Establish an effective offender management framework to support work on Pursue and Prevent

It is explained in the strategy that prevention will include better education and communications about organized crime, local coordination with existing work on troubled families and gangs, and wider use of interventions (e.g. Serious Crime Prevention Orders).
The strategy rely on local powers, in other words, a local network of prevention related to terrorism.

This project has received funding from the European Union's Horizon 2020 research and innovation programme under grant agreement N° 699824.

Organised crime Prevent work should also be coordinated with work on preventing terrorism. It may be possible in some areas for the same local team to take responsibility for both issues.

Main actions to stop people being drawn into OC:

- The Troubled Families Programme: focused on families that have multiple problems
- The ending gang and youth violence programme: prevent young people from becoming involved in street gangs as a previous step to enter OC groups.
- Preventing child sexual exploitation

In the strategy there are some statements could be interesting for our research. For example:

- About 50% of the international drug trafficking groups of interest to the US Department of Justice are associated with terrorist organisations.
- Crime groups can have a corrosive impact on the fabric and cohesion of communities. The abuse and exploitation of children can have a lifelong and devastating impact on victims.

- Over half of the organised crime groups operating against the UK are involved in drug-related crime; a significant proportion are also involved in violent crime.

QUESTIONNAIRE UK – EXPERT for the field of IMMIGRATION

We would like to know a little bit more about the serious and organized crime strategy, and also the prevent duty guidance against CT and how these strategies contain measures and actions, which can have a particular impact on the most vulnerable communities, such as ethnic minorities.

On the one hand we would like to talk about your perception of statements as the following:

- The majority of illegal migrants will rely on the services of organised crime groups at some point in their journey or during their time in the UK.
- The NCA will work closely with the Border Force which is responsible for detecting threats and seizing illicit goods, checking immigration status, searching baggage, vehicles and cargo for illicit goods or illegal immigrants, patrolling the UK coastline and searching vessels.

What is your perception of the protocols tackling irregular migration?

  - Does the government consider migrant communities/ civil society organizations to elaborate

This project has received funding from the European Union's Horizon 2020 research and innovation programme under grant agreement N° 699824.

and introduce the measures?

- Does the program foresee tools or mechanisms to evaluate the impact? And the efficacy?
- Does the strategy have received comments of disagreement? Or controversial discussions?
- What fundamental rights are affected?
  - Dignity
  - Personal data (processing of personal data, right and access to rectification?
  - Expulsion or extradition
  - Rights of the child? In case of unaccompanied minors
  - Working conditions (examples such as top manta in Spain, without a regular status in the country and with trouble to find a job)

Do you think that some of the prevent programs of the serious and organsied crime strategy, such as the Troubled Families Programme and the Ending Gang and Youth Violence Programme are being implemented focusing mainly to specific regions in the UK? Or to specific communities? Do you know if some of these protocols are mainly focus to migrant communities?

- Does the government consider migrant communities/ civil society organizations to elaborate and introduce the measures?

What kind of preventive measures is the government using to decrease discrimination/ racism?

What kind of barriers are migrants facing to integration?

- Including restrictions attached to their immigration status, hostile public attitudes and discrimination.

What kind of facilitators are migrants facing to integration?

- Facilitators: education, role models, work permits

Regarding to the Prevent strategy against CT:

Does the implementation of prevention strategies led to the creation of "suspect communities" on grounds of religion or belief?[27]

- Does the government consider migrant or religious communities/ civil society organizations to elaborate and introduce the measures?
- Does the option promote different treatment of groups or individuals on grounds of religion or belief?
- Do some measures affect freedom of thought, conscience or religion?
- Does the strategy have received comments of disagreement? Or controversial discussions?
- What fundamental rights are affected?

[27] **According to the Lit review Alam & Husband (2013):** Muslims saw **PREVENT strategies** as an assault on their integrity as law-abiding citizens; and Muslim are being exploited to further intrusive policies of control and surveillance.

This project has received funding from the European Union's Horizon 2020 research and innovation programme under grant agreement N° 699824.

- Dignity
- Personal data (processing of personal data, right and access to rectification?
- Expulsion or extradition
- Rights of the child? In case of unaccompanied minors
- Working conditions (examples such as top manta in Spain, without a regular status in the country and with trouble to find a job).

This project has received funding from the European Union's Horizon 2020 research and innovation programme under grant agreement N° 699824.

8 POLICY MAKERS' CONTRIBUTION

HUJI promoted the interaction between policy makers and PROTON researchers to target on the needs and requirements of policy authorities at different levels. For doing so, scientific and policy partners collaborated to establish a common approach to the analysis of the factors leading to recruitment in terrorist networks, and to integrate policy makers' perspectives into the final outputs of the studies.

In particular, representatives of Brå, DPPS, EUCPN, EUROPOL, UNODC and WODC received the outlines of activities and project proposals (feedforward process, M3-M4) and the draft reports on innovative studies of WP2 (feedback process, M11-M12), and they provided multidisciplinary comments and recommendations on task T2.3 to T2.7.

Moreover, policy makers commented and discussed the reports on preliminary finding of WP2 during the First Consortium meeting, hosted by The Hebrew University of Jerusalem on October 16th-17th 2017. Afterwards, they submitted a summary of their recommendations.

Feedback and feedforward comments were gathered in a single document by the project Coordinator and they were shared with WP2's tasks leaders. The following section briefly summarises their main contributions to the innovative studies of WP2; the original comments can be consulted in the abovementioned document.

Summary

Feedforward process

EUROPOL (comments submitted on December 22nd 2016):

- The results of the WP, in particular of T2.3, should be applicable to a wider, European geopolitical context.

Brå (comments submitted on December 22nd 2016):

- Researchers should evaluate the applicability of the study on security prisoners in Israel to the general context of the European Union;
- Recruitment in organised crime groups and recruitment in terrorist networks should be defined separately.

WODC (comments submitted on December 22nd 2016):

- Examined the utility of using cases of radicalization and recruitment into terrorism in the Israeli context in order to have a general understanding of the phenomenon;

This project has received funding from the European Union's Horizon 2020 research and innovation programme under grant agreement N° 699824.

– Asked for an in-depth explanation of the theoretical framework of the study and gave some suggestions for future lines of research.

HUJI sent clarifications to policy makers. Pertinent comments and recommendations were properly considered and integrated into the research.

Feedback process

EUROPOL (summary of recommendations submitted on October 26th 2017):

– The studies on terrorism should be translated into observations/questions over the recruitment phase;
– Researchers should reconsider the applicability of the ABM to a wider, not country- or region-specific context;
– The dynamic nature of terrorism should be considered and captured in the ABM.

UNODC (summary of recommendations submitted on October 24th 2017):

– The terms used to define radicalisation and recruitment in the context of terrorism should be appropriately reviewed and clarified.

BRA (summary of recommendations submitted on November 13th 2017):

– Researchers should emphasize how the results of their studies will contribute to the ABM and the data simulations;
– The results of the different studies of WP2 should not be generalised.

EUCPN (summary of recommendations submitted on November 13th 2017):

– Different ABMs could be created, due to the distinctive features of terrorism and OC;
– ABMs referring to security prisoners in Israel may not be applicable to the European context.

WODC (summary of recommendations submitted on October 16th 2017):

– Researchers should stress the fact that threats must be identified on a case-by-case basis, to avoid that policy makers apply a binary approach to the subject;
– Asked for an in-depth explanation of the theoretical framework of the study on societal impacts of terrorism policies;
– Suggested to provide a stricter definition of "counter-terrorism policy".

This project has received funding from the European Union's Horizon 2020 research and innovation programme under grant agreement N° 699824.

Conclusions

Task 2.8 established a systematic process of interaction between policy and scientific partners and allowed policy makers' views and requirements to be properly considered and integrated into D2.1.

This project has received funding from the European Union's Horizon 2020 research and innovation programme under grant agreement N° 699824.
